# The prokaryotic antecedents of the ubiquitin-signaling system and the early evolution of ubiquitin-like β-grasp domains

**DOI:** 10.1186/gb-2006-7-7-r60

**Published:** 2006-07-19

**Authors:** Lakshminarayan M Iyer, A Maxwell Burroughs, L Aravind

**Affiliations:** 1 National Center for Biotechnology Information, National Library of Medicine, National Institutes of Health, Bethesda, Maryland 20894, USA; 2 Bioinformatics Program, Boston University, Cummington Street, Boston, Massachusetts 02215, USA

## Abstract

**Background:**

Ubiquitin (Ub)-mediated signaling is one of the hallmarks of all eukaryotes. Prokaryotic homologs of Ub (ThiS and MoaD) and E1 ligases have been studied in relation to sulfur incorporation reactions in thiamine and molybdenum/tungsten cofactor biosynthesis. However, there is no evidence for entire protein modification systems with Ub-like proteins and deconjugation by deubiquitinating enzymes in prokaryotes. Hence, the evolutionary assembly of the eukaryotic Ub-signaling apparatus remains unclear.

**Results:**

We systematically analyzed prokaryotic Ub-related β-grasp fold proteins using sensitive sequence profile searches and structural analysis. Consequently, we identified novel Ub-related proteins beyond the characterized ThiS, MoaD, TGS, and YukD domains. To understand their functional associations, we sought and recovered several conserved gene neighborhoods and domain architectures. These included novel associations involving diverse sulfur metabolism proteins, siderophore biosynthesis and the gene encoding the transfer mRNA binding protein SmpB, as well as domain fusions between Ub-like domains and PIN-domain related RNAses. Most strikingly, we found conserved gene neighborhoods in phylogenetically diverse bacteria combining genes for JAB domains (the primary de-ubiquitinating isopeptidases of the proteasomal complex), along with E1-like adenylating enzymes and different Ub-related proteins. Further sequence analysis of other conserved genes in these neighborhoods revealed several Ub-conjugating enzyme/E2-ligase related proteins. Genes for an Ub-like protein and a JAB domain peptidase were also found in the tail assembly gene cluster of certain caudate bacteriophages.

**Conclusion:**

These observations imply that members of the Ub family had already formed strong functional associations with E1-like proteins, UBC/E2-related proteins, and JAB peptidases in the bacteria. Several of these Ub-like proteins and the associated protein families are likely to function together in signaling systems just as in eukaryotes.

## Abstract 2

A systematic analysis of prokaryotic ubiquitin-related beta-grasp fold proteins provides new insights into the Ubiquitin family functional history.

## Background

**Figure 1:**
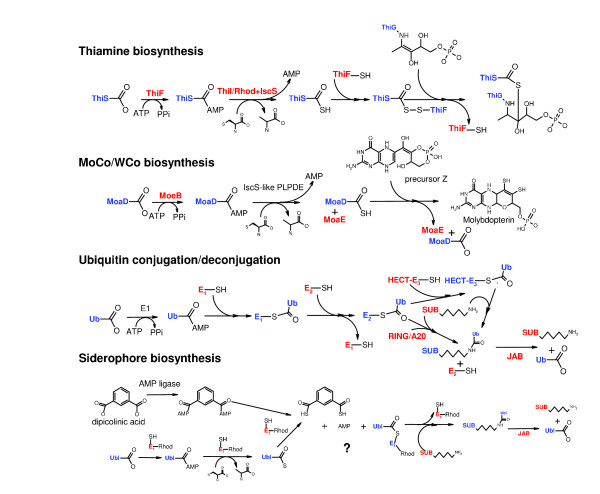
ThiS/MoaD/Ubiquitin-based protein conjugation system. The figure shows different themes by which a ThiS/MoaD/Ubiquitin-like polypeptide participates in thiamine biosynthesis, MoCo/WCo biosynthesis, and the ubiquitin conjugation/deconjugation system and the siderophore biosynthesis pathways. The '?' refers to the speculated part of the pathway inferred from operon organization. SUB refers to the polypeptide/protein substrate.

The ubiquitin (Ub) system is one of the most remarkable protein modification systems of eukaryotes, which appears to distinguish them from model prokaryotic systems. The modification of proteins by Ub or related polypeptides (Ubls) has been detected in all eukaryotes studied to date and is comprised of conserved machineries that both add Ub and remove it [1,2]. The Ub-conjugating system consists of a three-step cascade beginning with an E1 enzyme that uses ATP to adenylate the terminal carboxylate of Ub/Ubl and subsequently transfers this adenylated intermediate to a conserved internal cysteine in the form of a thioester linkage. The E1 enzyme then transfers this cysteine-linked Ub to the conserved cysteine of the E2 enzyme, which is the next enzyme in the cascade. Finally, the E2 enzyme transfers the Ub/Ubl to the target polypeptide with the help of an E3 enzyme [1,3]. The E3 enzymes of the HECT domain superfamily contain a conserved internal cysteine, which accepts the Ub/Ubl through a thioester linkage and finally transfers it to the ε-amino group of a lysine on the target protein. The E3 ligases of the treble-clef fold, namely the RING and A20 finger superfamilies, appear to facilitate directly the transfer of Ub to the lysine of target protein, without forming a covalent link with Ub/Ubl (Figure 1) [4,5].

The proteins modified by ubiquitination might have different fates depending both on the specific Ub or Ubl used, and the type of modification they undergo [6,7]. Mono-ubiquitination and poly-ubiquitination via G76-K63 linkages play regulatory roles in diverse systems such as signaling cascades, chromatin dynamics, DNA repair, and RNA degradation. Poly-ubiquitination via G76-K48 linkages is one of the major types of modification that results in targeting the polypeptide for proteasomal degradation [7]. Other polyubiquitin chains formed by linkages to K29, K6, and K11 are relatively minor species in model organisms and are poorly understood in functional terms. Similarly, modification by Ubls such as SUMO, Nedd8, URM1, Apg8/Apg12, and ISG15 have specialized regulatory roles in the context of chromatin dynamics, RNA processing, oxidative stress response, autophagy, and signaling [8,9]. The Ub modification is reversed by a variety of deubiquitinating peptidases (DUBs) belonging to various superfamilies of the papain-like fold and pepsin-like, JAB, and Zincin-like metalloprotease superfamilies [10-16]. Of these the most conserved are certain versions of the papain-like fold and the JAB superfamily metallo-peptidases, which are components of the proteasomal lid and signalosome [17-20]. The JAB peptidases are critical for removing the Ub chains before the targeted proteins are degraded in the proteasome [21,22].

Although the entire Ub system with the apparatus for conjugation and deconjugation has only been observed in the eukaryotes, several structural and biochemical studies have thrown light on prokaryotic antecedents of this system. Most of these studies are related to the experimental characterization of the key sulfur incorporation steps in the biosynthetic pathways for thiamine and molybdenum/tungsten cofactors (MoCo/WCo). Both these pathways involve a sulfur carrier protein, ThiS or MoaD, which is closely related to the eukaryotic URM1 and bears the sulfur in the form of a thiocarboxylate of a terminal glycine, just as the thioester linkages of Ub/Ubls formed in the course of their conjugation [23,24]. Furthermore, both ThiS and MoaD are adenylated by the enzymes ThiF and MoeB, respectively, prior to sulfur acceptance from the donor cysteine [25-29]. ThiF and MoeB are closely related to the Ub-conjugating E1 enzymes, and all of them exhibit a characteristic architecture, with an amino-terminal Rossmann-fold nucleotide-binding domain and a carboxyl-terminal β-strand-rich domain containing conserved cysteines [25]. Interestingly, in the case of the thiamine pathway, it has been shown that ThiS also gets covalently linked to a conserved cysteine in the ThiF enzyme, albeit via an acyl-persulfide linkage, unlike the direct thioester linkage of the E1-Ub covalent complex [26,27] (Figure 1). However, no equivalent covalent linkage between MoaD and MoeB has been reported [30] (Figure 1). There are other specific similarities between the eukaryotic Ub/Ubls and ThiS/MoaD, such as the presence of a conserved carboxyl-terminal glycine and the mode of interaction with their respective adenylating enzymes [23,25]. These observations indicated that core components of the eukaryotic Ub-signaling system and the interactions between them were already in place in the prokaryotic sulfur transfer systems, and implied direct evolutionary connection between them [25,31].

Homologs of other central components of the eukaryotic Ub-signaling pathway have also been detected in bacteria, such as the TS-N domain found in prokaryotic translation factors, which is the precursor of the helical Ub-binding UBA domain [32-34]. Similarly, members of the papain-like fold, zincin-like metallopeptidases, and the JAB domain superfamilies are also abundantly represented in prokaryotes [10-16,35]. However, to date there is no reported evidence of functional interactions of any of the prokaryotic versions of these domains with endogenous co-occurring counterparts of Ub/Ubls and their ligases in potential pathways analogous to eukaryotic Ub signaling. Thus, despite a reasonably clear understanding of the possible precursors of Ub/Ubls and the E1 enzymes, the evolutionary process by which the complete eukaryotic Ub-signaling system as an apparatus for protein modification was pieced together remains murky. To address this problem we conducted a systematic comparative genomic analysis of the Ub-like (also referred to as the β-grasp fold in the SCOP database [36]) fold in prokaryotes to decipher its early evolutionary radiations. We then utilized the vast dataset of contextual information derived from newly sequenced prokaryotic genomes to identify systematically the potential functional connections of the relevant members of the Ub-like fold and other functionally associated enzymes such as the E1/MoeB/ThiF (E1-like) family.

As a result of this analysis we were able to identify several new members of the Ub-like fold in prokaryotes as well as functionally associated components such as E1-like enzymes, JAB hydrolases, and E2-like enzymes, which appear to interact even in prokaryotes to form novel pathways related to eukaryotic Ub signaling. We not only present evidence that there are multiple adenylating systems of Ub-related proteins in prokaryotes, but also we predict intricate pathways using JAB-like peptidases and E2-like enzymes in the context of diverse Ub-related proteins.

## Results and discussion

### Identification of novel prokaryotic ubiquitin-related proteins

We investigated the origin of Ub and the Ub signaling system as a part of a comprehensive investigation into the evolutionary history of the Ub-like (β-grasp) fold (unpublished data). Earlier studies had shown that ThiS and MoaD are the closest prokaryotic relatives of the eukaryotic Ub/Ubls both in structural and in functional terms [27,28]. Structural similarity-based clustering using the pair-wise structural alignment Z-scores derived from the DALI program, as well morphologic examination of the structures, showed that several additional members of the β-grasp fold prevalent in prokaryotes are equally closely related to the eukaryotic Ub/Ubls. The most prominent of these was the RNA-binding TGS domain, which was previously reported by us as being fused to several other domains in multidomain proteins such as the threonyl tRNA synthetase, OBG-family GTPases, and the SpoT/RelA like ppGppp phosphohydrolases [37] (also see SCOP database [36]). The β-grasp ferredoxin, a widespread metal-chelating domain, is also closely related, but it is distinguished by the insertions of unique cysteine-containing flaps within the core β-grasp fold that chelate iron atoms [38]. Other versions of the β-grasp fold closely related to the Ub-like proteins are the subunit B of the toluene-4-mono-oxygenase system (for example, PDB: 1t0q) [39], which is sporadically encountered in several proteobacteria and actinobacteria, and the YukD protein of *Bacillus subtilis *and related bacteria (PDB: 2bps) [40] Table 1.

In order to identify novel prokaryotic Ub-related members of the β-grasp fold we initiated transitive PSI-BLAST searches, run to convergence, using multiple representatives from each of the above mentioned structurally characterized versions. Searches with the TGS domains and ThiS or MoaD proteins were considerably effective in recovering diverse homologs with significant expect (e) values (e ≤ 0.01). Searches from these starting points were reasonably symmetric; thus, searches initiated with various ThiS or MoaD proteins detected eukaryotic URM1, representatives of the TGS domain, as well as the β-grasp ferredoxins. Likewise, searches initiated with different representatives of the TGS domains also recovered ThiS, MoaD, and representatives of the β-grasp ferredoxins. These searches also recovered several previously uncharacterized prokaryotic proteins in addition to the above-stated previously known representatives of the Ub-like fold. These included several divergent small proteins equally related to both ThiS and MoaD, the amino-terminal regions of a group of ThiF/MoeB-related (E1-like) proteins from various bacteria, the amino-terminal regions of a family of bacterial RNAses with the Mut7-C domain, the amino-terminal region of the family of tail assembly protein I of the lambdoid and T1-like bacteriophages, and the RnfH family, which is highly conserved in numerous bacteria.

For example, searches initiated with the *Thermus thermophilus *MoaD homolog (gi: 46200137) recovered the tail protein I of the diverse caudate bacteriophages belonging to the lambda and T1 groups (for example, lambda tail protein I, e = 10^-3^, iteration 2). A search using the *Desulfovibrio desulfuricans *MoaD homolog (gi: 78219906) recovered the amino-terminal domains of an *Azotobacter *Mut7-C RNase (e = 10^-8^, iteration 2; gi: 67154055), the TGS domain of *Chlamydophila *threonyl tRNA synthetase (iteration 3, e = 10^-3^; gi: 15618715), RnfH from *Azoarcus *(iteration 3, e = 10^-3^; gi: 56312934), and a E1-like protein from *Campylobacter jejuni *(e = 0.01, iteration 11; gi: 57166736). Searches with the YuKD protein from low GC Gram-positive bacteria consistently recovered a homologous domain in large actinobacterial membrane proteins (e = 10^-3^-10^-4 ^in iteration 4).

**Figure 2:**
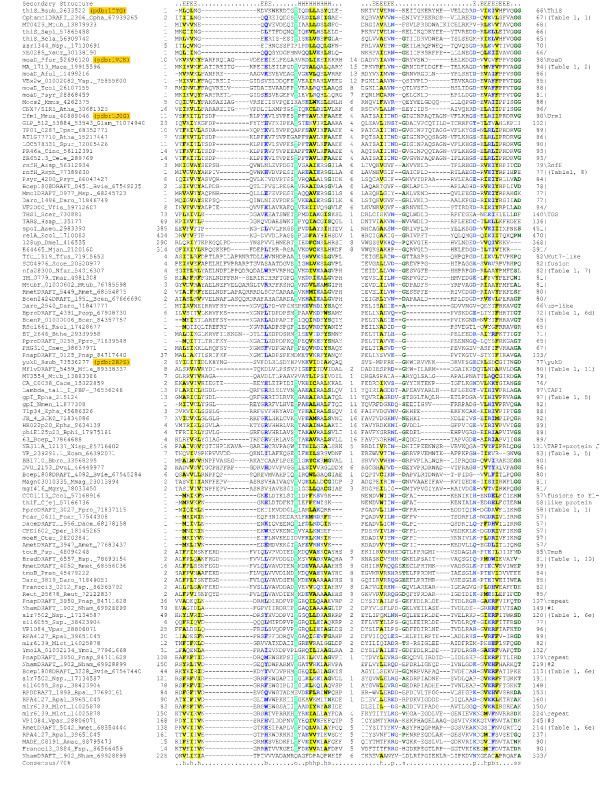
Multiple alignment of ThiS/MoaD-like ubiquitin domain containing proteins. Proteins are listed by gene name, species abbreviation and gi number, separated by underscores. Amino acid residues are colored according to side chain properties and the extent of conservation in the multiple alignment. Coloring is indicative of 70% consensus, which is shown on the last line of the alignment. Consensus similarity designations and coloring scheme are as follows: h, hydrophobic residues (ACFILMVWY), shaded yellow; s, small residues (AGSVCDN), colored green; o, alcohol group containing residues (ST), colored blue; and b, big residues (EFHIKLMQRWY), colored purple and shaded in light gray. Secondary structure assignments are shown above the alignment, where E represents a strand and H represents a helix. The families of the ubiquitin-related domains are shown to the right. Also shown to the right are the row numbers in Table 1, which describe a particular family. Species abbreviations are as follows: Aaeo, *Aquifex aeolicus*; Adeh, *Anaeromyxobacter dehalogenans*; Aehr, *Alkalilimnicola ehrlichei*; Aful, *Archaeoglobus fulgidus*; Amac, *Alteromonas macleodii*; Amet, *Alkaliphilus metalliredigenes*; Asp., *Arthrobacter *sp.; Azsp, *Azoarcus *sp.; Atha, *Arabidopsis thaliana*; Avar, *Anabaena variabilis*; BJK0, Bacteriophage JK06; Bbro, *Bordetella bronchiseptica*; Bcen, *Burkholderia cenocepacia*; Bcep, *Burkholderia cepacia*; Bcer, *Bacillus cereus*; Bcla, *Bacillus clausii*; Blic, *Bacillus licheniformis*, Bphi, Bacteriophage phiE125; Bsp., *Bradyrhizobium *sp.; Bsub, *Bacillus subtilis*; Bthe, *Bacteroides thetaiotaomicron*; Bthu, *Bacillus thuringiensis*; Bvie, *Burkholderia vietnamiensis*; Cace, *Clostridium acetobutylicum*; Caur, *Chloroflexus aurantiacus*; Ccol, *Campylobacter coli*; Cele, *Caenorhabditis elegans*; Cinc, *Chlamydomonas incerta*; Cjej, *Campylobacter jejuni*; Cnec, *Cupriavidus necator*; Cper, *Clostridium perfringens*; Cpha, *Chlorobium phaeobacteroides*; Csac, *Caldicellulosiruptor saccharolyticus*; Ctet, *Clostridium tetani*; Dace, *Desulfuromonas acetoxidans*; Daro, *Dechloromonas aromatica*; Dhaf, *Desulfitobacterium hafniense*; Dmel, *Drosophila melanogaster*; Dpsy, *Desulfotalea psychrophila*; Drad, *Deinococcus radiodurans*; Dvul, *Desulfovibrio vulgaris*; Ecol, *Escherichia coli*; Elit, *Erythrobacter litoralis*; Epha, Enterobacteria phage; Fsp., *Frankia *sp.; Glam, *Giardia lamblia*; Gmet, *Geobacter metallireducens*; Goxy, *Gluconobacter oxydans*; Gsul, *Geobacter sulfurreducens*; Gura, *Geobacter uraniumreducens*; Hsap, *Homo sapiens*; Hsp., *Halobacterium *sp.; Mace, *Methanosarcina acetivorans*; Maqu, *Marinobacter aquaeolei*; Mdeg, *Microbulbifer degradans*; Mfla, *Mycobacterium flavescens*, Mgry, *Magnetospirillum gryphiswaldense*; Mjan, *Methanocaldococcus jannaschii*; Mlot, *Mesorhizobium loti*; Mmag, *Magnetospirillum magnetotacticum*; Mmus, *Mus musculus*; Msp., *Magnetococcus *sp.; Mtub, *Mycobacterium tuberculosis*; Neur, *Nitrosomonas europaea*; Nfar, *Nocardia farcinica*; Nham, *Nitrobacter hamburgensis*; Nisp, *Nitrobacter *sp.; Nmen, *Neisseria meningitidis*; Nmul, *Nitrosospira multiformis*; Noce, *Nitrosococcus oceani*; Nosp, *Nocardioides *sp.; Nsp., *Nostoc *sp.; Nwin, *Nitrobacter winogradskyi*; Obat, *Oceanicola batsensis*; PBP-, Phage BP-4795; Paby, *Pyrococcus abyssi*; Paer, *Pseudomonas aeruginosa*; Parc, *Psychrobacter arcticus*; Pber, *Parvularcula bermudensis*; Pcar, *Pelobacter carbinolicus*; Pflu, *Pseudomonas fluorescens*; Pfur, *Pyrococcus furiosus*; Phor, *Pyrococcus horikoshii*; Pmen, *Pseudomonas mendocina*; Pnap, *Polaromonas naphthalenivorans*; Posp, *Polaromonas *sp.; Ppro, *Pelobacter propionicus*; Pput, *Pseudomonas putida*; Psp., *Pseudomonas *sp.; Psyr, *Pseudomonas syringae*; Retl, *Rhizobium etli*; Reut, *Ralstonia eutropha*; Rfer, *Rhodoferax ferrireducens*; Rmet, *Ralstonia metallidurans*; Rosp, *Roseovarius *sp.; Rpal, *Rhodopseudomonas palustris*; Rsol, *Ralstonia solanacearum*; RhNGR234a, *Rhizobium *sp. NGR234a plasmid; Rsp, *Rhizobium *sp. NGR234; Rsph, *Rhodobacter sphaeroides*; Rusp, *Ruegeria *sp.; Rxyl, *Rubrobacter xylanophilus*; Saci, *Syntrophus aciditrophicus*; Save, *Streptomyces avermitilis*; Scer, *Saccharomyces cerevisiae*; Scoe, *Streptomyces coelicolor*; Sdis, *Spisula solidissima*; Sepi, *Staphylococcus epidermidis*; Spom, *Schizosaccharomyces pombe*; Spur, *Strongylocentrotus purpuratus*; Srub, *Salinibacter ruber*; Ssol, *Sulfolobus solfataricus*; Ssp., *Synechocystis *sp.; Swsp, *Shewanella *sp.; Tfus, *Thermobifida fusca*; Tmar, *Thermotoga maritima*; Tpar, *Theileria parva*; Vcho, *Vibrio cholerae*; Vfis, *Vibrio fischeri*; Vpar, *Vibrio parahaemolyticus*; Vsp., *Vibrio *sp.; Wsuc, *Wolinella succinogenes*; Xaxo, *Xanthomonas axonopodis*; Xcam, *Xanthomonas campestris*; Ymol, *Yersinia mollaretii*; Ypes, *Yersinia pestis*.

We prepared individual multiple alignments of all of the novel families of proteins containing regions of similarity to the Ub-like β-grasp domains and predicted their secondary structures using the JPRED method, which combines information from Hidden Markov models (HMMs), PSI-BLAST profiles, and amino acid frequency distributions derived from the alignments. In each case the predicted secondary structure of the region detected in the searches exhibited a characteristic pattern with two amino-terminal strands, followed by a helical segment and another series of around three consecutive strands. This pattern is congruent with that observed in the Ub-like β-grasp proteins (see SCOP database [36]) and was used as a guide, along with the overall sequence conservation, to prepare a comprehensive multiple alignment that included all of the major prokaryotic representatives of the Ub-like β-grasp domains (Figure 2). Examination of the sequence across the different families revealed a similar pattern of hydrophobic residues that are likely to form the core of the β-grasp domain, as suggested by the structures of ThiS, MoaD and URM1, and a highly conserved alcohol group containing residue (serine or threonine) before helix-1. A similar secondary structure and conservation pattern was also found in two additional Ub-related protein families that we recovered using contextual information from analysis of gene neighborhoods and domain fusions (Figure 2; see the following two sections for details). Taken together, these observations strongly support the presence of an Ub-related β-grasp fold in all of the above-detected groups of proteins.

Like the ThiS, MoaD, and URM1 proteins, the phage tail assembly protein I (TAPI) and one of the other newly detected Ub-related families also exhibited a highly conserved glycine at the carboxyl-terminus of the β-grasp domain, suggesting that they might participate in similar functional interactions with other proteins or undergo thiolation (Figure 2). The remaining newly detected members, while exhibiting similar overall conservation to that of the above families, do not contain the glycine or any other highly conserved residue at the carboxyl-terminus of the domain. Individual families also possess their own exclusive set of highly conserved residues, suggesting that each might participate in their own specific conserved interactions with other proteins or nucleic acids.

### Identification of contextual associations of prokaryotic ubiquitin-related proteins and their functional partners

#### Detection of architectures and conserved gene neighborhoods

Different types of contextual information can be obtained by means of prokaryotic comparative genomics and used to elucidate functionally uncharacterized proteins. First, fusions of uncharacterized domains or genes to functionally characterized domains or genes suggest participation of the former in processes similar to those of the latter. Second, clustering of genes in operons usually implies coordinated gene expression, and conserved prokaryotic gene neighborhoods are a strong indication of functional interaction, especially through physical interactions of the encoded protein products. The power of contextual inference, especially for the less prevalent protein families, has been considerably boosted due to the enormous increase in data from the various microbial genome sequencing projects [41,42] and the development of publicly available resources such as WIT2/PUMA2 and STRING/SMART that integrate a variety of contextual information [43-46].

Accordingly, we set up a protocol to identify comprehensively the network of contextual connections centered on the prokaryotic Ub-related proteins detected in the above searches, and used it to infer the functional pathways in which they participate. We first determined the complete domain architectures of all the Ub-like proteins using a combination of case-by-case PSI-BLAST searches and searches against libraries of position specific score matrices (PSSMs) or HMMs of previously characterized protein domains. We then established the gene neighborhoods (see Materials and methods, below) for these Ub-like proteins and found a number of conserved neighborhoods containing genes for specific protein families often co-occurring with the Ub-like proteins. Each of the families belonging to the conserved neighborhoods were used as starting points for further PSI-BLAST searches to identify homologous proteins in prokaryotic genomes. These homologs were then used as foci to identify any conserved gene neighborhoods occurring with them. This way we built up a comprehensive set of conserved gene neighborhoods for the Ub-like proteins as well as their putative functional partners and their homologs, which were identified via contextual analysis. As a result we identified several persistent architectural and gene neighborhood themes associated with the prokaryotic Ub-like proteins. We discuss below the most prominent of these, especially those with relevance to the early evolution of the Ub-signaling related pathways.

#### Common architectural themes in prokaryotic ubiquitin-like proteins

**Figure 3:**
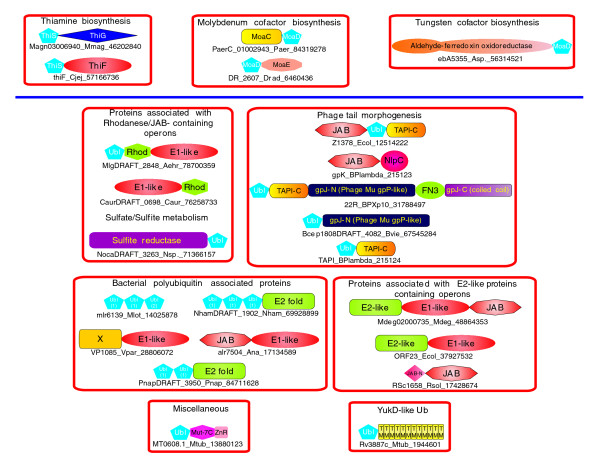
Domain architectures of ThiS/MoaD-like ubiquitin domains and functionally associated proteins. Architectures belonging to a particular gene neighborhood or related pathway are grouped in boxes. Proteins are identified below the architectures by gene name, species abbreviation and gi number, demarcated by underscores. Proteins belonging to the classical thiamine and MoCo/WCo biosynthesis pathways are shown above the purple line. Species abbreviations are listed in the legend to Figure 2. JAB-N, an α + β domain found amino-terminal to some JAB proteins; TAPI-C, domain found carboxyl-terminal to the phage λ-TAPI-like ubiquitin domain; Rhod, Rhodanese domain; X, β-strand rich, poorly conserved globular domain; ZnR, zinc ribbon domain.

Several families of prokaryotic Ub-like proteins, namely ThiS, MoaD, RnfH, TmoB, and a newly detected family typified by *Ralstonia solanacearum *RSc1661 (gi: 17428677; see below), are characterized by a single standalone Ub-like domain. In several cases the ThiS and MoaD are fused to ThiG and MoaE (Figure 3), which respectively are their functional partners in the transfer of sulfur to the substrates (Figure 1). We also noted that a distinct version of ThiS is fused to the carboxyl-terminus of the sulfite reductase in certain actinobacteria (for example, *Nocardiodes *and *Acidothermus cellulolyticus*), whereas MoaD might be fused to aldehyde ferredoxin oxidoreductase (*Azoarcus*; Figure 3). Another newly characterized family of Ub-domains typified by the protein mlr6139 from *Mesorhizobium loti *(gi: 14025878) is characterized by three tandem repeats of the Ub-like domain (Figure 3; see below for details).

A family of Ub-like domains, distinct from ThiS, is found fused to the amino-terminus of the adenylating Rossmann fold domain of certain ThiF proteins, such as that from *Campylobacter jejuni *(gi: 57166736; Figure 3). In the lambda and T1 phage TAPI proteins, the Ub-like domain is fused to another small globular carboxyl-terminal domain via a glycine-rich low complexity linker. In some cases the TAPI protein itself may be fused to the tail-assembly protein J (TAPJ) or K (TAPK), which contain two peptidase domains, namely the JAB domain and NlpC/P60 domain with the papain-like fold (Figure 3) [13].

In the proteins typified by the *Thermotoga maritima *TM_0779, the amino-terminal Ub-like domain is linked to a carboxyl-terminal Mut7-C RNAse domain and a zinc ribbon domain (Figure 3) [47]. Iterative sequence profile searches with the Mut7-C domain as a query recovered the previously characterized PIN (PilT-N) RNAse domains with significant e values (e < 10^-3^). The two domains share an identical pattern of conserved catalytic residues, suggesting a similar enzymatic mechanism [48]. In the actinobacteria, the YukD-like β-grasp domain is fused to an integral membrane domain with 12 transmembrane helices (Figure 3). The TGS domain, as previously reported, was almost always found in various RNA-binding multidomain proteins; hence it is not discussed here in detail [37]. Likewise, the architectures of β-grasp ferredoxins, which are typically found as a part of multidomain oxido-reductases, have previously been considered in depth and are not dwelt upon in detail here [49].

#### Conserved gene neighborhoods related to the thiamine biosynthesis pathway

The multistep biosynthetic pathways for the major cofactor thiamine is the experimentally best characterized of the prokaryotic systems involving Ub-like sulfur transfer proteins and associated E1-like enzymes. Furthermore, there has also been a comprehensive comparative genomics analysis of the components of the prokaryotic thiamine biosynthetic pathway [50]. In the present report we focus only on associations in these systems that are pertinent to the evolution of the Ub-signaling related pathways and previously unnoticed features of the distribution and gene neighborhoods of the ThiS genes.

**Figure 4:**
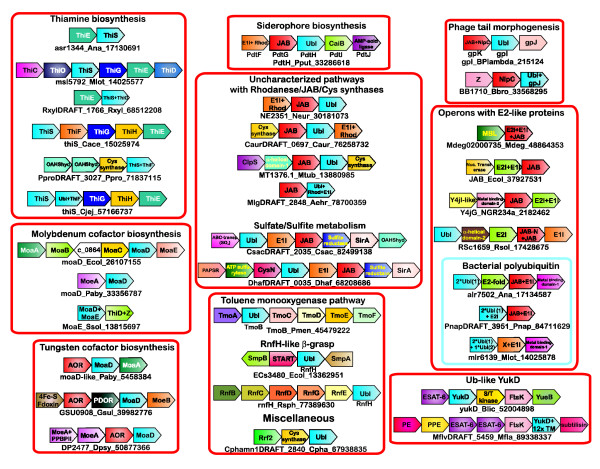
Gene neighborhoods of prokaryotic ThiS/MoaD-like ubiquitin domains and functionally associated proteins. Genes found in conserved neighborhoods are depicted as boxed arrows with the arrow head pointing from the 5' to the 3' direction. ThiS/MoaD-like proteins are shaded in blue. Other than in the classical ThiS and MoaD pathways, ThiS/MoaD/Ubiquitin-like proteins are labeled Ubl for ubquitin-like domain. The ThiS/MoaD-like proteins in each operon are identified in black lettering below the neighborhood by gene name, species abbreviation and gi number, demarcated by underscores. In the instances where ThiS/MoaD-like domains are absent, the gene neighborhoods are identified by the JAB domain containing protein. Alternative names of experimentally well characterized genes are shown below the boxed arrows for that gene. Boxed arrows with no colors represent poorly conserved proteins. Conserved neighborhoods are clustered according to major assemblages of gene neighborhood as described in the text. In Sulfolobus MoaD and MoaE are intriguingly linked to ThiD, but any possible role in thiamine biosynthesis remains unclear. Species abbreviations are listed in the legend to Figure 2. AOR, aldehyde ferredoxin oxidoreductase; Cys Synthase, cysteine synthase; PE, PE family of proteins; PPE, PPE family of proteins;Rhod, Rhodanese domain; Z, poorly characterized protein with an α + β domain with several conserved charged residues; X, β-strand rich globular domain; YueB, bacillus YueB-like membrane associated protein.

The ThiS protein is highly conserved in all of the major bacterial and archaeal lineages, suggesting that it may be traced back to the last universal common ancestor (LUCA). In most bacterial lineages ThiS is encoded within a large operon including several other genes for thiamine biosynthesis. These include genes encoding proteins for both the major branches of the thiamine biosynthetic pathway (for instance, the aminoimidazole ribotide utilizing branch with ThiC and ThiD, and the sulfur transfer and hydroxyl-ethyl-thiazole forming branch with ThiS, ThiG, ThiO, ThiH) and the stem combining the products of branches to form thiamine phosphate (ThiE; Figure 4) [50].

Although the individual genes occurring in this conserved gene neighborhood exhibit some variability across different bacteria, ThiS is most strongly coupled with ThiG (approximately 80%) - its physically interacting functional partner within the operon. The next strongest coupling of ThiS in bacteria is with its other complex forming partner, namely the adenylating enzyme ThiF (approximately 20%). This is not surprising, given that ThiF and ThiG compete for ThiS to catalyze two successive steps in the sulfur incorporation process [25,51]. Very rarely, ThiS may also be coupled with ThiC (for example, *Cytophaga hutchinsonii*). The genes for the group of ThiF proteins containing a fused Ub-like domain at their amino-termini (see above) typically co-occur in predicted operons with standalone ThiS genes (Figure 4). This suggests that their fused Ub-like domain plays a role different from the standalone ThiS protein. However, in a single case (*Pelobacter propionicus*), the Ub-like domain-ThiF fusion proteins do not occur in an operon with other thiamine biosynthesis genes, instead co-occurring with O-acetylhomoserine sulfhydrylase and cysteine synthase (Figure 4). Similar operonic association of ThiS alone, or ThiS and ThiG with genes for cysteine biosynthesis such as cysteine synthase, and sulfite transporter genes are also seen in *Pelodictyon *and *Chlorobium *(Figure 4 and Additional data file 1). These represent multiple independent associations of thiamine biosynthetic genes with sulfur assimilation and cysteine biosynthesis genes, which is consistent with the fact that cysteine is the sulfur donor for the ThiS thiocarboxylate.

The genes of the archaeal ThiS orthologs are not found in any conserved gene neighborhoods, and this is consistent with the previously noted absence of ThiF and ThiG orthologs in the archaea, and the presence of an alternative branch for hydroxyl-ethyl-thiazole biosynthesis [50]. This observation suggests that the archaeal ThiS genes might even have been recruited for a sulfur transfer process distinct from thiamine biosynthesis.

#### Conserved gene neighborhoods related to molybdenum and tungsten cofactor biosynthesis

The MoaD-MoeB system in molybdenum and tungsten cofactor biosynthesis mirrors the ThiS-ThiF system in thiamine biosynthesis. MoaD is also conserved across all major archaeal and bacterial lineages, suggesting that it existed in the LUCA. Unlike ThiS, MoaD is present in Mo/W cofactor biosynthesis operons in both bacteria and archaea (Table 1). This implies that both ThiS and MoaD had probably diverged from each other by the time of the LUCA, but the recruitment of ThiS for a sulfur transfer system in thiamine biosynthesis emerged early in the bacterial lineage, only after it had split from the archaeal lineage. In contrast, the deployment of MoaD in Mo/W cofactor biosynthesis appears to have happened in the LUCA itself. The Mo/W cofactor biosynthesis operons from different bacteria encode a variety of proteins, including those involved in using the GTP precursor (MoaA and MoaC); the MoeB, MoaD and MoaE products, which are downstream of the former and involved in molybdopterin biosynthesis; and MoeE, MogA, MobD, and the MOSC domain proteins, which are involved in formation of MoCo/WCo and its terminal derivatives (Figure 4, Table 1 and Additional data file 1) [52-54]. Although the predicted operons exhibit variability across prokaryotes in terms of the different genes included in them, the core conserved gene neighborhood in bacteria contains the genes for MoaD and MoaE, which together constitute the molybdopterin (MPT) synthase, which transfers the sulfur from the MoaD thiocarboxylate to the precursor Z (cyclic pyranopterin monophosphate) to form MPT [52,55] (Figures 1 and 4). In a few cases MoaD may be adjacent to the gene for MoeA, which acts on the product downstream of the reaction catalyzed by the MPT synthase. MoaD, unlike ThiS, is rarely found immediately adjacent to the gene for its adenylating enzyme, MoeB (Figure 4). This distinction may be related to experimental results, which indicate that MoaD and MoeB do not form a covalently linked persulfide or thioester complex, unlike ThiS and ThiF or the Ub/Ubl and the E1s (Figure 1) [30].

A distinct set of MoaD genes are found strictly adjacent to genes encoding an aldehyde ferredoxin oxidoreductase (AOR) in a sporadic group of phylogenetically distant archaea and bacteria (Table 1), suggesting that they might constitute a mobile gene cluster. Additionally, these gene neighborhoods often include MoeB and occasionally other cofactor biosynthesis genes such as MoaA and MoaE, and a pyridine disulfide oxidoreductase in close vicinity to MoaD and the AOR genes (Figure 4). In some organisms this MoaD containing gene cluster is distinct from the MoCo biosynthesis operon found elsewhere in the genome of the same organism. Experimentally characterized versions of these AORs have been shown to utilize a tungsten-containing variant of the cofactor [56]. Taken together, these observations suggest that these AOR linked MoaD genes might specifically participate in the synthesis of molybdopterin for WCo generation for the AORs.

#### Other potential novel pathways involving ThiS/MoaD-like proteins and E1-like enzymes

**Figure 5:**
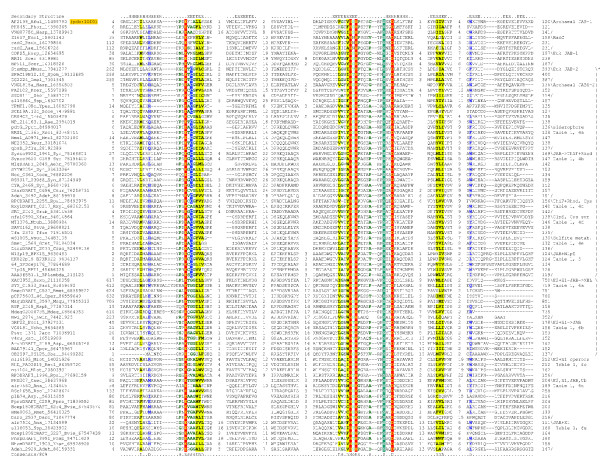
Multiple alignment of JAB domain containing proteins. Coloring is indicative of 80% consensus. The coloring scheme, consensus abbreviations and secondary structure representations are as described in the legend to Figure 2. The secondary structure, shown on the first line of the alignment, is derived from a JAB crystal structure whose primary sequence is found on the second line of the alignment, with PDB identifier shaded in gold. Conserved histidine and acidic residues (ED) are colored yellow and shaded in red. The conserved active site serine residue is colored light gray and shaded in teal. The conserved cysteine found in a subset of JABs (marked with an asterisk) are shaded blue and colored white. The alignment is grouped according to families, with family names listed to the right. Also provided are references to the appropriate row on Table 1, which describes a particular JAB containing operon.

Beyond the above-stated predicted operons, with the *bona fide *ThiS/MoaD and the ThiF/MoeB enzymes involved in conventional thiamine and MoCo/WCo biosynthesis, we also recovered several other predicted bacterial operons encoding homologous proteins. These gene clusters typically encode a ThiS/MoaD related protein and an E1-like enzyme related to ThiF/MoeB with a carboxyl-terminal rhodanese domain, but they do not contain any genes encoding other components of the two cofactor biosynthesis pathways (Figures 3 and 4, and Table 1). The bacteria that contain these predicted operons also contain independent thiamine or molybdenum operons, highlighting the functional distinctness of the pathways encoded by these gene neighborhoods (Table 1). Interestingly, this class of predicted operons also often contains a gene encoding a standalone version of the JAB metallopeptidase, which forms a monophyletic clade within the tree of all JAB domains (Figures 4 and 5; see Materials and methods, below, for details). There are at least five distinct subtypes of this class of gene neighborhoods, which exhibit a sporadic distribution across phylogenetically diverse bacteria, suggesting possible dispersion through lateral gene transfer (Table 1 rows 4a-4e and Figure 4). One of these subtypes of gene clusters has been shown to encode components of the biosynthetic pathway for the siderophores and secreted protective compounds PDTC (pyridine-2,6-bis[thiocarboxylic acid]) and quinolobactin in *Pseudomonas stutzeri*/*P. putida *and *P. fluorescens*, respectively [57,58]. Our analysis of gene neighborhoods revealed that related conserved gene neighborhoods are also found in several distantly related proteobacteria, such as *Ralstonia solanacearum *and *Nitrosomonas europaea*, suggesting that such compounds might be widely produced (Table 1 row 4a and Figure 4).

There are considerable differences in the genes and corresponding biosynthetic pathways (related to amino acid biosynthetic pathways) producing the basic molecular skeleton of each of these metabolites. For example, in the case of quinolobactin a xanthurenic acid skeleton is used, whereas in the case of PDTC a dipicolinic acid skeleton is used (Figure 1) [57,58]. However, all of these operons contain a conserved core of genes whose products catalyze the critical sulfurylation step required for the production of all of these compounds [57,58]. This core group encodes a carboxylate AMP ligase, which adenylates a carboxylate group on the precursor, and proteins for a sulfur transfer system that forms a thiocarboxylate group from the carboxy adenylate produced by the AMP ligase (Figure 1). The proteins of the sulfur transfer system include an E1-like protein with a carboxyl-terminal rhodanese domain, a ThiS/MoaD-like protein, and a protein with a JAB metallopeptidase domain (Figure 4). The first two enzymes are likely to participate in a sulfur transfer pathway similar to those seen in the conventional thiamine and MoCo/WCo pathways, with the rhodanese domain probably abstracting the sulfur from a small molecule donor such as cysteine (as in the case of ThiI), and the E1-like protein adenylating and transferring the sulfur to the ThiS/MoaD-like protein to form a terminal thiocarboxylate (Figure 1).

Most other predicted operon subtypes of this class appear to exhibit different variants of the core sulfur transfer system seen in the above-described siderophore biosynthesis gene clusters (Table 1 and Figure 4). A simple subtype seen in a wide range of bacteria contains just three genes encoding a ThiS/MoaD-like protein, a protein combining an E1-like module and a rhodanese domain, and JAB domain peptidase. Derivatives of this basic subtype might simply contain genes for the JAB domain peptidase and E1 + rhodanese protein (Table 1 row 4b and Figure 4). Another subtype additionally combines the cysteine synthase with the three genes of the basic operon, suggesting that they might couple sulfur transfer to production of the major cellular sulfur donor cysteine (Table 1 row 4c and Figure 4). A variant of the cysteine synthase containing operon subtype, which is particularly prevalent in the actinobacteria, includes ClpS that is involved in degradation of proteins through the Clp system and an uncharacterized helical protein that is almost exclusively encoded in this predicted operon subtype (Table 1 row 4d and Figure 4). Other links to sulfur metabolism are hinted at by another major subtype of this class of gene neighborhoods, where genes for the ThiS/MoaD, JAB, and E1-like proteins are combined with genes coding sulfite/sulfate ABC transporters, PAPS reductase, ATP sulfurylase, sulfite reductase, O-acetylhomoserine sulfhydrylase, and adenylylsulfate kinase. The E1-like protein of these predicted operons always lacks the carboxyl-terminal rhodanese-like domain. However, these gene neighborhoods always contain a SirA (cysteine containing domain 1 [CCD1]) protein, which was predicted to play a role similar to that of rhodanese [59] (Table 1 row 4e and Figure 4). These observations suggest that these gene clusters are principally involved in the assimilation of sulfur from sulfate/sulfite and that this sulfur might be terminally transferred to the ThiS/MoaD-like proteins encoded by them.

#### The tail assembly gene neighborhoods of Lambdoid and T1-like phages

The genomes of lambdoid and T1-like phages are known to contain related tail assembly gene complexes [60]. In a large number of phages this complex encodes a protein TAPI that contains an Ub-like domain related to ThiS/MoaD (Figure 2). The exact function of this protein tail assembly is unclear, but it is not incorporated into the mature tail. Analysis of the gene neighborhoods revealed that TAPI is most often flanked by the genes encoding the TAPK protein, with JAB and NlpC/P60 peptidase domains, and the TAPJ protein, which is required for host specificity (Table 1 row 5 and Figure 4). The JAB domains found in these gene associations are also a part of the monophyletic clade, including those from the above-described class of gene neighborhoods. Variants of this organization lacking either of the two flanking genes are seen in a few phages/prophages, and in a small group of phages TAPI is flanked by a version of TAPK containing only an NlpC/P60 peptidase domain (Figure 4). It is possible that the latter versions are actually degenerate variants of the former versions and are typical of integrated prophages.

#### Predicted gene clusters coding E1-like proteins, E2 (UBC)-like proteins, JAB peptidase, and novel Ub-like proteins

**Figure 6:**
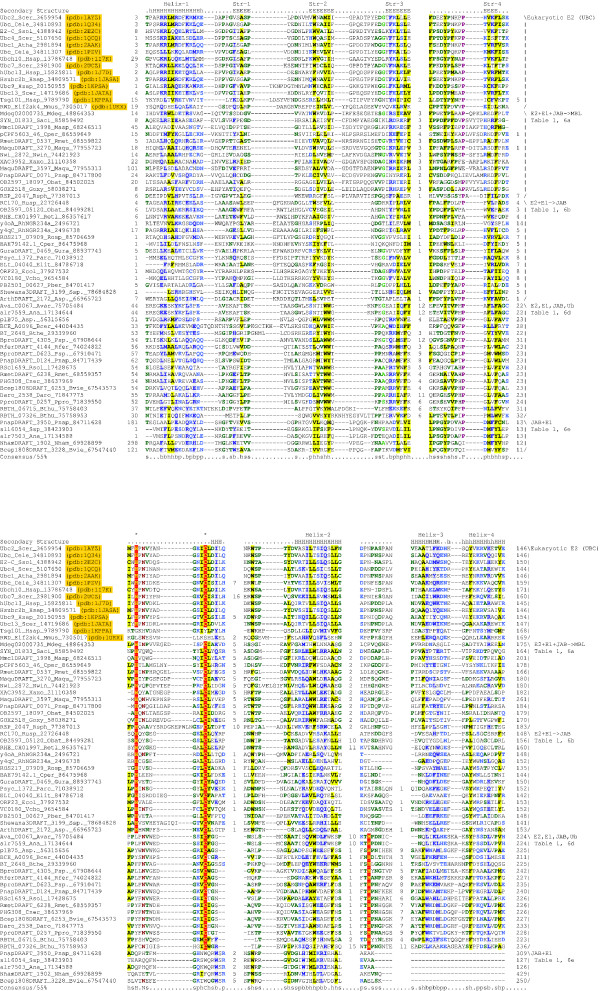
Multiple alignment of E2 (UBC)-like proteins with a special emphasis on bacterial versions. PDB identifiers of primary sequences derived from crystal structures are shaded in gold. Coloring is indicative of 55% consensus. The secondary structure, shown on the second line of the alignment, is derived from a general consensus of the secondary structure features from the different crystal structures shown in the alignment. Other features of the alignment are the same as in Figure 2, including coloring scheme, consensus abbreviations and secondary structure representations. Additionally, conserved polar residues (p; CDEHKNQRST) are colored blue. The strongly conserved proline and asparagine residues are colored purple brown respectively. The strongly conserved cysteine and histidine residues described in the text are shaded red and are also marked with an asterisk above their positions in the alignment. The major families of bacterial E2s are shown to the right. Also shown are the row numbers in Table 1, where a particular family is described. See the legend to Figure 2 for species abbreviations.

A number of sets of predicted operons, each with a distinctive sporadic distribution across several phylogenetically distant bacteria and encoding proteins with JAB domain and E1-like enzymes, were recovered in our search for conserved gene neighborhoods. E1-like enzymes in these gene neighborhoods never contained a carboxyl-terminal rhodanese domain. However, they were typically fused, either at the amino-terminus or the carboxyl-terminus, to the JAB domain. In the instances in which they were not fused to the JAB domain, there was always a JAB domain protein encoded by the immediately adjacent gene in the predicted operon (Table 1 rows 6a-6e and Figure 4). One group of proteins, typified by an E1-like protein fused to a JAB domain at the carboxyl-terminus, also contained an additional conserved amino-terminal domain, with a conserved histidine and cysteine (for example, Mdeg02000735 from *Microbulbifer degradans*, gi: 48864353; Table 1 row 6a and Figure 3). Iterative PSI-BLAST searches with the alignment of this domain as a seed recovered eukaryotic E2 (ubiquitin conjugating enzymes [UBC]) enzymes as hits with significant e values (e = 10^-3^, iteration 3). The predicted secondary structure of these domains was congruent with that of eukaryotic E2 domains, with a four-strand β-meander and two flanking helices on either side [61]. Furthermore, the conserved histidine and cysteine of the bacterial proteins also precisely matched the cognate active site residues of the eukaryotic E2 enzymes, suggesting that the amino-terminal domains of the bacterial domain are homologs of the E2 enzymes and likely to possess similar activity (Figure 6).

In addition, each set of these predicted operons contained a distinct group of genes that almost exclusively co-occurred with a particular operon type. Based on the different groups of co-occurring genes, we were able identify at least five major operon types (Table 1 rows 6a-6e and Figure 4). These groups of co-occurring genes encoded several conserved uncharacterized proteins, whose evolutionary relationships we systematically investigated using sequence profile searches, secondary structure prediction, and matches to libraries of profiles and HMMs for various previously characterized domains.

The first of these operon types exhibited a very simple organization, usually with two genes. One of them encoded the triple module protein, with amino-terminal E2-like and E1-like domains followed by a carboxyl-terminal JAB domain (Figure 3). The second gene in the operon encoded a specialized version of the metallo-β-lactamase domain (Table 1 row 6a and Figure 4). Another operon group typified by a conserved gene neighborhood from the *Escherichia coli *integrative and conjugative element (ICE) [62] and related mobile elements was found to contain a nucleotidyl transferase of the polymerase β-fold [63], in addition to the genes encoding the E1-like and JAB domain proteins (Table 1 row 6b and Figure 4). Like the E1-like proteins from the first group of conserved gene clusters the E1-like proteins of this group also show a fusion to an E2-related domain with a conserved active site cysteine (Figure 6). Similarly, a conserved operon group prototyped by a gene neighborhood from the megaplasmid NGR234 of *Rhizobium *sp. contains genes encoding two conserved uncharacterized proteins, one of which is predicted to contain a metal-binding domain based on the conserved pattern of two cysteines, a histidine, and an acidic residue (Table 1 row 6c and Figure 4). We observed that the E1-like proteins encoded by both of these operon types contained an additional amino-terminal domain with a conserved cysteine. Sequence searches with this amino-terminal region recovered the UBC-like E2 domains from a variety of eukaryotes. The best hit to these domains was from a profile of the E2-like proteins and included a match to the conserved cysteine (*P *< 10^-5 ^match for this cysteine containing motif in a Gibbs sampling search, with the MACAW program, including a wide range of known E2 domains). Secondary structure prediction for this conserved domain also showed complete congruence with the known structure of the E2 fold, suggesting that these amino-terminal domains fused to the E1-like enzymes are also homologs of the eukaryotic E2 ubiquitin conjugating enzymes (Figure 6).

A fourth operon type found in several diverse bacteria (Table 1 row 6d) typically contained three additional genes in the conserved gene neighborhood, in addition to the genes of the JAB domain and E1-like proteins (Figure 4). Furthermore, the JAB domain has an amino-terminal α + β domain that has a strictly conserved arginine and tryptophan residue (JAB-N; Figure 3). The first of these encodes a small protein with a highly conserved glycine at the carboxyl-terminus. Secondary structure prediction revealed that this small protein has a progression of structural elements identical to that seen in the β-grasp fold (Figure 2). The conservation pattern in this protein also strongly resembles that seen in the known β-grasp domains, and sequence-structure threading using the PHYRE program also recovered β-grasp proteins (for example, ThiS and PDB: 1tyg) as the best hits, suggesting that these are small standalone Ub-like proteins. The second protein encoded by this operon type was found to encode a largely α-helical protein with absolutely conserved charged and polar residues, suggesting that it might be an uncharacterized enzyme. The third conserved protein from these gene neighborhoods contained a conserved cysteine and gave significant hits to the profiles of the E2 Ub-conjugating enzymes, with the alignments spanning the conserved cysteine (Figure 6). This relationship was also supported by their predicted secondary structure and general conservation pattern. Although these proteins did not have the conserved histidine at the position often encountered in most E2 enzymes, they had an absolute conserved histidine further downstream (Figure 6). Mapping of the sequences of representatives of this family of proteins on the structures of E2 enzymes showed that this downstream histidine from the helix would be positioned very close to the active site histidine of the classical E2 enzymes (Figure 6). This would mean that these proteins are likely to effectively contain an active site similar to the classical E2 enzymes.

The fifth operon type is found sporadically in most proteobacterial lineages, cyanobacteria, and certain actinobacteria (Table 1 row 6e). Usually these gene neighborhoods contain two or three genes in addition to the central gene for an E1-like enzyme, which in most cases contains a JAB domain fused to the amino-terminus of the E1-like module. However, in a subset of bacteria the E1-like protein contains a fusion to an uncharacterized amino-terminal domain in place of the JAB domain (Figure 2). The conservation pattern of this domain is unrelated to that of the JAB domain, but it contains several conserved charged residues, making it tempting to speculate that it might perform a function analogous to the JAB domains. The other gene found in all gene neighborhoods of this type encodes a protein containing one to three repeats of an approximately 70-75 amino acid domain. The conservation pattern is similar to that seen in Ubls, and the predicted secondary structure of this domain exhibits a progression completely congruent to other β-grasp fold domains (Figure 2). Consistent with this, sequence-structure threading with the PHYRE program recovered the structures of the ThiS/MoaD proteins as the top hits (for example, PDB: 1tyg). These observations strongly suggest that this group of proteins is comprised of one or more Ub-like domains Table 1.

Furthermore, we noted that these predicted β-grasp domain proteins might also be fused with either of two unrelated carboxyl-terminal domains (Table 1). The first of these domains is a small domain of about 75 residues exhibiting a conservation pattern and secondary structure progression similar to the Ubls (Figure 2). These domains also recovered ThiS/MoaD as their best hits in sequence-structure threading with the PHYRE program, implying that it might form the third Ub-like domain in a subset of these proteins. The second carboxyl-terminal domain found in a mutually exclusive subset of these proteins also occasionally occurs as a standalone protein encoded by a separate gene sandwiched between the genes for the multi-β-grasp domain protein and the JAB + E1 domain proteins (Figure 3). Profile searches with an alignment of this domain recovered hits to the E2 enzymes and the eukaryotic RWD domain [61,64], which contains a catalytically inactive version of the E2 fold as the best hits (e about 0.01-0.005). This relationship was also supported by the congruence of the predicted secondary structure of these domains with that of the E2 and RWD domains [61]. Like the eukaryotic RWD domains, these bacterial domains also lacked the conserved cysteine residue, implying that they are likely to be catalytically inactive representatives of the E2-like fold (Figure 6). The above operon type was also seen to encode another conserved protein with a C-x(3)-C-x(35-38)-H-x(2)-C signature (Figure 4). The predicted secondary structure of this potential metal-binding signature is consistent with proteins containing a Zn finger domain, perhaps of the treble-clef fold.

### The RnfH associated conserved gene neighborhoods and other miscellaneous conserved gene neighborhoods

The RnfH protein is highly conserved across the β/γ proteobacteria (Table 1 row 8), and in each of these instances it occurs in a strongly conserved gene neighborhood also containing genes for a START domain protein, the transfer mRNA (tmRNA) binding protein SmpB, and a small membrane protein of unknown function SmpA. In this gene neighborhood we observed that the predicted promoter (or transcriptional regulatory regions) for the SmpB, the START domain protein, and RnfH appear to be shared in a small intergenic segment, with the former gene being transcribed in the opposite direction to the latter two (Figure 4). This neighborhood is of particular interest, given that the SmpB-tmRNA complex is used in bacteria to tag proteins from mRNAs lacking stop codons with small peptide. This tag targets proteins for degradation analogous to the eukaryotic Ub system [65]. A second type of conserved gene neighborhood containing an RnfH gene is found sporadically in a few proteobacteria, where it is linked to group of Rnf genes whose products form a membrane associated complex involved in transporting electrons for various reductive reactions such as nitrogen fixation [66].

In addition to this, there other gene clusters encoding Ub-related β-grasp domain proteins, such as the Tmo and YukD associated conserved gene neighborhoods. The Tmo operon encodes the toluene monooxygenase complex in several bacteria (Figure 4, Table 1 row 10). TmoB, the Ub-related protein of this complex, has been shown to be a subunit of the toluene/o-xylene mono-oxygenase hydroxylase, which binds a distinct conserved exposed ridge on the catalytic subunit [39]. However, it does not affect the activity of the enzyme *in vitro *and its exact role in the complex remains unknown. The predicted operons coding the Ub-like YukD proteins are found in several low GC Gram-positive bacteria, and we discovered additional homologs of them in actinobacteria (Figure 4, Table 1 row 11). In both of these bacterial taxa, the YukD protein is found in the neighborhood of the ESAT-6 export system (which at its core consists of a α-helical polypeptide), the virulence protein ESAT-6, and an FtsK-like ATPase that pumps these polypeptides outside the cell [67-69]. The actinobacterial YukD is always fused to a transmembrane domain consisting of 12 transmembrane helices. Additionally, the actinobacterial gene clusters contain a subtilisin-like protease (mycosin), members of the α-helical PE family, and the membrane-associated PPE family of proteins. The predicted operons of the low GC Gram-positive bacteria instead contain an S/T kinase and a membrane protein prototyped by the bacillus YueB protein (Figure 4). Experimental investigations showed that the YukD protein is not covalently conjugated with other proteins [40]. Our analysis of the gene neighborhood suggests that they may be involved as an assembly factor or structural component of the ESAT-6 polypeptide export system that might export a range of virulence factors in mycobacteria and potential signaling molecules in low GC Gram-positive bacteria.

### Functional implications of the prokaryotic systems with components related to eukaryotic to ubiquitin-signaling network

**Figure 7:**
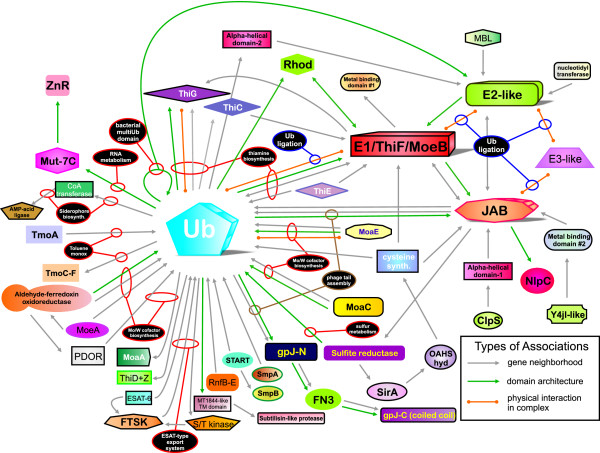
Network diagram of ThiS/MoaD-like β-grasp domains. The interaction network depicted here represents the known functional associations (arrows colored orange), the associations suggested by domain architectures (arrows colored green), and the associations suggested by gene neighborhood (arrows colored gray) between pairs of domains, as described in the text. The directionality of the network interactions, as indicated by an arrowhead, represents the order of a domain pair from the amino- to the carboxyl-terminus of the domain architecture or from the 5' to 3' end of a gene neighborhood. Lines with arrowheads at both ends represent domain pairs found both amino-terminal and carboxyl-terminal to each other in domain architectures or 5' to 3' in operonic contexts. The primary 'hubs' of the network are highlighted prominently. Domains are not exactly to scale. Selected interactions are encircled by small ellipses connected to the labels describing the functional role of the interaction. The labels are portrayed as large black ellipses with white lettering. MBL, metallo-β-lactamase domain; OAHS hyd, O-acetylhomoserine sulfhydrylase; PDOR, pyridine disulfide oxidoreductase; Rhod, Rhodanese-like domain; Toluene mono, toluene mono-oxygenase; ZnR, zinc-ribbon containing domain.

Much of the above-described diversity of prokaryotic functional systems involving Ub-signaling related proteins remains experimentally unexplored. However, the syntactical features of the domain architectures and conserved gene neighborhoods provide some hints regarding the general functional properties of these systems (Figures 4 and 7). One of the most striking features is the dichotomy in distribution, operon organization, and domain architectures of the versions involved in thiamine and MoCo/WCo biosynthesis and majority of other predicted operons (Table 1 and Figure 4). The former set of operons is highly conserved and is present across most bacterial and several archaeal lineages, which is suggestive of a pattern of vertical inheritance from LUCA or early in bacterial evolution. The other types of above-described predicted operons are instead sporadic in their distribution and found patchily across phylogenetically unrelated bacteria (Table 1). The former types do not contain a single instance of a gene encoding a JAB domain protein or a fusion to a JAB domain. In contrast to the thiamine and MoCo/Wco operons, the majority of other gene neighborhoods code a JAB domain protein along with an E1-like enzyme and/or Ub-like protein (Figure 4 and Table 1). A subset of these, namely those involved in the biosynthesis of siderophore-like compounds and those associated with sulfur assimilation and cysteine synthase, are linked with genes encoding metabolic enzymes. This suggests a role for them in the biochemistry of sulfur transfer, albeit in pathways that are likely to be distinct from the thiamine and MoCo/WCo (Figure 1). The other gene neighborhoods exhibit no major links to metabolic enzymes, suggesting that they might specify standalone regulatory pathways.

One of the most interesting features of these predicted functional systems is the presence of the JAB domain (Figure 5), which is universally conserved in eukaryotes and is the primary deubiqutinating peptidase/isopeptidase associated with the proteasome [21,22] (Figure 6). The association of the JAB peptidase with just an Ub-like protein with a carboxyl-terminal glycine in the phage tail assembly gene clusters strongly implies that the two domains form a functional unit even in the prokaryotes. It is quite probable that the phage TAPI is processed by the peptidase domains of TAPK, with the JAB probably releasing the Ub-like domain by cleaving at the point of the carboxyl-terminal-most glycine of the Ub domain. A similar function may be envisaged for the JAB domain in the organisms where ThiS or MoaD is fused to some other proteins; it might cleave off the Ubl-like moiety and generate a free carboxyl-terminus for sulfur transfer. However, the strong association of the JAB with sporadically distributed operon types related to the *Pseudomonas *siderophore biosynthesis pathways is more mysterious. Based on the complete absence of JAB proteins in the thiamine and MoCo/WCo pathways, we predict that in the pathways in which the E1-like enzyme is found in association with the JAB domain it functions via a mechanism distinct from that used by classical ThiF or MoeB. This mechanism is likely to be closer to the Ub transfer reaction of *bona fide *eukaryotic E1s, wherein the ThiS/MoaD or any other associated Ub-like protein is directly linked to a cysteine in the E1-like enzyme by a thioester linkage. In this situation, it is likely that the E1-like enzyme also transfers the covalently linked Ub-like protein to amino groups of lysines in particular target proteins. These linkages (equivalent to the isopeptide linkages of eukaryotic Ub-modified proteins) could then be cleaved by the associated JAB domain proteins (Figure 1).

The potential regulatory pathways defined by conserved gene neighborhoods that combine JAB and E1-like domain proteins often encode their own Ub domain proteins and homologs of the eukaryotic Ub conjugating E2 enzymes. Given the presence of E2 homologs, it is quite likely that these are indeed dedicated protein-modifying systems that add the associated Ub-like proteins or the available ThiS/MoaD to target proteins. In these cases we predict that the JAB domain is likely to be important for both processing the Ub-like proteins and removing them from the target proteins, thus constituting a genuine bacterial version of the eukaryotic Ub-signaling system. The operon type prototyped by the *E. coli *ICE element also encodes a nucleotidyl transferase (Figure 4 and Table 1 row 6b), which might provide an additional protein modification like its homolog the uridylyl transferase, which modifies glutamine synthase [63,70]. It is particularly interesting to note that some of these systems contain proteins with two to three tandem repeats of the Ub-like domain (reminiscent of the eukaryotic poly-ubiquitin) or RWD domain-like inactive versions of the E2-like fold, which probably bind the Ub moieties (Figures 1 and 6, and Table 1 row 6e). Some of the other uncharacterized proteins encoded specifically by these operon sets, such as the Zn finger protein (for example, sll6052 from *Synechocystis*), might be involved in recognizing specific target proteins for modification by these systems. The high mobility of these conserved gene clusters in bacteria is illustrated by their differential presence or absence even within closely related strains of same organism, and indeed some of them are borne by conjugative mobile elements (Table 1). This pattern of mobility is reminiscent of some other conserved operon systems such as the restriction-modification operons, the toxin-antitoxin systems, and the CRISPR system [68,71-74].

The predicted biochemical functions of these systems and the mobile gene clusters encoding β-grasp or JAB domain proteins are entirely unrelated. However, it is quite possible that in a general sense, like the two former systems, these gene clusters also maintain themselves by providing the cell with oppositely directed activities. Accordingly, we speculate that the JAB domain and the E1 + E2 complex provides a system that uses an endogenous ThiS/MoaD protein or the distinct Ub-like protein encoded by the mobile operon to alternately modify or de-modify cellular target proteins. This system might provide a means of regulating target protein stability and maintains itself by either acting as an addiction system like the toxin-antitoxin systems or as a means of protection against invasive replicons as the restriction-modification systems.

Other tantalizing, but uncertain, links between components of the bacterial Ub-like systems and protein stability are suggested by some of the conserved gene neighborhoods. The operon that encodes a JAB domain protein, an Ub-like protein related to ThiS/MoaD and ClpS, is one such (Figure 4 and Table 1 row 4d). The ClpS domain recognizes the amino-terminal domain of proteins targeted for destruction and links them to the protein-degrading ClpAP machine in bacteria and the RING finger E3 ligase of the eukaryotic N-recognins [75,76]. It is possible that this system may be involved in modification of proteins by an Ub-like modification before linkage by ClpS for degradation. A more enigmatic case is offered by the linkage between RnfH and SmpB; here apparently no Ub-like transfer system is involved. However, the tight neighborhood association with SmpB suggests that RnfH could in principle, under as yet unstudied conditions, interact with the tmRNA and influence protein stability.

### Evolutionary implications of prokaryotic cognates of the ubiquitin-signaling system

The identification of numerous prokaryotic systems containing proteins related to ubiquitin, E1, E2, and the JAB domain, beyond the previously known versions found in the thiamine and MoCo/WCo biosynthesis operons, throw considerable light on the emergence of the eukaryotic Ub-signaling system (Figure 7). Among the oldest versions of the Ub-fold are the TGS domains that are traced back to LUCA and bind RNA [37,77]. This suggests that the Ub-like versions of the β-grasp fold probably emerged before the LUCA as an RNA-binding domain. This is also supported by the observation that versions related to ThiS/MoaD, like the one fused to the Mut7-C RNAse domain (Figure 3), are also likely to participate in a RNA-binding function (Figure 7). Such a function might also hold for the RnfH protein, which is most closely related to the TGS domains (Figure 2). However, it is also clear that the MoaD and ThiS versions were also present in LUCA, implying that the divergence between sulfur carrier and RNA-binding versions occurred before the LUCA. The analysis of the phyletic patterns of the predicted operons suggests that the sulfur carrier version was a part of molybdenum metabolism in LUCA itself, whereas its recruitment for thiamine biosynthesis happened at the base of the bacterial tree. Likewise, at least a single representative of the E1-like enzymes had differentiated from the remaining Rossmann-type folds, through the acquisition of a distinct carboxyl-terminal module, by the time of the LUCA. Even in these two ancient pathways there appears to have been a progressive increase in the complexity of the reaction catalyzed by the E1-like enzyme on the Ub-like protein. Originally, it appears to have been merely an adenylation reaction, as has been suggested for the MoeB-MoaD pair [30]. However, the ThiS-ThiF pair involved an additional formation of a covalent persulfide linkage between the E1-like enzyme and the Ub-like protein (Figure 1).

The operon and domain architecture evidence suggests that reaction mechanisms similar to the eukaryotic E1 enzymes emerged next in specialized versions of the E1-like/Ub-like protein pairs found in the prokaryotes. These systems also added a JAB domain protein, probably in a role similar to that of their eukaryotic counterparts. The sequence and organizational diversity of the E1-like, E2-like, and Ub-like proteins from these remarkable bacterial systems is much higher than that seen in their eukaryotic cognates. This suggests that these systems probably first diversified in bacteria, and were acquired by the eukaryotes during their emergence via the symbiotic process involving the α-proteobacterial precursor of the mitochondrion. This is consistent with the frequent presence of the more complex Ub-signaling related systems in α-proteobacteria (Table 1). On the face of it, the E3 enzymes such as the RING domain and the HECT domain appear to be eukaryotic innovations. However, it cannot be ruled out that the additional uncharacterized proteins, such as the above-described Zn finger protein encoded in the bacterial gene neighborhoods (Figure 4 and Table 1), act as E3-like adaptors. However, it is clear that the core of the Ub transfer system, as well as the main peptidase required for its removal, namely the JAB domain, were already linked as a functional complex in the bacteria, before the emergence of the eukaryotes. The bacteriophage tail assembly system contains an NlpC/P60 peptidase, typically fused to the JAB domain (Figure 3), which might also be involved in processing the Ub-related protein. Given that the NlpC/P60 peptidase contains a papain-like fold also found in most of the eukaryotic DUBs, it is possible that the functional association between Ub-like domains and the papain-like peptidase emerged in the prokaryotic world. Links between these prokaryotic systems and protein degradation via ATP-dependent proteolytic machines are less clear, although there are some hints that the prokaryotic Ub-like domains might even play a role in such a process.

## Conclusion

By performing a systematic search for Ub-like domains in bacteria we identified several novel domains with diverse domain architectures. We present evidence that there are several predicted bacterial operons, beyond those specifying the previously well characterized thiamine and MoCo/WCo biosynthesis systems that encode Ub-related, JAB domain, and E1-like and E2-like proteins. These gene neighborhoods exhibit several distinct organizational themes, each of which is likely to specify a distinct functional system. Some of these systems are likely to possess the capacity to transfer Ub-like protein moieities onto target proteins via a relay of E1-like and E2-like proteins. This is the first report of a genuine prokaryotic ubiquitin-like signaling system, and we suggest that these systems were the precursors to the eukaryotic Ub-signaling system. We hope this report may stimulate experimental analysis of these bacterial systems and thereby throw light on the emergence of a signaling system that was hitherto considered the unique property of the eukaryotes.

## Materials and methods

The nonredundant (NR) database of protein sequences (National Center for Biotechnology Information [NCBI], NIH, Bethesda, MA, USA) was searched using the BLASTP program [78]. A complete list of these genomes and the predicted proteomes of prokaryotes used in this analysis in fasta format can be downloaded from the Complete Microbial Genomes database at the NCBI [79]. Additional sequences, from microbial genomes that have been sequenced but not completely assembled and submitted to the GenBank database, were also used in this analysis. A list of these prokaryotic genomes, from which sequences have been deposited in GenBank, can be accessed from the Draft Assembly Sequences database at the NCBI website [80]. Gene neighborhoods were determined using a custom script that uses completely sequenced genomes or whole genome shot gun sequences to derive a table of gene neighbors centered on a query gene. Then the BLASTCLUST program was used to cluster the products in the neighborhood and establish conserved co-occurring genes. These conserved gene neighborhood are then sorted as per a ranking scheme based on occurrence in at least one other phylogenetically distinct lineage ('phylum' in the NCBI Taxonomy database), complete conservation in a particular lineage ('phylum'), and physical closeness (<70 nucleotides) on the chromosome indicating sharing of regulatory -10 and -35 elements. Putative promoter regions were predicted if required by scanning for the consensus of the -10 and -35 elements in the predicted upstream regions.

Profile searches were conducted using the PSI-BLAST program with either a single sequence or an alignment used as the query, with a default profile inclusion expectation (e) value threshold of 0.01 (unless specified otherwise), and was iterated until convergence. For all searches involving membrane-spanning domains we used a statistical correction for compositional bias to reduce false positives due to the general hydrophobicity of these proteins [81]. The library of profiles for various signaling domains was prepared by extracting all alignments from the PFAM database [82] and updating them by adding new members from the NR database. These updated alignments were then used to make HMMs with the HMMER package [83] or PSSMs with PSI-BLAST.

Multiple alignments were constructed using the T_Coffee, MUSCLE, and PCMA programs followed by manual adjustments based on PSI-BLAST results [84-86]. The GIBSS sampling method, as implemented in the MACAW program, was used for the identification and statistical evaluation of conserved motifs in multiple protein sequences [87,88]. All large-scale sequence analysis procedures were carried out using the TASS package (Anantharaman V, Balaji S, Aravind L; unpublished data). Structural manipulations were carried out using the Swiss-PDB viewer program [89]. Searches of the PDB database with query structures were conducted using the DALI program [90,91]. Protein secondary structure was predicted using a multiple alignment as the input for the JPRED program, with information extracted from a PSSM, HMM, and the seed alignment itself [92]. Similarity-based clustering of proteins was carried out using the BLASTCLUST program [93]. Sequence-structure threading was carried out using the PHYRE and 3DPSSM programs [94]. Phylogenetic analysis was carried out using the maximum-likelihood, neighbor-joining, and least squares methods [95-97]. Briefly, this process involved the construction of a least squares tree using the FITCH program or a neighbor joining tree using the NEIGHBOR program (both from the Phylip package) [95], followed by local rearrangement using the Protml program of the Molphy package [96] to arrive at the maximum likelihood tree. The statistical significance of various nodes of this maximum likelihood tree was assessed using the relative estimate of logarithmic likelihood bootstrap (Protml RELL-BP), with 10,000 replicates. Text versions of all alignments reported in this study can be obtained in the Additional data file 1.

## Additional data files

The following additional data are included with the online version of this article: A text file containing a complete list of conserved gene neighborhoods, domain architectures, and alignments discussed in this article (Additional data file 1); a text file containing the complete list of all gi numbers for proteins encoded by conserved gene neighborhoods and their genomic position in various genomes (Additional data file 2); and a text file containing a list of major starting points for PSI-BLAST and HMMer searches and gi numbers detected in the searches conducted with them, along with e values (Additional data file 3).

The files are also available for download from the authors' FTP site [98].

## Supplementary Material

Additional data file 1 complete list of conserved gene neighborhoods, domain architectures, and alignments discussed in this article. Click here for file

Additional data file 2 Complete list of all gi numbers for proteins encoded by conserved gene neighborhoods and their genomic position in various genomes. Click here for file

Additional data file 3 A list of major starting points for PSI-BLAST and HMMer searches and gi numbers detected in the searches conducted with them, along with e values. Click here for file

## Figures and Tables

**Table 1:** Phyletic distribution and components of prominent gene neighborhoods of prokaryotic beta-grasp proteins

Row	Gene neighborhood type	Phyletic pattern	Protein coded by conserved genes neighborhoods/comments
1	Thiamine biosynthesis	All known bacterial lineages	ThiS, ThiG, ThiF, ThiC, ThiD, ThiE, ThiH and ThiO Comment: In many proteobacteria and the actinobacterium *Rubrobacter xylanophilus*, the ThiS is fused to a ThiG. In a subset of δ/ε proteobacteria and low GC Gram-positive bacteria, the ThiS is fused to a ThiF and these operons also encode a second solo ThiS-like protein
2	Molybdenum cofactor biosynthesis	All known bacterial and most archaeal lineages	MoaE, MoaC and MoaA Comment: In some rare instances, MoeB is present in the same operon as MoaD
3	Tungsten cofactor biosynthesis	Euryarchaea: Mace, Mmaz, Paby, Pfur, Pfur, Phor, and Tkod α, β, γ, δ/ε proteobacteria: Aehr, Asp., Dace, Ddes, Dpsy, Dvul, Gmet, Gsul, Mmag, Pcar, Pnap, Ppro, Rfer, Rgel, Sfum, and Wsuc Low GC Gram positive: Chyd, Moth, Swol, Teth, and The Actinobacteria: Sthe Other bacteria: Tth	MoaD, aldehyde-ferredoxin oxidoreductase, MoeB, MoaE, MoeA, pyridine disulfide oxidoreductase, and 4Fe-S ferredoxin Comment: In *Azoarcus*, the MoaD is fused carboxyl-terminal to the aldehyde ferredoxin oxidoreductase (Figure 3)
4a	Siderophore biosynthesis	β and γ proteobacteria: Neur, Nmul, Rsol, Pflu, Hche, Pstu, and Pput	ThiS/MoaD-like Ub (PdtH), E1-like enzyme fused to a Rhodanese domain (PdtF), JAB (PdtG), CaiB-like CoA transferase (PdtI), and AMP-acid ligase (PdtJ) Comment: Experimentally characterized siderophores encoded by this pathway include PDTC and quinolobactin
4b	Uncharacterized operon encoding a ThiS/MoaD, a JAB peptidase, and E1-like enzyme	γ, δ/ε proteobacteria: Adeh^a^, Aehr^a^, and Noce Cyanobacteria: Ana, Avar, Gvio^a^, Npun, Pmar Syn, and Telo	E1 fused to a Rhodanese domain and JAB Comment: ^a^These species also possess a ThiS/MoaD-like Ub
4c	Uncharacterized operon with a ThiS/MoaD, E1-like enzyme, a JAB, and a cysteine synthase	α, γ proteobacteria: Paer and Rpal Acidobacteria: Susi Actinobacteria: Rxyl Bacteroidetes/Chlorobi: Srub Chloroflexus: Caur	E1 is fused to a Rhodanese domain
4d	Uncharacterized operon with a ThiS/MoaD, JAB, cysteine synthase, and ClpS	Actinobacteria: Fsp., Mtub, Nfar, Nsp., Save, Scoe, and Tfus	Comment: Additionally the operon encodes an uncharacterized conserved protein with an α-helical domain (Figure 3)
4e	Operons with genes for sulfur metabolism proteins	δ/ε proteobacteria: Gmet and Wsuc Low GC Gram positive: Amet, Bcer, Chyd, Csac, Cthe, and Dhaf Bacteroidetes/Chlorobi: Cpha Actinobacteria: Nsp. and Acel Crenarchaea: Pyae	ThiS/MoaD-like protein, JAB, E1-like protein, SirA, sulfite/sulfate ABC transporters, PAPS reductase, ATP sulfurylase, sulfite reductase, O-acetylhomoserine sulfhydrylase, and adenylylsulfate kinase Comment: The ThiS/MoaD domain in Nsp and Acel are fused to a sulfite reductase
5	Phage tail assembly associated Ub	Lambdoid and T1 phages	Ub-like TAPI, TAPK protein with a JAB and NlpC domains, and TAPJ Comment: The TAPI proteins additionally have a carboxyl-terminal domain that is separated from the Ub domain by a glycine rich region. In some prophages, TAPI is fused to the TAPJ protein. In one particular prophage of Ecol (Figure 3) the TAPI is fused to the JAB. The NlpC domains of these versions almost always lack the JAB domain. These latter operons also encode a β-strand rich domain containing protein (labeled 'Z' in Figure 4)
6a	Uncharacterized operon with a triple module protein containing an E2-like, E1-like, and JAB domains	α, β, γ, δ/ε proteobacteria: gKT 71, Goxy, Maqu, Msp, Nwin, Obat, Pnap, Rmet, Rsph, Saci, Sdeg, and Xaxo Low GC Gram positive: Cper	Triple module protein with E2 (UBC), E1-like domain and JAB, lined in a single polypeptide in that order. Comment: In most operons, these are almost always next to a metallo-β-lactamase
6b	Uncharacterized operon encoding a multidomain protein with E2 and E1 domains	α, β, γ, δ/ε proteobacteria: Ecol, Elit, Gura, Obat, Parc, Pber, Retl, RhNGR234a, Rosp., Rusp., Shsp., and Vcho Actinobacteria: Asp. Low GC Gram positive: Cper	Multidomain protein with E2 and E1 domains, JAB, and polβ superfamily nucleotidyl transferase Comment: Both the E2 + E1 protein and the JAB are closely related to the corresponding sequences of the operons in the previous row of the table. Most of these operons are in ICE-like mobile elements and plasmids
6c	Uncharacterized operon encoding a distinctive multidomain protein with E2 and E1 related domains	α proteobacteria: Mlot, Mmag, Retl, RhNGR234, and Rpal	Multidomain E2 + E1 protein, JAB, and predicted metal binding protein Comment: In Mmag and Rpal, the E1 domain is fused to a distinct domain instead of E2. The E2-like domain has a conserved cysteine in place of the conserved histidine of the classical E2s
6d	Uncharacterized operon coding a Ub-like protein, a JAB, an E1-like protein, and an E2-like protein	β, δ/ε proteobacteria: Asp., Bvie, Cnec, Daro, Pnap, Ppro, Posp., Rfer, Rmet, and Rsol Low GC Gram positive: Bcer and Bthu Cyanobacteria: Ana and Avar Bacteroides: Bthe	Ub-like protein, JAB, E1-like, E2-like, and novel α-helical protein Comment: The E2-like protein lacks the conserved histidine of the classical E2-fold. However, they have an absolutely conserved histidine carboxyl-terminal to the conserved cysteine. The rapidly diverging α-helical protein has several absolutely conserved charged residues, suggesting that it may function as an enzyme. The JAB domains of this family additionally have an amino-terminal α + β domain characterized by a conserved arginine and tryptophan residue
6e	Uncharacterized operons coding a protein with tandem repeats of a ubiquitin-like domain (polyUbl)	α, β, γ, δ/ε proteobacteria: Amac, Bvie^c^, Mlot^b^, Nham^c^, Pnap^c^, Rmet^b^, Rpal^b^, Shsp.^b^, and Vpar^b^ Actinobacteria: Fsp.^b^ Cyanobacteria: Ana and Syn	PolyUbl, inactive E2-/RWD like UBC fold domain, multidomain protein with a JAB fused to an E1 domain, and a metal-binding protein (labeled Y in Figure 3) Comment: The polyUbls contain between two and three Ub-like domains (Figure 3). ^b^Some versions of the E1 domain have a distinct domain in place of the JAB domain (domain X in Figure 3). ^c^In some species the polyUbl is fused to an inactive E2-like domain. Amac has a solo Ub-like domain
7	Ubl fused to Mut7-C	Wide range of β proteobacteria and Avin Actinobacteria: Mtub, Scoe, Save, Mavi, Nfar, and Tfus Acidobacteria: Susi Cyanobacteria: Npun Tmar	No conserved genome context
8	Uncharacterized operon encoding a RnfH family protein	A wide range of β and γ proteobacteria and Mmag	Ub-like RnfH, a START domain containing protein, SmpA, and SmpB
9	Mobile RnfH operon	α, β, γ proteobacteria: Asp., Daro, Pstu, Rcap, and Zmob	Ub-like RnfH, RnfB, RnfC, RnfD, RnfG, and RnfE Comment: These components are part of an electron transport chain involved in reductive reactions such as nitrogen fixation
10	Toluene-O-xylene mono-oxygenase hydroxylase	α, β, and γ proteobacteria: Bcep, Bsp., Daro, Paer, Pmen, Psp. Reut, Rmet, Rpic, and Xaut Actinobacteria: Rsp. and Fsp.	Ub-like TmoB, toluene-4-mono-oxygenase hydroxylase (TmoA), hydroxylase/mono-oxygenase regulatory protein (TmoD), toluene-4-mono-oxygenase hydroxylase (TmoE), Rieske 2Fe-S protein (TmoC), NADH-ferredoxin oxidoreductase (TmoF), 4-oxalocrotonate decarboxylase (4OCDC), and 4-oxalocrotonate tautomerase (4OCTT)
11	YukD-like ubiquitin	Low GC Gram positive: Bcer, Bcla, Bhal, Blic, Bsub, Bthu, Cace, Cthe, Linn, Lmon, Oihe, Saga, Saur, and Saur Actinobacteria: Cjei, Jsp., Mavi, Mbov, Mfla, Mlep, Msp., Mtub, Mvan, Nfar, Nsp., Save, and Scoe	Ub-like YukD, FtsK-like ATPase, S/T kinase, YueB-like membrane protein, subtilisin-like protease, ESAT-6 like virulence factor, PE domain, and PPE domain Comment: The Ub-like YukD in actinobacteria is fused to a multipass integral membrane domain with 12 transmembrane helices

Proteobacteria: Adeh, *Anaeromyxobacter dehalogenans*; Aehr, *Alkalilimnicola ehrlichei*; Amac, *Alteromonas macleodii*; Asp., *Azoarcus *sp.; Avin, *Azotobacter vinelandii*; Bsp., Bradyrhizobium sp.; Bcep, *Burkholderia cepacia*; Bvie, *Burkholderia vietnamiensis*; Cnec, *Cupriavidus necator*; Dace, *Desulfuromonas acetoxidans*; Daro, *Dechloromonas aromatica*; Ddes, *Desulfovibrio desulfuricans*; Dpsy, *Desulfotalea psychrophila*; Dvul, *Desulfovibrio vulgaris*; Ecol, *Escherichia coli*; Elit, *Erythrobacter litoralis*; gKT 71, gamma proteobacterium KT 71; Gmet, *Geobacter metallireducens*; Gsul, *Geobacter sulfurreducens*; Goxy, *Gluconobacter oxydans*; Gura, *Geobacter uraniumreducens*, Hche, *Hahella chejuensis*; Maqu, *Marinobacter aquaeolei*; Mlot, *Mesorhizobium loti*; Mmag, *Magnetospirillum magnetotacticum*; Msp, *Magnetococcus *sp. MC-1; Neur, *Nitrosomonas europaea*; Nham, *Nitrobacter hamburgensis*; Nmul, *Nitrosospira multiformis*; Noce, *Nitrosococcus oceani*; Nwin, *Nitrobacter winogradskyi*; Obat, *Oceanicola batsensis*; Pber, *Parvularcula bermudensis*; Pnap, *Polaromonas naphthalenivorans*; Paer, *Pseudomonas aeruginosa*; Parc, *Psychrobacter arcticus*; Pcar, *Pelobacter carbinolicus*; Pflu, *Pseudomonas fluorescens*; Pmen, *Pseudomonas mendocina*; Pnap, *Polaromonas naphthalenivorans*; Posp., *Polaromonas *sp; Ppro, *Pelobacter propionicus*; Pput, *Pseudomonas putida*; Psp., *Pseudomonas *sp.; Pstu, *Pseudomonas stutzeri*; Rcap, *Rhodobacter capsulatus*; Retl, *Rhizobium etli*; Reut, *Ralstonia eutropha*; Rfer, *Rhodoferax ferrireducens*; Rgel, *Rubrivivax gelatinosus*; RhNGR234a, *Rhizobium *sp. NGR234a plasmid; Rmet, *Ralstonia metallidurans*; Rpal, *Rhodopseudomonas palustris*; Rpic, *Ralstonia pickettii*; Rmet, *Ralstonia metallidurans*; Rsph, *Rhodobacter sphaeroides*; Rosp., *Roseovarius *sp.; Rsol, *Ralstonia solanacearum*; Rusp., *Ruegeria *sp.; Saci, *Syntrophus aciditrophicus*; Sdeg, *Saccharophagus degradans*; Sfum, *Syntrophobacter fumaroxidans*; Shsp., *Shewanella *sp. ANA-3; Xax, *Xanthomonas axonopodis*; Vcho, *Vibrio cholerae*; Vpar, *Vibrio parahaemolyticus*; Wsuc, *Wolinella succinogenes*; Xaut, *Xanthobacter autotrophicus*; Zmob, *Zymomonas mobilis*. Low GC gram positive bacteria: Amet, *Alkaliphilus metalliredigenes*; Bcer, *Bacillus cereus*; Bcla, *Bacillus clausii*; Bhal, *Bacillus halodurans*; Blic, *Bacillus licheniformis*; Bsub, *Bacillus subtilis*; Bthu, *Bacillus thuringiensis*; Cace, *Clostridium acetobutylicum*; Chyd, *Carboxydothermus hydrogenoformans*; Cper, *Clostridium perfringens*; Csac, *Caldicellulosiruptor saccharolyticus*; Cthe, *Clostridium thermocellum*; Dhaf, *Desulfitobacterium hafniense*; Linn, *Listeria innocua*; Lmon, *Listeria monocytogenes*; Moth, *Moorella thermoacetica*; Oihe, *Oceanobacillus iheyensi*; Saga, *Streptococcus agalactiae*; Saur, *Staphylococcus aureus*; Swol, *Syntrophomonas *wolfei; Teth, *Thermoanaerobacter ethanolicus*. Actinobacteria: Asp., *Arthrobacter *sp.; Cjei, *Corynebacterium jeikeium*; Fsp., *Frankia *sp.; Jsp., *Janibacter *sp.; Mavi, *Mycobacterium avium*; Mbov, *Mycobacterium bovis*; Mfla, *Mycobacterium flavescens*; Mlep, *Mycobacterium leprae*; Msp., *Mycobacterium *sp.; Mtub, *Mycobacterium tuberculosis*; Mvan, *Mycobacterium vanbaalenii*; Nfar, *Nocardia farcinica*; Nsp., *Nocardioides *sp.; Rsp., *Rhodococcus *sp.; Rxyl, *Rubrobacter xylanophilus*; Save, *Streptomyces avermitilis*; Scoe, *Streptomyces coelicolor*; Sthe, *Symbiobacterium thermophilum*; Tfus, *Thermobifida fusca*. Cyanobacteria: Ana, *Anabaena *sp. PCC 7120; Avar, *Anabaena variabilis*; Gvio, *Gloeobacter violaceus*;, Npun, *Nostoc punctiforme*; Pmar, *Prochlorococcus marinus*; Syn, *Synechococcus sp*.; Telo, *Synechococcus elongates*; Tery, *Trichodesmium erythraeum*. Other bacterial groups: Bthe, *Bacteroides thetaiotaomicron*; Caur, *Chloroflexus aurantiacus*; Cpha, *Chlorobium phaeobacteroide*; Srub, *Salinibacter ruber*; Susi, *Solibacter usitatus*; Tmar, *Thermotoga maritima*; Tth, *Thermus thermophilus*. Euryarchaea: Mace, *Methanosarcina acetivorans*; Mmaz, *Methanosarcina mazei*; Paby, *Pyrococcus abyssi*; Pfur, *Pyrococcus furiosus*; Phor, *Pyrococcus horikoshii*; Tkod, *Thermococcus kodakarensis*. Crenarchaea: Pyae, *Pyrobaculum aerophilum*.
