# Supplementary material for: The prokaryotic antecedents of the ubiquitin-signaling system and the early evolution of ubiquitin-like β-grasp domains
Source: Genome Biol. 2006 Jul 19;7(7):R60. doi: 10.1186/gb-2006-7-7-r60 (PMC1779556; doi:10.1186/gb-2006-7-7-r60)
Supplement: Additional data file 1 — complete list of conserved gene neighborhoods, domain architectures, and alignments discussed in this article. [file gb-2006-7-7-r60-S1.html]

```
            Supplementary material- File 1
The prokaryotic antecedents of the Ubiquitin signaling system
and the early evolution of ubiquitin-like �-grasp domains

Lakshminarayan M. Iyer, A. Maxwell Burroughs and L. Aravind


Presented below are the domain architectures and operon contexts of the different
systems reported in the study. The different groups are represented by the gi of one of the
components from the operons (marked with an asterisk). The operons are usually shown next to the organism name
where "->" signifies gene order from the 5'to 3' direction. Domain architectures are shown with
a '+' separating the domains. Also shown are the species names and the evolutionary group to which
a particular species belongs.
The general order of the major subgroups/operon types follows the order in Table 1.
We also provide alignments of various families described in the study.
--------------------------------------------------------------------------------------------------------------
1A. Classical Thiamine biosynthesis pathway
^^^^^^^^^^^^^^^^^^^^^^^^^^^^^^^^^^^^^^^^^^^

# The gis shown below are ThiE, ThiD and ThiG like proteins (marked with an asterisk); also shown are the length of the protein
GI             LENGTH     Operon                                                                                                              ORGANISM                                                         Classification                                Protein description (if any)
13879931       222    <-ThiE*||ThiO->ThiS->ThiG->                                                                                             Mycobacterium tuberculosis CDC1551;                              actinobacteria
66869375       237    <-ThiG<-ThiS<-ThiO||ThiE*->                                                                                             Arthrobacter sp. FB24                                            actinobacteria
62424304       220    <-ThiF<-ThiG<-ThiS<-ThiO||ThiE*->                                                                                       Brevibacterium linens BL2                                        actinobacteria
13092621       235    ThiC->ThiE-><-ThiG<-ThiS<-ThiO||ThiE*->                                                                                 Mycobacterium leprae                                             actinobacteria
71915182       218    <-ThiG<-ThiS<-ThiO||?->ThiE*->                                                                                          Thermobifida fusca YX;                                           actinobacteria
41409995       223    <-ThiE*||ThiO->ThiS->ThiG->                                                                                             Mycobacterium avium subsp. paratuberculosis K-10                 actinobacteria
68263528       218    <-ThiF<-ThiG<-ThiS<-ThiO<-?<-ThiE*<-ThiC                                                                                Corynebacterium jeikeium K411                                    actinobacteria
86741756       237    <-ThiE*<-ThiS<-?<-?<-PDOR<-ThiH<-ThiG                                                                                   Frankia sp. CcI3                                                 actinobacteria
68230362       229    <-ThiE*<-ThiS<-?<-?<-PDOR<-ThiH<-ThiG                                                                                   Frankia sp. EAN1pec                                              actinobacteria
54018822       232    <-Mopterin_binding_protein<-ThiG<-ThiS<-ThiO||ThiE*->                                                                   Nocardia farcinica IFM 10152                                     actinobacteria
23493774       216    ThiE*->ThiO->ThiS->ThiG->ThiF->                                                                                         Corynebacterium efficiens YS-314                                 actinobacteria
38198930       222    ThiC->ThiE*->ThiO->ThiS->ThiG->ThiF->ThiD->                                                                             Corynebacterium diphtheriae                                      actinobacteria
13092617       279    ThiC->ThiD*-><-ThiG<-ThiS<-ThiO||ThiE->                                                                                 Mycobacterium leprae                                             actinobacteria
46191094       289    <-ThiF<-ThiG*<-ThiS                                                                                                     Bifidobacterium longum DJO10A                                    actinobacteria
5689919        264    <-PDOR||ThiO->ThiS->ThiG*->                                                                                             Streptomyces coelicolor A3(2)                                    actinobacteria
85666191       304    <-ThiG*<-ThiF<-ThiS                                                                                                     Bifidobacterium adolescentis                                     actinobacteria
71367701       197    ThiE->ThiO->ThiS->ThiG->ThiE*->ThiD->ThiC->                                                                             Nocardioides sp. JS614                                           actinobacteria
71481458       259    moaA-><-?||?->OAHSH->ThiS->ThiG*->ThiH->ThiF->                                                                          Prosthecochloris vibrioformis DSM 265                            bacteroidetes/chlorobi
34541689       259    <-ThiH<-ThiG*<-?<-ThiC<-ThiS                                                                                            Porphyromonas gingivalis W83                                     bacteroidetes/chlorobi
68550329       259    <-ThiF<-ThiH<-ThiG*<-ThiS<-OAHSH<-Cysteine_synthase<-permease                                                           Pelodictyon phaeoclathratiforme BU-1                             bacteroidetes/chlorobi
67939245       259    ThiS->ThiG*->ThiH->                                                                                                     Chlorobium phaeobacteroides BS1                                  bacteroidetes/chlorobi
21646637       259    <-ThiF<-ThiH<-ThiG*<-ThiS<-Cysteine_synthase<-OAHSH                                                                     Chlorobium tepidum TLS                                           bacteroidetes/chlorobi
67935885       259    <-ThiF<-ThiH<-ThiG*<-ThiS<-OAHSH||?-><-moaA                                                                             Chlorobium phaeobacteroides DSM 266                              bacteroidetes/chlorobi
78167321       275    OAHSH->ThiS->ThiG*->ThiH->ThiF->                                                                                        Pelodictyon luteolum DSM 273                                     bacteroidetes/chlorobi
78171432       256    OAHSH->?->ThiS->ThiG*->ThiH->?->ThiF->                                                                                  Chlorobium chlorochromatii CaD3                                  bacteroidetes/chlorobi
67919395       259    moaA-><-?||?->OAHSH->OAHSH->ThiS->ThiG*->ThiH->ThiF->                                                                   Chlorobium limicola DSM 245                                      bacteroidetes/chlorobi
60493472       204    ThiS->ThiE*->ThiG->ThiC->?->ThiH->                                                                                      Bacteroides fragilis NCTC 9343                                   bacteroidetes/chlorobi
52216685       204    ThiS->ThiE*->ThiG->ThiC->?->ThiH->                                                                                      Bacteroides fragilis YCH46                                       bacteroidetes/chlorobi
83758120       210    ThiO->ThiS->ThiG->ThiE*->ThiE->                                                                                         Salinibacter ruber DSM 13855                                     bacteroidetes/chlorobi
48855690       203    ThiS->ThiC->ThiD->ThiE*->ThiG->ThiH->                                                                                   Cytophaga hutchinsonii                                           bacteroidetes/chlorobi
83755862       290    ThiO->ThiS->ThiG->ThiE->ThiE*->                                                                                         Salinibacter ruber DSM 13855                                     bacteroidetes/chlorobi
29337956       209    <-ThiF<-ThiH<-ThiC<-ThiG<-ThiE*<-ThiS                                                                                   Bacteroides thetaiotaomicron VPI-5482                            bacteroidetes/chlorobi
33238326       346    ThiE*->ThiS->                                                                                                           Prochlorococcus marinus subsp. marinus str. CCMP1375             cyanobacteria
87125481       348    ThiE*->ThiS->                                                                                                           Synechococcus sp. RS9917                                         cyanobacteria
33639113       349    ThiE*->ThiS->                                                                                                           Synechococcus sp. WH 8102                                        cyanobacteria
86605751       257    <-ThiG*<-ThiS<-ThiO                                                                                                     Synechococcus sp. JA-3-3Ab                                       cyanobacteria
17130690       379    ThiE*->ThiS->                                                                                                           Nostoc sp. PCC 7120;                                             cyanobacteria
35210964       366    <-ThiS<-ThiE*                                                                                                           Gloeobacter violaceus PCC 7421;                                  cyanobacteria
67922607       338    ThiE*->ThiS->                                                                                                           Crocosphaera watsonii WH 8501                                    cyanobacteria
72002529       350    ThiE*->ThiS->                                                                                                           Prochlorococcus marinus str. NATL2A;                             cyanobacteria
33634552       353    <-ThiS<-ThiE*                                                                                                           Prochlorococcus marinus str. MIT 9313                            cyanobacteria
71674938       360    <-ThiS<-ThiE*                                                                                                           Trichodesmium erythraeum IMS101;                                 cyanobacteria
33640198       351    ThiE*->ThiS->                                                                                                           Prochlorococcus marinus subsp. pastoris str. CCMP1986            cyanobacteria
78713251       365    ThiE*->ThiS->                                                                                                           Prochlorococcus marinus str. MIT 9312;                           cyanobacteria
84512362       343    <-ThiS<-ThiE*                                                                                                           Prochlorococcus marinus str. MIT 9211                            cyanobacteria
56685459       343    <-ThiS<-ThiE*                                                                                                           Synechococcus elongatus PCC 6301                                 cyanobacteria
78169363       346    ThiE*->ThiS->                                                                                                           Synechococcus sp. CC9902                                         cyanobacteria
78196899       352    <-ThiS<-ThiE*                                                                                                           Synechococcus sp. CC9605                                         cyanobacteria
66797755       221    ThiC->ThiE*->ThiS->ThiG->?->ThiD->                                                                                      Deinococcus geothermalis DSM 11300                               deinococci
55772056       206    ThiE*->ThiS->ThiG->?->ThiC->?->ThiD->                                                                                   Thermus thermophilus HB8                                         deinococci
6460491        280    <-permease<-?<-?<-ThiD<-ThiG<-ThiS<-ThiE*<-ThiC                                                                         Deinococcus radiodurans R1                                       deinococci
82744798       256    Mopterin_binding_protein->?->ThiS->ThiG*->ThiH->                                                                        Clostridium beijerincki NCIMB 8052                               firmicutes
72496362       218    <-ThiF<-ThiG<-ThiS<-ThiO<-ThiE*                                                                                         Staphylococcus saprophyticus subsp. saprophyticus ATCC 15305     firmicutes
83590499       255    ThiS->ThiG*->ThiH->                                                                                                     Moorella thermoacetica ATCC 39073                                firmicutes
68055200       198    <-ThiF<-ThiG<-ThiS<-ThiO<-ThiE*                                                                                         Exiguobacterium sp. 255-15                                       firmicutes
15025970       195    <-ThiE*<-ThiH<-ThiG<-ThiF<-ThiS                                                                                         Clostridium acetobutylicum ATCC 824;                             firmicutes
77996134       215    ThiS->ThiG->ThiH->ThiF->ThiE*->                                                                                         Carboxydothermus hydrogenoformans Z-2901                         firmicutes
82499658       219    ThiS->ThiG->ThiH->ThiF->ThiE*->ThiC->                                                                                   Caldicellulosiruptor saccharolyticus DSM 8903                    firmicutes
2633520        205    ThiE*->ThiO->ThiS->ThiG->ThiF->ThiD->                                                                                   Bacillus subtilis subsp. subtilis str. 168;                      firmicutes
52002880       203    ThiE*->ThiO->ThiS->ThiG->ThiF->ThiD->                                                                                   Bacillus licheniformis ATCC 14580                                firmicutes
10174048       211    ThiE*->ThiS->ThiG->ThiO->ThiD->                                                                                         Bacillus halodurans C-125                                        firmicutes
68446290       197    <-ThiF<-ThiG<-ThiS<-ThiO<-ThiE*                                                                                         Staphylococcus haemolyticus JCSC1435                             firmicutes
57865486       152    ThiE*->ThiO->ThiS->ThiG->ThiF->                                                                                         Staphylococcus epidermidis RP62A                                 firmicutes
23023751       212    <-ThiG<-ThiF<-ThiS<-ThiE*                                                                                               Leuconostoc mesenteroides subsp. mesenteroides ATCC 8293         firmicutes
56909741       209    ThiE*->ThiS->ThiG->ThiO->ThiD->                                                                                         Bacillus clausii KSM-K16                                         firmicutes
77683441       196    ThiS->ThiF->ThiG->ThiH->ThiC->ThiE*->                                                                                   Alkaliphilus metalliredigenes QYMF                               firmicutes
67875149       356    <-ThiC<-ThiE*<-ThiF<-ThiH<-ThiG<-ThiS                                                                                   Clostridium thermocellum ATCC 27405                              firmicutes
18145262       193    <-ThiE*<-ThiH<-ThiG<-ThiF<-ThiS                                                                                         Clostridium perfringens str. 13                                  firmicutes
47501161       206    Mopterin_binding_protein->?->?->ThiE*->ThiO->ThiS->ThiG->ThiF->ThiD->                                                   Bacillus anthracis str. 'Ames Ancestor'                          firmicutes
56378999       201    ThiE*->ThiO->ThiS->ThiG->ThiF->                                                                                         Geobacillus kaustophilus HTA426                                  firmicutes
19712999       206    <-ThiE<-ThiH<-ThiG<-ThiF<-ThiS<-ThiC<-ThiE*<-ThiD                                                                       Fusobacterium nucleatum subsp. nucleatum ATCC 25586;             fusobacteria
32397912       287    <-ThiG*||?-><-ThiS                                                                                                      Rhodopirellula baltica SH 1                                      planctomycetes
27354938       208    ThiO->ThiS->ThiG->ThiE*->ThiC->                                                                                         Bradyrhizobium japonicum USDA 110;                               proteobacteria>alphaproteobacteria
78495123       202    <-ThiC<-ThiE*<-ThiG<-ThiS<-ThiO                                                                                         Rhodopseudomonas palustris BisB18                                proteobacteria>alphaproteobacteria
69299787       198    ThiD->ThiO->ThiS->ThiG->ThiE*->ThiF->                                                                                   Silicibacter sp. TM1040                                          proteobacteria>alphaproteobacteria
56676713       198    ThiD->ThiO->ThiS->ThiG->ThiE*->ThiF->                                                                                   Silicibacter pomeroyi DSS-3                                      proteobacteria>alphaproteobacteria
83751112       201    <-ThiD<-ThiE*<-ThiG<-ThiS<-ThiO<-ThiC                                                                                   Bartonella bacilliformis KC583;                                  proteobacteria>alphaproteobacteria
49238087       252    <-ThiD*<-ThiE<-ThiG<-ThiS<-ThiO<-ThiC||?-><-Mopterin_binding_protein                                                    Bartonella henselae str. Houston-1                               proteobacteria>alphaproteobacteria
84501018       198    <-ThiF<-ThiE*<-ThiG<-ThiS<-ThiO<-ThiD                                                                                   Oceanicola batsensis HTCC2597                                    proteobacteria>alphaproteobacteria
85705980       198    <-ThiF<-ThiE*<-ThiG<-ThiS<-ThiO<-ThiD                                                                                   Roseovarius sp. 217                                              proteobacteria>alphaproteobacteria
83952604       203    ThiC->ThiO->ThiS->ThiG->ThiE*->ThiF->ThiD->                                                                             Roseovarius nubinhibens ISM                                      proteobacteria>alphaproteobacteria
86137738       196    ThiC->ThiO->ThiS->ThiG->ThiE*->ThiF->ThiD->                                                                             Roseobacter sp. MED193                                           proteobacteria>alphaproteobacteria
39650494       202    ThiO->ThiS->ThiG->ThiE*->ThiC->                                                                                         Rhodopseudomonas palustris CGA009                                proteobacteria>alphaproteobacteria
17983764       203    ThiD->ThiO->ThiS->ThiG->ThiE*->ThiC                                                                                     Brucella melitensis 16M;                                         proteobacteria>alphaproteobacteria
83954398       198    <-ThiF<-ThiE*<-ThiG<-ThiS<-ThiO<-ThiD                                                                                   Sulfitobacter sp. NAS-14.1                                       proteobacteria>alphaproteobacteria
71062546       257    ThiS->ThiG*->                                                                                                           Candidatus Pelagibacter ubique HTCC1062                          proteobacteria>alphaproteobacteria
69926560       208    <-ThiC<-?<-ThiE*<-ThiG<-ThiS<-ThiO                                                                                      Nitrobacter hamburgensis X14                                     proteobacteria>alphaproteobacteria
68192290       206    ThiC->ThiO->ThiS->ThiG->ThiE*->ThiD-><-OmpA                                                                             Mesorhizobium sp. BNC1                                           proteobacteria>alphaproteobacteria
14025575       201    <-ThiD<-ThiE*<-ThiG<-ThiS<-ThiO<-ThiC                                                                                   Mesorhizobium loti MAFF303099                                    proteobacteria>alphaproteobacteria
23011961       189    ThiO->ThiS->ThiG->ThiE*->                                                                                               Magnetospirillum magnetotacticum MS-1;                           proteobacteria>alphaproteobacteria
13423327       269    <-ThiG*<-ThiS                                                                                                           Caulobacter crescentus CB15                                      proteobacteria>alphaproteobacteria
84703985       259    <-phosphatidylglycerophosphate_synthase<-?<-?||ThiS->ThiG*->                                                            Parvularcula bermudensis HTCC2503                                proteobacteria>alphaproteobacteria
17741060       257    <-ThiG*<-ThiS<-ThiO<-ThiC                                                                                               Agrobacterium tumefaciens str. C58                               proteobacteria>alphaproteobacteria
58417134       266    <-ThiG*<-ThiS                                                                                                           Ehrlichia ruminantium str. Gardel                                proteobacteria>alphaproteobacteria
56416397       264    ThiS->ThiG*->                                                                                                           Anaplasma marginale str. St. Maries                              proteobacteria>alphaproteobacteria
83858498       262    <-ThiG*<-ThiS                                                                                                           Oceanicaulis alexandrii HTCC2633                                 proteobacteria>alphaproteobacteria
68538042       256    <-ThiG*<-ThiS                                                                                                           Sphingopyxis alaskensis RB2256                                   proteobacteria>alphaproteobacteria
78698311       202    <-ThiC<-ThiE*<-ThiG<-ThiS<-ThiO                                                                                         Bradyrhizobium sp. BTAi1                                         proteobacteria>alphaproteobacteria
72394551       261    <-ThiG*<-ThiS                                                                                                           Ehrlichia canis str. Jake                                        proteobacteria>alphaproteobacteria
74022860       312    ThiE-><-?||?-><-ThiE*<-ThiG<-ThiS<-ThiO                                                                                 Rhodoferax ferrireducens DSM 15236                               proteobacteria>betaproteobacteria
74019423       374    ThiO->ThiS->ThiG->ThiE*->Mopterin_binding_protein->                                                                     Burkholderia ambifaria AMMD;                                     proteobacteria>betaproteobacteria
7227331        205    ThiO->ThiE*->ThiS->ThiG->                                                                                               Neisseria meningitidis MC58                                      proteobacteria>betaproteobacteria
84713028       270    ThiC->ThiO->ThiS->ThiG->ThiE*->                                                                                         Polaromonas naphthalenivorans CJ2                                proteobacteria>betaproteobacteria
72117331       290    ThiC->ThiO->ThiS->ThiG->ThiE->?-><-?<-ThiD*||?->?->?->?->ThiS->                                                         Ralstonia eutropha JMP134                                        proteobacteria>betaproteobacteria
30138189       268    <-methylase<-ThiG*<-ThiS                                                                                                Nitrosomonas europaea ATCC 19718                                 proteobacteria>betaproteobacteria
82701205       264    <-methylase<-ThiG*<-ThiS                                                                                                Nitrosospira multiformis ATCC 25196                              proteobacteria>betaproteobacteria
71849093       260    <-methylase<-ThiG*<-ThiS<-ADH                                                                                           Dechloromonas aromatica RCB                                      proteobacteria>betaproteobacteria
68554870       276    ThiC->ThiO->ThiS->ThiG->ThiE->?-><-?<-ThiE*||?->?->?->?->ThiS->                                                         Ralstonia metallidurans CH34                                     proteobacteria>betaproteobacteria
34499221       264    <-ThiG*<-ThiS                                                                                                           Chromobacterium violaceum ATCC 12472                             proteobacteria>betaproteobacteria
47571796       176    ThiC->ThiO->ThiS->ThiG->ThiD*->                                                                                         Rubrivivax gelatinosus PM1                                       proteobacteria>betaproteobacteria
17427116       383    <-ThiE*<-ThiG||?-><-ThiS<-ThiO<-ThiC                                                                                    Ralstonia solanacearum;                                          proteobacteria>betaproteobacteria
74318144       262    <-methylase<-ThiG*<-ThiS                                                                                                Thiobacillus denitrificans ATCC 25259                            proteobacteria>betaproteobacteria
68212742       259    <-ThiG*<-ThiS                                                                                                           Methylobacillus flagellatus KT                                   proteobacteria>betaproteobacteria
77544040       206    ThiC->ThiS->ThiG->ThiH->?->ThiE*->                                                                                      Pelobacter carbinolicus DSM 2380                                 proteobacteria>deltaproteobacteria
78219006       214    ThiS->ThiG->ThiH->ThiF->ThiE*->                                                                                         Desulfovibrio desulfuricans G20;                                 proteobacteria>deltaproteobacteria
86158938       203    <-ThiE*||?-><-ThiG<-ThiS                                                                                                Anaeromyxobacter dehalogenans 2CP-C                              proteobacteria>deltaproteobacteria
71836232       223    <-ThiE*<-ThiG<-ThiS                                                                                                     Pelobacter propionicus DSM 2379                                  proteobacteria>deltaproteobacteria
71545062       222    ThiF->ThiG->ThiH->ThiS->ThiE*->?-><-?<-Mopterin_binding_protein                                                         Syntrophobacter fumaroxidans MPOB                                proteobacteria>deltaproteobacteria
85859826       229    <-ThiF<-ThiH<-ThiG<-ThiS<-ThiE*||?-><-?||?->Cysteine_synthase->                                                         Syntrophus aciditrophicus SB                                     proteobacteria>deltaproteobacteria
39982458       213    <-ThiE*<-ThiG<-ThiS                                                                                                     Geobacter sulfurreducens PCA                                     proteobacteria>deltaproteobacteria
78195386       213    ThiS->ThiG->ThiE*->                                                                                                     Geobacter metallireducens GS-15                                  proteobacteria>deltaproteobacteria
68178162       203    ThiF->ThiS->ThiG->ThiH->ThiE*->                                                                                         Desulfuromonas acetoxidans DSM 684;                              proteobacteria>deltaproteobacteria
50876628       263    ThiS->ThiG*->ThiH->                                                                                                     Desulfotalea psychrophila LSv54                                  proteobacteria>deltaproteobacteria
77544304       208    <-ThiE*<-ThiH<-ThiG<-ThiS<-ThiF                                                                                         Pelobacter carbinolicus DSM 2380                                 proteobacteria>deltaproteobacteria
46449915       226    <-ThiE*<-ThiF<-ThiH<-ThiG<-ThiS                                                                                         Desulfovibrio vulgaris subsp. vulgaris str. Hildenborough        proteobacteria>deltaproteobacteria
57166733       201    <-ThiE*<-ThiH<-ThiG<-ThiF<-ThiS                                                                                         Campylobacter jejuni RM1221                                      proteobacteria>epsilonproteobacteria
57240558       200    ThiS->ThiF->ThiG->ThiH->ThiE*->                                                                                         Campylobacter lari RM2100                                        proteobacteria>epsilonproteobacteria
86155162       253    ThiS->ThiF->ThiG*->ThiH->                                                                                               Campylobacter fetus subsp. fetus 82-40                           proteobacteria>epsilonproteobacteria
56178885       504    ThiC->ThiD+ThiE*->ThiF->ThiS->ThiG->ThiH->                                                                              Idiomarina loihiensis L2TR;                                      proteobacteria>gammaproteobacteria
83643050       487    ThiC->ThiO->ThiS->ThiG->ThiD+ThiE*->                                                                                    Hahella chejuensis KCTC 2396;                                    proteobacteria>gammaproteobacteria
45437723       229    ThiC->ThiE*->ThiF->ThiS->ThiG->ThiH->                                                                                   Yersinia pestis biovar Medievalis str. 91001                     proteobacteria>gammaproteobacteria
51587926       215    <-ThiH<-ThiG<-ThiS<-ThiF<-ThiE*<-ThiC                                                                                   Yersinia pseudotuberculosis IP 32953                             proteobacteria>gammaproteobacteria
12518922       211    <-ThiH<-ThiG<-ThiS<-ThiF<-ThiE*<-ThiC                                                                                   Escherichia coli O157:H7 EDL933;                                 proteobacteria>gammaproteobacteria
49609718       213    <-ThiH<-ThiG<-ThiS<-ThiF<-ThiE*<-ThiC                                                                                   Erwinia carotovora subsp. atroseptica SCRI1043                   proteobacteria>gammaproteobacteria
77960646       217    ThiC->ThiE*->ThiF->ThiS->ThiG->ThiH->                                                                                   Yersinia mollaretii ATCC 43969                                   proteobacteria>gammaproteobacteria
75855406       471    <-ThiH<-ThiG<-ThiS<-ThiF<-ThiE*<-ThiC<-CcrB                                                                             Vibrio sp. Ex25;                                                 proteobacteria>gammaproteobacteria
68542221       650    <-ThiH<-ThiG<-ThiS<-ThiF<-ThiD+ThiE*<-ThiC                                                                              Shewanella baltica OS155;                                        proteobacteria>gammaproteobacteria
29540946       479    ThiC->ThiO->ThiS->ThiG->ThiD+ThiE*->                                                                                    Coxiella burnetii RSA 493;                                       proteobacteria>gammaproteobacteria
71145222       529    ThiC->ThiO->ThiS->ThiG->ThiD+ThiE*->                                                                                    Colwellia psychrerythraea 34H;                                   proteobacteria>gammaproteobacteria
69953446       559    <-ThiH<-ThiG<-ThiS<-ThiF<-ThiD+ThiE*<-ThiC                                                                              Shewanella frigidimarina NCIMB 400                               proteobacteria>gammaproteobacteria
53751266       488    ThiO->ThiS->ThiG->ThiD+ThiE*->ThiF->                                                                                    Legionella pneumophila str. Paris;                               proteobacteria>gammaproteobacteria
36783918       216    <-ThiH<-ThiG<-ThiS<-ThiF<-ThiE*<-ThiC                                                                                   Photorhabdus luminescens subsp. laumondii TTO1                   proteobacteria>gammaproteobacteria
87119893       203    <-ThiE*<-ThiG<-ThiS<-ThiO<-?<-?<-?<-Mopterin_binding_protein                                                            Marinomonas sp. MED121                                           proteobacteria>gammaproteobacteria
76874359       508    ThiC->ThiO->ThiS->ThiG->ThiD+ThiE*->ThiF->                                                                              Pseudoalteromonas haloplanktis TAC125;                           proteobacteria>gammaproteobacteria
78362775       218    <-ThiE<-ThiE*<-ThiG<-ThiS<-ThiO<-ThiC                                                                                   Thiomicrospira crunogena XCL-2                                   proteobacteria>gammaproteobacteria
28808052       444    <-ThiH<-ThiG<-ThiS<-ThiF<-ThiE*<-ThiC<-CcrB                                                                             Vibrio parahaemolyticus RIMD 2210633                             proteobacteria>gammaproteobacteria
77977810       226    <-ThiH<-ThiG<-ThiS<-ThiF<-ThiE*<-ThiC                                                                                   Yersinia intermedia ATCC 29909                                   proteobacteria>gammaproteobacteria
77972243       226    ThiC->ThiE*->ThiF->ThiS->ThiG->ThiH->                                                                                   Yersinia frederiksenii ATCC 33641                                proteobacteria>gammaproteobacteria
68514852       525    <-ThiH<-ThiG<-ThiS<-ThiF<-ThiE*<-ThiC                                                                                   Shewanella amazonensis SB2B                                      proteobacteria>gammaproteobacteria
77957006       216    ThiC->ThiE*->ThiF->ThiS->ThiG->ThiH->                                                                                   Yersinia bercovieri ATCC 43970 ;                                 proteobacteria>gammaproteobacteria
37200142       444    <-ThiH<-ThiG<-ThiS<-ThiF<-ThiE*<-ThiC<-CcrB                                                                             Vibrio vulnificus YJ016                                          proteobacteria>gammaproteobacteria
69156747       613    ThiC->ThiE*->ThiF->ThiS->ThiG->ThiH->                                                                                   Shewanella denitrificans OS217                                   proteobacteria>gammaproteobacteria
84393668       430    <-ThiH<-ThiG<-ThiS<-ThiF<-ThiE*<-ThiC<-CcrB                                                                             Vibrio splendidus 12B01                                          proteobacteria>gammaproteobacteria
78366585       581    <-ThiH<-ThiG<-ThiS<-ThiF<-ThiD+ThiE*<-ThiC                                                                              Shewanella sp. PV-4                                              proteobacteria>gammaproteobacteria
78362774       281    <-ThiD*<-ThiE<-ThiG<-ThiS<-ThiO<-ThiC                                                                                   Thiomicrospira crunogena XCL-2                                   proteobacteria>gammaproteobacteria
9654457        440    CcrB->ThiC->ThiE*->ThiF->ThiS->ThiG->ThiH->                                                                             Vibrio cholerae O1 biovar eltor str. N16961                      proteobacteria>gammaproteobacteria
16422721       211    <-ThiH<-ThiG<-ThiS<-ThiF<-ThiE*<-ThiC                                                                                   Salmonella typhimurium LT2                                       proteobacteria>gammaproteobacteria
24373991       526    <-ThiH<-ThiG<-ThiS<-ThiF<-ThiD+ThiE*<-ThiC                                                                              Shewanella oneidensis MR-1                                       proteobacteria>gammaproteobacteria
76793993       276    <-ThiF<-?<-ThiG*<-ThiS<-ThiO<-ThiC                                                                                      Pseudoalteromonas atlantica T6c                                  proteobacteria>gammaproteobacteria
49530466       261    <-ThiG*<-ThiS                                                                                                           Acinetobacter sp. ADP1                                           proteobacteria>gammaproteobacteria
26991780       270    <-methylase<-ThiG*<-ThiS<-?||?-><-?<-?<-Mopterin_binding_protein                                                        Pseudomonas putida KT2440                                        proteobacteria>gammaproteobacteria
71555612       264    <-methylase<-ThiG*<-ThiS<-?||?-><-?<-?<-Mopterin_binding_protein                                                        Pseudomonas syringae pv. phaseolicola 1448A                      proteobacteria>gammaproteobacteria
67677083       266    ThiS->ThiG*->methylase->                                                                                                Chromohalobacter salexigens DSM 3043                             proteobacteria>gammaproteobacteria
68347434       264    <-methylase<-ThiG*<-ThiS<-?||?-><-?<-?<-Mopterin_binding_protein                                                        Pseudomonas fluorescens Pf-5                                     proteobacteria>gammaproteobacteria
77953947       269    <-ThiG*<-ThiS                                                                                                           Marinobacter aquaeolei VT8                                       proteobacteria>gammaproteobacteria
67154906       264    <-methylase<-ThiG*<-ThiS<-?||?-><-JAB                                                                                   Azotobacter vinelandii AvOP                                      proteobacteria>gammaproteobacteria
78701989       262    ThiS->ThiG*->methylase->                                                                                                Alkalilimnicola ehrlichei MLHE-1                                 proteobacteria>gammaproteobacteria
48862780       269    ThiO->ThiS->ThiG*->?->?->Mopterin_binding_protein->                                                                     Microbulbifer degradans 2-40                                     proteobacteria>gammaproteobacteria
21109645       264    ThiS->ThiG*->methylase->                                                                                                Xanthomonas axonopodis pv. citri str. 306                        proteobacteria>gammaproteobacteria
9105679        275    ThiS->ThiG*->methylase->                                                                                                Xylella fastidiosa 9a5c                                          proteobacteria>gammaproteobacteria
71900706       275    <-methylase<-ThiG*<-ThiS                                                                                                Xylella fastidiosa Ann-1                                         proteobacteria>gammaproteobacteria

Bacterial ThiSs fused to ThiG (Gis are of the ThiS+ThiG protein-marked with an asterisk)
^^^^^^^^^^^^^^^^^^^^^^^^^^^^^^
GI             LENGTH     Operon                                                                                                              ORGANISM                                                         Classification                                Protein descriptions (if any)
68512207       252    ThiE->ThiS+ThiG*->                                                                                                      Rubrobacter xylanophilus DSM 9941;                               actinobacteria                                Thiamine monophosphate synthase [Rubrobacter xylanophilus DSM 9941]
79039407       331    ThiS+ThiG*                                                                                                              Novosphingobium aromaticivorans DSM 12444;                       proteobacteria>alphaproteobacteria            similar to Uncharacterized enzyme of thiazole biosynthesis [Novosphingobium aromaticivorans DSM 12444]
56551634       331    ThiS+ThiG*                                                                                                              Zymomonas mobilis subsp. mobilis ZM4;                            proteobacteria>alphaproteobacteria            thiazole biosynthesis protein [Zymomonas mobilis subsp. mobilis ZM4]
84788478       332    ThiS+ThiG*                                                                                                              Erythrobacter litoralis HTCC2594;                                proteobacteria>alphaproteobacteria            thiazole biosynthesis protein [Erythrobacter litoralis HTCC2594]
85709842       333    ThiS+ThiG*                                                                                                              Erythrobacter sp. NAP1;                                          proteobacteria>alphaproteobacteria            thiazole biosynthesis protein [Erythrobacter sp. NAP1]
83576681       334    ThiS+ThiG*                                                                                                              Rhodospirillum rubrum ATCC 11170;                                proteobacteria>alphaproteobacteria            ThiS, thiamine-biosynthesis [Rhodospirillum rubrum ATCC 11170]
76883424       347    ThiS+ThiG*                                                                                                              Nitrosococcus oceani ATCC 19707;                                 proteobacteria>gammaproteobacteria            ThiS, thiamine-biosynthesis [Nitrosococcus oceani ATCC 19707]
68246504       326    ThiS+ThiG*                                                                                                              Magnetococcus sp. MC-1;                                          proteobacteria                                ThiS, thiamine-biosynthesis [Magnetococcus sp. MC-1]
46202840       330    ThiS+ThiG*                                                                                                              Magnetospirillum magnetotacticum MS-1;                           proteobacteria>alphaproteobacteria            COG2022: Uncharacterized enzyme of thiazole biosynthesis [Magnetospirillum magnetotacticum MS-1]
53758359       326    ThiS+ThiG*                                                                                                              Methylococcus capsulatus str. Bath;                              proteobacteria>gammaproteobacteria            thiamine biosynthesis protein ThiS [Methylococcus capsulatus str. Bath]
82701206       162    ThiS->ThiG*->                                                                                                           Nitrosospira multiformis ATCC 25196;                             proteobacteria>betaproteobacteria             thiamine biosynthesis protein ThiS [Nitrosospira multiformis ATCC 25196]

Archaeal ThiS solos (Gis are for the ThiS protein -marked with an asterisk)
^^^^^^^^^^^^^^^^^^^^
GI             LENGTH     Operon (no particularly conserved operons were detected)                                                            ORGANISM (gis are of the ThiS protein)                           Classification                                Protein descriptions (if any)
48425680       77                                                                                                                             Pyrococcus furiosus DSM 3638                                     euryarchaeota                                 A Chain A, Backbone Solution Structure Of Mixed AlphaBETA PROTEIN Pf1061
33359535       71                                                                                                                             Pyrococcus furiosus DSM 3638                                     euryarchaeota                                 sulfur carrier protein ThiS [Pyrococcus furiosus DSM 3638]
18893126       69                                                                                                                             Pyrococcus furiosus DSM 3638                                     euryarchaeota                                 hypothetical protein [Pyrococcus furiosus DSM 3638]
19916735       77                                                                                                                             Methanosarcina acetivorans C2A                                   euryarchaeota                                 predicted protein [Methanosarcina acetivorans C2A]
19916952       77                                                                                                                             Methanosarcina acetivorans C2A                                   euryarchaeota                                 predicted protein [Methanosarcina acetivorans C2A]
13540947       68                                                                                                                             Thermoplasma volcanium GSS1                                      euryarchaeota                                 hypothetical protein TVN0116 [Thermoplasma volcanium GSS1]
14324330       64                                                                                                                             Thermoplasma volcanium GSS1                                      euryarchaeota                                 hypothetical protein [Thermoplasma volcanium GSS1]
10581690       174                                                                                                                            Halobacterium sp. NRC-1                                          euryarchaeota                                 Vng2279h [Halobacterium sp. NRC-1]
18893610       73                                                                                                                             Pyrococcus furiosus DSM 3638                                     euryarchaeota                                 hypothetical protein [Pyrococcus furiosus DSM 3638]
2622875        70                                                                                                                             Methanothermobacter thermautotrophicus str. Delta H              euryarchaeota                                 unknown [Methanothermobacter thermautotrophicus str. Delta H]
19917335       70                                                                                                                             Methanosarcina acetivorans C2A                                   euryarchaeota                                 predicted protein [Methanosarcina acetivorans C2A]
21226239       70                                                                                                                             Methanosarcina mazei Go1                                         euryarchaeota                                 hypothetical protein MM0137 [Methanosarcina mazei Go1]
68211447       70                                                                                                                             Methanococcoides burtonii DSM 6242                               euryarchaeota                                 hypothetical protein MburDRAFT_0612 [Methanococcoides burtonii DSM 6242]
72398144       70                                                                                                                             Methanosarcina barkeri str. fusaro                               euryarchaeota                                 conserved hypothetical protein [Methanosarcina barkeri str. fusaro]
33356745       69                                                                                                                             Pyrococcus abyssi GE5                                            euryarchaeota                                 sulfur carrier protein ThiS [Pyrococcus abyssi GE5]
88951090       69                                                                                                                             Methanosaeta thermophila PT                                      euryarchaeota                                 conserved hypothetical protein [Methanosaeta thermophila PT]
48430257       64                                                                                                                             Picrophilus torridus DSM 9790                                    euryarchaeota                                 hypothetical protein PTO0537 [Picrophilus torridus DSM 9790]
44920975       64                                                                                                                             Methanococcus maripaludis S2                                     euryarchaeota                                 hypothetical protein [Methanococcus maripaludis S2]
10640784       67                                                                                                                             Thermoplasma acidophilum                                         euryarchaeota                                 hypothetical protein [Thermoplasma acidophilum]
11498344       67                                                                                                                             Archaeoglobus fulgidus DSM 4304                                  euryarchaeota                                 hypothetical protein AF0737 [Archaeoglobus fulgidus DSM 4304]
14591747       67                                                                                                                             Pyrococcus horikoshii OT3                                        euryarchaeota                                 sulfur carrier protein ThiS [Pyrococcus horikoshii OT3]
57159352       67                                                                                                                             Thermococcus kodakarensis KOD1                                   euryarchaeota                                 sulfur transfer protein involved in thiamine biosynthesis [Thermococcus kodakarensis KOD1]
55379215       66                                                                                                                             Haloarcula marismortui ATCC 43049                                euryarchaeota                                 hypothetical protein rrnAC2563 [Haloarcula marismortui ATCC 43049]
76801103       66                                                                                                                             Natronomonas pharaonis DSM 2160                                  euryarchaeota                                 homolog to thiamine biosynthesis protein ThiS (probable sulfur donor) [Natronomonas pharaonis DSM 2160]
84489151       66                                                                                                                             Methanosphaera stadtmanae DSM 3091                               euryarchaeota                                 hypothetical protein Msp_0330 [Methanosphaera stadtmanae DSM 3091]
68141055       64                                                                                                                             Ferroplasma acidarmanus Fer1                                     euryarchaeota                                 conserved hypothetical protein [Ferroplasma acidarmanus Fer1]
15622711       68                                                                                                                             Sulfolobus tokodaii str. 7                                       crenarchaeota                                 68aa long conserved hypothetical protein [Sulfolobus tokodaii str. 7]
68568033       68                                                                                                                             Sulfolobus acidocaldarius DSM 639                                crenarchaeota                                 conserved Archaeal protein [Sulfolobus acidocaldarius DSM 639]

B. Variant Thiamine biosynthesis pathway (Gis are for the ThiS+ThiF protein- marked with an asterisk)
^^^^^^^^^^^^^^^^^^^^^^^^^^^^^^^^^^^^^^^^^^^^^^^^^^^^^^^^^^^^^^^^^^^^^^^^^^^^
GI             LENGTH     Operon                                                                                                              ORGANISM (gis are of the ThiS+ThiF protein)                          Classification                                 Protein descriptions (if any)
57240561       265     ThiS->ThiS+ThiF*->ThiG->ThiH->ThiE->                                                                                   Campylobacter lari RM2100                                        proteobacteria>epsilonproteobacteria          HesA/MoeB/ThiF family protein [Campylobacter lari RM2100]
57168916       266     ThiS->ThiS+ThiF*->ThiG->ThiH->ThiE->                                                                                   Campylobacter coli RM2228                                        proteobacteria>epsilonproteobacteria          HesA/MoeB/ThiF family protein [Campylobacter coli RM2228]
57166736       267     ThiS->ThiS+ThiF*->ThiG->ThiH->ThiE->                                                                                   Campylobacter jejuni RM1221                                      proteobacteria>epsilonproteobacteria          thiamine biosynthesis protein ThiF [Campylobacter jejuni RM1221]
86152451       267     ThiS->ThiS+ThiF*->ThiG->ThiH->ThiE->                                                                                   Campylobacter jejuni subsp. jejuni HB93-13                       proteobacteria>epsilonproteobacteria          thiamine biosynthesis protein ThiF [Campylobacter jejuni subsp. jejuni HB93-13]
86150511       267     ThiS->ThiS+ThiF*->ThiG->ThiH->ThiE->                                                                                   Campylobacter jejuni subsp. jejuni CF93-6                        proteobacteria>epsilonproteobacteria          thiamine biosynthesis protein ThiF [Campylobacter jejuni subsp. jejuni CF93-6]
86150854       267     ThiS->ThiS+ThiF*->ThiG->ThiH->ThiE->                                                                                   Campylobacter jejuni subsp. jejuni 260.94                        proteobacteria>epsilonproteobacteria          thiamine biosynthesis protein ThiF [Campylobacter jejuni subsp. jejuni 260.94]
87132835       267     ThiS->ThiS+ThiF*->ThiG->ThiH->ThiE->                                                                                   Campylobacter jejuni subsp. jejuni 84-25                         proteobacteria>epsilonproteobacteria          COG0476: Dinucleotide-utilizing enzymes involved in molybdopterin and thiamine biosynthesis                                               family 2 [Campylobacter jejuni subsp. jejuni 84-25]
71837115       267     OAHShyd->OAHShyd->Cyssynthase->ThiS+ThiF*-> (operon gene displacement)                                                 Pelobacter propionicus DSM 2379                                  proteobacteria>deltaproteobacteria            UBA/THIF-type NAD/FAD binding fold [Pelobacter propionicus DSM 2379]
77544308       268     ThiS+ThiF*->ThiS->ThiG->ThiH->ThiE->                                                                                   Pelobacter carbinolicus DSM 2380                                 proteobacteria>deltaproteobacteria            molybdopterin biosynthesis protein MoeB [Pelobacter carbinolicus DSM 2380]
68178158       272     ThiS+ThiF*->ThiS->ThiG->ThiH->ThiE->                                                                                   Desulfuromonas acetoxidans DSM 684                               proteobacteria>deltaproteobacteria            UBA/THIF-type NAD/FAD binding fold [Desulfuromonas acetoxidans DSM 684]
18145265       269     ThiS->ThiS+ThiF*->ThiG->ThiH->ThiE->                                                                                   Clostridium perfringens str. 13                                  firmicutes                                    probable molybdopterin biosynthesis protein [Clostridium perfringens str. 13]
82748786       267     ThiS+ThiF*->ThiE->                                                                                                     Clostridium beijerincki NCIMB 8052                               firmicutes                                    UBA/THIF-type NAD/FAD binding fold [Clostridium beijerincki NCIMB 8052]
28203841       267     ThiD->ThiM->ThiE->ThiS+ThiF*->ThiG->ThiH->                                                                             Clostridium tetani E88                                           firmicutes                                    molybdopterin biosynthesis protein moeB [Clostridium tetani E88]
77683437       268     ThiS->ThiS+ThiF*->ThiG->ThiH->ThiC->ThiE->                                                                             Alkaliphilus metalliredigenes QYMF                               firmicutes                                    UBA/THIF-type NAD/FAD binding fold [Alkaliphilus metalliredigenes QYMF]
15025973       266     ThiS->ThiS+ThiF*->ThiG->ThiH->ThiE->                                                                                   Clostridium acetobutylicum ATCC 824                              firmicutes                                    AE007789_11 Dinucleotide-utilizing enzyme involved in molybdopterin/thiamine biosynthesis [Cl                                              ostridium acetobutylicum ATCC 824]

Thiamine biosynthesis pathways in operons with a Cys synthase (gis are for the Cys synthase (Cys syn)- marked with an asterisk)
^^^^^^^^^^^^^^^^^^^^^^^^^^^^^^^^^^^^^^^^^^^^^^^^^^^^^^^^^^^^^^^^^^^^^^^^^^^^^^^^^^^^^^^^^^^^
GI             LENGTH     Operon                                                                                                              ORGANISM                                                         Classification                                  Protein descriptions (if any)
85859830       295    ThiF->ThiH->ThiG->ThiS<-?->?<-UbiA->Cys synthase*->                                                                     Syntrophus aciditrophicus SB                                     proteobacteria>deltaproteobacteria            cysteine synthase [Syntrophus aciditrophicus SB]
21646639       310    trans sulf->Cys synthase*->ThiS->ThiG->ThiH->                                                                           Chlorobium tepidum TLS                                           bacteroidetes/chlorobi                        cysteine synthase [Chlorobium tepidum TLS]
77545399       308    Cys syn*->OAHSH->ThiF->ThiS solo-> (probably molybdenum biosynthesis?)                                                  Pelobacter carbinolicus DSM 2380                                 proteobacteria>deltaproteobacteria            cysteine synthase [Pelobacter carbinolicus DSM 2380]

Miscellaneous pathway
67938818       328    Rrf2 (often fused to NifS)->Cys Synthase*->ThiS                                                                         Chlorobium phaeobacteroides BS1                                  bacteroidetes/chlorobi                        Cysteine synthase K/M:Cysteine synthase A [Chlorobium phaeobacteroides BS1]
-------------------------------------------------------------------------------------------------------------

2. Classical pathway: Molybdopterin cofactor biosynthesis and related pathways
^^^^^^^^^^^^^^^^^^^^^^^^^^^^^^^^^^^^^^^^^^^^^^^^^^^^^^^^^^^^^^^^^^^^^^
a.Bacterial versions of classical MOCO factor biosynthesis pathway (The gis represent the MoaE protein- marked with an asterisk)
^^^^^^^^^^^^^^^^^^^^^^^^^^^^^^^^^^^^^^^^^^^^^^^^^^^^^^^^^^^^^^^^^^^
GI             LENGTH     Operon                                                                                                              ORGANISM                                                         Classification                                Protein descriptions (if any)
32033744       151     moaA->MoaC->MoaD->MoaE*->                                                                                              Actinobacillus pleuropneumoniae serovar 1 str. 4074;             proteobacteria>gammaproteobacteria            COG0314: Molybdopterin converting factor, large subunit [Actinobacillus pleuropneumoniae serovar 1 str. 4074]
75431112       159     moaA->MoaC->MoaD->MoaE*->                                                                                              Actinobacillus succinogenes 130Z                                 proteobacteria>gammaproteobacteria            molybdopterin converting factor, large subunit [Actinobacillus succinogenes 130Z]
45435690       152     <-MoaE*<-MoaD<-MoaC<-moaA                                                                                              Yersinia pestis biovar Medievalis str. 91001                     proteobacteria>gammaproteobacteria            molybdopterin [mpt] converting factor, subunit 2 [Yersinia pestis biovar Medievalis str. 91001]
71038168       184     MoeA->moaA->MoaB->?->MoaC->MoaD->MoaE*->Mo_transporter->permease->                                                     Psychrobacter arcticus 273-4                                     proteobacteria>gammaproteobacteria            probable molybdopterin converting factor, large subunit [Psychrobacter arcticus 273-4]
67156788       148     MoaC->MoaD->MoaE*->                                                                                                    Azotobacter vinelandii AvOP                                      proteobacteria>gammaproteobacteria            Molybdopterin biosynthesis MoaE [Azotobacter vinelandii AvOP]
28868460       148     MoaC->MoaD->MoaE*->                                                                                                    Pseudomonas syringae pv. tomato str. DC3000                      proteobacteria>gammaproteobacteria            molybdenum cofactor biosynthesis protein E [Pseudomonas syringae pv. tomato str. DC3000]
21107238       146     MoaC->MoaD->MoaE*->                                                                                                    Xanthomonas axonopodis pv. citri str. 306                        proteobacteria>gammaproteobacteria            molybdopterin-converting factor chain 2 [Xanthomonas axonopodis pv. citri str. 306]
26988029       148     MoaC->MoaD->MoaE*->                                                                                                    Pseudomonas putida KT2440                                        proteobacteria>gammaproteobacteria            molybdenum cofactor biosynthesis protein E [Pseudomonas putida KT2440]
77381196       150     MoaC->MoaD->MoaE*->                                                                                                    Pseudomonas fluorescens PfO-1                                    proteobacteria>gammaproteobacteria            Molybdopterin biosynthesis MoaE [Pseudomonas fluorescens PfO-1]
68345385       152     <-MoaB||MobA->?->?-><-MoaE*<-MoaD<-MoaC<-?<-moaA                                                                       Pseudomonas fluorescens Pf-5                                     proteobacteria>gammaproteobacteria            molybdenum cofactor biosynthesis protein E [Pseudomonas fluorescens Pf-5]
77382375       153     <-MoaE*<-MoaD<-MoaC<-?<-?||MoaB->MoeA->                                                                                Pseudomonas fluorescens PfO-1                                    proteobacteria>gammaproteobacteria            Molybdopterin biosynthesis MoaE [Pseudomonas fluorescens PfO-1]
68342721       150     MoaC->MoaD->MoaE*->                                                                                                    Pseudomonas fluorescens Pf-5                                     proteobacteria>gammaproteobacteria            molybdopterin converting factor, subunit 2 [Pseudomonas fluorescens Pf-5]
84319277       150     <-MoeA<-MoaB<-MoaE*<-MoaD                                                                                              Pseudomonas aeruginosa C3719                                     proteobacteria>gammaproteobacteria            COG0314: Molybdopterin converting factor, large subunit [Pseudomonas aeruginosa C3719]
76791053       154     <-permease<-Mo_transporter<-MoaE*<-MoaD<-MoaC<-MoaB<-?<-moaA                                                           Pseudoalteromonas atlantica T6c                                  proteobacteria>gammaproteobacteria            Molybdopterin biosynthesis MoaE [Pseudoalteromonas atlantica T6c]
36784881       150     moaA->MoaC->MoaD->MoaE*->                                                                                              Photorhabdus luminescens subsp. laumondii TTO1                   proteobacteria>gammaproteobacteria            molybdopterin [MPT] converting factor, subunit 2 (molybdenum cofactor biosynthesis protein E) (molybdopterin converting factor large subunit) [Photorhabdus luminescens subsp. laumondii TTO1]
37198129       151     moaA->MoaB->MoaC->MoaD->MoaE*->?->?->?->Mopterin_binding_protein->                                                     Vibrio vulnificus YJ016                                          proteobacteria>gammaproteobacteria            molybdenum cofactor biosynthesis protein E [Vibrio vulnificus YJ016]
46912748       149     moaA->MoaC->MoaD->MoaE*->                                                                                              Photobacterium profundum SS9                                     proteobacteria>gammaproteobacteria            putative molybdenum cofactor biosynthesisprotein E [Photobacterium profundum SS9]
67676069       152     <-moaA<-MoaE*<-MoaD<-MoeA<-mobB<-MobA<-Mopterin_binding_protein<-permease                                              Chromohalobacter salexigens DSM 3043                             proteobacteria>gammaproteobacteria            Molybdopterin biosynthesis MoaE [Chromohalobacter salexigens DSM 3043]
71144053       156     <-MoeA||moaA->?->MoaB->MoaC->MoaD->MoaE*->Mo_transporter->permease->Mopterin_binding_protein->MoeB->                   Colwellia psychrerythraea 34H                                    proteobacteria>gammaproteobacteria            molybdopterin converting factor, subunit 2 [Colwellia psychrerythraea 34H]
12720896       150     <-MoaE*<-MoaD<-MoaC<-moaA                                                                                              Pasteurella multocida subsp. multocida str. Pm70                 proteobacteria>gammaproteobacteria            MoaE [Pasteurella multocida subsp. multocida str. Pm70]
49612263       150     <-MoaE*<-MoaD<-MoaC<-MoaB<-moaA                                                                                        Erwinia carotovora subsp. atroseptica SCRI1043                   proteobacteria>gammaproteobacteria            molybdopterin converting factor subunit 2 [Erwinia carotovora subsp. atroseptica SCRI1043]
84393379       157     <-Mopterin_binding_protein<-?<-?<-?<-MoaE*<-MoaD<-MoaC<-MoaB<-moaA                                                     Vibrio splendidus 12B01                                          proteobacteria>gammaproteobacteria            Molybdenum cofactor biosynthesis protein E [Vibrio splendidus 12B01]
28807085       151     <-Mopterin_binding_protein<-?<-?<-?<-MoaE*<-MoaD<-MoaC<-MoaB<-moaA                                                     Vibrio parahaemolyticus RIMD 2210633                             proteobacteria>gammaproteobacteria            molybdenum cofactor biosynthesis protein E [Vibrio parahaemolyticus RIMD 2210633]
26107156       150     moaA->MoaB-><-?||MoaC->MoaD->MoaE*->                                                                                   Escherichia coli CFT073                                          proteobacteria>gammaproteobacteria            AE016757_244 Molybdopterin converting factor subunit 2 [Escherichia coli CFT073]
59711550       148     moaA->MoaC->MoaD->MoaE*->                                                                                              Vibrio fischeri ES114                                            proteobacteria>gammaproteobacteria            molybdopterin converting factor, large subunit [Vibrio fischeri ES114]
33148682       151     <-MoaE*<-MoaD<-MoaC<-moaA                                                                                              Haemophilus ducreyi 35000HP                                      proteobacteria>gammaproteobacteria            molybdopterin converting factor subunit 2 [Haemophilus ducreyi 35000HP]
1574523        150     <-MoaE*<-MoaD<-MoaC<-moaA                                                                                              Haemophilus influenzae Rd KW20                                   proteobacteria>gammaproteobacteria            molybdopterin converting factor, subunit 2 (moaE) [Haemophilus influenzae Rd KW20]
23467045       150     moaA->MoaC->MoaD->MoaE*->                                                                                              Haemophilus somnus 129PT                                         proteobacteria>gammaproteobacteria            COG0314: Molybdopterin converting factor, large subunit [Haemophilus somnus 129PT]
68545075       152     <-Mopterin_binding_protein<-permease<-Mo_transporter<-MoaE*<-MoaD<-MoaC<-MoaB<-moaA                                    Shewanella amazonensis SB2B                                      proteobacteria>gammaproteobacteria            Molybdopterin biosynthesis MoaE [Shewanella amazonensis SB2B]
69158642       156     <-Mopterin_binding_protein<-permease<-Mo_transporter<-MoaE*<-MoaD<-MoaC<-moaA                                          Shewanella denitrificans OS217                                   proteobacteria>gammaproteobacteria            Molybdopterin biosynthesis MoaE [Shewanella denitrificans OS217]
75819544       153     <-MoaE*<-MoaD<-MoaC<-MoaB<-moaA                                                                                        Vibrio cholerae V51                                              proteobacteria>gammaproteobacteria            COG0314: Molybdopterin converting factor, large subunit [Vibrio cholerae V51]
48861422       145     MoaC->MoaD->MoaE*->                                                                                                    Microbulbifer degradans 2-40                                     proteobacteria>gammaproteobacteria            COG0314: Molybdopterin converting factor, large subunit [Microbulbifer degradans 2-40]
52307131       161     moaA->MoaC->MoaD->MoaE*->                                                                                              Mannheimia succiniciproducens MBEL55E                            proteobacteria>gammaproteobacteria            MoaE protein [Mannheimia succiniciproducens MBEL55E]
77951749       148     MobA-><-MoaE*<-MoaD<-MoeA<-MoaB                                                                                        Marinobacter aquaeolei VT8                                       proteobacteria>gammaproteobacteria            molybdenum cofactor biosynthesis protein E [Marinobacter aquaeolei VT8]
87121810       151     <-MoaE*<-MoaD<-MoaC                                                                                                    Marinomonas sp. MED121                                           proteobacteria>gammaproteobacteria            molybdenum cofactor biosynthesis protein E [Marinomonas sp. MED121]
78362791       155     moaA->MoaD->MoaE*->MoeA->MoaC->                                                                                        Thiomicrospira crunogena XCL-2                                   proteobacteria>gammaproteobacteria            Molybdopterin biosynthesis MoaE [Thiomicrospira crunogena XCL-2]
53756579       151     MoaD->MoaE*->                                                                                                          Methylococcus capsulatus str. Bath                               proteobacteria>gammaproteobacteria            molybdopterin converting factor, subunit 2 [Methylococcus capsulatus str. Bath]
24375927       155     <-Mopterin_binding_protein<-permease<-Mo_transporter<-MoaE*<-MoaD<-MoaC<-moaA                                          Shewanella oneidensis MR-1                                       proteobacteria>gammaproteobacteria            molybdenum cofactor biosynthesis protein E [Shewanella oneidensis MR-1]
69951943       172     <-Mopterin_binding_protein<-permease<-Mo_transporter<-MoaE*<-MoaD<-MoaC<-moaA                                          Shewanella frigidimarina NCIMB 400                               proteobacteria>gammaproteobacteria            Molybdopterin biosynthesis MoaE [Shewanella frigidimarina NCIMB 400]
71364350       163     <-permease<-Mo_transporter<-MoaE*<-MoaD<-MoaC<-?<-MoaB<-moaA<-MoeA                                                     Psychrobacter cryohalolentis K5                                  proteobacteria>gammaproteobacteria            Molybdopterin biosynthesis MoaE [Psychrobacter cryohalolentis K5]
86154629       148     <-MoeA<-?<-?<-MoaE*<-MoaD                                                                                              Campylobacter fetus subsp. fetus 82-40                           proteobacteria>epsilonproteobacteria          molybdopterin converting factor, subunit 2 [Campylobacter fetus subsp. fetus 82-40]
78776455       145     MoaD->MoaE*->MoeA->                                                                                                    Thiomicrospira denitrificans ATCC 33889                          proteobacteria>epsilonproteobacteria          possible molybdopterin converting factor, subunit 2 [Thiomicrospira denitrificans ATCC 33889]
15645419       145     <-MoaC<-MoaB<-MoaE*<-MoaD                                                                                              Helicobacter pylori 26695                                        proteobacteria>epsilonproteobacteria          molybdopterin converting factor, subunit 2 (moaE) [Helicobacter pylori 26695]
32261594       157     MoeA->MoaD->MoaE*->mobB->MoaB->                                                                                        Helicobacter hepaticus ATCC 51449                                proteobacteria>epsilonproteobacteria          molybdopterin converting factor [Helicobacter hepaticus ATCC 51449]
57167345       147     MoaD->MoaE*->?->MoeA->                                                                                                 Campylobacter jejuni RM1221;                                     proteobacteria>epsilonproteobacteria          molybdopterin converting factor, subunit 2 [Campylobacter jejuni RM1221]
34483283       145     MoeA->MoaD->MoaE*->mobB->MoaB->MoaC->                                                                                  Wolinella succinogenes                                           proteobacteria>epsilonproteobacteria          POSSIBLE MOLYBDOPTERIN CONVERTING FACTOR, SUBUNIT 2 [Wolinella succinogenes]
57505250       151     MoaD->MoaE*->MoeA->                                                                                                    Campylobacter upsaliensis RM3195                                 proteobacteria>epsilonproteobacteria          molybdopterin converting factor, subunit 2 [Campylobacter upsaliensis RM3195]
34495640       158     <-MoaE*<-MoaD                                                                                                          Chromobacterium violaceum ATCC 12472                             proteobacteria>betaproteobacteria             molybdopterin converting factor subunit 2 [Chromobacterium violaceum ATCC 12472]
18076268       172     MoeA->MoaD->MoaE*->CcrB->                                                                                              Cupriavidus necator                                              proteobacteria>betaproteobacteria             molybdopterin synthase large subunit [Cupriavidus necator]
67907156       226     <-MoaE*||?-><-MoaD<-MoeA<-mobB<-Threonine_synthase                                                                     Polaromonas sp. JS666                                            proteobacteria>betaproteobacteria             Molybdopterin biosynthesis MoaE [Polaromonas sp. JS666]
74022613       163     <-MoaE*<-MoaD<-MoeA<-mobB<-Threonine_synthase                                                                          Rhodoferax ferrireducens DSM 15236                               proteobacteria>betaproteobacteria             Molybdopterin biosynthesis MoaE [Rhodoferax ferrireducens DSM 15236]
47573809       159     Threonine_synthase->mobB->MoeA->MoaD->?->MoaE*->CcrB->                                                                 Rubrivivax gelatinosus PM1                                       proteobacteria>betaproteobacteria             COG0314: Molybdopterin converting factor, large subunit [Rubrivivax gelatinosus PM1]
74317045       151     <-moaA||mobB->MoeA->MoaD->MoaE*->                                                                                      Thiobacillus denitrificans ATCC 25259                            proteobacteria>betaproteobacteria             molybdenum cofactor biosynthesis protein E [Thiobacillus denitrificans ATCC 25259]
83719603       166     Threonine_synthase->MoeA->MoaD->MoaE*->                                                                                Burkholderia thailandensis E264                                  proteobacteria>betaproteobacteria             molybdopterin converting factor, subunit 2 [Burkholderia thailandensis E264]
77964629       189     <-MoaD<-MoaE*<-moaA<-MoeA                                                                                              Burkholderia sp. 383                                             proteobacteria>betaproteobacteria             Molybdopterin biosynthesis MoaE [Burkholderia sp. 383]
67664216       190     MoeA->moaA->MoaE*->MoaD->                                                                                              Burkholderia cenocepacia HI2424                                  proteobacteria>betaproteobacteria             Molybdopterin biosynthesis MoaE [Burkholderia cenocepacia HI2424]
74018016       187     MoeA->moaA->MoaE*->MoaD->                                                                                              Burkholderia ambifaria AMMD;                                     proteobacteria>betaproteobacteria             Molybdopterin biosynthesis MoaE [Burkholderia ambifaria AMMD]
84713091       157     Threonine_synthase->mobB->MoeA->MoaD->MoaE*->                                                                          Polaromonas naphthalenivorans CJ2                                proteobacteria>betaproteobacteria             moaE, RSc1332; probable molybdopterin mpt converting      factor (subunit 2) protein [Polaromonas naphthalenivorans CJ2]
33563746       163     ModE->moaA-><-MoeA<-MoaB<-MoaE*<-MoaD<-MoaC                                                                            Bordetella pertussis Tohama I                                    proteobacteria>betaproteobacteria             molybdopterin converting factor [Bordetella pertussis Tohama I]
56315291       161     <-MoaE*<-MoaD<-MoeA<-mobB                                                                                              Azoarcus sp. EbN1                                                proteobacteria>betaproteobacteria             Molybdenum cofactor biosynthesis protein E [Azoarcus sp. EbN1]
68212269       149     <-MoaE*<-MoaD                                                                                                          Methylobacillus flagellatus KT                                   proteobacteria>betaproteobacteria             Molybdopterin biosynthesis MoaE [Methylobacillus flagellatus KT]
68557891       163     <-CcrB<-MoaE*<-MoaD<-MoeA<-Threonine_synthase                                                                          Ralstonia metallidurans CH34                                     proteobacteria>betaproteobacteria             Molybdopterin biosynthesis MoaE [Ralstonia metallidurans CH34]
17428347       176     Threonine_synthase->?->MoeA->MoaD->MoaE*->CcrB->                                                                       Ralstonia solanacearum                                           proteobacteria>betaproteobacteria             PROBABLE MOLYBDOPTERIN MPT CONVERTING FACTOR (SUBUNIT 2) PROTEIN [Ralstonia solanacearum]
86357114       153     <-MoaE*<-MoaD<-phosphatidylglycerophosphate_synthase<-Excinuclease<-ADH||OmpA->                                        Rhizobium etli CFN 42                                            proteobacteria>alphaproteobacteria            molybdopterin converting factor subunit 2 protein [Rhizobium etli CFN 42]
77389070       146     <-ADH||?->?->?-><-MoaE*<-MoaD<-phosphatidylglycerophosphate_synthase<-Excinuclease<-ADH                                Rhodobacter sphaeroides 2.4.1                                    proteobacteria>alphaproteobacteria            Molybdopterin converting factor subunit 2 [Rhodobacter sphaeroides 2.4.1]
27355756       160     <-OmpA||Excinuclease->phosphatidylglycerophosphate_synthase->MoaD->MoaE*->                                             Bradyrhizobium japonicum USDA 110                                proteobacteria>alphaproteobacteria            molybdopterin converting factor large subunit [Bradyrhizobium japonicum USDA 110]
23347497       163     <-MoaE*<-MoaD<-phosphatidylglycerophosphate_synthase<-Excinuclease<-ADH||OmpA->                                        Brucella suis 1330                                               proteobacteria>alphaproteobacteria            molybdopterin converting factor, subunit 2 [Brucella suis 1330]
39648091       155     <-MoaE*<-MoaD<-phosphatidylglycerophosphate_synthase<-Excinuclease||OmpA->                                             Rhodopseudomonas palustris CGA009                                proteobacteria>alphaproteobacteria            molybdopterin converting factor, subunit 2 [Rhodopseudomonas palustris CGA009]
78494766       152     <-OmpA||Excinuclease->phosphatidylglycerophosphate_synthase->MoaD->MoaE*->                                             Rhodopseudomonas palustris BisB18                                proteobacteria>alphaproteobacteria            Molybdopterin biosynthesis MoaE [Rhodopseudomonas palustris BisB18]
83577061       162     MobA->MoaC->MoaD->MoaE*->                                                                                              Rhodospirillum rubrum ATCC 11170                                 proteobacteria>alphaproteobacteria            Molybdopterin biosynthesis MoaE [Rhodospirillum rubrum ATCC 11170]
85705895       147     <-MoaE*<-MoaD<-phosphatidylglycerophosphate_synthase<-?<-?<-?<-Excinuclease                                            Roseovarius sp. 217                                              proteobacteria>alphaproteobacteria            molybdopterin converting factor, subunit 2 [Roseovarius sp. 217]
84705082       155     <-MoaB<-MoaE*<-MoaD<-moaA                                                                                              Parvularcula bermudensis HTCC2503                                proteobacteria>alphaproteobacteria            molybdopterin converting factor, subunit 2 [Parvularcula bermudensis HTCC2503]
69936171       146     <-MoaE*<-MoaD<-phosphatidylglycerophosphate_synthase                                                                   Paracoccus denitrificans PD1222                                  proteobacteria>alphaproteobacteria            Molybdopterin biosynthesis MoaE [Paracoccus denitrificans PD1222]
69926308       155     <-OmpA||Excinuclease->phosphatidylglycerophosphate_synthase->MoaD->MoaE*->                                             Nitrobacter hamburgensis X14                                     proteobacteria>alphaproteobacteria            Molybdopterin biosynthesis MoaE [Nitrobacter hamburgensis X14]
13421104       150     <-MoaC<-MoaB<-MoaE*<-MoaD<-moaA                                                                                        Caulobacter crescentus CB15;(Note MoaB related to MoeA)          proteobacteria>alphaproteobacteria            molybdopterin converting factor, subunit 2 [Caulobacter crescentus CB15]
84786468       156     moaA->MoaD->MoaE*->                                                                                                    Erythrobacter litoralis HTCC2594                                 proteobacteria>alphaproteobacteria            molybdopterin converting factor, subunit 2 [Erythrobacter litoralis HTCC2594]
15074100       155     <-MoaE*<-MoaD<-phosphatidylglycerophosphate_synthase<-Excinuclease<-ADH||OmpA->                                        Sinorhizobium meliloti                                           proteobacteria>alphaproteobacteria            PROBABLE MOLYBDOPTERIN MPT CONVERTING FACTOR, SUBUNIT 2 PROTEIN [Sinorhizobium meliloti]
68538766       146     <-MoaE*<-MoaD<-phosphatidylglycerophosphate_synthase                                                                   Sphingopyxis alaskensis RB2256                                   proteobacteria>alphaproteobacteria            Molybdopterin biosynthesis MoaE [Sphingopyxis alaskensis RB2256]
68193705       154     <-OmpA||ADH->Excinuclease->phosphatidylglycerophosphate_synthase->MoaD->MoaE*->                                        Mesorhizobium sp. BNC1                                           proteobacteria>alphaproteobacteria            Molybdopterin biosynthesis MoaE [Mesorhizobium sp. BNC1]
14027319       159     <-OmpA||ADH->Excinuclease->phosphatidylglycerophosphate_synthase->MoaD->MoaE*->                                        Mesorhizobium loti MAFF303099                                    proteobacteria>alphaproteobacteria            molybdopterin converting factor, subunit 2 [Mesorhizobium loti MAFF303099]
23016727       158     ADH->Excinuclease->phosphatidylglycerophosphate_synthase->mobB->MoeA->MoaD->MoaE*->                                    Magnetospirillum magnetotacticum MS-1                            proteobacteria>alphaproteobacteria            COG0314: Molybdopterin converting factor, large subunit [Magnetospirillum magnetotacticum MS-1]
83854897       147     <-MoaE*<-MoaD<-phosphatidylglycerophosphate_synthase<-Excinuclease<-ADH                                                Sulfitobacter sp. NAS-14.1                                       proteobacteria>alphaproteobacteria            molybdopterin converting factor, subunit 2 [Sulfitobacter sp. NAS-14.1]
85707988       147     moaA->MoaD->MoaE*->                                                                                                    Erythrobacter sp. NAP1                                           proteobacteria>alphaproteobacteria            molybdopterin converting factor, subunit 2 [Erythrobacter sp. NAP1]
68180109       147     <-MoaE*<-MoaD<-phosphatidylglycerophosphate_synthase<-Excinuclease<-ADH                                                Jannaschia sp. CCS1                                              proteobacteria>alphaproteobacteria            Molybdopterin biosynthesis MoaE [Jannaschia sp. CCS1]
58001332       170     <-MoaE*<-MoaD<-MoaC<-moaA<-MoeA                                                                                        Gluconobacter oxydans 621H                                       proteobacteria>alphaproteobacteria            Molybdopterin (MPT) converting factor, subunit 2 [Gluconobacter oxydans 621H]
15156159       155     <-MoaE*<-MoaD<-phosphatidylglycerophosphate_synthase<-Excinuclease<-ADH||OmpA->                                        Agrobacterium tumefaciens str. C58;                              proteobacteria>alphaproteobacteria            AGR_C_2084p [Agrobacterium tumefaciens str. C58]
32444388       170     MoaD->MoaE*->                                                                                                          Rhodopirellula baltica SH 1                                      planctomycetes                                molybdopterin converting factor, large subunit [Rhodopirellula baltica SH 1]
28271029       133     <-Mopterin_binding_protein<-?<-?||MoaE*->MoaD->moaA->                                                                  Lactobacillus plantarum WCFS1                                    firmicutes                                    molybdopterin biosynthesis protein, E chain [Lactobacillus plantarum WCFS1]
16410446       140     <-permease||?->MoeA->mobB->MoaE*->MoaD->MoaC->moaA-><-MoaB<-MoeB                                                       Listeria monocytogenes                                           firmicutes                                    lmo1044 [Listeria monocytogenes]
56379151       155     moaA-><-?||MoeA->mobB->MoaE*->MoaD->                                                                                   Geobacillus kaustophilus HTA426                                  firmicutes                                    molybdopterin converting factor (subunit 2) [Geobacillus kaustophilus HTA426]
72494466       148     MoaB-><-MoaC||MoeA->mobB->MoaE*->MoaD->MobA->moaA->                                                                    Staphylococcus saprophyticus subsp. saprophyticus ATCC 15305     firmicutes                                    molybdopterin converting factor large subunit [Staphylococcus saprophyticus subsp. saprophyticus ATCC 15305]
29898351       139     <-ADH<-?<-?<-MoaD<-MoaE*<-mobB<-MoeA||MoaC-><-MoeB                                                                     Bacillus cereus ATCC 14579                                       firmicutes                                    Molybdopterin (MPT) converting factor, subunit 2 [Bacillus cereus ATCC 14579]
29895811       156     moaA->MoeB->MoeA->MoaE*->MoaD->                                                                                        Bacillus cereus ATCC 14579                                       firmicutes                                    Molybdopterin (MPT) converting factor, subunit 2 [Bacillus cereus ATCC 14579]
56908909       142     <-MoaB||moaA->MoeA->mobB->MoaE*->MoaD->                                                                                Bacillus clausii KSM-K16                                         firmicutes                                    molybdopterin converting factor subunit 2 MoaE [Bacillus clausii KSM-K16]
10175641       156     MobA-><-MoaD<-MoaE*<-mobB<-MoeA<-MoaB||MoaC->                                                                          Bacillus halodurans C-125                                        firmicutes                                    molybdopterin converting factor (subunit 2) [Bacillus halodurans C-125]
52003240       164     MobA->MoeB->MoeA->mobB->MoaE*->MoaD->                                                                                  Bacillus licheniformis ATCC 14580                                firmicutes                                    molybdopterin converting factor (subunit 2) [Bacillus licheniformis ATCC 14580]
2633801        157     MobA->MoeB->MoeA->mobB->MoaE*->MoaD->?->?->?->?->Mopterin_binding_protein->                                            Bacillus subtilis subsp. subtilis str. 168;                      firmicutes                                    molybdopterin converting factor (subunit 2) [Bacillus subtilis subsp. subtilis str. 168]
75760852       165     <-ADH<-?<-?<-MoaD<-MoaE*<-mobB<-MoeA||MoaC-><-MoeB                                                                     Bacillus thuringiensis serovar israelensis ATCC 35646            firmicutes                                    Molybdopterin converting factor, large subunit [Bacillus thuringiensis serovar israelensis ATCC 35646]
75762420       157     moaA->?->MoeB->MoeA->MoaE*->MoaD->                                                                                     Bacillus thuringiensis serovar israelensis ATCC 35646            firmicutes                                    Molybdopterin converting factor, large subunit [Bacillus thuringiensis serovar israelensis ATCC 35646]
49242615       148     <-moaA<-MobA<-MoaD<-MoaE*<-mobB<-MoeA||MoaC-><-MoaB                                                                    Staphylococcus aureus subsp. aureus MRSA252                      firmicutes                                    putative molybdopterin-synthase large subunit [Staphylococcus aureus subsp. aureus MRSA252]
3955206        150     MoaB-><-MoaC||MoeA->mobB->MoaE*->MoaD->MobA->moaA->                                                                    Staphylococcus carnosus                                          firmicutes                                    MoaE [Staphylococcus carnosus]
57867759       150     <-moaA<-MobA<-MoaD<-MoaE*<-mobB<-MoeA||MoaC-><-MoaB                                                                    Staphylococcus epidermidis RP62A                                 firmicutes                                    molybdenum cofactor biosynthesis protein E [Staphylococcus epidermidis RP62A]
68446506       149     MoaB-><-MoaC||MoeA->mobB->MoaE*->MoaD->MobA->moaA->                                                                    Staphylococcus haemolyticus JCSC1435                             firmicutes                                    molybdopterin converting factor moa [Staphylococcus haemolyticus JCSC1435]
78704014       132     MoaD->MoaE*->                                                                                                          Methanospirillum hungatei JF-1                                   euryarchaeota                                 Molybdopterin biosynthesis MoaE [Methanospirillum hungatei JF-1]
78705135       135     MoeB->MoaD->MoaE*-><-?||?->permease->Mopterin_binding_protein->                                                        Methanospirillum hungatei JF-1                                   euryarchaeota                                 Molybdopterin biosynthesis MoaE [Methanospirillum hungatei JF-1]
86604897       161     <-MoaE*<-MoaD<-?<-moaA<-MoeA                                                                                           Cyanobacteria bacterium Yellowstone A-Prime                      cyanobacteria                                 molybdopterin converting factor, subunit 2 [Cyanobacteria bacterium Yellowstone A-Prime]
35214942       149     MoaD->MoaE*->                                                                                                          Gloeobacter violaceus PCC 7421; MoaD->MoaE                       cyanobacteria                                 molybdopterin converting factor subunit 2 [Gloeobacter violaceus PCC 7421]
1001213        145     Ferr-nitrite_reductase->cyanate_lyase->MoeA->moaA->MoaC+MobA->MoaD->MoaE*->                                            Synechocystis sp. PCC 6803                                       cyanobacteria                                 molybdopterin (MPT) converting factor, subunit 2 [Synechocystis sp. PCC 6803]
33639603       142     MoaC->MoeA-><-?||?-><-MoaE*<-MoaD||MoaB->                                                                              Synechococcus sp. WH 8102                                        cyanobacteria                                 molybdenum cofactor biosynthesis protein E (molydbopterin converting factor large subunit) [Synechococcus sp. WH 8102]
78170140       148     MoaC->MoeA->sugar_epimerase-><-MoaE*<-MoaD||MoaB->                                                                     Synechococcus sp. CC9902                                         cyanobacteria                                 molybdenum cofactor biosynthesis protein E [Synechococcus sp. CC9902]
22295084       148     LysR<-MoaE*<-MoaD<-MoaC+MobA<-moaA<-MoeA                                                                               Thermosynechococcus elongatus BP-1                               cyanobacteria                                 molybdopterin (MPT) converting factor, subunit 2 [Thermosynechococcus elongatus BP-1]
76261575       137     ADH->MoaD->MoaE*->                                                                                                     Chloroflexus aurantiacus J-10-fl                                 chloroflexi                                   Molybdopterin biosynthesis MoaE [Chloroflexus aurantiacus J-10-fl]
86134371       140     <-MoaE*||?-><-MoaD                                                                                                     Tenacibaculum sp. MED152                                         bacteroidetes/chlorobi                        molybdopterin converting factor, subunit 2 [Tenacibaculum sp. MED152]
67937986       130     <-MoaE*<-MoaD<-MoeA<-MoaC+MoeA                                                                                         Chlorobium phaeobacteroides BS1;                                 bacteroidetes/chlorobi                        Molybdopterin biosynthesis MoaE [Chlorobium phaeobacteroides BS1]
86143256       142     <-moaA<-MoaC+MoeA<-MoaE*<-MoeB<-MoaD<-MobA<-ModE<-MoeA                                                                 Flavobacterium sp. MED217;                                       bacteroidetes/chlorobi                        molybdopterin converting factor, subunit 2 [Flavobacterium sp. MED217]
68553533       130     <-MoaE*<-MoaD<-MoeA<-?<-moaA                                                                                           Prosthecochloris aestuarii DSM 271                               bacteroidetes/chlorobi                        Molybdopterin biosynthesis MoaE [Prosthecochloris aestuarii DSM 271]
68562527       146     MoaD->MoaE*->                                                                                                          Rubrobacter xylanophilus DSM 9941; MoaD->MoaE                    actinobacteria                                Molybdopterin biosynthesis MoaE [Rubrobacter xylanophilus DSM 9941]
54017798       145     <-MoaD<-moaA||MoeA->?->MoaE*->                                                                                         Nocardia farcinica IFM 10152                                     actinobacteria                                putative molybdopterin biosynthesis protein [Nocardia farcinica IFM 10152]
13880439       141     MoaC->MoaB->MoaE*-><-?<-MoaD<-moaA                                                                                     Mycobacterium tuberculosis CDC1551                               actinobacteria                                molybdopterin cofactor biosynthesis protein E [Mycobacterium tuberculosis CDC1551]
62425449       140     <-MoaE*<-MoaC<-MoeA||moaA->MoaD-><-MoeB+Rhod<-MoeA                                                                     Brevibacterium linens BL2                                        actinobacteria                                COG0314: Molybdopterin converting factor, large subunit [Brevibacterium linens BL2]
25169125       155     <-MoaD<-MoaD<-MoaD<-moaA||MoeA->MoaC->MoaE*->                                                                          Arthrobacter nicotinovorans                                      actinobacteria                                molybdopterin synthase (large subunit moaE) [Arthrobacter nicotinovorans]
41406902       141     MoaC->MoaB->MoaE*-><-?<-MoaD<-moaA                                                                                     Mycobacterium avium subsp. paratuberculosis K-10                 actinobacteria                                MoaE2 [Mycobacterium avium subsp. paratuberculosis K-10]
12620120       150     moaA->MoaB->MoaC->MoaD->MoaE*->                                                                                        uncultured bacterium pCosHE1                                                                                   AF250774_5 putative molybdopterin converting factor subunit 2 [uncultured bacterium pCosHE1]
40062751       148     <-MoaB<-MoaD<-moaA<-MoaC<-MoeA||MobA-><-MoaE*                                                                          uncultured bacterium 439                                                                                       molydopterin converting factor, subunit 2 [uncultured bacterium 439]

Example of a MoaC fused to a MoaD
^^^^^^^^^^^^^^^^^^^^^^^^^^^^^^^^^
GI             LENGTH    Operon                                                                                                                  ORGANISM                                                        Classification                                Protein descriptions (if any)
84319278       243    PhoH(PIN+ATPase)->MoaC+MoaD->MoaE->MoaB->MoeA                                                                           Pseudomonas aeruginosa C3719                                     proteobacteria>gammaproteobacteria            COG0315: Molybdenum cofactor biosynthesis enzyme [Pseudomonas aeruginosa C3719]
67676070       262    permease->ABC ATPAse->MobA->MobB->MoeA->MoaC+MoaD->MoaE->MoaA->                                                         Chromohalobacter salexigens DSM 3043                             proteobacteria>gammaproteobacteria            Molybdopterin cofactor biosynthesis protein MoaC [Chromohalobacter salexigens DSM 3043]
Bacterial MoaDs that are fused to MoaE (Gis are for the MoaD+MoaE protein- marked with an asterisk)
^^^^^^^^^^^^^^^^^^^^^^^^^^^^^^^^^^^^^^
GI             LENGTH    Operon                                                                                                              ORGANISM                                                         Classification                                Protein descriptions (if any)
67927210       229    MoaD+MoaE*                                                                                                              Solibacter usitatus Ellin6076                                    fibrobacteres/acidobacteria                   Molybdopterin biosynthesis MoaE:ThiamineS [Solibacter usitatus Ellin6076]
46200249       223    MoaD+MoaE*                                                                                                              Thermus thermophilus HB27                                        deinococci                                    molybdopterin (MPT) converting factor, subunit 2 [Thermus thermophilus HB27]
66799395       273    MoaD+MoaE*                                                                                                              Deinococcus geothermalis DSM 11300                               deinococci                                    Molybdopterin converting factor, subunit 1 [Deinococcus geothermalis DSM 11300]
6460436        229    MoaD+MoaE*                                                                                                              Deinococcus radiodurans R1                                       deinococci                                    AE002090_1 molybdenum cofactor biosynthesis protein D/E [Deinococcus radiodurans R1]
51858004       230    MoaD+MoaE*                                                                                                              Symbiobacterium thermophilum IAM 14863                           actinobacteria                                molybdopterin converting factor-like protein [Symbiobacterium thermophilum IAM 14863]
13883249       221    MoaA->dehydratase-> MoaC->MoaD+MoaE*->                                                                                  Mycobacterium tuberculosis CDC1551                               actinobacteria                                (dehydratase-pterin-4-alpha-carbinolamine dehydratase) molybdopterin cofactor biosynthesis protein D/E [Mycobacterium tuberculosis CDC1551]


b.  Archaeal pathways involved in MOCO biosynthesis and related pathways (MoaD gis)
^^^^^^^^^^^^^^^^^^^^^^^^^^^^^^^^^^^^^^^^^^^^^^^^^^^^^^^^^^^
- Molybdenum pathway (Basic construction with minor elaboration)
Gis are for the MoaD containing protein (marked with an asterisk)
GI             LENGTH     Operon                                                                                                              ORGANISM                                                         Classification                                Protein descriptions (if any)
15621527       236        MoaD+MoaE*->ThiD+X->HD->InPP+X->Glucosaminyltransferase->                                                           Sulfolobus tokodaii str. 7                                       crenarchaeota                                 236aa long hypothetical molybdopterin converting factor [Sulfolobus tokodaii str. 7]
68567385       235        MoaD+MoaE*->ThiD+X->HD->InPP+X->Glucosaminyltransferase->                                                           Sulfolobus acidocaldarius DSM 639                                crenarchaeota                                 molybdenum cofactor biosynthesis protein D/E [Sulfolobus acidocaldarius DSM 639]
13815697       231        MoaD+MoaE*->ThiD+X->HD->InPP+X->Glucosaminyltransferase->                                                           Sulfolobus solfataricus P2                                       crenarchaeota                                 Molybdenum cofactor biosynthesis protein E (moaE) [Sulfolobus solfataricus P2]
18159566       229        MoaD+MoaE*                                                                                                          Pyrobaculum aerophilum str. IM2                                  crenarchaeota                                 molybdenum cofactor biosynthesis protein D/E [Pyrobaculum aerophilum str. IM2]
88950646       130        MoaD*->MoaE                                                                                                         Methanosaeta thermophila PT                                      euryarchaeota                                 MoaD, archaeal [Methanosaeta thermophila PT]
88603453       92         MoaD*->MoaE                                                                                                         Methanospirillum hungatei JF-1                                   euryarchaeota                                 thiamineS [Methanospirillum hungatei JF-1]
88601825       91         MoeB->MoaD*->MoaE->                                                                                                 Methanospirillum hungatei JF-1                                   euryarchaeota                                 thiamineS [Methanospirillum hungatei JF-1]
48430776       75         MoaC->MoaB->MoaE-><-Sugar_transporter<-MoaA<-MoaD*                                                                  Picrophilus torridus DSM 9790                                    euryarchaeota                                 molybdopterin (MPT) converting factor, subunit 1 [Picrophilus torridus DSM 9790]
57160377       88         MoaD*->MoeB<-?->MoaE->                                                                                              Thermococcus kodakarensis KOD1                                   euryarchaeota                                 molybdopterin converting factor, subunit 1 [Thermococcus kodakarensis KOD1]
33356787       94         MoeA->MoaD*->                                                                                                       Pyrococcus abyssi GE5                                            euryarchaeota                                 molybdopterin converting factor, subunit 1 [Pyrococcus abyssi GE5]
5458838        89         MoeA->MoaD*->                                                                                                       Pyrococcus abyssi GE5                                            euryarchaeota                                 moaD molybdopterin synthase, small subunit [Pyrococcus abyssi GE5]
33359306       89         MoeA->MoaD*->                                                                                                       Pyrococcus horikoshii OT3                                        euryarchaeota                                 putative molybdopterin converting factor, subunit 1 [Pyrococcus horikoshii OT3]
18892532       90         MoeA->MoaD*->                                                                                                       Pyrococcus furiosus DSM 3638                                     euryarchaeota                                 molybdopterin converting factor, subunit 1                                         ;                                     (moaD) [Pyrococcus furiosus DSM 3638]
10640334       85         MoaD*->?->TFIIB<-MoeA+PBPII<-MoeA                                                                                   Thermoplasma acidophilum                                         euryarchaeota                                 MoaD (involved in molybdopterin synthesis) related protein [Thermoplasma acidophilum]
14324783       90         MoeA->MoeA+PPBII-><-WcaG->MoaD*->                                                                                   Thermoplasma volcanium GSS1                                      euryarchaeota                                 molybdopterin converting factor subunit 1 [Thermoplasma volcanium GSS1]

Archaeal MoaD Solos (Gis are of the MoaD protein-marked with an asterisk)
^^^^^^^^^^^^^^^^^^^^
GI             LENGTH     Operon                                                                                                              ORGANISM                                                         Classification                                Protein descriptions (if any)
11499216       86         MoaD*                                                                                                               Archaeoglobus fulgidus DSM 4304                                  euryarchaeota                                 molybdopterin converting factor, subunit 1 (moaD) [Archaeoglobus fulgidus DSM 4304]
55378770       133        MoaD*-><-MoaD                                                                                                       Haloarcula marismortui ATCC 43049                                euryarchaeota                                 hypothetical protein rrnAC2058 [Haloarcula marismortui ATCC 43049]
55379974       92         MoaD*                                                                                                               Haloarcula marismortui ATCC 43049                                euryarchaeota                                 hypothetical protein rrnAC3439 [Haloarcula marismortui ATCC 43049]
10581293       100        MoaD*-><-MoaD                                                                                                       Halobacterium sp. NRC-1                                          euryarchaeota                                 Vng1848h [Halobacterium sp. NRC-1]
68210071       112        MoaD*->CrcB->CrcB->                                                                                                 Methanococcoides burtonii DSM 6242                               euryarchaeota                                 MoaD, archaeal [Methanococcoides burtonii DSM 6242]
19918186       97         MoaD*->MoeA->CrcB->CrcB->                                                                                           Methanosarcina acetivorans C2A                                   euryarchaeota                                 molybdopterin converting factor, subunit 1 [Methanosarcina acetivorans C2A]
21226933       97         MoaD*->MoeA->CrcB->CrcB->                                                                                           Methanosarcina mazei Go1                                         euryarchaeota                                 Molybdopterin converting factor small subunit [Methanosarcina mazei Go1]
72395205       97         MoaD*->MoeA->CrcB->CrcB->                                                                                           Methanosarcina barkeri str. fusaro                               euryarchaeota                                 molybdopterin converting factor small subunit [Methanosarcina barkeri str. fusaro]
76801893       93         MoaD*                                                                                                               Natronomonas pharaonis DSM 2160                                  euryarchaeota                                 probable molybdopterin converting factor, small subunit 2 [Natronomonas pharaonis DSM 2160]
76803138       92         MoaD*                                                                                                               Natronomonas pharaonis DSM 2160                                  euryarchaeota                                 probable molybdopterin converting factor, small subunit 1 [Natronomonas pharaonis DSM 2160]
76802608       97         MoaD*                                                                                                               Natronomonas pharaonis DSM 2160                                  euryarchaeota                                 homolog to molybdopterin converting factor, small subunit [Natronomonas pharaonis DSM 2160]
18160633       93         MoaD*                                                                                                               Pyrobaculum aerophilum str. IM2                                  crenarchaeota                                 conserved hypothetical protein [Pyrobaculum aerophilum str. IM2]
18160535       90         MoaD*                                                                                                               Pyrobaculum aerophilum str. IM2                                  crenarchaeota                                 conserved hypothetical protein [Pyrobaculum aerophilum str. IM2]
18161603       94         MoaD*                                                                                                               Pyrobaculum aerophilum str. IM2                                  crenarchaeota                                 conserved hypothetical protein [Pyrobaculum aerophilum str. IM2]
33356700       78         MoaD*->CBS->                                                                                                        Pyrococcus abyssi GE5                                            euryarchaeota                                 hypothetical protein PAB1981.1n [Pyrococcus abyssi GE5]
18893753       79         MoaD*->CBS->                                                                                                        Pyrococcus furiosus DSM 3638                                     euryarchaeota                                 hypothetical protein [Pyrococcus furiosus DSM 3638]
33359416       75         MoaD*->CBS->                                                                                                        Pyrococcus horikoshii OT3                                        euryarchaeota                                 hypothetical protein PH1595.1n [Pyrococcus horikoshii OT3]
68567124       84         MoaD*                                                                                                               Sulfolobus acidocaldarius DSM 639                                crenarchaeota                                 conserved Archaeal protein [Sulfolobus acidocaldarius DSM 639]
10640172       90         MoaD*                                                                                                               Thermoplasma acidophilum                                         euryarchaeota                                 conserved hypothetical protein [Thermoplasma acidophilum]
42557747       448        MoaD*                                                                                                               uncultured crenarchaeote                                         crenarchaeota                                 putative molybdopterin biosynthesis protein [uncultured crenarchaeote]

Archaeal operons that have the MoaE protein and do not include the MoaD protein
^^^^^^^^^^^^^^^^^^^^^^^^^^^^^^^^^^^^^^^^^^^^^^^^^^^^^^^^^^^^^^^^^^^^^^^^^^^^^^^^
--- Archaeal MoaEs without MoaD; MobB+MoaE (MobB: Nitrogenase like GTPase); note many of these have a MoaD solo elsewhere in the genome
--gis are of the MoaE protein (marked with an asterisk)
GI             LENGTH     Operon                                                                                                               ORGANISM                                                        Classification                                Protein descriptions (if any)
55377991       275        MoaC->MoaE*<-CysT<-ModA->ThiC->                                                                                      Haloarcula marismortui ATCC 43049                               euryarchaeota                                 molybdenum cofactor biosynthesis protein [Haloarcula marismortui ATCC 43049]
10579734       297        <-MobB+MoaE*<-MoeA-->MoaA<-MoeA+PPBDII                                                                               Halobacterium sp. NRC-1                                         euryarchaeota                                 (Molybd binding domain)-                               molybdenum cofactor biosynthesis protein; MoaE [Halobacterium sp. NRC-1]
68211451       276        <-MobB+MoaE*<-RadC                                                                                                   Methanococcoides burtonii DSM 6242                              euryarchaeota                                 Molybdopterin-guanine dinucleotide biosynthesis protein [Methanococcoides burtonii DSM 6242]
72397307       213        <-MoeA<-MobB+MoaE*                                                                                                   Methanosarcina barkeri str. fusaro                              euryarchaeota                                 molybdopterin converting factor, subunit 2 [Methanosarcina barkeri str. fusaro]
21228894       278        MobB+MoaE*->MobA<-MoeA+PPBDII                                                                                        Methanosarcina mazei Go1                                        euryarchaeota                                 Molybdopterin converting factor, subunit 2 [Methanosarcina mazei Go1]
19915893       279        <-RadC<-MobB+MoaE*->MobA->                                                                                           Methanosarcina acetivorans C2A                                  euryarchaeota                                 molybdopterin-guanine dinucleotide biosynthesis protein B/molybdopterin converting factor, large subunit [Methanosarcina acetivorans C2A]
72396955       285        MobB+MoaE*->MobA->                                                                                                   Methanosarcina barkeri str. fusaro                              euryarchaeota                                 molybdopterin converting factor, subunit 2 [Methanosarcina barkeri str. fusaro]
76801780       262        MobB+MoaE*->?->metalloprotease<-ThiL                                                                                 Natronomonas pharaonis DSM 2160                                 euryarchaeota                                 molybdopterin converting factor, large subunit [Natronomonas pharaonis DSM 2160]
5104258        249        MoaE*                                                                                                                Aeropyrum pernix K1                                             crenarchaeota                                 249aa long hypothetical molybdopterin (mpt) converting factor, subunit 2 [Aeropyrum pernix K1]
11499761       239        <-RecB<-phosphoesterase->MoaE*->                                                                                     Archaeoglobus fulgidus DSM 4304                                 euryarchaeota                                 molybdopterin converting factor, subunit 2 (moaE) [Archaeoglobus fulgidus DSM 4304]
2833554        119        MoaE*                                                                                                                Methanocaldococcus jannaschii                                   euryarchaeota                                 Y717_METJA Hypothetical protein MJ0717
2621190        143        MoaE*->HD hydrolase->Flavoprotein->                                                                                  Methanothermobacter thermautotrophicus str. Delta H             euryarchaeota                                 molybdenum cofactor biosynthesis protein MoaE [Methanothermobacter thermautotrophicus str. Delta H]
5457600        148        TPR<-MoaE*->FeS oxidoreductase->KaiC->                                                                               Pyrococcus abyssi GE5                                           euryarchaeota                                 moaE molybdopterin synthase, large chain [Pyrococcus abyssi GE5]
68139846       136        MoaE*                                                                                                                Ferroplasma acidarmanus Fer1                                    euryarchaeota                                 Molybdopterin biosynthesis MoaE [Ferroplasma acidarmanus Fer1]
45047664       141        MoaE*                                                                                                                Methanococcus maripaludis S2                                    euryarchaeota                                 Molybdopterin biosynthesis MoaE [Methanococcus maripaludis S2]
72397308       60         MoaE*                                                                                                                Methanosarcina barkeri str. fusaro                              euryarchaeota                                 hypothetical protein Mbar_A2676 [Methanosarcina barkeri str. fusaro]
18892013       145        MoaE*                                                                                                                Pyrococcus furiosus DSM 3638                                    euryarchaeota                                 molybdopterin converting factor (subunit 2) [Pyrococcus furiosus DSM 3638]
10640820       135        MoaE*                                                                                                                Thermoplasma acidophilum                                        euryarchaeota                                 molybdopterin-synthase large subunit related protein [Thermoplasma acidophilum]
14324300       137        MoaE*                                                                                                                Thermoplasma volcanium GSS1 a_b_hydrolase->MoaE                 euryarchaeota                                 molybdopterin converting factor subunit 2 [Thermoplasma volcanium GSS1]
52549594       130        MoaE*                                                                                                                uncultured archaeon GZfos28G7                                                                                 molybdopterin converting factor subunit 2 [uncultured archaeon GZfos28G7]
52550228       130        MoaE*                                                                                                                uncultured archaeon GZfos36D8                                                                                 molybdopterin converting factor large subunit [uncultured archaeon GZfos36D8]
52548569       134        MoaE*                                                                                                                uncultured archaeon GZfos17C7                                                                                 molybdopterin converting factor large subunit [uncultured archaeon GZfos17C7]

Miscellaneous pathways
^^^^^^^^^^^^^^^^^^^^^^^^^^^^
GI             LENGTH     Operon                                                                                                               ORGANISM                                                        Classification                                Protein descriptions (if any)
11498162       88        MoaD->MoeB->SirA->?->SirA->                                                                                           Archaeoglobus fulgidus DSM 4304;                                euryarchaeota                                 hypothetical protein AF0552 [Archaeoglobus fulgidus DSM 4304]
68550331   **  317       ModA->ModC->Cys synthase->cystathione gamme synthase->ThiS->ThiG->                                                    Pelodictyon phaeoclathratiforme BU-1                            bacteroidetes/chlorobi                        Cysteine synthase K/M:Cysteine synthase A [Pelodictyon phaeoclathratiforme BU-1]
-----------------------------------------------------------------------------------------------                                                --------------

3. Tungsten cofactor biosynthesis
^^^^^^^^^^^^^^^^^^^^^^^^^^^^^^^^^^^^^^^^
Abbreviations: 4Fe-S:4fe-SFerredoxin; AOR: Aldehyde ferredoxin oxidoreductase,
PDOR : Pyridine disulfide oxidoreductase, ADH: Alcohol dehydrogenase
Always have AOR and MoaD, often MoeB, occasionally MoeA and MoaA, MoaE

a. Archaeal operons (Gis are for the MoaD protein)
^^^^^^^^^^^^^^^^^^^^^^^^^^^^^^^^^^^^^^^^
GI             LENGTH     Operon                                                                                                               ORGANISM                                                        Classification                                Protein descriptions (if any)
5458384        84         AOR->MoaD*->MoaA->                                                                                                   Pyrococcus abyssi GE5                                           euryarchaeota                                 moaD-like molybdopterin converting factor related, subunit 1 [Pyrococcus abyssi GE5]
18892299       82         AOR->MoaD*->MoaA->                                                                                                   Pyrococcus furiosus DSM 3638                                    euryarchaeota                                 molybdopterin converting factor, subunit 1; (moaD) [Pyrococcus furiosus DSM 3638]
11497643       91         AOR->MoaD*->                                                                                                         Archaeoglobus fulgidus DSM 4304                                 euryarchaeota                                 hypothetical protein AF0022 [Archaeoglobus fulgidus DSM 4304]
19915596       94         AOR->MoaD*->                                                                                                         Methanosarcina acetivorans C2A                                  euryarchaeota                                 predicted protein [Methanosarcina acetivorans C2A]
21228746       94         AOR->MoaD*->                                                                                                         Methanosarcina mazei Go1                                        euryarchaeota                                 putative molybdopterin converting factor [Methanosarcina mazei Go1]
736275         69         AOR->MoaD*->MoaA->                                                                                                   Pyrococcus furiosus DSM 3638                                    euryarchaeota                                 unnamed protein product [Pyrococcus furiosus DSM 3638]
33359354       84         AOR->MoaD*-><-?->MoaA->                                                                                              Pyrococcus horikoshii OT3                                       euryarchaeota                                 putative molybdopterin converting factor, subunit 1 [Pyrococcus horikoshii OT3]
57159324       82         AOR->MoaD*->MoaA->                                                                                                   Thermococcus kodakarensis KOD1                                  euryarchaeota                                 molybdopterin converting factor, subunit 1 [Thermococcus kodakarensis KOD1]
14325024       91         <-MoaD*<-AOR                                                                                                         Thermoplasma volcanium GSS1                                     euryarchaeota                                 molybdopterin converting factor subunit 1 [Thermoplasma volcanium GSS1]

Possibly involved in tungsten cofactor biosynthesis (as the MoaAs, typically retrievethe tungsten cofactor protein in blast searches)
^^^^^^^^^^^^^^^^^^^^^^^^^^^^^^^^^^^^^^^^^^^^^^^^^^^^
gis are of the MoaD protein (marked with an asterisk)

GI             LENGTH     Operon                                                                                                                ORGANISM                                                       Classification                                Protein descriptions (if any)
11499688       89         MoaA->MoaD*->                                                                                                         Archaeoglobus fulgidus DSM 4304                                euryarchaeota                                 hypothetical protein AF2105 [Archaeoglobus fulgidus DSM 4304]
68140833       78         MoaA->MoaD*->                                                                                                         Ferroplasma acidarmanus Fer1                                   euryarchaeota                                 MoaD, archaeal [Ferroplasma acidarmanus Fer1]
76801420       145        MoaA->?->MoaA->MoaD*->                                                                                                Natronomonas pharaonis DSM 2160                                euryarchaeota                                 pterin cluster protein [Natronomonas pharaonis DSM 2160]

b. Bacterial operons (gis are for the AOR gene- marked with an asterisk)
^^^^^^^^^^^^^^^^^^^^^^^^^^^^^^^^^^^^^^^^^^^^^^^
GI             LENGTH     Operon                                                                                                                ORGANISM                                                       Classification                                Protein descriptions (if any)
78700229       616        4Fe-S->AOR*->PDOR->MoaD->                                                                                             Alkalilimnicola ehrlichei MLHE-1                               proteobacteria>gammaproteobacteria            aldehyde:ferredoxin oxidoreductase,tungsten-containing [Alkalilimnicola ehrlichei MLHE-1]
34482541       575        AOR*->MoaD->MoeB                                                                                                      Wolinella succinogenes                                         proteobacteria>epsilonproteobacteria          ALDEHYDE OXIDOREDUCTASE [Wolinella succinogenes]
68178064       576        Dehyd->AOR*->MoaD->MoeB->FeS_assembly?->MoaA                                                                          Desulfuromonas acetoxidans DSM 684                             proteobacteria>deltaproteobacteria            IMP dehydrogenase/GMP reductase:Aldehyde ferredoxin oxidoreductase [Desulfuromonas acetoxidans DSM 684]
77543953       576        MoeA<-dehyd->AOR*->MoaD->MoeB->dehyd                                                                                  Pelobacter carbinolicus DSM 2380                               proteobacteria>deltaproteobacteria            aldehyde ferredoxin oxidoreductase [Pelobacter carbinolicus DSM 2380]
71838535       576        MoeA<-dehyd->AOR*->MoaD->MoeB->permease->ABC ATPase                                                                   Pelobacter propionicus DSM 2379                                proteobacteria>deltaproteobacteria            Aldehyde ferredoxin oxidoreductase [Pelobacter propionicus DSM 2379]
77544154       577        AOR*->MoaD->                                                                                                          Pelobacter carbinolicus DSM 2380                               proteobacteria>deltaproteobacteria            aldehyde ferredoxin oxidoreductase [Pelobacter carbinolicus DSM 2380]
71544346       609        AOR*->MoaD->                                                                                                          Syntrophobacter fumaroxidans MPOB                              proteobacteria>deltaproteobacteria            Aldehyde ferredoxin oxidoreductase [Syntrophobacter fumaroxidans MPOB]
46449005       576        AOR*-><-MoaD                                                                                                          Desulfovibrio vulgaris subsp. vulgaris str. Hildenborough      proteobacteria>deltaproteobacteria            aldehyde:ferredoxin oxidoreductase, tungsten-containing [Desulfovibrio vulgaris subsp. vulgaris str. Hildenborough]
78193518       576        dehyd->AOR*->MoaD->MoeB                                                                                               Geobacter metallireducens GS-15                                proteobacteria>deltaproteobacteria            Aldehyde ferredoxin oxidoreductase [Geobacter metallireducens GS-15]
78219908       577        MoaD->MoeB<-AOR*                                                                                                      Desulfovibrio desulfuricans G20                                proteobacteria>deltaproteobacteria            aldehyde:ferredoxin oxidoreductase, tungsten-containing [Desulfovibrio desulfuricans G20]
68178220       576        AOR*->MoaD->MoeB                                                                                                      Desulfuromonas acetoxidans DSM 684                             proteobacteria>deltaproteobacteria            IMP dehydrogenase/GMP reductase:Aldehyde ferredoxin oxidoreductase [Desulfuromonas acetoxidans DSM 684]
50877365       575        MoeA->MoeA+PPBPII->AOR*->MoaD                                                                                         Desulfotalea psychrophila LSv54                                proteobacteria>deltaproteobacteria            related to tungsten-containing aldehyde ferredoxin oxidoreductase (AOR) [Desulfotalea psychrophila LSv54]
39982778   **  601        4Fe-S->AOR*->PDOR->MoaD-> MoeB->                                                                                      Geobacter sulfurreducens PCA                                   proteobacteria>deltaproteobacteria            aldehyde:ferredoxin oxidoreductase, tungsten-containing [Geobacter sulfurreducens PCA]
74023041       617        4Fe-S->AOR*->PDOR->MoaD->                                                                                             Rhodoferax ferrireducens DSM 15236                             proteobacteria>betaproteobacteria             Aldehyde ferredoxin oxidoreductase [Rhodoferax ferrireducens DSM 15236]
84716937       615        4Fe-S->AOR*->PDOR->MoaD->                                                                                             Polaromonas naphthalenivorans CJ2                              proteobacteria>betaproteobacteria             aldehyde:ferredoxin oxidoreductase,tungsten-containing [Polaromonas naphthalenivorans CJ2]
47572159       592        4Fe-S->AOR*->PDOR->MoaD->                                                                                             Rubrivivax gelatinosus PM1                                     proteobacteria>betaproteobacteria             COG2414: Aldehyde:ferredoxin oxidoreductase [Rubrivivax gelatinosus PM1]
56314521   **  774        AOR*+MoaD                                                                                                             Azoarcus sp. EbN1                                              proteobacteria>betaproteobacteria             putative tungsten-containing aldehyde ferredoxin oxidoreductase (AOR-1)
23015426       616        4Fe-S->AOR*->PDOR->PDOR->MoaD->                                                                                       Magnetospirillum magnetotacticum MS-1                          proteobacteria>alphaproteobacteria            COG2414: Aldehyde:ferredoxin oxidoreductase [Magnetospirillum magnetotacticum MS-1]
83589574       599        4Fe-S->AOR*->MoaD->PDOR->                                                                                             Moorella thermoacetica ATCC 39073                              firmicutes                                    Aldehyde ferredoxin oxidoreductase [Moorella thermoacetica ATCC 39073]
77996039       597        AOR*->MoaA->MoaD->MoaE->                                                                                              Carboxydothermus hydrogenoformans Z-2901                       firmicutes                                    aldehyde ferredoxin oxidoreductase, tungsten-containing [Carboxydothermus hydrogenoformans Z-2901]
76795288   **  599        AOR*->MoaA->MoaD->MoeB->MobB->PPBPII->permease->ABC ATPase->                                                          Thermoanaerobacter ethanolicus ATCC 33223                      firmicutes                                    Aldehyde ferredoxin oxidoreductase [Thermoanaerobacter ethanolicus ATCC 33223]
77995801       629        4Fe-S->AOR*->MoaD->                                                                                                   Carboxydothermus hydrogenoformans Z-2901                       firmicutes                                    aldehyde ferredoxin oxidoreductase, tungsten-containing [Carboxydothermus hydrogenoformans Z-2901]
77995423       597        ADH->AOR*->MoaD->                                                                                                     Carboxydothermus hydrogenoformans Z-2901                       firmicutes                                    aldehyde ferredoxin oxidoreductase, tungsten-containing [Carboxydothermus hydrogenoformans Z-2901]
71540750       597        MoaD->MoeB<-?->4Fe-S->AOR*->                                                                                          Syntrophomonas wolfei str. Goettingen                          firmicutes                                    Aldehyde ferredoxin oxidoreductase [Syntrophomonas wolfei str. Goettingen]
46200136       608        AOR*->MoaD->                                                                                                          Thermus thermophilus HB27                                      deinococci                                    tungsten-containing aldehyde ferredoxin oxidoreductase [Thermus thermophilus HB27]
51858106       604        AOR*->MoaD->                                                                                                          Symbiobacterium thermophilum IAM 14863                         actinobacteria                                aldehyde ferredoxin oxidoreductase [Symbiobacterium thermophilum IAM 14863]
51857711       603        AOR*->MoaD->                                                                                                          Symbiobacterium thermophilum IAM 14863                         actinobacteria                                aldehyde ferredoxin oxidoreductase [Symbiobacterium thermophilum IAM 14863]
-----------------------------------------------------------------------------------------------                                                 --------------
4. Uncharacterized operons with ThiS/ThiF+Rhodanese containing proteins (sulfur metabolism)
^^^^^^^^^^^^^^^^^^^^^^^^^^^^^^^^^^^^^^^^^^^^^^^^^^^^^^^^^^^^^^^^^^^^^^^^^^^^^^^^^^^^^^^^^^^
4a. Siderophore biosynthesis (Gis are of the E1+Rhodanese- marked with an asterisk)
GI             LENGTH     Operon                                                                                                                ORGANISM                                                       Classification                                Protein descriptions (if any)
28192388      387         Hist_phosphate_NH2transferase->E1+Rhodanese*->JAB->ThiS/MoaD->Trp-dioxygenase->hydroxybenzoate hydroxylase->          Pseudomonas fluorescens                                        proteobacteria>gammaproteobacteria            QbsC [Pseudomonas fluorescens]
83645618      390         E1+Rhodanese->JAB*->ThiS/MoaD->+CaiB-like coA transferase->AMP-acid ligase->                                          Hahella chejuensis KCTC 2396                                   proteobacteria>gammaproteobacteria            Dinucleotide-utilizing enzyme involved in molybdopterin and thiamine biosynthesis family 2 [Hahella chejuensis KCTC 2396]
82702101      390         E1+Rhodanese->JAB*->ThiS/MoaD->+CaiB-like coA transferase->                                                           Nitrosospira multiformis ATCC 25196                            proteobacteria>betaproteobacteria             UBA/THIF-type NAD/FAD binding fold [Nitrosospira multiformis ATCC 25196]
30181075      390         E1+Rhodanese->JAB*->ThiS/MoaD->+CaiB-like coA transferase->AMP-acid ligase->                                          Nitrosomonas europaea ATCC 19718                               proteobacteria>betaproteobacteria             Dinucleotide-utilizing enzymes involved in molybdopterin and thiamine biosynthesis family 2 [Nitrosomonas europaea ATCC 19718]
83748714      389         E1+Rhodanese->JAB*->ThiS/MoaD->+CaiB-like coA transferase->AMP-acid ligase->                                          Ralstonia solanacearum UW551                                   proteobacteria>betaproteobacteria             Molybdopterin biosynthesis MoeB protein [Ralstonia solanacearum UW551]
83748714      389         E1+Rhodanese->JAB*->ThiS/MoaD->                                                                                       Ralstonia solanacearum UW551                                   proteobacteria>betaproteobacteria             Molybdopterin biosynthesis MoeB protein [Ralstonia solanacearum UW551]
5070639       391         E1+Rhodanese->JAB*->ThiS/MoaD->+CaiB-like coA transferase->AMP-acid ligase->                                          Pseudomonas stutzeri KC                                        proteobacteria>gammaproteobacteria            AF149851_6 MoeB-like protein [Pseudomonas stutzeri KC]
84994030      390         E1+Rhodanese(PdtF)*->JAB(PdtG)->ThiS/MoaD(PdtH)->+CaiB-like coA transferase(PdtI)->AMP-acid ligase(PdtJ)->            Pseudomonas putida                                             proteobacteria>gammaproteobacteria            PdtF [Pseudomonas putida]
----------------------------------------------------
4b. Uncharacterized operon encoding a ThiS/MoaD, a JAB peptidase and E1-like enzyme (gis are of E1+Rhod- marked with an asterisk)
   ^^^^^^^^^^^^^^^^^^^^^^^^^^^^^^^^^^^^^^^^^^^^^^^^^^^^^^^^^^^^^^^^^^^^^^^^^^^^^^^^^^^^^^^^^^^^^^^^^
GI             LENGTH     Operon                                                                                                                ORGANISM                                                       Classification                                Protein descriptions (if any)
88807869      389         JAB->E1+Rhod*->                                                                                                       Synechococcus sp. WH 7805                                      cyanobacteria                                 gll3412 [Gloeobacter violaceus PCC 7421]
86607093      387         JAB->ThiS/MoaD->E1+Rhod*->                                                                                            Cyanobacteria bacterium Yellowstone A-Prime                    cyanobacteria                                 UBA/THIF-type NAD/FAD binding, MoeZ/MoeB fmaily protein [Anaeromyxobacter dehalogenans 2CP-C]
86609523      389         JAB->ThiS/MoaD->E1+Rhod*->                                                                                            Cyanobacteria bacterium Yellowstone B-Prime                    cyanobacteria                                 putative molybdopterin biosynthesis protein MoeB [Synechococcus sp. JA-2-3B'a(2-13)]
81298969      391         JAB->E1+Rhod*->                                                                                                       Synechococcus elongatus PCC 7942                               cyanobacteria                                 putative molybdopterin biosynthesis protein MoeB [Synechococcus sp. JA-3-3Ab]
87300927      390         JAB->E1+Rhod*->                                                                                                       Synechococcus sp. WH 5701                                      cyanobacteria                                 Rhodanese-like [Alkalilimnicola ehrlichei MLHE-1]
35213984      395         JAB->ThiS/MoaD->E1+Rhod*->                                                                                            Gloeobacter violaceus PCC 7421                                 cyanobacteria                                 Rhodanese-like [Synechococcus elongatus PCC 7942]
78700359      142         JAB->ThiS/MoaD+Rhodanese+E1*->                                                                                        Alkalilimnicola ehrlichei MLHE-1                               proteobacteria>gammaproteobacteria            molybdopterin biosynthesis MoeB protein [Synechococcus sp. WH 5701]
86159911      390         ThiS/MoaD->E1+Rhod*->JAB->                                                                                            Anaeromyxobacter dehalogenans 2CP-C                            proteobacteria>deltaproteobacteria            molybdopterin biosynthesis protein [Synechococcus sp. WH 7805]

-  JABs in operons with E1+Rhod  (No Ub_like) (Gis of E1 containing protein-marked with an asterisk)
   ^^^^^^^^^^^^^^^^^^^^^^^^^^^^^^^^^^^
GI            LENGTH      Operon                                                                                                                ORGANISM                                                       Classification                                Protein descriptions (if any)
75700942      390         JAB->E1+Rhod*->                                                                                                       Anabaena variabilis ATCC 29413                                 cyanobacteria                                 Rhodanese-like MoeZ/MoeB [Anabaena variabilis ATCC 29413]
17132000      390         JAB->E1+Rhod*->                                                                                                       Nostoc sp. PCC 7120                                            cyanobacteria                                 molybdopterin biosynthesis protein [Nostoc sp. PCC 7120]
56686316      391         JAB->E1+Rhod*->                                                                                                       Synechococcus elongatus PCC 6301                               cyanobacteria                                 molybdopterin biosynthesis MoeB protein [Synechococcus elongatus PCC 6301]
71676726      391         JAB->E1+Rhod*->                                                                                                       Trichodesmium erythraeum IMS101                                cyanobacteria                                 UBA/THIF-type NAD/FAD binding fold:Rhodanese-like:MoeZ/MoeB [Trichodesmium erythraeum IMS101]
23124399      390         JAB->E1+Rhod*->                                                                                                       Nostoc punctiforme PCC 73102                                   cyanobacteria                                 COG0476: Dinucleotide-utilizing enzymes involved in molybdopterin and thiamine biosynthesis family 2 [Nostoc punctiforme PCC 73102]
87124948      389         JAB->E1+Rhod*->                                                                                                       Synechococcus sp. RS9917                                       cyanobacteria                                 Rhodanese-like [Synechococcus sp. RS9917]
78169800      388         JAB->E1+Rhod*->                                                                                                       Synechococcus sp. CC9902                                       cyanobacteria                                 Rhodanese-like [Synechococcus sp. CC9902]
72002829      381         JAB->E1+Rhod*->                                                                                                       Prochlorococcus marinus str. NATL2A                            cyanobacteria                                 rhodanese-like [Prochlorococcus marinus str. NATL2A]
33238703      379         JAB->E1+Rhod*->                                                                                                       Prochlorococcus marinus subsp. marinus str. CCMP1375           cyanobacteria                                 Prochlorococcus marinus subsp. marinus str. CCMP1375 complete genome
33635570      409         JAB->E1+Rhod*->                                                                                                       Prochlorococcus marinus str. MIT 9313                          cyanobacteria                                 molybdopterin biosynthesis protein [Prochlorococcus marinus str. MIT 9313]
84513874      379         JAB->E1+Rhod*->                                                                                                       Prochlorococcus marinus str. MIT 9211                          cyanobacteria                                 Dinucleotide-utilizing enzyme [Prochlorococcus marinus str. MIT 9211]
78196401      378         JAB->E1+Rhod*->                                                                                                       Synechococcus sp. CC9605                                       cyanobacteria                                 Rhodanese-like [Synechococcus sp. CC9605]
33633363      377         JAB->E1+Rhod*->                                                                                                       Synechococcus sp. WH 8102                                      cyanobacteria                                 molybdopterin biosynthesis protein [Synechococcus sp. WH 8102]
76882207      257         E1*->JAB->                                                                                                            Nitrosococcus oceani ATCC 19707                                proteobacteria>gammaproteobacteria            Adenylyltransferase [Nitrosococcus oceani ATCC 19707]
----------------------------------------------------
4c. Uncharacterized operon with a  ThiS/MoaD, E1-like enzyme, a JAB and a Cysteine synthase  (gis are of the cys synthase- marked with an asterisk)
^^^^^^^^^^^^^^^^^^^^^^^^^^^^^^^^^^^^^^^^^^^^^^^^^^^^^^^^^^^^^^^^^^^^^^^^^^^^^^^^^^^^^^^^^^^
GI            LENGTH      Operon                                                                                                                ORGANISM                                                       Classification                                Protein descriptions (if any)
68563152       305        Cys synthase*->JAB->ThiS/MoaD->E1+Rhodanese->                                                                         Rubrobacter xylanophilus DSM 9941                              actinobacteria                                Cysteine synthase K/M [Rubrobacter xylanophilus DSM 9941]
83815753       317        Cys syn*->JAB->ThiS/MoaD->E1+Rhodanese->                                                                              Salinibacter ruber DSM 13855                                   bacteroidetes/chlorobi                        cysteine synthase B [Salinibacter ruber DSM 13855]
83757147       317        Cys syn*->JAB->ThiS/MoaD->E1+Rhod->                                                                                   Salinibacter ruber DSM 13855                                   bacteroidetes/chlorobi                         cysteine synthase B [Salinibacter ruber DSM 13855]
76258730       308        Cys synthase*->JAB->ThiS/MoaD->E1+Rhodanese->                                                                         Chloroflexus aurantiacus J-10-fl                               chloroflexi                                   Cysteine synthase K/M [Chloroflexus aurantiacus J-10-fl]
67932284       319        Cys syn*->JAB->ThiS/MoaD->E1+Rhodanese->                                                                              Solibacter usitatus Ellin6076                                  fibrobacteres/acidobacteria                   Cysteine synthase K/M [Solibacter usitatus Ellin6076]
78493973       304        JAB->E1+Rhodanese->Cys synthase*->                                                                                    Rhodopseudomonas palustris BisB18                              proteobacteria>alphaproteobacteria             Pyridoxal-5'-phosphate-dependent enzyme, beta subunit [Rhodopseudomonas palustris BisB18]
9948117        392        Cys synthase*->E1+Rhodanese->                                                                                         Pseudomonasaeruginosa PAO1;                                    proteobacteria>gammaproteobacteria            AE004638_1 probable molybdopterin biosynthesis protein MoeB [Pseudomonas aeruginosa PAO1]
----------------------------------------------------
4d. Uncharacterized operon with a ThiS/MoaD/MoaD, JAB, Cysteine synthase and ClpS  (gis of Cys synthases)
^^^^^^^^^^^^^^^^^^^^^^^^^^^^^^^^^^^^^^^^^^^^^^^^^^^^^^^^^^^^^^^^^^^^^^^^^^^^^^^^^^^^^^^^^^^^^^^^^^^^^^^^^
GI            LENGTH      Operon                                                                                                                ORGANISM                                                       Classification                                Protein descriptions (if any)
13880986       323       ClpS->alpha_helical_domain->JAB->ThiS/MoaD->Cys synthase*->                                                            Mycobacterium tuberculosis CDC1551;                            actinobacteria                                cysteine synthase [Mycobacterium tuberculosis CDC1551]
54014566       320       ClpS->alpha_helical_domain->dmpA_peptidase->JAB->ThiS/MoaD->Cys synthase*->                                            Nocardia farcinica IFM 10152;                                  actinobacteria                                putative cysteine synthase [Nocardia farcinica IFM 10152]
29608823       316       ClpS->alpha_helical_domain->permease->JAB->ThiS/MoaD->Cys synthase*->                                                  Streptomyces avermitilis; MA-4680                              actinobacteria                                putative cysteine synthase [Streptomyces avermitilis MA-4680]
68231907       315       ClpS->alpha_helical_domain->JAB->ThiS/MoaD->Cys synthase*->                                                            Frankia sp. EAN1pec;                                           actinobacteria                                Cysteine synthase K/M [Frankia sp. EAN1pec]
86739581       315       ClpS->alpha_helical_domain->JAB->ThiS/MoaD->Cys synthase*->                                                            Frankia sp. CcI3;                                              actinobacteria                                cysteine synthases [Frankia sp. CcI3]
71916499       315       ClpS->alpha_helical_domain->JAB->ThiS/MoaD->Cys synthase*->                                                            Thermobifida fusca YX;                                         actinobacteria                                cysteine synthase K/M [Thermobifida fusca YX]
71366891       320       alpha_helical_domain->MutT->JAB->ThiS/MoaD->Cys synthase*->                                                            Nocardioides sp. JS614;                                        actinobacteria                                Cysteine synthase K/M [Nocardioides sp. JS614]
5531359        316       JAB->alpha_helical_domain->ThiS/MoaD->Cys Syn*->alpha_helical_domain<-MBL                                              Streptomyces coelicolor A3(2);                                 actinobacteria                                putative cysteine synthase [Streptomyces coelicolor A3(2)]


Alignment of rapidly diverging alpha helical protein

ALIGN                              -------------EE--HHHHHHHHHHHHHHHHHHH-------------------HHHHH-------HH------------------------------EEE-----------------------HHHHHHHHH--HHHHHHHHHHHHHHHHH---------------------HH-HEEE--HHHHHHHHHHHHHHHHHHHHHHH-------------------------HHHHHHHHHHHHHHHHHH---
HMM                                -----------HEEEEHHHHHHHHHHHHHHHHHHHHH---------H-------HHHHHH-------HE----------------------------EEEEEE---------------------HHHHHHHHHH-HHHHHHHHHHHHHHHHH----------------------EEEEEEE--HHHHHHHHHHHHHHHHHHHH-EEE----HHHH-----H-------HHHHHHHHHHHHHHHHHHHHHH--
FREQ                               ---HHH------HEE---HHHHHHHHHHHHHHHHHHH------------------HHHHH-------H------------------------------H-H------------------------HHHHHHHHHHHHHHHHHHHHHHHHHHH----------------H-HHH----HHEHHHHHHHHHHHHHHHHHHHHHHHHH-------------------------HHHHHHHHHHHHHHHHHHHH--
PSSM                               ------------EEEEEHHHHHHHHHHHHHHHHHHH--------------------HHHH-------H------------------------------EEEE-----------------------HHHHHHHH----HHHHHHHHHHHHHHHH------------------------EEEEE-HHHHHHHHHHH---EEEEEEE-----------------------HHHHHHHHHHHHHHHHHHHHHH---
FINAL                              ------------EEEE-HHHHHHHHHHHHHHHHHHH-------------------HHHHH-------H------------------------------EEEE----------------------HHHHHHHHHHHHHHHHHHHHHHHHHHHHH------------------------EEEEE-HHHHHHHHHHHHHHHHHHHHH------------------------HHHHHHHHHHHHHHHHHHHHHH--
NocaDRAFT_2640_Nsp._71366887       MSGFQRHRRSKLIIANFTGFEADLLRSLAGQLVELLRNEAAVPRDPV-------DPFEAM-------MDF------------------SGPTQEPEDPVLARLFPTAYPGD-------------QEAASEFRRFTEGTLRDGKAAAAVAIIDGL--------EEAGLPPELTEDGLMIDIELDEATAETWMRSFTDLRLALATRLEVEEGDDAYW-----HSLPDDDPRAQAHDIYEWVGYLQETLVQALSG
Lxx13320_Lxyl_50951464             MRPFRRTRDGT-LRARFEPDEAEILARLAAETAELAV-----------------DAA-------------------------------SGAGDPREDPAFIRLLPDAYSGD-------------AEASAEFRRFTAGGLAERKALTAQVVMETL--------GGGSG---------AIEVRLDAPQAAAWLRTLTDIRLVLAARLGIVQDGDEG-------DIHDAD-SAFRRAVYDWLAGVQESLVLALRS
BlinB01002436_Blin_62424056        --MAAIDARGDDVVLKLEDNERSLMLTVFTDLAALLAEDDNEDGRPD------SENWEARLG--------------------------LVERPRPQDPALLRLFPDVDPLDE-------------ERSREFRRLTEFDLQQAKAHNVRIVLNGL---------AKGS-----------SITLNHDEVLAWMKGLNDLRLVLAVRMGIDTEEAQEEKYAQREDL--DESEELTLTLYDFLTWIQDRLTTTLLS
clpS_Jsp._84494379                 AFARKGKGKNLRYAAKLDAVERAVVAGLMEQVHDLVAPEPEEAVATGPSGASDHDDDFAAIVSGLGGLGMGVSISAEDQVADDRPVPADARSFGDRDPALERLLPAGNRAD-------------DQVSAEFRRLTEHGLRQRKAGHLESAITSL--------RAPGS-----------GVELDERAAIDMVIALTDVRLVLGERLGLREDADVDRLEEELADVDDDDPRGHAMSVYDFLTWLQETLATAMLP
cg2770_Cglu_41326695               WKKKKGLMRQARYAVVFEPMEREVLGDLSAAVSEALIQRAQS--VPK-------DPLAEMTGMT------------------------SGHKEAPTDPALARLLPDFQHEGD---------EEYDGDNSFLRSLHEGDITRAKLENLRVINDAL--------GPDGN----------VAVTASEEEAHAWLAALNDIRLYVASG-DVRGGEAAE---------------EDRENLVQWLAYNQESLLEAMMN
_Ceff_23494252                     WKRRKALMRSARYTCVLEPMEREVLGNLSAVVLEALIHRAQD--APK-------DPLAELTGIP------------------------SGHKEAPRDPALARLLPDFQQEGD---------EEYDGDNSLLRSLHENDITRQKIANLQVINSAL--------GPDGG----------VAVSIPEEEAHAWLAGLNDIRLYLASG-ELKGGEAAE---------------EDRENLVQWLAYNQESLLEAMMG
DIP1856_Cdip_38200689              WKKKKGLFKGARYQCTLEPIEREVLGNLAANISEVLISRAQS--APK-------DELAELTGMG------------------------GGHTEAPEDPGLARLLPDFEMQGD---------EEFDGDNSLLRSLHENDITRAKLANLQTIGQAL--------GPDGS----------VFVTVTEEEAQAWVAGLNDIRLYLASSE-VQDTEDRD-------------------ALVEWLAFAQESLLTAMMG
jk0494_Cjei_68263163               WTKKNSLLRGTRFNTQLEPLEREMLGDSAVAVSDKLMERART--APK-------DELAEMTGMA------------------------SGHADAPKDPGLARLLPSFFREGD---------EEVDGDAALTRQLNETDIIKTKLSNLRFVVDYL--------GPNGS----------VNVSLTQDEVHPWLSAINDIRLYHSAQYEEFKKELL-------EGEENSDQATAAQNYLDWLGYHQDSLLSAMMG
nfa10870_Nfar_54014562             KWTRKNSLGGLKLRAEMDAHEAEVLRSLVGAVSGLLAERAQS--APE-------DELSALTGLR------------------------TGNTAPPDDPRLARLLPDFHRSEPGSPDADRA-----GLNSALRALHEPEIIDAKLAAGSVVLDTV--------PARGG-----------KIVLTPEQADAWLSALTDVRLALGTVLGIDAETP--------DQLDPDDPRAPHLDVYHWLTWMQDSLLQALAP
SCO2915_Scoe_5531364               MPGQFEPLPGGGAAVALDDVEISIIRSLAVQLLELIGPGPAED-ASD-------DPLAELFA--------------------------EGPSEPPSDPVLRRLFPDAYGDPEGAPQAREA-EEQRAHSAEFRRYTENDLRAGKRDNALAVVRTLDTLSSASAGEEGA-----------VLKLSPQESQQWLRALNDLRLAIGSRLEIADEDDTDLLYR----LPDEDPRKPMVMAYLWLGGLQESLVATLMP
SAV5160_Save_29608819              MPGHFEPLPGGGAAVALDEVEISIIRSLAVQLLELIGPGPAED-AAA-------DPLAELFA--------------------------EGPSEPPSDPVLQRLFPDAYGGPGGEGGSPEEAEEQRAHSSEFRRFTENDLRAGKRENALVVIRTL--DGMTVAGEGGA-----------VLKLSPEESRQWLGSLNDLRLAIGSRLDVVDEEDTDLLYR----LPDEDPRKPMVMAYLWLGGLQETLIETLMS
Francci3_0865_Fsp._86739578        DVADGFRRTRAGIELRLPRLEAALLIELVGQIESLLEPPP-----VE-------DPLEALVGLR------------------------DTAPPPPDDPAIARLLPDPYPDD-------------PMASGDFRRRRTDDLLARKRDAARRVLSAV--------PAPGR-----------ALLLDEEAAQDWLTTLNDLRLVLGTRLGLTDDDSTAEL----EHLDPDDSRRPLVAVYAFLTELLDDLTRALG-
Franean1DRAFT_3648_Fsp._68231910   --MNGFRRTRAGIELRLPRLESSLLTELLGQVDALLEAPP-----VD-------DPLEALVGLR------------------------DTAPPPPEDPAVARLLPDPYPDD-------------PLASGDFRRRRTDEALARKRDAARRVLAAV--------PAPGA-----------VLVLDEDAAQDWLTVLNDLRLVLGTRLGLTDDESTAELEN----LTPEDPRRPVAAVYAFLTELLDELTRALL-
KradDRAFT_2533_Krad_67987809       -MATFRRTRNGHFSLTLHAAEADLLASLAREVLELLEVPAAAPPRPV-------DPLQAELGLS----------DLPGFDTPLDDLAGDGPVAPPEDEVLRRLLPDAYGDD-------------PDASADFRRFTERGLRERKAAAASGLLAGL-----APVEGQGG-----------RVQLDADGARTWLAALNDIRLALGTRLGVSEDADPD------ADLAEDDPARWAWAVYDFTTHLQETLVRSLS-
Tfu_2371_Tfus_71916502             MTAKIRSAPHGGARITIGPDEAQLLRSMADFLLRVVEEPEQ-----Q-------DELAALVGIS-------------------------SSATQPEDPALARLFPDAYTDD-------------AEAAADFRRYTESDLRRHKRENARRVASAI--------PEWGG-----------EIVLDAEDVQAWLQTLTDVRLYLGVRLGIETEEDADAL---RAAAVRDESLAAAMHVYEWFTYVQDSLVRAVWQ
ArthDRAFT_1846_Asp._66965396       -MAKAFKYGIKGITGYLEPAERELLRSLIDDVISMLQPAES---ASE-------DPLTALIGLD-------------------------MNVREPSDRALRRLLPNVTKDD-------------DAASLEFRQLTERSLRENKIGALRAAALGL----------DTN-----------ELVLSQADARHWSQALNDVRLVLAERLDIRDDADAEHVHTMQDWSQAEDVESYLALVYNFTTWLQESLVQAMLQ
MT1374_Mtub_13880982               WKRVET-RDGPRFRSSLAPHEAALLKNLAGAMIGLLDDRDSS--SPS-------DELEEITGIK------------------------TGHAQRPGDPTLRRLLPDFYRPDDLDDDDPTAVDGSESFNAALRSLHEPEIIDAKRVAAQQLLDTV--------PDNGG-----------RLELTESDANAWIAAVNDLRLALGVMLEIGPRGP--------ERLPGNHPLAAHFNVYQWLTVLQEYLVLVLMG
MtubF_01001398_Mtub_76784817       WKRVET-RDGPRFRSSLAPHEAALLKNLAGAMIGLLDDRDSS--SPS-------DELEEITGIK------------------------TGHAQRPGDPTLRRLLPDFYRPDDLDDDDPTAVDGSESFNAALRSLHEPEIIDAKRVAAQQLLDTV--------PDNGG-----------RLELTESDANAWIAAVNDLRLALGVMLEIGPRGP--------ERLPGNHPLAAHFNVYQWLTVLQEYLVLVLMG
MAP2428c_Mavi_41408526             WKRVET-AEGPRFRSALASHEAALLKNLATAMIGLLDERESS--SPA-------DELEEITGIK------------------------TGNAQPPKDPTLRRLLPDFYRPDDNGDESPDAAE---SLNAALRSLHEPGIVNAKRVAAQRLLGTV--------PDDGG-----------RFELTEDDANAWIAAVNDIRLTLGVMLEIGPDGP--------ERLPADHPLAVHFDVYQWLTVLQEYLVLVLMG
_Mlep_466922                       WKRVET-ANGPRFRSVVAPHEVALLKHLVGALLGLLNERESS--SPL-------DELEVITGIK------------------------AGNAQRPEDPTLRRLLPDFYTPDDKDQLDPAALDAVDSLNAALRSLHEPEIVDAKRSAAQQLLDTL--------PESDG-----------RLELTEASANAWIAAVNDLRLALGVILEIDRPAP--------ERVPAGHPLSVHFDVYQWLTVLQEYLVLALMA
ML1166_Mlep_13093139               WKRVET-ANGPRFRSVVAPHEVALLKHLVGALLGLLNERESS--SPL-------DELEVITGIK------------------------AGNAQRPEDPTLRRLLPDFYTPDDKDQLDPAALDAVDSLNAALRSLHEPEIVDAKRSAAQQLLDTL--------PESDG-----------RLELTEASANAWIAAVNDLRLALGVILEIDRPAP--------ERVPAGHPLSVHFDVYQWLTVLQEYLVLALMA
consensus/100%                     ............h...h...E..hh..........h..................-.........................................D..h.RLhPs.....................s...R.bp...h...K......h...l...........s.............h.hs...s..h..shsDlRL..u................................hh.a.s..b-.L..sh..
consensus/90%                      ............h...h...E..ll.plhs.h..hl.........s........D.h..b.s...........................s....PpDPsl.RLhPs....s................su.hRphpp..l...K..sh..l..sl...........ss............l.ls...s..Wh.slsDlRLhlus.b.l....s......................hh.ahs..Q-.L..sh..

Species abbreviations: Asp. : Arthrobacter sp.; Blin : Brevibacterium linens; Cdip : Corynebacterium diphtheriae; Ceff : Corynebacterium efficiens; Cglu : Corynebacterium glutamicum; Cjei : Corynebacterium jeikeium; Fsp. : Frankia sp.; Jsp. : Janibacter sp.; Krad : Kineococcus radiotolerans; Lxyl : Leifsonia xyli; Mavi : Mycobacterium avium; Mlep : Mycobacterium leprae; Mtub : Mycobacterium tuberculosis; Nfar : Nocardia farcinica; Nsp. : Nocardioides sp.; Save : Streptomyces avermitilis; Scoe : Streptomyces coelicolor; Tfus : Thermobifida fusca


Miscellaneous operons
Rhodanese+E1 (no JABs in operons- gis are of the Rhodanese+E1 protein)
^^^^^^^^^^^^^^^^^^^^^^^^^^^^^^^^^^^^
GI            LENGTH      Operon                                                                                                                ORGANISM                                                       Classification                                Protein descriptions (if any)
71898141      379        Rhodanese+E1                                                                                                           Xylella fastidiosa Ann-1                                       proteobacteria>gammaproteobacteria            UBA/THIF-type NAD/FAD binding fold:Rhodanese-like:MoeZ/MoeB [Xylella fastidiosa Ann-1]
71900908      386        Rhodanese+E1                                                                                                           Xylella fastidiosa Ann-1                                       proteobacteria>gammaproteobacteria            UBA/THIF-type NAD/FAD binding fold:MoeZ/MoeB [Xylella fastidiosa Ann-1]
9105314       379        Rhodanese+E1                                                                                                           Xylella fastidiosa 9a5c                                        proteobacteria>gammaproteobacteria            AE003897_1 molybdopterin biosynthesis protein [Xylella fastidiosa 9a5c]
77747707      379        Rhodanese+E1                                                                                                           Xylella fastidiosa Temecula1                                   proteobacteria>gammaproteobacteria            molybdopterin biosynthesis protein MoeB [Xylella fastidiosa Temecula1]
78036060      401        MoeA-><-Rhodanese+E1                                                                                                   Xanthomonas campestris pv. vesicatoria str. 85-10;             proteobacteria>gammaproteobacteria            molybdopterin biosynthesis protein MoeB [Xanthomonas campestris pv. vesicatoria str. 85-10]
58426731      472        Rhodanese+E1                                                                                                           Xanthomonas oryzae pv. oryzae KACC10331                        proteobacteria>gammaproteobacteria            molybdopterin biosynthesis protein [Xanthomonas oryzae pv. oryzae KACC10331]
21108248      380        Rhodanese+E1                                                                                                           Xanthomonas axonopodis pv. citri str. 306                      proteobacteria>gammaproteobacteria            molybdopterin biosynthesis protein [Xanthomonas axonopodis pv. citri str. 306]
84367975      379        Rhodanese+E1                                                                                                           Xanthomonas oryzae pv. oryzae MAFF 311018                      proteobacteria>gammaproteobacteria            molybdopterin biosynthesis protein [Xanthomonas oryzae pv. oryzae MAFF 311018]
21113107      378        Rhodanese+E1                                                                                                           Xanthomonas campestris pv. campestris str. ATCC 33913          proteobacteria>gammaproteobacteria            molybdopterin biosynthesis protein [Xanthomonas campestris pv. campestris str. ATCC 33913]
----------------------------------------------------
4e. Operons with genes for sulfur metabolism proteins
^^^^^^^^^^^^^^^^^^^^^^^^^^^^^^^^^^^^^^^^^^^^^^^^^^^^^
Domain abbreviations:
SirA-like redox proteins IF3C-fold, regulator of disulfide bond formation?,  (note in some instances this protein is fused to a Rhodanese)
OAHShyd: typically O-acetylhomoserine/serine sulfhydrylase/Methionine lyase; PLP dependent transferase superfamily
DsrE/H: ancient family, Conserved cysteine, often fused and solo versions, also in archaea,
involved in sulfur reduction, YchN-like fold, perhaps a breakaway Rossmannoid, DsrH like proteins
are involved in oxidation of intracellular sulfur (pdb: 1l1s ): solo gi:67938822
PAPSR: Phosphoadenosine phosphosulfate reductase
ATP_sulf: ATP sulfurylase

Gis are of the SirA or MoaD/ThiS  protein -marked with an asterisk

GI            LENGTH      Operon                                                                                                                                                              ORGANISM                                                       Classification                                Protein descriptions (if any)
67938823      82          ThiS/MoaD->OAHShyd->E1 solo->JAB->DsrE/H->SirA*->                                                                                                                   Chlorobium phaeobacteroides BS1;                               bacteroidetes/chlorobi                        SirA-like [Chlorobium phaeobacteroides BS1]
68208690      80          PAPSR->ATP_sulf->Sulf_adenyltransf_large->ThiS/MoaD->E1->JAB->Sulf_reductase(Fe-S binding protein)->SirA*->                                                         Desulfitobacterium hafniense DCB-2;                            firmicutes                                    SirA-like [Desulfitobacterium hafniense DCB-2]
77996033      72          sulfite_reductase->E1->ThiS/MoaD*->Sulf_adenylyltransferase->4Fe-S->Adenylylsulfate_reductase->?->Adenylylsulfate_kinase                                            Carboxydothermus hydrogenoformans Z-2901;                      firmicutes                                    thiamine biosynthesis protein ThiS [Carboxydothermus hydrogenoformans Z-2901]
67873788      81          PAPSR->ATP_sulf->Sulf_adenyltransf_large->ThiS/MoaD->E1->JAB->Sulf_reductase(Fe-S binding protein)->SirA*->                                                         Clostridium thermocellum ATCC 27405;                           firmicutes                                    SirA-like [Clostridium thermocellum ATCC 27405]
29894496      77          SirA+Rhodanese->Hydroxyacylglutathione hydrolase->SirA*->Rhod->Rhod->                                                                                               Bacillus cereus ATCC 14579;                                    firmicutes                                    Molybdopterin biosynthesis MoeB protein [Bacillus cereus ATCC 14579]
82499134      82          ABC sulfate transporter->ThiS/MoaD->E1->JAB->sulf_reductaseFe-S binding protein)->SirA*->OAHShyd->Adenylylsulfreduct->Ferredoxin->ATP_sulf->PAPSR->                 Caldicellulosiruptor saccharolyticus DSM 8903;                 firmicutes                                    conserved hypothetical protein [Caldicellulosiruptor saccharolyticus DSM 8903]
78194036      74          OAHShyd->ThiS/MoaD->E1solo->JAB->Sulf_reductase(Fe-S binding protein)->SirA*->                                                                                      Geobacter metallireducens GS-15;                               proteobacteria>deltaproteobacteria            conserved hypothetical protein [Geobacter metallireducens GS-15]
18160982      88          <-PAPSR<-?<-Sulfite_reductase<-?->ThiS/MoaD*->Rhod+Rhod->                                                                                                           Pyrobaculum aerophilum str. IM2;                               crenarchaeota                                 conserved hypothetical protein [Pyrobaculum aerophilum str. IM2]

Operons lacking sirA (gis are of the ThiF/E1-like protein-marked with an asterisk)
GI            LENGTH      Operon                                                                                                                ORGANISM                                                       Classification                                Protein descriptions (if any)
34483109      272         PAPSR->ATP_sulf->Sulf_adenyltransf_large->ThiS/MoaD->E1*->JAB->Sulf_reductase(Fe-S binding protein)->                 Wolinella succinogenes;                                        proteobacteria>epsilonproteobacteria          MOLYBDOPTERIN BIOSYNTHESIS PROTEIN MOEB [Wolinella succinogenes]
77686500      269         CysTRNAsyn_deacylase->ThiS/MoaD->E1*->JAB->Sulf_reductase(Fe-S binding protein)->                                     Alkaliphilus metalliredigenes QYMF                             firmicutes                                    UBA/THIF-type NAD/FAD binding fold:MoeZ/MoeB [Alkaliphilus metalliredigenes QYMF]

ThiS/MoaD+Sulf_reductase containing operon subtype  (Gis of ThiS/MoaD)
GI            LENGTH      Operon                                                                                                                ORGANISM                                                       Classification                                Protein descriptions (if any)
71366157      648         Sulf_reductase+ThiS/MoaD*->PAPSR->                                                                                    Nocardioides sp. JS614                                         actinobacteria                                Ferredoxin--nitrite reductase [Nocardioides sp. JS614]
88931571      639         Sulf_reductase+ThiS/MoaD*->PAPSR->                                                                                    Acidothermus cellulolyticus 11B                                actinobacteria                                Ferredoxin--nitrite reductase [Acidothermus cellulolyticus 11B]
-------------------------------------------------------------------------------------------------------------

5. Phage Tail assembly associated Ub
    ^^^^^^^^^^^^^^^^^^^^^^^^^^^^^^^^^^^^^
gp: Phage containing Ub domain; also called I-tail component; gpK-JAB
J: host specificity protein J; STF : lambda side tail fiber protein

- Operons of the type JAB+NlpC->Ub->gpJ (Gis are of the JAB protein- Marked with an asterisk)

GI            LENGTH      Operon                                                                                                                ORGANISM                                                       Classification                                Protein descriptions (if any)
38707909        194       JAB+NlpC*->Ub->gpJ->                                                                                                  Bacteriophage phi1026b                                         bacteriophages                                gp19 [Bacteriophage phi1026b]
76556246        226       gpM->gpL->JAB+NlpC*->Ub->gpJ->                                                                                        Phage BP-4795                                                  bacteriophages                                putative tail component [Phage BP-4795]
71834086        191       gpM->gpL->Ub->gpJ->                                                                                                   Bacteriophage JK06                                             bacteriophages                                hypothetical tail assembly protein I [Bacteriophage JK06]
77864688        187       gpL->JAB+NlpC*->Ub->gpJ->                                                                                             Burkholderia cepacia phage Bcep176                             bacteriophages                                gp63 [Burkholderia cepacia phage Bcep176]
80750693        190       gpL->HNH->JAB+NlpC*->Ub->gpJ->                                                                                        Bacteriophage RTP                                              bacteriophages                                putative tail assembly protein [Bacteriophage RTP]
46402106        197       gpL->JAB+NlpC*->?->Ub->gpJ+X->                                                                                        Bacteriophage phiKO2                                           bacteriophages                                Gp20 [Bacteriophage phiKO2]
17975181        194       gpL->JAB+NlpC*->Ub->gpJ->                                                                                             Bacteriophage phiE125                                          bacteriophages                                putative tail component protein [Bacteriophage phiE125]
11877308        240       gpL->JAB+NlpC*->Ub->gpJ->                                                                                             Neisseria meningitidis phage 2120                              bacteriophages                                putative protein I [Neisseria meningitidis phage 2120]
9630484         192       gpM->gpL->JAB+NlpC*->Ub->gpJ->                                                                                        Enterobacteria phage N15                                       bacteriophages                                gp20 [Bacteriophage N15]
215124          223       gpM->gpL->JAB+NlpC*->Ub->gpJ->                                                                                        Enterobacteria phage lambda                                    bacteriophages                                I (tail component;223) [bacteriophage lambda]
51773733        180       gpM->gpL->JAB+NlpC*->Ub->gpJ->                                                                                        Bacteriophage CP-1639                                          bacteriophages                                putative tail fiber component I [Bacteriophage CP-1639]
9634139         202       gpL->JAB+NlpC*->?->Ub->?->?<-?->gpJ->                                                                                 Enterobacteria phage HK022                                     bacteriophages                                gp21 [Enterobacteria phage HK022]
45686326        199       gpM->gpL->JAB+NlpC*->Ub->gpJ->                                                                                        Enterobacteria phage T1                                        bacteriophages                                putative tail assembly protein [Enterobacteria phage T1]
84357775        150       gpM->gpL->JAB+NlpC*->Ub solo->gpJ->                                                                                   Burkholderia cenocepacia PC184                                 proteobacteria>betaproteobacteria             COG4723: Phage-related protein, tail component [Burkholderia cenocepacia PC184]
83717443        194       gpL->JAB+NlpC*->Ub->gpJ->                                                                                             Burkholderia thailandensis E264                                proteobacteria>betaproteobacteria             Bacteriophage lambda tail assembly protein I [Burkholderia thailandensis E264]
76579036        188       gpL->JAB+NlpC*->Ub->gpJ->lysozyme->                                                                                   Burkholderia pseudomallei 1710b                                proteobacteria>betaproteobacteria             Bacteriophage lambda tail assembly protein I [Burkholderia pseudomallei 1710b]
16419562        234       JAB+NlpC*->Ub->gpJ->STF->                                                                                             Salmonella typhimurium LT2                                     proteobacteria>gammaproteobacteria            Gifsy-2 prophage probable tail assembly protein [phage Gifsy-2]
83587164        190       gpM->gpL->JAB+NlpC*->Ub->gpJ(N)->gpJ(C)->                                                                             Escherichia coli 101-1                                         proteobacteria>gammaproteobacteria            COG4723: Phage-related protein, tail component [Escherichia coli 101-1]
75208766        180       gpM->gpL->JAB+NlpC*->Ub->gpJ->                                                                                        Escherichia coli B171                                          proteobacteria>gammaproteobacteria            COG4723: Phage-related protein, tail component [Escherichia coli B171]
75210818        182       gpM->gpL->JAB+NlpC*->Ub->gpJ->                                                                                        Escherichia coli B171                                          proteobacteria>gammaproteobacteria            COG4723: Phage-related protein, tail component [Escherichia coli B171]
75208867        144       JAB+NlpC*->Ub->gpJ->                                                                                                  Escherichia coli B171                                          proteobacteria>gammaproteobacteria            COG4723: Phage-related protein, tail component [Escherichia coli B171]
75211970        190       gpM->gpL->JAB+NlpC*->Ub->gpJ(N)->gpJ(C)->                                                                             Escherichia coli B171                                          proteobacteria>gammaproteobacteria            COG4723: Phage-related protein, tail component [Escherichia coli B171]
75229909        190       gpL->JAB+NlpC*->Ub->gpJ(N)->gpJ(C)->                                                                                  Escherichia coli B7A                                           proteobacteria>gammaproteobacteria            COG4723: Phage-related protein, tail component [Escherichia coli B7A]
26107858        210       gpL->JAB+NlpC*->Ub->gpJ(N)->gpJ(C)->                                                                                  Escherichia coli CFT073                                        proteobacteria>gammaproteobacteria            AE016759_331 Putative tail component of prophage [Escherichia coli CFT073]
26107735        204       gpL-><-?->JAB+NlpC*->Ub->gpJ->                                                                                        Escherichia coli CFT073                                        proteobacteria>gammaproteobacteria            AE016759_208 Putative tail assembly protein of cryptic prophage [Escherichia coli CFT073]
26109404        210       gpL<-?->JAB+NlpC*->Ub->gpJ->                                                                                          Escherichia coli CFT073                                        proteobacteria>gammaproteobacteria            AE016765_9 Putative tail component of prophage [Escherichia coli CFT073]
75239817        180       JAB+NlpC*->Ub->gpJ->                                                                                                  Escherichia coli E110019                                       proteobacteria>gammaproteobacteria            COG4723: Phage-related protein, tail component [Escherichia coli E110019]
75235846        193       gpM->gpL->JAB+NlpC*->Ub->gpJ->                                                                                        Escherichia coli E110019                                       proteobacteria>gammaproteobacteria            COG4723: Phage-related protein, tail component [Escherichia coli E110019]
16421139        215       gpM->gpL->JAB+NlpC*->Ub->gpJ->                                                                                        Salmonella typhimurium LT2                                     proteobacteria>gammaproteobacteria            Gifsy-1 prophage protein [Salmonella typhimurium LT2]
75255450        193       gpM->gpL->JAB+NlpC*->Ub->gpJ->                                                                                        Escherichia coli E22                                           proteobacteria>gammaproteobacteria            COG4723: Phage-related protein, tail component [Escherichia coli E22]
75255278        193       gpM->gpL->JAB+NlpC*->Ub->gpJ->                                                                                        Escherichia coli E22                                           proteobacteria>gammaproteobacteria            COG4723: Phage-related protein, tail component [Escherichia coli E22]
24374467        209       NlpC(fragment)->?->Ub->gpJ->                                                                                          Shewanella oneidensis MR-1                                     proteobacteria>gammaproteobacteria            prophage LambdaSo, tail assembly protein I [Shewanella oneidensis MR-1]
75258709        130       gpM->gpL->JAB+NlpC*->Ub->gpJ->                                                                                        Escherichia coli E22                                           proteobacteria>gammaproteobacteria            COG4723: Phage-related protein, tail component [Escherichia coli E22]
75257430        180       Bro-NJAB+NlpC*->Ub->gpJ(N->gpJ(middle)->gpJ(C)->                                                                      Escherichia coli E22                                           proteobacteria>gammaproteobacteria            COG4723: Phage-related protein, tail component [Escherichia coli E22]
75259495        182       gpL->JAB+NlpC*->Ub->gpJ->gpM->                                                                                        Escherichia coli E22                                           proteobacteria>gammaproteobacteria            COG4723: Phage-related protein, tail component [Escherichia coli E22]
75175531        193       gpM->gpL->JAB+NlpC*->Ub->gpJ->                                                                                        Shigella boydii BS512                                          proteobacteria>gammaproteobacteria            COG4723: Phage-related protein, tail component [Shigella boydii BS512]
75239568        182       gpM->gpL->JAB+NlpC*->Ub->gpJ->                                                                                        Escherichia coli F11                                           proteobacteria>gammaproteobacteria            COG4723: Phage-related protein, tail component [Escherichia coli F11]
75239670        190       gpM->gpL->JAB+NlpC*->Ub->gpJ(N)->                                                                                     Escherichia coli F11                                           proteobacteria>gammaproteobacteria            COG4723: Phage-related protein, tail component [Escherichia coli F11]
12514222        300       gpL->JAB+NlpC*->JAB+Ub->gpJ(N)->gpJ(Fn3+C)->                                                                          Escherichia coli O157:H7 EDL933                                proteobacteria>gammaproteobacteria            AE005290_12 putative tail component encoded by cryptic prophage CP-933M; partial [Escherichia coli O157:H7 EDL933]
12515098        225       gpM->gpL->JAB+NlpC*->Ub solo->gpJ->gpM->                                                                              Escherichia coli O157:H7 EDL933                                proteobacteria>gammaproteobacteria            AE005349_14 putative tail component of prophage CP-933O [Escherichia coli O157:H7 EDL933]
12516097        178       gpM->gpL->JAB+NlpC*->Ub solo->gpJ->gpM->                                                                              Escherichia coli O157:H7 EDL933                                proteobacteria>gammaproteobacteria            AE005420_1 putative tail fiber component I of prophage CP-933U [Escherichia coli O157:H7 EDL933]
46143649        181       gpM->gpL->JAB+NlpC*->Ub solo->gpJ->                                                                                   Actinobacillus pleuropneumoniae serovar 1 str. 4074            proteobacteria>gammaproteobacteria            COG4723: Phage-related protein, tail component [Actinobacillus pleuropneumoniae serovar 1 str. 4074]
82543715        204       gpL->JAB+NlpC*->Ub->gpJ->                                                                                             Shigella boydii Sb227                                          proteobacteria>gammaproteobacteria            putative tail component [Shigella boydii Sb227]
32043835        200       gpM->gpL->JAB+NlpC*->Ub solo->gpJ->                                                                                   Pseudomonas aeruginosa UCBPP-PA14                              proteobacteria>gammaproteobacteria            COG4723: Phage-related protein, tail component [Pseudomonas aeruginosa UCBPP-PA14]
75259293        193       gpM->gpL->JAB+NlpC*->Ub solo->gpJ->                                                                                   Escherichia coli E22                                           proteobacteria>gammaproteobacteria            COG4723: Phage-related protein, tail component [Escherichia coli E22]
75234649        193       gpM->gpL->JAB+NlpC*->Ub solo->gpJ->                                                                                   Escherichia coli E110019                                       proteobacteria>gammaproteobacteria            COG4723: Phage-related protein, tail component [Escherichia coli E110019]
75176997        172       gpM->gpL->JAB+NlpC*->Ub solo->gpJ->                                                                                   Shigella boydii BS512                                          proteobacteria>gammaproteobacteria            COG4723: Phage-related protein, tail component [Shigella boydii BS512]
75238944        190       gpM->gpL->JAB+NlpC*->Ub solo->gpJ->                                                                                   Escherichia coli E110019                                       proteobacteria>gammaproteobacteria            COG4723: Phage-related protein, tail component [Escherichia coli E110019]
9946516         200       gpM->gpL->JAB+NlpC*->Ub solo->gpJ->                                                                                   Pseudomonas aeruginosa PAO1                                    proteobacteria>gammaproteobacteria            AE004499_8 probable bacteriophage protein [Pseudomonas aeruginosa PAO1]
75820383        200       gpM->gpL->JAB+NlpC*->Ub solo->gpJ->                                                                                   Vibrio cholerae V51                                            proteobacteria>gammaproteobacteria            COG4723: Phage-related protein, tail component [Vibrio cholerae V51]
74312870        210       gpM->gpL->JAB+NlpC*->Ub->gpJ->                                                                                        Shigella sonnei Ss046                                          proteobacteria>gammaproteobacteria            putative tail component of prophage [Shigella sonnei Ss046]
13361702        226       gpM->gpL->JAB+NlpC*->Ub->gpJ(N)->gpJ(C)->                                                                             Escherichia coli O157:H7                                       proteobacteria>gammaproteobacteria            putative tail assembly protein [Escherichia coli O157:H7 str. Sakai]
13361111        223       gpM->gpL->JAB+NlpC*->Ub->gpJ->                                                                                        Escherichia coli O157:H7                                       proteobacteria>gammaproteobacteria            tail assembly protein [Escherichia coli O157:H7 str. Sakai]
13362414        225       gpM->gpL->JAB+NlpC*->Ub->gpJ(N)->gpJ(C)->                                                                             Escherichia coli O157:H7                                       proteobacteria>gammaproteobacteria            putative tail assembly protein [Escherichia coli O157:H7 str. Sakai]
13360300        215       gpM->gpL->JAB+NlpC*->Ub->gpJ->                                                                                        Escherichia coli O157:H7                                       proteobacteria>gammaproteobacteria            putative tail assembly protein [Escherichia coli O157:H7 str. Sakai]
74312266        180       gpM->gpL->JAB+NlpC*->Ub->gpJ->                                                                                        Shigella sonnei Ss046                                          proteobacteria>gammaproteobacteria            putative tail component of prophage [Shigella sonnei Ss046]
84318835        200       gpM->gpL->JAB+NlpC*->Ub->gpJ(N)->                                                                                     Pseudomonas aeruginosa C3719                                   proteobacteria>gammaproteobacteria            COG4723: Phage-related protein, tail component [Pseudomonas aeruginosa C3719]
56383531        180       gpM->gpL->JAB+NlpC*->Ub->gpJ->gpM->                                                                                   Shigella flexneri 2a str. 301                                  proteobacteria>gammaproteobacteria            putative tail component [Shigella flexneri 2a str. 301]
68345404        188 **    Bro-N->KilA-N+C->Ub->gpJ->P5->                                                                                        Pseudomonas fluorescens Pf-5                                   proteobacteria>gammaproteobacteria            prophage LambdaSo, tail assembly protein I [Pseudomonas fluorescens Pf-5]
24050968        191       gpM->gpL->JAB+NlpC*->Ub->gpJ->gpM->                                                                                   Shigella flexneri 2a str. 301                                  proteobacteria>gammaproteobacteria            putative tail component [Shigella flexneri 2a str. 301]
71037999        187       gpL->JAB+NlpC*->Ub->gpJ->                                                                                             Psychrobacter arcticus 273-4                                   proteobacteria>gammaproteobacteria            probable phage protein tail protein [Psychrobacter arcticus 273-4]
52788057        195       gpM->gpL->JAB+NlpC*->Ub->STF (distinct tail fiber protein)->                                                          Yersinia pestis                                                proteobacteria>gammaproteobacteria            phage lambda tail assembly protein I [Yersinia pestis]
75254904        226       Ub->gpJ(N->)                                                                                                          EscherichiacoliE22                                             proteobacteria>gammaproteobacteria            COG4723: Phage-related protein, tail component [Escherichia coli E22]
2996351         183       gpM->gpL->JAB+NlpC*->Ub->host_specificity_J->                                                                         Yersinia pestis KIM                                            proteobacteria>gammaproteobacteria            unknown [Yersinia pestis KIM]
66046010        192       JAB+NlpC*<-?->Ub<-?->gpJ->                                                                                            Pseudomonas syringae pv. syringae B728a                        proteobacteria>gammaproteobacteria            Bacteriophage lambda tail assembly I [Pseudomonas syringae pv. syringae B728a]
16506034        195       gpM->gpL->JAB+NlpC*->Ub->gpJ->                                                                                        Salmonella enterica subsp. enterica serovar Typhi str. CT18    proteobacteria>gammaproteobacteria            putative phage tail protein [Salmonella enterica subsp. enterica serovar Typhi str. CT18]
62179803        168       gpM->gpL->JAB+NlpC*->Ub->gpJ->                                                                                        Salmonella enterica subsp. enterica serovar Choleraesuis str.  proteobacteria>gammaproteobacteria            Gifsy-1 prophage VtiI [Salmonella enterica subsp. enterica serovar Choleraesuis str. SC-B67]
16419434        225       JAB+NlpC*->gpM->gpL->Ub-><-superoxide_dismutase->host_specificity_J->                                                 Salmonella typhimurium LT2                                     proteobacteria>gammaproteobacteria            putative Fels-1 prophage tail assembly protein [phage Fels-1]
75208698        193       gpM->gpL->JAB+NlpC*->Ub-><-superoxide_dismutase->host_specificity_J->                                                 Escherichia coli B171                                          proteobacteria>gammaproteobacteria            COG4723: Phage-related protein, tail component [Escherichia coli B171]
75235151        193       gpM->gpL->JAB+NlpC*->Ub-><-superoxide_dismutase->host_specificity_J->                                                 Escherichia coli E110019                                       proteobacteria>gammaproteobacteria            COG4723: Phage-related protein, tail component [Escherichia coli E110019]
75214996        193       gpM->gpL->JAB+NlpC*->Ub-><-superoxide_dismutase->host_specificity_J->                                                 Escherichia coli E110019                                       proteobacteria>gammaproteobacteria            COG4723: Phage-related protein, tail component [Escherichia coli E110019]
75233804        193       JAB+NlpC*->gpM->gpL->JAB+NlpC*->Ub-><-superoxide_dismutase->host_specificity_J->                                      Escherichia coli E110019                                       proteobacteria>gammaproteobacteria            COG4723: Phage-related protein, tail component [Escherichia coli E110019]
13361452        226       gpM->gpL->JAB+NlpC*->Ub->?<-Superoxide_dismutase->host_specificity_J->                                                Escherichia coli O157:H7                                       proteobacteria>gammaproteobacteria            putative tail assembly protein [Escherichia coli O157:H7 str. Sakai]
13360578        226       gpM->gpL->JAB+NlpC*->Ub->?<-Superoxide_dismutase->host_specificity_J->                                                Escherichia coli O157:H7                                       proteobacteria>gammaproteobacteria            putative tail assembly protein [Escherichia coli O157:H7 str. Sakai]
84327632        60        gpH->gpL->JAB+NlpC*->Ub solo->gpJ->Lysozyme->                                                                         Pseudomonas aeruginosa 2192 fragment                           proteobacteria>gammaproteobacteria            COG4723: Phage-related protein, tail component [Pseudomonas aeruginosa 2192]

Operons with no gpJ in vicinity (Ub gis)
Gis are of the JAB or NlpC protein- Marked with an asterisk

GI              LENGTH    Operon                                                                                                                ORGANISM                                                       Classification                                Protein descriptions (if any)
13361023        94        gpM->gpL->JAB+NlpC*->Ub->YjbI->?->                                                                                    Escherichia coli O157:H7                                       proteobacteria>gammaproteobacteria            putative tail assembly protein [Escherichia coli O157:H7 str. Sakai]
82533244        194       gpL->JAB+NlpC*->Ub solo (perhaps incomplete assembly)->                                                               Burkholderia pseudomallei 1106b                                proteobacteria>betaproteobacteria             hypothetical protein Bpse110_02005448 [Burkholderia pseudomallei 1106b]
62179570        221       gpL->JAB+NlpC*->Ub->?->lambda p27(distinct tail fiber)->                                                              Salmonella enterica subsp. enterica serovar Choleraesuis str.  proteobacteria>gammaproteobacteria            Gifsy-2 prophage probable tail assembly protein [Salmonella enterica subsp. enterica serovar Choleraesuis str. SC-B67]
75207839        180       NlpC*(fragmented?)->Ub->                                                                                              Escherichia coli B171                                          proteobacteria>gammaproteobacteria            COG4723: Phage-related protein, tail component [Escherichia coli B171]
17428712        200       gpL->JAB+NlpC*->Ub->                                                                                                  Ralstonia solanacearum                                         proteobacteria>betaproteobacteria             probable phage hk022 gp20-related protein [Ralstonia solanacearum]

Operons  without JABs in the operon
^^^^^^^^^^^^^^^^^^^^^^^^^^^^^^^^^^^^^^^^^^
gis are of the Ub protein-marked with an asterisk
GI              LENGTH    Operon                                                                                                                ORGANISM                                                       Classification                                Protein descriptions (if any)
82741527        216       BroN->Ub->gpJ->                                                                                                       Shewanella sp. W3-18-1;                                        proteobacteria>gammaproteobacteria            prophage LambdaSo, tail assembly protein I [Shewanella sp. W3-18-1]
82743846        238       Ub solo                                                                                                               Shewanella sp. W3-18-1;                                        proteobacteria>gammaproteobacteria            prophage LambdaSo, tail assembly protein I [Shewanella sp. W3-18-1]
15980136        206       Bro-N->ribbon->?->Ub->?->host_specificty_J->                                                                          Yersinia pestis CO92;                                          proteobacteria>gammaproteobacteria            putative phage tail assembly protein [Yersinia pestis CO92]
71558268        152       HTH->Ub->gpJ(N)->                                                                                                     Pseudomonas syringae pv. phaseolicola 1448A;                   proteobacteria>gammaproteobacteria            prophage PSPPH03, putative tail assembly protein I [Pseudomonas syringae pv. phaseolicola 1448A]
84780140        198       HNH->Ub->gpJ->lysozyme->                                                                                              Sodalis glossinidius str. 'morsitans';                         proteobacteria>gammaproteobacteria            putative phage tail assembly protein [Sodalis glossinidius str. 'morsitans']

B. Note the Domain_Z protein.. (Domain Z: an all beta domain)
(Gis are of the Ub+gpJ protein- marked with an asterisk)
GI              LENGTH    Operon                                                                                                                ORGANISM                                                       Classification                                Protein descriptions (if any)
31788497        1574      Domain_Z->NlpC->Ub+gpJ*(N+FN3+C)  [1-173 Ubl+ 173-673 (N)]->                                                          Xanthomonas campestris phage Xp10                                                                            22R [Xanthomonas oryzae bacteriophage Xp10]
84570663        1571      Domain_Z->NlpC->Ub+gpJ*(N+FN3+C)->                                                                                    Xanthomonas oryzae phage OP1                                                                                 putative tail component protein [Xanthomonas oryzae phage OP1]
23013869        775       NlpC solo->Ub+gpJ*(N)->P5->?Y-> (Note no JAB)                                                                         Magnetospirillum magnetotacticum MS-1                          proteobacteria>alphaproteobacteria            COG4733: Phage-related protein, tail component [Magnetospirillum magnetotacticum MS-1]
85716602        1267      NlpC->Ub+gpJ*(N+FN3+distinct_C)-> (Note no JAB)*********                                                              Nitrobacter sp. Nb-311A                                        proteobacteria>alphaproteobacteria            tail fiber protein, putative [Nitrobacter sp. Nb-311A]
23016384        508       Domain_Z->NlpC solo->Ub+gpJ*(N)->gpJ(fragment of C)->                                                                 Magnetospirillum magnetotacticum MS-1                          proteobacteria>alphaproteobacteria            COG0001: Glutamate-1-semialdehyde aminotransferase [Magnetospirillum magnetotacticum MS-1]
82944335        775       Domain_Z->NlpC->Ub+gpJ*(N)->P5->?Y->                                                                                  Magnetospirillum magneticum AMB-1                              proteobacteria>alphaproteobacteria            Phage-related protein [Magnetospirillum magneticum AMB-1]
82945132        775       Domain_Z->NlpC->Ub+gpJ*(N)->P5->?Y->                                                                                  Magnetospirillum magneticum AMB-1                              proteobacteria>alphaproteobacteria            Phage-related protein [Magnetospirillum magneticum AMB-1]
33568295        1268      Domain_Z->NlpC->Ub+gpJ*(N+distinct_C)->                                                                               Bordetella bronchiseptica RB50                                 proteobacteria>betaproteobacteria             phage-related hypothetical protein [Bordetella bronchiseptica RB50]
33564325        1318      NlpC_solo->Ub+gpJ* (N+FN3+C)->                                                                                        Bordetella pertussis Tohama I                                  proteobacteria>betaproteobacteria             phage-related conserved hypothetical protein [Bordetella pertussis Tohama I]
67545284        767       Domain_Z->NlpC solo->Ub+gpJ*(N)->(Note no JAB)*********                                                               Burkholderia vietnamiensis G4                                  proteobacteria>betaproteobacteria             phage-related conserved hypothetical protein [Burkholderia vietnamiensis G4]
68212786        1171      Domain_Z->NlpC->Ub+gpJ*(N)->                                                                                          Methylobacillus flagellatus KT                                 proteobacteria>betaproteobacteria             similar to Phage-related protein tail component [Methylobacillus flagellatus KT]
33576899        1318      Domain_Z->NlpC->Ub+gpJ*(N+FN3+distinct C)->                                                                           Bordetella bronchiseptica RB50                                 proteobacteria>betaproteobacteria             phage-related conserved hypothetical protein [Bordetella bronchiseptica RB50]
46449977        1346      NlpC_solo->Ub+gpJ*(N+FN3+distinct_C)->                                                                                Desulfovibrio vulgaris subsp. vulgaris str. Hildenborough      proteobacteria>deltaproteobacteria            tail fiber protein, putative [Desulfovibrio vulgaris subsp. vulgaris str. Hildenborough]

Versions of above without NLpC or JAB
Gis are of the Ub+gpJ protein-marked with an asterisk
GI              LENGTH    Operon                                                                                                                ORGANISM                                                       Classification                                Protein descriptions (if any)
23015894        766       ?H->Ub+gpJ(N)*->Lysozyme->?Y->                                                                                        Magnetospirillum magnetotacticum MS-1                          proteobacteria>alphaproteobacteria            COG4733: Phage-related protein, tail component [Magnetospirillum magnetotacticum MS-1]
78033450        766       Domain_Z <-PIN<-YoeB->Ub+gpJN)*->X->?Y-> (note toxin-antitoxin insert)                                                Magnetospirillum gryphiswaldense                               proteobacteria>alphaproteobacteria            phage-related protein [Magnetospirillum gryphiswaldense]
71548099        1644      Ub+gpJ*(distinctN+gp44+distinct_C) (NlpC in genome but not in vicinity)                                               Syntrophobacter fumaroxidans MPOB                              proteobacteria>deltaproteobacteria            similar to Phage-related protein tail component [Syntrophobacter fumaroxidans MPOB]
46916380        1294      Ub+gpJ(N)*                                                                                                            Photobacterium profundum SS9                                   proteobacteria>gammaproteobacteria            hypothetical protein [Photobacterium profundum SS9]


Domain_Z alignment:
FINAL                                        -HHHHHHH-------EEEEEEEEE------------EEEEEE---EEEEE------------EEEEEEEEEEE-------------EEEEE----HHHHHHHHHH-------EEEEEEEEEE-------------EEEE---EEE--EEEEEEEEE-HHHH--------------------
ALIGN                                        ----HHHH-------EEEEHEEE-------------EEEEEE----HEHHHH----------EEEEEE-----------------EEEEEEE-----HHHHHHHHH------HEEEEEEEE--------------EEEEE---------EEEHHHHHHH----------------------
HMM                                          -HHHHHHH-------EEEEEEEEE-----E------EEEEEE--EEEEEE--H---------EEEEEE--EEEE-----------EEEEEEE---HHHHHHHHHH-------EEEEEEEEEE-----------E-EEEE---EEE--EEEEEEEEE-HHHHHH-H---EEEE---------
FREQ                                         -HHHHHHHH------EEEEEEEE-------------EEEEE-----HHHHHHH---------EEEEEEE-----------------EEEEE-----HHHHHHHHH-------EEEEEEEEE---------------EE----------HHHHHHHHHH-----------------------
PSSM                                         -HHHHHH------------EEEE-------------EEEEE----EEEEE------------EEEEEE-EEEE-------------EEEEE----HHHHHHHHH--------EEEEEEEEE------------E-EEEE---EE----EEEEEEEE-------------------------
mgI418_Mgry_78033453                         SQALKEAFASAPAGTVILDTLEIWHPTFDE------PIRVVRDHADLTARLEAGAPRDG-GKRVTFAALAFEFSPPPVDT-APVPEITVTLDNVGSDITDALEGAAV-SQQVIEITWRPYLSTDLNGPHMDPPI-TMTLTDVEAD--TMRVTGRARMLDAGNK-SFPSITYTARRFPGLAR
BB3488_Bbro_33576901                         EQALKEAYASAPQDRVVFDTLELRHPAFVDPHGEPTAVRVVLGYEDIRARLETEAPLDG-GQDVMFQAGAFRFRLPGFEE-GQVPSLLIAIDGASEQIVDHVEAAVQ-SRFPIYVTYRPYLSTDLSMPQMNPPI-TMELNKVTVT--GSSVSGTATLSDVHNW-AFPHERYVRERFPGLFR
BP3364_Bper_33564327                         EKALKEAYASAPQDRVVFDTLELRHPAFVDEHGERTAVRVVLGYEDIYARLEAEAPLDG-GKEVLFQAGAFRLRLPGFEE-GQVPSLLITIDGASEKIVDHVEAAVQ-SRYPIYATYRPYVSTDLSRPQMNPPI-TMELNKVTVT--GASVSGTATLADVHNW-AFPHQRYMRERFPGLFR
Bcep1808DRAFT_4080_Bvie_67545282             SEAIKEAYASAPSQQIILHTLELRHPAFVDEDGQQVAIRVVRDTGDLWARLESQAPLQA-GERVQFVAMGFELDLPPVDT-MPVPEITVTLDNVSREIVRHLDAAAE-SQSVIEVTYRPYLSTDLEGPQMDPPI-HLVLTEVEAD--IFRVTGRARMLDVGNK-AFPGVSYTAKTFPGLTR
amb1190_Mmag_82945130                        SQALKEAFASAPAGTVILDTLEIWHPTFIE------PIRVVRDHADLTARLEAGAPRDG-GKRVTFAALAFEFSPPPVDT-APVPEITVTLDNVGSDITDALEGAAI-SQQVIEITWRPYLSTDLNGPHMDPPI-TMTLTEVEAD--TMRVTGRARMLDAGNK-SFPSITYTARRFPGLAR
amb0393_Mmag_82944333                        SQALKEAFASAPAGTVVLDTLEIWHPTFDE------PIRVVRDHADLTARLEAGAPRDG-GKRVTFAALAFEFSPPPVDT-APVPEITVTLDNVGSDITDALEGAAI-SQQVIEITWRPYLSTDLNGPHMDPPI-TMTLTEVEAD--TMRVTGRARMLDAGNK-SFPSITYTARRFPGLAR
Magn03007629_Mmag_23013169                   SQALKEAFASAPAGTVILDTLEIWHPTFDE------PIRVVRDHADLTARLETGAPRDG-GKRVTFAALAFEFSPPPVDT-APVPEITVTLDNVGSDITDALEGAAI-SQQVIEITWRPYLSTDLNGPHMDPPI-TMALTEVEAD--TMRVTGRARMLDAGNK-SFPSITYTARRFPGLAR
Magn03010833_Mmag_46200892                   SQALKEAFASAPAGTVVLDTLEIWHPSFTT------PIRVVRDHADLTARLEAGAPRDG-GKRVTFAALAFEFSPPPVDT-APVPEITVTLDNVGSDITDALEGAAI-SQQVIEITWRPYLSTDLNGPHMDPPI-TMALTEVEAD--TMRVTGRARMLDAGNK-SFPSITYTARRFPGLAR
Magn03010336_Mmag_46201139                   ---MREAFAAAPTNTVILHTLEIWHPTFSE------PIRVVRDHADLTARLEAGAPRGG-GQKVTFIALAFDLDLPPVDT-APVPEITVTMDNVGQEIVDALEAAAI-SQDKIDIIYRPFLSTDLEGPHMDPPI-TLTLAEVEAD--TLRVTGRARMLDVGNK-AFPSITYTAKRFPGLAR
MflaDRAFT_2307_Mfla_68212788                 EEAIKEAYASNPVGEVELNTLEFRHPNFVDQNGDPSAIRVVLDNVDHYLTLEDDAPLNP-GESVLFVRMAFELTKPEVDS-VAGPAMDITLNNITPEIETQIRAATR-SPYPVIGMYRLYLLSDKTQPQNNPPM-EFQLDNVNAD--DESITARATFGNEAQR-PFPNENYTATRFPGLSR
PputDRAFT_2895_Pput_82737129                 MTALEVVYAS--GGDDIVPTLEISCPAWDK------TLYLVQDFEDFRATTEA-------GKTVTFLASAIDVALPAKDN-SGAQTLTFVIDNVTGEAQQLIDASLE-AEARVTIVYREYLYSIPGEPA-DRPY-RMTSFGGTMD--GPTIQIEAGYYDLINM-MWNRFRYTTDFAPGLTY
DaceDRAFT_2556_Dace_68177301                 TTAYKEAIAYANPETTIWEAIRITHSSWLE------SILLVNSYEVFTANL---------G---SFIPVQWSMKLPEVEA-ETRGELTLKIDLLPLSIKRTLFSGAS-KTDAMKL--YYYEYTDTTDPAGQLPA-ALEISKVEMDEDNQVTTIKALYADLVNI-VFPRRRMTTTLIPGGLV
BB1708_Ppro_46916381                         KNARINLNATT-ADEPFLILVEIHHQSFSE------PARIVADTQDITHA----------G--YRYTALPIDVTLPDEGE-GKLPQAKLIIDNVGRVLTDEIDGTRG-FEGGTCVI-MQVMRSNPS--HVEWGI-ELDVLDVSID--QLKISATLGYEDMLNK-PAVTMRFTPERSPGLF-
BB1708_Bbro_33568293                         TQAKRNVNATS-ADEPLLELIEITHPDLAV------PARFVNDTQDIQVE----------G--HAFLACRFDLSIPDDQA-EQVPGARLEVDNIGRELTQWLEYSQG-GKGAKC---RLILLLRSNPSNIELDM-TMDLTGLEIT--NFRVSGDLGFKNTLMQ-SGVAMRFDPLTAPGVF-
NB311A_12117_Nsp._85716598                   SLNFRQELFGQESGEVPILLVTITHPELPE------PIYLSTDPTERFSTDPLMYRTRS-R-GIDFLYAGIDVTLPDEQD-KSPPASKLTIANVTRGLIPLARSVS--TPPAVKIEV--VLASDPDTVE-MTWP-AMDMTNLTYD--ASFLTFDLTIDALVTE-PYPSGTFSPAYFPGLFY
RB2654_16431_Rbac_84684053                   -MPWLDAINDAETAEVVLTLVTLDHADWAA------PVRLVNDVADFEHD----------G--ETYTAAGFQVAMPDQAE-DRNAAMRWTLNDVDHDVAVLLRTTN--DVIDIEVSY--VLASDPDTVQ-AGPF-EAEIRQADLR--YGSVSGALVVYPVMEEVANASFRFSTGDFPGLI-
_BPMB78_4455819                              EAAYRRKLASNPDGEMDFITLEIYHPLLSK------RWLLVRGVKDLTATLET-------GEVVTFEGTPMEAKNAANNN-DMDQTASFSLPDVLNILDEEMDRIPYDNKELPKFIFRRYVSTDLTYP-CDGPV-VYELQTLTQE----KGVFTAETGTPMLNQRATGILMTPEEIPLLRG
_BPKS7_62327363                              EAAYRRKLASNPDGEMDFITLEIYHPLLSK------RWLLVRGADDLTATLET-------GEVVTFEGTPMEAKNAANNN-DMDQTASFSLPDVLNILDEEMDRIPYDNKELPKFIFRRYVSTDLTYP-CDGPV-VYELQTLTQE----KGVFTAETGTPMLNQRATGILMTPEEIPLLRG
mgI418_Mgry_78033453                         SQALKEAFASAPAGTVILDTLEIWHPTFDE------PIRVVRDHADLTARLEAGAPRDG-GKRVTFAALAFEFSPPPVDT-APVPEITVTLDNVGSDITDALEGAAV-SQQVIEITWRPYLSTDLNGPHMDPPI-TMTLTDVEAD--TMRVTGRARMLDAGNK-SFPSITYTARRFPGLAR
D3p22_BPD3_9635614                           ATALERFYAS-DGPDLPIATIEITRPSRPH------PIFICQGFKDLTCMTED-------GRLLTFIAGAIDVSIPKRDN-SGNQNVGFAIDNVTGFAQQYIAEAID-AGEPVTLVLRIYLESDLTAPA-ERPY-RMRVKGADFE--SLTVQVEAGYYDLINT-AALRHIYNVSEFPGLKY
YintA_01000766_Yint_77979284                 MTILNRLYASG-GSEVIIQTLEIAVGDK--------TYWLTKGWEDITAVLES-------GESATFTACGIDIALPARNS-DGTQDLQFAISNIDGIVSTAIRGALD-YLSTALLTYRYYVSTDLSAPA-AKPY-TLIVKSGYWT--ATEVQITAGYMNVLDT-AWPRYRYTLPNYPGLRY
PP1578_Pput_26988310                         MSILKRLYASS-GPEIIHEVLEITDGIT--------TYWMTKGWDELTITLET-------GQVVVCTPCGMDLALPARND-DGTQDLTFALSNIDGIASGFVRAALR-DGRRMSLVYRAYTSDDLGAPA-HAPH-RFKIKGGSVT--AAQVSVTAGYFDLLDT-RWPRNTYNLNEFPGLRY
PputDRAFT_4718_Pput_82734887                 MSLIEECYASGRGE--LVDTIEARKEGGTV------SHLYCSGWEDRVCTTED-------GRTLTFVAMAMDLALPKNDN-SAFQNLVLGLDNVTGEVQEVVEEAKA-ADDRFIITFRRYLAEDLTFPQ--ERY-RMTLLSREYE--DDVAKLTAGFFDLLNT-NGLRTVLTTTLAPGLKY
_BPXp10_31788495                             SFVSNRQRLTDYSG--ILQVLEISAAYLPD------TLRLVKDVKDWTIN----------G--QDYIGLEFTITLPEDRS-GSNGVLEIKMSNVGRDVTEDLEKRPPDQMMTAVLK----LSDRETPGEFYRII-PMPIDRVSID--AQTVTLTASMDSIMRQ-QACRLRFTPFITPGLF-
_BPOP1_84570661                              SFVSNRQRLTDYSG--ILQVLEISAAYLPD------TLRLVKDVKDWTIN----------G--QDYIGLEFTITLPEDRS-GSNGVLEIKMSNVGRDVTEDLEKRPPDQMMTAVLK----LSDRQTPGEFYRII-PMPIDRVSID--AQTVTLTASMDSIMRQ-QACRLRFTPFITPGLF-
_BPXp15_66392125                             MSTFKERKQRVRDPSGLLILMELSANSFQE------TLRIANDTDNWTSN----------G--LLYYGFPFKFTGPDDSD-GSNASSKIVIDNTGRGMSDDLESLQPNEIILVKL-----MITDFYNPSA-IIR-TLYLPMMGATIRVTQMEGRCGV-DYIMRQRSVQLASSPYTAPGSY-
SfumDRAFT_2313_Sfum_71544667                 VLVREKNKLATPDPWIVLLDIELDATH---------KLYFCSNNQNVTWS----------G--RVYTAFPFLLEPTEENSKGEIPSVSLKVANVTQVIHAYLEQLDGAVGATVTI--RVVNAGYLSEDASELDM-TFTVVSTSAD--AEWIVFTLGAPNPLRR-RFPPFRFIAKHCHWEFK
DVU_2155_Dvul_46449979                       -----------------------MHPSLAA------PLRISSDPTQRTVVTDEEVVYGTVSRAETFVFVPFSISLPNDSA-EETPQTSITIDNVGREMVPTIRALTSAPEITLEMV----MASTPDVVEAVFP--GFALSSVTYD--AMSISGTLSVTEFTTE-PCPAGTFNPAEFPGMF-
consensus/100%                               ....................hph...............bhs.s................. ....h....h.h..s............h.hs.....h...h..................................h.......p.........h...s......................
consensus/80%                                ..shcc.bhss.ssp.hh.slEl.ps.h........shblsps..Dhphp......... G....a.u.shchp.P..ps.s.s..hph.lssls..l.p.lc.........h.l....hl.sc.s.s..p.sh..h.l.phphs..s.pls.ph.h.sh.pp..hspbpass..hPGL..

Species abbreviations: BPD3 : Pseudomonas phage D3; BPKS7 : Bacteriophage KS7; BPMB78 : Bacteriophage MB78; BPOP1 : Xanthomonas oryzae phage OP1; BPXp10 : Xanthomonas campestris phage Xp10; BPXp15 : Xanthomonas campestris pv. pelargonii phage Xp15; Bbro : Bordetella bronchiseptica; Bper : Bordetella pertussis; Bvie : Burkholderia vietnamiensis; Dace : Desulfuromonas acetoxidans; Dvul : Desulfovibrio vulgaris; Mfla : Methylobacillus flagellatus; Mgry : Magnetospirillum gryphiswaldense; Mmag : Magnetospirillum magneticum; Mmag : Magnetospirillum magnetotacticum; Nsp. : Nitrobacter sp.; Ppro : Photobacterium profundum; Pput : Pseudomonas putida; Rbac : Rhodobacterales bacterium; Sfum : Syntrophobacter fumaroxidans; Yint : Yersinia intermedia
-------------------------------------------------------------------------------------------------------------
6. OPERONS WITH E2-like domains

6a.  Uncharacterized operon with a triple module protein containing an E2-like, E1-like and JAB domains (Metallo beta lactamase neighbor)
    ^^^^^^^^^^^^^^^^^^^^^^^^^^^^^^^^^^^^^^^^^^^^^^^^^^^^^^^^^^^^^^^^^^^^^^^^^^^^^^^^^^^^^^^^^^^^^^^^^^^^^^^^^^^^^^^^^^^^^^^^^^^^^^^^^^^^^
Gis are of the E2+E1 containing protein- marked with an asterisk
GI              LENGTH    Operon                                                                                                                ORGANISM                                                       Classification                                Protein descriptions (if any)
21110358        750         MBL->E2+E1+JAB*->                                                                                                   Xanthomonas axonopodis pv. citri str. 306                      proteobacteria>gammaproteobacteria            conserved hypothetical protein [Xanthomonas axonopodis pv. citri str. 306]
48864353        735         MBL->E2+E1+JAB*->                                                                                                   Microbulbifer degradans 2-40, 48864354                         proteobacteria>gammaproteobacteria            COG0476: Dinucleotide-utilizing enzymes involved in molybdopterin and thiamine biosynthesis family 2 [Microbulbifer degradans 2-40]
58038271        741         MBL->E2+E1+JAB*->                                                                                                   Gluconobacter oxydans 621H,                                    proteobacteria>alphaproteobacteria            hypothetical protein GOX2518 [Gluconobacter oxydans 621H]
68246513        495         MBL->E2+E*1->                              (E2+E1) only (JAB perhaps displaced by transposon);                      Magnetococcus sp. MC-1                                         proteobacteria                                UBA/THIF-type NAD/FAD binding fold [Magnetococcus sp. MC-1]
68559822        751         MBL->E2+E1+JAB*->MBL->                                                                                              Ralstonia metallidurans CH34                                   proteobacteria>betaproteobacteria             UBA/THIF-type NAD/FAD binding fold [Ralstonia metallidurans CH34]
74421923        223         (E2 only)\  MBL->E2->E1->JAB->                                                                                      Nitrobacter winogradskyi Nb-255                                proteobacteria>alphaproteobacteria            hypothetical protein Nwi_2872 [Nitrobacter winogradskyi Nb-255]
74421925        352         (JAB only)|                                                                                                         Nitrobacter winogradskyi Nb-255                                proteobacteria>alphaproteobacteria            hypothetical protein Nwi_2874 [Nitrobacter winogradskyi Nb-255]
74421924        235                  /                                                                                                          Nitrobacter winogradskyi Nb-255 ThiF solo, 74421925: JAB,      proteobacteria>alphaproteobacteria            hypothetical protein Nwi_2873 [Nitrobacter winogradskyi Nb-255]
77387013        601         E2+E1->JAB->                                                                                                        Rhodobacter sphaeroides 2.4.1 (E2+E1, JAB neighbor)            proteobacteria>alphaproteobacteria            ThiF family protein [Rhodobacter sphaeroides 2.4.1]
77955313        851         MBL->E2+E1+JAB*->                                                                                                   Marinobacter aquaeolei VT8                                     proteobacteria>gammaproteobacteria            conserved hypothetical protein [Marinobacter aquaeolei VT8]
77955723        725         MBL->E2+E1+JAB*->                                                                                                   Marinobacter aquaeolei VT8                                     proteobacteria>gammaproteobacteria            hypothetical protein MaquDRAFT_3270 [Marinobacter aquaeolei VT8]
84502025        761         MBL->E2+E1+JAB*->                                                                                                   Oceanicola batsensis HTCC2597                                  proteobacteria>alphaproteobacteria            hypothetical protein OB2597_18097 [Oceanicola batsensis HTCC2597]
84717800        751         MBL->E2+E1+JAB*->                                                                                                   Polaromonas naphthalenivorans CJ2                              proteobacteria>betaproteobacteria             conserved hypothetical protein [Polaromonas naphthalenivorans CJ2]
85859492        1158        MBL->E2+E1+JAB+Calcineurin*->              (C-terminal calcineurin)                                                 Syntrophus aciditrophicus SB                                   proteobacteria>deltaproteobacteria            hesA/moeB/thiF type protein [Syntrophus aciditrophicus SB]
86559649        760         MBL->E2+E1+JAB*->                                                                                                   Clostridium perfringens, l                                     firmicutes                                    ThiF [Clostridium perfringens]
88705878        751         E2+E1+JAB*                                                                                                          gamma proteobacterium KT 71                                    proteobacteria>gammaproteobacteria            conserved hypothetical protein [gamma proteobacterium KT 71]
90019857        735         E2+E1+JAB*                                                                                                          Saccharophagus degradans 2-40                                  proteobacteria>gammaproteobacteria            hypothetical protein Sde_0208 [Saccharophagus degradans 2-40]
90419011        746         MBL->E2+E1+JAB*                                                                                                     Aurantimonas sp. SI85-9A1                                      proteobacteria>alphaproteobacteria            conserved hypothetical protein [Aurantimonas sp. SI85-9A1]
86475921        760         MBL->E2+E1+JAB*                                                                                                     Clostridium perfringens                                        firmicutes                                    ThiF [Clostridium perfringens]

----------------------------                                                                                                                    ------------------------
6b. Uncharacterized operon coding a multidomain protein with E2 and E1 domains  (This version of the JAB is closer to the E2+E1+JAB type)
   ^^^^^^^^^^^^^^^^^^^^^^^^^^^^^^^^^^^^^^^^^^^^^^^^^^^^^^^^^^^^^^^^^^^^^^^^^^^^^^^^^^^^^^^^^^^^^^^^^^^^^^^^^^^^^^^^^^^^^^^^^^^^^^^^^^^^^^^^^^^^^
Gis are of the E2+E1 protein- marked with an asterisk
GI              LENGTH    Operon                                                                                                                ORGANISM                                                       Classification                                Protein descriptions (if any)
71038912        589         Patatin->nuct_transferase->E2+E1*->JAB->                                                                            Psychrobacter arcticus 273-4                                   proteobacteria>gammaproteobacteria            (in the vicinity of transposase)
9654584         584         Patatin->nuct_transferase->E2+E1*->JAB->                                                                            Vibrio cholerae O1 biovar eltor str. N16961                    proteobacteria>gammaproteobacteria            (transposase in vicinity)
37927532        538         Patatin->nuct_transferase->E2+E1*->JAB->                                                                            Escherichia coli                                               proteobacteria>gammaproteobacteria            (Integrative conjugative element)
84786718        558         nuct_transferase->E2+E1*->JAB->                                                                                     Erythrobacter litoralis HTCC2594                               proteobacteria>alphaproteobacteria
85706659        550         nuct_transferase->E2+E1*->                                                                                          Roseovarius sp. 217                                            proteobacteria>alphaproteobacteria            (in the vicinty of transposase)
66965723        592         E2+E1*->JAB->                                                                                                       Arthrobacter sp. FB24                                          actinobacteria                                UBA/E1-type NAD/FAD binding fold [Arthrobacter sp. FB24]
86357617        562         Nuct_transferase->E2+E1*->JAB->                                                                                     Rhizobium etli CFN 42                                          proteobacteria>alphaproteobacteria            hypothetical protein RHE_CH01997 [Rhizobium etli CFN 42]
84499281        557         Nuct_transferase->E2+E1*->JAB->                                                                                     Oceanicola batsensis HTCC2597                                  proteobacteria>alphaproteobacteria            hypothetical protein OB2597_05120 [Oceanicola batsensis HTCC2597]
86475968        567         Nuct_transferase->E2+E1*->JAB->                                                                                     Clostridium perfringens                                        firmicutes                                    conserved hypothetical protein [Clostridium perfringens]
88937743        576         Nuct_transferase->E2+E1*->JAB->                                                                                     Geobacter uraniumreducens Rf4                                  proteobacteria>deltaproteobacteria            similar to Dinucleotide-utilizing enzymes involved in molybdopterin and thiamine biosynthesis family 1 [Geobacter uraniumreducens Rf4]
22726448        572         Nuct_transferase->E2+E1*->                                                                                          Ruegeria sp. PR1b                                              proteobacteria>alphaproteobacteria            RC170 [Ruegeria sp. PR1b]
78684828        575         Nuct_transferase->E2+E1*->                                                                                          Shewanella sp. ANA-3                                           proteobacteria>gammaproteobacteria            similar to Dinucleotide-utilizing enzymes involved in molybdopterin and thiamine biosynthesis family 2 [Shewanella sp. ANA-3]
84701417        589         nuct_transferase->E2+E1*->                                                                                          Parvularcula bermudensis HTCC2503                              proteobacteria>alphaproteobacteria            hypothetical protein PB2503_00627 [Parvularcula bermudensis HTCC2503]
2496738         583         E2+E1*->JAB->                                                                                                       Rhizobium sp. NGR234                                           proteobacteria>alphaproteobacteria            Y4QC_RHISN Hypothetical 63.6 kDa protein y4qC
2496721         593         E2+E1*->                                                                                                            Rhizobium sp. NGR234                                           proteobacteria>alphaproteobacteria            Y4OA_RHISN Hypothetical 65.2 kDa protein y4oA
92915671        591         Patatin->nuct_transferase->E1+E2*                                                                                   Mycobacterium sp. KMS                                          actinobacteria
----------------------------------------------------
6c. Uncharacterized operon coding a distinctive multidomain protein with E2 and E1 related domains
   ^^^^^^^^^^^^^^^^^^^^^^^^^^^^^^^^^^^^^^^^^^^^^^^^^^^^^^^^^^^^^^^^^^^^^^^^^^^^^^^^^^^^^^^^^^^^^^^^^^
Gis are of the E2+E1 protein- marked with an asterisk
GI              LENGTH    Operon                                                                                                                ORGANISM                                                       Classification                                Protein descriptions (if any)
2496664         519       ?->Metal?->JAB->E2+E1->                                                                                               Rhizobium sp. NGR234                                           proteobacteria>alphaproteobacteria            Y4JF_RHISN Hypothetical 55.4 kDa protein y4jF
14025925        519       ?->Metal?->JAB->E2+E1->                                                                                               Mesorhizobium loti MAFF303099                                  proteobacteria>alphaproteobacteria            mll6192 [Mesorhizobium loti MAFF303099]
20803932        485       ?->Metal?->JAB->E2+E1-> ; note part of symbiosis island (Integrated element)                                          Mesorhizobium loti                                             proteobacteria>alphaproteobacteria            HYPOTHETICAL CONSERVED TRANSMEMBRANE PROTEIN [Mesorhizobium loti]
86359719        514       ?->Metal?->JAB->E2+E1->  (plasmid p42a)                                                                               Rhizobium etli CFN 42                                          proteobacteria>alphaproteobacteria            hypothetical protein RHE_PA00014 [Rhizobium etli CFN 42]
23011188        110       Metal?->JAB->N+E1->                                                                                                   Magnetospirillum magnetotacticum MS-1                          proteobacteria>alphaproteobacteria            hypothetical protein Magn03005843 [Magnetospirillum magnetotacticum MS-1]
77690158        455       Ubl+Ubl+Ubl->Metal?->JAB->N+E1->                                                                                      Rhodopseudomonas palustris BisB5                               proteobacteria>alphaproteobacteria            UBA/THIF-type NAD/FAD binding fold [Rhodopseudomonas palustris BisB5]


-Alignment of potential metal binding domain

FINAL                           ---HHHHHHHHHHHHH-----HHHHHHHHHHHH--EEEE----EEEEEE----------EEEEEEEE---------EEE----------------------------------HHHHHHHHHHHHH--------EEEEE---EE---------------------HHHHHHHHHHHH---HHHHHHHHHHHHH---E--------
ALIGN                           ---------HHHHHHHHHHHHHHHHHHHHHHHHHHHHHHH----EEEEE-----------EEEEE-----------EEEE-------HHHHHHHHHHH------------HHHHHHHHHHHHHHHH--------EEEEE----------------EEEE-------HHHHHHHHHHH----HHHHHHHHHHHH------------
HMM                             ----HHHHHHHHHHHHHHHHH-----HHHHHHH-HHEHH---EEEEEEE-------HHHHHEEEEEE---------EEEE--------HHHHHHHHH-----------HHHHEEEHHHHHHHHHHE--------EEEEEE--EEEE--------HHHHEE-----HHHHHHHHHHHHHHHHHHHHHHHHEEE----E--------
FREQ                            --------HHHHHH-------HHHHHHHHHHHH-EEEEE-----EEEEE-----------EEEEEE----------EE----------------------------------HHHHHHHHHHHHHHH--------EEEE----------------EEE-------HHHHHHHHHHH-----HHHHHHHHHHHH------------
PSSM                            ---HHHHHHHHHHHHHHHHHHH------HHHH---EEEE----EEEEE---------EEEEEEEEEE---------EEEE-------------------------------------------------------EEE---------------------------HHHHHHHHHHHH---HHHHHEEEEEEEE---E--------
RHE_PA00016_Retl_86359721       MTESQFVDEAVSRRKFEREVAQYRELEDSYRRRGWFLLDATFPTVLVLFVALKVTPRSLVCAVRLDFTNYDLEPPSVTFVDPSTGTALPAKSLGFKMLRLNGLKEASPETVTTLAQQQRLSVQELLQAHSPDETPFLCLPGVREYHDHPAHTGDLWLLHRRSGEGSLHFILEQIWASGINPIRMLEYQIQMNFSGFQMDAAALPR
NGR234_174_Rsp._2182463         MPELQTVDPKVSRAKFDREISRFRAYADAYRMQGCFLIEESFPSAFFIFASPKVKPRVIGAAIEIDFTNYDLRPLSVVFVDPFTRQPIARKDLPLNMLRRPQLPGTPTEMISNLIQQNAVSLTDFIQANSLEDQPFLCMAGVREYHDNPAHSGDPWLLHRGSGEGCLAFILDKIIKYGTGPAEQLQIHLQVALGGLLVPPQAIPE
msi103_Mlot_20803930            MPEIQTVDPAVSRAKFDRQIGWFQTQAGAYRAQGCFLIEARFPTAFFIFAPPKIRPQIIGAAVEIDFSNYDLRPPSVVFVDPFTRRPVARKDLLLSMLRRPHLPGTPPGMISVLMQQKALSLSDFLQANSAEHTPFLCMAGVREYHDNPAHSGDSWLLHRGSGEGCLAFILDKIIKYGTGPVEQIQYQFQISVGAMVVPPSAIPE
mll8758_Mlot_14025927           MPEIQTVDPAVSRAKFDRQIGWFQTQAGAYRAQGCFLIEARFPTAFFIFAPPKIRPQIIGAAVEIDFSNYDLRPPSVVFVDPFTRRPVARKDLLLSMLRRPHLPGTPPDMISVLMQQKALSLADFLQANSAEHTPFLCMAGVREYHDNPAHSGDSWLLHRGSGEGCLAFILDKIIKYGTGPVEQIQYQFQISVGAMVVPPSAIPE
RPDDRAFT_1997_Rpal_77690160     ------MLEALSKATFDRDIGRIDPRS--VRMYDWAIVQANYPVFDVIFNHAQVAP----LRLRLVCDDWDEIPPSIELLNK----------------EGQPLATAPPNVGNVFNG----------STHPNTGRPFVCMRGAREYHTHGSHTSDLWDNYRGQSGMDLGGIVVQLWRAWKRSVG----------------------
Magn03005843_Mmag_23011188      -------------------------------------------MLDVILGHPTAAP----LRLRFTCVDWDDLPPSVELLDAA----------------GQHLSQAPPGAGGIFHP----------SPHPVTGRMFVCMRGTREYHTHFSHVGERWDGYRGQSGLDLLGILDQIWRCWKRAVG----------------------
consensus/100%                  ......h...lS+.pF-Rplubhp.b...hR.bshhllp.paPsh.hlFs..bl.P....h.lclshssaDbbP.Sl.hls.................c...L..sss...ssh............psps.p.pPFlCh.GsREYHspsuHouD.W..aR.pu..sL..Il.blh.....sh.......................

-Alignment of domain marked with a ?:

FINAL                         -HHHHHHHHEEE-HHHHH-----------E------EEEEEEE-------------EEEE-E----EEE----EE--------E-------------EEEEEEEE----EEEE------HHHHHHHHHH--------------------EEEEE-------E--HHHHHHHHH------
mll6195_Mlot_14025928         MYRQYFRIALIDYSCEAQFQPVYLPLKSRIKEGSTDSVAYPLSFAYSRPVAPSGRLKIAG-LTSRWAQAPGAGWQATGVGQMSKDSGKGD-HGG---KIEITVVVNGQPTQVEANPNQPLHVVRAKALENTQNVAQPAENWEFKDEAGNLLDVDKKVGDFGFANIVTLFLSLKAGVAGA
msi102_Mlot_20803929          MYRQYFRIALIDYSCEAQFQPVYLPLKSRIKEGSTDSVAYPLSFAYSRPVAPSGRLKIAG-LTSGWAQAPGAGWQATGVGQMSKDSGKGD-HGGGPGKIEITVVVNGQPTQVEANPNQPLHVVRAKALENTQNVAQPAENWEFKDEAGNLLDVDKKVGDFGFANIVTLFLSLKAGVAGA
y4jI_Rsp._2496667             -------------------------------------------------MAPSGRSKTASPLTGRSAVVPWGRLASHWSMTMSKEAGKGDNHGGGPGKIEIIVVVNGQPTQVEANPNQPLHVVRTKALENTQNVAQPAENWEFKDEAGTLLDADKKIGDFGFANTGTLFLSLKAGVAGA
RHE_PA00017_Retl_86359722     --------------------------------------------------------------TLDTNRSIDGVSMAKSPNTAPEAAGK---KTGSKNKITLTIVVNGEPVSVEANVNAPLHTAIAKALEESGNVGQPPENWELKDENGTVFDASKKIEDLGITAGQKLFLSLKAGAAG-

Alignment of N-terminal domain fused to E1

FINAL                          ---HHHHHHHHHHHHH----HHHHHHH--EEEEE------HHHHHHHHHHH---EEEEEE--------EEEEE--------HHHHHHH------EEEEE------------HHHHHHHHHHHHHHHHHHH----HHHHHHHHHHHHH-----
ALIGN                          ---HHHHHHHHHHHHH----HHHHHHHHHEEEEE------HHHHHHHHHHH--EEEEEE---------EEEEE--------HHHHHHHH-----EE--------------HHHHHHHHHHHHHHHHHHHH----------HHHHHH------
HMM                            ---HHHHHHHHHHHH-----HHHHHHHH-EEEEE------HHHHHHHHHH----EEEEEEE-------EEEEEEE------HHHHHHHHH----EEEEEE----------HHHHHHHHHHHHHHHHHHHHH---HHHHHHHHHHHHH-----
FREQ                           ----HHHHHHHHHHHH---HHHHHHHH--EEEEE-------HEEHHHHHHHH---EEEE-------------------HH-HHHHHH-------EEEE-------------HHHHHHH------HHEE----HHHHHHHHHHHHHHHH----
PSSM                           ---------EEEEEEE------------EEEEEE--------HHHHHHHHH---EEEEEE--------EEEEE----------HEEH-------EEEEE--------------------HHHHHHHHHHH----HHHHHHHHHH--------
RPDDRAFT_1995_Rpal_77690158    MNKATQQNAMMLASLLGVGEAEAGERLARTVLITAAPGWKSGWAVEVGELIG-RTVQVSHQQEPTDPDLELVIGDVTPRTSARRVYADLGSEGAAASLEPVAKLAG-EPHGLYAAAAACAVSAVVVHAVIDAADLPQARLPMRLDYAQLGVP
Magn03005841_Mmag_46203362     MITPAQENARMLAAILGSDEDDASERLNRAVLVTAPPGGADAAWAAEVAALLARTVGVV-TSPAEEAQLELVIGEAAARTDLPRLHAAIDAGGATVDVRPVGRTGGPPPHPLLAAVAACPAAAATLRMLLDDPALPAVAYPLRLDFDQLGVP
----------------------------------------------------
6d. Uncharacterized operon coding a Ub-like protein, a JAB, an E1-like protein and an E2-like protein
   ^^^^^^^^^^^^^^^^^^^^^^^^^^^^^^^^^^^^^^^^^^^^^^^^^^^^^^^^^^^^^^^^^^^^^^^^^^^^^^^^^^^^^^^^^^^^^^^^^^^
Gis are of the E2-like protein- marked with an asterisk

GI              LENGTH    Operon                                                                                                                ORGANISM                                                       Classification                                Protein descriptions (if any)
84717439        265       Ub->alpha_helical_2->E2*->JAB->E1->                                                                                   Polaromonas naphthalenivorans CJ2                              proteobacteria>betaproteobacteria             conserved hypothetical protein [Polaromonas naphthalenivorans CJ2]
67910471        259       Ub->alpha_helical_2->E2*->JAB->E1->                                                                                   Polaromonas sp. JS666                                          proteobacteria>betaproteobacteria             hypothetical protein BproDRAFT_0623 [Polaromonas sp. JS666]
71847775        246       Ub->alpha_helical_2->E2*->JAB->E1->                                                                                   Dechloromonas aromatica RCB                                    proteobacteria>betaproteobacteria             conserved hypothetical protein [Dechloromonas aromatica RCB]
67543573        242       Ub->alpha_helical_2-->E2*->JAB->E1->                                                                                  Burkholderia vietnamiensis G4                                  proteobacteria>betaproteobacteria             conserved hypothetical protein [Burkholderia vietnamiensis G4]
38637969        241       Ub->alpha_helical_2->E2*->JAB->E1->                                                                                   Cupriavidus necator                                            proteobacteria>betaproteobacteria             hypothetical protein PHG308 [Cupriavidus necator]
17428675        240       Ub->alpha_helical_2->E2*->JAB->E1->                                                                                   Ralstonia solanacearum                                         proteobacteria>betaproteobacteria             conserved hypothetical protein [Ralstonia solanacearum]
56315656        239       E2*->JAB->E1->                                                                                                        Azoarcus sp. EbN1                                              proteobacteria>betaproteobacteria             conserved hypothetical protein [Azoarcus sp. EbN1]
56410324        239       E2*->JAB->E1->                                                                                                        Ralstonia metallidurans CH34                                   proteobacteria>betaproteobacteria             hypothetical protein [Ralstonia metallidurans CH34]
68559357        239       E2*->JAB->E1->                                                                                                        Ralstonia metallidurans CH34                                   proteobacteria>betaproteobacteria             conserved hypothetical protein [Ralstonia metallidurans CH34]
44004435        214       E2*->alpha_helical_2->JAB->alpha_helical_2->E1->                                                                      Bacillus cereus ATCC 10987                                     firmicutes                                    hypothetical protein BCE_A0096 [Bacillus cereus ATCC 10987]
67908644        255       E2*->JAB->E1->                                                                                                        Polaromonas sp. JS666                                          proteobacteria>betaproteobacteria             hypothetical protein BproDRAFT_4305 [Polaromonas sp. JS666]
74024822        241       E2*->JAB->E1->                                                                                                        Rhodoferax ferrireducens DSM 15236                             proteobacteria>betaproteobacteria             hypothetical protein RferDRAFT_4144 [Rhodoferax ferrireducens DSM 15236]
75705484        240       E2*->JAB->E1->                                                                                                        Anabaena variabilis ATCC 29413                                 cyanobacteria                                 conserved hypothetical protein [Anabaena variabilis ATCC 29413]
17134644        240       E2*->JAB->E1->                                                                                                        Nostoc sp. PCC 7120                                            cyanobacteria                                 alr7559 [Nostoc sp. PCC 7120]
29339960        233       Ub->alpha_helical_2->E2*->E1->                                                                                        Bacteroides thetaiotaomicron VPI-5482                          bacteroidetes/chlorobi                        hypothetical protein BT_2648 [Bacteroides thetaiotaomicron VPI-5482]
71839550        243       Ub->alpha_helical_2->E2*->E1 ->SFI helicase(note connection to F-box)->JAB->                                          Pelobacter propionicus DSM 2379                                proteobacteria>deltaproteobacteria            conserved hypothetical protein [Pelobacter propionicus DSM 2379]
75758403        241       E2*->alpha_helical_2->E1->                                                                                            Bacillus thuringiensis serovar israelensis ATCC 35646          firmicutes                                    hypothetical protein RBTH_06715 [Bacillus thuringiensis serovar israelensis ATCC 35646]
75758953        253       E2*->alpha_helical_2->E1->                                                                                            Bacillus thuringiensis serovar israelensis ATCC 35646          firmicutes                                    hypothetical protein RBTH_07326 [Bacillus thuringiensis serovar israelensis ATCC 35646]

**: note: all the JABs in these operons have an N-terminal domain, whose alignment is provided below:

Domain found N-terminal to the JABs (JAB-N)

FINAL                                   -HHHHHH---EEEE--------------EEEEEE----EEEE--HHHHHHHH---------E-----HHHHHHH-
ALIGN                                   -----------EE----------------EEEEE---EEEEEE---HHHEEH----------------HH-----
HMM                                     -HHHHH----EEEEE--------H----EEEEEE--HEEEEEHHHHHHHHHHH-------EEEEE--HHHHHH--
FREQ                                    -HHHHHH----EE----------------EEEEHH---EEEEE--HHHHHE----------------HHHHHHH-
PSSM                                    -HHHHHH-----E----------------EEEEE---HEHHH----HHHHHH---------------HHHH----
PproDRAFT_0259_Ppro_71839552            MDAILQEQFPTVMVPRYGD-FVPLAHNGRRFLSASDGLWLEEKNQWLHILWPLALQN--QVAMPYGSLQKKVDFL
RSc1658_Rsol_17428674                   ---KLWDSAPTVAVPKFAE-FKQLEDVGHRFLATAEGLFVEVRRPWLHVIQPVAPLNGQTVRPPYGTVKQKVDLA
BproDRAFT_0622_Psp._67910470            LDSIIQGMFPTVIMPREGT-IAPATKNGTRYVVAGDGLWREVVLPWVTVMHKIANS---DFMLPYGAAEEAVVIK
RmetDRAFT_6239_Rmet_68559358            ADMALQQSFPSVMVPRHGA-LPALEQVGERLLIAANGVFLEIVRPWLRVVRRLGEFQH-QTAIPYGDATEVTELR
RMe0063_Rmet_56410325                   --MALQQSFPSVMVPRHGA-LPALEQVGERLLIAANGVFLEIVRPWLRVVRRLGEFQH-QTAIPYGDATEVTELR
PHG307_Cnec_38637968                    ADAALQQSFPSVMVPRFGA-LAPMERSGERLLIAANGVFLEIVRPWLSVVRHLGAFQH-RTAIPYGEAAETTDLR
Bcep1808DRAFT_6254_Bvie_67543574        LDTVLQQSFPAVMVPSRET-VVPMTRSGERLLIASDGVYLEVLRPWVRVVRRIAQY-AVSIAVPYGKVEETTALL
p1B74_Asp._56315655                     RDMALQALTPTVMVPRFGC-FEPLSQPGHRFLVGQNGEWLEVRRAWMYARVQLTQP--SPVVKPYGVVTACLEWL
BproDRAFT_4306_Psp._67908645            MDAIIQSQFPTVLAPRFEA-LSPLETTGDRFILTRHQVLMEVSRPWLHAIQAISAP--FARQTPYGAGPRLGIKL
Daro_2537_Daro_71847774                 RDLALQAVCPVIAAPRFGP-LPDM-ANGQRIILAANGVFVQVKLDWLDCIQRLSPA--LPITLPYGGIEERLAFT
RferDRAFT_4145_Rfer_74024823            -DRFLATDCPVITMPHDSEVFEPLKTPGHRLIVAAGGLYKEIRRAWLHAIVHVAR-----AQTPFGELQTTLSM-
PnapDRAFT_0123_Pnap_84717438            LDQITMGVFPLLAASQTGV-LQDPEKHGVRYVAASDGMWRAIDTAWLKA--------------------------
PproDRAFT_0259_Ppro_71839552            MDAILQEQFPTVMVPRYGD-FVPLAHNGRRFLSASDGLWLEEKNQWLHILWPLALQN--QVAMPYGSLQKKVDFL
consensus/100%                          ....l....Psl.hPp....h..h..sGpRhl.s....h.b....Wh.h...ls.........PaG.......b.
consensus/95%                           ....l....Psl.hPp....h..h..sGpRhl.s....h.b....Wh.h...ls.........PaG.......b.
consensus/90%                           ...hlb..hPslhhP+....h.sh.psGpRhlhs.pGla.El.bsWlphh..lu.........PYG.h.p...h.
consensus/85%                           ...hlb..hPslhhP+....h.sh.psGpRhlhs.pGla.El.bsWlphh..lu.........PYG.h.p...h.
consensus/80%                           .D.hLQ..hPsVhhP+.us.h.shppsGcRhlluusGlahEl.bsWlphl..lu.........PYG.hpp...h.
consensus/75%                           .D.hLQ..hPsVhhP+.us.h.shppsGcRhlluusGlahEl.bsWlphl..lu.........PYG.hpp...h.
consensus/70%                           .D.hLQpphPoVhhPRaus.hsshppsGcRhllAusGlalEl.RsWLchlbplu.........PYGshpc.s.hb

Species abbreviations
Asp. : Azoarcus sp.; Bvie : Burkholderia vietnamiensis; Cnec : Cupriavidus necator; Daro : Dechloromonas aromatica; Pnap : Polaromonas naphthalenivorans; Ppro : Pelobacter propionicus; Psp. : Polaromonas sp.; Rfer : Rhodoferax ferrireducens; Rmet : Ralstonia metallidurans; Rsol : Ralstonia solanacearum


Alignment of alpha helical domain-2
FINAL                                 ----------------------------------------------------------HHHHHH-HHHHHHHH--------------HHHHHHHHHHHHHHHH---------------------------------------HHHHHHH------EEEEEE------HHHHHHH----HHHHHHHHHHH----------HHHHHHHH-EEE---------HHHHHHHHH-------HHHHHHH-------------------------------------HHHHHHHHHHHH----HHHHHHHHHHHHHHHHH---------------------------------EEEEEEE---HHHHHHHHHHHHH---------EEEEEEE-----HHHHHHHHHHHHHHHHHHHHHHHHHHHHH--
Daro_2539_Daro_71847776               ------------------------------------MMALVLPRIGPQVPRSIAPGPLLAANAM-VSRFLIEAEAFDEADIPVTWSDSLDACRQALDGWLKCQIGALHCLTPRF--------ALHMVSRDGESYRYYGSQPPKDFDFNAVEASWCEYHEQEWPVGAGLEALSARLHGLGTVVLHVLCRQSAFV-YPLFTPDIACDVATYLYWCGED---DEEAALDMNCGEDEEEREAMRAEMVTKSMLEA------------------SFPAWTRRWPRGLELAQCARFLRRATNRLSDPGAKATAEDALALATLEIDDSFRP---DMEGE-----------FVGFGAVLSWRDGDVTTRIYDDLLELAHQGEYC-EHMGEVQVPLD-DPAAFGAWQQAMASRFAAIRLIDRLIHHLSAG
BproDRAFT_0624_Psp._67910472          -----------------------MKSIALERPVPKAHGTFVLPQISSEVPLVIGGESIAHQTLAKFSLAAEKCGMELPGG---DIPKLESIVQMQLQGWLDKQVGAN--------------------ARACLGGQPLISANSSEIEFFMRAVSNLELLKL----KPVIEALEAKVPGLGWYVVDVIERSNGRG-ISIYSPAAM-GYHSFSQLQGAESDEDFVKEMQAMEGEDEPSPEELAELIEQARSDYAYLPSKVLESVEGHAHLLGWASPNAKHGPKRLKTKQAAYLLKTAELPDGLKQCVTDAIALDCLYGK--DKGAYTWDNSQDEE-----------QIGAACFIAWNDAEMLFELVQHYEEDTYNSGTAMECLCRLKVATGGTPAEFEQMARLMRAYFDQWNALGNLLVHFLDQ
PnapDRAFT_0133_Pnap_84717448          MLDRYEHGREFECDPESASLVTTSVRNLGVVAQGNQGGMWVLPTFSPEIPLEISRADAEASNLADFILLAHKKGIRIPDSI---YTTTSELMTQQFANYARSQVKN--------------------VRVELPLDVSIVAIDKKIEFAANATDRFQGIYQL----KDSVERLNAASPGLGWFITDTIRKGHGVG-LTTYDPCRIANCVQLIWFDSET---DQEAAAEVLDIDEKNVTEAHIEQARDERTFM---PSDFLASVGGHKHLLSWSQTKKEKAACRSMSASRVRACIHKLKLVEADRALV-MAALEFHDSIKVRKANAAIAPNGWFEHENLHEFECLDALGSLAFIVWDDSEFAREAITHYEEYAMNGEGSHSQLVGLFVEL-DEPASWGPFIDAYKLYIKRYAAFSNFIGALPEE
RSc1660_Rsol_17428676                 ------------------------------------MTALALPRLAA-MPTRYRTRDDGAAWCTPALLGLVDADALSADDVRRDPATPAELLQHTLQRHWDEITAGARIFDW--------HLSANPSQLGWWIPTTTSKNLWLAITPHNNNRVDVPLYYL----GPTITTLENIRKGLGQTVLAVFYDALRLL-PNTLTPADTYGHASWVHWHGET---DETMAIQWLYDEGDFETMEQAAAAYDGPTREA---------------LFEYMPEWAAYPRRVLSDRQVRRIARSHPFVAKVVDAVDGIWNHVHATHATGGYADCRVDADGD-------------SITWIAIFRWHPEDLALRIADDFTEFVTQGEY-QDASTLVCVE--SESDSLARWLHQMRANGQLARLVENLVDLIAMP
BcenP_01000005_Bcen_84357756          -----------------------------------------------------------MLTIEDAQLADDHRNERELARIALTRTWQELTDAHSIFEWSLRLSSD---------------------SCGPSYYRTGDDNSVWVSIHSDGGAGTAPVRFL----RGSISHLESVMPGLGQTVLAVLYEACAHYLPSVLTPSETISIAGYMYWQGHA---NEIEALPELRMHYDDVDEATPEEFFEACSIPR------------RTEFFRDAPDWLVNPQQVLNTFDVHRAAEQDEIAALAVSACDEIYSLIAHGGPFARVDHFDSNAG--------------PGIDFSLFLLWDHDDGTGRVIDDFLEHEMQG-DALEAACAVSLS--LAGKAVGNWFARVRNTSRLALAVEHLLDVIALR
p1B76_Asp._56315657                   ------MRGRTYAVSRKLTEEFGVSGSASASIKRHPNDPLRLPR-KCLAPGAYVEHASAGLPLANLALALYEEGLITEADPDWGLAEVVKLGLMRLTEGTLGDLVFVAPVDL--------AVSSTLEGCEGFSVEESDPVPQTYWLALELTNALEPCFA-----GKRLLELEKAVPGLGKTALDVAQSAGART-TGCWSPLFVRDLSSYIYWRGAD---TQEEWLEELEASGEDPDDYGFSPKQYEEGFE--------------------VDWACSAQMELDGFALVQALDHPDPAVGDVAEKLCELMCLLNN-----RRSAFPDASPTDRE-----------SVYRGCLIRWDKNDPIEQVIDDHIEYANQGADCYTTLCSVWDVK-ITREDFSEWLKSYRLGLQLYKSLDQLLAMLHTS
PproDRAFT_0256_Ppro_71839549          -------------------------------MATSPPSFLSLPNIPKSVPRLY-EFDTASTCVANIALHLLDLGVVTESE---AIMPLQDIVKQSLNRWCRSKTKDLECFSPILMVSDTFAGIGGYAVDADTVLEQESITPETSIVALGITFDNTKCFTL----KDKCDTLHTVEPDFIEFVIQKLYH-SLCV-MFAVTPELAHDTADMFYWDCYD---DSDEEPYVTKE-----------------------------------EFYKIIPEWVANPTYKEGWVKEFDRCLEHDNENIRKIAQHILTWQDIEHTRKSDVLPYYPDQVSDDG---------CTTIQNGTWISWDENELFDRIIDDWGEYHYQTST-TDLNNFFVVP--ATKQGIEKGLTLLEYYFVRLEWADKLLRLVGKI
RmetDRAFT_6237_Rmet_68559356          --MLFDPRSFVPALDGGQPG-WSFARQHPAARHRPSHGFLTLPAIAAETPGRAFLSFGDEPDALELARAQFETGVLRASDV-VNPTSAADAFAQAMFAWLAARMPTCRRLNFSFS-------LVDLNAAKDQLMQFGWDDQVDASLYLAIDLPGDEVYFIG---KARADALRAVHPYLLYTAMSLINLASSKS-LHLRTPDVLLDLFARWHWEYDCTLANDDDAREFLKNGCGM-DEGDIARYLPSAVRP------ELAPDDVLPPFCHAYPE-SRKLKTVGSRKLYELARSQHGWLKDVCVALAELNLAVKRQRDR-----SAVADSQWAE-----------PAHSAATLAYAESDYVTQVLDDLYDGYANSGDATLFQCFIPIA--VEPKAIRQQFEDLSGMFKIIAALDRVLTLISD-
PHG309_Cnec_38637970                  --MLFDPRSFVPEVDAGQPA-WTPARQHPIARRRPAHDFLTLPAIPAGVPNSGLLTFGDEVDVLGLVRAQFATGVLRANDVS-TPTGAGDAFAQAMFAWLRARTPECKRLSFGFS-------LIDIGAAKDQLMQFGWEDEIEAPLYLAIDMPGDDVYFIG---EARASALRAVHPHLLYTAMSLINTASAKS-LFLRTPEALLDLFARWHWEYDSTLADDKNAREFLAESCDM-DEGDIERYLPSVVRP------ELAPDDVLPPACHGYPA-SSKLRAFGSRKLYELSRANNGWVKDLCVALAELNLTLKRQGDR-----SAVAGSQWAE-----------PAYSAATLAYRQSDYVTQVLDDLYDGLNCSGDATLFQCFIPIA--GEPKAIRQQFRDLDGMLKTIAALDRVLTLISD-
Bcep1808DRAFT_6252_Bvie_67543572      --MFFDPALPDSSIAAGSAARWQPPRAAP-ARRRPAADLLTLPSFSTEVPGAVRLKWREDVNLSDLVLKHFQYGPLRAGDVH-DPADAGDAFQQAFHAWTRRQYGRLSRLRFTPH-----LFDAHAVRDVLDGLGNGNNDDDPTPLFFGFGLEDEWVYSL----EGAIETLRSTHPLLFRTVMGALYRASART-MFIRLPDWFMYEFSCWYWDGDPHISDKDADEALKERFDDDT-E-TRSAYLPSVVRP------QLCPDDADPCVFSGGKWRYRSALTAPELMRLR--ARSRGMPRRVCTEVLKLRALMRRSRSRD-----LLHVNYAAN-----------PAYALCSVIVEDNQFVGDLLDCHFENESQSGDATTYSGFSRLA--STPKAIRRQYADLALAFRILTHLDRLLALVSQS
BproDRAFT_4304_Psp._67908643          --------------------------------------------------------------MAKLARALCNVHPELLDLVTLSEQDLPKSCIEIVERWQASLRSFLPKDALAI-----------QPEVTGYRSGNNPEFGGDLLTVQLFLDCPEPIYMK-------EFMKRCRNKVLAHDAAKAIDQVAYLG-LEIWAPEVIRDMYGSMNWYHCDNDADILEEFAMNHWEGEGEDIPAMKPEDFP----------YVLPSKWDAHMKKLGYKKPGPKPLASIQQLREMAKGRSQKDAALATAILKLRKVIKRGHLRCSDDEDRWGC----------------VEPSFVFLWDTDSAQLRHALDEAVEDRHNAGVSRENVLQVSVRPESALQQVEDDVRAVEHLLAMQIAVGDLHTAMKTF
consensus/100%                        ..............................................................h..h.b..........s................h............................................................h.............bp.....h...h...h.............P...........h.......s......................................................................................h...........................................h.........phhpp..-...ps....................h......h.........h.ph...h...
consensus/95%                         ..............................................................h..h.b..........s................h............................................................h.............bp.....h...h...h.............P...........h.......s......................................................................................h...........................................h.........phhpp..-...ps....................h......h.........h.ph...h...
consensus/90%                         ..............................................................h..h.b...p...b..s....s.....p.....h..a...b.....................................................hb.b.......h..Lps....L...hh..h.p.........bsP....s..s.h.ap..s...s........................................................................p.............h....................s................h...s.l.hppsp...phhsc..-...pu.......s.h.l........h...h..h......h..h.pll..h...
consensus/85%                         ..............................................................h..h.b...p...b..s....s.....p.....h..a...b.....................................................hb.b.......h..Lps....L...hh..h.p.........bsP....s..s.h.ap..s...s........................................................................p.............h....................s................h...s.l.hppsp...phhsc..-...pu.......s.h.l........h...h..h......h..h.pll..h...
----------------------------------------------------
6e. Uncharacterized operons coding a protein with tandem repeats of a ubiquitin-like domain (polyUbl) (First evidence of polyubiquitins in bacteria)
   ^^^^^^^^^^^^^^^^^^^^^^^^^^^^^^^^^^^^^^^^^^^^^^^^^^^^^^^^^^^^^^^^^^^^^^^^^^^^^^^^^^^^^^^^^^^^^^^^^^^^^^^^^^^^^^^^^^^^^^^^^^^^^^^^^^^^^^^^^^^^^^^^^^^
Abbreviations: Y: Metal_binding domain_1; X Unknown domain
Gis are of the E1-like protein: Marked with an asterisk

GI              LENGTH    Operon                                                                                                                ORGANISM                                                       Classification                                Protein descriptions (if any)
17134589        416       Ubl+Ubl->E2l->JAB+E1*->Y->                                                                                            Nostoc sp. PCC 7120                                            cyanobacteria                                 alr7504 [Nostoc sp. PCC 7120]
38423902        471       Ubl+Ubl->E2l->JAB+E1*->Y->                                                                                            Synechocystis sp. PCC 6803                                     cyanobacteria                                 sll6053 [Synechocystis sp. PCC 6803]
67547439        468       Ubl+Ubl+E2l->JAB+E1*->                                                                                                Burkholderia vietnamiensis G4                                  proteobacteria>betaproteobacteria             UBA/THIF-type NAD/FAD binding fold [Burkholderia vietnamiensis G4]
84711629        469       Ubl+Ubl+E2l->JAB+E1*->                                                                                                Polaromonas naphthalenivorans CJ2                              proteobacteria>betaproteobacteria             unknown protein [Polaromonas naphthalenivorans CJ2]
69928900        458       Ubl+Ubl+Ubl?+E2l->JAB+E1*->                                                                                           Nitrobacter hamburgensis X14                                   proteobacteria>alphaproteobacteria            UBA/THIF-type NAD/FAD binding fold [Nitrobacter hamburgensis X14]
86159351        604       (JAB+E1+thioredoxin-like*?)                                                                                           Anaeromyxobacter dehalogenans 2CP-C                            proteobacteria>deltaproteobacteria            UBA/THIF-type NAD/FAD binding protein [Anaeromyxobacter dehalogenans 2CP-C]
86742694        476       Ub->X+E1*->Y->                                                                                                        Frankia sp. CcI3                                               actinobacteria                                UBA/THIF-type NAD/FAD binding fold [Frankia sp. CcI3]
14025879        396       Ubl+Ubl+Ubl->X+E1*->Y->                                                                                               Mesorhizobium loti MAFF303099                                  proteobacteria>alphaproteobacteria            mlr6140 [Mesorhizobium loti MAFF303099]
68554445        389       Ubl+Ubl+Ubl->X+E1*->Y->                                                                                               Ralstonia metallidurans CH34                                   proteobacteria>betaproteobacteria             conserved hypothetical protein [Ralstonia metallidurans CH34]
28806072        392       Ubl+Ubl+Ubl->X+E1*->Y->                                                                                               Vibrio parahaemolyticus RIMD 2210633                           proteobacteria>gammaproteobacteria            hypothetical protein [Vibrio parahaemolyticus RIMD 2210633]
39651044        400       Ubl+Ubl+Ubl->X+E1*->Y->                                                                                               Rhodopseudomonas palustris CGA009                              proteobacteria>alphaproteobacteria            hypothetical protein [Rhodopseudomonas palustris CGA009]
77690161        239       Ubl+Ubl+Ubl->Metal?->JAB->N+E1*->                                                                                     Rhodopseudomonas palustris BisB5                               proteobacteria>alphaproteobacteria            hypothetical protein RPDDRAFT_1998 [Rhodopseudomonas palustris BisB5]
82740919        398       X+E1*->Y->                                                                                                            Shewanella sp. W3-18-1                                         proteobacteria>gammaproteobacteria            conserved hypothetical protein [Shewanella sp. W3-18-1]
88795472        291       Ub->E1*->                                                                                                             Alteromonas macleodii 'Deep ecotype'                           proteobacteria>gammaproteobacteria            hypothetical protein MADE_08186 [Alteromonas macleodii 'Deep ecotype']

***Note operon fusion: (The polyub is next to another operon that has been cited in a different context)

Alignment of Y: metal binding domain_1
FINAL                               HH---------EEEE-----------EEEEEE-----EEEEEE------EEEEEE--------EEEE---------------------EEEEEE--EEEEE-------
ALIGN                               HHHHH------EEEE-----------EEEEE-----EEEEEEE-------EEEEE--------EEEE----------------------EEEEE--EEEE--------
HMM                                 HH--------EEEEEE---------EEEEEEE-HH--EEEEEE------EEEEEE--------EEEEE-------EEE----------EEEEEE--EEEEE-------
FREQ                                HHHHHH-----EEEE-----------EEEEE-----EEEEEEE-------EEE----------EEEE----------------------EEEE---EEEEE-------
PSSM                                HH---------EE-------------EEEEE-------EEEE--------EEEEE-------EEEEEE--------------------EEEEEE--EEEEE-------
RES                                 LGFLKKSELTSRMVNFHPAPEEIMSGEVVIVGDRNHKKWACFRCPSGCGELILLSLNKNQHPSWRVDCDWLNRPTLHPSVRQLN-HCQCHFWIKRGVTQWCADSRHNK
sll6052_Ssp_38423901                LGFLKKSELTSRMVNFHPAPEEIMSGEVVIVGDRNHKKWACFRCPSGCGELILLSLNKNQHPSWRVDCDWLNRPTLHPSVRQLN-HCQCHFWIKRGVTQWCADSRHNK-----------------------------------------------------------
alr7505_Ana_17134590                LRFLPQPDLSARIVPTHPAPENIKPGEILVVGDAEYQKWACFRCPGGCGENILLSLNQKRHPCWAIAIDSLGRPTLNPSVRQLN-ECHCHFWVRQGVVEWCADSGQK------------------------------------------------------------
mlr6141_Mlot_14025880               -MMARVDCLTTVFVED--IPEQLDDGVLYV--SRQCHV-ALHNCACGCGEEVSTPLVPTE---YDLVMED-EGASIWPSIGNHDFPCGSHYIVKRGRIHWAGKMSREQIEAGRAYDRLLKRG--------------AQPKGLRAILAWIKRLWI-KFIG--------
Sputw3181DRAFT_3760_Ssp._82740918   ---MAVHYITPVFVEF--IPENIEQGKLYI--SETYKT-AIHKCCCGCGEEVVTPLSPAD---WQLKNGV-NTVSLYPSIGNWNYKCKSHYFINNNRIIWAPKFSPEQIQAVQVRDRVDKLNYIA-------DKNKAGPIAWSNFIGWLVKSWR-F---IRSLFSLR
RPA4125_Rpal_39651043               RKSMKLDQIKLQRVEF--MPKQLEPGILYV--SEKYRA-VAHLCACGCGAKIRTPLGITE---WAFTDNT-AGPSLWPSVGNWQQACKSHYIIDGGEIIWCGTWTPEQIMAGRRAEQARRKAHY--------DAMYVKR-------GLFNRVWQ-W---LKSLFGG-
Francci3_0886_Fsp._86566461         --MTRLDAVRHEFVEC--IPETLIQGVVYV--SIAYAT-VAHSCCCGCGNVAYTPLAPGR---WALTFDG-RSISLDPSIGNWSFPCQSHYWIERNRVHWHAAWTAEKIQKGRARTLQMI------------NKDIERTDGAKSATTAVQTRWRGWFARLRRRFK--
VP1086_Vpar_28806073                SLVLKHTHLAHKFVRS--IPKQLEPGILYV--SMEYAT-AIHSCCCGCGNQVVTPITPTD---WQLMFDG-DSISLSPSIGNWGFKCRSHYFIRKGMIVEAGQWDKKTITAGRDNDKHNKAHYYQ-------AKPKGDDNTYSHRVGLFKRVWH-WFLGKREFAKKR
RmetDRAFT_5044_Rmet_68554446        --MMRYKELEPRFVTT--VPRQLEPGVLYV--SMEYGT-VVHSCCCGCGEKVVTPLTPTD---WSITFNG-ESVSLWPSIGSWNLPCQSHYVIKGNRVLESGRWNRQMIDAEISRDNEAKAKYYKRTVSNETEPSLAHPIDIETGSQTYARQSF-WKTILSRLLR--
consensus/95%                       ........l...bV....hPcpl..G.lhl..s......sha.CssGCG..h..sls..p...a.h........ol.PSl.p....C.sHahlp.s...b.s....p............................................................
consensus/90%                       ........l...bV....hPcpl..G.lhl..s......sha.CssGCG..h..sls..p...a.h........ol.PSl.p....C.sHahlp.s...b.s....p............................................................
consensus/85%                       ...h..p.lp..hVp...hPcplb.G.lhl..sbpa...shapCssGCGp.l..sLs.sc...W.l..ss....oL.PSl.phs..CpsHahlc.s.l.bsup.s.p............................................................
consensus/80%                       ...h..p.lp..hVp...hPcplb.G.lhl..sbpa...shapCssGCGp.l..sLs.sc...W.l..ss....oL.PSl.phs..CpsHahlc.s.l.bsup.s.p............................................................
consensus/75%                       ..hh+.splp.bhVp...hPcplcsG.lYV..SbpY.s.shHpCsCGCGpblhTPLs.sc...W.lshss.pssSL.PSlGsasb.CpSHYhIcpsbl.Wsuphs.cbI...b..p...b............................h.p....b...........
consensus/70%                       ..hh+.splp.bhVp...hPcplcsG.lYV..SbpY.s.shHpCsCGCGpblhTPLs.sc...W.lshss.pssSL.PSlGsasb.CpSHYhIcpsbl.Wsuphs.cbI...b..p...b............................h.p....b...........
consensus/100%                      ........l...bV....hPcpl..G.lhl..s......sha.CssGCG..h..sls..p...a.h........ol.PSl.p....C.sHahlp.s...b.s....p............................................................

Species abbreviations:
Ana : Nostoc sp.; Fsp. : Frankia sp.; Mlot : Mesorhizobium loti; Rmet : Ralstonia metallidurans; Rpal : Rhodopseudomonas palustris; Ssp : Synechocystis sp.; Ssp. : Shewanella sp.; Vpar : Vibrio parahaemolyticus


Alignment of domain X: fused to E1: Perhaps a novel protease that displaced the JAB

FINAL                               --HHHH-----HHHHHHH--EEEEE--EEE-EEEEEEE-----EEEEEEEEEEE--------------EEEEE---------HHHHHHH-----EE------EEEEEEEE---------EE--------HHHHHHHHHHHHHHH------EE--EE--------HHHHHHHHHHHHHHHHHHHHHH---                        EEEEEE---HHHHHHHHHHH----EEEE--------------HHHHHHHHHHH--HHHHHHHHHHH-----EEEEE---HHHHHHH---EEEEEE---HHHHHHHHHHHHH----EEEEEEEE---------EEEEEEEEE-----HHHHHHHHHEE---------EEEEEEEHHHHHHHHHHHHHHHHHHHH------------EEEEE-------------
ALIGN                               -----------HHHHHH---HHHHH--EEE-EEE-------------EEEEEEEE-------------EEEE------------EEEEE------E------EEEEEEEE------------EE----EEEEEEEE-H---E--------EE--EE---------EEE------HHHHHHHHHHH----                        EEEEE-----EEEEHHH----EEEEEE------------------HHHHHHHH--HHHHHHHHHHHHHH----------H---------EEEEEEE------HEHHHHHH----EEEEE---EE------------EEEEE-------HHHH----------------HHHHHHHHHHHHHHHHHHHHHHH----E---------EEE----EE---------
HMM                                 --HHHH---HHHHHHHHH-EEEEEE--EEE-EEEEEEE-----EEEEEEEEHHHH------------EEEEEE--------HHHHHHHHH----EEEEEE-HHEEEEEEEE--------EEEEEHHEEEHHHHHHHHH--HEE------EEE--EEEE------EEEEE-----HHHHHHHHHHH----                        EEEEEE---HHHHHHHHHHHHHHHHH--------H--E-------HHHHHHHH--HHHHHHHHHHHHH-------HEHHHHHHHHH---EEEEEEEE-----EEEEEEE-----EEEEEEEEEEEE-------EEEEEEEEE----HHHHHH---E----------E--HEEEHHHHHHHHHHHHHHHHHHHH--HHHH--HEEEEEHHHHHHHH--------
FREQ                                --HHHHH----HHHHHHH---EEE---EEE-EE-----------EEEEEEEEE---------------EEEE-------------EEEE-------------EEEEEEE--------------------HHHHHHHHHHHHHH---------------------HHHHHHHHHHHH--HHHHHHHH---                        EEEEE---HHHHHHHHHHHH----EEEE----EE---HHHHHEHH-------H--HHHHHHHHHH------EEEEE--------E-----EEEE---HHHHHHHHHHHHHH----EEEE-------------EEEEEE--------HHHHHHHHHHH------------HHHHHHHHHHHHHHHHHHHHHHHH--E---------HEEE--------------
PSSM                                --HHHH-----HHHHHH---EEEE---EEE-EEE---E------EEEEEEEEEEE-------------EEEEE---------HHHHHHH-----HH-----HEEEEE----------------------EEEHHHHHH------------EE--EE-----------HHHH-H----HHHHHHHHHH--                        -EEEE-----HHHHHHHHH----EEEEE-----------------HHHHHHHH--HHHHHHHHHHH----EEEE-----HHHHHH----EEEEEE---HHHHHHHHHHHHH----EEEEE-------------E-EEEEEE------------EEEE---------EEE--------HHHHHHHHHHHHHHH---------H----EEE--------------
RPA4126_Rpal_39651044               MFQKLVSHNDDIKRLVDKGYAVGFDSNYMI-VRDIPYLDAQGSECWGAIVTKLVAT--DQGHVIQDDHQIFFAGSSPYNTDGTAIANLSDRPTALGLSEAAADVAVQRQFSNKPRIDGQLVGFNSFFDKIESYVGIISGPARAKFGSNWLTY--RSVEKVANDSVFKIHDTMTSRAEITALSAKFKDEV                        IAIIGLGGTGAYILDFMVKTPVKEIRGFDLDPFHVHNAFRSPGRFEDSEFKRS--KADVYQTRYDNFRHGLTLKAKFIDASCASDFDGVTFAFVCVDKGSSRAGIFEVLMAKGIPFIDVGMGLNRKRGP---LAGMMRATYYDPANAQAMKDKGFSELSDRPN--DEYRVNIQIGELNALNATLAVIKYKKLKGFYIETNPDFNFLFDLSDCKITRRSKIDEA
mlr6140_Mlot_14025879               MSADLISRDPHLKRLLDEGFELEMRELVLLLVHSVPYVKRDKSLGRGTLVCTLSLDTQGLTASPQTDHTMWFTGETPCHRDGAPMTNIIHNSNEATVGS---DIKVHHYFSSKPEGTGQ---YANIYDKVVTYESHLGAAARSHDKTANART-GVTLASAQDDSPFAIPDSASARYGIVAANRKLRG-R                        VAIIGLGGTGAYLLDLAAKTRVAEIHLYDDDQLLNHNLFRSPGAPEPVLAKNFPRKVDYYAALYARMHKGVKPHPTRVKADNIDEFAGYDFVFVCVDKGSSRRVIAEGLVRLGIPFVDTGIGLGLEHNT---LDGCARATFIAPGTPWAE-VATHLSFGDDDEEADVYGTEIQTAELNSLNAIMAIMRWKRWLTFYRDERNERNATYMIEGNNITNRGA----
Sputw3181DRAFT_3761_Ssp._82740919   MSSKLTVHNPSILRLIEEGFEIDIVRQHLL-VHSIPYLNQSGEVKFATLACPFVEN--GEQDTRPQDHTMWFKGEYPHDGKGRPMTEVVNSPNQHVLFD---EFGVDFYLSNKPNGQD----FSNFYDKVVHYHTLFVSQARLVDSNADGRT-GIVHGQRDESSVFCYPDTASSRAGITAITQKLEGSR                        IAIVGVGGTGSFILDLLAKTPIAEIHLFDADDFEPHNAFRAPGAASLEQLQSAPKKVDYFFDVYSAMRHGVVAHPYFLDEQNVYELDSFTFVFVAVDNGQARRVVTQHLVNRGIPFIDVGMGIEIVEDASLQLRGTCRVTLVTNEKNLHL--AQRANLHDDDDE-ALYKSNIQVADLNAMNAALAVMRWKQYMGFYLDQGQAHNLNYTLSLQSLTRDDGPEED
Francci3_0885_Fsp._86566460         MSQRLIVRSADLGRLREEGYHLETRGNVLL-VHDVPYVNPSREVLRGTLVTELELA--GDMTIQPSNHVAQFIGQTPSDSEGHPLSKLINSGAASLVG----SVHVNFTFSKKPMG-GDQ-RYRDYHHKVTTYVALLLMHAQVLDPTVAATTFPVITPDEDDDSPFEYLDTASVRAGISEVTKKLRLGP                        IAIIGLGGTGAYTLDLVAKTPVREIHLFDGDRYLQHNAFRSPGAPSIEELATVPKKVDYFAARYAKMRKKIVPHGDFVTEANVDELRGMTFVFLALDDGPARKLIVTKLEEYGIDFIDVGIGVEHVDNS---LTGLVRTTLSTVDSRKHLDADHRLPFGKANDA-NDYNRNIQIADLNALNAALAVIKWKKLAGFYLDLEREHYSAYAVNGNTLINEDLG---
VP1085_Vpar_28806072                MSLQLINLNSDLKRLRDEGYFIQVKNGFLI-MRDVPYVNSNRHVCRGTIISSLSLA--GDRTRIPDTHVVHFDGDMPCNAEGEALNAVVLQSSIFDLGR---GITAKHMFSSKPKS-G----YTDYYHKMTTYASILSGHAEVLNSGISPKV--FSTPEDEEDSVFNYTETASGRVGIGALSDLLTEES                        VAIIGLGGTGSYILDLVAKTPVREILLFDSDEFLQHNAFRAPGAPTLEALRDAEKKVEYFKSIYSNMHKRISTSSTYIDEENLELLNGVTFAFICIDAGTSKKSIVQKLEELDIPFVDVGMGVELTDGS---LGGILRVTASTSGKRQHV-HEGRVSFGGGEGN-DVYSSNIQVADLNALNAALAVIKWKKIRGFYRDLEQEHHSTYTTDGNLLLNGESCA--
RmetDRAFT_5043_Rmet_68554445        MSAALFNRNSDLKRLWDEGYRMRVEGGSLV-MLNVPYVNAKGEVKEGKIISPLLLA--GDVTQKPEPHTVHFEGEFPCDAGGKPLQAISACGVPADL-----HAVAQYYLSTKPDANG----YTDYHQKMATYAAIISGHATVLDREASPRK--VWQPLDDEESVFNYVENASGRAGIDKLTALLAGDC                        VAIIGLGGTGSYVLDFVAKTPVREIRLIDGDDFLQHNAFRAPGAPTAEQLREVPKKVDHFRSIYANMHRGIAAHAVALDASTVGLLTGVTFAFLCMDAGHGKRIAIDQLESLGVPFVDVGMGLELSNGT---LGGILRTSLSTPDCRDIA--RSTISFDEPDRD-GIYSSNIQVADLNAMNAVMAVMRWKRYRNFYRDFEGEFHSSFTTDVNMLLNGEPK---
consensus/100%                      M...L.s.sspl.RL.-cGa.h......hl.h.slPYlp.p.p..bu.lhs.h..s..s......psH.h.F.Gp.P.p..G.sh..l..pss...l......h.sp..hSpKP...s....a.shapKh.pY.s.h...Ap........p..........ppSsF.h.-shosRh.Is.hs.bh....                        lAIlGlGGTGua.LDhhsKT.l.EI..hD.D.h..HNhFRuPG..p...h.p...Ks-.a.s.Ys.h++.l..ps..lp..sh..h.uhsFsFlshD.G.u+..h.p.L..bslsFlDsGhGl...pss...L.G.hRsoh.ss.p...........h.......s.Y..pIQ.u-LNuhNA.hAlh+aKbh.sFYb-........a..p.p.l.p.......
consensus/95%                       M...L.s.sspl.RL.-cGa.h......hl.h.slPYlp.p.p..bu.lhs.h..s..s......psH.h.F.Gp.P.p..G.sh..l..pss...l......h.sp..hSpKP...s....a.shapKh.pY.s.h...Ap........p..........ppSsF.h.-shosRh.Is.hs.bh....                        lAIlGlGGTGua.LDhhsKT.l.EI..hD.D.h..HNhFRuPG..p...h.p...Ks-.a.s.Ys.h++.l..ps..lp..sh..h.uhsFsFlshD.G.u+..h.p.L..bslsFlDsGhGl...pss...L.G.hRsoh.ss.p...........h.......s.Y..pIQ.u-LNuhNA.hAlh+aKbh.sFYb-........a..p.p.l.p.......
consensus/90%                       M...L.s.sspl.RL.-cGa.h......hl.h.slPYlp.p.p..bu.lhs.h..s..s......psH.h.F.Gp.P.p..G.sh..l..pss...l......h.sp..hSpKP...s....a.shapKh.pY.s.h...Ap........p..........ppSsF.h.-shosRh.Is.hs.bh....                        lAIlGlGGTGua.LDhhsKT.l.EI..hD.D.h..HNhFRuPG..p...h.p...Ks-.a.s.Ys.h++.l..ps..lp..sh..h.uhsFsFlshD.G.u+..h.p.L..bslsFlDsGhGl...pss...L.G.hRsoh.ss.p...........h.......s.Y..pIQ.u-LNuhNA.hAlh+aKbh.sFYb-........a..p.p.l.p.......
consensus/85%                       M...L.s.sspl.RL.-cGa.h......hl.h.slPYlp.p.p..bu.lhs.h..s..s......psH.h.F.Gp.P.p..G.sh..l..pss...l......h.sp..hSpKP...s....a.shapKh.pY.s.h...Ap........p..........ppSsF.h.-shosRh.Is.hs.bh....                        lAIlGlGGTGua.LDhhsKT.l.EI..hD.D.h..HNhFRuPG..p...h.p...Ks-.a.s.Ys.h++.l..ps..lp..sh..h.uhsFsFlshD.G.u+..h.p.L..bslsFlDsGhGl...pss...L.G.hRsoh.ss.p...........h.......s.Y..pIQ.u-LNuhNA.hAlh+aKbh.sFYb-........a..p.p.l.p.......
consensus/80%                       MS.pLhs+ssclbRLb-EGa.lphc...Ll.h+slPYls.p.pl.bGslls.L.hs..Gp.s.b.psHshaF.Gp.PpsscGpshs.l..pss..sl.....ph.spabhSsKPpu.G....assaacKhsoY.ullsu.Aps.s.ssssp...h..sp.p--SsFph.-oASuRhGIs.lo.bLp...                        lAIIGLGGTGuYlLDhhAKTPV.EI+LaDsDpab.HNAFRuPGAsp.pbhbps.+KVDaa.sbYupM++.lss+s.hlc.psl.bhsGhTFsFlslD.Gpu++.lhp.L.pbGIPFlDVGhGlpb.css...LsGhhRsTh.sssp.b.h....phshscssp..s.YpsNIQlA-LNAhNAshAVh+WK+hbsFYbDbp.-ap.sas.sss.l.p.p.....

Fsp. : Frankia sp.; Mlot : Mesorhizobium loti; Rmet : Ralstonia metallidurans; Rpal : Rhodopseudomonas palustris; Ssp. : Shewanella sp.; Vpar : Vibrio parahaemolyticus
-------------------------------------------------------------------------------------------------------------

7. Ub fused to Mut7-C (Operons uninformative)
    ^^^^^^^^^^^^^^^^^^^^
Gis are of the Ub+Mut7C protein

GI              LENGTH    Operon                                                                                                                ORGANISM                                                       Classification                                Protein descriptions (if any)
41410171        200       Ub+Mut7C                                                                                                              Mycobacterium avium subsp.paratuberculosisK-10                 actinobacteria                                hypothetical protein MAP4073 [Mycobacterium avium subsp. paratuberculosis K-10]
20520977        242       Ub+Mut7C                                                                                                              Streptomyces coelicolor A3(2)                                  actinobacteria                                conserved hypothetical protein [Streptomyces coelicolor A3(2)]
71915653        241       Ub+Mut7C                                                                                                              Thermobifida fusca YX                                          actinobacteria                                conserved hypothetical protein [Thermobifida fusca YX]
54016307        251       Ub+Mut7C                                                                                                              Nocardia farcinica IFM 10152                                   actinobacteria                                hypothetical protein [Nocardia farcinica IFM 10152]
76785598        252       Ub+Mut7C                                                                                                              Mycobacterium tuberculosisF11                                  actinobacteria                                COG1656: Uncharacterized conserved protein [Mycobacterium tuberculosis F11]
13880123        236       Ub+Mut7C                                                                                                              Mycobacterium tuberculosisCDC1551                              actinobacteria                                conserved hypothetical protein [Mycobacterium tuberculosis CDC1551]
29606942        241       Ub+Mut7C                                                                                                              Streptomyces avermitilis MA-4680                               actinobacteria                                hypothetical protein [Streptomyces avermitilis MA-4680]
53688960        250       Ub+Mut7C                                                                                                              Nostoc punctiforme PCC 73102                                   cyanobacteria                                 COG1656: Uncharacterized conserved protein [Nostoc punctiforme PCC 73102]
67930484        226       Ub+Mut7C                                                                                                              Solibacter usitatus Ellin6076                                  fibrobacteres/acidobacteria                   Protein of unknown function DUF82 [Solibacter usitatus Ellin6076]
56311907        265       Ub+Mut7C                                                                                                              Azoarcus sp. EbN1                                              proteobacteria>betaproteobacteria             conserved hypothetical protein [Azoarcus sp. EbN1]
74318176        264       Ub+Mut7C                                                                                                              Thiobacillus denitrificansATCC25259                            proteobacteria>betaproteobacteria             hypothetical protein Tbd_2158 [Thiobacillus denitrificans ATCC 25259]
68554875        266       Ub+Mut7C                                                                                                              Ralstonia metallidurans CH34                                   proteobacteria>betaproteobacteria             Protein of unknown function DUF82 [Ralstonia metallidurans CH34]
74019866        268       Ub+Mut7C                                                                                                              Burkholderia ambifaria AMMD                                    proteobacteria>betaproteobacteria             Protein of unknown function DUF82 [Burkholderia ambifaria AMMD]
17431582        257       Ub+Mut7C                                                                                                              Ralstonia solanacearum                                         proteobacteria>betaproteobacteria             hypothetical protein of unknown function duf82 [Ralstonia solanacearum]
77965627        254       Ub+Mut7C                                                                                                              Burkholderia sp. 383                                           proteobacteria>betaproteobacteria             protein of unknown function DUF82 [Burkholderia sp. 383]
84363527        252       Ub+Mut7C                                                                                                              Burkholderia dolosa AUO158                                     proteobacteria>betaproteobacteria             COG1656: Uncharacterized conserved protein [Burkholderia dolosa AUO158]
48782379        251       Ub+Mut7C                                                                                                              Burkholderia fungorum LB400                                    proteobacteria>betaproteobacteria             COG1656: Uncharacterized conserved protein [Burkholderia fungorum LB400]
83719003        251       Ub+Mut7C                                                                                                              Burkholderia thailandensisE264                                 proteobacteria>betaproteobacteria             Protein of unknown function family [Burkholderia thailandensis E264]
67908809        251       Ub+Mut7C                                                                                                              Polaromonas sp. JS666                                          proteobacteria>betaproteobacteria             Protein of unknown function DUF82 [Polaromonas sp. JS666]
82701281        251       Ub+Mut7C                                                                                                              Nitrosospira multiformis ATCC25196                             proteobacteria>betaproteobacteria             Protein of unknown function DUF82 [Nitrosospira multiformis ATCC 25196]
83745680        277       Ub+Mut7C                                                                                                              Ralstonia solanacearum UW551                                   proteobacteria>betaproteobacteria             Zinc finger protein [Ralstonia solanacearum UW551]
67759108        251       Ub+Mut7C                                                                                                              Burkholderia pseudomallei S13                                  proteobacteria>betaproteobacteria             hypothetical protein BpseS_02004453 [Burkholderia pseudomallei S13]
72117336        247       Ub+Mut7C                                                                                                              Ralstonia eutropha JMP134                                      proteobacteria>betaproteobacteria             Protein of unknown function DUF82 [Ralstonia eutropha JMP134]
67738583        251       Ub+Mut7C                                                                                                              Burkholderia pseudomallei 668                                  proteobacteria>betaproteobacteria             COG1656: Uncharacterized conserved protein [Burkholderia pseudomallei 668]
67666690        259       Ub+Mut7C                                                                                                              Burkholderia cenocepacia HI2424                                proteobacteria>betaproteobacteria             Protein of unknown function DUF82 [Burkholderia cenocepacia HI2424]
67669903        251       Ub+Mut7C                                                                                                              Burkholderia pseudomallei 1655                                 proteobacteria>betaproteobacteria             hypothetical protein Bpse1_02004518 [Burkholderia pseudomallei 1655]
67154055        308       Ub+Mut7C                                                                                                              Azotobacter vinelandii AvOP                                    proteobacteria>gammaproteobacteria            Protein of unknown function DUF82 [Azotobacter vinelandii AvOP]
4981308         247       Ub+Mut7C                                                                                                              Thermotoga maritima MSB8                                       thermotogae                                   AE001747_4 conserved hypothetical protein [Thermotoga maritima MSB8]

Alignment of Ubl fused to Mut7-C

                                               <------------------------------Ub-like-------------------------------------------><------NYN/PIN------------------------------------------------------------------------------------------>|
FINAL                                          ---EEEEEHHHHHH---------EEEE------HHHHHHHEE-----EEEEEEE----------------EEEE-------|---------------EEEHHHHHHHHHHHHHHH------------HHHHHHHHHH--EEEEE-HHHHHHHHH----EEEE---HHHHHHHHHHHH--H----------------------------HHHHH----EEEEE---EEEEE---HHHHHHHHHHHHH------
ALIGN                                          ----HHHHHHHHHH--------------------EEEEEEE------EEEEEEE---------------EEEEE-------|---------------EEEHHHHHHHHHHHHHHH-------------HHHHHHHHH---EEEEHHHHHHHHHHHH----EE----HHHHHHHHHHH--H-------E---------HH--HH---------------EE-----EEEEE----HHHHHHHHHHHH------
HMM                                            ----EEEEHH-HHH--------EEEEEEE-----EEEEEEEEE---EEEEEEEEE--------------EEEEEE------|---------------HEEEHHHHHHHHHHHHHHHHHHHH------HHHHHHHHH---EEEEEHHHHHHHHHHH-EEEEEE----HHHHHHHHHHH--H-HHHHHHHHHH----HHHHHHHHHHH---HHHHHEEEEEEEE---EEEEE--HHHHHHHHHHHHHHH-----
FREQ                                           ---HHHHHHHHH------------HE-------HHHHHHHH------EEEEEEE----------------EEEE-------|--------------HEEHHHHHHHHHHHHHHHH----E--------HHHHHHHHHH--EEEE--HHHHHHHHH---EEEE---HHHHHHHHHHHH--H-------------------------------------EEEEE----EEEE----HHHHHHHHHHH-------
PSSM                                           ---EEEE----------------EEE-------HHHHHH---------HHHEE-----------------EE---------|---------------EEEEHHHHHHHHHHHH----E---------HHHHHHHHH----EEEE---HHHHH------EEE-----HHHHHHHHHHH-------------------EE------------HHH----EEEEE-----EEE---HHHHHHHHHHHHH------
Tfu_1519_Tfus_71915653                         HASITLRFDPTLRPLLAPRNRTDLLHVNHDPAASLSHVVESLGVPLTEIGELRINGTTASPSQHPQPGDLIEVLTVPKPQP|---------VPFSPIRFILDVHLGTLARRLRLLGVDTVYYT-HRDDPALVQQANEEQRILLTRDRGILYRKNLRAGGHIYASNPDEQLFEVLDRY--APPLAPWTRCLTCNGPLAQVDKDNIADQLPAGTRATYDTFVQCTECRQIYWPGAHHARLTQIIEAAQKRVAAI-
SCO4976_Scoe_20520977                          GPEIHVEFAPELHLFVPRARPTGVASAATDGVSTLGHLVESLGVPLTEVGALLVDGREVPPGHIPAGGESVRVRPVRHPQR|---------VPGAPLRFLLDVHLGTLARRLRLLGVDTAYESTDLGDPALAALSAAEKRVLLSRDRGLLRRRELWAGAYVYSTRPEEQLQEVLDRF--RPALSPWTRCTACNGLLRTATKEEVAEQLEGGTRRSYDVFAQCTACGRAYWRGAHHEQLEAIVERAVSSTRDA-
SAV3291_Save_29606942                          GPEIHVAFAPELRLFVPHERRSGTTAVGTDGASTLGHVVESLGVPLPEVGALVVNGRETPVSYIPAAGDSVEVRPVERPQR|---------VPGAPLRFLLDVHLGTLARRLRLLGVDTAYESTDLGDPALAALSAAEKRVLLSRDRGLLRRRELWAGAYIYSTRPDDQLRDVLDRF--APGLAPWTRCTACNGVLEKATKEQVADQLEGGTQRSYDVFAQCEECGRAYWKGAHHDRLEAIVERALAEFGA--
Npun02000115_Npun_53688960                     MAIAYFYFHAELNHFLPRHHKQVKISHFFEEKASIKDMIESLGVPHPEVDFINVNGKYVNFSYIVSDGDAINVYPISARSV|IIPSISVFPEPLSIIHFVVDIHLGKLATSLRLLGFDTLYRN-DYEDEKLAQISSSQGRILLTRDKGLLMRSLVTHGYYVRNTNPQEQIIEVLQRFDLFKLITPFKRCLRCNGLLEWVDKQSIIEQVPEKVRSQIDQFQRCQDCDRIYWKGSHYERLQQFIDGVLNSQKGE-
TM_0779_Tmar_4981308                           EKIAFFRFFGRLNDFFRNSERIK--THRFTGFQTVKDRIEALGVPHVEVSLITLNGKPVGFDHMVEDGELFFVYPEFQNIE|IPEDWLVTPRYIGEPRFVLDIHLGKLARLLRMLGFEAVFGE-E-SDEKLCWMAVKKKAILLSRDTGLLKRKELVFGYYVRNTDPKEQLVEVVERYDLKKWMKPFTRCIECGVELEEVPKEAVKNRVPPKVYGFFNEFARCPVCGRIYWKGSHYDHMVEFIKSNINKG----
AcidDRAFT_4098_Susi_67930484                   MPDGRFYFEGDLSLFLLPSLRGREVKRTWSDTDTLMHVIESIGVPHTEV------------ARIERDGSLIRVYPRTREIL|------------QDPRFVLDQHLGRLAAYLRMLGFDVLHTV-PAPDQHLAAASSREDRVLLTRDVGLLKRKEVRRGYFVRATDPRAQLLEVLKRFGLVDAIAPFTRCFLCNTPLESVDKAVIARQLPERIADLHNHFMRCPSCGRVYWKGSHYDRMRELIEDIKKRALFD-
nfa28300_Nfar_54016307                         ASGIELRLYAELNDFLPPQDRQDALWRPVRPHQTVKDIVEAAGVPHTEIDLLLVNGESVGFEHHPRPGDRLAAYPMFESLD|ISGLTRVRPHPLREPRMLIDVNLGGLARLLRLMGQDVRCDF-DATDARLAEISAEDHRILLTRDRGLLARRIVSHGVYVRADRPFEQIVEVIGRLDLADQLAPFTRCLRCGAVLADVAKDEIVHELSPGTRENYDTFRRCTGCGRIYWAGAHQRRLDDLVTQILAAVRR--
MtubF_01000602_Mtub_76785598                   VGYVDVRAYAELNEFVELQARGLTVRRPFRSHQTVKDVLEAMGIPHTEVDLILVNGDPADFSYRPVAGDRIAAYPMFEALD|IGSTARLRPAPLRNPRFVVDVNLGQLARLLRLLGFDTRWSS-AADDPTLADISLGEQRILLTRDRGLLKRRAITHGLFVHSQHPEEQALEVLRRLDLNGRLAPLSRCLRCNGELAAVSKDEVIGQLEPLTRRYYESFSRCFGCGRIYWPGSHHARLVRLVERLRDQLTTST
RRSL_04745_Rsol_83745680                       MPTLLFTFDASLTPLLPLTQRQRPAARAWPEGATLKHAIETFGVPHTEVGAVHVDGCAAPLESLLPARGAVAVAGVQAALP|-----------QAPLHFLCDAHLGATARLLRMAGFDTAYDN-NYADATIEALADTEDWIVLSRDRELLKRRGIRRGAFVRAREPQAQMREIVARFKLAEAARPFSRCLECNAPLRLLSAEEAASSVPPRVRERQHLFSTCDVCRRVYWPGSHWARMNTALARMLAPHQEDG
RSp1109_Rsol_17431582                          MPTLLFTFDASLTPLLPVAQRERPAARAWPEGATLKHAIETFGVPHTEVGVVQVDGHAALLDALLPARGAVAVAGVRAALP|-----------DAPLHFLCDAHLGATARLLRMAGFDTAYDN-NYADATIEALADTEDWIVLSRDRELLKRRGIRRGAFIRAREPQAQMREIVARFRLAEAARPFSRCLECNAPLRLLSAEEAAASVPPRVRERQHLFSTCDVCRRVYWPGSHWARMNTSLARMLAPHPDGA
BdolA_01000029_Bdol_84363527                   MATATFRFHGELNAFVARTQRDRAFAHACARDATLKHAIEALGVPHTEIGQLTVNGAAAGLDRPVGDGDRIDVYPERAREP|--AAAPPATPRSEQWRFVADAHLGGLAQLLRLAGFDTCYDN-HYRDDEIAALAEREGRLVLTRDRELLKRRAVARGCYLHALQPADQLRELFSRLALAPYMRPFRLCLRCNAPLHALDADAAAPRVPAGVRQRHRRFVECDVCRRVFWEGSHWRRMRALVDSMRTAAVPDE
BambDRAFT_0385_Bamb_74019866                   MATATFRFHGELNAFLARAQRGCAFAHVFARDATVKHAVEALGVPHTEIGRLCVNGAPAALDRPLGDGDRVDIHPERARPA|---IESPVQPQPESWRFIADAHLGGLAQLLRLAGFDTCYDN-HYRDDELVALAAREGRIVLTRDRELLKRRAVVRGCYLHAQQPDAQLHELFARLDLAPHMRPFRLCLRCNAPLHALDAADAAPRVPAGVRQRHRRFAACDVCRRVFWEGSHWRRMRAVVDAMRALPPVAP
Bcen2424DRAFT_1951_Bcen_67666690               MATASLRVVVELNAFLASQQRDRAFAHACARDATVKHAIEALGVPHTEIGRLYVNDAPAALDRPLDDGDRVEVLPERAGPA|---ANGATGPPPAAWRFVADAHLGGLAQLLRLAGFDTCYDN-HYRDDELAALAEREQRIVLTRDRELLKRRAVVRGCYLHALQPADQLRELFERLDLAPHMRPFRLCLRCNAPLHPLDAAAAAPSVPAGVRLRHRRFAACDVCRRVFWEGSHWRRMRAVVDAMRTPSPVRR
Bcep18194_A3405_Bsp._77965627                  MATATFRFHDELNAFLPRAQRDRAFGHACARDATLKHAIEALGVPHTEIGRLCVNDAPATLDRPLDDGDRVEAFPERAQPA|---ANGATVPPSAHWRFAADAHLGGLAQLLRLAGFDTCYDN-HYRDDELAALAAREGRIVLTRDRELLKRRAVERGCYLHALQPADQLRELFERLDLAPHMRPFRLCLRCNAPLHPLDAAAAAPRVPAGVRLRHRRFAACDVCRRVFWEGSHWRRMRTVVDAMRAPPPPAP
AvinDRAFT_7917_Avin_67154055                   MVSVTFRFYEELNDFLPSERRRQAFACDCARAATVKHMIEALGVPHTEVELVLLNGESVDFSRPLHDGDRVAVYPRFEALD|IGPLLKVRDHPLRELRFIADAHLGGLASLLRMCGFDTLYDN-HYEDRQIAALAAEQRRIVLSRDRELLKRRIVTHGCYLHALKPALQLRELFERLDLAGSARPFSRCLHCNLPLHEVTVEQARPRLPPRIAALYSRFFGCDACQRLYWEGSHWRSMRSLLAPLLDDRPPER
Bpse1_02004518_Bpse_67669903                   MVTVTFRFYEELNDFLARPLRRREFAHACMRGASVKHAIEALGVPHTEVELILVNGESTPFSHVLEEGDRVAVYPSFEAID|IRPLLRVRAAPLRVTRFIADAHLGGLAQLLRLAGFDTLYDN-HYPDKLIETIAAREARIVLTRDRELLKRRTITHGCYVRALKPQAQLQELFDRLDLAGSARPFRLCLSCNAPLRRIDPAEAAGRAPQGVLQRHTRFVTCDVCRRVFWEGSHWRRMRALIEHVSQPKPPPG
Bcep02006224_Bfun_48782379                     MVTATFRFYEELNDFLARPLRRRAFTYACAPGATAKHMIEALGVPHTEVELILVNGESVGFNHPLSDGDRLAVYPKFEALD|IHPLLRVRERPLRVVRFIADAHLGGLAPLLRLAGFDTLYDN-HYPDADIEALAAAQQRIVLTRDRELLKRRNITHGCYVRTLRPREQLREVFERLDLAGSAQPFRLCLMCNVPLRRIPKEEVGTRAPDGVLERHAQFVTCDVCRRVFWEGTHWQRMRALMDSVAAAPDRSA
Tbd_2158_Tden_74318176                         MVIATFRFYEELNDFLAPDRRKREFTVPCARAATTKHMIEALGVPHTEVELILVNGESAGFDRRLQDGDRVAVYPRFEAMD|VSPLLRVRERPLRETRFVADAHLGGLAHMLRMLGFDTLYDN-HFHDDAIVAICEHDGRIVLTRDRELLKRRSVTHGCYIHALKSEAQLREVVARLDLARSARPFTRCLHCNVPLRTVDKASVLDRLPPKVREHYAHFPTCDSCGRIYWAGSHWRNMRRLLDDVLSGERDSG
Nmul_A0146_Nmul_82701281                       MVTATFRFYEELNDFLVPERRKREFSCPCARAATTKHMIEALGVPHTEVELVLVNGESVGFDRILEHGDRVAVFPKFEMVD|VAPLLRVREHPLRVTRFIADAHLGGLAHLLRMTGFDTLYDN-NYHDRQIELLAAQEKRIVLTRDRELLKRRSITHGCYVRTLKPPEQLCEIFDRLDLAHSIKPFTLCLNCNAPLRPVEKSVVLERLPPSVRERFDHFSTCDICHRVFWEGSHWQRMRTMLEECIKPNRFGG
BproDRAFT_2323_Psp._67908809                   MVMASFRFYEELNEFLAPERRGREFACPCARAATTKHMIEALGVPHTEVELVLVNGESVGFDRQLREGDRVAVYPKFEALD|VTPLLRVRGQTLRVTRFVADAHLGGLAHLLRMAGFDTLYDN-HFRDEEIERIAAEQGRIVLTRDRDLLKRRTITHGCYVHALRTELQLREIFGRLDLARSARPFTLCLHCNAPLHAIEKMRVATMLPPQVREHYQRFSACDVCHRVFWEGSHWRRMRLMLDGLLS------
ebA822_Asp._56311907                           MVTATFRFYEELNDFLAPARRRREFDAPCARAATVKHMVEALGVPHTEVELVLVNGESVDFGRLLRDGDRVAVYPKFESLD|ITPLLRVRSHPLRVMRFVADAHLGGLAHLLRMTGFDTLYDN-HFDDGEIEIIAGRDARIVLTRDRELLKRRTLTHGCYVRALKPAQQVREIFDRLDLAGSAKPFTLCLDCNAPLRPIGKAQVEDRLPPGVRASHTRFSTCDVCRRVFWEGSHWRRMRVLVDELLAGSPPLP
RmetDRAFT_5449_Rmet_68554875                   MVTATFRFYEELNDFLAPAQRRRDLSCPCARAATVKHMIEALGVPHTEVELILVNGESSPFERIVCDGDRIAVYPKFESFD|IAPLLRVREQPLREIRFVADAHLGGLAHLLRMTGFDTLYDN-HFEDCEIARIASDEKRIVLTRDRELLKRRGITHGCYVRAIRSSLQVREIFSRLDLARSARPFSLCLDCNVPLRRIGKTDVDGRVPEGVFERHEHFVTCPHCHRVFWEGSHWRKMRTLVEELMSAQADQV
Reut_A0217_Reut_72117336                       MVTATFRFYEELNDFLAPDQRRRDLSCPCARAATVKHMIEALGVPHTEVELILVNGESSGFDRMLEDGDRVSVYPKFESLD|VSPLLRVRAHPLRIMRFVADAHLGGLAHLLRMMGFDTLYDN-HFEDSEIERIAEREGRIVLTRDRELLKRRGITHGCYVRAIKSTPQVREIFQRLDLARSARPFSLCLDCNVPLQPVARDVVADRVPPAVLERHDRFVTCDGCRRVFWEGSHWRCMRALVDELVCAG----
consensus/100%                                 .....h.h..pLp.hh.................o..c.lEshGlP.sEl.....................h.h.s......|...............+hhhD.pLG..A..LRh.G.-s........D..l...s..p..llLoRD..lL.Rp.l..G.al.s.ps..Qh.-lh.Rh.....h.PhpbC..Cs..L..h....h...h..........F..C..C.bhaW.GsH..ph...h...........
consensus/95%                                  .s.h.h.h..pLp.hl....+............o..chlEshGVP.sEl..l.lss..s...........l.h.s......|...............+FlhDhpLG..A.bLRh.GhDs.a......D..l..hu..p.bllLoRDp.LLbR+.l..Ghal.s.ps..Qh.Elh.Rh.....h.PapbC..Css.L..h....h..ph..........F..C..C.RhaW.GuHa.ph..hl..h........
consensus/90%                                  .s.h.h.h..pLp.hl....R...h..s.....o..chlEuhGVP.sEl..l.lss..ss.......Gs.l.h.s......|...............+FlhDhHLG.LApbLRh.GhDshaps.ph.D..l..hu..p.bllLoRD+.LLbR+.l..Ghal.s.ps..Qh.Elh.Rh.....h.PapbCh.CNs.L..ls...h..pl...sb...p.F..C..C.RlaW.GuHa.php.hl..h........
consensus/85%                                  .s.h.hbF..cLs.Fls...R...h..sh...sThbchlEuhGVPHTEl..l.lsG..sshs....sG-.l.lhP......|...............RFlhDhHLG.LApbLRhhGhDThaps.ph.D..l..lu..p.RllLoRDR.LL+R+.l..Ghal+s.ps..Ql.Elh.Rh.Ls..h.PapbCl.CNs.Lp.ls...h..pls..sb..ap.F..Cs.C.RlaW.GuHa.php.hlp.h........
consensus/80%                                  hs.hphbF..-Ls.Fls..bR.p.hs.shs.suTlKHhlEulGVPHTEl..l.VNG.ssshsp...sG-.l.VhP.b....|......s...s....RFlhDhHLGsLAphLRhhGFDThYcs.ph.D..l..lu.pc.RIlLoRDR.LL+RR.l.+Ghal+u.pP..QlbElh.Rh.Ls..h.PFpbCLpCNssLc.ls...h..plPs.sb.pappFs.CssC.RlaW.GoHap+hp.hlc.hbs......
consensus/75%                                  hs.hphcF..ELs.FLs..bRpp.hs.shs.suTlKHhlEuLGVPHTEl.bl.VNGpssshsp...sGDbl.VhP.b....|......s...P....RFlhDhHLGsLAphLRhhGFDThY-s.ch.D.pl..lusp-.RIlLoRDR.LLKRR.l.+GhYl+u.pP..QlbElh.RhcLA..hpPFpbCLpCNssLc.ls...hhsplPs.lb.pappFspCssC.RlaW.GSHap+hp.hl-.hbs......
consensus/70%                                  MsphpFRF..ELNsFLs..bRpc.hspshspsATlKHhlEuLGVPHTEV.hl.VNGpssshs+.l.sGDbl.VaPbb....|......sp..P....RFlhDhHLGsLApLLRhhGFDThY-s.ch.D.pl..lusp-.RIlLTRDR.LLKRR.lp+GhYl+u.cP..QlbElhpRhcLA..hpPFpbCLpCNsPLc.ls...hhsplPs.lbbpappFspCssC.RlaWcGSHacRMc.ll-.hbs......

Species abbreviations:
Asp. : Azoarcus sp.; Avin : Azotobacter vinelandii; Bamb : Burkholderia ambifaria; Bcen : Burkholderia cenocepacia; Bdol : Burkholderia dolosa; Bfun : Burkholderia fungorum; Bpse : Burkholderia pseudomallei; Bsp. : Burkholderia sp.; Mtub : Mycobacterium tuberculosis; Nfar : Nocardia farcinica; Nmul : Nitrosospira multiformis; Npun : Nostoc punctiforme; Psp. : Polaromonas sp.; Reut : Ralstonia eutropha; Rmet : Ralstonia metallidurans; Rsol : Ralstonia solanacearum; Save : Streptomyces avermitilis; Scoe : Streptomyces coelicolor; Susi : Solibacter usitatus; Tden : Thiobacillus denitrificans; Tfus : Thermobifida fusca; Tmar : Thermotoga maritima
-------------------------------------------------------------------------------------------------------------

8. Uncharacterized operon encoding a Ub-like (RnfH)  family protein
^^^^^^^^^^^^^^^^^^^^^^^^^^^^^^^^^^^^^^^^^^^^^^^^^^^^^^^^^^^^^^
Abbreviations: c/d: aromatic cyclase/dehydrase (c/d)--82
Gis are of the Ub/RnfH protein

GI              LENGTH    Operon                                                                                                                ORGANISM                                                       Classification                                Protein descriptions (if any)
13362951        102          <-SmpB-c/d->Ub*-><-SmpA-                                                                                           Escherichia coli O157:H7                                       proteobacteria>gammaproteobacteria
67549235        107          <-SmpB-c/d->Ub*->                                                                                                  Burkholderia vietnamiensis G4                                  proteobacteria>betaproteobacteria
24373049        111          <-SmpB-c/d->Ub*-><-SmpA                                                                                            Shewanella oneidensis MR-1                                     proteobacteria>gammaproteobacteria
9655302         103          <-SmpB-c/d->Ub*-><-SmpA-                                                                                           Vibrio cholerae O1 biovar eltor str. N16961                    proteobacteria>gammaproteobacteria
71898356        84           <-SmpB-c/d->Ub*-><-SmpA-                                                                                           Xylella fastidiosa Ann-1                                       proteobacteria>gammaproteobacteria
45435745        94           <-SmpB-c/d->Ub*-><-SmpA-                                                                                           Yersinia pestis biovar Medievalis str. 91001                   proteobacteria>gammaproteobacteria
75857320        117          <-SmpB-c/d->Ub*-><-SmpA-                                                                                           Vibrio sp. Ex25                                                proteobacteria>gammaproteobacteria
46156590        110          -c/d->Ub*->                                                                                                        Haemophilus somnus 2336                                        proteobacteria>gammaproteobacteria
67638926        107          <-SmpB-c/d->Ub*->                                                                                                  Burkholderia mallei 10399                                      proteobacteria>betaproteobacteria
84387679        103          <-SmpB-c/d->Ub*-><-SmpA-                                                                                           Vibrio splendidus 12B01                                        proteobacteria>gammaproteobacteria
46202194        97           Ub*                                                                                                                Magnetospirillum magnetotacticum MS-1                          proteobacteria>alphaproteobacteria
16421233        96           <-SmpB-c/d->Ub*-><-SmpA-                                                                                           Salmonella typhimurium LT2                                     proteobacteria>gammaproteobacteria
77958607        94           <-SmpB-c/d->Ub*-><-SmpA-                                                                                           Yersinia bercovieri ATCC 43970                                 proteobacteria>gammaproteobacteria
7379707         92           FtsJ->FtsH->c/d->Ub*                                                                                               Neisseria meningitidis Z2491                                   proteobacteria>betaproteobacteria
58581648        91           <-SmpB-c/d->Ub*-><-SmpA-                                                                                           Xanthomonas oryzae pv. oryzae KACC10331                        proteobacteria>gammaproteobacteria
52627727        90           -c/d->Ub*-><-SmpA-                                                                                                 Legionella pneumophila subsp. pneumophila str                  proteobacteria>gammaproteobacteria
76579340        269          <-SmpB-Ub*->                                                                                                       Burkholderia pseudomallei 1710b                                proteobacteria>betaproteobacteria
68245723        165          Ub*                                                                                                                Magnetococcus sp. MC-1                                         proteobacteria
71362697        157          Ub*-><-SmpA-                                                                                                       Psychrobacter cryohalolentis K5                                proteobacteria>gammaproteobacteria
71037825        134          Ub*-><-SmpA-                                                                                                       Psychrobacter arcticus 273-4                                   proteobacteria>gammaproteobacteria
71143975        131          <-SmpB-c/d->Ub*-><-SmpA-                                                                                           Colwellia psychrerythraea 34H                                  proteobacteria>gammaproteobacteria
84717489        129          <-SmpB-c/d->Ub*->                                                                                                  Polaromonas naphthalenivorans CJ2                              proteobacteria>betaproteobacteria
56179014        125          <-SmpB-c/d->Ub*->                                                                                                  Idiomarina loihiensis L2TR                                     proteobacteria>gammaproteobacteria
68559691        121          Hjlc<-SmpB-c/d->Ub*->                                                                                              Ralstonia metallidurans CH34                                   proteobacteria>betaproteobacteria
17428441        118          Hjlc<-SmpB-c/d->Ub*->                                                                                              Ralstonia solanacearum                                         proteobacteria>betaproteobacteria
83749959        118          Hjlc<-SmpB-c/d->Ub*->                                                                                              Ralstonia solanacearum UW551                                   proteobacteria>betaproteobacteria
37197746        117          <-SmpB-c/d->Ub*-><-SmpA-                                                                                           Vibrio vulnificus YJ016                                        proteobacteria>gammaproteobacteria
67158728        117          <-SmpB-c/d->Ub*-><-SmpA-                                                                                           Azotobacter vinelandii AvOP                                    proteobacteria>gammaproteobacteria
33576043        116          <-SmpB-c/d->Ub*->                                                                                                  Bordetella bronchiseptica RB50                                 proteobacteria>betaproteobacteria
67677127        115          <-SmpB-c/d->Ub*-><-SmpA-                                                                                           Chromohalobacter salexigens DSM 3043                           proteobacteria>gammaproteobacteria
74020818        115          -c/d->Ub*->                                                                                                        Rhodoferax ferrireducens DSM 15236                             proteobacteria>betaproteobacteria
85711977        115          -c/d->Ub*-><-SmpA-SmpB->                                                                                           Idiomarina baltica OS145                                       proteobacteria>gammaproteobacteria
68545933        112          <-SmpB-c/d->Ub*-><-SmpA-                                                                                           Shewanella amazonensis SB2B                                    proteobacteria>gammaproteobacteria
74317772        112          <-SmpB-c/d->Ub*-><-SmpA-                                                                                           Thiobacillus denitrificans ATCC 25259                          proteobacteria>betaproteobacteria
69157448        111          <-SmpB-c/d->Ub*-><-SmpA-                                                                                           Shewanella denitrificans OS217                                 proteobacteria>gammaproteobacteria
69952904        111          <-SmpB-c/d->Ub*-><-SmpA-                                                                                           Shewanella frigidimarina NCIMB 400                             proteobacteria>gammaproteobacteria
72118961        111          <-SmpB-c/d->Ub*->                                                                                                  Ralstonia eutropha JMP134                                      proteobacteria>betaproteobacteria
48787671        110          Hjlc<-SmpB-c/d->Ub*->                                                                                              Burkholderia fungorum LB400                                    proteobacteria>betaproteobacteria
52306665        110          Ub*                                                                                                                Mannheimiasucciniciproducens MBEL55E                           proteobacteria>gammaproteobacteria
68212386        110          <-SmpB-c/d->Ub*-><-SmpA-                                                                                           Methylobacillus flagellatus KT                                 proteobacteria>betaproteobacteria
78366485        110          <-SmpB-c/d->Ub*-><-SmpA-                                                                                           Shewanella sp. PV-4                                            proteobacteria>gammaproteobacteria
67907298        109          <-SmpB-c/d->Ub*-><-SmpA-                                                                                           Polaromonas sp. JS666                                          proteobacteria>betaproteobacteria
46912321        108          <-SmpB-c/d->Ub*-><-SmpA-                                                                                           Photobacterium profundum SS9                                   proteobacteria>gammaproteobacteria
48861843        108          -c/d->Ub*-><-SmpA-                                                                                                 Microbulbifer degradans 2-40                                   proteobacteria>gammaproteobacteria
71847580        108          <-SmpB-c/d->Ub*-><-SmpA-                                                                                           Dechloromonas aromatica RCB                                    proteobacteria>betaproteobacteria
76791575        108          <-SmpB-c/d->Ub*->                                                                                                  Pseudoalteromonas atlantica T6c                                proteobacteria>gammaproteobacteria
56315277        107          <-SmpB-c/d->Ub*->                                                                                                  Azoarcus sp. EbN1                                              proteobacteria>betaproteobacteria
76874703        107          <-SmpB-c/d->Ub*-><-SmpA-                                                                                           Pseudoalteromonas haloplanktis TAC125                          proteobacteria>gammaproteobacteria
47571785        106          <-SmpB-c/d->Ub*->                                                                                                  Rubrivivax gelatinosus PM1                                     proteobacteria>betaproteobacteria
71548925        105          Cox15-><-SmpB--c/d->Ub*->                                                                                          Nitrosomonas eutropha C71                                      proteobacteria>betaproteobacteria
28871646        104          DC3000;<-SmpB--X->-c/d->Ub*->-X->-X-><-SmpA-                                                                       Pseudomonas syringae pv. tomato str.                           proteobacteria>gammaproteobacteria
66047427        104          <-SmpB--X->-c/d->Ub*-><-SmpA-                                                                                      Pseudomonas syringae pv. syringae B728a                        proteobacteria>gammaproteobacteria
68348616        104          <-SmpB--X->-c/d->Ub*-><-SmpA-                                                                                      Pseudomonas fluorescens Pf-5                                   proteobacteria>gammaproteobacteria
71558661        104          <-SmpB--X->-c/d->Ub*-><-SmpA-                                                                                      Pseudomonas syringae pv. phaseolicola 1448A                    proteobacteria>gammaproteobacteria
77380988        104          <-SmpB-X->c/d->Ub*-><-SmpA-                                                                                        Pseudomonas fluorescens PfO-1                                  proteobacteria>gammaproteobacteria
30138331        103          <-SmpB-c/d->Ub*->                                                                                                  Nitrosomonas europaea ATCC 19718                               proteobacteria>betaproteobacteria
33572188        103          <-SmpB-c/d->Ub*->                                                                                                  Bordetella pertussis Tohama I                                  proteobacteria>betaproteobacteria
49530092        103          ->Ub*-><-SmpA                                                                                                      Acinetobacter sp. ADP1                                         proteobacteria>gammaproteobacteria
59712607        103          <-SmpB-c/d->Ub*-><-SmpA-                                                                                           Vibrio fischeri ES114                                          proteobacteria>gammaproteobacteria
68057197        102          Ub*                                                                                                                Haemophilusinfluenzae 86-028NP                                 proteobacteria>gammaproteobacteria
29541871        101          <-SmpB-c/d->Ub*-><-SmpA-                                                                                           Coxiella burnetii RSA 493                                      proteobacteria>gammaproteobacteria
33149060        100          -c/d->Ub*->                                                                                                        Haemophilus ducreyi 35000HP                                    proteobacteria>gammaproteobacteria
34498917        100          -c/d->Ub*-><-SmpA-                                                                                                 Chromobacterium violaceum ATCC 12472                           proteobacteria>betaproteobacteria
10038928        99           <-SmpB-Ub*->                                                                                                       Buchnera aphidicola str. APS (Acyrthosipho                     proteobacteria>gammaproteobacteria
12720385        99           -c/d->Ub*->                                                                                                        Pasteurella multocida subsp. multocida str                     proteobacteria>gammaproteobacteria
76883017        99           <-SmpB-c/d->Ub*-><-SmpA-                                                                                           Nitrosococcus oceani ATCC 19707                                proteobacteria>gammaproteobacteria
87120236        99           <-SmpB--X->-c/d->Ub*-><-SmpA-                                                                                      Marinomonas sp. MED121                                         proteobacteria>gammaproteobacteria
21112537        98           -serinepeptidase-><-SmpB--c/d->Ub*-><-SmpA-                                                                        Xanthomonas campestris pv. campestris str. ATC                 proteobacteria>gammaproteobacteria
32035426        98           -c/d->Ub*->                                                                                                        Actinobacillus pleuropneumoniae serovar 1 str                  proteobacteria>gammaproteobacteria
78035544        98           -serinepeptidase-><-SmpB--c/d->Ub*-><-SmpA-                                                                        Xanthomonas campestris pv. vesicatoria str                     proteobacteria>gammaproteobacteria
36786684        97           <-SmpB-c/d->Ub*-><-SmpA-                                                                                           Photorhabdus luminescens subsp. laumondii TTO1                 proteobacteria>gammaproteobacteria
78701201        97           <-SmpB-c/d->Ub*-><-SmpA-                                                                                           Alkalilimnicola ehrlichei MLHE-1                               proteobacteria>gammaproteobacteria
82702330        96           -c/d->Ub*-><-SmpA-                                                                                                 Nitrosospira multiformis ATCC 25196                            proteobacteria>betaproteobacteria
83644081        96           <-SmpB--X->-c/d->Ub*-><-SmpA-                                                                                      Hahella chejuensis KCTC 2396                                   proteobacteria>gammaproteobacteria
21623148        95           <-SmpB--Ub*->                                                                                                      Buchnera aphidicola str. Sg (Schizaphi                         proteobacteria>gammaproteobacteria
77978427        94           <-SmpB-c/d->Ub*-><-SmpA-                                                                                           Yersinia intermedia ATCC 29909                                 proteobacteria>gammaproteobacteria
84780300        94           <-SmpB-c/d->Ub*-><-SmpA-                                                                                           Sodalis glossinidius str. 'morsitans'                          proteobacteria>gammaproteobacteria
49610307        93           <-SmpB-c/d->Ub*-><-SmpA-                                                                                           Erwinia carotovora subsp. atroseptica SCRI1043                 proteobacteria>gammaproteobacteria
77953016        92           <-SmpB--X->-c/d->Ub*-><-SmpA-                                                                                      Marinobacter aquaeolei VT8                                     proteobacteria>gammaproteobacteria
78364037        92           <-SmpB-c/d->Ub*-><-SmpA-                                                                                           Thiomicrospira crunogena XCL-2                                 proteobacteria>gammaproteobacteria
21107692        87           -serinepeptidase-><-SmpB--c/d->Ub*-><-SmpA-                                                                        Xanthomonas axonopodis pv. citri str. 306                      proteobacteria>gammaproteobacteria
27904127        86           Ub*-><-SmpA-                                                                                                       Buchneraaphidicola str. Bp (Baizongi                           proteobacteria>gammaproteobacteria
-------------------------------------------------------------------------------------------------------------

9. Mobile RnfH operon (electron transport chain--9)
^^^^^^^^^^^^^^^^^^^^^^^^^^^^^^^^^^^^^^^^^^^^^^^^^^^^
Gis are of the RnfH protein (marked with an asterisk)
GI              LENGTH    Operon                                                                                                                ORGANISM                                                       Classification                                Protein descriptions (if any)
56312934        101       rnfB->rnfC->rnfD->rnfG->rnfE->(Ub)rnfH*->                                                                             Azoarcus sp. EbN1;                                             proteobacteria>betaproteobacteria             Protein rnfH [Azoarcus sp. EbN1]
71846749        90        rnfB->rnfC->rnfD->rnfG->rnfE->(Ub)rnfH*->                                                                             Dechloromonas aromatica RCB;                                   proteobacteria>betaproteobacteria             Protein of unknown function UPF0125 [Dechloromonas aromatica RCB]
56552704        88        rnfB->rnfC->rnfD->rnfG->rnfE->(Ub)rnfH*->                                                                             Zymomonas mobilis subsp. mobilis ZM4;                          proteobacteria>alphaproteobacteria            hypothetical protein ZMO1808 [Zymomonas mobilis subsp. mobilis ZM4]
53756757        95        rnfB->rnfC->rnfD->rnfG->rnfE->(Ub)rnfH*->                                                                             Methylococcus capsulatus str. Bath;                            proteobacteria>gammaproteobacteria            electron transport complex, H subunit [Methylococcus capsulatus str. Bath]
9843879         86        rnfB->rnfC->rnfD->rnfG->rnfE->(Ub)rnfH*->                                                                             Pseudomonas stutzeri;                                          proteobacteria>gammaproteobacteria            RnfH protein [Pseudomonas stutzeri]
77389630        85        rnfB->rnfC->rnfD->rnfG->rnfE->(Ub)rnfH*->                                                                             Rhodobacter sphaeroides 2.4.1;                                 proteobacteria>alphaproteobacteria            probable rnfH protein [Rhodobacter sphaeroides 2.4.1]
67158346        86        rnfB->rnfC->rnfD->rnfG->rnfE->(Ub)rnfH*->                                                                             Azotobacter vinelandii AvOP;                                   proteobacteria>gammaproteobacteria            Protein of unknown function UPF0125 [Azotobacter vinelandii AvOP]
1905814         85        rnfB->rnfC->rnfD->rnfG->rnfE->(Ub)rnfH*->                                                                             Rhodobacter capsulatus;                                        proteobacteria>alphaproteobacteria            RnfH protein [Rhodobacter capsulatus]
46202216        84        rnfB->rnfC->rnfD->rnfG->rnfE->(Ub)rnfH*->                                                                             Magnetospirillum magnetotacticum MS-1;                         proteobacteria>alphaproteobacteria            COG2914: Uncharacterized protein conserved in bacteria [Magnetospirillum magnetotacticum MS-1]
-------------------------------------------------------------------------------------------------------------

10. Aromatic amino acid hydroxylase; TolueneO-Xylene Monooxygenase Hydroxylase protein B
^^^^^^^^^^^^^^^^^^^^^^^^^^^^^^^^^^^^^^^^^^^^^^^^^^^^^^^^^^^^^^^^^^^^^^^^^^^^^^^^^^^^^^^^
Gis are of the TmoB/Ub  protein- marked with an asterisk
GI              LENGTH    Operon                                                                                                                ORGANISM                                                       Classification                                Protein descriptions (if any)
78693154        81        TmoA->TmoB/Ub*->TmoC->TmoD->TmoE->TmoF                                                                                Bradyrhizobium sp. BTAi1                                       proteobacteria>alphaproteobacteria            hypothetical protein BradDRAFT_6557 [Bradyrhizobium sp. BTAi1]
48094248        86        TmoA->TmoB/Ub*->TmoC->TmoD->TmoE->TmoF                                                                                Pseudomonas sp. OX1                                            proteobacteria>gammaproteobacteria            toluene o-xylene monooxygenase component [Pseudomonas stutzeri]
68556036        102       4OCDC->4OCTT->TmoA->TmoB/Ub*->TmoC->TmoD->TmoE->TmoF                                                                  Ralstonia metallidurans CH34                                   proteobacteria>betaproteobacteria             Toluene-4-monooxygenase system B [Ralstonia metallidurans CH34]
5911739         94        TmoA->TmoB/Ub*->TmoC->TmoD->TmoE->TmoF                                                                                Rhodococcus sp. AD45                                           actinobacteria                                putative isoprene monooxygenase gamma subunit [Rhodococcus sp. AD45]
45479222        84        TmoA->TmoB/Ub*->TmoC->TmoD->TmoE->TmoF                                                                                Pseudomonas mendocina                                          proteobacteria>gammaproteobacteria            gammahydroxylase [Pseudomonas mendocina]
1754624         88        TmoA->TmoB/Ub*->TmoC->TmoD                                                                                            Pseudomonas aeruginosa                                         proteobacteria>gammaproteobacteria            bmoB[Pseudomonas aeruginosa]
71849051        88        TmoA->TmoB/Ub*->TmoC->TmoD->TmoE->TmoF->TodX                                                                          Dechloromonas aromatica RCB                                    proteobacteria>betaproteobacteria             Toluene-4-monooxygenase system B [Dechloromonas aromatica RCB]
86565792        82        TmoA->TmoB/Ub*->TmoC->TmoD->TmoE                                                                                      Frankia sp. CcI3                                               actinobacteria                                Toluene-4-monooxygenase system B [Frankia sp. CcI3]
4210875         88        TmoA->TmoB/Ub*->TmoC->TmoD->TmoE->TmoF                                                                                Xanthobacter autotrophicus Py2                                 proteobacteria>alphaproteobacteria            oxygenase gamma subunit [Xanthobacter sp. Py2]
72122837        86        Note:phenol hydroxylase operon<-TmoA->TmoB/Ub*->TmoC->TmoD->TmoE->TmoF                                                Ralstonia eutropha JMP134                                      proteobacteria>betaproteobacteria             Toluene-4-monooxygenase system B [Ralstonia eutropha JMP134]
44893909        86        TmoA->TmoB/Ub*->TmoC->TmoD->TmoE->TmoF                                                                                Ralstonia pickettii                                            proteobacteria>betaproteobacteria             gamma hydroxylase subunit [Ralstonia pickettii]
2150114         89        4OCDC->TmoA->TmoB/Ub*->TmoC->TmoD->TmoE->TmoF                                                                         Burkholderia cepacia                                           proteobacteria>betaproteobacteria             TbhB[Burkholderia cepacia]

Abbreviations:
TmoA: Toluene-4-monooxygenase hydroxylase; Ferritin-like
TmoD: hydroxylase/monooxygenase regulatory protein; Ferritin-like
TmoE: Toluene-4-monooxygenase hydroxylase
TmoB: Ubiquitin fold
TmoC: Rieske 2Fe-S protein
TmoF: NADH-ferredoxin oxidoreductase
4OCDC: 4-oxalocrotonate decarboxylase
4OCTT: 4-oxalocrotonate tautomerase
TodX: Aromatic amino acid transporter, Porin like beta-barrel
* Note The ribonucleotide large and small subunits also correspond to the TmoA/D pair
-------------------------------------------------------------------------------------------------------------

11. YukD-like proteins

Abbreviations:
YukD: YukD like ubiquitin
S/TK: serine/threonine kinase;
gis are of the YukD-like Ub protein protein- marked with an asterisk

GI              LENGTH    Operon                                                                                                                                                                           ORGANISM                                                       Classification                                Protein descriptions (if any)
15026816        90        <-FtsK<-S/TK<-yukD*<-?<-ESAT-6                                                                                                                                                   Clostridium acetobutylicum ATCC 824                            firmicutes                                    AE007866_9 Hypothetical protein [Clostridium acetobutylicum ATCC 824]
15022859        81        yukD*->FtsK->ESAT-6->                                                                                                                                                            Clostridium acetobutylicum ATCC 824                            firmicutes                                    E007517_5 Hypothetical protein [Clostridium acetobutylicum ATCC 824]
52004898        79        <-Mem_prot<-FtsK<-S/TK<-yukD*<-ESAT-6                                                                                                                                            Bacillus licheniformis ATCC 14580                              firmicutes                                    conserved protein YukD [Bacillus licheniformis ATCC 14580]
2635685         79        <-Mem_prot<-FtsK<-FtsK<-S/TK<-yukD*<-ESAT-6                                                                                                                                      Bacillus subtilis subsp. subtilis str. 168                     firmicutes                                    yukD [Bacillus subtilis subsp. subtilis str. 168]
56908701        79        ESAT-6->yukD*->S/TK->FtsK->Mem_prot->                                                                                                                                            Bacillus clausii KSM-K16                                       firmicutes                                    conserved hypothetical protein [Bacillus clausii KSM-K16]
10173588        80        <-Mem_prot||ESAT-6->yukD*->S/TK->FtsK->?->?->transp->                                                                                                                            Bacillus halodurans C-125                                      firmicutes                                    BH0973 [Bacillus halodurans C-125]
67875114        82        yukD*                                                                                                                                                                            Clostridium thermocellum ATCC 27405                            firmicutes                                    hypothetical protein CtheDRAFT_2497 [Clostridium thermocellum ATCC 27405]
76563722        80        <-FtsK<-S/TK<-yukD*<-?<-Mem_prot<-ESAT-6<-ESAT-6                                                                                                                                 Streptococcus agalactiae A909                                  firmicutes                                    conserved hypothetical protein [Streptococcus agalactiae A909]
88194066        93        ESAT-6->Mem_prot->?->yukD*->S/TK->FtsK->                                                                                                                                         Staphylococcus aureus subsp. aureus NCTC 8325                  firmicutes                                    hypothetical protein SAOUHSC_00260 [Staphylococcus aureus subsp. aureus NCTC 8325]
49482522        80        ESAT-6->Mem_prot->?->yukD*->S/TK->FtsK->?->?->transp->                                                                                                                           Staphylococcus aureus subsp. aureus MRSA252                    firmicutes                                    hypothetical protein SAR0282 [Staphylococcus aureus subsp. aureus MRSA252]
22776996        84        ESAT-6->Mem_prot->?->yukD*->S/TK->FtsK->?->?->transp->                                                                                                                           Oceanobacillus iheyensis HTE831                                firmicutes                                    hypothetical conserved protein [Oceanobacillus iheyensis HTE831]
16412473        83        ESAT-6->Mem_prot->?->yukD*->S/TK->FtsK->                                                                                                                                         Listeria innocua                                               firmicutes                                    lin0052 [Listeria innocua]
46906292        83        ESAT-6->Mem_prot->?->yukD*->S/TK->FtsK->                                                                                                                                         Listeria monocytogenes str. 4b F2365                           firmicutes                                    hypothetical protein LMOf2365_0070 [Listeria monocytogenes str. 4b F2365]
89203070        83        <-FtsK<-S/TK<-yukD*<-?<-Mem_prot<-ESAT-6                                                                                                                                         Bacillus cereus subsp. cytotoxis NVH 391-98                    firmicutes                                    conserved hypothetical protein [Bacillus cereus subsp. cytotoxis NVH 391-98]
49329053        83        ESAT-6->Mem_prot->?->yukD*->S/TK->FtsK->                                                                                                                                         Bacillus thuringiensis serovar konkukian str. 97-27            firmicutes                                    conserved hypothetical protein [Bacillus thuringiensis serovar konkukian str. 97-27]
13093361        503       <-FtsK<-?<-subtilisin<-Ub+12xTM*<-?<-FtsK<-memb_associated                                                                                                                       Mycobacterium leprae                                           actinobacteria                                probable membrane protein [Mycobacterium leprae]
41407608        503       memb_associated->FtsK-><-?||?->PPE_family->PPE_family->PE_family->ESAT-6->?->Ub+12xTM*->subtilisin->?->FtsK->PE_family->PPE_family->PPE_family->?->PPE_family->PPE_family->      Mycobacterium avium subsp. paratuberculosis K-10               actinobacteria                                hypothetical protein MAP1510 [Mycobacterium avium subsp. paratuberculosis K-10]
13881491        503       PPE_family->PE_family->PPE_family->?->PPE_family-><-?||PE_family->ESAT-6->?->Ub+12xTM*->subtilisin->?->FtsK->?->PPE_family-><-?||PPE_family->PPE_family->                        Mycobacterium tuberculosis CDC1551                             actinobacteria                                hypothetical protein MT1844 [Mycobacterium tuberculosis CDC1551]
31618574        503       PPE_family->PE_family->PPE_family->PPE_family->PE_family->ESAT-6->ESAT-6->?->Ub+12xTM*->subtilisin->?->FtsK->?->PPE_family->PPE_family->PPE_family-><-?<-PE_family               Mycobacterium bovis AF2122/97                                  actinobacteria                                CONSERVED HYPOTHETICAL MEMBRANE PROTEIN [Mycobacterium bovis AF2122/97]
76784314        481       PPE_family->PE_family->PPE_family->PPE_family->PE_family->ESAT-6->ESAT-6->?->Ub+12xTM*->subtilisin->?->FtsK->?->PPE_family->PPE_family->PPE_family-><-?<-?||PE_family->          Mycobacterium tuberculosis F11                                 actinobacteria                                hypothetical protein MtubF_01001866 [Mycobacterium tuberculosis F11]
41406262        509       PE_family->?-><-?||?->?->?->FtsK->Ub+12xTM*->subtilisin->?->FtsK-><-?<-?||?-><-FtsK                                                                                              Mycobacterium avium subsp. paratuberculosis K-10               actinobacteria                                hypothetical protein MAP0164 [Mycobacterium avium subsp. paratuberculosis K-10]
1944601         509       <-subtilisin<-FtsK<-?<-subtilisin<-?*<-FtsK<-?<-?<-?<-?<-PE_family<-FtsK<-memb_associated                                                                                        Mycobacterium tuberculosis H37Rv                               actinobacteria                                PROBABLE CONSERVED TRANSMEMBRANE PROTEIN [Mycobacterium tuberculosis H37Rv]
31620222        467       <-ESAT-6<-ESAT-6<-?<-FtsK||Ub+12xTM*->subtilisin-><-memb_associated||cutinase->cutinase->                                                                                        Mycobacterium bovis AF2122/97                                  actinobacteria                                PROBABLE CONSERVED INTEGRAL MEMBRANE PROTEIN [Mycobacterium bovis AF2122/97]
13883386        467       <-ESAT-6<-ESAT-6<-?<-FtsK||Ub+12xTM*->subtilisin-><-memb_associated||cutinase->cutinase->                                                                                        Mycobacterium tuberculosis CDC1551                             actinobacteria                                hypothetical protein MT3554 [Mycobacterium tuberculosis CDC1551]
41410338        452       <-cutinase<-cutinase||memb_associated-><-subtilisin<-Ub+12xTM*||FtsK->?->ESAT-6->ESAT-6->                                                                                        Mycobacterium avium subsp. paratuberculosis K-10               actinobacteria                                hypothetical protein MAP4240c [Mycobacterium avium subsp. paratuberculosis K-10]
92916372        475       <-ESAT-6<-ESAT-6<-?<-FtsK||Ub+12xTM*->subtilisin-><-memb_associated                                                                                                              Mycobacterium sp. KMS                                          actinobacteria                                conserved hypothetical protein [Mycobacterium sp. KMS]
92911534        475       <-ESAT-6<-ESAT-6<-?<-FtsK||Ub+12xTM*->subtilisin-><-memb_associated||?-><-?||?->cutinase->cutinase->                                                                             Mycobacterium sp. JLS                                          actinobacteria                                conserved hypothetical protein [Mycobacterium sp. JLS]
89338189        434       <-ESAT-6<-ESAT-6<-?<-FtsK||Ub+12xTM*->subtilisin-><-memb_associated<-?||?->cutinase->cutinase->cutinase->                                                                        Mycobacterium flavescens PYR-GCK                               actinobacteria                                conserved hypothetical protein [Mycobacterium flavescens PYR-GCK]
90203437        447       Ub+12xTM*->subtilisin-><-memb_associated<-?||?->cutinase->cutinase->cutinase->                                                                                                   Mycobacterium vanbaalenii PYR-1                                actinobacteria                                conserved hypothetical protein [Mycobacterium vanbaalenii PYR-1]
92917561        472       FtsK->memb_associated->FtsK->PE_family->PPE_family->ESAT-6->ESAT-6-><-?<-subtilisin<-Ub+12xTM*<-FtsK<-DNA_binding                                                                Mycobacterium sp. KMS                                          actinobacteria                                Protein of unknown function DUF571 [Mycobacterium sp. KMS]
13093791        485       <-subtilisin<-Ub+12xTM*<-DNA_binding<-ESAT-6<-ESAT-6<-PE_family<-FtsK<-memb_associated<-FtsK                                                                                     Mycobacterium leprae                                           actinobacteria                                conserved membrane protein [Mycobacterium leprae]
31617055        472       FtsK->memb_associated->FtsK->PE_family->PPE_family->ESAT-6->ESAT-6->DNA_binding->Ub+12xTM*->subtilisin->                                                                         Mycobacterium bovis AF2122/97                                  actinobacteria                                PROBABLE CONSERVED TRANSMEMBRANE PROTEIN [Mycobacterium bovis AF2122/97]
13879797        472       FtsK->memb_associated->FtsK->PE_family->PPE_family->ESAT-6->ESAT-6->DNA_binding->Ub+12xTM*->subtilisin->                                                                         Mycobacterium tuberculosis CDC1551                             actinobacteria                                hypothetical protein MT0303 [Mycobacterium tuberculosis CDC1551]
41409884        480       FtsK->memb_associated->FtsK->PE_family->PPE_family->ESAT-6->ESAT-6->DNA_binding->Ub+12xTM*->subtilisin->                                                                         Mycobacterium avium subsp. paratuberculosis K-10               actinobacteria                                hypothetical protein MAP3786 [Mycobacterium avium subsp. paratuberculosis K-10]
92910002        476       <-subtilisin<-Ub+12xTM*<-DNA_binding<-ESAT-6<-ESAT-6<-PPE_family<-PE_family<-FtsK<-memb_associated<-FtsK                                                                         Mycobacterium sp. JLS                                          actinobacteria                                Protein of unknown function DUF571 [Mycobacterium sp. JLS]
92915201        476       <-subtilisin<-Ub+12xTM*<-DNA_binding<-ESAT-6<-ESAT-6<-PPE_family<-PE_family<-FtsK<-memb_associated<-FtsK                                                                         Mycobacterium sp. KMS                                          actinobacteria                                Protein of unknown function DUF571 [Mycobacterium sp. KMS]
89343513        532       <-subtilisin<-Ub+12xTM*<-DNA_binding<-ESAT-6<-ESAT-6<-PPE_family                                                                                                                 Mycobacterium flavescens PYR-GCK                               actinobacteria                                Protein of unknown function DUF571 [Mycobacterium flavescens PYR-GCK]
90205295        533       <-subtilisin<-Ub+12xTM*<-DNA_binding<-ESAT-6<-ESAT-6||?-><-PPE_family<-PE_family<-FtsK<-memb_associated<-FtsK                                                                    Mycobacterium vanbaalenii PYR-1                                actinobacteria                                Protein of unknown function DUF571 [Mycobacterium vanbaalenii PYR-1]
92917997        505       <-subtilisin<-Ub+12xTM*<-FtsK<-memb_associated<-?<-ESAT-6<-?<-PPE_family<-PE_family<-FtsK                                                                                        Mycobacterium sp. KMS                                          actinobacteria                                Protein of unknown function DUF571 [Mycobacterium sp. KMS]
89338337        473       <-subtilisin<-Ub+12xTM*<-FtsK<-ESAT-6<-ESAT-6<-PPE_family<-PE_family<-FtsK<-memb_associated<-FtsK                                                                                Mycobacterium flavescens PYR-GCK                               actinobacteria                                hypothetical protein MflvDRAFT_5459 [Mycobacterium flavescens PYR-GCK]
13092444        512       subtilisin->?->?-><-Ub+12xTM*<-FtsK<-ESAT-6<-ESAT-6<-PPE_family<-FtsK<-FtsK<-memb_associated<-FtsK                                                                               Mycobacterium leprae                                           actinobacteria                                putative membrane protein [Mycobacterium leprae]
2370277         480       subtilisin->?->?->?->?-><-Ub+12xTM*<-FtsK<-ESAT-6<-ESAT-6<-PPE_family<-FtsK<-FtsK<-memb_associated<-FtsK                                                                         Mycobacterium leprae                                           actinobacteria                                hypothetical protein [Mycobacterium leprae]
90202132        508       ESAT-6->?->?->?->?-><-?<-?<-Ub+12xTM*<-FtsK<-ESAT-6<-?<-?<-PE_family<-FtsK<-FtsK<-memb_associated<-FtsK                                                                          Mycobacterium vanbaalenii PYR-1                                actinobacteria                                Protein of unknown function DUF571 [Mycobacterium vanbaalenii PYR-1]
89340379        549       FtsK->memb_associated->FtsK->FtsK->PE_family->PPE_family->ESAT-6->ESAT-6->FtsK->Ub+12xTM*->?->?-><-?<-?<-?<-?<-?<-?<-subtilisin                                                  Mycobacterium flavescens PYR-GCK                               actinobacteria                                Protein of unknown function DUF571 [Mycobacterium flavescens PYR-GCK]
92915077        509       FtsK->memb_associated->FtsK->FtsK->PE_family->PPE_family->ESAT-6->ESAT-6->FtsK->Ub+12xTM*->?-><-?<-?<-?<-subtilisin                                                              Mycobacterium sp. KMS                                          actinobacteria                                Protein of unknown function DUF571 [Mycobacterium sp. KMS]
92909344        509       FtsK->memb_associated->FtsK->FtsK->PE_family->PPE_family->ESAT-6->ESAT-6->FtsK->Ub+12xTM*->?-><-?<-?<-?<-subtilisin                                                              Mycobacterium sp. JLS                                          actinobacteria                                Protein of unknown function DUF571 [Mycobacterium sp. JLS]
2960229         511       FtsK->memb_associated->FtsK->FtsK->PE_family->PPE_family->ESAT-6->ESAT-6->FtsK->?*->?-><-?<-?<-?<-?<-subtilisin<-FtsK<-?<-subtilisin                                             Mycobacterium tuberculosis H37Rv                               actinobacteria                                PROBABLE CONSERVED TRANSMEMBRANE PROTEIN [Mycobacterium tuberculosis H37Rv]
81252663        487       FtsK->memb_associated->FtsK->FtsK->PE_family->PPE_family->ESAT-6->ESAT-6->FtsK->Ub+12xTM*->?-><-?<-?<-?<-?<-?<-subtilisin<-FtsK                                                  Mycobacterium tuberculosis C                                   actinobacteria                                COG0477: Permeases of the major facilitator superfamily [Mycobacterium tuberculosis C]
54014302        493       DNA_binding->ESAT-6->ESAT-6-><-?||memb_associated-><-FtsK||Ub+12xTM*->subtilisin->FtsK->?-><-FtsK                                                                                Nocardia farcinica IFM 10152                                   actinobacteria                                hypothetical protein [Nocardia farcinica IFM 10152]
54014325        488       memb_associated-><-?<-subtilisin<-Ub+12xTM*||FtsK->?-><-ESAT-6<-ESAT-6                                                                                                           Nocardia farcinica IFM 10152                                   actinobacteria                                hypothetical protein [Nocardia farcinica IFM 10152]
68264440        383       <-ESAT-6<-ESAT-6<-?<-FtsK||Ub+12xTM*->?-><-memb_associated                                                                                                                       Corynebacterium jeikeium K411                                  actinobacteria                                putative membrane protein [Corynebacterium jeikeium K411]
84494284        443       <-FtsK||?-><-?<-?<-?<-ESAT-6<-ESAT-6||Ub+12xTM*->                                                                                                                                Janibacter sp. HTCC2649                                        actinobacteria                                putative integral membrane protein [Janibacter sp. HTCC2649]
29831983        451       Ub+12xTM*->?->FtsK->                                                                                                                                                             Streptomyces avermitilis MA-4680                               actinobacteria                                hypothetical protein SAV5440 [Streptomyces avermitilis MA-4680]
71369935        451       <-Ub+12xTM*<-ESAT-6<-ESAT-6                                                                                                                                                      Nocardioides sp. JS614                                         actinobacteria                                hypothetical protein NocaDRAFT_4675 [Nocardioides sp. JS614]
21224082        491       ESAT-6->?->?-><-?<-?<-?<-?<-?<-?<-FtsK||Ub+12xTM*->                                                                                                                              Streptomyces coelicolor A3(2)                                  actinobacteria                                integral membrane protein [Streptomyces coelicolor A3(2)]
29829069        502       <-Ub+12xTM*||FtsK->?->?-><-?<-ESAT-6<-ESAT-6<-?<-ESAT-6                                                                                                                          Streptomyces avermitilis MA-4680                               actinobacteria                                hypothetical protein SAV2527 [Streptomyces avermitilis MA-4680]


---------------------------------------------------------------------------------------------------------------------------------------------------------------------------------------

II. Comprehensive alignments of different protein families described in the study

1. ThiS/MoaD/Ubiquitin

FINAL                                                  ---EEEE------------------E-----HHHHHHHHHH----------------------EEEEEE-----------------EE------EEEEEEEE---
ALIGN                                                  -----EEE---------------EEE-------HHHHHHHH----------------------HHEEEE-----------------EE-------EEEEE-----
HMM                                                    ---EEEEE---------------EEEE------HHHHHHHH----------------------EEEEEEE----------------EEE-----EEEEEEE----
FREQ                                                   ---EEEE-----------------------HHHHHHHHHHH----------------------EEEEEE----E-------------E-------EEEEEEE---
PSSM                                                   ---------------------------------HHHHHHHH----------------------EEEEE--------------------------EEEEEEE----

FINAL                                                  --EEE-------------------EEE----HHHHHHHHHH----------------------EEEEEE-----EE------E--EEEE----EEEEEEEE---\ThiS
2633522  Bacillus_subtilis_subsp_subtilis_str_168      -MLQLNG---------------KDVKWKKDTGTIQDLLASYQLE-----------------NKIVIVERN--KEIIGKERYHE--VELCDRD-VIEIVHFVGGG|
67939265 Chlorobium_phaeobacteroides_BS1               ITITLNG----------------QQREIQEGSTVEDILSIIGAE-----------------KQRVAVVVN--ENIVYPEKRGS--VLLREKD-QVEVLSFVAGG|
13879933 Mycobacterium_tuberculosis_CDC1551            MIVVVNE----------------QQVEVDEQTTIAALLDSLGFG-----------------DRGIAVALN--FSVLPRSDWATKICELRKPV-RLEVVTAVQGG|
29609756 Streptomyces_avermitilis_MA_4680              MNISVNG----------------ERRRIAPGTALDTLVKTLTAA-----------------PSGVAAALN--ETVVPRAQWSS--TALSEGD-RVEVLTAVQGG|
57865488 Staphylococcus_epidermidis_RP62A              MKCIING----------------DLFTFDQNQSIQEVLHSLELD-----------------PKRVIVELN--KELIKQDKYEE--YTVREDD-RLELLEIVGGG|
56909742 Bacillus_clausii_KSM_K16                      MRLVVNG----------------EERIS-ESTTLSELVSEFGLA-----------------SQLVVAEVN--GTIIDRVDWEA--TSLSEGM-KIELVHFVGGG|
17130691 Nostoc_sp_PCC_7120                            ITLQVNG----------------ETHNCSSPTPLPDLLQQLGFN-----------------PRLVAVEYN--GEILHRQFWEQ--TQVQSGD-RLEVVTIVGGG|
30138190 Nitrosomonas_europaea_ATCC_19718              MQLIING----------------QQQSYDGPMNVQQLVEKLSLQ-----------------NKRFAIERN--GEIIPRSRFPE--LLLNEGD-QLEIIVAVGGG/
FINAL                                                  -HHHHHHHHHHHHHH--------EEEE---HHHHHHHHHHHHHH--HHHHHHHH-------HHHHHHH-------------------------EEEEE------\MoaB
52696120 Pyrococcus_furiosus                           VKVKVKYFARFRQLAG----VDEEEIELPEGARVRDLIEEIKKRHEKFKEEVFGEGYDE--DADVNIAVN--GRYVSWD------EELKDGD-VVGVFPPVSGG|
10640172 Thermoplasma_acidophilum                      -MVTVRYYATLRPI------TKKKEETFNGISKISELLERLKVEYGSEFTKQMYDGNNL--FKNVIILVN--GNNITSMKGLD--TEIKDDD-KIDLFPPVAGG|
19915596 Methanosarcina_acetivorans_C2A                MKIHVKFLATIREITG----KPEIELEILPGDTVGTALQALQARYGPEFKEATTGTTAGG-IPKVRFLVN--GRNTDFLDGFE--TELKAGD-VMVFVPPVAGG|
11499216 Archaeoglobus_fulgidus_DSM_4304               -MVRVKLFANFRE-------AAGVKEVEVEAGTVGEVLQELVRRFPKLESLFYEEGRL---RDYVNIMVN--GRNVRGDLN----YPLSHTD-EVAIFPPVSGG|
75855800 Vibrio_sp_Ex25                                -MIKVLFFAQTRELI-----GIDSVELDDQFETVEAIRAHLVEEGADKNGKWDLALE----PGKLLAAVN--QSIVPLD------TEVKAGD-EVAFFPPVTGG|
26107155 Escherichia_coli_CFT073                       RMINVLFFAQVRELV-----GTDATEVAADFPTVEALRQHLAAQSDRWALALE--------DGKLLAAVN--QTLVSFD------HSLTDGD-EVAFFPPVTGG|
28868459 Pseudomonas_syringae_pv_tomato_str_DC3000     MKIEVQYFARYRETL-----GIDSESVEGEFVTLEVLRQHLLQRGEAWQVLA---------EQNLMCARN--QELCKLD------EPLLDGD-EVAFFPPVTGG|
4262375  Mus_musculus                                  CQIDVLYFAKSAEIAG----VRSETISVPQEIKASELWKELEMLHPGLADV----------RNQVIFAVR--QEYVELGDQQ---LLLQPGD-EVAIIPPISGG|
30681325 Arabidopsis_thaliana                          VEIKVLLFARARELTG----VPDLTLKMPSGSTTQKCLDELVLKFPSLEEV----------RSCVVLALN--EEYTTDS------AIVQHRD-ELAIIPPISGG/
FINAL                                                  -EEEEEE----------------EEEEE------HHHHHHHHHH--------------------EEEE------EEE---HHHH-HHHH-----EEEE------\Urm1
40889046 Mus_musculus                                  VSFKITLTSDP---------RLPYKVLSVPESTPFTAVLKFAAEEFKVP------------AATSAIITND-GIGINPAQTAGN-VFLKHGS-ELRIIPRDRVG|
71074940 Giardia_lamblia_ATCC_50                       IQVKIYKGFDP---------FYTYHVFNIPEASSTEKVIRLAARAFEIP------------QLEAVLINST-GDAIVPCQTILD-TCRRFGT-TLTVAHLKPII|
68352771 Theileria_parva                               VTFKIVLASDA---------NQPYKVLSVPEQAPFSAVIKFAAEEFRLN------------PATCAIITND-GVGINPTQTAGG-VFLKYGS-NLRLIPRDRVG|
15217447 Arabidopsis_thaliana                          VSFKVTLTSDP---------KLPFKVFSVPEGAPFTAVLKFAAEEFKVP------------PQTSAIITND-GIGINPQQSAGN-VFLKHGS-ELRLIPRDRVG|
72005426 Strongylocentrotus_purp                       VTFKITLTSDP---------KLPFKVLSVPESTPFTAVLKFAAEEFRVP------------AATSAIITND-GIGINPAQSAGN-VFLKHGS-ELRLIPRDRVG|
56112391 Chlamydomonas_incerta                         VTFKVTLTSDP---------KLPFRVFSVPEEAPFTAVLKFAAEEFKVP------------AQTSAIITND-GVGINPQQTAGN-VFLKHGS-ELRLIPRDRVG|
289769   Caenorhabditis_elegans                        VTFKITLTSDP---------KLPFKVLSVPESTPFTAVLKFAAEEFKVP------------AATSAIITND-GVGVNPAQPAGN-IFLKHGS-ELRLIPRDRVG/
FINAL                                                  --EEEEEE------------EEEEEEE----HHHHHHHHHHHHHH----------------------EEEE-EE--------------------EEEE------\RnfH
56312934 Azoarcus_sp_EbN1                              MKIGVAYSEPSH--------QVWLNLEVPDGTTVGAAIERSGILAQFPHID----------LTVQKVGVF--AKVVKLD------TPLRHGD-RVEIYRPITCD|
77389630 Rhodobacter_sphaeroides_241                   MIVGVAYAKPTV--------QVWKHVDVPEGTSAREAIERSGLLAQFPEID----------LAVNKVGIF--GAICPLD------RTLAEGD-RVEIYRPIHPE|
66047427 Pseudomonas_syringae_pv_syringae_B728a        IQIEVVYASVQR--------QVLKTVDVPTGSSVRQALALSGIDKEFPELD----------LSQCAVGIF--GKVVTDPAA----RVLEAGE-RIEIYRLLVAD|
67549235 Burkholderia_vietnamiensis_G4                 LSIEVCYALPDR--------QTLIPVSLPEGATVRAAIDASGVLALHPEID----------LAQAKTGVF--GKLAPLD------APLADHD-RVEIYRPLIVD|
68245723 Magnetococcus_sp_MC_1                         MRVAVTYAQPNR--------QLLLEFEVPEGTTAQQAVERSGILSKFPDIN----------LAEQKLGIY--AKLVEND------QVLEEGD-RVEIYRPAKGK|
71846749 Dechloromonas_aromatica_RCB                   MQIGVAYSEPSQ--------QIWLNIEVPDESSVKEAIERSGILKQFPHID----------LSTQKVGVF--GRLVKLD------AALKPGD-RIEIYRGIIAD|
59712607 Vibrio_fischeri_ES114                         IHVEVVYALPTE--------QVVFKLAVKAEQTVEEIIVQSGVLERYPEID----------LKVNKVGVF--SRNVKLD------STIRDKD-RIEIYRPLLAD/
FINAL                                                  --EEEEE-----------------EEE-------HHHEEE----------------------EEEEEEE----EE------------------EEEEEE-----\TGS
5107656  Escherichia_coli                              -MPVITL-------------PDGSQRHYDHAVSPMDVALDIGPGLA---------------KACIAGRVN--GELVDAC------DLIENDA-QLSIITAKDEE|
730881   Saccharomyces_cerevisiae                      VPLKIVLK------------DGAVKEATSWETTPMDIAKGISKSLA---------------DRLCISKVN--GQLWDLD------RPFEGEA-NEEIKLELLDF|
135177   Homo_sapiens                                  KPIKVTLP------------DGKQVDAESWKTTPYQIACGISQGLA---------------DNTVIAKVN--NVVWDLD------RPLEEDC-TLELLKFEDEE|
2983390  Aquifex_aeolicus_VF5                          EEVFVFTP------------KG-DLVVLPKGSTPVDLAYKIHTEVG---------------NHCAGAKSN--GRIVPLN------YELKSGD-VVEIITNPNKS|
1710082  Shigella_flexneri                             DRVYVFTP------------KG-DVVDLPAGSTPLDFAYHIHSDVG---------------HRCIGAKIG--GRIVPFT------YQLQMGD-QIEIITQKQPN|
416555   Drosophila_melanogaster                       RLQRIYTKPKGQLPD-----YNSPVVLHNERTSIEDFCNKLHRSIAKEFKYAL--------VWGSSVKHQ--PQKVGIE------HVLNDED-VVQIVKKV---|
2120160  Methanocaldococcus_jannaschii                 GFIKIYLKPQGKKPD-----FDEPLIMR-RGATVKDVCEKLHKDFVRNFRYAQ--------VWGKSAKHP--GQRVGLD------HKLEDGD-ILTIVIKR---/
FINAL                                                  ---EEEEHHHHHH---------EEEE------HHHHHHHH----------------------EEEEEEE------------------------EEEEE------\DUF82 fusion
71915653 Thermobifida_fusca_YX                         ASITLRFDPTLRPLLAPRNRTDLLHVNHDPAASLSHVVESLGVPL----------------TEIGELRIN--GTTASPS------QHPQPGD-LIEVLTVPKPQ|
20520977 Streptomyces_coelicolor_A32                   PEIHVEFAPELHLFVPRARPTGVASAATDGVSTLGHLVESLGVPL----------------TEVGALLVD--GREVPPG------HIPAGGE-SVRVRPVRHPQ|
54016307 Nocardia_farcinica_IFM_10152                  SGIELRLYAELNDFLPPQDRQDALWRPVRPHQTVKDIVEAAGVPH----------------TEIDLLLVN--GESVGFE------HHPRPGD-RLAAYPMFESL|
4981308  Thermotoga_maritima_MSB8                      KIAFFRFFGRLNDFFRN--SERIKTHRFTGFQTVKDRIEALGVPH----------------VEVSLITLN--GKPVGFD------HMVEDGE-LFFVYPEFQNI|
76785598 Mycobacterium_tuberculosis_F11                GYVDVRAYAELNEFVELQARGLTVRRPFRSHQTVKDVLEAMGIPH----------------TEVDLILVN--GDPADFS------YRPVAGD-RIAAYPMFEAL|
68554875 Ralstonia_metallidurans_CH34                  VTATFRFYEELNDFLAPAQRRRDLSCPCARAATVKHMIEALGVPH----------------TEVELILVN--GESSPFE------RIVCDGD-RIAVYPKFESF|
67666690 Burkholderia_cenocepacia_HI2424               ATASLRVVVELNAFLASQQRDRAFAHACARDATVKHAIEALGVPH----------------TEIGRLYVN--DAPAALD------RPLDDGD-RVEVLPERAGP/
FINAL                                                  -HHHHHHEEEEEEE---------E---------HHHHHHHHH------------------------EEEE-----------------E---E-EEEEEEEEEE-\ub-like
71847777 Dechloromonas_aromatica RCB                   MTIAVNEIRRVFRY------NGVQLPD-VPGMEPKEVRDLYSAQY----------------PELISAEIE--A------------GDVVNGV-QEYTFRKAVGT|
67908730 Polaromonas_sp_JS666                          ILVSTTVLKRVFMS------NGNPLTDPDPSMSPAAVKDFWSAMY----------------PELLNAEVQ--G------------PVSKDGE-LTYTFHRTTGT|
84357757 Burkholderia_cenocepacia_PC184                --MEIETLAREFSY------NGAKLADPAPTFTLQQIRDFYSQTY----------------PELTNAEIE--G------------PVIKGNR-NVYTFRRAVGT|
17428677 Ralstonia_solanacearum                        --MQTIQLTREFRY------NGVRLADPSPQFTLEQVRDFYANTY----------------PEILNADID--G------------PSVEGTL-QVYGFRRAVGR|
29339958 Bacteroides_thetaiotaomicron_VPI_5482         MALDIKGLKRVFILKKGN--DTLTLEDPDSRMSLSEVTDFYSMNY----------------PELTTATLH--G------------PELEEDR-AIYRFKTTIGT|
71839548 Pelobacter_propionicus_DSM_2379               --MQITTLTRTFKY------NGATLRDPDPKQTPEQVKEFYSMAY----------------PELTTAVVE--G------------PEENNGQ-LQYSFRKGAGT|
38637971 Cupriavidus_necator                           MALEIKKLLRQFSY------NGMSFVDPGPAFTPEQVRDIYSAQY----------------PELTTASVD--G------------PEVKGEV-ASFTFVRAAGA|
84717440 Polaromonas_naphthalenivorans CJ2             MALIAKTISRTFKF------NGMTLADPSPEMDMETVKRFYANQY----------------PELLNSVVE--G------------PVTKGTV-STYTFIRAVGA/
FINAL                                                  ---EEEEE--H---------HHH-EEEEEEE--HHHHHHHHHHHHHHHHHHHH--------EEEEE-------HHHHHHHH------------EEEEEEEE---\TAPI
76556246 Phage_BP_4795                                 PLARICLHGDL---------QRFGRRLSLYVNTAAEAIRALSLQVPGFRRQMNEGW-----YQIRIAGDDT-APEAVYARLH---EPLGEGT-VIHIVPRLAGA|
215124   bacteriophage_lambda                          GMARICLYGDL---------QRFGRRIDLRVKTGAEAIRALATQLPAFRQKLSDGW-----YQVRIAGRDV-STSGLTAQLH---ETLPDGA-VIHIVPRVAGA|
11877308 Neisseria_meningitidis_phage_2120             -MITVCLYGGL---------REYGRRFVLHVETPAEALHALFTQIKGLRQRIRDGV-----YQVRFDGKDQ-SEETIGSV-----FRRPADG-VLHIVPRVQGA|
45686326 Enterobacteria_phage_T1                       DVKVIKLSGSLG--------RRFGVFHRYAVDSYPEAIRALSSQVDGFKEYMQSEVGSRSKFAIFVDGVNV-GHHEE--------EKFKCAK-EIRIVPIPTGS|
71834086 Bacteriophage_JK06                            NVIDVKLGLGLG--------RKFGKLHKLCVKTVPEAMRALSVNIPEFKEFMRSHVGQNTRFAVFVDGKNV-NEHKI--------NDLETVS-EIRIMPIPQGR|
46402106 Bacteriophage_phiKO2                          VMTRIELSGILG--------KKFGAYHERLVSTTSEGIQALCCTIDGFEKFLNNSKEKGLTFAIFKGKKNI-GKDDL--------GFPVNGD-VIRIVPVIIGS|
9634139  Enterobacteria_phage_HK022                    VMTRIELSGVLA--------KTYGRVHHRLVRTTAEAINALAKTINGFEKFLNTSKARGLTYAVYRDKKNI-GVDDL--------GFPVTGE-VIRIVPVVIGS|
17975181 Bacteriophage_phiE125                         TFRTIRLYGVLG--------GRFGRVHRLAVSSTAKAVRALSVLIPGFRAFLTSARDGGLTFAVFNGRRNL-GEDEL--------EHPVGRD-EIRIAPVIVGS|
77864688 Burkholderia_cepacia_phage_Bcep176            KLREVRLYGIAG--------TRFGRVHRLAVSSTAEAVRALSVLLPGFRKFLLEARDNGLTFAVFNGRRNL-SQDDL--------TAPVGDE-AIRIAPVIIGS/
FINAL                                                  --EEEEEE--------------EEEEEEE--HHHHHHHHHH-----------------------EEEEE----EEE----EE---EEE-----EEEEEEE----\TAPI+protein J
85716602 Nitrobacter_sp_Nb_311A                        PAATVSVYGTTHPLNAVA--GARIHCRVPAGWSITEILGEALSHKPGWHR-----------RRDLIVRIN--DHIIPEENWSR--VRVKQGA-TVTFIPRLQDG|
66392071 Xanthomonas_campestris_pv_pelargonii_phag     THQVIVSPHPVVVDD-----QKNLILAFKQGESLFEILSRSVDNFE---------------EREWVVTIN--GRRVPVEMWTK--AFPKPGH-IIEVR--GNVG|
33568295 Bordetella_bronchiseptica_RB50                MPALMVVHNPFVASEG----RKAYCAAFLPGETLGRYCERMGVALP---------------SRVVNVWHN--GRPVPLALWQR--LIPRQGD-QVVIRAKGEGG|
46449977 Desulfovibrio_vulgaris_subsp_vulgaris_str     KADVVSVTGCPHPFRP----GDRVHDVVPVGGTLESIVVRGLDDMGVPEAL----------RGCGHAFVD--GEYVPRDRWAD--VTPRAGS-TVTYRLVPAGG|
67545284 Burkholderia_vietnamiensis_G4                 QSAVVLLRNPFQP-------SQREVMVAHPTQTIRQWLGAQGIAEF---------------DQPTVCIKN--DAPVLRTDWAV--T-PIDG--VVLFITLPQGG|
23015894 Magnetospirillum_magnetotacticum_MS_1         TASVIIIANPFEPV------ASRSVHAIVAGVTVGELLLDCGIDPDRW-------------ADGPEIRIN--GNVVAAEIFAV--RVIGEDE-IISIIRWPLGG|
78033450 Magnetospirillum_gryphiswaldense              TASIVIVTNPFEPV------ASRSVHAVESGITLGGLLQACGIAEDCW-------------SDGPEILIG--GMTVPVGIYAV--RAIVDGE-VVTVIRWPQGG/
FINAL                                                  ----EEE-----------------EEE----HHHHHHHHH-------------------------EEEEE-------------------E------EEEEEE--\fusions to E1-like proteins
57168916 Campylobacter coli RM2228                     --MRIKFN--------------GKELDTKLSTSLDFFKSVSK-------------------NENDVWIIN--GFAT---------KENIKIH-ENDELFCIERN|
57166736 Campylobacter jejuni RM1221                   -MMRVKFN--------------GKELDTDFKTSLEFFENISK-------------------NENDVWIIN--GFAT---------KENIALN-EDDELFCIERN|
71837115 Pelobacter propionicus DSM 2379               -MIQIRLN--------------EKTIMVDDGLTLAMLAKQRR-------------------PGADVLILN--GFPA---------EDDTQIN-DGDAVFLIKRG|
77544308 Pelobacter carbinolicus DSM 2380              --MHIWIN--------------EQPHNISEDARLFEMRDRFK-------------------PQADVVILN--GFPV---------TSDRPLS-NGDRIVLIRRG|
68178158 Desulfuromonas acetoxidans DSM 684            --MIIVLN--------------ENKIQVEENQSLFDLRDQIK-------------------PEADVLICN--GLPI---------QSDRTLQ-PFDHVILIRRG|
18145265 Clostridium perfringens str. 13               --MNIKIN--------------EKWREVKENCTVYALKNEEF-------------------PDSHVIVLN--GFPL---------VEDKKLK-DGDRIVFIKKG|
28203841 Clostridium tetani E88                        --MKIYVN--------------EIFLNVEEDIDVFKLKNKIK-------------------KDADIVIYN--GFPI---------NNNIVLK-PLDRIVFIKRG|
77683437 Alkaliphilus metalliredigenes QYMF            --MKLIVN--------------EDEMDVKKGTTAFEVRNKVK-------------------KDADVVVYN--GFII---------KEDVLLQ-EGDLITLIQRG/
FINAL                                                  -EEEEE--------------EEEEEEEE-----HHHHHHHHH---------------------EEEEEEE---EEE-------------------EEEEEEEE-\TmoB
48094248 Pseudomonas_sp_OX1                            ATFPIMSNFERD--------FVIQLVPVDTEDTMDQVAEKCAYHSINRRVHPQP-------EKILRVRRHEDGTLFPRGMI----VSDAGLR-PTETLDIIFMD|
78693154 Bradyrhizobium_sp_BTAi1                       ALFPLQANFRGD--------FVVLLVPVDDGDTMSVVADKVAQHAVGLRVAE---------KNASKCVYHN-GKELPSAIT----VAQSGIQ-PMDWIEVAYV-|
68556036 Ralstonia_metallidurans_CH34                  ALFPLSSNFEGD--------FVLQLVAVDTENTMDEVAAAAAHHSVGRRVKARP-------GHILRVRQQGSKECLPRTMK----VADSGLK-PTECVEVIWEP|
45479222 Pseudomonas_mendocina                         SAFPVHAAFEKD--------FLVQLVVVDLNDSMDQVAEKVAYHCVNRRVAPR--------EGVMRVRKHRSTELFPRDMT----IAESGLN-PTEVIDVVFEE|
71849051 Dechloromonas_aromatica_RCB                   ALFPLTSNFEGD--------FVLQLVAVDSENTMDEVAAAAAHHSVGRRVRARP-------GQILRVRRQGGEEFLPRTMR----VSESGLK-PTETVEIIWEA|
86565792 Frankia_sp_CcI3                               ALLPLSAVFEHD--------FVSLLVAVDDADTVEVVGQKIAHHVVGRRLPAS--------DAPVGIRHN--GQVLAREAR----IGEAGVG-PLDHVEAFFDE|
72122837 Ralstonia_eutropha_JMP134                     ALFPVISNFQYD--------FVLQLVAVDTENSMDEVAAAAAHHSVGRRVAPQP-------GKVVRVRRQGGDQFYPRDAR----IGDTDIK-PMESLEFIFCD/
FINAL                                                  EEEEEE-----------------EEE-------HHHHHHHH---------------------EEEEEE------EEEE--HH---HHHH-------EEEEEE--\repeat
84711628 Polaromonas_naphthalenivorans_CJ2             VVADEQLN-------------DRHLDLRDPVPTGRQILQAAEVRPVA--------------DYSIYAILPS-GEFEDLRLDE---TYDLRGR-GAERFVIFQTD|#1
69928899 Nitrobacter_hamburgensis_X14                  EVAGTDLA-------------FGPVIIRDRTPTGAQIAAAAGLTPAQ--------------DPYVLSFLPD-GELVEILASE---TVDLDE--GRRRFIVTSAD|
17134587 Nostoc_sp_PCC_7120                            KHYLVRID-------------DRSYKVDDPVITGGQLLDKASKRPVD--------------EYLIFQMLNN-GQLEEIRLDE---TVELRKP-GIERFITWRSD|
38423904 Synechocystis_sp_PCC_6803                     QQFRIQVD-------------QQQLMIPDPVPTGRQILEIAQKRPAD--------------EFLVFYLLPS-GQLEEIRLDE---TVDLRQT-GIERFITFRSD|
28806071 Vibrio_parahaemolyticus_RIMD_2210633          FFALDSLQ-------------FRSLSVQDPVPTGRQLIEIAGLDSFD--------------DYSLFAILPS-GDFEDIRLNE---TVDLRAR-GVERFIAFKTD|
68554444 Ralstonia_metallidurans_CH34                  ------LN-------------FIKIEIDDPVPLGRQVLTAAGMHGDD--------------NYSLFNILES-GDFEDVRLDE---QIDLRRP-GAERFIAFKSD|
77690161 Rhodopseudomonas_palustris_BisB5              RGMEYPVN-------------GAMAAFPDNVVNGREVLTRSGLVPAS--------------EYRLI-LVRN-GRTRLIGTDD---DVDLDKE-HGGSFRAFLSD|
39651045 Rhodopseudomonas_palustris_CGA009             LIADESFN-------------FRSFPFDDRQVTGAQIGEVFGAHPIS--------------DFVIIQQLES-LELETLRPTE---LADLRKS--VRFFV-IRGD|
14025878 Mesorhizobium_loti_MAFF303099                 TNFTFKLD-------------GRVVATNDAIISGREVRALGGLDPAS--------------DYILIQIADR-TS-RSIGLEE---AIDFREM-PHSEFLSFQGD|
77961668 Yersinia_mollaretii_ATCC_43969                LFAQENLA-------------FRAIEVNDPVPLGRQILIAAGLRAND--------------DYSLFAILET-GDFEDLRLDE---TFDLRGR-GAERFVAFQTD/
FINAL                                                  --EEEEEE---------------EEEE-----HHHHHHHH----------------------EEEEEEEE-----EEE--------EE-------EEEEE----\repeat
84711628 Polaromonas_naphthalenivorans_CJ2             RAFKFTID-------------DRQMEWGKPSISGKILKVLAGVPTD---------------TYDVYLEVRS-GGQDVLIRDTD--LIDLSKP-GIERFITLIRD|#2
69928899 Nitrobacter_hamburgensis_X14                  RSYRLTVD-------------GEQYDWPARMVTGATVRKLARVPAE---------------FL-VYLERQD-EPDRLIGNQD---IVNLGDK-GVEHFHARKQT|
67547440 Burkholderia_vietnamiensis_G4                 --YKIRID-------------KDYYVVDVPHMTGEQILGLAGKTSA---------------GY-LLSEKVH-GQMRPVAPAQ---TVDFTAH-GVERFATIPKE|
17134587 Nostoc_sp_PCC_7120                            RSFRFVID-------------GRRFEWGAPIITGLKLKELAGVDLA---------------SYGVWLELRG-AEDRPIADNE---SVDLQAP-GVERFFTGKKT|
38423904 Synechocystis_sp_PCC_6803                     RSFRFVID-------------GRRFEWGIPLISGLKLKQLAQVSPQ---------------AYGVWLEVRG-GEDRPIADHE---TVNLEAP-GVERFFTGKKT|
28806071 Vibrio_parahaemolyticus_RIMD_2210633          RDFKFSLK-------------GRQIVWGKSEIDGSDLYFLADV-AD---------------EQAIFLDVRG-GTDRLIEPDD---TVDLSEA-GIEHFVVADKP|
68554444 Ralstonia_metallidurans_CH34                  RNFKLTVN-------------GSQVVWGRPTISGADLYALSKP-AD---------------GEAVFMVVSG-GEDRQIERED---DVDLAAP-GVERFENAPKR|
77690161 Rhodopseudomonas_palustris_BisB5              RDFGFTVD-------------EVGQVWGTADMEVDEFLRIWPQHPE---------------HR-WVLERDD-EPDTVLTPGG---VLSFGPK-GVEHVVSRKDA|
39651045 Rhodopseudomonas_palustris_CGA009             ATYTFIVD-------------GLTMVWPKKTITGKAVKMLTNKDED---------------DIEVLLERED-RPDKVIGDDD---DIQLAAD-GVEKLKTRYAK|
14025878 Mesorhizobium_loti_MAFF303099                 RAFSFTVN-------------ERGWEWGSATISAADIYRYASIDED---------------LE-LIL--DS-AGDTVIPADG---AVTLGGQ-GVERIRSREAK/
FINAL                                                  -EEEEEEE------------------------HHHHHHEE---------------------EEEEEEEEE-----------------EEEEE--EEEEEEE---\repeat
14025878 Mesorhizobium_loti_MAFF303099                 KTVVIKVN-------------GRSRTVPRRKHSYREIALLAYPDA-NFEK-----------FKYTITYLKG-VHGA-EGDLVE--GENIEVKNGMVFNVRRSDK|#3
28806071 Vibrio_parahaemolyticus_RIMD_2210633          PDYIITVN-------------SREHVLDDPNVTYEQIVSFEFQYPPSNPN-----------TCYSMTYRHA-KSKPHAGELAAG-GSVIVKKKGTVFNVTATDK|
68554444 Ralstonia_metallidurans_CH34                  PKVVIIVN-------------GTKEELPAPLVTFDQLVALAYPGQPPQPG-----------ITYSITYYKV-ASYPHQGPMAP--GGSVEAKNGSIFNVGRTIQ|
39651045 Rhodopseudomonas_palustris_CGA009             TTVTIIVE-------------GTPHKWDKKKISYAEVVTLEVSDYEHHPD-----------ITYSVNFTNG-PHNRPEGDLAK--GESVKVRDGMIFSVSETGQ|
88795473 Alteromonas_macleodii_Deep_ecotype            KIFEIIVN-------------GRMKSVEDKFLTFVEIVKLAFGEFKECQN-----------QIYTMTFKRG-VGKK-EGSLVL--GDKVRIKDGVIFNVTATNK|
86566459 Frankia_sp_CcI3                               KTVEIIVN-------------GRRRTVVKGELSFDEVVALAFDPVPAGDN-----------VDFTITFRRG-HGDKPEGTLRP--GGTVKIKEGMIFDVTATDR|
69928899 Nitrobacter_hamburgensis_X14                  QNVLIEIA--------------TPTVVVAD--AMRQAGFDPAQPWHIFLKVQDQ-------TKREVAANYV-LDLRTPGIEKLR-LIPKDVNNGEACAPR----/
consensus/100%                                         ........................................................................................................
consensus/95%                                          ....................................h............................................................h......
consensus/90%                                          ....h...........................s...h............................................................h......
consensus/85%                                          ..h.h...........................s...h................................p...........................h......
consensus/80%                                          ..h.h...........................o..ph...h.........................h..ps.......................h..h......
consensus/75%                                          ..h.l.h.........................o..phh..h.........................h..ps....................p..hp.h....ss
consensus/70%                                          ..h.l.h.........................o..phhp.hs........................h..ss....................p..lp.h....ss

---------------------------------------------------------------------------------------------------------------------------------------------------------------------------------------

2. UBC/E2 like domain
                                                         Helix-1         Str-1                Str-2                Str-3                             Str-4  |                                  *                  *                                     Helix-2                                        Helix-3       Helix-4
Secondary Structure                              -hHHHHHHHHHHHHHh--------EEEEE----------------EEEEEEE--------------EEEEEEE---------------------------EEEE-----------------------------------------------------------HHH-------------------------------HHHHHHHHHHHHH-----------------------------------HHHHHHHH--h---hhhHHHHhhHHH
1ayzA_Ubc2_Scer_3659954                          TPARRRLMRDFKRMKE---DAPPGVSASP----------LPDNVMVWNAMII----GPADTPYEDGTFRLLLE------------FDEEYPNKPP-----HVKFLSE---------------------------------MFHPNVYAN---------GEICLDILQ----------------NRWTP------TYDVASILTSIQSLFN---------------DPNPASPAN-----------VEAATLFKDHK---SQYVKRVKETVE
1Q34A_Ubc_Cele_34810893                          TPSRRRLMRDFKKLQE---DPPAGVSGAP----------TEDNILTWEAIIF----GPQETPFEDGTFKLSLE------------FTEEYPNKPP-----TVKFISK---------------------------------MFHPNVYAD---------GSICLDILQ----------------NRWSP------TYDVAAILTSIQSLLD---------------EPNPNSPAN-----------SLAAQLYQENR---REYEKRVQQIVE
2E2C_E2-C_Ssol_4388942                           HSVSKRLQQELRTLLM---SGDPGITAFP----------DGDNLFKWVATLD----GPKDTVYESLKYKLTLE------------FPSDYPYKPP-----VVKFTTP---------------------------------CWHPNVDQS---------GNICLDILK----------------ENWTA------SYDVRTILLSLQSLLG---------------EPN-NASPL-----------NAQAADMWSNQ---TEYKKVLHEKYK
1QCQA_Ubc4_Scer_5107650                          MSSSKRIAKELSDLER---DPPTSCSAGP----------VGDDLYHWQASIM----GPADSPYAGGVFFLSIH------------FPTDYPFKPP-----KISFTTK---------------------------------IYHPNINAN---------GNICLDILK----------------DQWSP------ALTLSKVLLSICSLLT---------------DANPDDPLV-----------PEIAHIYKTDR---PKYEATAREWTK
2AAK_Ubc1_Atha_2981894                           TPARKRLMRDFKRLQQ---DPPAGISGAP----------QDNNIMLWNAVIF----GPDDTPWDGGTFKLSLQ------------FSEDYPNKPP-----TVRFVSR---------------------------------MFHPNIYAD---------GSICLDILQ----------------NQWSP------IYDVAAILTSIQSLLC---------------DPNPNSPAN-----------SEAARMYSESK---REYNRRVRDVVE
1PZVA_Ubc_Cele_34811307                          EQSSLLLKKQLADMRR---VPVDGFSAGL---------VDDNDIYKWEVLVI----GPPDTLYEGGFFKAILD------------FPRDYPQKPP-----KMKFISE---------------------------------IWHPNIDKE---------GNVCISILH---------DPPEEEEERWLP------VHTVETILLSVISMLT---------------DPNFESPAN-----------VDAAKMQRENY---AEFKKKVAQCVR
1I7KA_Ubch10_Hsap_13786748                       GPVGKRLQQELMTLMM---SGDKGISAFP----------ESDNLFKWVGTIH----GAAGTVYEDLRYKLSLE------------FPSGYPYNAP-----TVKFLTP---------------------------------CYHPNVDTQ---------GNICLDILK----------------EKWSA------LYDVRTILLSIQSLLG---------------EPN-IDSPL-----------NTHAAELWKNP---TAFKKYLQETYS
2UCZ_Ubc7_Scer_2981900                           KTAQKRLLKELQQLIK---DSPPGIVAGP---------KSENNIFIWDCLIQ----GPPDTPYADGVFNAKLE------------FPKDYPLSPP-----KLTFTPS---------------------------------ILHPNIYPN---------GEVCISILHSPGDDPNMYELAEEEEERWSP------VQSVEKILLSVMSMLS---------------EPNIESGAN-----------IDACILWRDNR---PEFERQVKLSIL
1J7DB_hUbc13_Hsap_15825811                       AGLPRRIIKETQRLLA---EPVPGIKAEP----------DESNARYFHVVIA----GPQDSPFEGGTFKLELF------------LPEEYPMAAP-----KVRFMTK---------------------------------IYHPNVDKL---------GRICLDILK----------------DKWSP------ALQIRTVLLSIQALLS---------------APNPDDPLA-----------NDVAEQWKTNE---AQAIETARAWTR
1JASA_Hsubc2b_Hsap_34809571                      TPARRRLMRDFKRLQE---DPPVGVSGAP----------SENNIMQWNAVIF----GPEGTPFEDGTFKLVIE------------FSEEYPNKPP-----TVRFLSK---------------------------------MFHPNVYAD---------GSICLDILQ----------------NRWSP------TYDVSSILTSIQSLLD---------------EPNPNSPAN-----------SQAAQLYQENK---REYEKRVSAIVE
1KPSA_Ubc9_Hsap_20150955                         GIALSRLAQERKAWRK---DHPFGFVAVP-----TKNPDGTMNLMNWECAIP----GKKGTPWEGGLFKLRML------------FKDDYPSSPP-----KCKFEPP---------------------------------LFHPNVYPS---------GTVCLSILE-----------EDDDDKDWRP------AITIKQILLGIQELLN---------------EPNIQDPAQ-----------AEAYTIYCQNR---VEYEKRVRAQAK
1KPPA_Tsg101_Hsap_21465897                       YKYRDLTVRETVNVIT------LYKDLKPVLDSYVFNDGSSRELMNLTGTIP----VPYR--GNTYNIPICLW------------LLDTYPYNPP-----ICFVKPT----------------------------SSMTIKTGKHVDAN---------GKIYLPYLH-----------------EWKHP-----QSDLLGLIQVMIVVFG---------------DEPPVFSRP----I------SASYPPYQATG---PPNTSYMPGMPG
1JATA_Ubc13_Scer_14719686                        ASLPKRIIKETEKLVS---DPVPGITAEP----------HDDNLRYFQVTIE----GPEQSPYEDGIFELELY------------LPDDYPMEAP-----KVRFLTK---------------------------------IYHPNIDRL---------GRICLDVLK----------------TNWSP------ALQIRTVLLSIQALLA---------------SPNPNDPLA-----------NDVAEDWIKNE---QGAKAKAREWTK
FINAL                                            HHHHHHHHHHHHHHH------------------------------EEEEEEE----------E----EEEEEE----------------------------EEE--------------------EE--------------EE-----------------EEEEEE-------------------------------HHHHHHHHHHHHH-----------------------------------------HHHHH----HHHH--------
_Rsp._22726448                                   TAGEARLIRECEELAS---LAAASAWLEEP-----QFGKNADGLLTWSFVLL----------AGDRRIPLRLV------------FPALFPDLPP-----FVLPADS-----------------------------SVRLSQHQYGEG----------GELCLQYRP----------------DNWHP------DCKSADVVRSAKALLE---------------ATPKDDGFS------------DVESAHPTDL---PSLLSGCSRRFM
OB2597_05120_Obat_84499281                       LVDSARLAAERRSIEQ----AAAGEWFRFA------RWTLHHGLVCVEGEIL----------AHDNTYPVRLI------------YPDQFPLVPA-----WVEPAEK------------------------------ARWSSHQYSG-----------GSLCLELRP----------------DNWIP------TATGADVLESAFNLLH-----TEDPLGEGGATAPSDHRVG------------EVQTYGDLHL---PALIGAGCLDRL
RHE_CH01997_Retl_86357617                        LNNTVRVAREKEAVEN---LATETEWFVLD------RWEIHDYKFAAIGSIV----------AHGATYPIRLV------------YPDNFPLVPA-----WVEPQDP-----------------------------EAKWSYHQYGKG----------GALCLELRP----------------DNWTS------RANGADVLRSAYGLLN----LENPLGDGEKGKVTSAHNVG------------EIQKYNWGES---PVFIGQECLTRL
y4oA_Rsp._2496721                                RLTEVNVLKRGSDQDN---WWQAYPGLYAR-----ELAAYEGHGASHRPLIQ----------QDGTLILEVLWP-----------MDSAGSIRLN-----VGYSPLH-------------------PFCRPSISAPELQLERHQNPFT----------RDLCLLTQDS---------------AQWYPH---QMVADFIAERLSQVLQVM-------------------T----------------LRRNEQWSEA---ASLEEQAPDPVT
y4qC_Rsp._2496738                                PAGRRRLAELQKLHSA------AGESLLVD-----EEAAAAGILRIEFSWPL----------NDGRTIGLRAV------------YPDTFPRLRP-----HVFLTCD----------------------------PSEYPERHCGSE-----------GALCLLGRDT---------------RYWQAN------MSLAELLDENLAHVL---------------DGT-------------------GAEDPQGEP---IEYWWNSLGQAS
ROS217_07909_Rsp._85706659                       RTAQDHSAHDFGVMDA---WERVREVLAGH-----GFTLVPGSGRDRYQGQI----------KVGSVPVSLEIE-----------IADYDFLDLP-----KVRVLKR--------------------------EALPKRLTGHIVSD-----------GTLCYADKAT---------------FLLDRY----QPDRSVVSCLEQARTTL---------------NTLLHG---------------NPSVAYMAEL---AAYWSATPYCL-
_Cper_86475968                                   -MVILILDLFNSLNSF---ENIKNVKEIKK-----NNDNFEVNYSKIYEFTL----------NIQKQNFDIIMC-----------IPEEWNLKLI-----DFYIKDY----------------------------KNIKFIPHLEEN-----------GKICLFDKEG---------------LLVEEN----LNGIAIESIERLNKVLY---------------EGLNDI----------------NKLDFINEF---DAYWNLLSTNNI
GuraDRAFT_0469_Gura_88937743                     DESLLKEALETCLLVK---SVAELHPKRLA-----EPWAKDRFVCRSYKLVI----------ELNGVPVDFYFG-----------VKKSFPLSLP-----YIFLAQW----------------------------DSFGILPHVETD-----------GYICYAQEDG---------------SVLDFD---DVAGIAQEALSRAIQVVV---------------DGISGK----------------NHQDFLDEF---GAYWDRLKKVKF
Psyc_1372_Parc_71038912                          -MMSELHQTMLSCGFK---YLKNSQRQSIS-----FFDSIPTTRPIYVKDYK----------TSEGIFNVALV------------FGDDLYTTLP-----RAQVLKK-------------------------PKKIEQVLLPHINSG-----------GYLCYVEEKE---------------ADWNPN----NLNALYRAVDEQVQNTL---------------NTAISSLQNG----------QIDQAEFEGEF---VSYWKPEQTIY-
ELI_04040_Elit_84786718                          -----FRFRMMSLADR---WRAIAATLANK-----GFTEQQGASPEFRGSIN----------VHGRAVDIELV------------IPDSKFVELP-----IVRLVDR--------------------------KQLPAGAFGHISRDDIEG-------SVVCFAPATG---------------LPLDFH----DPGGSVLRVLRQTELSL---------------EKSFAGQG---------------GAEVAAEY---QEYWIEKEPNFR
_Ecol_37927532                                   MKDGQLHQVMTGCGYR---YTRARNLPEKS-----ILHSRERGAGYYTKEYA----------TDAGNFNVALV------------IHPDPFTELP-----TAFIIEQ-------------------------PEQFKSCLMPHVALE-----------GFLCYVEQME---------------ADWDSN----DLEATYKEVDAQIHQTL---------------IDSVSAATQG----------VNDKRELEGEF---AAYWRPSETLFL
VC0180_Vcho_9654584                              -MKQELHHTLLGCGFR---YTPAKQMPKGI-----LLDTKSRRKGYYVKEYS----------TKGGVFVIALV------------LWNDPHIQLP-----FAYILQQ-------------------------PEQYKGRLLPHINFG-----------FCLCYVTQME---------------ADWNSN----DLKSTYQDVDEQIQLTL---------------DNSVASVESG----------TSNDVELEGEF---SAYWQSEEELYL
PB2503_00627_Pber_84701417                       GVISEARTALADRLGA---YLLSAFDAQPF-----SASDLQAYNGKKVDRGW----------RLPGDPPLHLL------------LDPEFPYAPP-----RIALPDE----------------------------TQRLLWPHVETA-----------GLLCVFPTQ----------------TNIDAF---EPEKVATALITDARDLIT---------------RNQSGD----------------LDEEFRKEF---QSYWTLAIDDKA
Shewana3DRAFT_3199_Ssp._78684828                 ------LERHRGHSVL---SEIKQHLINQG-----FNCTTSEVAGGERIVVE----------TTILNHGIQLML-----------VADPPYYRLP-----EFFLINP----------------------------DSIGRLAHVSVHEYAGIQI----GTVCVNAPES---------------LSVNFE---QPLLVVEESLRRHILLLE---------------KCITNPD--------------WNHSELLREF---SSEWLRICAPDS
ArthDRAFT_2172_Asp._66965723                     WERYAGLLQSEISWLQ---DLGIACRIDET-----KRDDHQTLTMELSVPET----------VTGTAPLELTAV-----------FPDFYPLVPP-----KVFAVDL--------------------------------GMPHHWNPFS---------NEVCLLGTPS---------------EEWGTN------GSLAQLLKDQLPAAL---------------KAGMSGDEH----------ADWNEKPQAEPF---GAYYNSYANSAM
FINAL                                            HHHHHHHHHHH----------EEE-------------------EEEEEEEE---------------EEEEEEEE--------EEE---------------------------------------------------------------------------EEEEE-HH---------------HHH------HHHHHHHHHHHHHHHHHH-------------------------------------------------------------
Mdeg02000735_Mdeg_48864353                       IHDVIRWLDETRSVAG--IQTVTSSDDGV------------VVATNWRVDLP--IRFESEGETESGIRSIEAV---------SWVFPWEYPLRAP-QPKLREDFPLT---------------------------------LPHINPVVEGED------ISPCIAEVDL-----------TDLLHSSGI-----EAVFGAMTHWLNNAASGEL-------------LCPVQGWE------------PVRRDNASGLI---SADTYAIREELN
SYN_01833_Saci_85859492                          AQEELREIEAASEGAF--EVLSVRFPEGD------------HRSAIAEISVT--CFDMPYAEGGIKLRDRERF---------LIYIPPDFPFDVPSVYTPHRRFSG----------------------------------NPHVQWQ-----------TYLCLYQSRN-----------TEWDASDGM-----FGFISRLELWLRRAALNQL-------------DMEGAPLH------------PPVAYPTERIT---------------
Mmc1DRAFT_1998_Masp_68246513                     ALEQVADIVAASNGTV--ELVQIDPPTSE------------GDTLLLRVSID--TSDYTFQKGGLKFRKREGF---------HIRVSSRFPIEPPIAKFTHQRFMG----------------------------------QAHVQWG-----------NQICLYLATD-----------VEWSASDGM-----FGFIKRLDQWLGDAAQDQL-------------DPDDAPLH------------PPAVYHSSDTK---FSVEIDTPELAD
pCPF5603_46_Cper_86559649                        NDDFTMFYKGLLECKN--VKNITIYKLNI------------NSVIIRLELKI--NLPSRRSLMEFDIKEFEPIK--------LLCSTNEIKYKAPLVFSDRNDFPVE--------------------------------KLPHTLAMGLNY-------SYICLHRGNI-----------DDWYIDHSV-----EDFVNRIRFWFSDAACNNL-------------IKPGDDFE------------PMINYTETGNI---VYSYNKLTKFIE
RmetDRAFT_0537_Rmet_68559822                     IADALHQLQRHRGLIR--VGEPRTTGAST------------EIEVDVAVQLP--NRSRRNGISETGVRTVETC---------VLVFGSDWPLSAP-EPFLRADFPLN---------------------------------LPHINPHRQGEL------VSPCLFEGSL-----------NELLHRFGL-----DAVVDQLIDWLHKAAAGTL-------------LDLEQGWE------------PTRRDSCPSTV---VFSAEKVAAAAP
MaquDRAFT_3270_Maqu_77955723                     HIQMLVAAILQHQRSE--DHQVTERENEL------------VLDVSWRVQLS--SRDVEVGQSGTGIKRLEPV---------RFLIPFAFPLRPP-DITLRSDFPRE--------------------------------FVPHIYPGSPGDP------VCPCIAEVGI-----------TDLMFQEGI-----SGVLRSLQAWLDRAAQGTL-------------MDPSQGWE------------PILFQNIAGSF---LDDKGSFLRGVR
Nwi_2872_Nwin_74421923                           AERFLAAALRHPECRG--GRLISVDAGGS------------RIELDLNVEMP--LAFKVDGASPNGVRVVETV---------NVRLWPSYPWSSP-SFYLRMDFPRD---------------------------------LPHVQPGPVTEP------PRPCLIDGNQ-----------REYFFQFGLVELGIFNLVHQLVLWLQRAAEGTL-------------IHHGRGWE------------PTLRCDLNDVI---ALNAEACRAVVD
XAC3952_Xaxo_21110358                            DGRMQALLRACNAHAD--INVVELRRIED------------PFIAEIIVADV--GDGAVSPGNDAGIHRIERM---------ALLYRTGARFPFE-ARPLRKTFPKA----------------------------------LHQYATGNEGP------PSLCIMEGDW-----------ELAEHRFTP-----EALLETLLAWLEKTADGTI-------------HEADRGLE------------PVFYSLGQCLM---LPPDFAEALSDP
MaquDRAFT_3597_Maqu_77955313                     NLPEPLSDLADACNDN--SDFDIVEFRRI------------SKDSYALVVDA--GDGTFDAENPVGIRRIERL---------AFVLNPNLGFPWE-VRALRSDFPVT----------------------------------MHQNHVEPNSP------RSLCLYVEPW-----------SSVERTWSP-----QSFLARALWWLRETACENL-------------HQANQPLE------------QLFFEPADQFV---LPEDYFERLTDT
PnapDRAFT_0071_Pnap_84717800                     RAKTLFDVVSRQRDYA--VVQLLQHCDDG------------TPKLECIVVEV--ECDGVPPKNGVGINYRERL----------ALCVSDDPKQLIEVLAMRKDFPVL----------------------------------MHQNQGILDAP------ASLCLYFESV-----------AAVMRTWTP-----QSFLRRIQWWLEKSARGEL-------------HPTDQPVE------------HLFFATRYELV---LPWNLSTLRKSA
OB2597_18097_Obat_84502025                       LTSSAAASFARFVDRH--AAELAAIVALR------------RGGAGELVELA--FRTGRPQQSVVPIRRTERI-----------GVRFAGGDSMPFVYVLRSDFPDT----------------------------------AHQNLTAEGSP------RAICIDDRGW-----------AEARLTWTP-----AELVQRILAWFRRAAEGAL-------------HDARQPVD------------PLMFGTGYNII---MSRALIDNANTQ
GOX2518_Goxy_58038271                            RSRLARSVIEYVCDSV--EHPYATIQEFQ------------SDGLSDIVDLE--LEIDLAQDRAVPIRHREPV---------RIVFASPDDLIAPRVLSLREDFPSG---------------------------------QVHTNLDREVDG------LCLCIWEEGW-----------HDLSRNLTG-----QALVERIRWWFAGMADGSL-------------HADDQILE------------PLVATTSDTIV---FPLGTFVGPWFI
RSP_2047_Rsph_77387013                           DEEIPDVLHPVTSLLR--IGVGPVTALEG------------WKEWRRGFFSL--PLVARVTISPGQSFPAESR-----------WHLVVSSGSYPA---DIFILPDK--------------------VAGPNLT------FPHQAAVYSRDGKEPWLNGEPCLTDPTAAFGDR------HGSRPEPIAL---ADRLIWKVERFSRWCELAAA-------------GRLHNPGD------------HFELPPLSGHT---NPMTIGFHETEG
FINAL                                            HHHHHHHHHHHHHH-------------------------------EEEEEEE---------------EEEEEE-EEEE----EE----------------E---EEE------------EEEEEEEE----------EE-----EE-------------EEEEE-----------------------H---------HHHHHH-HHHH-----------------------------------EE-HHHHHHHHH----------------
Ava_C0067_Avar_75705484                          EREGKESKYKFLSPE-----AVEKAFTSK-TAAS-------GWLSSNTIWWG---------KNPEGEAIIQFYSPQKYQIQIMGQEPEVITVPMP-----AFLFAGCSS----------RYYLWAIKGRVF-KPDTQLYKPPLPNVWED---------SSICFGG-------------------NSLS----MCSAATISQVWDLFWKSPFNKDLSQGKS-----KTHPDNIC--------------NQLIKLHESKA-KSYPSSDLVPVH
alr7559_Ana_17134644                             EREGKESKYKFLSPE-----AVEKAFTSK-TAAS-------GWLSSNTIWWG---------KNPEGEAIIQFYSPQKYQIQIMGQETEVITVPMP-----AFLFAGCGS----------RYYLWAVKGRVF-KPDAQLYKPPLPNVWED---------SSICFGG-------------------NSLS----MCSAATISQVWDLFWKSPFNKDLSQGKS-----KTHPDNIC--------------NQLIKLHESKA-KSYPSSDLVPVH
p1B75_Asp._56315656                              TVDGLRKMFDSLDPS----RSARPVFLEP-NVLS--------QGPGWLVWWM-----------KPQTRRVWFES--------KEIKLETAEVPHP-----GLVFAVTQE----------EWRVFAVQGRSRPRPGTKLYQAPYWNVWKG---------GRICAGS-------------------ARLP----SAGLQADPSGWEESFFSSR--------------FSHPNIHEKDALVKYKGG--SAKFWNAMLSGKF-KSFPQEVLVPAE
BCE_A0096_Bcer_44004435                          TFKDFYLALKEVMEQGTQDNTHYSSGVLPKGCIKH--EVLSKSGDKQAVWIE----------VPKAQWDIHFFE------------RPFQQVGFP-----RLLFRYTVYQKRVT-----NISVFAVKEDMELEEGMKLYQFPYSNVHPS---------GSVCTGR-------------------VVIP----EFRTLKDLETFHVLFFASS--------------FNHDLTHTHTEP--------VGELFKRFEN----QSFDDSILMESE
BT_2648_Bthe_29339960                            TYEFMNSLVESYTES----MSGIPHGRIPGNMLLC----DSRKGRERYIWYN-----------PPQKRKMYFQD---------GLHITDGTFNVP-----GVIYVVERE----------CMDIHAFKGA-IPEERTELYLAPFFNVAG----------ANVCLGSSS-----------------PKKPQ---DMDFLEFQEYWEKRFWMSE--------------FSHLGGNRNP----------TRSNLVSVTEHARNNPFDYSELQQSG
BproDRAFT_4305_Psp._67908644                     TLTSKNLKLLAQQAQQ---GLKQDFEVIPANVLV--------ANDSLLAWWM-----------PKGTQLMSFDVSMHELAGKSRLQGVSGNVPTP-----ALVFAMMRNRNAGGAFE--GLYVFALEKSERPTSDTSLYRAPLLNVGED---------GSVCWGD-------------------GVKP----AGKTVKDISAWQALFFSSV--------------FTHYNGTVPIVGDD------PYAFIADLMETEA-KEFPAAALKPMK
RferDRAFT_4144_Rfer_74024822                     KKDSLMAALRQLARQQ---GISDLVWVDD-QTIA--------TSSTLQVWWT-----------PAQSRWMHFQS---------QGLQLSLPAQNP-----PLVWLACGE----------CLMVFALKENIKPGPTTALHHAPLFNVFAN---------AEVCAGS-------------------MQKP-------KDGNAKEWVESFYAAT--------------FTHANPPSRRLTTYRQG---EKALWKHLMTSKKKPAFPTDKLKPFG
BproDRAFT_0623_Psp._67910471                     TEADYLAMVKVLAPQQ----RPQMEWQDH-CILA--------KGMGKMIWWT-----------PPMNRAMFFKKS---DMFGATTFSGQGICPLP-----GMVWMSDGR----------DLFVYAYRGSAMPGKETRLCQAPLFNVWAR---------GEVCVGN-------------------ASRP----DDSAKGNPQAWERFLFDSH--------------FTHPNFAQVDRLTKGVK---PAEFWKKMVAKP-AQKFPESVLVDLE
PnapDRAFT_0124_Pnap_84717439                     TQSDLNELVTGLSQSQ---SLSVPSWIDT-TMLA--------LGAGRMIWYT-----------PACQRAMFFKTS----SFTKDTFEAQGQLPTP-----GLVWLVMQG----------ALYVYAYKGSGRPDKETKLYQAPFFNVWSQ---------GKVCTGN-------------------AAMP----VGDNAAIPHMWVDAFFGSN--------------FTHPNFKEKDRLVKGVC---PIDFWKAMTEKP-LPVFPEGRLVDLP
RSc1659_Rsol_17428675                            SLGELSEFVEAAQTA-----TAYRGFIEP-HVLY--------LAPNTVAWWR-----------PAAPRTVWFSAE-------KPIGTRHGVTAHP-----PLVFIVHER----------QWYVFALAKNERPAPNTPLHVAPYFNVWER---------GEICTGN-------------------VSLP----DRPAPDALKAYETAFFDSR--------------FTHPNHARITRHKDG-----GGALWAHLLDHPEITEFPATALLPRK
RmetDRAFT_6238_Rmet_68559357                     NRMALIHAVRQVAANA----LPKGEFLTP-NVLS--------ISATTVTWWC-----------PAASRRVFFKCE--------EFGERNAIVAHP-----ALVFQASHS----------GFSVFALQGEDRPGPETALFEPPYFNTWDH---------GRICIGS-------------------AQVP----KQIDVASISGWEEGFFNSA--------------FTHPNHGGKRVAYERG----VYAFWKDMLDGKF-PDFPKQVLVPMK
PHG308_Cnec_38637969                             NRMALIHAVREVAEAS----LPNGEFLTP-NVLS--------ISPTAVTWWC-----------PAAQRRVFFDCK--------EFGKRSAVVPHP-----ALVFQASQS----------GFRVFALRGDERPVPASELCEPPYFNTWDH---------GKICIGS-------------------AHVP----KQIDVASIAGWEAGFFNSA--------------FTHPNHGSKRVTYERG----AYAFWKDMLDGQF-PDYPKQVLVPMK
Bcep1808DRAFT_6253_Bvie_67543573                 DRKVLVQTLQQLAEHV----APRAEFLPA-TVLG--------VSPEAVTWWC-----------PPAMRRVFFECE--------NLGKRSAVVPHP-----GLVFQALNQ----------GFRVFAVACSDRPVRETPLFEPPYFNTWDM---------GRICIGS-------------------AQVP----KRVDVASIDGWEAGFFDSA--------------FTHPNAGGKRIEYKDG----EYAFWRDMLDGKFGETFPLNALVPMK
Daro_2538_Daro_71847775                          TPRAAMDLAKALLKR-----AAHGGFLPE-TVLY--------MDGDLIVWWM-----------PPARRHIAFRVD-AEQAEAFGGQERGESVPHP-----GLVFAASSR----------VWRVWAVKGAGRPTPATALFQVPYFNVNVQ---------GNICHGN-------------------APVP----EGTTVEKIAAWNDAFLRSY--------------FTHPNGPGKLIRYRGG----AYTFWRDMLDGRF-QRFPERVLVDVK
PproDRAFT_0257_Ppro_71839550                     DVEMLGTLINALGRN-----VSIGGYLPP-NILS--------VGFDSMVWWV-----------KPSKRRVFFKTN------EEIIGERSEVVPHP-----GLVFGVNGSG---------VWAVCAVKGNTRPTEDTPIWQAPYFNVWSS---------GNICTGT-------------------IETP----KSVAVTETGKWEECFFSSY--------------FSHPNAHGSRQLINSRIN--PYQFWKTVLDGKY-KTFPTQKLVQTN
RBTH_06715_Bthu_75758403                         NTLFEFVQKNCYETKTNTKKLDIPVFETP-A-----------LPPGTVKYMALPDGKI-----VLFMEKKEFKHNL------TYHSTKYKQIPFP-----NLLFVFVFRPNGDKYILE-NKRCYAFRDKVF-RDTTKLYRFPFSHVQKD---------GEMCFFF---------------------LT----EMQDLAQMSSFIHNWLSAA-------------FTDHYYNLENKNKW-------GWPLRQIFSETQGQPHFNYDKLIEED
RBTH_07326_Bthu_75758953                         NTNIETIQQIFMKEQA------METPLLP-------------SQWGVVKYYRKNHYEGYVLTTPPTERVVKFDIG------RSSELPTEVTLPIP-----PMLWVFEVMTDQSGKKKLTHSMTYVIKHELL-SLKDKVFHAPFCNIGIS---------HGICWGR--------------------TLP----EVPIPKSIQSIPARFFSQPFNYDLSGNRVKPFEWTHPNGNTEDTECAVYHMMNEADKLKAAKEAGEAYSYPFDSLKPAG
FINAL                                            ---------HHHHHHH-------EEEEEE---------------EEEEE-------------------EEEEE----------------------------EEEE----------------------HHHHH-------------EE-------------EEEEEEE-------------------------  ---HHHHHHHHHHHHHHHH---
PnapDRAFT_3950_Pnap_84711628                     TEGLAALPEADQRYLD---SHGFTVEVVS----------DGPHTGVVLKQMQ----LPQGK-FNHPAADVLVI------------LPPGYPDVAP-----DMFFCNL--------------------WLTLVSAGRYPTCADQPHTFM----------GHNWQRWSRH--------------NNSWRP------GVDGLHTMIKRIEHALAEAK---------------------------------------------------------
sll6054_Ssp_38423903                             --VMTFLPESDRQYLA---NKDYTYEEIT----------EGSRKGLIFSKFP----LPNQK-YDVSEVDLLIL------------LPNGYPDIVP-----DMFYLEP--------------------AVKLVQGNRPPRATEARQQFN----------GRSWQRWSRH--------------EREWRR------GVDGIWTMLKRVEHALEVAA---------------------------------------------------------
alr7503_Ana_17134588                             --VMSFLPSNDRQYLE---NRGLPFEEVV----------DASQKGVILREFQ----LPLGR-FDTEQADILIL------------LPSGYPDAPP-----DMFYLLP--------------------WVKLVQGAKYPKAADQPHQFN----------GQKWQRWSRH--------------NNEWRP------GTDGIWTMLKRIENALEVAA---------------------------------------------------------
NhamDRAFT_1902_Nham_69928899                     PRQAFALLPVDERHLD---TMGLKWETVV----------DGGRRWLLIEGYP----VPEG--YNAAVVTLALE------------IPGPYPGAQI-----DMFYVHP--------------------ALRRLVGEEIP-ATQATETVL----------GRIFQRWSRHRGP-----------NSPWSS------RLDNVMTHLTLVDGALAKEVNQ-------------------------------------------------------
Bcep1808DRAFT_3228_Bvie_67547440                 VRADFTVMEEDAEFLN---SKGYTWEAVA----------SDAKR-IVVRGFE----PPQG--FAPTKVDMFVI------------LPQGYPDTQI-----DMVYFSP--------------------PLTRNDGKPI--RSLVTNEFE----------GKTWQGWSRHRTA-----------NSPWRQ------GIDNVGTHLMLVDDFLRAELSK-------------------------------------------------------
FINAL                                            EEHHHHHHHHHHHHHH-HHHHHHHHH-------------------EEEEE------HHHHHHHHHHHHHHH-----------------------------EEEEEEE----------------------------------EEEEEE----------------EE--------------------EE--------------HHHHH--------------------------------------------HHHHHH---HHHHHHHHHHHH
y4jF_Rsp._2496664                                AFDDQAASCAEGQATL-DLAVRLLARLYP----------------VLAILPL---DSASSFQAQALERLAKSI--------------------NPK----IGIRRSGKS------------------------------AMVCLVAGATRP-------SLRCTTFF------------------IGS-------------DGWAAKLSRT---------------DPVGSGSSLL----------PYGAGAASCFG---AANVFRTIFAAQ
mll6192_Mlot_14025925                            AFDDQAASCAEGQATL-DLAVRLLARLYP----------------VLAILPL---GSAASFQAQALERLAKSI--------------------NPK----VGIRRSGKS------------------------------ATICVVAGVTRP-------PLRCPTFF------------------MGS-------------DGWAAKLSRT---------------DPVGSGSSLL----------PYGAGAASCFG---AANVFRTIFAAQ
msi105_Mlot_20803932                             AFDDQAASCAEGQATL-DLAVRLLARLYP----------------VLAILPL---GSAASSQAQALERLAKSI--------------------NPK----VGIRRSGKS------------------------------ATICVVAGVTRP-------PLRCPTFF------------------MGS-------------DGWAAKLSRT---------------DPVGSGSSLL----------PYGAGAASCFG---AANVFRTIFAAQ
RHE_PA00014_Retl_86359719                        AFDEQACA-TEGRASL-DLLVRLVARLYP----------------TICLLPS---GEEAKKLAKNLASLARSI--------------------NED----ITIARRGSS-----------------------------ALSHCLVVGSTNP-------EISCPKFF------------------LGS-------------DGWIAKFSPE---------------EPVGTAGSNN----------AFGAGAAACIA---ASNLFRHIFRDQ

---------------------------------------------------------------------------------------------------------------------------------------------------------------------------------------

3. The JAB domain
                                        EE.HHHHHHHHHHHH.........EEEEEEEE......E..................EEEEEEEEEE..........................................................................EEEEEEEEE..........HHHHHHHH..................EEEEEEEE........................EEEEEEE.....EEE..E.EE....
                                        EE.HHHHHHHHHHHH.........EEEEEE...............................EEEEEE..........................................................................EEEEEEEE...........HHHHHHHH...................EEEEEE..........................EEEE....................
AF2198_Aful_11499780                    SRGLLKTILEAAKSA-----HPDEFIALLSGSK-----------------------DVMDELIFLPFVS-------------------------------------GSVSAVIHL-------------------DMLPIGMKVFGTVHSHPSPSC--RPSEEDLSLFTRFG---------------KYHIIVCY-----------PYDEN--------SWKCYNR----KGEEVELEVVEKD-
PH0451_Phor_14590365                    RRELLEYLLELAKSF-----YPREVAGFLRMKDG-----------------------VFEEVLIVPKGFF------------------------------------GESSVYFDL-------------------TLMPHDESIKGTFHSHPSPFP--YPSEGDLMFFSKFG---------------GIHIIAAF-----------PYDED--------SVKAFDS----EGREVELEVID---\Archaeal JAB
MK0214_Mkan_20093654                    DARLLDSLLEASDKN-----HPDEFFAMLGGSI------------------------DAETITIDSLIVVP-----------------------FEA---------SDSGAIFDL--------------------LSVHTCDVIGTFHSHPYGDP--VPSEDDLMLFKRLG---------------AVHAIAAY-----------PYTPD--------RVEFYDK----SGRNITPVVEVRYT|
MA1736_Mace_20090588                    LLYMQIKGIARDTLD-----FILEASKSMAPEEFAGLL-------------------QEQDGIITEVLILP----------------------GTES---------SNTNAVIR--------------------LYMMPNVKAVGSVHSHPGANR--RPSKADLRLFSKTG---------------NCHIIAGR-----------PYGRE--------SWTCYDR----EGNVRDLPVLDVEF|
MTH971_Mthe_15678989                    FKPVRRVVVDSEVMD-----EVLEIARRSHPHEFAALLEGRQ---------------EGEVLHVTGLIFLP-----------------------SET---------SDEGAVMDV-------------------LMLPPFTGAVGSVHSHPGPVN--LPSAADLHFFSKNG---------------LFHLIIAH-----------PYTME--------TVAAYTR----NGDPVDFEVVP---|
VNG0778C_Hasp_15789943                  GGRPSVLGIAEDALE-----FAREAAQDSHPDEYLGLLRATPASAFDLD--------ADDGYVVTDVLVIP----------------------GTET---------NPVSATFGS-------------------TQVPNDMRNVGSIHSHPNGVL--APSDADRSMFGKG----------------QLHIILGH-----------PYGPD--------CWRAFDS----EGEPRTTTVLDVDL/
Z1657_Ecol_15801143                     STRAAREWLILNMAG-----LEREEFRVLYLN-------------------------NQNQLIAGETXF-----------------------TGTINRTE------VHPREVIK--------------------RALYHNAAAVVLAHNHPSGEV--TPSKADRLITERL----------------VQALGLVDI----------RVP----------DHLIVGG----NQVFSFAEH-----\RadC
radC_Bsub_16079856                      SPEDGANLVMEDMRF-----LTQEHFVCLYLN-------------------------TKNQVIHKRTVF-----------------------IGSLNSSI------VHPREVFK--------------------EAFKRSAASFICVHNHPSGDP--TPSREDIEVTRRL----------------FECGNLIGI----------ELL----------DHLVIGD----KKFVSLKEK-----|
yfjY_Ecol_16130559                      STQAARDWLKLKMAG-----LEREEFMMLYLN-------------------------QQNQLIAHETLF-----------------------AGSISSTE------VHPREVVK--------------------RALYFNAAAVILAHNHPSGDT--TPSQADKTITQRL----------------VQALQLVDI----------RVP----------DHLIVGG----RQIYSFAEH-----|
radC_Mace_20090827                      SPKDVYALMYPRMRE-----QKKEKFITLYLD-------------------------TKNQILKEEVVS-----------------------IGSLNASI------VHPREVFK--------------------SALLESSASVIMVHNHPSGDP--SPSREDIMVTEKL----------------VEGGKLLGI----------DIL----------DHIIIGD----GRYVSLKDE-----|
radC_Ecol_6686314                       SPEMTREFLQSQLTG-----EEREIFMVIFLD-------------------------SQHRVITHRRLF-----------------------SGTLNHVE------VHPREIIR--------------------EAIKINASALILAHNHPSGCA--EPSKADKLITERI----------------IKSCQFMDL----------RVL----------DHIVIGR----GEYVSFAER-----|
RSc2620_Rsol_17547339                   SPAAVKEYLRAKLAG-----FEHEVFAVLFMD-------------------------TQHRLIEYAEMF-----------------------RGTIDGAS------VYPRELVK--------------------EALRLNAAAVIVSHNHPSGNP--EPSGADRALTQRL----------------KEALGLVDV----------RVL----------DHVIVAG----TDTTSFAER-----|
VC1786_Vcho_15641789                    RTENTTEYLRCKLAG-----YEHEIFAVLFLD-------------------------NQHRLIEFKELF-----------------------RGTVDAAS------VYPREVLK--------------------EALNVNAAAVIFAHNHPSGDP--EPSQADRRITQRL----------------KDALSLVDI----------RVL----------DHVVVGK----SS-VSFAER-----|
radC_Smel_15965481                      SWSAVIDYCHAAMAH-----ETKEQFRILFLD-------------------------KRNTLIADEVQQ-----------------------QGTIDHTP------VYPREVVK--------------------RALELSATALILVHNHPSGDP--TPSRADIDMTKLI----------------AEAAKPLGI----------ALH----------DHVIIGK----DGHVSLKGL-----|
radC_Paer_15600512                      SPQAVRDYLKARLRH-----EQHEVFACLFLD-------------------------TRHRVLSFEVLF-----------------------QGSIDGAS------VYPRQVVK--------------------RTLAHNAAALILTHNHPSGDA--RPSLADRQLTARL----------------KEALALIDV----------RVL----------DHFIIGD----GEPLSLAEY-----|
radC_Rsol_17547163                      SPQSVKDFLRLTLGH-----RPQEVFACLFLD-------------------------VRHRLIAWEELF-----------------------QGTLTEAR------VYPREIAK--------------------RALHHNASALILSHNHPTGHV--EPSESDLVLTREL----------------CRALALLDV----------RVL----------DHMIVGR----AEVYSFLEH-----|
radC_Atum_17935503                      SWSSVIDYCHAAMAH-----ETREQFRILFLD-------------------------KRNVLIADEVQG-----------------------QGTVDHTP------VYPREIVR--------------------RALELSSTALILIHNHPSGDP--TPSRADIEMTKTI----------------IDTAKPLGI----------TVH----------DHIIIGK----DGHASFKGL-----|
radC_Ssp_16331325                       SPEAAAIALSQDLMW-----QTQEHFAIVMLD-------------------------VKNRLLATKVIT-----------------------IGTATETL------IHPREIFR--------------------EVIKQGATRLIVAHNHPSGGL--EPSPEDIRLTEFL----------------LQGAQYLQI----------PVL----------DHLILGH----GKHQSLRQC-----|
[truncated: 83,799 more chars]
