# Supplementary material for: The prokaryotic antecedents of the ubiquitin-signaling system and the early evolution of ubiquitin-like β-grasp domains
Source: Genome Biol. 2006 Jul 19;7(7):R60. doi: 10.1186/gb-2006-7-7-r60 (PMC1779556; doi:10.1186/gb-2006-7-7-r60)
Supplement: Additional data file 2 — Complete list of all gi numbers for proteins encoded by conserved gene neighborhoods and their genomic position in various genomes. [file gb-2006-7-7-r60-S2.html]

```
            Supplementary material- File 2
The prokaryotic antecedents of the Ubiquitin signaling system
and the early evolution of ubiquitin-like �-grasp domains

Lakshminarayan M. Iyer, A. Maxwell Burroughs and L. Aravind


The following are the PTT tables that were derived using the TASS program.

------------PTT Tables of operons----------------------
  ORGANISM  Ruegeria sp. PR1b    accession no is AF416331.1 gi is 22726448
   cds                dir len   gi         gene     locus                pid                  product
   72909..73448       +  179   22726443                                 AAN05238.1           RC165
   73379..74824       +  481   22726444                                 AAN05239.1           RC166
   74766..75815       +  349   22726445                                 AAN05240.1           RC167
   76429..77505       +  358   22726446                                 AAN05241.1           RC168
   77677..78933       +  418   22726447                                 AAN05242.1           RC169
-->78933..80651       +  572   22726448                                 AAN05243.1           RC170
   81395..82372       +  325   22726449                                 AAN05244.1           RC171
   82508..84508       -  666   22726450                                 AAN05245.1           RC172
   84517..86850       -  777   22726451                                 AAN05246.1           RC173
   87840..88970       -  376   22726452                                 AAN05247.1           RC174
   88736..89716       -  326   22726453                                 AAN05248.1           RC175
---------------------------------------
  ORGANISM  Shewanella sp. ANA-3         accession no is NZ_AALH01000004.1 gi is 78684828
   cds                dir len   gi         gene     locus                pid                  product
   88538..88750       +  70    78684826            Shewana3DRAFT_3197   ZP_00849608.1        conserved hypothetical protein
   88929..89594       +  221   78684827            Shewana3DRAFT_3198   ZP_00849609.1        umuC protein
   90150..91682       -  510   78684919            Shewana3DRAFT_3290   ZP_00849701.1        hypothetical protein
   91679..92617       -  312   78684920            Shewana3DRAFT_3291   ZP_00849702.1        similar to Patatin
   92698..94338       -  546   78684921            Shewana3DRAFT_3292   ZP_00849703.1        hypothetical protein
-->94725..96452       -  575   78684828            Shewana3DRAFT_3199   ZP_00849610.1        similar to Dinucleotide-utilizing enzymes
   96454..97485       -  343   78684829            Shewana3DRAFT_3200   ZP_00849611.1        hypothetical protein
   97686..98030       +  114   78684922            Shewana3DRAFT_3293   ZP_00849704.1        Helix-turn-helix motif
   98040..98849       +  269   78684923            Shewana3DRAFT_3294   ZP_00849705.1        similar to Zn peptidase
   99707..100372      -  221   78684924            Shewana3DRAFT_3295   ZP_00849706.1        putative site-specific recombinase
   100510..100857     -  115   78684830            Shewana3DRAFT_3201   ZP_00849612.1        hypothetical protein
---------------------------------------
  ORGANISM  Parvularcula bermudensis HTCC2503    accession no is ZP_01015992.1 gi is 84701417
   cds                dir len   gi         gene     locus                pid                  product
   17382..20156       -  924   84701412            PB2503_00602         ZP_01015987.1        plasma membrane H+-transporting two-sector
   20781..22346       +  521   84701413            PB2503_00607         ZP_01015988.1        hypothetical protein
   22351..23205       +  284   84701414            PB2503_00612         ZP_01015989.1        hypothetical protein
   23789..24619       -  276   84701415            PB2503_00617         ZP_01015990.1        hypothetical protein
   24714..25880       +  388   84701416            PB2503_00622         ZP_01015991.1        nucleotidyl trnasferase, hypothetical protein
-->25877..27646       +  589   84701417            PB2503_00627         ZP_01015992.1        hypothetical protein
   28176..28586       +  136   84701418            PB2503_00632         ZP_01015993.1        hypothetical protein
   28596..31514       -  972   84701419            PB2503_00637         ZP_01015994.1        TraG
   31526..32944       -  472   84701420            PB2503_00642         ZP_01015995.1        TraH
   32929..33237       -  102   84701421            PB2503_00647         ZP_01015996.1        hypothetical protein
   33237..34022       -  261   84701422            PB2503_00652         ZP_01015997.1        hypothetical protein
---------------------------------------

 ORGANISM  Pseudomonas aeruginosa PAO1  accession no is AE004637.1 gi is 9948115
   cds                dir len   gi         gene     locus                pid                  product
   6121..6876         +  251   9948112             PA2099               AAG05487.1           probable short-chain dehydrogenase
   7097..8530         -  477   9948113             PA2100               AAG05488.1           probable transcriptional regulator
   8663..9553         +  296   9948114             PA2101               AAG05489.1           Permease
-->9555..10013        +  152   9948115             PA2102               AAG05490.1           hypothetical protein
---------------------------------------
  ORGANISM  Deinococcus radiodurans R1   accession no is AE000513.1 gi is 6458082
   cds                dir len   gi         gene     locus                pid                  product
   399596..401467     -  623   6458080             DR_0399              AAF09979.1           osteoblast specific factor 2-related protein
   401573..404515     -  980   6458081             DR_0400              AAF09980.1           cell division protein FtsK, putative
   404533..405204     -  223   6458085             DR_0401              AAF09984.1           hypothetical protein
-->405291..405701     +  136   6458082             DR_0402              AAF09981.1           conserved hypothetical protein
   405795..406739     -  314   6458083             DR_0403              AAF09982.1           inosine-uridine preferring nucleoside hydrolase
   406761..407105     +  114   6458086             DR_0404              AAF09985.1           hypothetical protein
   407319..410051     -  910   6458084             DR_0405              AAF09983.1           alpha-dextran endo-1,6-alpha-glucosidase
---------------------------------------
  ORGANISM  Pseudomonas aeruginosa PAO1  accession no is AE004499.1 gi is 9946515
   cds                dir len   gi         gene     locus                pid                  product
   1250..3085         +  611   9946512             PA0636               AAG04025.1           hypothetical protein
   3078..3419         +  113   9946513             PA0637               AAG04026.1           conserved hypothetical protein
   3427..4122         +  231   9946514             PA0638               AAG04027.1           probable bacteriophage protein
-->4125..4895         +  256   9946515             PA0639               AAG04028.1           JAB+NlpC;  conserved hypothetical protein
   4950..5552         +  200   9946516             PA0640               AAG04029.1           ThiS solo; probable bacteriophage protein
   5611..9225         +  1204  9946517             PA0641               AAG04030.1           probable bacteriophage protein
   9461..10249        +  262   9946518             PA0642               AAG04031.1           hypothetical protein
---------------------------------------
  ORGANISM  Pseudomonas stutzeri KC      accession no is AF149851.2 gi is 5070640
   cds                dir len   gi         gene     locus                pid                  product
   446..1012          +  188   5070637                                  AAD39223.1           hypothetical protein
   1009..2262         +  417   5070638                                  AAD39224.1           putative cell membrane protein
   2435..3610         +  391   5070639                                  AAD39225.1           MoeB-like protein
-->3626..4036         +  136   5070640                                  AAD39226.1           putative protein
   4099..4371         +  90    5070641                                  AAD39227.1           MoaD-like protein
   4460..6292         +  610   8469169                                  AAD39228.2           putative oxidoreductase
   6289..7983         +  564   5070644                                  AAD39230.1           putative AMP ligase
---------------------------------------
  ORGANISM  Pseudomonas aeruginosa 2192  accession no is NZ_AAKW01000061.1 gi is 84324136
   cds                dir len   gi         gene     locus                pid                  product
   2636..4474         +  612   84324133            Paer2_01004599       ZP_00972197.1        COG5281: Phage-related minor tail protein
   4467..4808         +  113   84324134            Paer2_01004600       ZP_00972198.1        COG4718: Phage-related protein
   4816..5511         +  231   84324135            Paer2_01004601       ZP_00972199.1        COG4672: Phage-related protein
-->5514..6284         +  256   84324136            Paer2_01004602       ZP_00972200.1        JAB+NlpC;  COG1310: Predicted metal-dependent protease of
   6339..6941         +  200   84324137            Paer2_01004603       ZP_00972201.1        COG4723: Phage-related protein, tail component
   7000..10614        +  1204  84324138            Paer2_01004604       ZP_00972202.1        COG4733: Phage-related protein, tail component
   11905..12753       +  282   84324139            Paer2_01004605       ZP_00972203.1        hypothetical protein
---------------------------------------
  ORGANISM  Burkholderia cenocepacia PC184       accession no is NZ_AAKX01000001.1 gi is 84357774
   cds                dir len   gi         gene     locus                pid                  product
   22474..26412       +  1312  84357771            BcenP_01000022       ZP_00982579.1        COG5281: Phage-related minor tail protein
   26412..26750       +  112   84357772            BcenP_01000023       ZP_00982580.1        COG4718: Phage-related protein
   28380..28937       +  185   84357773            BcenP_01000024       ZP_00982581.1        COG4672: Phage-related protein
-->28987..29739       +  250   84357774            BcenP_01000025       ZP_00982582.1        COG1310: Predicted metal-dependent protease of
   29847..30299       +  150   84357775            BcenP_01000026       ZP_00982583.1        COG4723: ThiS solo; Phage-related protein, tail component
   30452..34201       +  1249  84357776            BcenP_01000027       ZP_00982584.1        COG4733: Phage-related protein, tail component
   35384..35566       +  60    84357777            BcenP_01000028       ZP_00982585.1        hypothetical protein
---------------------------------------
  ORGANISM  Escherichia coli B7A         accession no is NZ_AAJT01000110.1 gi is 75227216
   cds                dir len   gi         gene     locus                pid                  product
   6393..9458         +  1021  75227213            EcolB7_01004347      ZP_00714015.1        COG5281: Phage-related minor tail protein
   9458..9787         +  109   75227214            EcolB7_01004348      ZP_00714016.1        COG4718: Phage-related protein
   9797..10495        +  232   75227215            EcolB7_01004349      ZP_00714017.1        COG4672: Phage-related protein
-->10645..>10914      +  90    75227216            EcolB7_01004350      ZP_00714018.1        JAB domain-possible fragment; COG1310: Predicted metal-dependent protease of
---------------------------------------
  ORGANISM  Magnetospirillum magnetotacticum MS-1        accession no is NZ_AAAP01003860.1 gi is 23015591
   cds                dir len   gi         gene     locus                pid                  product
   34419..35309       -  296   23015588            Magn03010026         ZP_00055360.1        COG0568: DNA-directed RNA polymerase, sigma
   35438..36421       -  327   23015589            Magn03010027         ZP_00055361.1        COG0564: Pseudouridylate synthases, 23S
   36449..36778       +  109   23015590            Magn03010028         ZP_00055362.1        hypothetical protein
-->36784..37254       +  156   23015591            Magn03010029         ZP_00055363.1        COG1310: Predicted metal-dependent protease of
---------------------------------------
  ORGANISM  Actinobacillus pleuropneumoniae serovar 1 str. 4074  accession no is NZ_AACK01000018.1 gi is 32034630
   cds                dir len   gi         gene     locus                pid                  product
   14958..17585       +  875   32034627            Aple02001181         ZP_00134774.1        COG0840: Methyl-accepting chemotaxis protein
   17589..17918       +  109   46143648            Aple02001182         ZP_00134775.2        hypothetical protein
   17915..18625       +  236   32034629            Aple02001183         ZP_00134776.1        COG4672: Phage-related protein
-->18629..19375       +  248   32034630            Aple02001184         ZP_00134777.1        JAB+NlpC, COG1310: Predicted metal-dependent protease of
   19408..19953       +  181   46143649            Aple02001185         ZP_00134778.2        ThiS solo; COG4723: Phage-related protein, tail component
   20002..24687       +  1561  32034632            Aple02001186         ZP_00134779.1        COG4733: Phage-related protein, tail component
   25153..25728       +  191   46143650            Aple02001187         ZP_00204539.1        hypothetical protein
---------------------------------------
  ORGANISM  Escherichia coli E110019     accession no is NZ_AAJW01000030.1 gi is 75234650
   cds                dir len   gi         gene     locus                pid                  product
   321..902           -  193   75234649            EcolE1_01003422      ZP_00718969.1        COG4723: ThiS solo; Phage-related protein, tail component
-->899..1642          -  247   75234650            JAB+NlpC EcolE1_01003423      ZP_00718970.1        COG1310: Predicted metal-dependent protease of
   1648..2346         -  232   75234651            EcolE1_01003424      ZP_00718971.1        COG4672: Phage-related protein
   2346..2687         -  113   75234652            EcolE1_01003425      ZP_00718972.1        COG4718: Phage-related protein
   2680..5922         -  1080  75234653            EcolE1_01003426      ZP_00718973.1        COG5281: Phage-related minor tail protein
---------------------------------------
  ORGANISM  Escherichia coli 53638       accession no is NZ_AAKB01000050.1 gi is 75511109
   cds                dir len   gi         gene     locus                pid                  product
   13330..16371       +  1013  75511106            Ecol5_01004566       ZP_00733834.1        COG5281: Phage-related minor tail protein
   16371..16700       +  109   75511107            Ecol5_01004567       ZP_00733835.1        COG4718: Phage-related protein
   16710..17408       +  232   75511108            Ecol5_01004568       ZP_00733836.1        COG4672: Phage-related protein
-->17557..>17922      +  122   75511109            Ecol5_01004569       ZP_00733837.1        JAB+NlpC;  COG1310: Predicted metal-dependent protease of
---------------------------------------
  ORGANISM  Escherichia coli B7A         accession no is NZ_AAJT01000016.1 gi is 75230308
   cds                dir len   gi         gene     locus                pid                  product
   69628..72189       +  853   75230305            EcolB7_01001565      ZP_00716802.1        COG5281: Phage-related minor tail protein
   72186..72515       +  109   75230306            EcolB7_01001566      ZP_00716803.1        COG4718: Phage-related protein
   72515..73213       +  232   75230307            EcolB7_01001567      ZP_00716804.1        COG4672: Phage-related protein
-->73218..>73628      +  137   75230308            EcolB7_01001568      ZP_00716805.1        JAB only; COG1310: Predicted metal-dependent protease of
---------------------------------------
  ORGANISM  Pseudomonas aeruginosa UCBPP-PA14    accession no is NZ_AABQ07000003.1 gi is 53727249
   cds                dir len   gi         gene     locus                pid                  product
   923818..925653     +  611   32043829            Paer03005044         ZP_00141091.1        COG5281: Phage-related minor tail protein
   925646..925987     +  113   32043830            Paer03005045         ZP_00141092.1        COG4718: Phage-related protein
   925995..926690     +  231   32043831            Paer03005046         ZP_00141093.1        COG4672: Phage-related protein
-->926693..927463     +  256   53727249            Paer03005047         ZP_00347819.1        JAB+NlpC;  COG1310: Predicted metal-dependent protease of
   927518..928120     +  200   32043835            Paer03005048         ZP_00141097.1        ThiS solo
   928179..931838     +  1219  32043836            Paer03005049         ZP_00141098.1        COG4733: Phage-related protein, tail component
   934291..934449     +  52    53727250            Paer03005050         ZP_00347820.1        hypothetical protein
---------------------------------------
  ORGANISM  Shigella boydii BS512        accession no is NZ_AAKA01000013.1 gi is 75176996
   cds                dir len   gi         gene     locus                pid                  product
   63793..66405       +  870   75176993            SboyB_01003016       ZP_00697100.1        COG5281: Phage-related minor tail protein
   66402..66731       +  109   75176994            SboyB_01003017       ZP_00697101.1        COG4718: Phage-related protein
   66731..67429       +  232   75176995            SboyB_01003018       ZP_00697102.1        COG4672: Phage-related protein
-->67440..68189       +  249   75176996            SboyB_01003019       ZP_00697103.1        JAB+NlpC; COG1310: Predicted metal-dependent protease of
   68152..68670       +  172   75176997            SboyB_01003020       ZP_00697104.1        ThiS solo; COG4723: Phage-related protein, tail component
   69012..72485       +  1157  75176998            SboyB_01003021       ZP_00697105.1        COG4733: Phage-related protein, tail component
   72553..73152       +  199   75176999            SboyB_01003022       ZP_00697106.1        hypothetical protein
---------------------------------------
  ORGANISM  Ralstonia solanacearum       accession no is AL646071.1 gi is 17429642
   cds                dir len   gi         gene     locus                pid                  product
   8109..9902         -  597   17429639   RSc2617                       CAD16324.1           HYPOTHETICAL PROTEIN
   9983..10813        -  276   17429640   RSc2618                       CAD16325.1           PROBABLE HOMOLOGUE TO GENE 32 PROTEIN OF F
   11359..13662       +  767   17429641   RSc2619                       CAD16326.1           CONSERVED HYPOTHETICAL PROTEIN
-->13729..14238       -  169   17429642   RSc2620                       CAD16327.1           CONSERVED HYPOTHETICAL PROTEIN
   14536..15603       -  355   17429643   RSc2621                       CAD16328.1           CONSERVED HYPOTHETICAL PROTEIN
   15600..16802       -  400   17429644   RSc2622                       CAD16329.1           PROBABLE INTEGRASE PROTEIN
   17032..18093       -  353   17429645   purM                          CAD16330.1           PROBABLE PHOSPHORIBOSYLFORMYLGLYCINAMIDINE
---------------------------------------
  ORGANISM  Burkholderia pseudomallei 1106b      accession no is NZ_AAMB01000101.1 gi is 82533243
   cds                dir len   gi         gene     locus                pid                  product
   <3..227            +  74    82533241            Bpse110_01005450     ZP_00892337.1        hypothetical protein
   224..907           +  227   82533242            Bpse110_01005451     ZP_00892338.1        COG4672: Phage-related protein
-->957..1709          +  250   82533243            Bpse110_01005452     ZP_00892339.1        JAB+NlpC;  COG1310: Predicted metal-dependent protease of
   1706..2290         +  194   82533244            Bpse110_01005453     ZP_00892340.1        ThiS, COG4723: Phage-related protein, tail component
---------------------------------------
  ORGANISM  Vibrio cholerae V51  accession no is NZ_AAKI01000014.1 gi is 75820381
   cds                dir len   gi         gene     locus                pid                  product
   20474..23290       +  938   75820378            VchoV5_01000732      ZP_00750427.1        COG5281: Phage-related minor tail protein
   23293..23631       +  112   75820379            VchoV5_01000733      ZP_00750428.1        COG4718: Phage-related protein
   23641..24387       +  248   75820380            VchoV5_01000734      ZP_00750429.1        COG4672: Phage-related protein
-->24389..25171       +  260   75820381            VchoV5_01000735      ZP_00750430.1        COG1310: Predicted metal-dependent protease of
   26488..26913       +  141   75820382            VchoV5_01000736      ZP_00750431.1        COG3637: Opacity protein and related surface
   26980..27582       +  200   75820383            VchoV5_01000737      ZP_00750432.1        COG4723: ThiS solo; Phage-related protein, tail component
   27594..31481       +  1295  75820384            VchoV5_01000738      ZP_00750433.1        COG4733: Phage-related protein, tail component
---------------------------------------
  ORGANISM  Ralstonia solanacearum       accession no is AL646065.1 gi is 17428674
   cds                dir len   gi         gene     locus                pid                  product
   169995..170999     +  334   17428671   RSc1655                       CAD15357.1           PROBABLE INTEGRASE/RECOMBINASE PROTEIN
   171362..172927     -  521   17428672   RSc1656                       CAD15358.1           HYPOTHETICAL PROTEIN
   172929..173729     -  266   17428673   RSc1657                       CAD15359.1           HYPOTHETICAL PROTEIN
-->173726..174511     -  261   17428674   RSc1658                       CAD15360.1           HYPOTHETICAL PROTEIN
   174513..175235     -  240   17428675   RSc1659                       CAD15361.1           Divergent E2, HYPOTHETICAL PROTEIN
   175243..176292     -  349   17428676   RSc1660                       CAD15362.1           HYPOTHETICAL PROTEIN
   176289..176678     -  129   17428677   RSc1661                       CAD15363.1           Ubiquitin, HYPOTHETICAL PROTEIN
---------------------------------------
  ORGANISM  Mesorhizobium loti   accession no is AL672112.1 gi is 20803931
   cds                dir len   gi         gene     locus                pid                  product
   126843..127796     -  317   20803928   msi101                        CAD31506.1           HYPOTHETICAL CONSERVED PROTEIN
   128280..128813     +  177   20803929   msi102                        CAD31507.1           HYPOTHETICAL CONSERVED PROTEIN
   128806..129423     +  205   20803930   msi103                        CAD31508.1           HYPOTHETICAL CONSERVED PROTEIN
-->129420..129908     +  162   20803931   msi104                        CAD31509.1           HYPOTHETICAL CONSERVED PROTEIN
   130013..131470     +  485   20803932   msi105                        CAD31510.1           ThiF
   131520..133490     -  656   20803933   msi106                        CAD31511.1           HYPOTHETICAL PROTEIN
   133681..134364     -  227   20803934   msi107                        CAD31512.1           HYPOTHETICAL PROTEIN
---------------------------------------
  ORGANISM  Leptospirillum ferrooxidans  accession no is AY204388.1 gi is 31747679
   cds                dir len   gi         gene     locus                pid                  product
   <1..124            -  41    31747678                                 AAO38321.1           Lfe139p1
-->124..552           -  142   31747679                                 AAO38322.1           Lfe139p2
   567..737           -  56    31747680                                 AAO38323.1           Lfe139p3
---------------------------------------
  ORGANISM  Desulfitobacterium hafniense DCB-2   accession no is NZ_AAAW03000108.1 gi is 68208688
   cds                dir len   gi         gene     locus                pid                  product
   6910..8754         +  614   68208685            DhafDRAFT_0034       ZP_00560783.1        ATP sulfurylase
   8780..9040         +  86    68208686            DhafDRAFT_0035       ZP_00560784.1        ThiS, thiamine-biosynthesis
   9040..9849         +  269   68208687            DhafDRAFT_0036       ZP_00560785.1        UBA/THIF-type NAD/FAD binding fold:MoeZ/MoeB
-->9862..10278        +  138   68208688            DhafDRAFT_0037       ZP_00560786.1        Mov34/MPN/PAD-1
   10282..11154       +  290   68208689            DhafDRAFT_0038       ZP_00560787.1        4Fe-4S ferredoxin, iron-sulfur binding, sulfite redutase
   11144..11386       +  80    68208690            DhafDRAFT_0039       ZP_00560788.1        SirA-like
   11642..12241       +  199   68208691            DhafDRAFT_0040       ZP_00560789.1        Metal-dependent phosphohydrolase, HD subdomain
---------------------------------------
  ORGANISM  Rubrobacter xylanophilus DSM 9941    accession no is NZ_AAEB02000114.1 gi is 68563153
   cds                dir len   gi         gene     locus                pid                  product
   2342..3331         +  329   68563150            RxylDRAFT_0214       ZP_00602352.1        Aldo/keto reductase
   3335..4057         +  240   68563151            RxylDRAFT_0215       ZP_00602353.1        Chlorite dismutase
   4227..5144         +  305   68563152            RxylDRAFT_0216       ZP_00602354.1        Cysteine synthase K/M
-->5147..5569         +  140   68563153            RxylDRAFT_0217       ZP_00602355.1        Mov34/MPN/PAD-1
   5588..5863         +  91    68563154            RxylDRAFT_0218       ZP_00602356.1        ThiamineS
   5913..7091         +  392   68563155            RxylDRAFT_0219       ZP_00602357.1        UBA/THIF-type NAD/FAD binding
---------------------------------------
  ORGANISM  Frankia sp. EAN1pec  accession no is NZ_AAII01000048.1 gi is 68231909
   cds                dir len   gi         gene     locus                pid                  product
   25278..25907       +  209   68231906            Franean1DRAFT_3644   ZP_00571065.1        Ham1-like protein
   26016..26963       -  315   68231907            Franean1DRAFT_3645   ZP_00571066.1        Cysteine synthase K/M
   26986..27414       -  142   68231908            Franean1DRAFT_3646   ZP_00571067.1        ThiamineS
-->27689..28135       -  148   68231909            Franean1DRAFT_3647   ZP_00571068.1        Mov34/MPN/PAD-1
   28291..28824       -  177   68231910            Franean1DRAFT_3648   ZP_00571069.1        ?
   28875..29387       -  170   68231911            Franean1DRAFT_3649   ZP_00571070.1        ClpS
   29723..30469       -  248   68231912            Franean1DRAFT_3650   ZP_00571071.1        NUDIX hydrolase
---------------------------------------
  ORGANISM  Nitrosomonas europaea ATCC 19718     accession no is BX321864.1 gi is 30181074
   cds                dir len   gi         gene     locus                pid                  product
   131337..133019     -  560   30181071   ydiD,ppsA NE2349               CAD86261.1           AMP-dependent synthetase and ligase
   133016..134902     -  628   30181072            NE2350               CAD86262.1           CAIB/BAIF family
   134940..135212     -  90    30181073            NE2351               CAD86263.1           ThiS DUF170
-->135419..135865     -  148   30181074            NE2352               CAD86264.1           Mov34 family
   135967..137139     -  390   30181075   moeZ     NE2353               CAD86265.1           Dinucleotide-utilizing enzymes involved in
   137540..138205     -  221   30181076            NE2354               CAD86266.1           hypothetical protein
   138314..139822     -  502   30181077   lysS     NE2355               CAD86267.1           lysS; putative lysyl-tRNA synthetase protein
---------------------------------------
  ORGANISM  Ralstonia solanacearum UW551         accession no is NZ_AAKL01000043.1 gi is 83748715
   cds                dir len   gi         gene     locus                pid                  product
   13765..19266       -  1833  83748712            RRSL_01362           ZP_00945728.1        DNA helicase
   19263..20456       -  397   83748713            RRSL_01363           ZP_00945729.1        Predicted hydrolases or acyltransferases
   21795..22964       +  389   83748714            RRSL_01364           ZP_00945730.1        ThiF+Rhod
-->22983..23438       +  151   83748715            RRSL_01365           ZP_00945731.1        JAB
   23435..23749       +  104   83748716            RRSL_01366           ZP_00945732.1        ThiS family protein
   23746..25626       +  626   83748717            RRSL_01367           ZP_00945733.1        Bile acid-inducible operon protein F
   25623..27275       +  550   83748718            RRSL_01368           ZP_00945734.1        2,3-dihydroxybenzoate-AMP ligase
---------------------------------------
  ORGANISM  Rhodospirillum rubrum ATCC 11170     accession no is CP000230.1 gi is 83575363
   cds                dir len   gi         gene     locus                pid                  product
   1305537..1306436   -  299   83575360            Rru_A1110            ABC21911.1           Sigma 32 (RpoH)
   1306656..1307687   -  343   83575361            Rru_A1111            ABC21912.1           Pseudouridine synthase, RluD
   1307770..1308183   +  137   83575362            Rru_A1112            ABC21913.1           hypothetical protein
-->1308173..1308628   +  151   83575363            Rru_A1113            ABC21914.1           Mov34/MPN/PAD-1
   1308659..1310119   -  486   83575364            Rru_A1114            ABC21915.1           Major facilitator superfamily MFS_1
   1310195..1311133   +  312   83575365            Rru_A1115            ABC21916.1           Transcriptional Regulator, XRE family
   1311278..1311832   +  184   83575366            Rru_A1116            ABC21917.1           Transcriptional Regulator, TetR family
---------------------------------------
  ORGANISM  Shewanella sp. W3-18-1       accession no is NZ_AALN01000024.1 gi is 82741532
   cds                dir len   gi         gene     locus                pid                  product
   20801..21475       -  224   82741529            Sputw3181DRAFT_2548  ZP_00904248.1        DNA-binding protein
   21753..22244       +  163   82741530            Sputw3181DRAFT_2549  ZP_00904249.1        hypothetical protein
   22283..22801       -  172   82741531            Sputw3181DRAFT_2550  ZP_00904250.1        lipoprotein, putative
-->22888..23601       -  237   82741532            Sputw3181DRAFT_2551  ZP_00904251.1        Mov34/MPN/PAD-1
   23653..24378       -  241   82741533            Sputw3181DRAFT_2552  ZP_00904252.1        Phage minor tail protein L
   24371..24658       -  95    82741559            Sputw3181DRAFT_2578  ZP_00904278.1        hypothetical protein
   24645..25919       -  424   82741560            Sputw3181DRAFT_2579  ZP_00904279.1        hypothetical protein
---------------------------------------
  ORGANISM  Trichodesmium erythraeum IMS101      accession no is NZ_AABK04000009.1 gi is 71676725
   cds                dir len   gi         gene     locus                pid                  product
   27966..29390       -  474   71676722            TeryDRAFT_1303       ZP_00674462.1        Deoxyribodipyrimidine photolyase
   30872..31225       -  117   71676723            TeryDRAFT_1304       ZP_00674463.1        hypothetical protein
   32666..33331       -  221   71676724            TeryDRAFT_1305       ZP_00674464.1        Forkhead-associated
-->35126..35593       +  155   71676725            TeryDRAFT_1306       ZP_00674465.1        Mov34/MPN/PAD-1
   35862..37037       +  391   71676726            TeryDRAFT_1307       ZP_00674466.1        UBA/THIF-type NAD/FAD binding
   39011..40468       +  485   71676727            TeryDRAFT_1308       ZP_00674467.1        Peptidase U62, modulator of DNA gyrase
   40614..41468       +  284   71676728            TeryDRAFT_1309       ZP_00674468.1        Ribonuclease III
---------------------------------------
  ORGANISM  Frankia sp. CcI3     accession no is NC_007777.1 gi is 86739579
   cds                dir len   gi         gene     locus                pid                  product
   1006240..1008525   -  761   86739576            Francci3_0863        YP_479976.1          molybdopterin oxidoreductase
   1008715..1009074   +  119   86739577            Francci3_0864        YP_479977.1          ClpS
   1009254..1009808   +  184   86739578            Francci3_0865        YP_479978.1          ?
-->1009861..1010265   +  134   86739579            Francci3_0866        YP_479979.1          Mov34/MPN/PAD-1
   1010506..1010880   +  124   86739580            Francci3_0867        YP_479980.1          thiamine S
   1010903..1011850   +  315   86739581            Francci3_0868        YP_479981.1          cysteine synthases
   1012117..1012779   -  220   86739582            Francci3_0869        YP_479982.1          Ham1-like protein
---------------------------------------
  ORGANISM  Caldicellulosiruptor saccharolyticus DSM 8903        accession no is NZ_AALW01000006.1 gi is 82499136
   cds                dir len   gi         gene     locus                pid                  product
   50076..51305       -  409   82499133            CsacDRAFT_2030       ZP_00884584.1        O-acetylhomoserine (thiol)-lyase
   51302..51550       -  82    82499134            CsacDRAFT_2031       ZP_00884585.1        SirA
   51543..52433       -  296   82499135            CsacDRAFT_2032       ZP_00884586.1        sulfite reductase, beta subunit
-->52454..52864       -  136   82499136            CsacDRAFT_2033       ZP_00884587.1        Mov34/MPN/PAD-1
   52916..53728       -  270   82499137            CsacDRAFT_2034       ZP_00884588.1        ThiF family protein
   53725..53934       -  69    82499138            CsacDRAFT_2035       ZP_00884589.1        ThiS, thiamine-biosynthesis
   53961..54755       -  264   82499139            CsacDRAFT_2036       ZP_00884590.1        ABC-type probable sulfate transporter, permease
---------------------------------------
  ORGANISM  Chloroflexus aurantiacus J-10-fl     accession no is NZ_AAAH02000004.1 gi is 76258731
   cds                dir len   gi         gene     locus                pid                  product
   181867..182259     +  130   76258729            CaurDRAFT_0694       ZP_00766383.1        Nitrogen-fixing NifU-like, N-terminal
   182304..183323     +  339   76258776            CaurDRAFT_0741       ZP_00766430.1        hypothetical protein
   183388..184314     +  308   76258730            CaurDRAFT_0695       ZP_00766384.1        Cysteine synthase K/M
-->184328..184813     +  161   76258731            CaurDRAFT_0696       ZP_00766385.1        Mov34/MPN/PAD-1
   184861..185145     +  94    76258732            CaurDRAFT_0697       ZP_00766386.1        ThiamineS
   185142..186296     +  384   76258733            CaurDRAFT_0698       ZP_00766387.1        UBA/THIF-type NAD/FAD binding
   186384..187397     +  337   76258734            CaurDRAFT_0699       ZP_00766388.1        Oxidoreductase, N-terminal:Oxidoreductase,
---------------------------------------
  ORGANISM  Salinibacter ruber DSM 13855         accession no is NC_007677.1 gi is 83814538
   cds                dir len   gi         gene     locus                pid                  product
   2521074..2521832   +  252   83816569            SRU_2037             YP_446143.1          oxidoreductase, short chain
   2521943..2523487   +  514   83816493            SRU_2038             YP_446144.1          Deoxyribodipyrimidine photolyase-related
   2523867..2524820   +  317   83815753            SRU_2039             YP_446145.1          cysteine synthase B
-->2524966..2525403   +  145   83814538            SRU_2040             YP_446146.1          Mov34/MPN/PAD-1 family
   2525463..2525756   +  97    83816286            SRU_2041             YP_446147.1          ThiS family
   2525937..2527154   +  405   83815308            SRU_2042             YP_446148.1          hypothetical protein
   2527280..2527714   -  144   83815082            SRU_2043             YP_446149.1          hypothetical protein
---------------------------------------
  ORGANISM  Thermobifida fusca YX        accession no is CP000088.1 gi is 71916501
   cds                dir len   gi         gene     locus                pid                  product
   2790173..2790988   -  271   71916498            Tfu_2367             AAZ56400.1           conserved hypothetical protein
   2791084..2792031   -  315   71916499            Tfu_2368             AAZ56401.1           cysteine synthase K/M
   2792037..2792315   -  92    71916500            Tfu_2369             AAZ56402.1           ThiS
-->2792434..2792841   -  135   71916501            Tfu_2370             AAZ56403.1           Mov34/MPN/PAD-1
   2792966..2793514   -  182   71916502            Tfu_2371             AAZ56404.1           ?
   2793554..2793844   -  96    71916503            Tfu_2372             AAZ56405.1           ClpS
   2793991..2795307   +  438   71916504            Tfu_2373             AAZ56406.1           nicotinate phosphoribosyltransferase related
---------------------------------------
  ORGANISM  Alkalilimnicola ehrlichei MLHE-1     accession no is NZ_AALK01000001.1 gi is 78700360
   cds                dir len   gi         gene     locus                pid                  product
   381842..382729     +  295   78700357            MlgDRAFT_2846        ZP_00864814.1        cytochrome C
   382870..383151     +  93    78700358            MlgDRAFT_2847        ZP_00864815.1        conserved hypothetical protein
   383215..384657     -  480   78700359            MlgDRAFT_2848        ZP_00864816.1        ThiS+Rhodanese+ThiF
-->384639..385067     -  142   78700360            MlgDRAFT_2849        ZP_00864817.1        Mov34/MPN/PAD-1
   385137..386225     +  362   78700361            MlgDRAFT_2850        ZP_00864818.1        conserved hypothetical protein
   386261..386659     -  132   78700362            MlgDRAFT_2851        ZP_00864819.1        hypothetical protein
   386643..388592     -  649   78700363            MlgDRAFT_2852        ZP_00864820.1        Helicase c2
---------------------------------------
  ORGANISM  Solibacter usitatus Ellin6076        accession no is NZ_AAIA01000050.1 gi is 67932292
   cds                dir len   gi         gene     locus                pid                  product
   46007..47695       +  562   67932281            AcidDRAFT_1947       ZP_00525428.1        N-acyl-D-amino-acid deacylase
   48025..49191       -  388   67932282            AcidDRAFT_1948       ZP_00525429.1        UBA/THIF-type NAD/FAD binding
   49188..49619       -  143   67932283            AcidDRAFT_1949       ZP_00525430.1        ThiamineS
-->49468..49884       -  138   67932292            AcidDRAFT_1958       ZP_00525439.1        Mov34/MPN/PAD-1
   49881..50840       -  319   67932284            AcidDRAFT_1950       ZP_00525431.1        Cysteine synthase K/M
   50808..53978       +  1056  67932285            AcidDRAFT_1951       ZP_00525432.1        Peptidase S9, prolyl oligopeptidase active site
   54023..55693       -  556   67932286            AcidDRAFT_1952       ZP_00525433.1        PAS
---------------------------------------
  ORGANISM  Methylococcus capsulatus str. Bath   accession no is AE017282.2 gi is 53756442
   cds                dir len   gi         gene     locus                pid                  product
   95813..96361       -  182   53756445            MCA0091              AAU90736.1           conserved domain protein
   96425..97483       -  352   53756444            MCA0092              AAU90735.1           conserved hypothetical protein
   97563..98783       +  406   53756443            MCA0093              AAU90734.1           ABC transporter, ATP-binding family protein
-->98827..99231       +  134   53756442            MCA0094              AAU90733.1           Mov34/MPN/PAD-1 family protein
   99236..101890      -  884   53756434            MCA0095              AAU90725.1           cation-transporting ATPase, E1-E2 family
   102004..103104     +  366   53756433            MCA0096              AAU90724.1           ISMca1, transposase
   103385..104815     -  476   53756432   gatB     MCA0097              AAU90723.1           glutamyl-tRNA(Gln) amidotransferase, B subunit
---------------------------------------
  ORGANISM  Deinococcus geothermalis DSM 11300   accession no is NZ_AAHE01000007.1 gi is 66798213
   cds                dir len   gi         gene     locus                pid                  product
   76308..77144       +  278   66798142            DgeoDRAFT_0576       ZP_00396898.1        Metallophosphoesterase
   77231..79021       -  596   66798143            DgeoDRAFT_0577       ZP_00396899.1        Beta-Ig-H3/fasciclin
   79238..82378       -  1046  66798144            DgeoDRAFT_0578       ZP_00396900.1        Cell divisionFtsK/SpoIIIE protein
-->82167..82859       +  230   66798213            DgeoDRAFT_0647       ZP_00396969.1        Mov34/MPN/PAD-1
   82896..83846       -  316   66798145            DgeoDRAFT_0579       ZP_00396901.1        Purine nucleosidase
   84219..84641       -  140   66798146            DgeoDRAFT_0580       ZP_00396902.1        Peptidylprolyl isomerase
   84604..85368       +  254   66798220            DgeoDRAFT_0655       ZP_00396976.1        Peptidase S24, S26A and S26B
---------------------------------------
  ORGANISM  Streptomyces kasugaensis     accession no is M29166.1 gi is 153352
   cds                dir len   gi         gene     locus                pid                  product
-->142..489           +  115   153352                                   AAA26783.1
---------------------------------------
  ORGANISM  Geobacter metallireducens GS-15      accession no is CP000148.1 gi is 78194034
   cds                dir len   gi         gene     locus                pid                  product
   1768827..1770071   +  414   78194031            Gmet_1566            ABB31798.1           O-acetyl-homoserine/serine sulfhydrylase/ methionine-gamma lyase
   1770084..1770296   +  70    78194032            Gmet_1567            ABB31799.1           ThiS, thiamine-biosynthesis
   1770296..1771105   +  269   78194033            Gmet_1568            ABB31800.1           UBA/THIF-type NAD/FAD binding fold:MoeZ/MoeB
-->1771108..1771506   +  132   78194034            Gmet_1569            ABB31801.1           Mov34/MPN/PAD-1
   1771521..1772483   +  320   78194035            Gmet_1570            ABB31802.1           Iron-sulfur cluster-binding protein
   1772480..1772704   +  74    78194036            Gmet_1571            ABB31803.1           conserved hypothetical protein
   1773608..1774387   +  259   78194037            Gmet_1572            ABB31804.1           Enoyl-CoA hydratase/isomerase
---------------------------------------
  ORGANISM  Clostridium thermocellum ATCC 27405  accession no is NZ_AABG04000002.1 gi is 67873786
   cds                dir len   gi         gene     locus                pid                  product
   5934..7733         +  599   67873783            CtheDRAFT_3345       ZP_00503770.1        Sulfate adenylyltransferase large subunit, Small GTP-binding protein domain
   7751..7960         +  69    67873784            CtheDRAFT_3346       ZP_00503771.1        ThiS, thiamine-biosynthesis
   7957..8769         +  270   67873785            CtheDRAFT_3347       ZP_00503772.1        UBA/THIF-type NAD/FAD binding fold:MoeZ/MoeB
-->8793..9212         +  139   67873786            CtheDRAFT_3348       ZP_00503773.1        Mov34/MPN/PAD-1
   9213..10085        +  290   67873787            CtheDRAFT_3349       ZP_00503774.1        4Fe-4S ferredoxin, iron-sulfur binding
   10123..10368       +  81    67873788            CtheDRAFT_3350       ZP_00503775.1        SirA-like
   10442..10738       -  98    67873789            CtheDRAFT_3508       ZP_00503776.1        hypothetical protein
---------------------------------------
  ORGANISM  Nitrosospira multiformis ATCC 25196  accession no is NC_007614.1 gi is 82702100
   cds                dir len   gi         gene     locus                pid                  product
   1124942..1126603   -  553   82702097            Nmul_A0968           YP_411663.1          AMP-dependent synthetase and ligase
   1126600..1128501   -  633   82702098            Nmul_A0969           YP_411664.1          L-carnitine dehydratase/bile acid-inducible
   1128565..1128837   -  90    82702099            Nmul_A0970           YP_411665.1          ThiamineS
-->1129043..1129522   -  159   82702100            Nmul_A0971           YP_411666.1          Mov34/MPN/PAD-1
   1129676..1130848   -  390   82702101            Nmul_A0972           YP_411667.1          UBA/THIF-type NAD/FAD binding fold
   1130887..1132827   -  646   82702102            Nmul_A0973           YP_411668.1          TonB-dependent receptor
   1133408..1136512   +  1034  82702103            Nmul_A0974           YP_411669.1          Glycosyl transferase, family 51
---------------------------------------
  ORGANISM  Prosthecochloris aestuarii DSM 271   accession no is NZ_AAIJ01000003.1 gi is 68551989
   cds                dir len   gi         gene     locus                pid                  product
   87490..88620       -  376   68551986            PaesDRAFT_1782       ZP_00591379.1        Tetraacyldisaccharide-1-P 4'-kinase
   88562..89209       -  215   68551987            PaesDRAFT_1783       ZP_00591380.1        Protein of unknown function DUF374
   89468..90751       +  427   68551988            PaesDRAFT_1784       ZP_00591381.1        Phosphoribosylglycinamide synthetase
-->90777..91211       +  144   68551989            PaesDRAFT_1785       ZP_00591382.1        Mov34/MPN/PAD-1
   91298..92785       +  495   68551990            PaesDRAFT_1786       ZP_00591383.1        Carbamoyl-phosphate synthase L chain,
   92782..93561       +  259   68551992            PaesDRAFT_1788       ZP_00591385.1        Indole-3-glycerol-phosphate synthase
   93542..94207       -  221   68551991            PaesDRAFT_1787       ZP_00591384.1        Ribulose-phosphate 3-epimerase
---------------------------------------
  ORGANISM  Chlorobium phaeobacteroides BS1      accession no is NZ_AAIC01000012.1 gi is 67938821
   cds                dir len   gi         gene     locus                pid                  product
   33838..34059       +  73    67938835            Cphamn1DRAFT_2840    ZP_00531354.1        ThiS, thiamine-biosynthesis
   34047..35288       +  413   67938819            Cphamn1DRAFT_2824    ZP_00531338.1        O-acetyl-homoserine/serine sulfhydrylase
   35285..36091       +  268   67938820            Cphamn1DRAFT_2825    ZP_00531339.1        UBA/THIF-type NAD/FAD binding fold:MoeZ/MoeB
-->36113..36553       +  146   67938821            Cphamn1DRAFT_2826    ZP_00531340.1        Mov34/MPN/PAD-1
   36543..37019       +  158   67938822            Cphamn1DRAFT_2827    ZP_00531341.1        conserved hypothetical protein
   37038..37286       +  82    67938823            Cphamn1DRAFT_2828    ZP_00531342.1        SirA-like
   38054..39451       -  465   67938824            Cphamn1DRAFT_2829    ZP_00531343.1        Transposase, IS4
---------------------------------------
  ORGANISM  Cyanobacteria bacterium Yellowstone B-Prime  accession no is NC_007776.1 gi is 86609521
   cds                dir len   gi         gene     locus                pid                  product
   2166882..2168315   +  477   86609518   argH     CYB_2070             YP_478280.1          argininosuccinate lyase
   2168361..2169515   -  384   86609519            CYB_2071             YP_478281.1          ISSoc1, transposase
   2169715..2170011   +  98    86609520            CYB_2072             YP_478282.1          hypothetical protein
-->2170173..2170661   +  162   86609521            CYB_2073             YP_478283.1          Mov34/MPN/PAD-1 family protein
   2170908..2171189   +  93    86609522            CYB_2074             YP_478284.1          ThiS domain protein
   2171192..2172361   +  389   86609523            CYB_2075             YP_478285.1          putative molybdopterin biosynthesis protein
   2172472..2173725   +  417   86609524   ribAB    CYB_2077             YP_478286.1          riboflavin biosynthesis protein RibA
---------------------------------------
  ORGANISM  Chlorobium phaeobacteroides BS1      accession no is NZ_AAIC01000001.1 gi is 67938030
   cds                dir len   gi         gene     locus                pid                  product
   261533..262198     +  221   67938028            Cphamn1DRAFT_3684    ZP_00530558.1        Ribulose-phosphate 3-epimerase
   262179..262955     -  258   67938027            Cphamn1DRAFT_3683    ZP_00530557.1        Indole-3-glycerol-phosphate synthase
   262957..264444     -  495   67938029            Cphamn1DRAFT_3685    ZP_00530559.1        Carbamoyl-phosphate synthase L chain,
-->264528..265046     -  172   67938030            Cphamn1DRAFT_3686    ZP_00530560.1        Mov34/MPN/PAD-1
   264976..266256     -  426   67938031            Cphamn1DRAFT_3687    ZP_00530561.1        Phosphoribosylglycinamide synthetase
   266480..267097     +  205   67938032            Cphamn1DRAFT_3688    ZP_00530562.1        Protein of unknown function DUF374
   267060..268130     +  356   67938033            Cphamn1DRAFT_3689    ZP_00530563.1        Tetraacyldisaccharide-1-P 4'-kinase
---------------------------------------
  ORGANISM  Crocosphaera watsonii WH 8501        accession no is NZ_AADV02000164.1 gi is 67925314
   cds                dir len   gi         gene     locus                pid                  product
   2405..3034         -  209   67925321            CwatDRAFT_1392       ZP_00518678.1        Transaldolase
   3371..4066         +  231   67925313            CwatDRAFT_1382       ZP_00518670.1        Peptidase M15D, vanX D-ala-D-ala dipeptidase
   4190..5296         -  368   67925320            CwatDRAFT_1390       ZP_00518677.1        Peptidase S1, chymotrypsin:PDZ/DHR/GLGF
-->5458..5970         +  170   67925314            CwatDRAFT_1383       ZP_00518671.1        Mov34/MPN/PAD-1
   6022..6549         +  175   67925315            CwatDRAFT_1384       ZP_00518672.1        hypothetical protein
   6925..7350         -  141   67925319            CwatDRAFT_1389       ZP_00518676.1        hypothetical protein
   7290..7652         -  120   67925318            CwatDRAFT_1388       ZP_00518675.1        hypothetical protein
---------------------------------------
  ORGANISM  Chlorobium phaeobacteroides DSM 266  accession no is NZ_AAIB01000002.1 gi is 67934804
   cds                dir len   gi         gene     locus                pid                  product
   111156..111821     +  221   67934802            Cpha266DRAFT_2566    ZP_00527828.1        Ribulose-phosphate 3-epimerase
   111802..112578     -  258   67934801            Cpha266DRAFT_2565    ZP_00527827.1        Indole-3-glycerol-phosphate synthase
   112584..114062     -  492   67934803            Cpha266DRAFT_2567    ZP_00527829.1        Carbamoyl-phosphate synthase L chain,
-->114150..114581     -  143   67934804            Cpha266DRAFT_2568    ZP_00527830.1        Mov34/MPN/PAD-1
   114604..115887     -  427   67934805            Cpha266DRAFT_2569    ZP_00527831.1        Phosphoribosylglycinamide synthetase
   115839..116678     +  279   67934806            Cpha266DRAFT_2570    ZP_00527832.1        Protein of unknown function DUF374
   116668..117735     +  355   67934807            Cpha266DRAFT_2571    ZP_00527833.1        Tetraacyldisaccharide-1-P 4'-kinase
---------------------------------------
  ORGANISM  Solibacter usitatus Ellin6076        accession no is NZ_AAIA01000084.1 gi is 67933547
   cds                dir len   gi         gene     locus                pid                  product
-->192..1613          +  473   67933547            AcidDRAFT_0525       ZP_00526660.1        Mov34/MPN/PAD-1
   1967..3373         -  468   67933548            AcidDRAFT_0526       ZP_00526661.1        Protein of unknown function DUF1501
   3388..5364         -  658   67933549            AcidDRAFT_0527       ZP_00526662.1        Protein of unknown function DUF1549
   5328..7343         +  671   67933550            AcidDRAFT_0528       ZP_00526663.1        hypothetical protein
---------------------------------------
  ORGANISM  Cyanobacteria bacterium Yellowstone A-Prime  accession no is NC_007775.1 gi is 86607091
   cds                dir len   gi         gene     locus                pid                  product
   2476487..2477509   -  340   86607088            CYA_2466             YP_475851.1          glycosyl transferase, group 2 family protein
   2477559..2478521   -  320   86607089            CYA_2467             YP_475852.1          Hsp33 family protein
   2478895..2479182   +  95    86607090            CYA_2468             YP_475853.1          hypothetical protein
-->2479464..2479952   +  162   86607091            CYA_2469             YP_475854.1          Mov34/MPN/PAD-1 family protein
   2480163..2480444   +  93    86607092            CYA_2470             YP_475855.1          ThiS domain protein
   2480447..2481610   +  387   86607093            CYA_2471             YP_475856.1          putative molybdopterin biosynthesis protein
   2481734..2483035   +  433   86607094   ribAB    CYA_2473             YP_475857.1          riboflavin biosynthesis protein RibA
---------------------------------------
  ORGANISM  Anabaena variabilis ATCC 29413       accession no is CP000117.1 gi is 75700941
   cds                dir len   gi         gene     locus                pid                  product
   1190855..1191586   +  243   75700938            Ava_0990             ABA20614.1           Twin-arginine translocation pathway signal
   1191961..1192695   +  244   75700939            Ava_0991             ABA20615.1           Twin-arginine translocation pathway signal
   1193091..1195304   +  737   75700940            Ava_0992             ABA20616.1           Cd/Co/Hg/Pb/Zn-translocating P-type ATPase
-->1195522..1196019   +  165   75700941            Ava_0993             ABA20617.1           Mov34/MPN/PAD-1
   1196083..1197255   +  390   75700942            Ava_0994             ABA20618.1           ThiF+Rhodanese-like MoeZ/MoeB
   1197572..1199356   -  594   75700943            Ava_0995             ABA20619.1           Transposase, IS4
   1199375..1202707   -  1110  75700944            Ava_0996             ABA20620.1           PBS lyase HEAT-like repeat
---------------------------------------
  ORGANISM  Prosthecochloris vibrioformis DSM 265        accession no is NZ_AAJD01000009.1 gi is 71482021
   cds                dir len   gi         gene     locus                pid                  product
   52693..53418       +  241   71482018            CvibDRAFT_0766       ZP_00661719.1        Ribulose-phosphate 3-epimerase
   53447..54223       -  258   71482019            CvibDRAFT_0767       ZP_00661720.1        Indole-3-glycerol-phosphate synthase
   54234..55712       -  492   71482020            CvibDRAFT_0768       ZP_00661721.1        Carbamoyl-phosphate synthase L chain,
-->55774..56205       -  143   71482021            CvibDRAFT_0769       ZP_00661722.1        Mov34/MPN/PAD-1
   56270..57553       -  427   71482022            CvibDRAFT_0770       ZP_00661723.1        Phosphoribosylglycinamide synthetase
   57720..58337       +  205   71482023            CvibDRAFT_0771       ZP_00661724.1        Protein of unknown function DUF374
   58330..59391       +  353   71482024            CvibDRAFT_0772       ZP_00661725.1        Tetraacyldisaccharide-1-P 4'-kinase
---------------------------------------
  ORGANISM  Pelodictyon phaeoclathratiforme BU-1         accession no is NZ_AAIK01000011.1 gi is 68549813
   cds                dir len   gi         gene     locus                pid                  product
   21507..22172       +  221   68549811            PphaDRAFT_2661       ZP_00589270.1        Ribulose-phosphate 3-epimerase
   22159..22929       -  256   68549810            PphaDRAFT_2660       ZP_00589269.1        Indole-3-glycerol-phosphate synthase
   22939..24417       -  492   68549812            PphaDRAFT_2662       ZP_00589271.1        Carbamoyl-phosphate synthase L chain,
-->24509..24940       -  143   68549813            PphaDRAFT_2663       ZP_00589272.1        Mov34/MPN/PAD-1
   24962..26242       -  426   68549814            PphaDRAFT_2664       ZP_00589273.1        Phosphoribosylglycinamide synthetase
   26419..27030       +  203   68549815            PphaDRAFT_2665       ZP_00589274.1        Protein of unknown function DUF374
   27023..28090       +  355   68549816            PphaDRAFT_2666       ZP_00589275.1        Tetraacyldisaccharide-1-P 4'-kinase
---------------------------------------
  ORGANISM  Salinibacter ruber DSM 13855         accession no is CP000159.1 gi is 83755932
   cds                dir len   gi         gene     locus                pid                  product
   2521074..2521832   +  252   83757963            SRU_2037             ABC46076.1           oxidoreductase, short chain
   2521943..2523487   +  514   83757887            SRU_2038             ABC46000.1           Deoxyribodipyrimidine photolyase-related
   2523867..2524820   +  317   83757147            SRU_2039             ABC45260.1           cysteine synthase B
-->2524966..2525403   +  145   83755932            SRU_2040             ABC44045.1           Mov34/MPN/PAD-1 family
   2525463..2525756   +  97    83757680            SRU_2041             ABC45793.1           ThiS family, putative
   2525937..2527154   +  405   83756702            SRU_2042             ABC44815.1           MoeZ/MoeB domain family
   2527280..2527714   -  144   83756476            SRU_2043             ABC44589.1           hypothetical protein
---------------------------------------
  ORGANISM  Magnetococcus sp. MC-1       accession no is AAAN03000008.1 gi is 68246999
   cds                dir len   gi         gene     locus                pid                  product
   40332..41189       +  285   68246996            Mmc1DRAFT_2597       EAN29086.1           Methenyltetrahydrofolate cyclohydrolase
   41308..44739       -  1143  68246997            Mmc1DRAFT_2598       EAN29087.1           Protein kinase:Sporulation related
   45110..45934       -  274   68246998            Mmc1DRAFT_2599       EAN29088.1           Conserved hypothetical protein 268
-->45943..46344       -  133   68246999            Mmc1DRAFT_2600       EAN29089.1           Mov34/MPN/PAD-1
   46613..46909       +  98    68247000            Mmc1DRAFT_2601       EAN29090.1           WGR
   46893..47522       +  209   68247001            Mmc1DRAFT_2602       EAN29091.1           Beta-lactamase-like
   47804..48133       +  109   68247002            Mmc1DRAFT_2603       EAN29092.1           hypothetical protein
---------------------------------------
  ORGANISM  Chlorobium limicola DSM 245  accession no is NZ_AAHJ01000004.1 gi is 67917899
   cds                dir len   gi         gene     locus                pid                  product
   32596..33261       +  221   67917897            ClimDRAFT_0424       ZP_00511500.1        Ribulose-phosphate 3-epimerase
   33242..34018       -  258   67917896            ClimDRAFT_0423       ZP_00511499.1        Indole-3-glycerol-phosphate synthase
   34015..35502       -  495   67917898            ClimDRAFT_0425       ZP_00511501.1        Carbamoyl-phosphate synthase L chain,
-->35605..36036       -  143   67917899            ClimDRAFT_0426       ZP_00511502.1        Mov34/MPN/PAD-1
   36066..37373       -  435   67917900            ClimDRAFT_0427       ZP_00511503.1        Phosphoribosylglycinamide synthetase
   37294..38151       +  285   67917975            ClimDRAFT_0502       ZP_00511578.1        Protein of unknown function DUF374
   38144..39196       +  350   67917901            ClimDRAFT_0428       ZP_00511504.1        Tetraacyldisaccharide-1-P 4'-kinase
---------------------------------------
  ORGANISM  Alkaliphilus metalliredigenes QYMF   accession no is NZ_AAKU01000106.1 gi is 77686499
   cds                dir len   gi         gene     locus                pid                  product
   2665..4107         -  480   77686496            AmetDRAFT_0929       ZP_00801841.1        Sodium/sulphate symporter
   4120..4746         -  208   77686497            AmetDRAFT_0930       ZP_00801842.1        hypothetical protein
   4755..7055         -  766   77686498            AmetDRAFT_0931       ZP_00801843.1        Nitrite/sulfite reductase, hemoprotein
-->7075..7488         -  137   77686499            AmetDRAFT_0932       ZP_00801844.1        Mov34/MPN/PAD-1
   7516..8325         -  269   77686500            AmetDRAFT_0933       ZP_00801845.1        UBA/THIF-type NAD/FAD binding fold:MoeZ/MoeB
   8315..8530         -  71    77686501            AmetDRAFT_0934       ZP_00801846.1        ThiS, thiamine-biosynthesis
   8958..9440         -  160   77686502            AmetDRAFT_0935       ZP_00801847.1        Conserved hypothetical protein 11
---------------------------------------
  ORGANISM  Nocardioides sp. JS614       accession no is NZ_AAJB01000020.1 gi is 71366889
   cds                dir len   gi         gene     locus                pid                  product
   27..1670           -  547   71366886            NocaDRAFT_2639       ZP_00657421.1        Nicotinate phosphoribosyltransferase related
   1725..2309         +  194   71366887            NocaDRAFT_2640       ZP_00657422.1        ?
   2430..2798         +  122   71366888            NocaDRAFT_2641       ZP_00657423.1        NUDIX hydrolase
-->2816..3274         +  152   71366889            NocaDRAFT_2642       ZP_00657424.1        Mov34/MPN/PAD-1
   3277..3549         +  90    71366890            NocaDRAFT_2643       ZP_00657425.1        ThiamineS
   3572..4534         +  320   71366891            NocaDRAFT_2644       ZP_00657426.1        Cysteine synthase K/M
   4557..5285         -  242   71366892            NocaDRAFT_2645       ZP_00657427.1        PAS
---------------------------------------
  ORGANISM  Prochlorococcus marinus subsp. marinus str. CCMP1375         accession no is AE017126.1 gi is 33238702
   cds                dir len   gi         gene     locus                pid                  product
   1571764..1573263   -  499   33238699            Pro1720              AAQ00764.1           Phytoene dehydrogenase/carotenoid isomerase
   1573302..1574192   +  296   33238700            Pro1721              AAQ00765.1           Predicted kinase, fructosamine/homoserine kinase
   1574201..1574566   -  121   33238701            Pro1722              AAQ00766.1           Predicted membrane protein
-->1574688..1575215   +  175   33238702            Pro1723              AAQ00767.1           Predicted metal-dependent protease of the
   1575228..1576367   +  379   33238703   thiF     Pro1724              AAQ00768.1           Dinucleotide-utilizing enzyme
   1576382..1577590   -  402   33238704   ctuR     Pro1725              AAQ00769.1           ATP:corrinoid adenosyltransferase
   1577671..1578528   +  285   33238705            Pro1726              AAQ00770.1           ATP-utilizing enzyme, PP-loop superfamily
---------------------------------------
  ORGANISM  Prochlorococcus marinus str. MIT 9211        accession no is AALP01000001.1 gi is 84513875
   cds                dir len   gi         gene     locus                pid                  product
   1529837..1530703   -  288   84513872            P9211_09702          EAQ10210.1           ATP-utilizing enzyme, PP-loop superfamily
   1530777..1531988   +  403   84513873            P9211_09707          EAQ10211.1           cob(I)yrinic acid a,c-diamide
   1532009..1533148   -  379   84513874            P9211_09712          EAQ10212.1           Dinucleotide-utilizing enzyme
-->1533192..1533686   -  164   84513875            P9211_09717          EAQ10213.1           Predicted metal-dependent protease of the
   1533812..1534198   +  128   84513876            P9211_09722          EAQ10214.1           Predicted membrane protein
   1534225..1535118   -  297   84513877            P9211_09727          EAQ10215.1           Predicted kinase, fructosamine/homoserine kinase
   1535153..1536664   +  503   84513878            P9211_09732          EAQ10216.1           Phytoene dehydrogenase/carotenoid isomerase
---------------------------------------
  ORGANISM  Pseudomonas putida   accession no is AY319946.2 gi is 84994017
   cds                dir len   gi         gene     locus                pid                  product
   10954..13323       +  789   84994028   pdtL                          ABC68354.1           PdtL
   13323..15842       +  839   84994029   pdtM                          ABC68355.1           PdtM
   16013..17185       +  390   84994030   pdtF                          ABC68356.1           PdtF E1+Rhodanese
-->17196..17606       +  136   84994017   pdtG                          AAQ01710.2           PdtG JAB
   17651..17923       +  90    33286618   pdtH                          AAQ01711.1           PdtH ThiS
   17994..19796       +  600   84994018   pdtI                          AAQ01712.2           PdtI
   19793..21442       +  549   84994019   pdtJ                          AAQ01713.2           PdtJ

---------------------------------------
  ORGANISM  Pseudomonas fluorescens      accession no is AY271621.1 gi is 28192389
   cds                dir len   gi         gene     locus                pid                  product
   10874..11677       +  267   28192386   qbsA                          AAL65283.1           QbsA
   11711..12802       +  363   28192387   qbsB                          AAL65284.1           QbsB
   12909..14072       +  387   28192388   qbsC                          AAL65285.1           QbsC
-->14083..14496       +  137   28192389   qbsD                          AAL65286.1           QbsD
   14518..14790       +  90    28192390   qbsE                          AAL65287.1           QbsE
   14815..15669       +  284   28192391   qbsF                          AAL65288.1           QbsF
   15693..17078       +  461   28192392   qbsG                          AAL65289.1           QbsG
---------------------------------------
  ORGANISM  Escherichia coli     accession no is AY233333.1 gi is 37927531
   cds                dir len   gi         gene     locus                pid                  product
   26817..27848       +  343   37927529                                 AAP70294.1           TnpA-like protein
   28380..28640       +  86    37927544                                 AAP70309.1           unknown
   28650..29252       +  200   37927545                                 AAP70310.1           unknown
-->29598..30134       -  178   37927531                                 AAP70296.1           VC0181-like protein
   30198..31814       -  538   37927532                                 AAP70297.1           VC0180-like protein
   31814..33112       -  432   37927533                                 AAP70298.1           VC0179-like protein
   33112..34197       -  361   37927534                                 AAP70299.1           VC0178-like protein
---------------------------------------
  ORGANISM  Rhizobium sp. NGR234         accession no is U00090.1 gi is 2182587
   cds                dir len   gi         gene     locus                pid                  product
   329159..329977     +  272   2182583             NGR234_304           AAB91823.1           Y4pM
   332506..333522     -  338   2182584    syrM1    NGR234_306           AAB91824.1           SyrM1
   335062..336264     +  400   2182585             NGR234_308           AAB91825.1           Y4pO
-->339053..339547     -  164   2182587             NGR234_311           AAB91826.1           Y4qB
   339535..341286     -  583   2182588             NGR234_312           AAB91827.1           Y4qC
   343216..343950     -  244   2182589             NGR234_313           AAB91828.1           Y4qD
   344114..345286     +  390   2182590             NGR234_314           AAB91829.1           Y4qE
---------------------------------------
sh: line 1: -d: command not found
   cds                dir len   gi         gene     locus                pid                  product
   329159..329977     +  272   2182583             NGR234_304           AAB91823.1           Y4pM
   332506..333522     -  338   2182584    syrM1    NGR234_306           AAB91824.1           SyrM1
   335062..336264     +  400   2182585             NGR234_308           AAB91825.1           Y4pO
-->339053..339547     -  164   2182587             NGR234_311           AAB91826.1           Y4qB
---------------------------------------
  ORGANISM  Nostoc sp. PCC 7120  accession no is BA000019.2 gi is 17133032
   cds                dir len   gi         gene     locus                pid                  product
   4697783..4699507   -  574   17133029   all3895                       BAB75594.1
   4699617..4700447   +  276   17133030   alr3896                       BAB75595.1
   4700541..4701365   +  274   17133031   alr3897                       BAB75596.1
-->4701353..4702180   -  275   17133032   all3898                       BAB75597.1
   4702284..4702730   -  148   17133033   mutT                          BAB75598.1           mutator protein
   4703282..4704481   +  399   17133034   alr3900                       BAB75599.1
   4704938..4705432   +  164   17133035   alr3901                       BAB75600.1
---------------------------------------
  ORGANISM  Nostoc sp. PCC 7120  accession no is BA000019.2 gi is 17132001
   cds                dir len   gi         gene     locus                pid                  product
   3544523..3545695   -  390   17131998   all2904                       BAB74603.1
   3545757..3546326   -  189   17131999   all2905                       BAB74604.1
   3546463..3547635   -  390   17132000   moeB                          BAB74605.1           molybdopterin biosynthesis protein
-->3547707..3548105   -  132   17132001   all2907                       BAB74606.1
   3548200..3550407   -  735   17132002   all2908                       BAB74607.1           cation transport ATPase
   3550757..3551374   -  205   17132003   all2909                       BAB74608.1
   3551520..3552251   -  243   17132004   moeB                       BAB74609.1           carbonate dehydratase
---------------------------------------
  ORGANISM  Chlorobium chlorochromatii CaD3      accession no is CP000108.1 gi is 78170354
   cds                dir len   gi         gene     locus                pid                  product
   189581..190696     -  371   78170351            Cag_0169             ABB27447.1           Tetraacyldisaccharide-1-P 4'-kinase
   190689..191300     -  203   78170352            Cag_0170             ABB27448.1           conserved hypothetical protein
   191587..192873     +  428   78170353            Cag_0171             ABB27449.1           Phosphoribosylglycinamide synthetase
-->192870..193304     +  144   78170354            Cag_0172             ABB27450.1           conserved hypothetical protein
   193438..194916     +  492   78170355            Cag_0173             ABB27451.1           carbamoyl-phosphate synthase, medium subunit
   194938..195711     +  257   78170356            Cag_0174             ABB27452.1           indole-3-glycerol phosphate synthase
   195698..196354     -  218   78170357            Cag_0175             ABB27453.1           Ribulose-phosphate 3-epimerase
---------------------------------------
  ORGANISM  Streptomyces coelicolor A3(2)        accession no is AL939114.1 gi is 5531362
   cds                dir len   gi         gene     locus                pid                  product
   128632..128775     -  565   78170326   SCO2912  Cag_0144             ABB27422.1           ATPase
   frameplot"         +  47    5531361                                  CAB50993.1           hypothetical protein
   128807..129229     -  298   78170328   SCO2913  Cag_0146             ABB27424.1           Monofunctional biosynthetic peptidoglycan
-->aa)                +  140   5531362                                  CAB50994.1           JAB
   129397..130824     -  475   5531363    SCO2914                       CAB50995.1           putative amino acid permease
   131176..131784     -  202   5531364    SCO2915                       CAB50996.1           ?
   131838..132155     -  105   5531365    SCO2916                       CAB50997.1           ClpS
---------------------------------------
  ORGANISM  Polaromonas naphthalenivorans CJ2    accession no is NZ_AANM01000038.1 gi is 84717438
   cds                dir len   gi         gene     locus                pid                  product
   11724..12059       +  111   84717446            PnapDRAFT_0131       ZP_01023526.1        hypothetical protein
   12566..13108       -  180   84717447            PnapDRAFT_0132       ZP_01023527.1        hypothetical protein
   13202..14047       -  281   84717437            PnapDRAFT_0122       ZP_01023517.1        ThiF solo, conserved hypothetical protein, containing a
-->14044..14688       -  214   84717438            PnapDRAFT_0123       ZP_01023518.1        JAB, conserved hypothetical protein
   14688..15485       -  265   84717439            PnapDRAFT_0124       ZP_01023519.1        Divergent E2
   15488..16648       -  386   84717448            PnapDRAFT_0133       ZP_01023528.1        hypothetical protein
   16765..17082       -  105   84717440            PnapDRAFT_0125       ZP_01023520.1        Ubiquitin, hypothetical protein
---------------------------------------
  ORGANISM  Polaromonas naphthalenivorans CJ2    accession no is NZ_AANM01000044.1 gi is 84717800
   cds                dir len   gi         gene     locus                pid                  product
   119..562           +  147   84717797            PnapDRAFT_0068       ZP_01023607.1        regulatory protein, LuxR
   835..1527          -  230   84717798            PnapDRAFT_0069       ZP_01023608.1        conserved hypothetical protein
   1533..2396         -  287   84717799            PnapDRAFT_0070       ZP_01023609.1        conserved hypothetical protein
-->2461..4716         -  751   84717800            PnapDRAFT_0071       ZP_01023610.1        conserved hypothetical protein
   4716..5870         -  384   84717801            PnapDRAFT_0072       ZP_01023611.1        conserved hypothetical protein
   5966..6577         -  203   84717802            PnapDRAFT_0073       ZP_01023612.1        hypothetical protein
   6555..7598         +  347   84717803            PnapDRAFT_0074       ZP_01023613.1        probable transposase
---------------------------------------
  ORGANISM  Symbiobacterium thermophilum IAM 14863       accession no is AP006840.1 gi is 51855978
   cds                dir len   gi         gene     locus                pid                  product
   1279381..1280559   +  392   51855975            STH1148              BAD40133.1           N-acyl-L-amino acid amidohydrolase
   1280564..1281529   +  321   51855976            STH1149              BAD40134.1           NH(3)-dependent NAD+ synthetase
   1281543..1282052   +  169   51855977            STH1150              BAD40135.1           conserved hypothetical protein
-->1282087..1282542   +  151   51855978            STH1151              BAD40136.1           conserved hypothetical protein
   1282646..1283290   +  214   51855979            STH1152              BAD40137.1           RNA polymerase ECF-type sigma factor
   1283287..1284120   +  277   51855980            STH1153              BAD40138.1           hypothetical protein
   1284177..1284764   -  195   51855981            STH1154              BAD40139.1           putative hydrolase
---------------------------------------
  ORGANISM  Mycobacterium tuberculosis CDC1551   accession no is AE000516.2 gi is 13880984
   cds                dir len   gi         gene     locus                pid                  product
   1501875..1502180   +  101   13880981            MT1373               AAK45637.1           ClpS
   1502140..1502796   +  218   13880982            MT1374               AAK45638.1           ?
   1502813..1503847   +  344   13880983            MT1375               AAK45639.1           DmpA like peptidase
-->1503855..1504295   +  146   13880984            MT1376               AAK45640.1           JAB
   1504317..1504598   +  93    13880985            MT1376.1             AAK45641.1           ThiS
   1504608..1505579   +  323   13880986   cysM     MT1377               AAK45642.1           cysteine synthase
   1505609..1506292   +  227   13880987            MT1378               AAK45643.1           Rhomboid family protein
---------------------------------------
  ORGANISM  Ralstonia metallidurans CH34         accession no is NZ_AAAI03000012.1 gi is 68559358
   cds                dir len   gi         gene     locus                pid                  product
   117618..118136     +  172   68559355            RmetDRAFT_6236       ZP_00598689.1        hypothetical protein
   118462..119601     +  379   68559356            RmetDRAFT_6237       ZP_00598690.1        hypothetical protein
   119611..120330     +  239   68559357            RmetDRAFT_6238       ZP_00598691.1        Divergent E2, conserved hypothetical protein
-->120333..120974     +  213   68559358            RmetDRAFT_6239       ZP_00598692.1        conserved hypothetical protein
   120971..121774     +  267   68559359            RmetDRAFT_6240       ZP_00598693.1        UBA/THIF-type NAD/FAD binding fold
   121969..122673     +  234   68559360            RmetDRAFT_6241       ZP_00598694.1        hypothetical protein
   122831..123004     +  57    68559361            RmetDRAFT_6242       ZP_00598695.1        hypothetical protein
---------------------------------------
  ORGANISM  Psychrobacter arcticus 273-4         accession no is CP000082.1 gi is 71038911
   cds                dir len   gi         gene     locus                pid                  product
   1651145..1652332   +  395   71038908   cca      Psyc_1368            AAZ19216.1           probable tRNA nucleotidyltransferase
   1652394..1653197   +  267   71038909            Psyc_1369            AAZ19217.1           possible Short-chain dehydrogenase/reductase
   1653540..1654811   +  423   71038910            Psyc_1370            AAZ19218.1           possible phage integrase
-->1655016..1655477   -  153   71038911            Psyc_1371            AAZ19219.1           conserved hypothetical protein
   1655467..1657236   -  589   71038912            Psyc_1372            AAZ19220.1           conserved hypothetical protein
   1657236..1658540   -  434   71038913            Psyc_1373            AAZ19221.1           conserved hypothetical protein
   1658537..1659637   -  366   71038914            Psyc_1374            AAZ19222.1           probable patatin-related protein
---------------------------------------
  ORGANISM  Synechococcus sp. CC9605     accession no is CP000110.1 gi is 78196400
   cds                dir len   gi         gene     locus                pid                  product
   386588..388090     -  500   78196397            Syncc9605_0386       ABB34162.1           phytoene dehydrogenase related enzyme
   388141..389028     +  295   78196398            Syncc9605_0387       ABB34163.1           conserved hypothetical protein
   389032..389418     -  128   78196399            Syncc9605_0388       ABB34164.1           conserved hypothetical protein
-->389483..389935     +  150   78196400            Syncc9605_0389       ABB34165.1           conserved hypothetical protein
   389928..391064     +  378   78196401            Syncc9605_0390       ABB34166.1           Rhodanese-like
   391074..392222     -  382   78196402            Syncc9605_0391       ABB34167.1           conserved hypothetical protein
   392276..393103     +  275   78196403            Syncc9605_0392       ABB34168.1           conserved hypothetical protein
---------------------------------------
  ORGANISM  Burkholderia vietnamiensis G4        accession no is NZ_AAEH02000006.1 gi is 67543574
   cds                dir len   gi         gene     locus                pid                  product
   214678..214896     +  72    67543571            Bcep1808DRAFT_6251   ZP_00421503.1        Ubiquitin, conserved hypothetical protein
   214996..216138     +  380   67543572            Bcep1808DRAFT_6252   ZP_00421504.1        hypothetical protein
   216150..216878     +  242   67543573            Bcep1808DRAFT_6253   ZP_00421505.1        Divergent E2,, conserved hypothetical protein
-->216875..217510     +  211   67543574            Bcep1808DRAFT_6254   ZP_00421506.1        JAB, conserved hypothetical protein
   217510..218328     +  272   67543575            Bcep1808DRAFT_6255   ZP_00421507.1        UBA/THIF-type NAD/FAD binding fold
   218409..218891     -  160   67543665            Bcep1808DRAFT_6345   ZP_00421597.1        hypothetical protein
   218960..219421     +  153   67543576            Bcep1808DRAFT_6256   ZP_00421508.1        15kd outer membrane lipoprotein
---------------------------------------
  ORGANISM  Wolinella succinogenes       accession no is BX571659.1 gi is 34483108
   cds                dir len   gi         gene     locus                pid                  product
   271867..273870     -  667   34483105            WS1002               CAE10104.1           CHEMOTAXIS PROTEIN (TLPB)
   274103..275368     -  421   34483106   CYSG     WS1003               CAE10105.1           SIROHEME SYNTHASE
   275365..277659     -  764   34483107            WS1004               CAE10106.1           conserved hypothetical protein
-->277663..278055     -  130   34483108            WS1005               CAE10107.1           conserved hypothetical protein
   278039..278857     -  272   34483109   MOEB     WS1006               CAE10108.1           MOLYBDOPTERIN BIOSYNTHESIS PROTEIN MOEB
   278854..279063     -  69    34483110            WS1007               CAE10109.1           hypothetical protein
   279060..280439     -  459   34483111            WS1008               CAE10110.1           GTPASE, SULFATE ADENYLATE TRANSFERASE large SUBUNIT 1
---------------------------------------
  ORGANISM  Dechloromonas aromatica RCB  accession no is CP000089.1 gi is 71847774
   cds                dir len   gi         gene     locus                pid                  product
   2730575..2730988   -  137   71847771            Daro_2534            AAZ47267.1           PilT protein, N-terminal
   2730992..2731249   -  85    71847772            Daro_2535            AAZ47268.1           Prevent-host-death protein
   2731339..2732154   -  271   71847773            Daro_2536            AAZ47269.1           UBA/THIF-type NAD/FAD binding fold
-->2732142..2732846   -  234   71847774            Daro_2537            AAZ47270.1           conserved hypothetical protein
   2732843..2733583   -  246   71847775            Daro_2538            AAZ47271.1           Divergent E2, conserved hypothetical protein
   2733601..2734620   -  339   71847776            Daro_2539            AAZ47272.1           hypothetical protein
   2734617..2735051   -  144   71847777            Daro_2540            AAZ47273.1           Ubiquitin, hypothetical protein
---------------------------------------
  ORGANISM  Vibrio cholerae O1 biovar eltor str. N16961  accession no is AE004108.1 gi is 9654585
   cds                dir len   gi         gene     locus                pid                  product
   4152..5219         +  355   9654582    VC0178                        AAF93354.1           patatin-related protein
   5233..6543         +  436   9654583    VC0179                        AAF93355.1           hypothetical protein
   6546..8300         +  584   9654584    VC0180                        AAF93356.1           conserved hypothetical protein
-->8290..8760         +  156   9654585    VC0181                        AAF93357.1           conserved hypothetical protein
   8771..9202         -  143   9654586    VC0182                        AAF93358.1           hypothetical protein
   9174..11285        -  703   9654587    VC0183                        AAF93359.1           hypothetical protein
   11295..12983       -  562   9654588    VC0184                        AAF93360.1           hypothetical protein
---------------------------------------
  ORGANISM  Azoarcus sp. EbN1    accession no is CR555307.1 gi is 56315655
   cds                dir len   gi         gene     locus                pid                  product
   31598..33397       -  599   56315652            p1B69                CAI10296.1           hypothetical protein
   33435..33620       +  61    56315653            p1B71                CAI10297.1           hypothetical protein
   33783..34574       -  263   56315654            p1B72                CAI10298.1           conserved hypothetical protein, containing a
-->34685..35350       -  221   56315655            p1B74                CAI10299.1           conserved hypothetical protein
   35350..36069       -  239   56315656            p1B75                CAI10300.1           Divergent E2, conserved hypothetical protein
   36070..37164       -  364   56315657            p1B76                CAI10301.1           hypothetical protein
   37494..37973       -  159   56315658            p1B78                CAI10302.1           hypothetical protein
---------------------------------------
  ORGANISM  Rhodoferax ferrireducens DSM 15236   accession no is NZ_AAJK01000090.1 gi is 74024823
   cds                dir len   gi         gene     locus                pid                  product
   <1..875            +  290   74024821            RferDRAFT_4143       ZP_00695321.1        hypothetical protein
   885..1610          +  241   74024822            RferDRAFT_4144       ZP_00695322.1        conserved protein with Cys, hypothetical protein
-->1801..2430         +  209   74024823            RferDRAFT_4145       ZP_00695323.1        JAB, conserved hypothetical protein
   2427..2930         +  167   74024824            RferDRAFT_4146       ZP_00695324.1        UBA/THIF-type NAD/FAD binding fold
---------------------------------------
  ORGANISM  Symbiobacterium thermophilum IAM 14863       accession no is AP006840.1 gi is 51855977
   cds                dir len   gi         gene     locus                pid                  product
   1277572..1279251   +  559   51855974            STH1147              BAD40132.1           ABC transporter permease protein
   1279381..1280559   +  392   51855975            STH1148              BAD40133.1           N-acyl-L-amino acid amidohydrolase
   1280564..1281529   +  321   51855976            STH1149              BAD40134.1           NH(3)-dependent NAD+ synthetase
-->1281543..1282052   +  169   51855977            STH1150              BAD40135.1           conserved hypothetical protein
   1282087..1282542   +  151   51855978            STH1151              BAD40136.1           conserved hypothetical protein
   1282646..1283290   +  214   51855979            STH1152              BAD40137.1           RNA polymerase ECF-type sigma factor
   1283287..1284120   +  277   51855980            STH1153              BAD40138.1           hypothetical protein
---------------------------------------
  ORGANISM  Marinobacter aquaeolei VT8   accession no is NZ_AALG01000049.1 gi is 77955313
   cds                dir len   gi         gene     locus                pid                  product
   7039..8121         +  360   77955310            MaquDRAFT_3594       ZP_00819681.1        hypothetical protein
   8588..9286         -  232   77955311            MaquDRAFT_3595       ZP_00819682.1        hypothetical protein
   10273..10530       -  85    77955312            MaquDRAFT_3596       ZP_00819683.1        hypothetical protein
-->13222..15777       -  851   77955313            MaquDRAFT_3597       ZP_00819684.1        conserved hypothetical protein
   15521..16690       -  389   77955314            MaquDRAFT_3598       ZP_00819685.1        conserved hypothetical protein
   16710..17333       -  207   77955326            MaquDRAFT_3610       ZP_00819697.1        hypothetical protein
   17694..17918       -  74    77955315            MaquDRAFT_3599       ZP_00819686.1        conserved hypothetical protein
---------------------------------------
  ORGANISM  Polaromonas sp. JS666        accession no is NZ_AAFQ02000007.1 gi is 67908645
   cds                dir len   gi         gene     locus                pid                  product
   323262..323702     +  146   67908730            BproDRAFT_4391       ZP_00507129.1        Ubiquitin, hypothetical protein
   323762..324712     +  316   67908643            BproDRAFT_4304       ZP_00507042.1        hypothetical protein
   324878..325645     +  255   67908644            BproDRAFT_4305       ZP_00507043.1        Cons protein with cys, hypothetical protein
-->325645..326238     +  197   67908645            BproDRAFT_4306       ZP_00507044.1        JAB, conserved hypothetical protein
   326238..327080     +  280   67908646            BproDRAFT_4307       ZP_00507045.1        UBA/THIF-type NAD/FAD binding fold
   327282..327539     -  85    67908731            BproDRAFT_4392       ZP_00507130.1        hypothetical protein
   327509..328075     -  188   67908732            BproDRAFT_4393       ZP_00507131.1        hypothetical protein
---------------------------------------
  ORGANISM  Thermus thermophilus HB8     accession no is AP008226.1 gi is 55772879
   cds                dir len   gi         gene     locus                pid                  product
   1422243..1423148   +  301   55772876   TTHA1494                      BAD71317.1           putative protease
   1423142..1423552   +  136   55772877   TTHA1495                      BAD71318.1           conserved hypothetical protein
   1423629..1424504   -  291   55772878   TTHA1496                      BAD71319.1           arginase
-->1424557..1425003   -  148   55772879   TTHA1497                      BAD71320.1           conserved hypothetical protein
   1425002..1426978   +  658   55772880   TTHA1498                      BAD71321.1           elongation factor G (EF-G-2)
   1426979..1427869   +  296   55772881   TTHA1499                      BAD71322.1           MoxR-related protein
   1427866..1430259   +  797   55772882   TTHA1500                      BAD71323.1           phosphoenolpyruvate synthase
---------------------------------------
  ORGANISM  Chlorobium tepidum TLS       accession no is AE006470.1 gi is 21647681
   cds                dir len   gi         gene     locus                pid                  product
   1581093..1581755   +  220   21647678   rpe      CT1670               AAM72895.1           ribulose-phosphate 3-epimerase
   1581845..1582615   -  256   21647679   trpC     CT1671               AAM72896.1           indole-3-glycerol phosphate synthase
   1582612..1584096   -  494   21647680   carB1    CT1672               AAM72897.1           carbamoyl-phosphate synthase, medium subunit
-->1584232..1584663   -  143   21647681            CT1673               AAM72898.1           conserved hypothetical protein
   1584696..1585973   -  425   21647682   purD     CT1674               AAM72899.1           phosphoribosylamine--glycine ligase
   1586157..1586786   +  209   21647683            CT1675               AAM72900.1           conserved hypothetical protein
   1586755..1587831   +  358   21647684   lpxK     CT1676               AAM72901.1           tetraacyldisaccharide 4'-kinase
---------------------------------------
  ORGANISM  Xanthomonas axonopodis pv. citri str. 306    accession no is AE012044.1 gi is 21110358
   cds                dir len   gi         gene     locus                pid                  product
   4312..4947         +  211   21110355   XAC3949                       AAM38786.1           hypothetical protein
   4960..5841         -  293   21110356   XAC3950                       AAM38787.1           conserved hypothetical protein
   6177..7145         +  322   21110357   XAC3951                       AAM38788.1           hypothetical protein
-->7142..9394         +  750   21110358   XAC3952                       AAM38789.1           conserved hypothetical protein
   9919..10233        -  104   21110359   XAC3953                       AAM38790.1           conserved hypothetical protein
   10283..11056       +  257   21110360   XAC3954                       AAM38791.1           hypothetical protein
---------------------------------------
  ORGANISM  Prochlorococcus marinus str. MIT 9313        accession no is BX572100.1 gi is 33635571
   cds                dir len   gi         gene     locus                pid                  product
   75400..76266       -  288   33635568            PMT1717              CAE21892.1           conserved hypothetical protein
   76316..77524       +  402   33635569            PMT1718              CAE21893.1           ATP:corrinoid adenosyltransferase
   77648..78877       -  409   33635570   moeB     PMT1719              CAE21894.1           molybdopterin biosynthesis protein
-->78849..79352       -  167   33635571            PMT1720              CAE21895.1           conserved hypothetical protein
   79406..79795       +  129   33635572            PMT1721              CAE21896.1           conserved hypothetical protein
   79818..80711       -  297   33635573            PMT1722              CAE21897.1           conserved hypothetical protein
   80747..82258       +  503   33635574            PMT1723              CAE21898.1           conserved hypothetical protein
---------------------------------------
  ORGANISM  Rhodopseudomonas palustris BisB18    accession no is NZ_AALR01000008.1 gi is 78493975
   cds                dir len   gi         gene     locus                pid                  product
   209548..210549     -  333   78493972            RPCDRAFT_2252        ZP_00846203.1        transferase hexapeptide repeat
   210564..211478     -  304   78493973            RPCDRAFT_2253        ZP_00846204.1        Cysteine synthase, Pyridoxal-5'-phosphate-dependent enzyme, beta
   211506..212906     -  466   78493974            RPCDRAFT_2254        ZP_00846205.1        ThiF+Rhodanese UBA/THIF-type NAD/FAD binding
-->212903..213397     -  164   78493975            RPCDRAFT_2255        ZP_00846206.1        JAB, conserved hypothetical protein
   213381..214346     -  321   78493976            RPCDRAFT_2256        ZP_00846207.1        Permease
   214416..215876     +  486   78493977            RPCDRAFT_2257        ZP_00846208.1        regulatory protein GntR, HTH
   216298..217575     -  425   78493978            RPCDRAFT_2258        ZP_00846209.1        ATP-binding region, ATPase-like:Histidine
---------------------------------------
  ORGANISM  Synechococcus sp. WH 8102    accession no is BX569694.1 gi is 33633364
   cds                dir len   gi         gene     locus                pid                  product
   207724..208554     -  276   33633361            SYNW2051             CAE08566.1           conserved hypothetical protein
   208608..209756     +  382   33633362            SYNW2052             CAE08567.1           conserved hypothetical protein
   209766..210899     -  377   33633363   moeB     SYNW2053             CAE08568.1           molybdopterin biosynthesis protein
-->210889..211350     -  153   33633364            SYNW2054             CAE08569.1           conserved hypothetical protein
   211400..211792     +  130   33633365            SYNW2055             CAE08570.1           conserved hypothetical protein
   211797..212687     -  296   33633366            SYNW2056             CAE08571.1           conserved hypothetical protein
   212738..214240     +  500   33633367            SYNW2057             CAE08572.1           phytoene dehydrogenase related enzyme
---------------------------------------
  ORGANISM  Gloeobacter violaceus PCC 7421       accession no is BA000045.2 gi is 35213986
   cds                dir len   gi         gene     locus                pid                  product
   3609638..3610114   +  158   35213983   glr3411                       BAC91352.1
   3610217..3611404   -  395   35213984   ThiF+Rhod. gll3412                       BAC91353.1
   3611407..3611685   -  92    35213985 ThiS  gsl3413                       BAC91354.1
-->3611690..3612151   -  153   35213986 JAB   gll3414                       BAC91355.1
   3612264..3612572   -  102   35213987  ? gll3415                       BAC91356.1
   3612670..3613779   -  369   35213988   recA                          BAC91357.1           recombination protein
   3614036..3615457   +  473   35213989   leuC                          BAC91358.1           3-isopropylmalate dehydratase large subunit
---------------------------------------
  ORGANISM  Rhodoferax ferrireducens DSM 15236   accession no is NZ_AAJK01000014.1 gi is 74023131
   cds                dir len   gi         gene     locus                pid                  product
   25421..26296       -  291   74023129            RferDRAFT_1304       ZP_00693705.1        Phage integrase:Phage integrase, N-terminal
   27072..27386       -  104   74023194            RferDRAFT_1369       ZP_00693770.1        hypothetical protein
   27455..28747       -  430   74023130            RferDRAFT_1305       ZP_00693706.1        hypothetical protein
-->28758..29237       -  159   74023131            RferDRAFT_1306       ZP_00693707.1        hypothetical protein
   29212..29667       -  151   74023132            RferDRAFT_1307       ZP_00693708.1        hypothetical protein
   29676..30158       -  160   74023133            RferDRAFT_1308       ZP_00693709.1        hypothetical protein
   30281..33619       -  1112  74023134            RferDRAFT_1309       ZP_00693710.1        UvrD/REP helicase:Protein of unknown function
---------------------------------------
  ORGANISM  Nitrobacter winogradskyi Nb-255      accession no is CP000115.1 gi is 74421925
   cds                dir len   gi         gene     locus                pid                  product
   3103444..3104322   +  292   74421922            Nwi_2871             ABA06121.1           conserved hypothetical protein
   3104297..3104968   +  223   74421923            Nwi_2872             ABA06122.1           hypothetical protein
   3104952..3105659   +  235   74421924            Nwi_2873             ABA06123.1           hypothetical protein
-->3105391..3106449   +  352   74421925            Nwi_2874             ABA06124.1           hypothetical protein
   3106724..3107272   +  182   74421926            Nwi_2875             ABA06125.1           hypothetical protein
   3107347..3108009   -  220   74421927            Nwi_2876             ABA06126.1           hypothetical protein
   3108006..3108965   -  319   74421928            Nwi_2877             ABA06127.1           conserved hypothetical protein
---------------------------------------
  ORGANISM  Oceanicola batsensis HTCC2597        accession no is NZ_AAMO01000001.1 gi is 84499282
   cds                dir len   gi         gene     locus                pid                  product
   88223..88540       +  105   84499279            OB2597_05110         ZP_00997567.1        hypothetical protein
   89029..90048       +  339   84499280            OB2597_05115         ZP_00997568.1        hypothetical protein
   90045..91718       +  557   84499281            OB2597_05120         ZP_00997569.1        hypothetical protein
-->91750..92172       +  140   84499282            OB2597_05125         ZP_00997570.1        hypothetical protein
   92165..93586       +  473   84499283            OB2597_05130         ZP_00997571.1        hypothetical protein
   93656..94195       +  179   84499284            OB2597_05135         ZP_00997572.1        hypothetical protein
   94237..94755       -  172   84499285            OB2597_05140         ZP_00997573.1        hypothetical protein
---------------------------------------
  ORGANISM  Polaromonas sp. JS666        accession no is NZ_AAFQ02000016.1 gi is 67910470
   cds                dir len   gi         gene     locus                pid                  product
   76150..77271       -  373   67910517            BproDRAFT_0669       ZP_00508907.1        hypothetical protein
   77268..77519       -  83    67910518            BproDRAFT_0670       ZP_00508908.1        hypothetical protein
   77749..78606       -  285   67910469            BproDRAFT_0621       ZP_00508859.1        UBA/THIF-type NAD/FAD binding fold
-->78603..79223       -  206   67910470            BproDRAFT_0622       ZP_00508860.1        hypothetical protein
   79223..80002       -  259   67910471            BproDRAFT_0623       ZP_00508861.1        conserved protein with cysteine, hypothetical protein
   79989..81083       -  364   67910472            BproDRAFT_0624       ZP_00508862.1        hypothetical protein
   81412..81654       +  80    67910519            BproDRAFT_0671       ZP_00508909.1        hypothetical protein
---------------------------------------
  ORGANISM  Cupriavidus necator  accession no is NC_005241.1 gi is 38637968
   cds                dir len   gi         gene     locus                pid                  product
   319797..320201     -  134   38637965            PHG304               NP_942939.1          hypothetical protein
   320288..320587     -  99    38637966            PHG305               NP_942940.1          hypothetical protein
   321439..322242     -  267   38637967            PHG306               NP_942941.1          putative nucleotide-binding protein
-->322239..322880     -  213   38637968            PHG307               NP_942942.1          hypothetical protein
   322877..323602     -  241   38637969            PHG308               NP_942943.1          Divergent E2, hypothetical protein
   323612..324751     -  379   38637970            PHG309               NP_942944.1          hypothetical protein
   324842..325066     -  74    38637971            PHG310               NP_942945.1          Ubiquitin, hypothetical protein
---------------------------------------
  ORGANISM  Oceanicola batsensis HTCC2597        accession no is NZ_AAMO01000007.1 gi is 84502025
   cds                dir len   gi         gene     locus                pid                  product
   222092..222361     -  89    84502022            OB2597_18082         ZP_01000180.1        hypothetical protein
   222571..222792     +  73    84502023            OB2597_18087         ZP_01000181.1        hypothetical protein
   222862..223992     +  376   84502024            OB2597_18092         ZP_01000182.1        hypothetical protein
-->223992..226277     +  761   84502025            OB2597_18097         ZP_01000183.1        hypothetical protein
   226274..227467     +  397   84502026            OB2597_18102         ZP_01000184.1        hypothetical protein
   228091..229014     +  307   84502027            OB2597_18107         ZP_01000185.1        antirestriction protein
   229105..229494     +  129   84502028            OB2597_18112         ZP_01000186.1        hypothetical protein

---------------------------------------
  ORGANISM  Ralstonia metallidurans CH34         accession no is X90708.2 gi is 56410325
   cds                dir len   gi         gene     locus                pid                  product
   88310..88777       +  155   56410322            RMe0060              CAI30204.1           hypothetical protein
   89103..90242       +  379   56410323            RMe0061              CAI30205.1           hypothetical protein
   90252..90971       +  239   56410324            RMe0062              CAI30206.1           Divergent E2
-->90989..91615       +  208   56410325            RMe0063              CAI30207.1           hypothetical protein
   91612..92415       +  267   56410326            RMe0064              CAI30208.1           hypothetical protein
   92760..93314       +  184   56410327            RMe0065              CAI30209.1           hypothetical protein
   93796..94269       +  157   56410328   sylB     RMe0066              CAI30210.1           hypothetical outer membrane lipoprotein SlyB
---------------------------------------
  ORGANISM  Nocardia farcinica IFM 10152         accession no is AP006618.1 gi is 54014564
   cds                dir len   gi         gene     locus                pid                  product
   1204925..1205245   +  106   54014561            nfa10860             BAD55931.1           ClpS
   1205272..1205859   +  195   54014562            nfa10870             BAD55932.1           ?
   1205874..1206932   +  352   54014563            nfa10880             BAD55933.1           putative peptidase
-->1207027..1207476   +  149   54014564            nfa10890             BAD55934.1           JAB
   1207542..1207829   +  95    54014565            nfa10900             BAD55935.1           ThiS
   1207834..1208796   +  320   54014566   cysM     nfa10910             BAD55936.1           putative cysteine synthase
   1208843..1209115   -  90    54014567            nfa10920             BAD55937.1           hypothetical protein
---------------------------------------
  ORGANISM  Geobacter metallireducens GS-15      accession no is CP000148.1 gi is 78193672
   cds                dir len   gi         gene     locus                pid                  product
   1340254..1341639   -  461   78193669            Gmet_1199            ABB31436.1           conserved hypothetical protein
   1341653..1348132   -  2159  78193670            Gmet_1200            ABB31437.1           hypothetical protein
   1348163..1349710   -  515   78193671            Gmet_1201            ABB31438.1           Pentapeptide repeat protein
-->1349928..1350632   -  234   78193672            Gmet_1202            ABB31439.1           hypothetical protein
   1350691..1351155   -  154   78193673            Gmet_1203            ABB31440.1           protein of unknown function DUF1003
   1351196..1351915   -  239   78193674            Gmet_1204            ABB31441.1           LrgB-like protein
   1351912..1352280   -  122   78193675            Gmet_1205            ABB31442.1           LrgA
---------------------------------------
  ORGANISM  Streptomyces avermitilis MA-4680     accession no is BA000030.2 gi is 29608821
   cds                dir len   gi         gene     locus                pid                  product
   6267279..6267596   +  105   29608818   clpS     SAV5159              BAC72871.1           ClpS
   6267609..6268214   +  201   29608819            SAV5160              BAC72872.1           ?
   6268547..6269998   +  483   29608820            SAV5161              BAC72873.1           putative proline permease
-->6270089..6270511   +  140   29608821            SAV5162              BAC72874.1           JAB
   6270890..6271177   +  95    29608822            SAV5163              BAC72875.1           ThiS
   6271195..6272145   +  316   29608823   cysM2    SAV5164              BAC72876.1           putative cysteine synthase
   6272273..6272719   -  148   29608824            SAV5165              BAC72877.1           hypothetical protein
---------------------------------------
  ORGANISM  Streptomyces avermitilis MA-4680     accession no is BA000030.2 gi is 29609625
   cds                dir len   gi         gene     locus                pid                  product
   7176435..7177229   -  264   29609622            SAV5960              BAC73672.1           putative ABC transporter integral membrane
   7177226..7178224   -  332   29609623            SAV5961              BAC73673.1           putative ABC transporter ATP-binding protein
   7178491..7179432   -  313   29609624            SAV5962              BAC73674.1           hypothetical protein
-->7179438..7180094   -  218   29609625            SAV5963              BAC73675.1           hypothetical protein
   7180384..7180920   -  178   29609626            SAV5964              BAC73676.1           hypothetical protein
   7182115..7182849   -  244   29609627            SAV5965              BAC73677.1           putative hydrolase
   7183132..7183341   -  69    29609628            SAV5966              BAC73678.1           hypothetical protein
---------------------------------------
  ORGANISM  Escherichia coli O157:H7     accession no is NC_002695.1 gi is 38703992
   cds                dir len   gi         gene     locus                pid                  product
   2163056..2166535   -  1159  15831415            ECs2161              NP_310188.1          putative host specificity protein
   2166776..2167453   -  225   15831416            ECs2162              NP_310189.1          putative tail assembly protein
   2167351..2168034   -  227   15831417            ECs2163              NP_310190.1          putative tail assembly protein
-->2167863..2168093   -  76    38703992            ECs5445              NP_944539.1          hypothetical protein
   2168104..2168802   -  232   15831418            ECs2164              NP_310191.1          putative minor tail protein
   2168802..2169131   -  109   15831419            ECs2165              NP_310192.1          putative minor tail protein
   2169128..2171740   -  870   15831420            ECs2166              NP_310193.1          putative tail length tape measure protein
---------------------------------------
  ORGANISM  Aquifex aeolicus VF5         accession no is AE000657.1 gi is 2984019
   cds                dir len   gi         gene     locus                pid                  product
   1183451..1186099   -  882   2984014    alg      aq_1684              AAC07558.1           alginate synthesis-related protein
   1186099..1187541   -  480   2984016             aq_1687              AAC07560.1           putative protein
   1187534..1189078   -  514   2984017             aq_1689              AAC07561.1           putative protein
-->1189111..1189527   +  138   2984019             aq_1691              AAC07563.1           hypothetical protein
   1189596..1190183   +  195   2984015    dplF     aq_1693              AAC07559.1           N-terminus of phage SPO1 DNA polymerase
   1190170..1190883   -  237   2984020             aq_1694              AAC07564.1           hypothetical protein
   1190915..1191805   -  296   2984018             aq_1695              AAC07562.1           putative protein
---------------------------------------
  ORGANISM  Solibacter usitatus Ellin6076        accession no is NZ_AAIA01000001.1 gi is 67926558
   cds                dir len   gi         gene     locus                pid                  product
   467017..468183     +  388   67926555            AcidDRAFT_7385       ZP_00519751.1        Aminotransferase, class I and II
   468187..468678     -  163   67926556            AcidDRAFT_7386       ZP_00519752.1        hypothetical protein
   468754..469515     -  253   67926557            AcidDRAFT_7387       ZP_00519753.1        similar to glycosyltransferases
-->469760..471517     +  585   67926558            AcidDRAFT_7388       ZP_00519754.1        hypothetical protein
   471559..472008     -  149   67926559            AcidDRAFT_7389       ZP_00519755.1        hypothetical protein
   472081..473610     -  509   67926560            AcidDRAFT_7390       ZP_00519756.1        TPR repeat
   473685..474368     +  227   67926561            AcidDRAFT_7391       ZP_00519757.1        KDPG and KHG aldolase
---------------------------------------
  ORGANISM  Gluconobacter oxydans 621H   accession no is NC_006672.1 gi is 58038271
   cds                dir len   gi         gene     locus                pid                  product
   12182..12658       +  158   58038268            GOX2515              YP_190237.1          hypothetical protein
   12741..12998       +  85    58038269            GOX2516              YP_190238.1          hypothetical protein
   13510..14625       +  371   58038270            GOX2517              YP_190239.1          hypothetical protein
-->14622..16847       +  741   58038271            GOX2518              YP_190240.1          hypothetical protein
   16834..17982       +  382   58038272            GOX2519              YP_190241.1          patatin-like phosphoesterase, hypothetical protein
   17984..18802       -  272   58038273            GOX2520              YP_190242.1          hypothetical protein
   19192..19665       +  157   58038274            GOX2521              YP_190243.1          transposase
---------------------------------------
  ORGANISM  Rhizobium etli CFN 42        accession no is NC_007762.1 gi is 86359720
   cds                dir len   gi         gene     locus                pid                  product
   15202..15708       -  168   86359717            RHE_PA00012          YP_471608.1          hypothetical protein
   15893..16207       +  104   86359718            RHE_PA00013          YP_471609.1          hypothetical protein
   16293..17837       -  514   86359719            RHE_PA00014          YP_471610.1          hypothetical protein
-->17841..18479       -  212   86359720            RHE_PA00015          YP_471611.1          hypothetical protein
   18326..18943       -  205   86359721            RHE_PA00016          YP_471612.1          hypothetical protein
   18940..19245       -  101   86359722            RHE_PA00017          YP_471613.1          hypothetical protein
   19620..21338       -  572   86359723            RHE_PA00018          YP_471614.1          hypothetical protein
---------------------------------------
  ORGANISM  Synechococcus elongatus PCC 6301     accession no is AP008231.1 gi is 56686315
   cds                dir len   gi         gene     locus                pid                  product
   1457128..1457478   -  116   56686312            syc1344_c            BAD79534.1           unknown protein
   1457577..1458119   -  180   56686313            syc1345_c            BAD79535.1           hypothetical protein
   1458222..1459157   -  311   56686314   era      syc1346_c            BAD79536.1           GTP-binding protein ERA homolog
-->1459251..1459685   +  144   56686315            syc1347_d            BAD79537.1           hypothetical protein
   1459712..1460887   +  391   56686316   moeB     syc1348_d            BAD79538.1           molybdopterin biosynthesis MoeB protein
   1461323..1461841   +  172   56686317            syc1349_d            BAD79539.1           unknown protein
   1462042..1463673   +  543   56686318            syc1350_d            BAD79540.1           phosphoglucomutase
---------------------------------------
  ORGANISM  Solibacter usitatus Ellin6076        accession no is NZ_AAIA01000001.1 gi is 67926408
   cds                dir len   gi         gene     locus                pid                  product
   250505..257773     -  2422  67926405            AcidDRAFT_7235       ZP_00519601.1        Putative Ig
   257770..258804     -  344   67926407            AcidDRAFT_7237       ZP_00519603.1        hypothetical protein
   258829..259404     -  191   67926668            AcidDRAFT_7498       ZP_00519864.1        hypothetical protein
-->259564..261366     +  600   67926408            AcidDRAFT_7238       ZP_00519604.1        hypothetical protein
   261775..262209     +  144   67926409            AcidDRAFT_7239       ZP_00519605.1        hypothetical protein
   262602..263099     +  165   67926410            AcidDRAFT_7240       ZP_00519606.1        Sigma-70 region 2
   263092..263826     +  244   67926724            AcidDRAFT_7554       ZP_00519920.1        hypothetical protein
---------------------------------------
  ORGANISM  Marinobacter aquaeolei VT8   accession no is NZ_AALG01000071.1 gi is 77955723
   cds                dir len   gi         gene     locus                pid                  product
   2307..2684         -  125   77955720            MaquDRAFT_3267       ZP_00820069.1        tISRso10a, RSp0461; ISRSO10-transposase orfA
   3871..4224         -  117   77955721            MaquDRAFT_3268       ZP_00820070.1        transposase Tra5 related protein
   4431..5573         +  380   77955722            MaquDRAFT_3269       ZP_00820071.1        hypothetical protein
-->5548..7725         +  725   77955723            MaquDRAFT_3270       ZP_00820072.1        hypothetical protein
   8700..8987         -  95    77955724            MaquDRAFT_3271       ZP_00820073.1        hypothetical protein
---------------------------------------
  ORGANISM  Synechococcus sp. RS9917     accession no is NZ_AANP01000005.1 gi is 87124949
   cds                dir len   gi         gene     locus                pid                  product
   57997..59205       -  402   87124946            RS9917_03053         ZP_01080793.1        putative transporter
   59334..60494       +  386   87124947            RS9917_03058         ZP_01080794.1        ATP:corrinoid adenosyltransferase
   60534..61703       -  389   87124948            RS9917_03063         ZP_01080795.1        Rhodanese-like
-->61704..62186       -  160   87124949            RS9917_03068         ZP_01080796.1        hypothetical protein
   62240..62626       +  128   87124950            RS9917_03073         ZP_01080797.1        hypothetical protein
   62676..63626       -  316   87124951            RS9917_03078         ZP_01080798.1        hypothetical protein
   63625..65157       +  510   87124952            RS9917_03083         ZP_01080799.1        hypothetical protein
---------------------------------------
  ORGANISM  Pseudomonas aeruginosa 2192  accession no is NZ_AAKW01000016.1 gi is 84327400
   cds                dir len   gi         gene     locus                pid                  product
   21004..21201       +  65    84327397            Paer2_01001208       ZP_00975416.1        COG0657: Esterase/lipase
   21205..21939       +  244   84327398            Paer2_01001209       ZP_00975417.1        COG1028: Dehydrogenases with different
   23759..24616       +  285   84327399            Paer2_01001210       ZP_00975418.1        COG0697: Permeases of the drug/metabolite
-->24813..25076       +  87    84327400            Paer2_01001211       ZP_00975419.1        hypothetical protein
   26152..26517       +  121   84327401            Paer2_01001212       ZP_00975420.1        COG0607: Rhodanese-related sulfurtransferase
   29053..29451       -  132   84327402            Paer2_01001213       ZP_00975421.1        hypothetical protein
   29646..31394       +  582   84327403            Paer2_01001214       ZP_00975422.1        COG0028: Thiamine pyrophosphate-requiring
---------------------------------------
  ORGANISM  Coxiella burnetii    accession no is NC_002131.1 gi is 10956045
   cds                dir len   gi         gene     locus                pid                  product
   20868..21197       +  109   10956042                                 NP_052864.1          hypothetical protein
   23386..23688       -  100   10956043                                 NP_052865.1          hypothetical protein
   23685..25265       -  526   10956044                                 NP_052866.1          hypothetical protein
-->25536..26282       -  248   10956045                                 NP_052867.1          JAB+NlpC;  hypothetical protein
   26736..27821       +  361   10956046                                 NP_052868.1          hypothetical protein
   28019..28948       -  309   10956047                                 NP_052869.1          hypothetical protein
   29298..29867       -  189   10956048                                 NP_052870.1          hypothetical protein
---------------------------------------
  ORGANISM  Arthrobacter sp. FB24        accession no is NZ_AAHG01000007.1 gi is 66965740
   cds                dir len   gi         gene     locus                pid                  product
   129393..129884     -  163   66965631            ArthDRAFT_2080       ZP_00413197.1        hypothetical protein
   129953..130489     -  178   66965632            ArthDRAFT_2081       ZP_00413198.1        hypothetical protein
   130519..130773     -  84    66965633            ArthDRAFT_2082       ZP_00413199.1        hypothetical protein
-->131270..131776     -  168   66965740            ArthDRAFT_2189       ZP_00413306.1        hypothetical protein
   131758..133536     -  592   66965723            ArthDRAFT_2172       ZP_00413289.1        UBA/THIF-type NAD/FAD binding fold
   133985..134314     -  109   66965741            ArthDRAFT_2190       ZP_00413307.1        hypothetical protein
   134402..135766     -  454   66965634            ArthDRAFT_2083       ZP_00413200.1        similar to Coenzyme F390 synthetase
---------------------------------------
  ORGANISM  Synechococcus sp. CC9902     accession no is CP000097.1 gi is 78169801
   cds                dir len   gi         gene     locus                pid                  product
   1853659..1854807   +  382   78169798            Syncc9902_1938       ABB26895.1           conserved hypothetical protein
   1855051..1855806   +  251   78169799            Syncc9902_1939       ABB26896.1           hypothetical protein
   1856231..1857397   -  388   78169800            Syncc9902_1940       ABB26897.1           Rhodanese-like
-->1857354..1857908   -  184   78169801            Syncc9902_1941       ABB26898.1           hypothetical protein
   1857907..1858305   +  132   78169802            Syncc9902_1942       ABB26899.1           conserved hypothetical protein
   1858310..1859203   -  297   78169803            Syncc9902_1943       ABB26900.1           conserved hypothetical protein
   1859269..1860756   +  495   78169804            Syncc9902_1944       ABB26901.1           phytoene dehydrogenase related enzyme
---------------------------------------
  ORGANISM  Rhizobium etli CFN 42        accession no is NC_007761.1 gi is 86357616
   cds                dir len   gi         gene     locus                pid                  product
   2080280..2080837   +  185   86357613            RHE_CH01993          YP_469505.1          probable resolvase protein
   2081423..2085139   +  1238  86357614            RHE_CH01994          YP_469506.1          hypothetical protein
   2085357..2086727   -  456   86357615            RHE_CH01995          YP_469507.1          hypothetical protein
-->2086769..2087230   -  153   86357616            RHE_CH01996          YP_469508.1          hypothetical protein
   2087205..2088893   -  562   86357617            RHE_CH01997          YP_469509.1          hypothetical protein
   2088890..2089903   -  337   86357618            RHE_CH01998          YP_469510.1          hypothetical protein
   2090223..2090729   -  168   86357619            RHE_CH01999          YP_469511.1          hypothetical protein
---------------------------------------
  ORGANISM  Bacillus cereus ATCC 10987   accession no is NC_005707.1 gi is 44004437
   cds                dir len   gi         gene     locus                pid                  product
   90338..91159       +  273   44004434            BCEA0095             NP_982102.1          hypothetical protein
   91137..91781       +  214   44004435            BCEA0096             NP_982103.1          Divergent E2, hypothetical protein
   91898..92068       +  56    44004436            BCEA0097             NP_982104.1          hypothetical protein
-->92134..93099       +  321   44004437            BCEA0098             NP_982105.1          JAB, hypothetical protein
   93150..93560       +  136   44004438            BCEA0099             NP_982106.1          ? Ubi??-perhaps not, hypothetical protein
   93563..94411       +  282   44004439            BCEA0100             NP_982107.1          ThiF solo, hypothetical protein
   94431..94802       +  123   44004440            BCEA0101             NP_982108.1          hypothetical protein
---------------------------------------
  ORGANISM  Sphingopyxis alaskensis RB2256       accession no is NZ_AAIP01000011.1 gi is 68539669
   cds                dir len   gi         gene     locus                pid                  product
   94616..97954       +  1112  68539666            SalaDRAFT_0814       ZP_00579436.1        UvrD/REP helicase:Protein of unknown function
   98038..98499       +  153   68539667            SalaDRAFT_0815       ZP_00579437.1        hypothetical protein
   98423..98959       +  178   68539668            SalaDRAFT_0816       ZP_00579438.1        hypothetical protein
-->98883..99422       +  179   68539669            SalaDRAFT_0817       ZP_00579439.1        hypothetical protein
   99436..100803      +  455   68539670            SalaDRAFT_0818       ZP_00579440.1        hypothetical protein
   100903..102153     +  416   68539671            SalaDRAFT_0819       ZP_00579441.1        Metallophosphoesterase
   102153..105620     +  1155  68539672            SalaDRAFT_0820       ZP_00579442.1        similar to Uncharacterized conserved protein
---------------------------------------
  ORGANISM  Anaeromyxobacter dehalogenans 2CP-C  accession no is NC_007760.1 gi is 86159910
   cds                dir len   gi         gene     locus                pid                  product
   4004793..4005716   -  307   86159907            Adeh_3489            YP_466692.1          Pirin-like
   4005799..4008660   +  953   86159908            Adeh_3490            YP_466693.1          protein of unknown function DUF748
   4008678..4010267   -  529   86159909            Adeh_3491            YP_466694.1          hypothetical protein
-->4010424..4010879   -  151   86159910            Adeh_3492            YP_466695.1          hypothetical protein
   4010893..4012065   -  390   86159911            Adeh_3493            YP_466696.1          UBA/THIF-type NAD/FAD binding, MoeZ/MoeB fmaily
   4012068..4012343   -  91    86159912            Adeh_3494            YP_466697.1          thiamineS
   4012470..4012835   -  121   86159913            Adeh_3495            YP_466698.1          protein of unknown function DUF156
---------------------------------------
  ORGANISM  Rhodobacter sphaeroides 2.4.1        accession no is CP000143.1 gi is 77387014
   cds                dir len   gi         gene     locus                pid                  product
   639541..641055     +  504   77387011            RSP_2045             ABA78196.1           hypothetical protein
   641174..642385     +  403   77387012            RSP_2046             ABA78197.1           nucleotidyl transferase
   642382..644187     +  601   77387013            RSP_2047             ABA78198.1           ThiF family protein
-->644180..644698     +  172   77387014            RSP_2048             ABA78199.1           hypothetical protein
   644853..645350     -  165   77387015            RSP_2049             ABA78200.1           hypothetical protein
   645363..646172     -  269   77387016            RSP_2050             ABA78201.1           hypothetical protein
   646227..647033     -  268   77387017            RSP_2052             ABA78202.1           N6 adenine-specific DNA methyltransferase, D12
---------------------------------------
  ORGANISM  Hahella chejuensis KCTC 2396         accession no is NC_007645.1 gi is 83645617
   cds                dir len   gi         gene     locus                pid                  product
   2941150..2942811   -  553   83645614            HCH_02847            YP_434049.1          Peptide arylation enzyme
   2942796..2944715   -  639   83645615            HCH_02848            YP_434050.1          predicted acyl-CoA transferase/carnitine
   2944721..2945011   -  96    83645616            HCH_02849            YP_434051.1          Molybdopterin converting factor, small subunit
-->2944947..2945465   -  172   83645617            HCH_02850            YP_434052.1          predicted metal-dependent protease of the
   2945484..2946656   -  390   83645618            HCH_02851            YP_434053.1          Dinucleotide-utilizing enzyme involved in
   2947175..2948269   +  364   83645619            HCH_02852            YP_434054.1          Integrase
   2948773..2949015   -  80    83645620            HCH_02853            YP_434055.1          hypothetical protein
---------------------------------------
  ORGANISM  Erythrobacter litoralis HTCC2594     accession no is CP000157.1 gi is 84786147
   cds                dir len   gi         gene     locus                pid                  product
   271575..272243     -  222   84786144            ELI_01170            ABC62326.1           monofunctional biosynthetic peptidoglycan
   272331..273236     -  301   84786145            ELI_01175            ABC62327.1           RNA polymerase sigma-32 factor
   273359..274327     -  322   84786146            ELI_01180            ABC62328.1           pseudouridine synthase D large subunit
-->274428..274820     +  130   84786147            ELI_01185            ABC62329.1           predicted metal-dependent protease
   274960..275604     +  214   84786148            ELI_01190            ABC62330.1           hypothetical protein
   275601..276305     +  234   84786149            ELI_01195            ABC62331.1           N-acetylmuramoyl-L-alanine amidase
   276841..278328     +  495   84786150            ELI_01200            ABC62332.1           hypothetical protein
---------------------------------------
  ORGANISM  Solibacter usitatus Ellin6076        accession no is NZ_AAIA01000067.1 gi is 67933019
   cds                dir len   gi         gene     locus                pid                  product
   14942..16633       +  563   67932997            AcidDRAFT_0786       ZP_00526127.1        hypothetical protein
   16665..18026       +  453   67932998            AcidDRAFT_0787       ZP_00526128.1        Argininosuccinate synthase
   18037..18675       +  212   67932999            AcidDRAFT_0788       ZP_00526129.1        Glutamine amidotransferase class-I
-->18901..19308       -  135   67933019            AcidDRAFT_0808       ZP_00526149.1        similar to metal-dependent protease of the
   19310..20437       -  375   67933000            AcidDRAFT_0789       ZP_00526130.1        hypothetical protein
   20443..22008       -  521   67933001            AcidDRAFT_0790       ZP_00526131.1        hypothetical protein
   22177..22995       +  272   67933002            AcidDRAFT_0791       ZP_00526132.1        hypothetical protein
---------------------------------------
  ORGANISM  Novosphingobium aromaticivorans DSM 12444    accession no is NZ_AAAV03000003.1 gi is 79041872
   cds                dir len   gi         gene     locus                pid                  product
   397674..398672     -  332   79041869            SaroDRAFT_1644       ZP_00873005.1        Pseudouridylate synthase
   398695..399870     -  391   79041870            SaroDRAFT_1643       ZP_00873006.1        conserved hypothetical protein
   399867..401672     -  601   79041871            SaroDRAFT_1642       ZP_00873007.1        methyl-accepting chemotaxis protein McpQ
-->401729..402199     +  156   79041872            SaroDRAFT_1588       ZP_00873008.1        similar to metal-dependent protease of the
   402356..402997     +  213   79041873            SaroDRAFT_1589       ZP_00873009.1        conserved hypothetical protein
   403014..403718     +  234   79041874            SaroDRAFT_1590       ZP_00873010.1        N-acetylmuramoyl-L-alanine amidase, family 2
   403752..404084     -  110   79041875            SaroDRAFT_1641       ZP_00873011.1        hypothetical protein
---------------------------------------
  ORGANISM  Synechocystis sp. PCC 6803   accession no is BA000022.2 gi is 1652702
   cds                dir len   gi         gene     locus                pid                  product
   1156580..1156975   +  131   1652699    slr0890                       BAA17619.1
   1157029..1158909   -  626   1652700    uvrC                          BAA17620.1           excinuclease ABC subunit C
   1159029..1160804   +  591   1652701    amiA                          BAA17621.1           N-acetylmuramoyl-L-alanine amidase
-->1160787..1161260   -  157   1652702    sll0864                       BAA17622.1
   1161441..1161842   -  133   1652703    sll0863                       BAA17623.1
   1161856..1163367   -  503   1652704    sll0862                       BAA17624.1
   1163421..1164338   -  305   1652705    sll0861                       BAA17625.1
---------------------------------------
  ORGANISM  Coxiella burnetii    accession no is AF064960.1 gi is 3248969
   cds                dir len   gi         gene     locus                pid                  product
--><1..431            +  142   3248969                                  AAD09944.1           unknown
---------------------------------------
  ORGANISM  Erythrobacter sp. NAP1       accession no is NZ_AAMW01000001.1 gi is 85707641
   cds                dir len   gi         gene     locus                pid                  product
   72633..73259       +  208   85707638            NAP1_00345           ZP_01038704.1        hypothetical protein
   73263..74159       -  298   85707639            NAP1_00350           ZP_01038705.1        RNA polymerase sigma factor
   74292..75245       -  317   85707640            NAP1_00355           ZP_01038706.1        pseudouridine synthase D large subunit
-->75262..75669       +  135   85707641            NAP1_00360           ZP_01038707.1        predicted metal-dependent protease
   75681..76334       +  217   85707642            NAP1_00365           ZP_01038708.1        hypothetical protein
   76331..77095       +  254   85707643            NAP1_00370           ZP_01038709.1        N-acetylmuramoyl-L-alanine amidase
   77634..78419       +  261   85707644            NAP1_00375           ZP_01038710.1        hypothetical protein
---------------------------------------
  ORGANISM  Yersinia pestis Angola       accession no is NZ_AAKS01000028.1 gi is 77634474
   cds                dir len   gi         gene     locus                pid                  product
   44156..44776       -  206   77634471            YpesA_01002191       ZP_00796571.1        COG4723: Phage-related protein, tail component
   45840..46934       -  364   77634472            YpesA_01002192       ZP_00796572.1        COG3617: Prophage antirepressor
   47722..48246       -  174   77634473            YpesA_01002193       ZP_00796573.1        hypothetical protein
-->48601..49311       -  236   77634474            YpesA_01002194       ZP_00796574.1        JAB+NlpC; COG1310: Predicted metal-dependent protease of
   49314..50066       -  250   77634475            YpesA_01002195       ZP_00796575.1        COG4672: Phage-related protein
   51010..51399       -  129   77634476            YpesA_01002197       ZP_00796576.1        COG2077: Peroxiredoxin
   51562..52536       +  324   77634477            YpesA_01002198       ZP_00796577.1        COG4948: L-alanine-DL-glutamate epimerase and
---------------------------------------
  ORGANISM  Burkholderia pseudomallei 1710a      accession no is NZ_AAHS01000005.1 gi is 67685465
   cds                dir len   gi         gene     locus                pid                  product
   149647..150288     -  213   67685462            Bpse17_01000803      ZP_00479306.1        COG0338: Site-specific DNA methylase
   152907..156095     -  1062  67685463            Bpse17_01000804      ZP_00479307.1        COG4733: Phage-related protein, tail component
   156209..156775     -  188   67685464            Bpse17_01000805      ZP_00479308.1        COG4723: Phage-related protein, tail component
-->156790..157542     -  250   67685465            Bpse17_01000806      ZP_00479309.1        COG1310: Predicted metal-dependent protease of
   157592..158275     -  227   67685466            Bpse17_01000807      ZP_00479310.1        COG4672: Phage-related protein
   158272..158607     -  111   67685467            Bpse17_01000808      ZP_00479311.1        hypothetical protein
   159669..160007     -  112   67685468            Bpse17_01000809      ZP_00479312.1        COG4718: Phage-related protein
---------------------------------------
  ORGANISM  Escherichia coli E22         accession no is NZ_AAJV01000005.1 gi is 75259294
   cds                dir len   gi         gene     locus                pid                  product
   212597..213196     -  199   75259291            EcolE2_01001182      ZP_00730639.1        COG3637: Opacity protein and related surface
   213263..216736     -  1157  75259292            EcolE2_01001183      ZP_00730640.1        COG4733: Phage-related protein, tail component
   216983..217564     -  193   75259293            EcolE2_01001184      ZP_00730641.1        COG4723: ThiS solo; Phage-related protein, tail component
-->217561..218304     -  247   75259294            EcolE2_01001185      ZP_00730642.1        COG1310: JAB+NlpC; Predicted metal-dependent protease of
   218310..219008     -  232   75259295            EcolE2_01001186      ZP_00730643.1        COG4672: Phage-related protein
   219008..219349     -  113   75259296            EcolE2_01001187      ZP_00730644.1        COG4718: Phage-related protein
   219342..219794     -  150   75259297            EcolE2_01001188      ZP_00730645.1        COG5281: Phage-related minor tail protein
---------------------------------------
  ORGANISM  Escherichia coli E110019     accession no is NZ_AAJW01000003.1 gi is 75238945
   cds                dir len   gi         gene     locus                pid                  product
   138573..139172     -  199   75238942            EcolE1_01000708      ZP_00722924.1        hypothetical protein
   139239..142637     -  1132  75238943            EcolE1_01000709      ZP_00722925.1        COG4733: Phage-related protein, tail component
   142698..143270     -  190   75238944            EcolE1_01000710      ZP_00722926.1        COG4723: ThiS solo; Phage-related protein, tail component
-->143267..144010     -  247   75238945            EcolE1_01000711      ZP_00722927.1        JAB+NlpC;  COG1310: Predicted metal-dependent protease of
   144016..144714     -  232   75238946            EcolE1_01000712      ZP_00722928.1        COG4672: Phage-related protein
   144714..145043     -  109   75238947            EcolE1_01000713      ZP_00722929.1        COG4718: Phage-related protein
   145040..147601     -  853   75238948            EcolE1_01000714      ZP_00722930.1        COG5281: Phage-related minor tail protein
---------------------------------------
  ORGANISM  Nostoc punctiforme PCC 73102         accession no is NZ_AAAY02000107.1 gi is 23124400
   cds                dir len   gi         gene     locus                pid                  product
   14782..16647       +  621   23124397            Npun02007634         ZP_00106389.1        COG0465: ATP-dependent Zn proteases
   16829..18043       -  404   53686959            Npun02007636         ZP_00106390.2        COG0477: Permeases of the major facilitator
   18372..19544       -  390   23124399            Npun02007638         ZP_00106391.1        COG0476: Dinucleotide-utilizing enzymes involved
-->19687..20145       -  152   23124400            Npun02007639         ZP_00106392.1        COG1310: Predicted metal-dependent protease of
   20927..23050       +  707   23124401            Npun02007640         ZP_00106393.1        COG2931: RTX toxins and related Ca2+-binding
   23941..25914       +  657   23124402            Npun02007641         ZP_00106394.1        COG0744: Membrane carboxypeptidase
   25967..26512       +  181   23124403            Npun02007642         ZP_00106395.1        COG3335: Transposase and inactivated
---------------------------------------
  ORGANISM  Prochlorococcus marinus str. NATL2A  accession no is CP000095.1 gi is 72002828
   cds                dir len   gi         gene     locus                pid                  product
   1073465..1074982   -  505   72002825            PMN2A_1137           AAZ58627.1           phytoene dehydrogenase/carotenoid isomerase
   1075011..1075898   +  295   72002826            PMN2A_1138           AAZ58628.1           kinase, fructosamine/homoserine kinase family
   1075935..1076297   -  120   72002827            PMN2A_1139           AAZ58629.1           conserved hypothetical protein
-->1076421..1076933   +  170   72002828            PMN2A_1140           AAZ58630.1           metal-dependent protease of the PAD1/JAB1
   1076957..1078102   +  381   72002829            PMN2A_1141           AAZ58631.1           rhodanese-like
   1078109..1079260   -  383   72002830            PMN2A_1142           AAZ58632.1           ATP:corrinoid adenosyltransferase
   1079393..1080235   +  280   72002831            PMN2A_1143           AAZ58633.1           Conserved hypothetical protein 268
---------------------------------------
  ORGANISM  Actinobacillus succinogenes 130Z     accession no is NZ_AAKC01000004.1 gi is 75428743
   cds                dir len   gi         gene     locus                pid                  product
   36651..36989       -  112   75428744            AsucDRAFT_1433       ZP_00731873.1        hypothetical protein
   37002..38012       -  336   75428745            AsucDRAFT_1434       ZP_00731874.1        hypothetical protein
   38277..42248       -  1323  75428724            AsucDRAFT_1413       ZP_00731853.1        Phage-related protein tail component-like
-->42861..43838       -  325   75428743            AsucDRAFT_1432       ZP_00731872.1        JAB+NlpC;  metal-dependent protease of the PAD1/JAB1
   43642..44379       -  245   75428725            AsucDRAFT_1414       ZP_00731854.1        Fels-1 prophage; putative minor tail protein
   44379..44714       -  111   75428726            AsucDRAFT_1415       ZP_00731855.1        Gifsy-2 prophage; probable minor tail protein
   44724..48020       -  1098  75428727            AsucDRAFT_1416       ZP_00731856.1        hypothetical protein
---------------------------------------
  ORGANISM  Nitrosococcus oceani ATCC 19707      accession no is CP000127.1 gi is 76882206
   cds                dir len   gi         gene     locus                pid                  product
   392061..393788     +  575   76882203            Noc_0358             ABA56884.1           Cl- channel, voltage gated
   393830..394552     -  240   76882204            Noc_0359             ABA56885.1           Protein of unknown function DUF533
   394641..395396     -  251   76882205            Noc_0360             ABA56886.1           hypothetical protein
-->395480..395884     -  134   76882206            Noc_0361             ABA56887.1           metal-dependent protease of the PAD1/JAB1
   395884..396657     -  257   76882207            Noc_0362             ABA56888.1           ThiF
   396714..397589     -  291   76882208            Noc_0363             ABA56889.1           Oxidoreductase FAD/NAD(P)-binding
   397586..398875     -  429   76882209            Noc_0364             ABA56890.1           Dihydroorotase multifunctional complex type
---------------------------------------
  ORGANISM  Mesorhizobium loti MAFF303099        accession no is BA000012.4 gi is 14025926
   cds                dir len   gi         gene     locus                pid                  product
   5009628..5010773   +  381   14025923   mlr6190                       BAB52522.1           transposase
   5011308..5013278   +  656   14025924   mlr6191                       BAB52523.1
   5013328..5014887   -  519   14025925   E2?+E1 mll6192                       BAB52524.1
-->5014891..5015529   -  212   14025926   JAB, mll6193                       BAB52525.1
   5015376..5015993   -  205   14025927   proteasE? mll8758                       BAB52526.1
   5015986..5016510   -  174   14025928   mll6195                       BAB52527.1
   5016994..5017953   +  319   14025929   mlr6196                       BAB52528.1
---------------------------------------

  ORGANISM  Microbulbifer degradans 2-40         accession no is NZ_AABI03000001.1 gi is 48864353
   cds                dir len   gi         gene     locus                pid                  product
   835286..836041     +  251   48864350            Mdeg02000732         ZP_00318243.1        hypothetical protein
   836301..837053     -  250   48864351            Mdeg02000733         ZP_00318244.1        COG1403: Restriction endonuclease
   837050..838759     -  569   48864352            Mdeg02000734         ZP_00318245.1        COG1049: Aconitase B
-->839696..841903     -  735   48864353            Mdeg02000735         ZP_00318246.1        COG0476: Dinucleotide-utilizing enzymes involved
   841896..843068     -  390   48864354            Mdeg02000736         ZP_00318247.1        COG2333: Predicted hydrolase
   843425..844000     +  191   48864355            Mdeg02000737         ZP_00318248.1        hypothetical protein
   843997..844434     +  145   48864356            Mdeg02000738         ZP_00318249.1        COG2183: Transcriptional accessory protein

  ORGANISM  Xylella fastidiosa Ann-1     accession no is NZ_AAAM03000004.1 gi is 71898141
   cds                dir len   gi         gene     locus                pid                  product
   88367..89761       -  464   71898139            XfasoDRAFT_3703      ZP_00680325.1        Conserved hypothetical protein 730
   90164..91732       -  522   71898140            XfasoDRAFT_3704      ZP_00680326.1        EAL
   92096..92257       +  53    71898183            XfasoDRAFT_3748      ZP_00680369.1        conserved hypothetical protein
-->92781..93920       +  379   71898141            XfasoDRAFT_3705      ZP_00680327.1        UBA/THIF-type NAD/FAD binding
   94196..95593       -  465   71898142            XfasoDRAFT_3706      ZP_00680328.1        Small GTP-binding protein domain:GTP-binding
   95619..96854       -  411   71898143            XfasoDRAFT_3707      ZP_00680329.1        quinoprotein
   96855..97493       -  212   71898144            XfasoDRAFT_3708      ZP_00680330.1        conserved hypothetical protein
---------------------------------------
  ORGANISM  Xylella fastidiosa Ann-1     accession no is NZ_AAAM03000064.1 gi is 71900908
   cds                dir len   gi         gene     locus                pid                  product
   2679..3317         +  212   71900905            XfasoDRAFT_1233      ZP_00683020.1        conserved hypothetical protein
   3318..4553         +  411   71900906            XfasoDRAFT_1234      ZP_00683021.1        quinoprotein
   4579..5976         +  465   71900907            XfasoDRAFT_1235      ZP_00683022.1        Small GTP-binding protein domain:GTP-binding
-->6210..7370         -  386   71900908            XfasoDRAFT_1236      ZP_00683023.1        UBA/THIF-type NAD/FAD binding fold:MoeZ/MoeB
   7887..8048         -  53    71900923            XfasoDRAFT_1251      ZP_00683038.1        conserved hypothetical protein
   8410..9978         +  522   71900909            XfasoDRAFT_1237      ZP_00683024.1        EAL
   10408..11802       +  464   71900910            XfasoDRAFT_1238      ZP_00683025.1        Conserved hypothetical protein 730
---------------------------------------
  ORGANISM  Xylella fastidiosa 9a5c      accession no is AE003897.1 gi is 9105314
   cds                dir len   gi         gene     locus                pid                  product
-->147..1286          -  379   9105314             XF0466               AAF83276.1           molybdopterin biosynthesis protein
   1283..1411         -  42    9105315             XF0467               AAF83277.1           hypothetical protein
   1665..1850         -  61    9105316             XF0468               AAF83278.1           hypothetical protein
   1926..2186         -  86    9105317             XF0469               AAF83279.1           hypothetical protein
---------------------------------------
  ORGANISM  Xylella fastidiosa Temecula1         accession no is NC_004556.1 gi is 77747707
   cds                dir len   gi         gene     locus                pid                  product
   1868421..1868933   -  170   28199492            PD1615               NP_779806.1          hypothetical protein
   1869079..1870473   -  464   28199493            PD1616               NP_779807.1          hypothetical protein
   1870904..1872472   -  522   77747706            PD1617               NP_779808.2          hypothetical protein
-->1873512..1874651   +  379   77747707   moeB     PD1618               NP_779809.2          molybdopterin biosynthesis protein MoeB
   1874892..1876289   -  465   28199496   engA     PD1619               NP_779810.1          GTP-binding protein EngA
   1876315..1877538   -  407   28199497            PD1620               NP_779811.1          serine/threonine protein kinase
   1877551..1878189   -  212   28199498            PD1621               NP_779812.1          hypothetical protein
---------------------------------------
  ORGANISM  Xanthomonas campestris pv. vesicatoria str. 85-10    accession no is AM039952.1 gi is 78036060
   cds                dir len   gi         gene     locus                pid                  product
   2360505..2361722   +  405   78036057            XCV2071              CAJ23748.1           putative quinoprotein
   2361733..2363130   +  465   78036058   engA     XCV2072              CAJ23749.1           GTP-binding protein
   2363433..2364653   +  406   78036059   moeA1    XCV2073              CAJ23750.1           molybdopterin biosynthesis protein MoeA
-->2364993..2366198   -  401   78036060   moeB     XCV2074              CAJ23751.1           molybdopterin biosynthesis protein MoeB
   2366384..2369047   +  887   78036061            XCV2075              CAJ23752.1           TonB-dependent outer membrane receptor
   2369145..2369360   +  71    78036062            XCV2076              CAJ23753.1           conserved hypothetical protein
   2369856..2370434   +  192   78036063            XCV2077              CAJ23754.1           putative secreted protein
---------------------------------------
  ORGANISM  Xanthomonas oryzae pv. oryzae KACC10331      accession no is AE013598.1 gi is 58426731
   cds                dir len   gi         gene     locus                pid                  product
   2658710..2659150   -  146   58426728            XOO2511              AAW75765.1           putative transposase
   2659108..2659767   -  219   58426729            XOO2512              AAW75766.1           putative transposase
   2659802..2661184   +  460   58426730            XOO2513              AAW75767.1           putative ISXo8 transposase
-->2661157..2662575   +  472   58426731   moeB     XOO2514              AAW75768.1           molybdopterin biosynthesis protein
   2662572..2662718   -  48    58426732            XOO2515              AAW75769.1           Transposase and inactivated derivatives
   2662801..2663817   +  338   58426733            XOO2516              AAW75770.1           transposase
   2663795..2665270   -  491   58426734            XOO2517              AAW75771.1           IS1478 transposase
---------------------------------------
  ORGANISM  Xanthomonas axonopodis pv. citri str. 306    accession no is AE011838.1 gi is 21108248
   cds                dir len   gi         gene     locus                pid                  product
   187..1407          +  406   21108247   moeA                          AAM36884.1           molybdopterin biosynthesis
-->1759..2901         -  380   21108248   moeB                          AAM36885.1           molybdopterin biosynthesis protein
   3211..5811         +  866   21108249   cirA                          AAM36886.1           TonB-dependent receptor
   5870..6109         +  79    21108250   XAC2025                       AAM36887.1           conserved hypothetical protein
   6488..7060         +  190   21108251   XAC2026                       AAM36888.1           conserved hypothetical protein
---------------------------------------
  ORGANISM  Xanthomonas oryzae pv. oryzae MAFF 311018    accession no is AP008229.1 gi is 84367975
   cds                dir len   gi         gene     locus                pid                  product
   2635865..2636305   -  146   84367972   XOO2375                       BAE69130.1           ISXoo2 transposase
   2636263..2636922   -  219   84367973   XOO2376                       BAE69131.1           ISXoo2 transposase
   2637020..2638339   +  439   84367974   XOO2377                       BAE69132.1           ISXo8 transposase
-->2638591..2639730   +  379   84367975   XOO2378                       BAE69133.1           molybdopterin biosynthesis protein
   2639974..2640972   +  332   84367976   XOO2379                       BAE69134.1           ISXo5 transposase
   2640950..2642341   -  463   84367977   XOO2380                       BAE69135.1           ISXoo4 transposase
   2643112..2644161   +  349   84367978   XOO2381                       BAE69136.1           ISXoo8 transposase
---------------------------------------
  ORGANISM  Xanthomonas campestris pv. campestris str. ATCC 33913        accession no is AE012303.1 gi is 21113107
   cds                dir len   gi         gene     locus                pid                  product
   4145..5365         +  406   21113104   XCC1986                       AAM41275.1           conserved hypothetical protein
   5376..6773         +  465   21113105   engA                          AAM41276.1           GTP-binding protein
   6805..8025         +  406   21113106   moeA                          AAM41277.1           molybdopterin biosynthesis
-->8036..9172         -  378   21113107   moeB                          AAM41278.1           molybdopterin biosynthesis protein
   9486..12104        +  872   21113108   cirA                          AAM41279.1           TonB-dependent receptor
---------------------------------------
  ORGANISM  Nostoc sp. PCC 7120  accession no is AP003602.1 gi is 17134589
   cds                dir len   gi         gene     locus                pid                  product
   186374..186614,1..116) -  118   17134772   all7501                       BAB77330.1           transcriptional regulator
   153..740           +  195   17134587   alr7502                       BAB77145.1
   744..1124          +  126   17134588   alr7503                       BAB77146.1
-->1298..2548         +  416   17134589   alr7504                       JAB+E1, BAB77147.1
   2552..2911         +  119   17134590   alr7505                       BAB77148.1
   3368..4456         +  362   17134591   alr7506                       BAB77149.1
   4437..4931         +  164   17134592   alr7507                       BAB77150.1
---------------------------------------
  ORGANISM  Synechocystis sp. PCC 6803   accession no is AP006585.1 gi is 38423902
   cds                dir len   gi         gene     locus                pid                  product
   47827..51129       +  1100  38423899   slr6050                       BAD02107.1
   51133..51513       +  126   38423900   slr6051                       BAD02108.1
   51619..52014       -  131   38423901   sll6052                       BAD02109.1
-->51986..53401       -  471   38423902   sll6053                       JAB+E1, BAD02110.1
   53398..53781       -  127   38423903   sll6054                       BAD02111.1
   53782..54240       -  152   38423904   sll6055                       BAD02112.1
   54343..54702       +  119   38423905   slr6056                       BAD02113.1
---------------------------------------
  ORGANISM  Burkholderia vietnamiensis G4        accession no is NZ_AAEH02000035.1 gi is 67547439
   cds                dir len   gi         gene     locus                pid                  product
   49758..50393       +  211   67547438            Bcep1808DRAFT_3226   ZP_00425341.1        hypothetical protein
   50369..50653       +  94    67547471            Bcep1808DRAFT_3259   ZP_00425374.1        hypothetical protein
   50943..51254       +  103   67547472            Bcep1808DRAFT_3260   ZP_00425375.1        hypothetical protein
-->52431..53837       -  468   67547439            Bcep1808DRAFT_3227   ZP_00425342.1        JAB+E1, UBA/THIF-type NAD/FAD binding fold
   53834..54586       -  250   67547440            Bcep1808DRAFT_3228   ZP_00425343.1        hypothetical protein
   54741..55076       +  111   67547473            Bcep1808DRAFT_3261   ZP_00425376.1        Helix-turn-helix motif
   55084..55641       +  185   67547474            Bcep1808DRAFT_3262   ZP_00425377.1        Protein of unknown function DUF955
---------------------------------------
  ORGANISM  Polaromonas naphthalenivorans CJ2    accession no is NZ_AANM01000003.1 gi is 84711629
   cds                dir len   gi         gene     locus                pid                  product
   197097..197690     +  197   84711745            PnapDRAFT_4067       ZP_01019891.1        conserved hypothetical protein
   197693..201118     +  1141  84711627            PnapDRAFT_3949       ZP_01019773.1        putative ATP-dependent DNA helicase
   201211..202140     +  309   84711628            PnapDRAFT_3950       ZP_01019774.1        hypothetical protein
-->202137..203546     +  469   84711629            PnapDRAFT_3951       ZP_01019775.1        JAB+E1,
   205134..205556     -  140   84711746            PnapDRAFT_4068       ZP_01019892.1        conserved hypothetical protein
   205516..206157     -  213   84711630            PnapDRAFT_3952       ZP_01019776.1        similar to Transcriptional activator of
   206365..207003     -  212   84711631            PnapDRAFT_3953       ZP_01019777.1        Maleylacetoacetate isomerase
---------------------------------------
  ORGANISM  Nitrobacter hamburgensis X14         accession no is NZ_AAIS01000003.1 gi is 69928900
   cds                dir len   gi         gene     locus                pid                  product
   114424..114846     -  140   69929315            NhamDRAFT_2318       ZP_00626329.1        hypothetical protein
   114843..115421     -  192   69928898            NhamDRAFT_1901       ZP_00625912.1        hypothetical protein
   115378..116667     +  429   69928899            NhamDRAFT_1902       ZP_00625913.1        hypothetical protein
-->116664..118040     +  458   69928900            NhamDRAFT_1903       ZP_00625914.1        UBA/THIF-type NAD/FAD binding fold
   118522..119460     +  312   69928901            NhamDRAFT_1904       ZP_00625915.1        hypothetical protein
   119493..121838     -  781   69928902            NhamDRAFT_1905       ZP_00625916.1        unknown protein
   121842..122909     -  355   69928903            NhamDRAFT_1906       ZP_00625917.1        hypothetical protein
---------------------------------------
  ORGANISM  Frankia sp. CcI3     accession no is NC_007777.1 gi is 86742694
   cds                dir len   gi         gene     locus                pid                  product
   4796627..4797937   -  436   86742691            Francci3_4012        YP_483091.1          restriction modification system DNA specificity
   4798193..4799617   -  474   86742692            Francci3_4013        YP_483092.1          transposase, IS4
   4799751..4802201   -  816   86742693            Francci3_4014        YP_483093.1          N-6 DNA methylase
-->4802257..4803687   -  476   86742694            Francci3_4015        YP_483094.1          JAB+ UBA/THIF-type NAD/FAD binding fold
   4803669..4804367   -  232   86742695            Francci3_4016        YP_483095.1          hypothetical protein
   4804477..4804851   +  124   86742696            Francci3_4017        YP_483096.1          transcriptional regulator, XRE family
   4804852..4805436   +  194   86742697            Francci3_4018        YP_483097.1          protein of unknown function DUF955
---------------------------------------
  ORGANISM  Anaeromyxobacter dehalogenans 2CP-C  accession no is NC_007760.1 gi is 86159351
   cds                dir len   gi         gene     locus                pid                  product
   3301025..3303004   -  659   86159348            Adeh_2926            YP_466133.1          Polysaccharide deacetylase
   3303413..3305362   +  649   86159349            Adeh_2927            YP_466134.1          hypothetical protein
   3305527..3306870   +  447   86159350            Adeh_2928            YP_466135.1          peptidase M48, Ste24p
-->3306876..3308690   -  604   86159351            Adeh_2929            YP_466136.1          JAB+ UBA/THIF-type NAD/FAD binding protein
   3308928..3309425   -  165   86159352            Adeh_2930            YP_466137.1          hypothetical protein
   3309380..3310270   -  296   86159353            Adeh_2931            YP_466138.1          protein of unknown function DUF815
   3310355..3311164   -  269   86159354            Adeh_2932            YP_466139.1          4Fe-4S ferredoxin, iron-sulfur binding protein
---------------------------------------

  ORGANISM  Chlorobium tepidum TLS       accession no is AE006470.1 gi is 21646639
   cds                dir len   gi         gene     locus                pid                  product
   682844..683911     -  355   21646636   thiH     CT0697               AAM71934.1           ThiH protein
   683908..684687     -  259   21646637   thiG     CT0698               AAM71935.1           ThiG protein
   684722..685039     -  105   21646638   thiS     CT0699               AAM71936.1           thiamine biosynthesis protein ThiS
-->685297..686229     -  310   21646639   cysK     CT0700               AAM71937.1           cysteine synthase
   686241..687383     -  380   21646640            CT0701               AAM71938.1           trans-sulfuration enzyme family protein
   687637..687822     -  61    21646641            CT0702               AAM71939.1           hypothetical protein
   687859..688518     -  219   66576269            CT0703               AAM71940.2           trans-sulfuration enzyme family protein
---------------------------------------
  ORGANISM  Pelobacter carbinolicus DSM 2380     accession no is CP000142.1 gi is 77545399
   cds                dir len   gi         gene     locus                pid                  product
   2001694..2003196   -  500   77545396            Pcar_1715            ABA88958.1           cobalt-zinc-cadmium resistance protein (CzcB)
   2003426..2004787   -  453   77545397            Pcar_1716            ABA88959.1           metal ion efflux outer membrane protein family
   2005104..2005496   -  130   77545398            Pcar_1717            ABA88960.1           hypothetical protein
-->2005646..2006572   -  308   77545399            Pcar_1718            ABA88961.1           cysteine synthase
   2006649..2007938   -  429   77545400            Pcar_1719            ABA88962.1           O-acetylhomoserine (thiol)-lyase
   2008260..2009078   -  272   77545401            Pcar_1720            ABA88963.1           Dinucleotide-utilizing enzymes involved in
   2009081..2009305   -  74    77545402            Pcar_1721            ABA88964.1           molybdopterin converting factor, subunit 1-like
---------------------------------------
  ORGANISM  Syntrophus aciditrophicus SB         accession no is NC_007759.1 gi is 85859830
   cds                dir len   gi         gene     locus                pid                  product
   2064115..2065056   +  313   85859827            SYN_00443            YP_462029.1          hypothetical membrane protein
   2065139..2065354   -  71    85859829            SYN_00444            YP_462030.1          hypothetical cytosolic protein
   2065344..2066324   +  326   85859828            SYN_00445            YP_462031.1          1,4-dihydroxy-2-naphthoate
-->2066687..2067574   +  295   85859830            SYN_00446            YP_462032.1          cysteine synthase
   2067531..2068139   +  202   85859831            SYN_00447            YP_462033.1          hypothetical membrane protein
   2068264..2070417   -  717   85859832            SYN_00448            YP_462034.1          general secretion pathway protein D
   2070354..2071001   -  215   85859833            SYN_00449            YP_462035.1          lytic transglycosylase -like protein
---------------------------------------

  ORGANISM  Anabaena variabilis ATCC 29413       accession no is CP000121.1 gi is 75705484
   cds                dir len   gi         gene     locus                pid                  product
   94784..95266       +  160   75705481            Ava_C0064            ABA25153.1           conserved hypothetical protein
   95272..95694       +  140   75705482            Ava_C0065            ABA25154.1           conserved hypothetical protein
   95738..96466       +  242   75705483            Ava_C0066            ABA25155.1           conserved hypothetical protein
-->96463..97185       +  240   75705484            Ava_C0067            ABA25156.1           conserved hypothetical protein
   97188..97784       +  198   75705485            Ava_C0068            ABA25157.1           conserved hypothetical protein
   97772..98629       +  285   75705486            Ava_C0069            ABA25158.1           UBA/THIF-type NAD/FAD binding fold
   98674..99357       +  227   75705487            Ava_C0070            ABA25159.1           conserved hypothetical protein
---------------------------------------
  ORGANISM  Nostoc sp. PCC 7120  accession no is AP003602.1 gi is 17134644
   cds                dir len   gi         gene     locus                pid                  product
   52590..53072       +  160   17134641   alr7556                       BAB77199.1
   53078..53500       +  140   17134642   alr7557                       BAB77200.1
   53544..54272       +  242   17134643   alr7558                       BAB77201.1
-->54269..54991       +  240   17134644   alr7559                       BAB77202.1
   54994..55590       +  198   17134645   alr7560                       BAB77203.1
   55578..56369       +  263   17134646   alr7561                       BAB77204.1
   56493..57710       +  405   17134647   alr7562                       BAB77205.1
---------------------------------------
  ORGANISM  Bacteroides thetaiotaomicron VPI-5482        accession no is AE015928.1 gi is 29339960
   cds                dir len   gi         gene     locus                pid                  product
   3298565..3299647   +  360   29339957            BT2645               AAO77752.1           hypothetical protein
   3299669..3299890   +  73    29339958            BT2646               AAO77753.1           conserved hypothetical protein
   3299935..3301041   +  368   29339959            BT2647               AAO77754.1           hypothetical protein
-->3301054..3301755   +  233   29339960            BT2648               AAO77755.1           hypothetical protein
   3301752..3302549   +  265   29339961            BT2649               AAO77756.1           ThiF family protein, ubiquitin-activating
   3302647..3303348   -  233   29339962            BT2650               AAO77757.1           conserved hypothetical protein
   3303406..3303699   -  97    29339963            BT2651               AAO77758.1           conserved hypothetical protein
---------------------------------------
  ORGANISM  Pelobacter propionicus DSM 2379      accession no is NZ_AAJH01000023.1 gi is 71839550
   cds                dir len   gi         gene     locus                pid                  product
   12573..12929       +  118   71839547            PproDRAFT_0254       ZP_00679294.1        hypothetical protein
   12945..13148       +  67    71839548            PproDRAFT_0255       ZP_00679295.1        conserved hypothetical protein
   13333..14328       +  331   71839549            PproDRAFT_0256       ZP_00679296.1        hypothetical protein
-->14360..15091       +  243   71839550            PproDRAFT_0257       ZP_00679297.1        conserved hypothetical protein
   15088..15894       +  268   71839551            PproDRAFT_0258       ZP_00679298.1        UBA/THIF-type NAD/FAD binding fold
   15939..17480       +  513   71839519            PproDRAFT_0226       ZP_00679266.1        similar to Superfamily I DNA and RNA helicases
   17477..18103       +  208   71839552            PproDRAFT_0259       ZP_00679299.1        conserved hypothetical protein
---------------------------------------
  ORGANISM  Bacillus thuringiensis serovar israelensis ATCC 35646        accession no is NZ_AAJM01000003.1 gi is 75758403
   cds                dir len   gi         gene     locus                pid                  product
   35659..37797       -  712   75758400            RBTH_06712           ZP_00738523.1        hypothetical protein
   38210..39052       -  280   75758401            RBTH_06713           ZP_00738524.1        Hypothetical protein
   39057..39533       -  158   75758402            RBTH_06714           ZP_00738525.1        hypothetical protein
-->39571..40296       -  241   75758403            RBTH_06715           ZP_00738526.1        hypothetical protein
   40277..40831       -  184   75758404            RBTH_06716           ZP_00738527.1        hypothetical protein
   41908..42183       -  91    75758405            RBTH_06719           ZP_00738528.1        Transcription state regulatory protein abrB
   42447..42869       -  140   75758406            RBTH_06720           ZP_00738529.1        hypothetical protein
---------------------------------------
  ORGANISM  Bacillus thuringiensis serovar israelensis ATCC 35646        accession no is NZ_AAJM01000016.1 gi is 75758953
   cds                dir len   gi         gene     locus                pid                  product
   8370..9026         -  218   75758950            RBTH_07329           ZP_00739060.1        hypothetical protein
   9040..9861         -  273   75758951            RBTH_07328           ZP_00739061.1        Hypothetical protein
   9975..11165        -  396   75758952            RBTH_07327           ZP_00739062.1        hypothetical protein
-->11323..12084       -  253   75758953            RBTH_07326           ZP_00739063.1        hypothetical protein
   12100..13119       -  339   75758954            RBTH_07325           ZP_00739064.1        hypothetical protein
   14188..14928       -  246   75758955            RBTH_07322           ZP_00739065.1        hypothetical protein
   15798..16277       -  159   75758956            RBTH_07319           ZP_00739066.1        Arsenate reductase
---------------------------------------

  ORGANISM  Yersinia pestis KIM  accession no is AF053947.1 gi is 2996351
   cds                dir len   gi         gene     locus                pid                  product
   2932..5514         -  860   2996320                                  AAC13200.1           tail fiber protein homolog
   5901..6518         -  205   2996331                                  AAC13211.1           unknown
   6571..11049        -  1492  2996342                                  AAC13222.1           phage lambda host specific protein J
-->11188..11739       -  183   2996351                                  AAC13231.1           unknown
   11763..12521       -  252   2996362                                  AAC13242.1           unknown
   12553..13251       -  232   2996365                                  AAC13245.1           phage lambda minor tail protein L homolog
   13341..13676       -  111   2996289                                  AAC13169.1           unknown
---------------------------------------
  ORGANISM  Phage BP-4795        accession no is AJ556162.1 gi is 76556246
   cds                dir len   gi         gene     locus                pid                  product
   41004..41345       +  113   49523651                                 CAD88869.1           putative minor tail protein
   41345..42043       +  232   49523652                                 CAD88870.1           putative tail fiber component
   41916..42797       +  293   71794525                                 CAD88871.2           JAB+NlpC; putative tail fiber component
-->42695..43375       +  226   76556246                                 CAD88872.2           ThiS, putative tail component
   43622..47098       +  1158  49523655                                 CAD88873.1           putative tail component
   47165..47764       +  199   49523656   lom                           CAD88874.1           outer membrane protein Lom precursor
   47765..47938       -  57    49523657                                 CAD88875.1           hypothetical protein
---------------------------------------
  ORGANISM  Bacteriophage JK06   accession no is NC_007291.1 gi is 71834086
   cds                dir len   gi         gene     locus                pid                  product
   336..1307          +  323   71834083            JK_1                 YP_277441.1          hypothetical protein
   1338..4763         -  1141  71834084            JK_2                 YP_277442.1          tail fiber
-->4805..5380         -  191   71834086            JK_4                 YP_277443.1          hypothetical tail assembly protein I
   5010..5390         +  126   71834085            JK_3                 YP_277444.1          hypothetical protein
   5352..6155         -  267   71834088            JK_6                 YP_277445.1          hypothetical GP19
   5654..5914         -  86    71834087            JK_5                 YP_277446.1          hypothetical protein
---------------------------------------
  ORGANISM  Yersinia pestis      accession no is NC_006323.1 gi is 52788057
   cds                dir len   gi         gene     locus                pid                  product
   987..3923          -  978   52788054                                 YP_093882.1          phage lambda-related protein
   3956..4432         -  158   52788055                                 YP_093883.1          hypothetical protein
   4320..4589         -  89    52788056                                 YP_093884.1          hypothetical protein
-->9246..9833         -  195   52788057                                 YP_093885.1          phage lambda tail assembly protein I
   9821..10645        -  274   52788058                                 YP_093886.1          JAB+NlpC; putative phage tail protein
   10611..11342       -  243   52788059                                 YP_093887.1          phage lambda-related tail protein L
   11399..11734       -  111   52788060                                 YP_093888.1          phage lambda-related tail protein M
---------------------------------------
  ORGANISM  Bacteriophage RTP    accession no is AM156909.1 gi is 80750693
   cds                dir len   gi         gene     locus                pid                  product
   20641..21396       +  251   80750690   rtp39                         CAJ42243.1           putative minor tail protein
   21464..21979       +  171   80750691   rtp40                         CAJ42244.1           putative HNH endonuclease
   21960..22718       +  252   80750692   rtp41                         CAJ42245.1           JAB+NlpC; putative minor tail protein
-->22699..23271       +  190   80750693   rtp42                         CAJ42246.1           putative tail assembly protein
   23313..26723       +  1136  80750694   rtp43                         CAJ42247.1           putative tail fiber protein
   26752..27687       -  311   80750695   rtp44                         CAJ42248.1           hypothetical protein
   27687..27917       -  76    80750696   rtp45                         CAJ42249.1           putative phage lipoprotein
---------------------------------------
  ORGANISM  Bacteriophage phiKO2         accession no is NC_005857.1 gi is 46402106
   cds                dir len   gi         gene     locus                pid                  product
   12788..13543       +  251   46402103            phiKO2p17            YP_006597.1          Protein A; Gp17
   13545..14255       +  236   46402104            phiKO2p18            YP_006598.1          JAB+NlpC; Gp18
   14292..14627       +  111   46402105            phiKO2p19            YP_006599.1          Gp19
-->14673..15266       +  197   46402106            phiKO2p20            YP_006600.1          Gp20
   15332..25633       +  3433  46402107            phiKO2p21            YP_006601.1          large protein with host_specificity protein J
   25707..25949       -  80    46402108            phiKO2p22            YP_006602.1          Gp22
   26027..26410       +  127   46402109            phiKO2p23            YP_006603.1          Gp23
---------------------------------------
  ORGANISM  Bacteriophage phiE125        accession no is NC_003309.1 gi is 17975181
   cds                dir len   gi         gene     locus                pid                  product
   13695..15083       +  462   17975178            phiE125p17           NP_536373.1          hypothetical protein
   15080..15763       +  227   17975179            phiE125p18           NP_536374.1          putative minor tail protein
   15783..16565       +  260   17975180            phiE125p19           NP_536375.1          JAB+NlpC; putative tail component protein
-->16562..17146       +  194   17975181            phiE125p20           NP_536376.1          putative tail component protein
   17143..20448       +  1101  17975182            phiE125p21           NP_536377.1          putative tail tip fiber protein
   20445..20759       +  104   17975183            phiE125p22           NP_536378.1          hypothetical protein
   20759..21493       +  244   17975184            phiE125p23           NP_536379.1          hypothetical protein
---------------------------------------
  ORGANISM  Yersinia pestis CO92         accession no is AJ414151.1 gi is 15980136
   cds                dir len   gi         gene     locus                pid                  product
   128149..129243     +  364   15980133   YPO2126                       CAC90937.1           BroN-; putative phage protein
   129342..129893     +  183   15980134   YPO2127                       CAC90938.1           zinc ribbon; putative phage-related membrane protein
   129901..130251     +  116   15980135   YPO2128                       CAC90939.1           ? putative phage-related lipoprotein
-->130307..130927     +  206   15980136   YPO2129                       CAC90940.1           putative phage tail assembly protein
   131102..131413     -  103   15980137   YPO2130                       CAC90941.1           hypothetical phage protein
   131499..134702     +  1067  15980138   YPO2131                       CAC90942.1           putative phage host specificity protein
   134702..135700     +  332   15980139   YPO2132                       CAC90943.1           hypothetical phage protein
---------------------------------------
  ORGANISM  Shigella sonnei Ss046        accession no is NC_007384.1 gi is 74312266
   cds                dir len   gi         gene     locus                pid                  product
   1865254..1865583   -  109   74312263            SSO_1760             YP_310682.1          IS21 ORF1
   1866060..1866659   -  199   74312264            SSO_1762             YP_310683.1          putative membrane protein precursor
   1866727..1870206   -  1159  74312265            SSO_1763             YP_310684.1          host specificity protein
-->1870267..1870809   -  180   74312266            SSO_1764             YP_310685.1          putative tail component of prophage
   1870806..1871549   -  247   74312267            SSO_1765             YP_310686.1          JAB+NlpC;  tail assembly protein
   1871554..1872252   -  232   74312268            SSO_1766             YP_310687.1          minor tail protein
   1872262..1872591   -  109   74312269            SSO_1767             YP_310688.1          putative minor tail protein
---------------------------------------
  ORGANISM  Shigella sonnei Ss046        accession no is NC_007384.1 gi is 74312870
   cds                dir len   gi         gene     locus                pid                  product
   2545274..2548363   -  1029  74312867            SSO_2410             YP_311286.1          STF phage protein-related
   2548456..2548938   -  160   74312868            SSO_2411             YP_311287.1          putative outer membrane protein
   2549009..2552506   -  1165  74312869            SSO_2412             YP_311288.1          putative host specificity protein
-->2552567..2553199   -  210   74312870            SSO_2413             YP_311289.1          putative tail component of prophage
   2553136..2553879   -  247   74312871            SSO_2414             YP_311290.1          JAB+NlpC;  tail assembly protein
   2553885..2554583   -  232   74312872            SSO_2415             YP_311291.1          putative minor tail protein
   2554583..2554912   -  109   74312873            SSO_2416             YP_311292.1          putative minor tail protein
---------------------------------------
  ORGANISM  Shigella boydii Sb227        accession no is NC_007613.1 gi is 82543715
   cds                dir len   gi         gene     locus                pid                  product
   1190211..1191065   -  284   82543712            SBO_1196             YP_407659.1          putative tail component encoded by cryptic
   1191130..1191729   -  199   82543713            SBO_1197             YP_407660.1          putative membrane protein precursor
   1191797..1195270   -  1157  82543714            SBO_1198             YP_407661.1          host specificity protein
-->1195511..1196125   -  204   82543715            SBO_1199             YP_407662.1          putative tail component
   1196089..1196907   -  272   82543716            JAB+NlpC;  SBO_1200             YP_407663.1          tail assembly protein
   1196843..1197541   -  232   82543717            SBO_1201             YP_407664.1          minor tail protein
   1197875..1201036   -  1053  82543718            SBO_1202             YP_407665.1          putative tail length tape measure protein
---------------------------------------
  ORGANISM  Shigella boydii BS512        accession no is NZ_AAKA01000030.1 gi is 75175531
   cds                dir len   gi         gene     locus                pid                  product
   15016..15297       -  93    75175528            SboyB_01004419       ZP_00695744.1        hypothetical protein
   15294..15605       -  103   75175529            SboyB_01004420       ZP_00695745.1        COG1629: Outer membrane receptor proteins,
   15673..19080       -  1135  75175530            SboyB_01004421       ZP_00695746.1        COG4733: Phage-related protein, tail component
-->19386..19967       -  193   75175531            SboyB_01004422       ZP_00695747.1        COG4723: Phage-related protein, tail component
   19964..20707       -  247   75175532            JAB+NlpC;  SboyB_01004423       ZP_00695748.1        COG0791: Cell wall-associated hydrolases
   20718..21416       -  232   75175533            SboyB_01004424       ZP_00695749.1        COG4672: Phage-related protein
   21416..21757       -  113   75175534            SboyB_01004425       ZP_00695750.1        COG4718: Phage-related protein
---------------------------------------
  ORGANISM  Shewanella sp. W3-18-1       accession no is NZ_AALN01000124.1 gi is 82743846
   cds                dir len   gi         gene     locus                pid                  product
   162..587           -  141   82743845            Sputw3181DRAFT_0534  ZP_00906464.1        hypothetical protein
   728..967           -  79    82743860            Sputw3181DRAFT_0549  ZP_00906479.1        hypothetical protein
-->2188..2904         +  238   82743846            Sputw3181DRAFT_0535  ZP_00906465.1        prophage LambdaSo, tail assembly protein I
   2964..3860         -  298   82743847            Sputw3181DRAFT_0536  ZP_00906466.1        DNA polymerase III, epsilon subunit
   3873..4295         -  140   82743848            Sputw3181DRAFT_0537  ZP_00906467.1        conserved hypothetical protein
   4330..4989         -  219   82743849            Sputw3181DRAFT_0538  ZP_00906468.1        Helix-turn-helix motif
---------------------------------------
  ORGANISM  Shewanella sp. W3-18-1       accession no is NZ_AALN01000024.1 gi is 82741527
   cds                dir len   gi         gene     locus                pid                  product
   11900..12610       -  236   82741525            Sputw3181DRAFT_2544  ZP_00904244.1        conserved hypothetical protein
   12614..12937       -  107   82741558            Sputw3181DRAFT_2577  ZP_00904277.1        hypothetical protein
   12934..19020       -  2028  82741526            Sputw3181DRAFT_2545  ZP_00904245.1        Carbohydrate-binding family V/XII
-->19143..19793       -  216   82741527            Sputw3181DRAFT_2546  ZP_00904246.1        prophage LambdaSo, tail assembly protein I
   19914..20804       -  296   82741528            Sputw3181DRAFT_2547  ZP_00904247.1        BroN, similar to Uncharacterized phage-encoded
   20801..21475       -  224   82741529            Sputw3181DRAFT_2548  ZP_00904248.1        DNA-binding protein
   21753..22244       +  163   82741530            Sputw3181DRAFT_2549  ZP_00904249.1        hypothetical protein
---------------------------------------
  ORGANISM  Shewanella oneidensis MR-1   accession no is NC_004347.1 gi is 24374467
   cds                dir len   gi         gene     locus                pid                  product
   3074866..3075408   +  180   24374464            SO2938               NP_718507.1          hypothetical protein
   3075409..3076119   -  236   24374465            SO2939               NP_718508.1          hypothetical protein
   3076443..3080468   -  1341  24374466            SO2940               NP_718509.1          prophage LambdaSo, host specificity protein J,
-->3080473..3081102   -  209   24374467            SO2941               NP_718510.1          prophage LambdaSo, tail assembly protein I
   3081179..3081745   -  188   24374468            SO2942               NP_718511.1          hypothetical protein
   3081823..3082080   -  85    24374469            SO2943               NP_718512.1          hypothetical protein
   3082148..3083743   -  531   24374470            SO2944               NP_718513.1          hypothetical protein
---------------------------------------
  ORGANISM  Salmonella typhimurium LT2   accession no is AE008743.2 gi is 16419562
   cds                dir len   gi         gene     locus                pid                  product
   45243..45776       -  177   16419559   sodC                          AAL19978.1           Gifsy-2 prophage superoxide dismutase precursor
   45866..46561       +  231   16419560   STM1045                       AAL19979.1           Gifsy-2 prophage probable minor tail protein
   46571..47308       +  245   16419561   STM1046                       AAL19980.1           JAB+NlpC; Gifsy-2 prophage probable tail assembly protein
-->47206..47910       +  234   16419562   STM1047                       AAL19981.1           Gifsy-2 prophage probable tail assembly protein
   47982..50429       +  815   16419563   STM1048                       AAL19982.1           Gifsy-2 prophage host specificity protein J,
   51666..54104       +  812   16419564   STM1049                       AAL19983.1           Gifsy-2 prophage probable tail fiber protein
   54104..54685       +  193   16419565   STM1050                       AAL19984.1           Gifsy-2 prophage tail fiber assembly
---------------------------------------
  ORGANISM  Salmonella typhimurium LT2   accession no is AE008737.2 gi is 16419434
   cds                dir len   gi         gene     locus                pid                  product
   42739..43257       +  172   16419431   STM0920                       AAL19856.1           Fels-1 prophage attachment and invasion protein
   43389..44084       +  231   16419432   STM0921                       AAL19857.1           putative Fels-1 prophage minor tail protein
   44096..44830       +  244   16419433   STM0922                       AAL19858.1           putative Fels-1 prophage tail assembly protein
-->44728..45405       +  225   16419434   STM0923                       AAL19859.1           putative Fels-1 prophage tail assembly protein
   45459..45983       -  174   16419435   STM0924                       AAL19860.1           putative Fels-1 prophage Cu/Zn superoxide
   46077..49523       +  1148  16419436   STM0925                       AAL19861.1           putative Fels-1 prophage host specificity
   49567..51939       +  790   16419437   STM0926                       AAL19862.1           putative Fels-1 prophage minor tail protein
---------------------------------------
  ORGANISM  Enterobacteria phage HK022   accession no is NC_002166.1 gi is 9634139
   cds                dir len   gi         gene     locus                pid                  product
   11233..11988       +  251   9634136             HK022p17             NP_037678.1          Protein_A; gp18
   11990..12700       +  236   9634137             HK022p18             NP_037679.1          JAB+NlpC; gp19
   12748..12972       +  74    9634138             HK022p19             NP_037680.1          gp20
-->13022..13630       +  202   9634139             HK022p20             NP_037681.1          gp21
   13652..13831       +  59    9634140             HK022p21             NP_037682.1          gp22
   13751..13972       -  73    9634141             HK022p22             NP_037683.1          srb protein
   14137..17688       +  1183  9634142             HK022p23             NP_037684.1          gp24
---------------------------------------
  ORGANISM  Enterobacteria phage lambda  accession no is J02459.1 gi is 215124
   cds                dir len   gi         gene     locus                pid                  product
   13100..13429       +  109   215121       gpM                            AAA96549.1
   13429..14127       +  232   215122       gpL                            AAA96550.1
   14276..14875       +  199   215123       gpK                            JAB+NlpC;  AAA96551.1
-->14773..15444       +  223   215124       gpI ThiS                           AAA96552.1
   15505..18903       +  1132  215125       gpJ                            AAA96553.1
   18965..19585       +  206   215126                                   AAA96554.1
   19650..20855       +  401   215127                                   AAA96555.1
---------------------------------------
  ORGANISM  Enterobacteria phage N15     accession no is NC_001901.1 gi is 9630484
   cds                dir len   gi         gene     locus                pid                  product
   12880..13227       +  115   9630481             N15p17               NP_046912.1          gp17
   13224..13979       +  251   9630482             N15p18               NP_046913.1          gp18
   13981..14712       +  243   9630483             N15p19               NP_046914.1          gp19
-->14712..15290       +  192   9630484             N15p20               NP_046915.1          gp20
   15342..18527       +  1061  9630485             N15p21               NP_046916.1          gp21
   18527..18829       +  100   9630486             N15p22               NP_046917.1          gp22
   18829..19506       +  225   9630487             N15p23               NP_046918.1          gp23
---------------------------------------
  ORGANISM  Enterobacteria phage T1      accession no is NC_005833.1 gi is 45686326
   cds                dir len   gi         gene     locus                pid                  product
   22423..22776       +  117   45686323            T1p37                YP_003908.1          putative minor tail protein
   22856..23638       +  260   45686324            T1p36                YP_003909.1          putative minor tail protein
   23635..24369       +  244   45686325            T1p35                YP_003910.1          putative minor tail protein
-->24366..24965       +  199   45686326            T1p34                YP_003911.1          putative tail assembly protein
   25043..28561       +  1172  45686327            T1p33                YP_003912.1          putative tail fiber protein
   28607..28912       +  101   45686328            T1p32                YP_003913.1          hypothetical protein
   28912..29601       +  229   45686329            T1p31                YP_003914.1          hypothetical protein
---------------------------------------
  ORGANISM  Escherichia coli 101-1       accession no is NZ_AAMK01000006.1 gi is 83587164
   cds                dir len   gi         gene     locus                pid                  product
   68457..68786       +  109   83587161            Ecol1_01001548       ZP_00925790.1        COG4718: Phage-related protein
   68786..69484       +  232   83587162            Ecol1_01001549       ZP_00925791.1        COG4672: Phage-related protein
   69634..70233       +  199   83587163            Ecol1_01001550       ZP_00925792.1        COG0791: Cell wall-associated hydrolases
-->70230..70802       +  190   83587164            Ecol1_01001551       ZP_00925793.1        COG4723: Phage-related protein, tail component
   70863..71204       +  113   83587165            Ecol1_01001552       ZP_00925794.1        COG4733: Phage-related protein, tail component
   71201..73057       +  618   83587166            Ecol1_01001553       ZP_00925795.1        COG4733: Phage-related protein, tail component
   73125..73724       +  199   83587167            Ecol1_01001554       ZP_00925796.1        hypothetical protein
---------------------------------------
  ORGANISM  Escherichia coli B171        accession no is NZ_AAJX01000143.1 gi is 75207839
   cds                dir len   gi         gene     locus                pid                  product
   2..448             +  148   75207838            EcolB_01004797       ZP_00708319.1        NlpC;  COG0791: Cell wall-associated hydrolases
-->445..987           +  180   75207839            EcolB_01004798       ZP_00708320.1        COG4723: Phage-related protein, tail component
   1660..2310         +  216   75207840            EcolB_01004800       ZP_00708321.1        COG2963: Transposase and inactivated
---------------------------------------
  ORGANISM  Escherichia coli B171        accession no is NZ_AAJX01000049.1 gi is 75208766
   cds                dir len   gi         gene     locus                pid                  product
   6441..6770         +  109   75208763            EcolB_01004022       ZP_00709052.1        COG4718: Phage-related protein
   6770..7468         +  232   75208764            EcolB_01004023       ZP_00709053.1        COG4672: Phage-related protein
   7473..8216         +  247   75208765            EcolB_01004024       ZP_00709054.1        JAB+NlpC;  COG0791: Cell wall-associated hydrolases
-->8213..8755         +  180   75208766            EcolB_01004025       ZP_00709055.1        COG4723: Phage-related protein, tail component
   8816..12211        +  1131  75208767            EcolB_01004026       ZP_00709056.1        COG4733: Phage-related protein, tail component
   12279..12878       +  199   75208768            EcolB_01004027       ZP_00709057.1        hypothetical protein
   12943..14256       +  437   75208769            EcolB_01004028       ZP_00709058.1        hypothetical protein
---------------------------------------
  ORGANISM  Escherichia coli B171        accession no is NZ_AAJX01000012.1 gi is 75210818
   cds                dir len   gi         gene     locus                pid                  product
   89459..92374       -  971   75210815            EcolB_01002126       ZP_00710947.1        COG5651: PPE-repeat proteins
   92439..93038       -  199   75210816            EcolB_01002127       ZP_00710948.1        hypothetical protein
   93105..96503       -  1132  75210817            EcolB_01002128       ZP_00710949.1        COG4733: Phage-related protein, tail component
-->96564..97112       -  182   75210818            EcolB_01002129       ZP_00710950.1        COG4723: Phage-related protein, tail component
   97109..97708       -  199   75210819            EcolB_01002130       ZP_00710951.1        fragment JAB+NlpC; COG0791: Cell wall-associated hydrolases
   97858..98556       -  232   75210820            EcolB_01002131       ZP_00710952.1        COG4672: Phage-related protein
   98556..98885       -  109   75210821            EcolB_01002132       ZP_00710953.1        COG4718: Phage-related protein
---------------------------------------
  ORGANISM  Escherichia coli B171        accession no is NZ_AAJX01000046.1 gi is 75208867
   cds                dir len   gi         gene     locus                pid                  product
   17928..18257       +  109   75208864            EcolB_01003940       ZP_00709145.1        COG4718: Phage-related protein
   18267..18965       +  232   75208865            EcolB_01003941       ZP_00709146.1        COG4672: Phage-related protein
   19115..19714       +  199   75208866            EcolB_01003942       ZP_00709147.1        JAB+NlpC;  COG0791: Cell wall-associated hydrolases
-->19826..20260       +  144   75208867            EcolB_01003944       ZP_00709148.1        COG4723: Phage-related protein, tail component
   20321..23800       +  1159  75208868            EcolB_01003945       ZP_00709149.1        COG4733: Phage-related protein, tail component
   23868..24467       +  199   75208869            EcolB_01003946       ZP_00709150.1        COG3637: Opacity protein and related surface
   24468..24641       -  57    75208870            EcolB_01003947       ZP_00709151.1        hypothetical protein
---------------------------------------
  ORGANISM  Escherichia coli B171        accession no is NZ_AAJX01000051.1 gi is 75208698
   cds                dir len   gi         gene     locus                pid                  product
   <1..768            +  255   75208695            EcolB_01004066       ZP_00708989.1        COG3436: Transposase and inactivated
   684..3284          -  866   75208696            EcolB_01004067       ZP_00708990.1        COG4733: Phage-related protein, tail component
   3418..3945         +  175   75208697            EcolB_01004068       ZP_00708991.1        COG2032: Cu/Zn superoxide dismutase
-->4136..4717         -  193   75208698            EcolB_01004069       ZP_00708992.1        COG4723: Phage-related protein, tail component
   4714..5457         -  247   75208699            EcolB_01004070       ZP_00708993.1        JAB+NlpC;  COG0791: Cell wall-associated hydrolases
   5463..6161         -  232   75208700            EcolB_01004071       ZP_00708994.1        COG4672: Phage-related protein
   6161..6490         -  109   75208701            EcolB_01004072       ZP_00708995.1        COG4718: Phage-related protein
---------------------------------------
  ORGANISM  Escherichia coli B171        accession no is NZ_AAJX01000004.1 gi is 75211970
   cds                dir len   gi         gene     locus                pid                  product
   83218..83547       +  109   75211967            EcolB_01000968       ZP_00712022.1        COG4718: Phage-related protein
   83547..84245       +  232   75211968            EcolB_01000969       ZP_00712023.1        COG4672: Phage-related protein
   84358..84993       +  211   75211969            EcolB_01000970       ZP_00712024.1        JAB+NlpC;  COG0791: Cell wall-associated hydrolases
-->84990..85562       +  190   75211970            EcolB_01000971       ZP_00712025.1        COG4723: Phage-related protein, tail component
   85623..87809       +  728   75211971            EcolB_01000972       ZP_00712026.1        COG4733: Phage-related protein, tail component
   87845..89035       +  396   75211972            EcolB_01000973       ZP_00712027.1        COG4733: Phage-related protein, tail component
   89105..89704       +  199   75211973            EcolB_01000974       ZP_00712028.1        COG1629: Outer membrane receptor proteins,
---------------------------------------
  ORGANISM  Escherichia coli B7A         accession no is NZ_AAJT01000021.1 gi is 75229909
   cds                dir len   gi         gene     locus                pid                  product
   3950..4279         +  109   75229906            EcolB7_01001836      ZP_00716423.1        COG4718: Phage-related protein
   4279..4977         +  232   75229907            EcolB7_01001837      ZP_00716424.1        COG4672: Phage-related protein
   4982..5725         +  247   75229908            EcolB7_01001838      ZP_00716425.1        JAB+NlpC;  COG0791: Cell wall-associated hydrolases
-->5722..6294         +  190   75229909            EcolB7_01001839      ZP_00716426.1        COG4723: Phage-related protein, tail component
   6355..7983         +  542   75229910            EcolB7_01001840      ZP_00716427.1        COG4733: Phage-related protein, tail component
   7953..9755         +  600   75229911            EcolB7_01001841      ZP_00716428.1        COG4733: Phage-related protein, tail component
   9822..10421        +  199   75229912            EcolB7_01001842      ZP_00716429.1        hypothetical protein
---------------------------------------
  ORGANISM  Escherichia coli CFT073      accession no is AE014075.1 gi is 26107858
   cds                dir len   gi         gene     locus                pid                  product
   1438260..1438910   -  216   26107855            c1586                AAN80054.1           Hypothetical protein
   1438356..1439054   +  232   26107856            c1587                AAN80055.1           Putative tail component of prophage
   1439060..1439803   +  247   26107857            c1588                AAN80056.1           JAB+NlpC;  Putative tail component of prophage
-->1439740..1440372   +  210   26107858            c1589                AAN80057.1           Putative tail component of prophage
   1440433..1443915   +  1160  26107859            c1590                AAN80058.1           Putative tail component of prophage
   1443968..1445635   +  555   26107860            c1591                AAN80059.1           Hypothetical protein
   1444336..1444590   -  84    26107861            c1592                AAN80060.1           Hypothetical protein
---------------------------------------
  ORGANISM  Escherichia coli CFT073      accession no is AE014075.1 gi is 26107735
   cds                dir len   gi         gene     locus                pid                  product
   1362115..1362813   +  232   26107732            c1462                AAN79931.1           Putative tail component of prophage
   1362166..1362969   -  267   26107733            c1463                AAN79932.1           Hypothetical protein
   1362864..1363562   +  232   26107734            c1464                AAN79933.1           JAB+NlpC;  Putative tail fiber component K of prophage
-->1363526..1364140   +  204   26107735            c1465                AAN79934.1           Putative tail assembly protein of cryptic
   1364484..1368176   +  1230  26107736            c1466                AAN79935.1           Putative tail component of prophage
   1368244..1368843   +  199   26107737   lomP     c1467                AAN79936.1           Putative Lom-like outer membrane protein of
   1368995..1371058   +  687   26107738            c1468                AAN79937.1           Hypothetical protein
---------------------------------------
  ORGANISM  Escherichia coli CFT073      accession no is AE014075.1 gi is 26109404
   cds                dir len   gi         gene     locus                pid                  product
   3026388..3026897   -  169   26109401            c3152                AAN81604.1           Hypothetical protein
   3027043..3027642   -  199   26109402            c3153                AAN81605.1           Putative outer membrane protein of prophage
   3027710..3031189   -  1159  26109403            c3154                AAN81606.1           Putative tail component of prophage
-->3031250..3031882   -  210   26109404            c3155                AAN81607.1           Putative tail component of prophage
   3031819..3032604   -  261   26109405            c3156                AAN81608.1           JAB+NlpC;  Putative tail fiber component K of prophage
   3032561..3033367   +  268   26109406            c3157                AAN81609.1           Hypothetical protein
   3032567..3033265   -  232   26109407            c3158                AAN81610.1           Putative tail component of prophage
---------------------------------------
  ORGANISM  Escherichia coli E110019     accession no is NZ_AAJW01000001.1 gi is 75239817
   cds                dir len   gi         gene     locus                pid                  product
   60704..61051       -  115   75239814            EcolE1_01000061      ZP_00723775.1        COG3436: Transposase and inactivated
   61048..61452       -  134   75239815            EcolE1_01000062      ZP_00723776.1        COG2963: Transposase and inactivated
   61587..62186       +  199   75239816            EcolE1_01000063      ZP_00723777.1        JAB+NlpC;  COG0791: Cell wall-associated hydrolases
-->62183..62725       +  180   75239817            EcolE1_01000064      ZP_00723778.1        COG4723: Phage-related protein, tail component
   62786..66199       +  1137  75239818            EcolE1_01000065      ZP_00723779.1        COG4733: Phage-related protein, tail component
   66269..66868       +  199   75239819            EcolE1_01000066      ZP_00723780.1        hypothetical protein
   66933..69893       +  986   75239820            EcolE1_01000067      ZP_00723781.1        COG5651: PPE-repeat proteins
---------------------------------------
  ORGANISM  Escherichia coli E110019     accession no is NZ_AAJW01000023.1 gi is 75235151
   cds                dir len   gi         gene     locus                pid                  product
   19631..20230       -  199   75235148            EcolE1_01003009      ZP_00719390.1        COG1629: Outer membrane receptor proteins,
   20298..23774       -  1158  75235149            EcolE1_01003010      ZP_00719391.1        COG4733: Phage-related protein, tail component
   23908..24435       +  175   75235150            EcolE1_01003011      ZP_00719392.1        COG2032: Cu/Zn superoxide dismutase
-->24626..25207       -  193   75235151            EcolE1_01003012      ZP_00719393.1        COG4723: Phage-related protein, tail component
   25204..25947       -  247   75235152            EcolE1_01003013      ZP_00719394.1        COG0791: JAB+NlpC; Cell wall-associated hydrolases
   25953..26651       -  232   75235153            EcolE1_01003014      ZP_00719395.1        COG4672: Phage-related protein
   26651..26980       -  109   75235154            EcolE1_01003015      ZP_00719396.1        COG4718: Phage-related protein
---------------------------------------
  ORGANISM  Escherichia coli E110019     accession no is NZ_AAJW01000057.1 gi is 75214996
   cds                dir len   gi         gene     locus                pid                  product
   11961..12557       -  198   75214993            EcolE1_01004373      ZP_00713458.1        COG3637: Opacity protein and related surface
   12653..16132       -  1159  75214994            EcolE1_01004374      ZP_00713459.1        COG4733: Phage-related protein, tail component
   16266..16793       +  175   75214995            EcolE1_01004375      ZP_00713460.1        COG2032: Cu/Zn superoxide dismutase
-->16984..17565       -  193   75214996            EcolE1_01004376      ZP_00713461.1        COG4723: Phage-related protein, tail component
   17562..18305       -  247   75214997            EcolE1_01004377      ZP_00713462.1        JAB+NlpC;  COG0791: Cell wall-associated hydrolases
   18316..19014       -  232   75214998            EcolE1_01004378      ZP_00713463.1        COG4672: Phage-related protein
   19014..19355       -  113   75214999            EcolE1_01004379      ZP_00713464.1        COG4718: Phage-related protein
---------------------------------------
  ORGANISM  Escherichia coli E110019     accession no is NZ_AAJW01000052.1 gi is 75215134
   cds                dir len   gi         gene     locus                pid                  product
   116..457           +  113   75215131            EcolE1_01004234      ZP_00713579.1        COG4718: Phage-related protein
   457..1155          +  232   75215132            EcolE1_01004235      ZP_00713580.1        COG4672: Phage-related protein
   1161..1904         +  247   75215133            EcolE1_01004236      ZP_00713581.1        COG0791: Cell wall-associated hydrolases
-->1901..2482         +  193   75215134            EcolE1_01004237      ZP_00713582.1        COG4723: Phage-related protein, tail component
   2717..6190         +  1157  75215135            EcolE1_01004238      ZP_00713583.1        COG4733: Phage-related protein, tail component
   6258..6857         +  199   75215136            EcolE1_01004239      ZP_00713584.1        COG1629: Outer membrane receptor proteins,
   6922..8235         +  437   75215137            EcolE1_01004240      ZP_00713585.1        hypothetical protein
---------------------------------------
  ORGANISM  Escherichia coli E110019     accession no is NZ_AAJW01000017.1 gi is 75235846
   cds                dir len   gi         gene     locus                pid                  product
   73759..74100       +  113   75235843            EcolE1_01002594      ZP_00719998.1        COG4718: Phage-related protein
   74100..74798       +  232   75235844            EcolE1_01002595      ZP_00719999.1        COG4672: Phage-related protein
   74912..75547       +  211   75235845            JAB+NlpC; EcolE1_01002596      ZP_00720000.1        COG0791: Cell wall-associated hydrolases
-->75544..76125       +  193   75235846            EcolE1_01002597      ZP_00720001.1        COG4723: Phage-related protein, tail component
   76366..79842       +  1158  75235847            EcolE1_01002598      ZP_00720002.1        COG4733: Phage-related protein, tail component
   79938..80534       +  198   75235848            EcolE1_01002599      ZP_00720003.1        COG3637: Opacity protein and related surface
   80599..81912       +  437   75235849            EcolE1_01002600      ZP_00720004.1        hypothetical protein
---------------------------------------
  ORGANISM  Escherichia coli E110019     accession no is NZ_AAJW01000050.1 gi is 75233804
   cds                dir len   gi         gene     locus                pid                  product
   8809..9150         +  113   75233801            EcolE1_01004185      ZP_00718278.1        COG4718: Phage-related protein
   9150..9848         +  232   75233802            EcolE1_01004186      ZP_00718279.1        COG4672: Phage-related protein
   9859..10602        +  247   75233803            JAB+NlpC EcolE1_01004187      ZP_00718280.1        COG0791: Cell wall-associated hydrolases
-->10599..11180       +  193   75233804            EcolE1_01004188      ZP_00718281.1        COG4723: Phage-related protein, tail component
   11371..11976       -  201   75233805            EcolE1_01004189      ZP_00718282.1        COG2032: Cu/Zn superoxide dismutase
   12031..15507       +  1158  75233806            EcolE1_01004190      ZP_00718283.1        COG4733: Phage-related protein, tail component
   15576..16199       +  207   75233807            EcolE1_01004191      ZP_00718284.1        COG3637: Opacity protein and related surface
---------------------------------------
  ORGANISM  Salmonella typhimurium LT2   accession no is AE008818.1 gi is 16421139
   cds                dir len   gi         gene     locus                pid                  product
   5164..5988         -  274   16421136   STM2587                       AAL21482.1           Gifsy-1 prophage protein
   5985..8357         -  790   16421137   STM2588                       AAL21483.1           Gifsy-1 prophage protein
   8775..12053        -  1092  16421138   STM2589                       AAL21484.1           Gifsy-1 prophage protein
-->12115..12762       -  215   16421139   STM2590                       AAL21485.1           Gifsy-1 prophage protein
   12660..13259       -  199   16421140   STM2591                       AAL21486.1           Gifsy-1 prophage protein
   13404..14102       -  232   16421141   STM2592                       AAL21487.1           Gifsy-1 prophage protein
   14112..14441       -  109   16421142   STM2593                       AAL21488.1           Gifsy-1 prophage protein
---------------------------------------
  ORGANISM  Ralstonia solanacearum       accession no is AL646065.1 gi is 17428712
   cds                dir len   gi         gene     locus                pid                  product
   198847..199443     +  198   17428709   RSc1693                       CAD15395.1           PUTATIVE TAIL FIBER ASSEMBLY PROTEIN HOMOLOG
   199440..200141     +  233   17428710   RSc1694                       CAD15396.1           PROBABLE PHAGE HK022 GP18-RELATED PROTEIN
   200143..200853     +  236   17428711   RSc1695                       CAD15397.1           PROBABLE PHAGE HK022 GP19-RELATED PROTEIN
-->200857..201459     +  200   17428712   RSc1696                       CAD15398.1           PROBABLE PHAGE HK022 GP20-RELATED PROTEIN
   201529..201867     -  112   17428713   RSc1697                       CAD15399.1           CONSERVED HYPOTHETICAL PROTEIN
---------------------------------------
  ORGANISM  Escherichia coli E22         accession no is NZ_AAJV01000052.1 gi is 75255450
   cds                dir len   gi         gene     locus                pid                  product
   <3..347            -  115   75255447            EcolE2_01004466      ZP_00727250.1        hypothetical protein
   412..1011          -  199   75255448            EcolE2_01004467      ZP_00727251.1        COG1629: Outer membrane receptor proteins,
   1082..4495         -  1137  75255449            EcolE2_01004468      ZP_00727252.1        COG4733: Phage-related protein, tail component
-->4736..5317         -  193   75255450            EcolE2_01004469      ZP_00727253.1        COG4723: Phage-related protein, tail component
   5314..6057         -  247   75255451            EcolE2_01004470      ZP_00727254.1        COG0791: JAB+NlpC; Cell wall-associated hydrolases
   6063..6761         -  232   75255452            EcolE2_01004471      ZP_00727255.1        COG4672: Phage-related protein
   6761..7090         -  109   75255453            EcolE2_01004472      ZP_00727256.1        COG4718: Phage-related protein
---------------------------------------
  ORGANISM  Escherichia coli E22         accession no is NZ_AAJV01000060.1 gi is 75255278
   cds                dir len   gi         gene     locus                pid                  product
   <1..873            -  291   75255275            EcolE2_01004619      ZP_00727113.1        hypothetical protein
   938..1537          -  199   75255276            EcolE2_01004620      ZP_00727114.1        hypothetical protein
   1604..4996         -  1130  75255277            EcolE2_01004621      ZP_00727115.1        COG4733: Phage-related protein, tail component
-->5241..5822         -  193   75255278            EcolE2_01004622      ZP_00727116.1        COG4723: Phage-related protein, tail component
   5819..6562         -  247   75255279            EcolE2_01004623      ZP_00727117.1        JAB+NlpC;  COG0791: Cell wall-associated hydrolases
   6573..7271         -  232   75255280            EcolE2_01004624      ZP_00727118.1        COG4672: Phage-related protein
   7271..7600         -  109   75255281            EcolE2_01004625      ZP_00727119.1        COG4718: Phage-related protein
---------------------------------------
  ORGANISM  Escherichia coli E22         accession no is NZ_AAJV01000100.1 gi is 75254904
   cds                dir len   gi         gene     locus                pid                  product
   <1..1377           -  459   75254903            EcolE2_01004895      ZP_00726868.1        COG4733: Phage-related protein, tail component
-->1623..2303         -  226   75254904            EcolE2_01004896      ZP_00726869.1        COG4723: Phage-related protein, tail component
---------------------------------------
  ORGANISM  Escherichia coli E22         accession no is NZ_AAJV01000008.1 gi is 75258709
   cds                dir len   gi         gene     locus                pid                  product
   137535..140450     -  971   75258706            EcolE2_01001652      ZP_00730113.1        COG5651: PPE-repeat proteins
   140515..141114     -  199   75258707            EcolE2_01001653      ZP_00730114.1        hypothetical protein
   141181..144579     -  1132  75258708            EcolE2_01001654      ZP_00730115.1        COG4733: Phage-related protein, tail component
-->144796..145188     -  130   75258709            EcolE2_01001656      ZP_00730116.1        COG4723: Phage-related protein, tail component
   145185..145784     -  199   75258710            EcolE2_01001657      ZP_00730117.1        COG0791: Cell wall-associated hydrolases
   145934..146632     -  232   75258711            EcolE2_01001658      ZP_00730118.1        COG4672: Phage-related protein
   146632..146961     -  109   75258712            EcolE2_01001659      ZP_00730119.1        COG4718: Phage-related protein
---------------------------------------
  ORGANISM  Escherichia coli E22         accession no is NZ_AAJV01000018.1 gi is 75257430
   cds                dir len   gi         gene     locus                pid                  product
   4615..4788         +  57    75257427            EcolE2_01002682      ZP_00728930.1        hypothetical protein
   4778..5782         +  334   75257428            EcolE2_01002683      ZP_00728931.1        hypothetical protein
   5939..6574         +  211   75257429            EcolE2_01002684      ZP_00728932.1        COG0791: Cell wall-associated hydrolases
-->6571..7113         +  180   75257430            EcolE2_01002685      ZP_00728933.1        COG4723: Phage-related protein, tail component
   7591..8295         +  234   75257431            EcolE2_01002686      ZP_00728934.1        COG4733: Phage-related protein, tail component
   8285..9244         +  319   75257432            EcolE2_01002687      ZP_00728935.1        COG4733: Phage-related protein, tail component
   9222..11072        +  616   75257433            EcolE2_01002688      ZP_00728936.1        COG4733: Phage-related protein, tail component
---------------------------------------
  ORGANISM  Escherichia coli E22         accession no is NZ_AAJV01000004.1 gi is 75259495
   cds                dir len   gi         gene     locus                pid                  product
   166105..169224     +  1039  75259492            EcolE2_01000926      ZP_00730811.1        COG5281: Phage-related minor tail protein
   169549..170247     +  232   75259493            EcolE2_01000927      ZP_00730812.1        COG4672: Phage-related protein
   170360..170995     +  211   75259494            EcolE2_01000928      ZP_00730813.1        JAB+NlpC;  COG0791: Cell wall-associated hydrolases
-->170992..171540     +  182   75259495            EcolE2_01000929      ZP_00730814.1        COG4723: Phage-related protein, tail component
   171601..175014     +  1137  75259496            EcolE2_01000930      ZP_00730815.1        COG4733: Phage-related protein, tail component
   175084..175683     +  199   75259497            EcolE2_01000931      ZP_00730816.1        COG1629: Outer membrane receptor proteins,
   175748..177061     +  437   75259498            EcolE2_01000932      ZP_00730817.1        hypothetical protein
---------------------------------------
  ORGANISM  Bacteriophage CP-1639        accession no is AJ304858.2 gi is 51773733
   cds                dir len   gi         gene     locus                pid                  product
   37563..37892       +  109   51773730            EC_CP1639_57         CAH23255.1           putative tail fiber component M
   37892..38590       +  232   51773731            EC_CP1639_58         CAH23256.1           putative tail fiber component L
   38596..39339       +  247   51773732            EC_CP1639_59         CAH23257.1           JAB+NlpC; putative tail fiber component K
-->39371..39913       +  180   51773733            EC_CP1639_60         CAH23258.1           putative tail fiber component I
   40154..43630       +  1158  51773734            EC_CP1639_61         CAH23259.1           putative tail fiber component J
   43697..44296       +  199   51773735   lom      EC_CP1639_62         CAH23260.1           putative outer membrane protein Lom precursor
   44361..45683       +  440   51773736            EC_CP1639_63         CAH23261.1           putative tail fiber protein
---------------------------------------
  ORGANISM  Escherichia coli F11         accession no is NZ_AAJU01000023.1 gi is 75239568
   cds                dir len   gi         gene     locus                pid                  product
   46943..47200       -  85    75239565            EcolF_01003072       ZP_00723535.1        hypothetical protein
   47524..49584       -  686   75239566            EcolF_01003073       ZP_00723536.1        hypothetical protein
   49643..53125       -  1160  75239567            EcolF_01003074       ZP_00723537.1        COG4733: Phage-related protein, tail component
-->53186..53734       -  182   75239568            EcolF_01003075       ZP_00723538.1        COG4723: Phage-related protein, tail component
   53731..54330       -  199   75239569            EcolF_01003076       ZP_00723539.1        JAB+NlpC;  COG0791: Cell wall-associated hydrolases
   54480..55178       -  232   75239570            EcolF_01003077       ZP_00723540.1        COG4672: Phage-related protein
   55188..55517       -  109   75239571            EcolF_01003078       ZP_00723541.1        COG4718: Phage-related protein
---------------------------------------
  ORGANISM  Escherichia coli F11         accession no is NZ_AAJU01000022.1 gi is 75239670
   cds                dir len   gi         gene     locus                pid                  product
   72506..72835       +  109   75239667            EcolF_01003023       ZP_00723634.1        COG4718: Phage-related protein
   72835..73533       +  232   75239668            EcolF_01003024       ZP_00723635.1        COG4672: Phage-related protein
   73539..74282       +  247   75239669            EcolF_01003025       ZP_00723636.1        JAB+NlpC;  COG0791: Cell wall-associated hydrolases
-->74279..74851       +  190   75239670            EcolF_01003026       ZP_00723637.1        COG4723: Phage-related protein, tail component
   74912..>75601      +  230   75239671            EcolF_01003027       ZP_00723638.1        COG4733: Phage-related protein, tail component
---------------------------------------
  ORGANISM  Nitrobacter sp. Nb-311A      accession no is NZ_AAMY01000021.1 gi is 85716602
   cds                dir len   gi         gene     locus                pid                  product
   41371..41460       -  29    85716599            NB311A_12122         ZP_01047569.1        hypothetical protein
   41472..41645       -  57    85716600            NB311A_12127         ZP_01047570.1        hypothetical protein
   41671..42087       +  138   85716601            NB311A_12132         ZP_01047571.1        NlpC, tail assembly protein, putative
-->42088..45891       +  1267  85716602            NB311A_12137         ZP_01047572.1        tail fiber protein, putative
   45903..47351       +  482   85716603            ? NB311A_12142         ZP_01047573.1        hypothetical protein
   47373..49427       +  684   85716604            NB311A_12147         ZP_01047574.1        putative membrane-anchored cell surface protein
   49424..49648       -  74    85716605            NB311A_12152         ZP_01047575.1        hypothetical protein
---------------------------------------
  ORGANISM  Escherichia coli O157:H7     accession no is BA000007.2 gi is 13361452
   cds                dir len   gi         gene     locus                pid                  product
   1960975..1961316   +  113   13361449   ECs1984                       BAB35407.1           putative minor tail protein
   1961316..1962014   +  232   13361450   ECs1985                       BAB35408.1           putative minor tail protein
   1962025..1962768   +  247   13361451   ECs1986                       BAB35409.1           JAB+NlpC;  putative tail assembly protein
-->1962666..1963346   +  226   13361452   ECs1987                       BAB35410.1           putative tail assembly protein
   1963300..1963506   +  68    13361453   ECs1988                       BAB35411.1           hypothetical protein
   1963537..1964064   -  175   13361454   ECs1989                       BAB35412.1           putative copper/zinc-superoxide dismutase
   1964198..1967695   +  1165  13361455   ECs1990                       BAB35413.1           putative host specificity protein
---------------------------------------
  ORGANISM  Bacteriophage phi1026b       accession no is NC_005284.1 gi is 38707909
   cds                dir len   gi         gene     locus                pid                  product
   12954..14342       +  462   38707906            phi1026bp16          NP_945046.1          gp16
   14339..15022       +  227   38707907            phi1026bp17          NP_945047.1          gp17
   15072..15824       +  250   38707908            phi1026bp18          NP_945048.1          JAB+NlpC;  gp18
-->15821..16405       +  194   38707909            phi1026bp19          NP_945049.1          gp19
   16402..19707       +  1101  38707910            phi1026bp20          NP_945050.1          gp20
   19704..20018       +  104   38707911            phi1026bp21          NP_945051.1          gp21
   20018..20752       +  244   38707912            phi1026bp22          NP_945052.1          gp22
---------------------------------------
  ORGANISM  Escherichia coli O157:H7     accession no is BA000007.2 gi is 13360578
   cds                dir len   gi         gene     locus                pid                  product
   1200126..1200455   +  109   13360575   ECs1115                       BAB34538.1           putative minor tail protein
   1200455..1201153   +  232   13360576   ECs1116                       BAB34539.1           putative minor tail protein
   1201272..1201907   +  211   13360577   ECs1117                       BAB34540.1           putative tail assembly protein
-->1201805..1202485   +  226   13360578   ECs1118                       BAB34541.1           putative tail assembly protein
   1202439..1202645   +  68    13360579   ECs1119                       BAB34542.1           hypothetical protein
   1202676..1203203   -  175   13360580   ECs1120                       BAB34543.1           putative copper/zinc-superoxide dismutase
   1203337..1206810   +  1157  13360581   ECs1121                       BAB34544.1           putative host specificity protein
---------------------------------------
  ORGANISM  Escherichia coli O157:H7     accession no is BA000007.2 gi is 13361702
   cds                dir len   gi         gene     locus                pid                  product
   2213781..2213996   -  71    13361699   ECs2233                       BAB35656.1           putative host specificity protein
   2213999..2215960   -  653   13361700   ECs2234                       BAB35657.1           putative host specificity protein
   2216080..2217255   -  391   13361701   ECs2235                       BAB35658.1           putative host specificity protein
-->2217597..2218277   -  226   13361702   ECs2236                       BAB35659.1           putative tail assembly protein
   2218175..2218849   -  224   13361703   ECs2237                       BAB35660.1           JAB+NlpC;  putative tail assembly protein
   2218929..2219627   -  232   13361704   ECs2238                       BAB35661.1           minor tail protein
   2219627..2219968   -  113   13361705   ECs2239                       BAB35662.1           putative minor tail protein
---------------------------------------
  ORGANISM  Escherichia coli O157:H7     accession no is BA000007.2 gi is 13361023
   cds                dir len   gi         gene     locus                pid                  product
   1576413..1576667   -  84    13361020   ECs1556                       BAB34979.1           putative regulatory protein
   1577190..1578068   +  292   13361021   ECs1557                       BAB34980.1           putative antirepressor protein
   1578122..1578859   +  245   13361022   ECs1558                       BAB34981.1           JAB+NlpC; putative tail assembly protein
-->1578757..1579041   +  94    13361023   ECs1559                       BAB34982.1           putative tail assembly protein
   1579163..1581511   +  782   13361024   ECs1560                       BAB34983.1           putative secreted effector protein
   1582102..1585503   +  1133  13361025   ECs1561                       BAB34984.1           hypothetical protein
   1585490..1585645   +  51    13361026   ECs1562                       BAB34985.1           hypothetical protein
---------------------------------------
  ORGANISM  Escherichia coli O157:H7     accession no is BA000007.2 gi is 13361111
   cds                dir len   gi         gene     locus                pid                  product
   1642980..1643309   +  109   13361108   ECs1644                       BAB35067.1           minor tail protein
   1643309..1644007   +  232   13361109   ECs1645                       BAB35068.1           minor tail protein
   1644013..1644756   +  247   13361110   ECs1646                       BAB35069.1           tail assembly protein
-->1644654..1645325   +  223   13361111   ECs1647                       BAB35070.1           tail assembly protein
   1645386..1648784   +  1132  13361112   ECs1648                       BAB35071.1           host specificity protein
   1648851..1649450   +  199   13361113   ECs1649                       BAB35072.1           putative membrane protein precursor
   1649515..1652430   +  971   13361114   ECs1650                       BAB35073.1           putative tail fiber protein
---------------------------------------
  ORGANISM  Escherichia coli O157:H7     accession no is BA000007.2 gi is 13362414
   cds                dir len   gi         gene     locus                pid                  product
   2898416..2899015   -  199   13362411   ECs2942                       BAB36365.1           putative outer membrane protein Lom precursor
   2899083..2901410   -  775   13362412   ECs2943                       BAB36366.1           putative host specificity protein
   2901380..2902555   -  391   13362413   ECs2944                       BAB36367.1           putative host specificity protein
-->2902796..2903473   -  225   13362414   ECs2945                       BAB36368.1           putative tail assembly protein
   2903371..2904114   -  247   13362415   JAB+NlpC; ECs2946                       BAB36369.1           putative tail assembly protein
   2904125..2904823   -  232   13362416   ECs2947                       BAB36370.1           putative minor tail protein
   2904823..2905152   -  109   13362417   ECs2948                       BAB36371.1           putative minor tail protein
---------------------------------------
  ORGANISM  Escherichia coli O157:H7     accession no is BA000007.2 gi is 13360300
   cds                dir len   gi         gene     locus                pid                  product
   916322..916651     +  109   13360297   ECs0838                       BAB34261.1           putative minor tail protein
   916661..917359     +  232   13360298   ECs0839                       BAB34262.1           putative minor tail protein
   917509..918108     +  199   13360299   ECs0840                       BAB34263.1           putative tail assembly protein
-->918006..918653     +  215   13360300   ECs0841                       BAB34264.1           putative tail assembly protein
   918714..922127     +  1137  13360301   ECs0842                       BAB34265.1           putative host specificity protein
   922198..922797     +  199   13360302   ECs0843                       BAB34266.1           putative outer membrane protein precursor
   922857..924173     +  438   13360303   ECs0844                       BAB34267.1           putative tail fiber protein
---------------------------------------
  ORGANISM  Escherichia coli O157:H7 EDL933      accession no is AE005174.2 gi is 12516097
   cds                dir len   gi         gene     locus                pid                  product
   2746319..2747638   -  439   12516091            Z3074                AAG56993.1           putative tail fiber protein of prophage CP-933U
   2747697..2748296   -  199   12516092   lomU     Z3075                AAG56994.1           putative outer membrane protein of prophage
   2748365..2751844   -  1159  12516093            Z3077                AAG56995.1           putative tail fiber component J of prophage
-->2752085..2752621   -  178   12516097            Z3079                AAG56996.1           putative tail fiber component I of prophage
   2752660..2753541   -  293   12516098            Z3081                AAG56997.1           JAB+NlpC; JAB; putative tail fiber component K of prophage
   2753414..2754112   -  232   12516099            Z3082                AAG56998.1           putative tail fiber component L of prophage
   2754112..2754441   -  109   12516100            Z3083                AAG56999.1           putative tail fiber component M of prophage
---------------------------------------
  ORGANISM  Magnetospirillum gryphiswaldense     accession no is AM085146.1 gi is 78033450
   cds                dir len   gi         gene     locus                pid                  product
   18910..19230       +  938   12512709   ileS     mgI414               AAG54328.1           isoleucine tRNA synthetase
   19441..20040       -  199   78033448            mgI414               CAJ30064.1           hypothetical protein
   20113..20397       -  94    78033449            mgI415               CAJ30065.1           hypothetical protein
-->20544..22844       -  766   78033450            mgI416               CAJ30066.1           phage-related protein
   23320..23568       +  82    78033451            mgIa14               CAJ30067.1           conserved hypothetical protein
   23565..23978       +  137   78033452            mgI417               CAJ30068.1           putative plasmid stability-like protein
   23985..24587       -  200   78033453            mgI418               CAJ30069.1           ?H conserved hypothetical protein
---------------------------------------
  ORGANISM  Magnetospirillum magneticum AMB-1    accession no is AP007255.1 gi is 82944335
   cds                dir len   gi         gene     locus                pid                  product
   419880..420230     +  116   82944332            amb0392              BAE49196.1           hypothetical protein
   420223..420747     +  174   82944333            amb0393              BAE49197.1           ?H, hypothetical protein
   420791..421243     +  150   82944334            amb0394              BAE49198.1           NlpC solo hypothetical protein
-->421240..423567     +  775   82944335            amb0395              BAE49199.1           Phage-related protein
   423624..424067     +  147   82944336            amb0396              BAE49200.1           P5 hypothetical protein
   424070..424666     +  198   82944337            amb0397              BAE49201.1           lysozyme, hypothetical protein
   429860..430327     +  155   82944338            amb0398              BAE49202.1           Insertion element IS402 hypothetical 162 kDa
---------------------------------------
  ORGANISM  Magnetospirillum magneticum AMB-1    accession no is AP007255.1 gi is 82945132
   cds                dir len   gi         gene     locus                pid                  product
   1264072..1264422   +  116   82945129            amb1189              BAE49993.1           hypothetical protein
   1264415..1264939   +  174   82945130            amb1190              BAE49994.1           ?H, hypothetical protein
   1265007..1265402   +  131   82945131            amb1191              BAE49995.1           NlpC; hypothetical protein
-->1265399..1267726   +  775   82945132            amb1192              BAE49996.1           Phage-related protein
   1267825..1268262   +  145   82945133            amb1193              BAE49997.1           P5, hypothetical protein
   1268265..1268864   +  199   82945134            amb1194              BAE49998.1           lysozyme, hypothetical protein
   1269056..1269955   -  299   82945135            amb1195              BAE49999.1           Predicted transcriptional regulator
---------------------------------------
  ORGANISM  Bordetella bronchiseptica RB50       accession no is BX640447.1 gi is 33576899
   cds                dir len   gi         gene     locus                pid                  product
   245307..246119     -  270   33576896            BB3483               CAE33976.1           phage-related hypothetical protein
   246119..246382     -  87    33576897            BB3484               CAE33977.1           phage-related hypothetical protein
   246601..247017     -  138   33576898            BB3485               CAE33978.1           phage-related hypothetical protein
-->247022..250978     -  1318  33576899            BB3486               CAE33979.1           phage-related conserved hypothetical protein
   250971..251360     -  129   33576900            BB3487               CAE33980.1           NlpC, phage-related conserved hypothetical protein
   251357..251950     -  197   33576901            BB3488               CAE33981.1           phage-related conserved hypothetical protein
   251957..252316     -  119   33576902            BB3489               CAE33982.1           phage-related conserved hypothetical protein
---------------------------------------
  ORGANISM  Xanthomonas oryzae phage OP1         accession no is AP008979.1 gi is 84570663
   cds                dir len   gi         gene     locus                pid                  product
   13552..13905       +  117   84570660                                 BAE72723.1           conserved hypothetical protein
   13905..14363       +  152   84570661                                 BAE72724.1           ?H conserved hypothetical protein
   14318..14758       +  146   84570662                                 BAE72725.1           NlpC, conserved hypothetical protein
-->14743..19458       +  1571  84570663                                 BAE72726.1           putative tail component protein
   19492..19794       +  100   84570664                                 BAE72727.1           hypothetical protein
   19794..20489       +  231   84570665                                 BAE72728.1           conserved hypothetical protein
   20486..20857       -  123   84570666                                 BAE72729.1           hypothetical protein
---------------------------------------
  ORGANISM  Xanthomonas campestris phage Xp10    accession no is AY299121.1 gi is 31788497
   cds                dir len   gi         gene     locus                pid                  product
   13362..13718       +  118   31788494                                 AAP58686.1           19R
   13715..14173       +  152   31788495                                 AAP58687.1           ?H. 20R
   14128..14568       +  146   31788496                                 AAP58688.1           21R
-->14553..19277       +  1574  31788497                                 AAP58689.1           22R
   19322..19612       +  96    31788498                                 AAP58690.1           23R
   19612..20304       +  230   31788499                                 AAP58691.1           24R
   20301..20669       -  122   31788500                                 AAP58692.1           25L
---------------------------------------
  ORGANISM  Methylobacillus flagellatus KT       accession no is NZ_AADX02000002.1 gi is 68212786
   cds                dir len   gi         gene     locus                pid                  product
   266957..267496     -  179   68212783            MflaDRAFT_2313       ZP_00564619.1        Sigma-70 region 2:Sigma-70 region 4
   267708..268499     -  263   68212784            MflaDRAFT_2311       ZP_00564620.1        N6 adenine-specific DNA methyltransferase, D12
   268648..269331     -  227   68212785            MflaDRAFT_2310       ZP_00564621.1        hypothetical protein
-->270264..273779     -  1171  68212786            MflaDRAFT_2309       ZP_00564622.1        similar to Phage-related protein tail component
   273776..274177     -  133   68212787            MflaDRAFT_2308       ZP_00564623.1        NlpC solo, hypothetical protein
   274174..274716     -  180   68212788            MflaDRAFT_2307       ZP_00564624.1        ?H. hypothetical protein
   274726..275127     -  133   68212789            MflaDRAFT_2306       ZP_00564625.1        hypothetical protein
---------------------------------------
  ORGANISM  Neisseria meningitidis phage 2120    accession no is AJ278707.1 gi is 11877308
   cds                dir len   gi         gene     locus                pid                  product
   <2..586            +  194   11877306                                 CAC19020.1           putative protein L
   583..1338          +  251   11877307                                 JAB+NlpC;  CAC19021.1
-->1335..2057         +  240   11877308                                 CAC19022.1           putative protein I
   2193..6458         +  1421  11877309                                 CAC19023.1
---------------------------------------
  ORGANISM  Escherichia coli O157:H7 EDL933      accession no is AE005174.2 gi is 12515098
   cds                dir len   gi         gene     locus                pid                  product
   1919249..1919578   +  109   12515095            Z2141                AAG56207.1           putative tail component of prophage CP-933O
   1919578..1920276   +  232   12515096            Z2142                AAG56208.1           putative tail component of prophage CP-933O
   1920149..1921030   +  293   12515097            Z2143                AAG56209.1           JAB+NlpC; putative tail component of prophage CP-933O
-->1920928..1921605   +  225   12515098            Z2144                AAG56210.1           putative tail component of prophage CP-933O
   1921846..1925322   +  1158  12515102            Z2145                AAG56211.1           putative tail component of prophage CP-933O
   1925390..1925989   +  199   12515103            Z2146                AAG56212.1           putative outer membrane protein Lom precursor of
   1926048..1927367   +  439   12515104            Z2147                AAG56213.1           putative tail fiber protein of prophage CP-933O
---------------------------------------
  ORGANISM  Syntrophobacter fumaroxidans MPOB    accession no is NZ_AAJF01000105.1 gi is 71548099
   cds                dir len   gi         gene     locus                pid                  product
   922..1506          +  194   71548096            SfumDRAFT_3597       ZP_00668555.1        hypothetical protein
   1752..1979         -  75    71548097            SfumDRAFT_3598       ZP_00668556.1        hypothetical protein
   1993..2271         -  92    71548098            SfumDRAFT_3599       ZP_00668557.1        hypothetical protein
-->2281..7215         -  1644  71548099            SfumDRAFT_3600       ZP_00668558.1        similar to Phage-related protein tail component
---------------------------------------
  ORGANISM  Photobacterium profundum SS9         accession no is CR378679.1 gi is 46916380
   cds                dir len   gi         gene     locus                pid                  product
   25818..27059       +  413   46916377   SO3013   PBPRB1271            CAG23142.1           hypothetical phage integrase family
   27229..28278       -  349   46916378            PBPRB1272            CAG23143.1           hypothetical protein
   28275..28607       -  110   46916379            PBPRB1273            CAG23144.1           hypothetical protein
-->28709..32593       -  1294  46916380            PBPRB1274            CAG23145.1           hypothetical protein
   32973..33449       -  158   46916381   BB1708   PBPRB1275            CAG23146.1           novel protease, hypothetical protein
   33450..33809       -  119   46916382   BB1707   PBPRB1276            CAG23147.1           hypothetical protein
   33806..36211       -  801   46916383            PBPRB1277            CAG23148.1           gpH, hypothetical protein
---------------------------------------
  ORGANISM  Bordetella bronchiseptica RB50       accession no is BX640442.1 gi is 33568295
   cds                dir len   gi         gene     locus                pid                  product
   57143..57490       +  115   33568292            BB1707               CAE32204.1           phage-related hypothetical protein
   57492..57968       +  158   33568293            BB1708               CAE32205.1           phage-related hypothetical protein
   57968..58354       +  128   33568294            BB1709               CAE32206.1           NlpC, phage-related hypothetical protein
-->58381..62187       +  1268  33568295            BB1710               CAE32207.1           phage-related hypothetical protein
   62192..62968       +  258   33568296            BB1711               CAE32208.1           phage-related putative exported protein
   62965..63354       +  129   33568297            BB1712               CAE32209.1           phage-related hypothetical protein
   63414..63884       +  156   33568298            BB1713               CAE32210.1           phage-related putative membrane protein
---------------------------------------
  ORGANISM  Pseudomonas aeruginosa C3719         accession no is NZ_AAKV01000073.1 gi is 84318835
   cds                dir len   gi         gene     locus                pid                  product
   17977..18582       -  201   84318832            PaerC_01003342       ZP_00967249.1        COG0512: Anthranilate/para-aminobenzoate
   22079..23359       -  426   84318833            PaerC_01003344       ZP_00967250.1        COG4733: Phage-related protein, tail component
   23623..25689       -  688   84318834            PaerC_01003345       ZP_00967251.1        COG4733: Phage-related protein, tail component
-->25809..26411       -  200   84318835            PaerC_01003346       ZP_00967252.1        COG4723: Phage-related protein, tail component
   26466..27236       -  256   84318836            PaerC_01003347       ZP_00967253.1        JAB+NlpC;  COG0791: Cell wall-associated hydrolases
   27239..27934       -  231   84318837            PaerC_01003348       ZP_00967254.1        COG4672: Phage-related protein
   27942..28283       -  113   84318838            PaerC_01003349       ZP_00967255.1        COG4718: Phage-related protein
---------------------------------------
  ORGANISM  Bordetella pertussis Tohama I        accession no is BX640421.1 gi is 33564325
   cds                dir len   gi         gene     locus                pid                  product
   97098..97910       -  270   33564322            BP3358               CAE43623.1           phage-related hypothetical protein
   97910..98170       -  86    33564323            BP3359               CAE43624.1           phage-related hypothetical protein
   98392..98808       -  138   33564324            BP3361               CAE43625.1           phage-related hypothetical protein
-->98813..102769      -  1318  33564325            BP3362               CAE43626.1           phage-related conserved hypothetical protein
   102762..103151     -  129   33564326            BP3363               CAE43627.1           NlpC solo, phage-related conserved hypothetical protein
   103148..103681     -  177   33564327            BP3364               CAE43628.1           phage-related conserved hypothetical protein
   103749..104108     -  119   33564328            BP3365               CAE43629.1           phage-related conserved hypothetical protein
---------------------------------------
  ORGANISM  Shigella flexneri 2a str. 301        accession no is AE005674.1 gi is 56383531
   cds                dir len   gi         gene     locus                pid                  product
   1920778..1921839   -  353   56383530            SF1882               AAN43437.2           putative tail component encoded by cryptic
   1921903..1922502   -  199   24052220            SF1883               AAN43438.1           putative membrane protein precursor
   1922570..1926049   -  1159  24052221            SF1884               AAN43439.1           host specificity protein
-->1926110..1926652   -  180   56383531            SF1885               AAN43440.2           putative tail component
   1926649..1927248   -  199   56383532            SF1886               AAN43441.2           JAB+NlpC;  putative tail assembly protein
   1927397..1928095   -  232   24052224            SF1887               AAN43442.1           minor tail protein
   1928095..1928424   -  109   24052225            SF1888               AAN43443.1           putative minor tail protein
---------------------------------------
  ORGANISM  Pseudomonas fluorescens Pf-5         accession no is CP000076.1 gi is 68345404
   cds                dir len   gi         gene     locus                pid                  product
   4341320..4341841   -  173   68345401            PFL_3742             AAY93007.1           phage protein, putative
   4341838..4342413   -  191   68345402            PFL_3743             AAY93008.1           structural protein P5, putative
   4343541..4346630   -  1029  68345403            PFL_3744             AAY93009.1           host specificity protein J, truncation
-->4346688..4347254   -  188   68345404            PFL_3745             AAY93010.1           prophage LambdaSo, tail assembly protein I
   4347840..4348646   -  268   68345405            PFL_3746             AAY93011.1           Sb46
   4348643..4349527   -  294   68345406            PFL_3747             AAY93012.1           probable phage protein YPO2126
   4350411..4351133   +  240   68345407            PFL_3748             AAY93013.1           hypothetical protein
---------------------------------------
  ORGANISM  Shigella flexneri 2a str. 301        accession no is AE005674.1 gi is 24050968
   cds                dir len   gi         gene     locus                pid                  product
   739164..739493     +  109   24050965            SF0714               AAN42349.1           putative minor tail protein
   739493..740191     +  232   24050966            SF0715               AAN42350.1           minor tail protein
   740196..740939     +  247   24050967            SF0716               AAN42351.1           putative tail assembly protein
-->740903..741478     +  191   24050968            SF0717               AAN42352.1           putative tail component
   741539..745018     +  1159  24050971            SF0718               AAN42353.1           host specificity protein
   745086..745685     +  199   24050972            SF0719               AAN42354.1           putative membrane protein precursor
   745707..746810     +  367   24050973            SF0720               AAN42355.1           putative tail component encoded by cryptic
---------------------------------------
  ORGANISM  Burkholderia cepacia phage Bcep176   accession no is NC_007497.1 gi is 77864688
   cds                dir len   gi         gene     locus                pid                  product
   24332..25048       -  238   77864685   60       BCPBV176_60          YP_355395.1          gp60
   25048..25356       -  102   77864686   61       BCPBV176_61          YP_355396.1          gp61
   25356..28658       -  1100  77864687   62       BCPBV176_62          YP_355397.1          gp62
-->28655..29218       -  187   77864688   63       BCPBV176_63          YP_355398.1          gp63
   29215..29967       -  250   77864689   64       BCPBV176_64          YP_355399.1          gp64
   30016..30699       -  227   77864690   65       BCPBV176_65          YP_355400.1          gp65
   30742..31749       -  335   77864691   66       BCPBV176_66          YP_355401.1          gp66
---------------------------------------
  ORGANISM  Psychrobacter arcticus 273-4         accession no is CP000082.1 gi is 71037999
   cds                dir len   gi         gene     locus                pid                  product
   551327..551650     -  107   71037996            Psyc_0441            AAZ18304.1           hypothetical protein
   551715..552266     -  183   71037997            Psyc_0442            AAZ18305.1           hypothetical protein
   552271..555444     -  1057  71037998            Psyc_0443            AAZ18306.1           putative prophage LambdaSo, host specificity
-->555447..556010     -  187   71037999            Psyc_0444            AAZ18307.1           probable phage protein tail protein
   556066..556815     -  249   71038000            Psyc_0445            AAZ18308.1           probable prophage LambdaSo, tail assembly
   556815..557633     -  272   71038001            Psyc_0446            AAZ18309.1           probable phage minor tail protein
   557921..559009     -  362   71038002            Psyc_0447            AAZ18310.1           possible RNA-directed DNA polymerase (Reverse
---------------------------------------
  ORGANISM  Burkholderia pseudomallei 1710b      accession no is CP000124.1 gi is 76579036
   cds                dir len   gi         gene     locus                pid                  product
   1809946..1811334   +  462   76580169            BURPS1710b_1690      ABA49644.1           gp16
   1811331..1812014   +  227   76581190            BURPS1710b_1691      ABA50665.1           phage minor tail protein L
   1812064..1812816   +  250   76578089            BURPS1710b_1692      ABA47564.1           gp18
-->1812831..1813397   +  188   76579036            BURPS1710b_1693      ABA48511.1           Bacteriophage lambda tail assembly protein I
   1813421..1816699   +  1092  76580608            BURPS1710b_1694      ABA50083.1           gp20
   1817992..1818483   +  163   76581312            BURPS1710b_1695      ABA50787.1           gp24
   1818483..1819028   +  181   76578821            BURPS1710b_1696      ABA48296.1           Bacteriophage lysis protein
---------------------------------------
  ORGANISM  Burkholderia thailandensis E264      accession no is NC_007650.1 gi is 83717443
   cds                dir len   gi         gene     locus                pid                  product
   1230418..1231824   +  468   83717968            BTH_II1058           YP_439255.1          gp16
   1231821..1232504   +  227   83716138            BTH_II1059           YP_439256.1          phage minor tail protein L
   1232554..1233306   +  250   83716657            BTH_II1060           YP_439257.1          JAB+NlpC;  gp19
-->1233303..1233887   +  194   83717443            BTH_II1061           YP_439258.1          Bacteriophage lambda tail assembly protein I
   1233884..1237189   +  1101  83716294            BTH_II1062           YP_439259.1          host specificity protein J
   1237183..1237497   +  104   83718055            BTH_II1063           YP_439260.1          gp21
   1237473..1238213   +  246   83717361            BTH_II1064           YP_439261.1          gp22
---------------------------------------
  ORGANISM  Escherichia coli O157:H7 EDL933      accession no is AE005174.2 gi is 12514222
   cds                dir len   gi         gene     locus                pid                  product
   1285628..1286059   +  143   12514219            Z1375                AAG55508.1           gpL, putative tail component encoded by cryptic
   1286065..1286235   +  56    12514220            Z1376                AAG55509.1           gpL, putative tail component encoded by cryptic
   1286409..1286927   +  172   12514221            Z1377                AAG55510.1           JAB+NlpC, putative tail component encoded by cryptic
-->1286761..1287663   +  300   12514222            Z1378                AAG55511.1           JAB+ThiS, putative tail component encoded by cryptic
   1288074..1289009   +  311   12514223            Z1379                AAG55512.1           J (N)putative tail component encoded by cryptic
   1289061..1291484   +  807   12514224            Z1380                AAG55513.1           J (C)putative tail component encoded by cryptic
   1291552..1292151   +  199   12514225            Z1381                AAG55514.1           gpM, putative outer membrane protein Lom precursor
---------------------------------------
  ORGANISM  Burkholderia vietnamiensis G4        accession no is NZ_AAEH02000015.1 gi is 67545284
   cds                dir len   gi         gene     locus                pid                  product
   59549..61195       +  548   67545281            Bcep1808DRAFT_4079   ZP_00423204.1        Regulator of chromosome condensation, RCC1
   61209..61751       +  180   67545282            Bcep1808DRAFT_4080   ZP_00423205.1        phage-related conserved hypothetical protein
   61748..62164       +  138   67545283            Bcep1808DRAFT_4081   ZP_00423206.1        NlpC, phage-related conserved hypothetical protein
-->62161..64464       +  767   67545284            Bcep1808DRAFT_4082   ZP_00423207.1        phage-related conserved hypothetical protein
   64476..64766       +  96    67545285            Bcep1808DRAFT_4083   ZP_00423208.1        probable transmembrane protein
   64763..65191       +  142   67545286            Bcep1808DRAFT_4084   ZP_00423209.1        hypothetical protein
   65500..66558       +  352   67545287            Bcep1808DRAFT_4085   ZP_00423210.1        Glycosyl transferase, family 2
---------------------------------------
  ORGANISM  Pseudomonas syringae pv. phaseolicola 1448A  accession no is CP000058.1 gi is 71558268
   cds                dir len   gi         gene     locus                pid                  product
   2503232..2503648   +  138   71558720            PSPPH_2150           AAZ37931.1           conserved hypothetical protein
   2505417..2506277   -  286   71557442            PSPPH_2151           AAZ36653.1           prophage PSPPH03, transcriptional regulator,
   2506776..2507567   +  263   71556629            PSPPH_2152           AAZ35840.1           conserved domain protein
-->2508072..2508530   +  152   71558268            PSPPH_2153           AAZ37479.1           prophage PSPPH03, putative tail assembly protein
   2508586..2510475   +  629   71556432            PSPPH_2154           AAZ35643.1           prophage PSPPH03, host specificity protein J,
   2510577..2510675   +  32    71556676            PSPPH_2155           AAZ35887.1           hypothetical protein
   2511167..2512426   +  419   71558465            PSPPH_2156           AAZ37676.1           ISPsy18, transposase
---------------------------------------
  ORGANISM  Magnetospirillum magnetotacticum MS-1        accession no is NZ_AAAP01003868.1 gi is 23015894
   cds                dir len   gi         gene     locus                pid                  product
   15991..16347       -  118   46201137            Magn03010332         ZP_00207980.1        hypothetical protein
   16456..17055       -  199   23015893            Magn03010333         ZP_00055657.1        , hypothetical protein
   17058..17687       -  209   46201138            Magn03010334         ZP_00207981.1        lysozyme, COG3179: Predicted chitinase
-->17700..20000       -  766   23015894            Magn03010335         ZP_00055658.1        COG4733: Phage-related protein, tail component
   20410..20907       -  165   46201139            Magn03010336         ZP_00055659.2        ?H hypothetical protein
   20927..21280       -  117   23015896            Magn03010337         ZP_00055660.1        hypothetical protein
   21287..21556       -  89    46201140            Magn03010338         ZP_00207982.1        hypothetical protein
---------------------------------------
  ORGANISM  Magnetospirillum magnetotacticum MS-1        accession no is NZ_AAAP01003876.1 gi is 23016384
   cds                dir len   gi         gene     locus                pid                  product
   <3..554            -  184   23016383            Magn03010830         ZP_00056139.1        N-terminus of protein J COG4733: Phage-related protein, tail component
-->542..2068          -  508   23016384            Magn03010831         ZP_00056140.1        COG0001: Glutamate-1-semialdehyde
   2065..2460         -  131   23016385            Magn03010832         ZP_00056141.1        COG0791: Cell wall-associated hydrolases
   2528..3037         -  169   46200892            Magn03010833         ZP_00056142.2        hypothetical protein
   3045..3398         -  117   23016387            Magn03010834         ZP_00056143.1        hypothetical protein
---------------------------------------
  ORGANISM  Sodalis glossinidius str. 'morsitans'        accession no is AP008232.1 gi is 84780140
   cds                dir len   gi         gene     locus                pid                  product
   2801623..2802141   +  172   84780137            SG1639               BAE74914.1           hypothetical phage protein
   2803187..2803870   +  227   84780138            SG1640               BAE74915.1           putative phage outer membrane protein
   2804030..2804731   -  233   84780139            SG1641               BAE74916.1           hypothetical protein
-->2806595..2807191   -  198   84780140            SG1642               BAE74917.1           HNH, putative phage tail assembly protein
   2807831..2808169   -  112   84780141            SG1643               BAE74918.1           hypothetical phage protein
   2808414..2808863   -  149   84780142            SG1644               BAE74919.1           phage lysozyme lysis protein
   2809625..2810335   -  236   84780143            SG1645               BAE74920.1           gp40 putative phage antiterminator Q protein
---------------------------------------
  ORGANISM  Magnetospirillum magnetotacticum MS-1        accession no is NZ_AAAP01003780.1 gi is 23013869
   cds                dir len   gi         gene     locus                pid                  product
   6947..7969         -  340   46202129            Magn03008321         ZP_00053718.2        COG0697: Permeases of the drug/metabolite
   8118..8717         -  199   23013867            Magn03008322         ZP_00053719.1        lysozyme, hypothetical protein
   8714..9157         -  147   23013868            Magn03008323         ZP_00053720.1        P5, hypothetical protein
-->9221..11548        -  775   23013869            Magn03008324         ZP_00053721.1        COG4733: Phage-related protein, tail component
   11545..11853       -  102   46202130            Magn03008325         ZP_00053722.2        NlpC solohypothetical protein
---------------------------------------
  ORGANISM  Pseudomonas syringae pv. syringae B728a      accession no is NC_007005.1 gi is 66046010
   cds                dir len   gi         gene     locus                pid                  product
   3355904..3356590   -  228   66046007            Psyr_2771            YP_235848.1          hypothetical protein
   3356907..3360491   -  1194  66046008            Psyr_2772            YP_235849.1          Fibronectin, type III
   3360547..3361158   -  203   66046009            Psyr_2773            YP_235850.1          hypothetical protein
-->3361185..3361763   -  192   66046010            Psyr_2774            YP_235851.1          Bacteriophage lambda tail assembly I
   3361820..3362155   -  111   66046011            Psyr_2775            YP_235852.1          hypothetical protein
   3362430..3363095   +  221   66046012            Psyr_2776            YP_235853.1          hypothetical protein
   3363125..3363889   -  254   66046013            Psyr_2777            YP_235854.1          JAB+NLP/P60
---------------------------------------
  ORGANISM  Desulfovibrio vulgaris subsp. vulgaris str. Hildenborough    accession no is AE017285.1 gi is 46449977
   cds                dir len   gi         gene     locus                pid                  product
   2249277..2249639   -  120   46449974            DVU2150              AAS96623.1           dnaK suppressor protein, putative
   2250828..2251187   -  119   46449975            DVU2151              AAS96624.1           conserved hypothetical protein
   2251197..2252213   -  338   46449976            DVU2152              AAS96625.1           hypothetical protein
-->2252215..2256255   -  1346  46449977            DVU2153              AAS96626.1           tail fiber protein, putative
   2256245..2256670   -  141   46449978            DVU2154              AAS96627.1           tNlpC solo ail assembly protein, putative
   2256670..2257095   -  141   46449979            DVU2155              AAS96628.1           hypothetical protein
   2257176..2257541   -  121   46449980            DVU2156              AAS96629.1           hypothetical protein
---------------------------------------
  ORGANISM  Salmonella enterica subsp. enterica serovar Typhi str. CT18  accession no is AL513384.1 gi is 16506034
   cds                dir len   gi         gene     locus                pid                  product
   37229..37552       -  107   16506031   HCM2.0050c                      CAD09917.1           hypothetical protein
   37667..40219       -  850   16506032   HCM2.0051c                      CAD09918.1           putative phage tail protein
   40302..44690       -  1462  16506033   HCM2.0052c                      CAD09919.1           putative phage tail protein
-->44705..45292       -  195   16506034   HCM2.0053c                      CAD09920.1           putative phage tail protein
   45280..46077       -  265   16506035   HCM2.0054c                      CAD09921.1           JAB+NlpC; putative phage protein
   46070..46801       -  243   16506036   HCM2.0055c                      CAD09922.1           putative phage tail protein
   46858..47193       -  111   16506037   HCM2.0056c                      CAD09923.1           putative phage protein
---------------------------------------
  ORGANISM  Salmonella enterica subsp. enterica serovar Choleraesuis str.        accession no is NC_006905.1 gi is 62179570
   cds                dir len   gi         gene     locus                pid                  product
   1110743..1111276   -  177   62179567   sodC     SC0997               YP_215984.1          Gifsy-2 prophage superoxide dismutase precursor
   1111366..1112061   +  231   62179568   vmtL     gpL, SC0998               YP_215985.1          Gifsy-2 prophage probable minor tail protein
   1112071..1112808   +  245   62179569   vtaK     JAB, SC0999               YP_215986.1          Gifsy-2 prophage probable tail assembly protein
-->1112745..1113410   +  221   62179570   vtaI     ThiS, SC1000               YP_215987.1          Gifsy-2 prophage probable tail assembly protein
   1116870..1117112   +  80    62179571            SC1001               YP_215988.1          hypothetical protein
   1117166..1119604   +  812   62179572   stf      SC1002               YP_215989.1          Gifsy-2 prophage probable tail fiber protein
   1119604..1120185   +  193   62179573   ycdD     SC1003               YP_215990.1          Gifsy-2 prophage tail fiber assembly
---------------------------------------
  ORGANISM  Salmonella enterica subsp. enterica serovar Choleraesuis str.        accession no is NC_006905.1 gi is 62179803
   cds                dir len   gi         gene     locus                pid                  product
   1340085..1340414   +  109   62179800   vmtM     SC1230               YP_216217.1          Gifsy-1 prophage VmtM
   1340424..1341122   +  232   62179801   vmtL     SC1231               YP_216218.1          Gifsy-1 prophage VmtL
   1341312..1341866   +  184   62179802   vtaK     SC1232               YP_216219.1          Gifsy-1 prophage VtaK
-->1341905..1342411   +  168   62179803   vtiI     SC1233               YP_216220.1          Gifsy-1 prophage VtiI
   1342474..1345836   +  1120  62179804   vhsJ     SC1234               YP_216221.1          Gifsy-1 prophage VhsJ
   1345875..1346117   +  80    62179805            SC1235               YP_216222.1          hypothetical protein
   1346171..1348849   +  892   62179806   stf      SC1236               YP_216223.1          side tail fiber protein

---------------------------------------
  ORGANISM  Campylobacter lari RM2100    accession no is NZ_AAFK01000001.1 gi is 57240561
   cds                dir len   gi         gene     locus                pid                  product
   78893..79993       +  366   57240564   dapE     CLA1531              ZP_00368513.1        succinyl-diaminopimelate desuccinylase
   80128..80886       +  252   57240563            CLA1530              ZP_00368512.1        conserved hypothetical protein
   80920..81111       +  63    57240562   thiS     CLA1529              ZP_00368511.1        thiamine biosynthesis protein ThiS
-->81111..81908       +  265   57240561            CLA1528              ZP_00368510.1        HesA/MoeB/ThiF family protein
   81908..82666       +  252   57240560   thiG     CLA1527              ZP_00368509.1        thiamin biosynthesis ThiG
   82668..83801       +  377   57240559   thiH     CLA1526              ZP_00368508.1        thiH protein VC0066
   83791..84393       +  200   57240558            CLA1525              ZP_00368507.1        probable transferase Cj1043c
---------------------------------------
  ORGANISM  Campylobacter coli RM2228    accession no is NZ_AAFL01000007.1 gi is 57168916
   cds                dir len   gi         gene     locus                pid                  product
   10488..11588       +  366   57168919   dapE     CCO1116              ZP_00368049.1        succinyl-diaminopimelate desuccinylase
   11597..14683       +  1028  57168918            CCO1115              ZP_00368048.1        adenine specific DNA methyltransferase
   14673..14864       +  63    57168917   thiS     CCO1114              ZP_00368047.1        thiamine biosynthesis protein ThiS
-->14864..15664       +  266   57168916            CCO1113              ZP_00368046.1        HesA/MoeB/ThiF family protein
   15668..16444       +  258   57168915            CCO1112              ZP_00368045.1        thiamin biosynthesis protein thiG Cj1045c
   16447..17592       +  381   57168914            CCO1111              ZP_00368044.1        thiH protein Cj1044c
   17582..18187       +  201   57168913            CCO1110              ZP_00368043.1        thiamine-phosphate pyrophosphorylase, putative
---------------------------------------
  ORGANISM  Campylobacter jejuni RM1221  accession no is AAW35515.1 gi is 57166736
   cds                dir len   gi         gene     locus                pid                  product
   1105290..1105895   -  201   57166733            CJE1187              AAW35512.1           thiamine-phosphate pyrophosphorylase, putative
   1105885..1107030   -  381   57166734   thiH     CJE1188              AAW35513.1           ThiH
   1107033..1107809   -  258   57166735   thiG     CJE1189              AAW35514.1           ThiG
-->1107812..1108615   -  267   57166736   thiF     CJE1190              AAW35515.1           thiamine biosynthesis protein ThiF
   1108612..1108803   -  63    57166737   thiS     CJE1191              AAW35516.1           thiamine biosynthesis protein ThiS
   1108813..1109910   -  365   57166738   dapE     CJE1192              AAW35517.1           succinyl-diaminopimelate desuccinylase
   1109915..1110514   -  199   57166739            CJE1193              AAW35518.1           transporter, LysE family
---------------------------------------
  ORGANISM  Campylobacter jejuni subsp. jejuni HB93-13   accession no is ZP_01070656.1 gi is 86152451
   cds                dir len   gi         gene     locus                pid                  product
   614838..615437     +  199   86152634            CJJHB9313_1066       ZP_01070839.1        transporter, LysE family
   615441..616538     +  365   86152589   dapE     CJJHB9313_1065       ZP_01070794.1        succinyl-diaminopimelate desuccinylase
   616548..616739     +  63    86153164   thiS     CJJHB9313_1064       ZP_01071369.1        thiamine biosynthesis protein ThiS
-->616736..617539     +  267   86152451   thiF     CJJHB9313_1063       ZP_01070656.1        thiamine biosynthesis protein ThiF
   617542..618318     +  258   86153113   thiG     CJJHB9313_1062       ZP_01071318.1        Thiazole biosynthesis protein ThiG
   618321..619466     +  381   86152623   thiH     CJJHB9313_1061       ZP_01070828.1        thiazole biosynthesis protein ThiH
   619456..620061     +  201   86152721            CJJHB9313_1060       ZP_01070926.1        thiamine-phosphate pyrophosphorylase, putative
---------------------------------------
  ORGANISM  Campylobacter jejuni subsp. jejuni CF93-6    accession no is NZ_AANJ01000008.1 gi is 86150511
   cds                dir len   gi         gene     locus                pid                  product
   51323..51928       -  201   86150443            CJJCF936_1126        ZP_01068668.1        thiamine-phosphate pyrophosphorylase, putative
   51918..53063       -  381   86150457   thiH     CJJCF936_1127        ZP_01068682.1        thiH protein
   53066..53512       -  148   86150421   thiG     CJJCF936_1128        ZP_01068646.1        thiG protein
-->53843..54646       -  267   86150511   thiF     CJJCF936_1129        ZP_01068736.1        thiamine biosynthesis protein ThiF
   54643..54834       -  63    86150485   thiS     CJJCF936_1130        ZP_01068710.1        thiamine biosynthesis protein ThiS
   54831..56246       -  471   86150444            CJJCF936_1131        ZP_01068669.1        dna methylase-type I restriction-modification
   56239..56772       -  177   86150461            CJJCF936_1132        ZP_01068686.1        conserved hypothetical protein
---------------------------------------
  ORGANISM  Campylobacter jejuni subsp. jejuni 260.94    accession no is NZ_AANK01000001.1 gi is 86150854
   cds                dir len   gi         gene     locus                pid                  product
   324959..325558     +  199   86150876            CJJ26094_1114        ZP_01069092.1        transporter, LysE family
   325562..326659     +  365   86151108   dapE     CJJ26094_1113        ZP_01069324.1        succinyl-diaminopimelate desuccinylase
   326669..326860     +  63    86150724   thiS     CJJ26094_1112        ZP_01068940.1        thiamine biosynthesis protein ThiS
-->326857..327660     +  267   86150854   thiF     CJJ26094_1111        ZP_01069070.1        thiamine biosynthesis protein ThiF
   327663..328439     +  258   86151062   thiG     CJJ26094_1110        ZP_01069278.1        thiG protein
   328442..329587     +  381   86150959   thiH     CJJ26094_1109        ZP_01069175.1        thiH protein
   329577..330182     +  201   86151069            CJJ26094_1108        ZP_01069285.1        thiamine-phosphate pyrophosphorylase, putative
---------------------------------------
  ORGANISM  Campylobacter jejuni subsp. jejuni 84-25     accession no is ZP_01083242.1 gi is 87132835
   cds                dir len   gi         gene     locus                pid                  product
   676456..677055     +  199   87132832            Cjejjeju_01000705    ZP_01083239.1        COG1280: Putative threonine efflux protein
   677059..678156     +  365   87132833            Cjejjeju_01000706    ZP_01083240.1        COG0624: Acetylornithine
   678166..678357     +  63    87132834            Cjejjeju_01000707    ZP_01083241.1        COG2104: Sulfur transfer protein involved in
-->678354..679157     +  267   87132835            Cjejjeju_01000708    ZP_01083242.1        COG0476: Dinucleotide-utilizing enzymes involved
   679160..679936     +  258   87132836            Cjejjeju_01000709    ZP_01083243.1        COG2022: Uncharacterized enzyme of thiazole
   679939..681084     +  381   87132837            Cjejjeju_01000710    ZP_01083244.1        COG1060: Thiamine biosynthesis enzyme ThiH and
   681074..681679     +  201   87132838            Cjejjeju_01000711    ZP_01083245.1        COG0352: Thiamine monophosphate synthase
---------------------------------------
  ORGANISM  Pelobacter propionicus DSM 2379      accession no is NZ_AAJH01000004.1 gi is 71837115
   cds                dir len   gi         gene     locus                pid                  product
   89088..90446       -  452   71837112            PproDRAFT_3024       ZP_00676878.1        Transposase, IS4
   90675..91427       +  250   71837113            PproDRAFT_3025       ZP_00676879.1        Cyclic nucleotide-binding domain:Bacterial
   91448..92473       -  341   71837114            PproDRAFT_3026       ZP_00676880.1        GGDEF
-->92597..93400       -  267   71837115            PproDRAFT_3027       ZP_00676881.1        UBA/THIF-type NAD/FAD binding fold
   93496..94449       -  317   71837116            PproDRAFT_3028       ZP_00676882.1        Cysteine synthase K/M:Cysteine synthase K
   94554..95699       -  381   71837117            PproDRAFT_3029       ZP_00676883.1        Cystathionine gamma-synthase
   95696..96847       -  383   71837118            PproDRAFT_3030       ZP_00676884.1        Cystathionine gamma-synthase
---------------------------------------
  ORGANISM  Pelobacter carbinolicus DSM 2380     accession no is ABA87870.1 gi is 77544308
   cds                dir len   gi         gene     locus                pid                  product
   739855..740967     -  370   77544305            Pcar_0608            ABA87867.1           thiH protein
   740964..741740     -  258   77544306            Pcar_0609            ABA87868.1           thiamine biosynthesis protein ThiG
   741770..741973     -  67    77544307            Pcar_0610            ABA87869.1           thiamine biosynthesis protein ThiS
-->741991..742797     -  268   77544308            Pcar_0611            ABA87870.1           molybdopterin biosynthesis protein MoeB
   743207..743509     +  100   77544309            Pcar_0612            ABA87871.1           hypothetical protein
   743651..743836     +  61    77544310            Pcar_0613            ABA87872.1           conserved hypothetical protein
   744008..745075     -  355   77544311            Pcar_0614            ABA87873.1           phospho-2-dehydro-3-deoxyheptonate aldolase
---------------------------------------
  ORGANISM  Desulfuromonas acetoxidans DSM 684   accession no is NZ_AAEW01000027.1 gi is 68178158
   cds                dir len   gi         gene     locus                pid                  product
   5844..6041         -  65    68178155            DaceDRAFT_1953       ZP_00551287.1        regulatory protein, MerR
   6330..7289         +  319   68178156            DaceDRAFT_1954       ZP_00551288.1        GGDEF
   7301..7621         -  106   68178157            DaceDRAFT_1955       ZP_00551289.1        hypothetical protein
-->7981..8799         +  272   68178158            DaceDRAFT_1956       ZP_00551290.1        UBA/THIF-type NAD/FAD binding fold
   8801..9001         +  66    68178159            DaceDRAFT_1957       ZP_00551291.1        ThiS, thiamine-biosynthesis
   9086..9859         +  257   68178160            DaceDRAFT_1958       ZP_00551292.1        IMP dehydrogenase/GMP reductase:Thiazole
   9862..10986        +  374   68178161            DaceDRAFT_1959       ZP_00551293.1        Biotin and thiamin synthesis associated
---------------------------------------
  ORGANISM  Clostridium perfringens str. 13      accession no is BAB81308.1 gi is 18145265
   cds                dir len   gi         gene     locus                pid                  product
   1872446..1873027   -  193   18145262   thiE                          BAB81305.1           thiamin phosphate pyrophosphorylase
   1873057..1874160   -  367   18145263   thiH                          BAB81306.1           thiamin biosynthesis protein
   1874173..1874937   -  254   18145264   thiG                          BAB81307.1           thiamin biosynthesis protein
-->1875061..1875870   -  269   18145265   CPE1602                       BAB81308.1           probable molybdopterin biosynthesis protein
   1875946..1876140   -  64    18145266   CPE1603                       BAB81309.1           conserved hypothetical protein
   1876416..1878116   -  566   18145267   CPE1604                       BAB81310.1           probable multidrug-efflux transporter
   1878452..1879261   -  269   18145268   CPE1605                       BAB81311.1           conserved hypothetical protein
---------------------------------------
  ORGANISM  Clostridium beijerincki NCIMB 8052   accession no is NZ_AALO01000039.1 gi is 82748786
   cds                dir len   gi         gene     locus                pid                  product
   24156..24347       -  63    82748783            CbeiDRAFT_0773       ZP_00911260.1        conserved hypothetical protein
   24437..25303       -  288   82748784            CbeiDRAFT_0774       ZP_00911261.1        similar to ATPase of the PP-loop superfamily
   25487..26137       -  216   82748785            CbeiDRAFT_0775       ZP_00911262.1        regulatory protein tenI
-->26145..26948       -  267   82748786            CbeiDRAFT_0776       ZP_00911263.1        UBA/THIF-type NAD/FAD binding fold
   27573..28169       +  198   82748787            CbeiDRAFT_0777       ZP_00911264.1        conserved hypothetical protein
   28439..31030       -  863   82748788            CbeiDRAFT_0778       ZP_00911265.1        ATPas
   31297..31683       +  128   82748789            CbeiDRAFT_0779       ZP_00911266.1        CBS
---------------------------------------
  ORGANISM  Clostridium tetani E88       accession no is AAO36281.1 gi is 28203841
   cds                dir len   gi         gene     locus                pid                  product
   1860244..1860858   -  204   28203838   tenI     CTC01746             AAO36278.1           regulatory protein tenI
   1861171..1861923   -  250   28203839   thiH     CTC01748             AAO36279.1           thiH protein
   1861943..1862710   -  255   28203840   thiG     CTC01749             AAO36280.1           thiG protein
-->1862722..1863525   -  267   28203841   ThiS+ThiFCTC01750             AAO36281.1           molybdopterin biosynthesis protein moeB
   1863853..1864521   -  222   28203842   ThiE     CTC01751             AAO36282.1           thiamin-phosphate pyrophosphorylase
   1864475..1865299   -  274   28203843   ThiM     CTC01752             AAO36283.1           hydroxyethylthiazole kinase
   1865320..1866210   -  296   28203844   ThiD     CTC01753             AAO36284.1           phosphomethylpyrimidine kinase
---------------------------------------
  ORGANISM  Alkaliphilus metalliredigenes QYMF   accession no is NZ_AAKU01000002.1 gi is 77683437
   cds                dir len   gi         gene     locus                pid                  product
   82855..83499       -  214   77683434            AmetDRAFT_3944       ZP_00798883.1        conserved hypothetical protein
   83694..84554       +  286   77683435            AmetDRAFT_3945       ZP_00798884.1        hypothetical protein
   84857..85051       +  64    77683436            AmetDRAFT_3946       ZP_00798885.1        ThiS, thiamine-biosynthesis
-->85053..85859       +  268   77683437            AmetDRAFT_3947       ZP_00798886.1        UBA/THIF-type NAD/FAD binding fold
   85874..86650       +  258   77683438            AmetDRAFT_3948       ZP_00798887.1        Thiazole biosynthesis
   86650..87753       +  367   77683439            AmetDRAFT_3949       ZP_00798888.1        Radical SAM:Biotin and thiamin synthesis
   87777..89090       +  437   77683440            AmetDRAFT_3950       ZP_00798889.1        Thiamine biosynthesis protein ThiC
---------------------------------------
  ORGANISM  Clostridium acetobutylicum ATCC 824  accession no is AAK80865.1 gi is 15025973
   cds                dir len   gi         gene     locus                pid                  product
   3054432..3055019   -  195   15025970   tenI     CA_C2920             AAK80862.1           Thiamine monophosphate synthase
   3055016..3056122   -  368   15025971   thiH     CA_C2921             AAK80863.1           Thiamine biosynthesis enzyme, thiH
   3056136..3056903   -  255   15025972   thiG     CA_C2922             AAK80864.1           Uncharacterized enzyme of thiazol biosynthesis
-->3056923..3057723   -  266   15025973            CA_C2923             AAK80865.1           Dinucleotide-utilizing enzyme involved in
   3057724..3057918   -  64    15025974   thiS     CA_C2924             AAK80866.1           Uncharacterized protein, possibly involved in
   3058364..3058849   +  161   15025975            CA_C2925             AAK80867.1           HD superfamily hydrolase
   3058860..3059666   +  268   15025976   sul      CA_C2926             AAK80868.1           Dihydropteroate synthase
---------------------------------------


  ORGANISM  Bradyrhizobium sp. BTAi1     accession no is ZP_00857668.1 gi is 78693154
   cds                dir len   gi         gene     locus                pid                  product
   819805..820359     -  184   78693149            BradDRAFT_6552       ZP_00857663.1        hypothetical protein
   820343..821332     -  329   78693150            BradDRAFT_6553       ZP_00857664.1        nagAa, RSc1091; probable ferredoxin
   821390..822415     -  341   78693151            BradDRAFT_6554       ZP_00857665.1        hypothetical protein
   822438..822737     -  99    78693152            BradDRAFT_6555       ZP_00857666.1        hypothetical protein
   822747..823127     -  126   78693153            BradDRAFT_6556       ZP_00857667.1        similar to Ferredoxin subunits of nitrite
-->823120..823365     -  81    78693154            BradDRAFT_6557       ZP_00857668.1        hypothetical protein
   823462..824946     -  494   78693155            BradDRAFT_6558       ZP_00857669.1        hypothetical protein
   825417..825740     -  107   78693156            BradDRAFT_6559       ZP_00857670.1        ferrodoxin
   825757..826518     -  253   78693157            BradDRAFT_6560       ZP_00857671.1        putative 3-oxoacyl -(acyl-carrier protein)
   826515..827132     -  205   78693158            BradDRAFT_6561       ZP_00857672.1        conserved hypothetical protein
   827129..828574     -  481   78693159            BradDRAFT_6562       ZP_00857673.1        Succinate-semialdehyde dehydrogenase (NAD(P)+)
---------------------------------------
  ORGANISM  Pseudomonas sp. OX1  accession no is AY621080.1 gi is 48094248
   cds                dir len   gi         gene     locus                pid                  product
   1..1497            +  498   48094247   touA                          AAT40431.1           toluene o-xylene monooxygenase component
-->1533..1793         +  86    48094248   touB                          AAT40432.1           toluene o-xylene monooxygenase component
   1808..2146         +  112   48094249   touC                          AAT40433.1           toluene o-xylene monooxygenase component
   2333..2665         +  110   48094250   touD                          AAT40434.1           toluene o-xylene monooxygenase component
   2696..3688         +  330   48094251   touE                          AAT40435.1           toluene o-xylene monooxygenase component
   3770..4795         +  341   48094252   touF                          AAT40436.1           toluene o-xylene monooxygenase component
---------------------------------------
  ORGANISM  Ralstonia metallidurans CH34         accession no is ZP_00595380.1 gi is 68556036
   cds                dir len   gi         gene     locus                pid                  product
   854242..854367     -  41    68556035            RmetDRAFT_4051       ZP_00595379.1        glutathione S-transferase
   854433..855455     -  340   68556001            RmetDRAFT_4017       ZP_00595345.1        Ferredoxin:Oxidoreductase
   855555..856541     -  328   68555975            RmetDRAFT_3991       ZP_00595319.1        Methane/phenol/toluene hydroxylase
   856596..856856     -  86    68555976            RmetDRAFT_3992       ZP_00595320.1        Monooxygenase component MmoB/DmpM
   856951..857286     -  111   68555977            RmetDRAFT_3993       ZP_00595321.1        Rieske [2Fe-2S] region
-->857342..857650     -  102   68556036            RmetDRAFT_4052       ZP_00595380.1        Toluene-4-monooxygenase system B
   857677..859179     -  500   68555978            RmetDRAFT_3994       ZP_00595322.1        Methane/phenol/toluene hydroxylase:YHS
   859239..859430     -  63    68555979            RmetDRAFT_3995       ZP_00595323.1        4-oxalocrotonate tautomerase
   859442..860230     -  262   68555980            RmetDRAFT_3996       ZP_00595324.1        4-oxalocrotonate decarboxylase
   860227..861273     -  348   68555981            RmetDRAFT_3997       ZP_00595325.1        HMG-CoA lyase-like:Aminotransferase, class-II
   861293..862204     -  303   68555982            RmetDRAFT_3998       ZP_00595326.1        Acetaldehyde dehydrogenase
---------------------------------------
  ORGANISM  Rhodococcus sp. AD45         accession no is AJ249207.1 gi is 5911739
   cds                dir len   gi         gene     locus                pid                  product
   57..1274           +  405   5911734    isoG                          CAB55821.1           putative racemase
   1291..1971         +  226   5911735    isoH                          CAB55822.1           1-hydroxy-2-glutathionyl-2-methyl-3-butene
   2038..2754         +  238   5911736    isoI                          CAB55823.1           glutathione S-transferase
   2789..3490         +  233   5911737    isoJ                          CAB55824.1           glutathione S-transferase
   3796..5340         +  514   5911738    isoA                          CAB55825.1           putative isoprene monooxygenase alpha subunit
-->5376..5660         +  94    5911739    isoB                          CAB55826.1           putative isoprene monooxygenase gamma subunit
   5653..5997         +  114   5911740    isoC                          CAB55827.1           putative ferredoxin
   6016..6348         +  110   5911741    isoD                          CAB55828.1           putative effector/coupling protein
   6345..7373         +  342   5911742    isoE                          CAB55829.1           putative isoprene monooxygenase beta subunit
   7387..8424         +  345   5911743    isoF                          CAB55830.1           putative reductase
---------------------------------------
  ORGANISM  Pseudomonas mendocina        accession no is AY552601.1 gi is 45479222
   cds                dir len   gi         gene     locus                pid                  product
   37..1539           +  500   45479221   tmoA                          AAS66660.1           alpha hydroxylase
-->1558..1812         +  84    45479222   tmoB                          AAS66661.1           gamma hydroxylase
   1818..2156         +  112   45479223   tmoC                          AAS66662.1           ferredoxin
   2217..2528         +  103   45479224   tmoD                          AAS66663.1           effector
   2539..3522         +  327   45479225   tmoE                          AAS66664.1           beta hydroxylase
   3598..4578         +  326   45479226   tmoF                          AAS66665.1           reductase
---------------------------------------
  ORGANISM  Pseudomonas aeruginosa       accession no is D83068.1 gi is 1754624
   cds                dir len   gi         gene     locus                pid                  product
   324..1826          +  500   1754623    bmoA                          BAA11761.1           benzene monooxygenase oxygenase subunit
-->1906..2172         +  88    1754624    bmoB                          BAA11762.1
   2221..2556         +  111   1754625    bmoC                          BAA11763.1           benzene monooxygenase ferredoxin
   2626..3069         +  147   1754626    bmoD1                         BAA11764.1
---------------------------------------
  ORGANISM  Dechloromonas aromatica RCB  accession no is AAZ48547.1 gi is 71849051
   cds                dir len   gi         gene     locus                pid                  product
   4098547..4099941   -  464   71849046            Daro_3814            AAZ48542.1           Membrane protein involved in aromatic
   4100009..4101025   -  338   71849047            Daro_3815            AAZ48543.1           Ferredoxin:Oxidoreductase
   4101098..4102084   -  328   71849048            Daro_3816            AAZ48544.1           Methane/phenol/toluene hydroxylase
   4102141..4102581   -  146   71849049            Daro_3817            AAZ48545.1           Monooxygenase component MmoB/DmpM
   4102619..4102954   -  111   71849050            Daro_3818            AAZ48546.1           Rieske (2Fe-2S) region
-->4103002..4103268   -  88    71849051            Daro_3819            AAZ48547.1           Toluene-4-monooxygenase system B
   4103332..4104837   -  501   71849052            Daro_3820            AAZ48548.1           Methane/phenol/toluene hydroxylase:YHS
   4105108..4107387   -  759   71849053            Daro_3821            AAZ48549.1           Response regulator receiver:ATP-binding region,
   4107965..4109254   -  429   71849054            Daro_3822            AAZ48550.1           RNA-directed DNA polymerase (Reverse
   4109905..4110492   -  195   71849055            Daro_3823            AAZ48551.1           hypothetical protein
   4110880..4112361   -  493   71849056            Daro_3824            AAZ48552.1           Transposase, IS4
---------------------------------------
  ORGANISM  Frankia sp. CcI3     accession no is ABD09601.1 gi is 86565792
   cds                dir len   gi         gene     locus                pid                  product
   245245..246375     -  376   86565787            Francci3_0207        ABD09596.1           hypothetical protein
   246568..247401     -  277   86565788            Francci3_0208        ABD09597.1           transcriptional regulator, XRE family
   247515..248108     +  197   86565789            Francci3_0209        ABD09598.1           NADPH-dependent FMN reductase
   248256..248930     -  224   86565790            Francci3_0210        ABD09599.1           transcriptional regulator, MarR family
   249173..250768     +  531   86565791            Francci3_0211        ABD09600.1           methane/phenol/toluene hydroxylase
-->250881..251129     +  82    86565792            Francci3_0212        ABD09601.1           Toluene-4-monooxygenase system B
   251167..251547     +  126   86565793            Francci3_0213        ABD09602.1           Rieske (2Fe-2S) protein
   251544..251849     +  101   86565794            Francci3_0214        ABD09603.1           monooxygenase component MmoB/DmpM
   251852..252877     +  341   86565795            Francci3_0215        ABD09604.1           methane/phenol/toluene hydroxylase
   252938..253426     +  162   86565796            Francci3_0216        ABD09605.1           carbonic anhydrase
   253701..256295     -  864   86565797            Francci3_0217        ABD09606.1           RNA binding S1
---------------------------------------
  ORGANISM  Xanthobacter autotrophicus Py2       accession no is AJ012090.1 gi is 4210875
   cds                dir len   gi         gene     locus                pid                  product
   330..1823          +  497   4210874    xamoA                         CAA09911.1           oxygenase alpha subunit
-->1860..2126         +  88    4210875    xamoB                         CAA09912.1           oxygenase gamma subunit
   2123..2491         +  122   4210876    xamoC                         CAA09913.1           ferredoxin
   2494..2799         +  101   4210877    xamoD                         CAA09914.1           coupling/effector protein
   2831..3856         +  341   4210878    xamoE                         CAA09915.1           oxygenase beta subunit
   3912..4895         +  327   4210879    xamoF                         CAA09916.1           reductase
---------------------------------------
  ORGANISM  Ralstonia eutropha JMP134    accession no is AAZ65023.1 gi is 72122837
   cds                dir len   gi         gene     locus                pid                  product
   2505787..2507565   -  592   72122832            Reut_B5673           AAZ65018.1           Helix-turn-helix, Fis-type
   2507800..2508798   -  332   72122833            Reut_B5674           AAZ65019.1           Ferredoxin:Oxidoreductase
   2508847..2509845   -  332   72122834            Reut_B5675           AAZ65020.1           Methane/phenol/toluene hydroxylase
   2509858..2510175   -  105   72122835            Reut_B5676           AAZ65021.1           Monooxygenase component MmoB/DmpM
   2510195..2510545   -  116   72122836            Reut_B5677           AAZ65022.1           Rieske (2Fe-2S) region
-->2510538..2510798   -  86    72122837            Reut_B5678           AAZ65023.1           Toluene-4-monooxygenase system B
   2510823..2512328   -  501   72122838            Reut_B5679           AAZ65024.1           Methane/phenol/toluene hydroxylase:YHS
   2512944..2513153   +  69    72122839            Reut_B5680           AAZ65025.1           Phenol hydroxylase subunit
   2513207..2514214   +  335   72122840            Reut_B5681           AAZ65026.1           Methane/phenol/toluene hydroxylase
   2514253..2514522   +  89    72122841            Reut_B5682           AAZ65027.1           Monooxygenase component MmoB/DmpM
   2514572..2516116   +  514   72122842            Reut_B5683           AAZ65028.1           TmoA-like; Methane/phenol/toluene hydroxylase:YHS
   2516113..2516481   +  122   72122843            Reut_B5684           AAZ65029.1           Phenol hydroxylase conserved region
   2516486..2517550   +  354   72122844            Reut_B5685           AAZ65030.1           Ferredoxin:Oxidoreductase
   2517567..2517932   +  121   72122845            Reut_B5686           AAZ65031.1           Ferredoxin
   2517955..2518899   +  314   72122846            Reut_B5687           AAZ65032.1           Catechol 2,3-dioxygenase
---------------------------------------
  ORGANISM  Ralstonia pickettii  accession no is AY541701.1 gi is 44893909
   cds                dir len   gi         gene     locus                pid                  product
   293..1798          +  501   44893908   tbuA1                         AAS48547.1           alpha hydroxylase subunit
-->1823..2083         +  86    44893909   tbuU                          AAS48548.1           gamma hydroxylase subunit
   2091..2426         +  111   44893910   tbuB                          AAS48549.1           ferredoxin subunit
   2446..2760         +  104   44893911   tbuV                          AAS48550.1           effector subunit
   2773..3762         +  329   44893912   tbuA2                         AAS48551.1           beta hydroxylase subunit
   3827..4831         +  334   44893913   tbuC                          AAS48552.1           reductase subunit
---------------------------------------
  ORGANISM  Burkholderia cepacia         accession no is AF001356.1 gi is 2150114
   cds                dir len   gi         gene     locus                pid                  product
   766..1602          +  278   2150112    tbhG                          AAB58739.1           4-oxalocrotonate decarboxylase
   1818..3323         +  501   2150113    tbhA                          AAB58740.1           toluene-3-monooxygenase oxygenase subunit 1
-->3392..3661         +  89    2150114    tbhB                          AAB58741.1           TbhB
   3710..4009         +  99    2150115    tbhC                          AAB58742.1           ferredoxin
   4085..4399         +  104   2150116    tbhD                          AAB58743.1           TbhD
   4455..5453         +  332   2150117    tbhE                          AAB58744.1           toluene-3-monooxygenase oxygenase subunit 2
   5553..6578         +  341   2150118    tbhF                          AAB58745.1           putative oxidoreductase
---------------------------------------

  ORGANISM  Pyrococcus abyssi GE5        accession no is NP_127095.2 gi is 33356787
   cds                dir len   gi         gene     locus                pid                  product
   1370521..1371963   +  480   14521615            PAB0938              NP_127091.1          hypothetical protein
   1372000..1372608   +  202   14521616            PAB0939              NP_127092.1          hypothetical protein
   1372667..1373959   +  430   14521617            PAB0940              NP_127093.1          hypothetical protein
   1373889..1374179   +  96    33356786            PAB0940.1n           NP_877694.1          hypothetical protein
   1374182..1374583   +  133   14521618            PAB0941              NP_127094.1          hypothetical protein
-->1374580..1374864   -  94    33356787   moaD     PAB3357              NP_127095.2          molybdopterin converting factor, subunit 1
   1374851..1376059   -  402   14521620   moeA-1   PAB1436              NP_127096.1          molybdenum cofactor biosynthesis protein
   1376190..1376624   -  144   14521621            PAB1435              NP_127097.1          putative endonuclease.
   1376626..1377513   -  295   14521622            PAB1434              NP_127098.1          methionine aminopeptidase
   1377555..1378307   -  250   14521623            PAB1433              NP_127099.1          hypothetical protein
   1378366..1379526   +  386   14521624   nifs-like PAB0943              NP_127100.1          capreomycin acetyltransferase or nifs-like
---------------------------------------
  ORGANISM  Pyrococcus abyssi GE5        accession no is AJ248287.2 gi is 5458838
   cds                dir len   gi         gene     locus                pid                  product
   174593..175942     -  449   5458833                                  CAB50320.1           Multi antimicrobial extrusion (MatE) protein
   176071..177513     +  480   5458834                                  CAB50321.1           Hypothetical protein
   177550..178158     +  202   5458835                                  CAB50322.1           Hypothetical protein
   178217..179509     +  430   5458836                                  CAB50323.1           Hypothetical protein
   179732..180133     +  133   5458837                                  CAB50324.1           Hypothetical protein
-->180130..180399     -  89    5458838                                  CAB50325.1           moaD molybdopterin synthase, small subunit
   180401..181609     -  402   5458839                                  CAB50326.1           moeA-1 molybdenum cofactor biosynthesis protein
   181740..182174     -  144   5458840                                  CAB50327.1           Archaeal endonuclease, putative
   182176..183063     -  295   5458841                                  CAB50328.1           map methionine aminopeptidase (EC 3.4.11.18)
   183105..183857     -  250   5458842                                  CAB50329.1           Hypothetical protein
   183916..185076     +  386   5458843    nifs-like                      CAB50330.1           Aminotransferase, class-V
---------------------------------------
  ORGANISM  Pyrococcus horikoshii OT3    accession no is NP_877770.1 gi is 33359306
   cds                dir len   gi         gene     locus                pid                  product
   515244..516617     +  457   14590474            PH0578               NP_142542.1          hypothetical protein
   516644..517258     +  204   14590475            PH0579               NP_142543.1          hypothetical protein
   517316..518611     +  431   14590476            PH0580               NP_142544.1          hypothetical protein
   518625..518840     +  71    33359305            PH0580.1n            NP_877769.1          hypothetical protein
   518837..519238     +  133   14590477            PH0581               NP_142545.1          hypothetical protein
-->519235..519504     -  89    33359306            PH0581.1n            NP_877770.1          putative molybdopterin converting factor,
   519506..520714     -  402   14590478            PH0582               NP_142546.1          molybdopterin biosynthesis moea protein
   520815..521771     +  318   14590479            PH0583               NP_142547.1          hypothetical protein
   521768..522697     -  309   14590480            PH0584               NP_142548.1          modification methylase
   523088..523243     +  51    33359307            PH0584.1n            NP_877771.1          hypothetical protein
   523240..524382     +  380   14590481            PH0585               NP_142549.1          neutral protease
---------------------------------------
  ORGANISM  Pyrococcus furiosus DSM 3638         accession no is AE010177.1 gi is 18892532
   cds                dir len   gi         gene     locus                pid                  product
   join(84..514,514..955) +  290   18892528   PF0539                        AAL80663.1           transposase
   1100..1834         +  244   18892529   PF0540                        AAL80664.1           hypothetical protein
   2035..2922         +  295   18892530   PF0541                        AAL80665.1           methionine aminopeptidase (map) (peptidase m)
   3079..4284         +  401   18892531   PF0542                        AAL80666.1           molybdenum cofactor biosynthesis protein
-->4296..4568         +  90    18892532   PF0543                        AAL80667.1           molybdopterin converting factor, subunit 1
   4565..4966         -  133   18892533   PF0544                        AAL80668.1           hypothetical protein
   4959..5252         -  97    18892534   PF0545                        AAL80669.1           hypothetical protein
   5212..6504         -  430   18892535   PF0546                        AAL80670.1           hypothetical protein
   6724..8070         -  448   18892536   PF0547                        AAL80671.1           hypothetical protein
   8311..8538         +  75    18892537   PF0548                        AAL80672.1           hydrogenase expression/formation protein
---------------------------------------
  ORGANISM  Pyrococcus abyssi GE5        accession no is AJ248286.2 gi is 5458384
   cds                dir len   gi         gene     locus                pid                  product
   12446..12898       +  150   5458379                                  CAB49867.1           Hypothetical protein
   12898..14691       +  597   5458380                                  CAB49868.1           Hypothetical protein
   15078..16319       +  413   5458381                                  CAB49869.1           Sugar-phosphate nucleotidyl transferase
   16381..17697       +  438   5458382    aspS                          CAB49870.1           aspS aspartyl-tRNA synthetase
   17819..19642       +  607   5458383    aor-2                         CAB49871.1           aor-2 tungsten-containing aldehyde ferredoxin
-->19676..19930       +  84    5458384    moaD-like                      CAB49872.1           moaD-like molybdopterin converting factor
   19931..21052       +  373   5458385    cmo                           CAB49873.1           cmo tungsten-containing aldehyde ferredoxin
   21049..21360       +  103   5458386                                  CAB49874.1           Hypothetical protein
   21343..22182       -  279   5458387                                  CAB49875.1           Hypothetical protein
   22245..22790       +  181   5458388    d1                            CAB49876.1           ubiX 3-octaprenyl-4-hydroxybenzoate
   22783..23271       +  162   5458389    b0652                         CAB49877.1           Transcriptional regulatory protein, Lrp-AsnC
---------------------------------------
  ORGANISM  Pyrococcus furiosus DSM 3638         accession no is AE010158.1 gi is 18892299
   cds                dir len   gi         gene     locus                pid                  product
   5294..5620         -  108   18892294   PF0340                        AAL80464.1           putative HTH transcription regulator
   5815..6783         +  322   18892295   PF0341                        AAL80465.1           hypothetical protein
   6755..7438         -  227   18892296   PF0342                        AAL80466.1           hypothetical protein
   7542..8807         -  421   18892297   PF0343                        AAL80467.1           s-adenosylhomocysteinase
   8848..9981         -  377   18892298   PF0344                        AAL80468.1           tungsten-containing aldehyde ferredoxin
-->9983..10231        -  82    18892299   PF0345                        AAL80469.1           molybdopterin converting factor, subunit 1
   10280..12097       -  605   18892300   PF0346                        AAL80470.1           aldehyde:ferredoxin oxidoreductase (aor)
---------------------------------------
  ORGANISM  Sulfolobus tokodaii str. 7   accession no is BAB65522.1 gi is 15621527
   cds                dir len   gi         gene     locus                pid                  product
   518388..520115     +  575   15621522   ST0522                        BAB65517.1           575aa long conserved hypothetical protein
   520096..521355     -  419   15621523   ST0523                        BAB65518.1           419aa long conserved hypothetical protein
   521352..521876     -  174   15621524   ST0524                        BAB65519.1           174aa long inorganic pyrophosphatase
   521893..522432     -  179   15621525   ST0525                        BAB65520.1           179aa long conserved hypothetical protein
   522453..523799     -  448   15621526   ST0526                        BAB65521.1           448aa long hypothetical phosphomethylpyrimidine
-->523777..524487     -  236   15621527   ST0527                        BAB65522.1           236aa long hypothetical molybdopterin converting
   524522..524941     +  139   15621528   ST0528                        BAB65523.1           139aa long hypothetical protein
   524938..527214     -  758   15621529   ST0529                        BAB65524.1           758aa long hypothetical protein
   527260..528009     +  249   15621530   ST0530                        BAB65525.1           249aa long conserved hypothetical protein
   527964..528764     +  266   15621531   ST0532                        BAB65526.1           266aa long conserved hypothetical protein
   528743..529546     -  267   15621532   ST0533                        BAB65527.1           267aa long hypothetical 3-methyl-2-oxobutanoate
---------------------------------------
  ORGANISM  Sulfolobus acidocaldarius DSM 639    accession no is AAY80314.1 gi is 68567385
   cds                dir len   gi         gene     locus                pid                  product
   757834..758526     +  230   68567380            Saci_0947            AAY80309.1           conserved protein
   758408..759214     +  268   68567381            Saci_0948            AAY80310.1           3-methyl-2-oxobutanoate
   759193..759951     -  252   68567382            Saci_0949            AAY80311.1           conserved Archaeal protein
   759948..760697     -  249   68567383            Saci_0950            AAY80312.1           conserved Archaeal protein
   760719..761144     -  141   68567384            Saci_0951            AAY80313.1           conserved protein
-->761181..761888     +  235   68567385            Saci_0952            AAY80314.1           molybdenum cofactor biosynthesis protein D/E
   761866..763209     +  447   68567386            Saci_0953            AAY80315.1           conserved protein
   763213..763746     +  177   68567387            Saci_0954            AAY80316.1           conserved Archaeal protein
   763773..764294     +  173   68567388   ppa      Saci_0955            AAY80317.1           inorganic pyrophosphatase
   764375..765568     +  397   68567389            Saci_0956            AAY80318.1           conserved protein
   765569..766651     -  360   68567390            Saci_0957            AAY80319.1           GTPase-like protein
---------------------------------------
  ORGANISM  Sulfolobus solfataricus P2   accession no is AE006840.1 gi is 13815697
   cds                dir len   gi         gene     locus                pid                  product
   533..1813          -  426   13815693   icaA     SSO2389              AAK42537.1           Glucosaminyltransferase, intercellular adhesion
   1810..2328         -  172   13815694   ppa      SSO2390              AAK42538.1           Inorganic pyrophosphatase, putative (ppa)
   2345..2893         -  182   13815695            SSO2391              AAK42539.1           Conserved hypothetical protein
   2911..4281         -  456   13815696   thiD-2   SSO2393              AAK42540.1           Phosphomethylpyrimidine kinase (thiD-2)
-->4242..4937         -  231   13815697   moaE     SSO2394              AAK42541.1           Molybdenum cofactor biosynthesis protein E
   4975..5430         +  151   13815698            SSO2395              AAK42542.1           Hypothetical protein
   5421..7676         -  751   13815699            SSO2398              AAK42543.1           Iron-sulfur protein, putative
   7658..8473         +  271   13815700            SSO2397              AAK42544.1           Conserved hypothetical protein
   8428..9228         +  266   13815701            SSO2399              AAK42545.1           Conserved hypothetical protein
   9207..10010        -  267   13815702   panB     SSO2400              AAK42546.1           Ketopantoate hydroxymethyltransferase (panB)
---------------------------------------
  ORGANISM  Pyrobaculum aerophilum str. IM2      accession no is AE009782.1 gi is 18159566
   cds                dir len   gi         gene     locus                pid                  product
   3395..4165         -  256   18159561   PAE0720                       AAL62977.1           proliferating-cell nuclear antigen homolog
   4259..5299         -  346   18159562   PAE0721                       AAL62978.1           electron transfer flavoprotein alpha subunit
   5303..6097         -  264   18159563   PAE0722                       AAL62979.1           electron transfer flavoprotein beta subunit
   6102..6392         -  96    18159564   PAE0723                       AAL62980.1           ferredoxin like protein
   6389..7663         -  424   18159565   PAE0725                       AAL62981.1           electron transfer flavoprotein-quinone
-->7890..8579         +  229   18159566   PAE0727                       AAL62982.1           molybdenum cofactor biosynthesis protein D/E
   8560..8946         -  128   18159567   PAE0728                       AAL62983.1           hypothetical protein
   9008..9784         +  258   18159568   PAE0729                       AAL62984.1           conserved hypothetical protein
   9942..10316        +  124   18159569   PAE0730                       AAL62985.1           hypothetical protein
   10343..10579       +  78    18159570   PAE0731                       AAL62986.1           transcriptional regulatory protein, conjectural
   10647..10850       +  67    18159571   PAE0732                       AAL62987.1           hypothetical protein
---------------------------------------
  ORGANISM  Methanosaeta thermophila PT  accession no is NZ_AAOR01000001.1 gi is 88950646
   cds                dir len   gi         gene     locus                pid                  product
   732348..732623     -  91    88950641            MtheDRAFT_1221       ZP_01153215.1        RNA polymerase, dimerisation
   732634..733227     -  197   88950642            MtheDRAFT_1222       ZP_01153216.1        RNA binding S1
   733196..733825     -  209   88950643            MtheDRAFT_1223       ZP_01153217.1        Putative RNA methylase:Methyltransferase small
   733947..735701     -  584   88950644            MtheDRAFT_1224       ZP_01153218.1        4Fe-4S ferredoxin, iron-sulfur binding:ABC
   735711..736130     -  139   88950645            MtheDRAFT_1225       ZP_01153219.1        conserved hypothetical protein
-->736614..737006     +  130   88950646            MtheDRAFT_1226       ZP_01153220.1        MoaD, archaeal
   737007..737402     +  131   88950647            MtheDRAFT_1227       ZP_01153221.1        Molybdopterin biosynthesis MoaE
   737381..737698     +  105   88950807            MtheDRAFT_1387       ZP_01153381.1        CutA1 divalent ion tolerance protein
   737952..738419     +  155   88950808            MtheDRAFT_1388       ZP_01153382.1        Protein of unknown function UPF0153
   738823..740106     +  427   88950648            MtheDRAFT_1228       ZP_01153222.1        Phenylacetate--CoA ligase
   740139..740522     +  127   88950649            MtheDRAFT_1229       ZP_01153223.1        Phosphoribosylaminoimidazole carboxylase
---------------------------------------
  ORGANISM  Pyrococcus horikoshii OT3    accession no is NP_877809.1 gi is 33359354
   cds                dir len   gi         gene     locus                pid                  product
   920818..921363     -  181   14590854            PH1014               NP_142926.1          3-octaprenyl-4-hydroxybenzoate carboxy-lyase
   921424..922242     +  272   14590855            PH1015               NP_142927.1          hypothetical protein
   922222..922569     -  115   14590856            PH1016               NP_142928.1          hypothetical protein
   922530..923651     -  373   14590857            PH1017               NP_142929.1          cofactor modifying protein
   923431..923739     +  102   14590858            PH1018               NP_142930.1          hypothetical protein
-->923652..923906     -  84    33359354            PH1017.1n            NP_877809.1          putative molybdopterin converting factor,
   923939..925762     -  607   14590859            PH1019               NP_142931.1          aldehyde:ferredoxin oxidoreductase
   925899..927215     -  438   14590860            PH1020               NP_142932.1          aspartyl-tRNA synthetase
   927281..928531     -  416   14590861            PH1022               NP_142933.1          sugar-phosphate nucleotydyl transferase
   928754..928918     +  54    33359355            PH1022.2n            NP_877810.1          hypothetical protein
   928912..930708     -  598   14590862            PH1023               NP_142934.1          hypothetical protein
---------------------------------------
  ORGANISM  Thermococcus kodakarensis KOD1       accession no is BAD85254.1 gi is 57159324
   cds                dir len   gi         gene     locus                pid                  product
   933073..933375     +  100   57159319            TK1060               BAD85249.1           hypothetical protein, conserved
   933541..933849     +  102   57159320            TK1061               BAD85250.1           hypothetical protein, conserved
   933864..934337     -  157   57159321            TK1062               BAD85251.1           rubrerythrin-related protein
   934512..935324     +  270   57159322            TK1063               BAD85252.1           small-conductance mechanosensitive channel
   935458..936609     -  383   57159323            TK1064               BAD85253.1           predicted metallocofactor modifying protein,
-->936611..936859     -  82    57159324            TK1065               BAD85254.1           molybdopterin converting factor, subunit 1
   936932..938749     -  605   57159325            TK1066               BAD85255.1           tungsten-containing aldehyde:ferredoxin
   938899..940137     +  412   57159326            TK1067               BAD85256.1           hypothetical protein, conserved, containing
   940211..940414     +  67    57159327            TK1068               BAD85257.1           hypothetical protein, conserved
   940411..940755     +  114   57159328            TK1069               BAD85258.1           hypothetical protein, conserved
   940748..940882     +  44    57159329            TK1070               BAD85259.1           hypothetical protein
---------------------------------------
  ORGANISM  uncultured crenarchaeote     accession no is AJ627421.1 gi is 42557747
   cds                dir len   gi         gene     locus                pid                  product
   18029..18628       -  199   42557742                                 CAF28716.1           hypothetical protein
   18737..19096       +  119   42557743                                 CAF28717.1           hypothetical protein
   19211..19624       +  137   42557744                                 CAF28718.1           hypothetical protein
   19627..21171       +  514   42557745                                 CAF28719.1           putative queuine/archaeosine tRNA
   21241..21648       +  135   42557746                                 CAF28720.1           hypothetical protein
-->21689..23035       -  448   42557747                                 CAF28721.1           putative molybdopterin biosynthesis protein
   23053..24279       -  408   42557748                                 CAF28722.1           putative threonine synthase
   24456..24803       -  115   42557749                                 CAF28723.1           putative phosphoribosyl-AMP cyclohydrolase
   25335..27137       -  600   42557750                                 CAF28724.1           putative oligoendopeptidase F
   27435..28505       -  356   42557751                                 CAF28725.1           putative deoxyhypusine synthase
   28608..29204       -  198   42557752                                 CAF28726.1           hypothetical protein
---------------------------------------
  ORGANISM  Natronomonas pharaonis DSM 2160      accession no is YP_326428.1 gi is 76801420
   cds                dir len   gi         gene     locus                pid                  product
   748067..749437     +  456   76801415   purA     NP1530A              YP_326423.1          adenylosuccinate synthase
   749594..750253     +  219   76801416            NP1532A              YP_326424.1          pterin operon protein (predicted DNA binding
   750260..750694     +  144   76801417            NP1534A              YP_326425.1          pterin operon protein
   750695..751201     +  168   76801418   mobB     NP1536A              YP_326426.1          probable molybdopterin-guanine dinucleotide
   751198..751827     -  209   76801419            NP1538A              YP_326427.1          pterin cluster protein (predicted DNA binding
-->751921..752358     -  145   76801420            NP1540A              YP_326428.1          pterin cluster protein
   752415..753617     -  400   76801421   cmo_2    NP1542A              YP_326429.1          molybdopterin-based tungsten cofactor
   753614..753922     -  102   76801422            NP1544A              YP_326430.1          conserved pterin operon protein
   753986..755101     -  371   76801423   cmo_1    NP1546A              YP_326431.1          molybdopterin-based tungsten cofactor
   755190..755471     +  93    76801424            NP1548A              YP_326432.1          hypothetical protein
   755510..755740     -  76    76801425            NP1550A              YP_326433.1          hypothetical protein
---------------------------------------
  ORGANISM  Haloarcula marismortui ATCC 43049    accession no is YP_136620.1 gi is 55378770
   cds                dir len   gi         gene     locus                pid                  product
   1841946..1842737   -  263   55378765            rrnAC2052            YP_136615.1          transcription regulator
   1842827..1843228   +  133   55378766            rrnAC2053            YP_136616.1          hypothetical protein
   1843244..1844023   -  259   55378767            rrnAC2054            YP_136617.1          hypothetical protein
   1844113..1845075   -  320   55378768   serA3    rrnAC2056            YP_136618.1          D-3-phosphoglycerate dehydrogenase
   1845240..1845512   +  90    55378769   moaD     rrnAC2057            YP_136619.1          molybdopterin converting factor subunit 1
-->1845538..1845939   -  133   55378770            rrnAC2058            YP_136620.1          hypothetical protein
   1846031..1846399   +  122   55378771            rrnAC2059            YP_136621.1          hypothetical protein
   1846504..1846758   +  84    55378772            rrnAC2060            YP_136622.1          hypothetical protein
   1846769..1847137   +  122   55378773            rrnAC2061            YP_136623.1          hypothetical protein
   1847121..1847486   -  121   55378774            rrnAC2062            YP_136624.1          hypothetical protein
   1847523..1849469   -  648   55378775   aor1     rrnAC2064            YP_136625.1          aldehyde ferredoxin oxidoreductase
---------------------------------------
  ORGANISM  Methanococcoides burtonii DSM 6242   accession no is NZ_AADH02000002.1 gi is 68210071
   cds                dir len   gi         gene     locus                pid                  product
   35494..36303       -  269   68210066            MburDRAFT_2201       ZP_00561932.1        similar to secreted protein
   37053..37973       -  306   68210067            MburDRAFT_2202       ZP_00561933.1        LPPG:Fo 2-phospho-L-lactate transferase
   38039..39241       +  400   68210068            MburDRAFT_2203       ZP_00561934.1        Metal-dependent phosphohydrolase, HD subdomain
   39238..39600       +  120   68210069            MburDRAFT_2204       ZP_00561935.1        conserved protein
   39603..40154       +  183   68210070            MburDRAFT_2205       ZP_00561936.1        Phenylacrylic acid decarboxylase
-->40617..40955       +  112   68210071            MburDRAFT_2206       ZP_00561937.1        MoaD, archaeal
   40988..41365       +  125   68210072            MburDRAFT_2207       ZP_00561938.1        Camphor resistance CrcB protein
   41379..41543       +  54    68210347            MburDRAFT_2482       ZP_00562213.1        camphor resistance CrcB protein
   41754..42062       +  102   68210073            MburDRAFT_2208       ZP_00561939.1        Protein of unknown function DUF190
   42065..42325       -  86    68210074            MburDRAFT_2209       ZP_00561940.1        Protein of unknown function UPF0044
   42416..43117       -  233   68210075            MburDRAFT_2210       ZP_00561941.1        Vng0609c
---------------------------------------
  ORGANISM  Halobacterium sp. NRC-1      accession no is AE005085.1 gi is 10581293
   cds                dir len   gi         gene     locus                pid                  product
   277..1578          -  433   10581288            VNG1842H             AAG20048.1           Vng1842h
   1654..2310         +  218   10581289            VNG1843C             AAG20049.1           Vng1843c
   2346..3629         +  427   10581290   ansA     VNG1844G             AAG20050.1           L-asparaginase
   3617..4558         +  313   10581291            VNG1845C             AAG20051.1           Vng1845c
   4559..5290         -  243   10581292            VNG1846C             AAG20052.1           Vng1846c
-->5290..5592         -  100   10581293            VNG1848H             AAG20053.1           Vng1848h
   5593..6828         +  411   10581294   pchA     VNG1847G             AAG20054.1           potassium channel homolog
   6838..8019         -  393   10581295            VNG1849H             AAG20055.1           Vng1849h
   8364..9140         -  258   10581296   udp1     VNG1850G             AAG20056.1           uridine phosphorylase
   9189..10076        -  295   10581297   suk      VNG1851G             AAG20057.1           sugar kinase
   10101..10934       -  277   10581298            VNG1852H             AAG20058.1           Vng1852h
---------------------------------------
  ORGANISM  Methanosarcina acetivorans C2A       accession no is AAM07434.1 gi is 19918186
   cds                dir len   gi         gene     locus                pid                  product
   5001782..5003095   -  437   19918180            MA_4081              AAM07429.1           conserved hypothetical protein
   5003360..5006089   +  909   19918181   pacL     MA_4082              AAM07430.1           cation-transporting ATPase
   5006170..5007351   -  393   19918183            MA_4083              AAM07431.1           NaH antiporter protein
   5007383..5008042   -  219   19918184            MA_4084              AAM07432.1           hypothetical protein (multi-domain)
   5008379..5010058   -  559   19918185            MA_4085              AAM07433.1           conserved hypothetical protein
-->5010139..5010432   +  97    19918186   moaD     MA_4086              AAM07434.1           molybdopterin converting factor, subunit 1
   5010454..5011647   +  397   19918187            MA_4087              AAM07435.1           molybdenum cofactor biosynthesis protein MoeA2
   5011792..5012181   +  129   19918188   crcB     MA_4088              AAM07436.1           CrcB family protein
   5012218..5012553   +  111   19918189   crcB     MA_4089              AAM07437.1           CrcB family protein
   5012735..5013007   +  90    19918190            MA_4090              AAM07438.1           predicted protein
   5013762..5014478   +  238   19918191            MA_4091              AAM07439.1           hypothetical protein (multi-domain)
---------------------------------------
  ORGANISM  Methanosarcina mazei Go1     accession no is NP_632855.1 gi is 21226933
   cds                dir len   gi         gene     locus                pid                  product
   976865..977551     +  228   21226928            MM0826               NP_632850.1          hypothetical protein
   977671..978390     -  239   21226929            MM0827               NP_632851.1          hypothetical protein
   979245..979613     -  122   21226930            MM0828               NP_632852.1          hypothetical protein
   979617..979997     -  126   21226931            MM0829               NP_632853.1          hypothetical protein
   980136..981326     -  396   21226932            MM0830               NP_632854.1          Molybdopterin biosynthesis MoeA protein
-->981348..981641     -  97    21226933            MM0831               NP_632855.1          Molybdopterin converting factor small subunit
   981722..983308     +  528   21226934            MM0832               NP_632856.1          hypothetical protein
   983548..984144     +  198   21226935            MM0833               NP_632857.1          hypothetical protein
   984394..985539     +  381   21226936            MM0834               NP_632858.1          Na(+)/H(+) antiporter
   985716..988448     -  910   21226937            MM0835               NP_632859.1          Cation-transporting ATPase
   988734..990065     +  443   21226938            MM0836               NP_632860.1          hypothetical protein
---------------------------------------
  ORGANISM  Methanosarcina barkeri str. fusaro   accession no is AAZ69478.1 gi is 72395205
   cds                dir len   gi         gene     locus                pid                  product
   588372..589112     -  246   72395200            Mbar_A0491           AAZ69473.1           conserved hypothetical protein
   589441..589620     +  59    72395201            Mbar_A0492           AAZ69474.1           conserved hypothetical protein
   589825..590193     -  122   72395202            Mbar_A0493           AAZ69475.1           protein crcB homolog 1
   590198..590578     -  126   72395203            Mbar_A0494           AAZ69476.1           CrcB family protein
   591021..592214     -  397   72395204            Mbar_A0495           AAZ69477.1           molybdenum cofactor biosynthesis protein MoeA2
-->592235..592528     -  97    72395205            Mbar_A0496           AAZ69478.1           molybdopterin converting factor small subunit
   592608..594260     +  550   72395206            Mbar_A0497           AAZ69479.1           conserved hypothetical protein
   594471..595094     +  207   72395207            Mbar_A0498           AAZ69480.1           conserved hypothetical protein
   595349..596518     +  389   72395208            Mbar_A0499           AAZ69481.1           NaH antiporter protein
   596709..599453     -  914   72395209            Mbar_A0500           AAZ69482.1           cation-transporting ATPase
   599740..601053     +  437   72395210            Mbar_A0501           AAZ69483.1           conserved hypothetical protein
---------------------------------------
  ORGANISM  Natronomonas pharaonis DSM 2160      accession no is YP_327616.1 gi is 76802608
   cds                dir len   gi         gene     locus                pid                  product
   1918581..1919090   -  169   76802603            NP3936A              YP_327611.1          hypothetical protein
   1919208..1919552   +  114   76802604            NP3938A              YP_327612.1          hypothetical protein
   1919604..1920587   +  327   76802605   purM     NP3940A              YP_327613.1          phosphoribosylaminoimidazole synthetase
   1920720..1921304   -  194   76802606            NP3942A              YP_327614.1          hypothetical protein
   1921301..1922335   -  344   76802607            NP3944A              YP_327615.1          hydrolase (probable hydroxyacylglutathione
-->1922332..1922625   -  97    76802608   moaD_3   NP3946A              YP_327616.1          homolog to molybdopterin converting factor,
   1922706..1923317   -  203   76802609   glo_1    NP3948A              YP_327617.1          lyase/ dioxygenase 1 (probable
   1923586..1924173   -  195   76802610            NP3952A              YP_327618.1          hypothetical protein
   1924255..1924752   -  165   76802611   hcp_1    NP3954A              YP_327619.1          halocyanin 1
   1924900..1926435   +  511   76802612            NP3956A              YP_327620.1          hypothetical protein
   1926489..1927991   +  500   76802613   deoA     NP3958A              YP_327621.1          putative thymidine phosphorylase
---------------------------------------
  ORGANISM  Pyrobaculum aerophilum str. IM2      accession no is AE009927.1 gi is 18161603
   cds                dir len   gi         gene     locus                pid                  product
   73..465            +  130   18161598   PAE3367                       AAL64869.1           hypothetical protein
   485..1720          +  411   18161599   PAE3368                       AAL64870.1           conserved hypothetical protein
   1749..3266         +  505   18161600   PAE3369                       AAL64871.1           GMP synthetase (glutamine-hydrolysing)
   3457..4848         +  463   18161601   PAE3371                       AAL64872.1           gamma-glutamyltransferase
   5444..5644         -  66    18161602   PAE3373                       AAL64873.1           paREP2b
-->5768..6052         +  94    18161603   PAE3375                       AAL64874.1           conserved hypothetical protein
   6049..6993         -  314   18161604   PAE3376                       AAL64875.1           beta-lactamase-like protein
   7257..7976         +  239   18161605   PAE3378                       AAL64876.1           sugar fermentation stimulation protein,
   7956..8807         -  283   18161606   PAE3380                       AAL64877.1           conserved hypothetical protein
   8846..9430         +  194   18161607   PAE3381                       AAL64878.1           beta-lactamase-like protein
   9773..12520        +  915   18161608   PAE3383                       AAL64879.1           pyruvate, phosphate dikinase
---------------------------------------
  ORGANISM  Methanosarcina acetivorans C2A       accession no is AAM05120.1 gi is 19915596
   cds                dir len   gi         gene     locus                pid                  product
   2046993..2047688   +  231   19915590            MA_1708              AAM05115.1           uncharacterized Fe-S protein
   2048705..2049928   +  407   19915591            MA_1709              AAM05116.1           hypothetical protein
   2050465..2051430   +  321   19915593   mch      MA_1710              AAM05117.1           methenyltetrahydromethanopterin cyclohydrolase
   2051782..2052249   +  155   19915594            MA_1711              AAM05118.1           conserved hypothetical protein
   2052881..2054050   +  389   19915595            MA_1712              AAM05119.1           aspartate aminotransferase
-->2054261..2054545   -  94    19915596            MA_1713              AAM05120.1           predicted protein
   2054615..2056561   -  648   19915597            MA_1714              AAM05121.1           aldehyde ferredoxin oxidoreductase
   2056707..2057468   -  253   19915598            MA_1715              AAM05122.1           conserved hypothetical protein
   2057993..2058400   -  135   19915599            MA_1716              AAM05123.1           conserved hypothetical protein
   2058677..2059861   +  394   19915600   slg      MA_1717              AAM05124.1           cell surface protein
   2060242..2061168   -  308   19915602            MA_1718              AAM05125.1           conserved hypothetical protein
---------------------------------------
  ORGANISM  Methanosarcina mazei Go1     accession no is NP_634668.1 gi is 21228746
   cds                dir len   gi         gene     locus                pid                  product
   3150428..3151348   -  306   21228741            MM2639               NP_634663.1          hypothetical protein
   3151632..3152702   +  356   21228742            MM2640               NP_634664.1          Magnesium and cobalt transport protein CorA
   3153400..3154143   +  247   21228743            MM2641               NP_634665.1          hypothetical protein
   3154140..3155084   +  314   21228744            MM2642               NP_634666.1          hypothetical protein
   3155266..3155847   -  193   21228745            MM2643               NP_634667.1          hypothetical protein
-->3156001..3156285   -  94    21228746            MM2644               NP_634668.1          putative molybdopterin converting factor
   3156303..3158129   -  608   21228747            MM2645               NP_634669.1          aldehyde ferredoxin oxidoreductase
   3158469..3161120   -  883   21228748            MM2646               NP_634670.1          sensory transduction protein kinase
   3161691..3162995   -  434   21228749            MM2647               NP_634671.1          Transposase
   3163288..3164058   -  256   21228750            MM2648               NP_634672.1          hypothetical protein
   3164334..3165503   -  389   21228751            MM2649               NP_634673.1          aspartate aminotransferase
---------------------------------------
  ORGANISM  Pyrobaculum aerophilum str. IM2      accession no is AE009859.1 gi is 18160633
   cds                dir len   gi         gene     locus                pid                  product
   112..717           -  201   18160629   PAE2131                       AAL63968.1           fumarate hydratase class I beta subunit
   823..1689          +  288   18160630   PAE2132                       AAL63969.1           fumarate hydratase class I alpha subunit
   1681..1842         -  53    18160631   PAE2133                       AAL63970.1           conserved hypothetical protein
   1872..2294         +  140   18160632   PAE2134                       AAL63971.1           hypothetical protein
-->2291..2572         -  93    18160633   PAE2135                       AAL63972.1           conserved hypothetical protein
   2718..2858         +  46    18160634   PAE2136                       AAL63973.1           paREP2a
   3029..3409         -  126   18160635   PAE2137                       AAL63974.1           hypothetical protein
   3513..4406         +  297   18160636   PAE2138                       AAL63975.1           conserved hypothetical protein
   4574..4882         -  102   18160637   PAE2138a                      AAL63976.1           hypothetical protein
   5516..5692         +  58    18160638   PAE2140                       AAL63977.1           hypothetical protein
---------------------------------------
  ORGANISM  Natronomonas pharaonis DSM 2160      accession no is YP_326901.1 gi is 76801893
   cds                dir len   gi         gene     locus                pid                  product
   1207271..1208068   +  265   76801888            NP2490A              YP_326896.1          hypothetical protein
   1208711..1209955   +  414   76801889   gatD     NP2492A              YP_326897.1          glutamyl-tRNA(Gln) amidotransferase subunit D
   1209955..1210854   +  299   76801890            NP2494A              YP_326898.1          protein N-acetyltransferase homolog
   1210840..1211214   -  124   76801891            NP2496A              YP_326899.1          hypothetical protein
   1211214..1211468   -  84    76801892            NP2498A              YP_326900.1          hypothetical protein
-->1211469..1211750   -  93    76801893   moaD_2   NP2500A              YP_326901.1          probable molybdopterin converting factor, small
   1211773..1212159   -  128   76801894            NP2502A              YP_326902.1          hypothetical protein
   1212198..1212512   -  104   76801895            NP2504A              YP_326903.1          hypothetical protein
   1212546..1214303   -  585   76801896   tgtA2    NP2506A              YP_326904.1          queuine/archaeosine tRNA-ribosyltransferase II
   1214300..1215862   -  520   76801897   tgtA1    NP2508A              YP_326905.1          queuine/archaeosine tRNA-ribosyltransferase I
   1215922..1216851   -  309   76801898            NP2510A              YP_326906.1          probable KaiC-like transcriptional regulator 2
---------------------------------------
  ORGANISM  Haloarcula marismortui ATCC 43049    accession no is YP_137824.1 gi is 55379974
   cds                dir len   gi         gene     locus                pid                  product
   3034827..3035552   +  241   55379969   udp1     rrnAC3434            YP_137819.1          uridine phosphorylase
   3035670..3037184   +  504   55379970            rrnAC3435            YP_137820.1          hypothetical protein
   3037181..3038395   -  404   55379971   pch2     rrnAC3436            YP_137821.1          potassium channel-like
   3038419..3039699   +  426   55379972            rrnAC3437            YP_137822.1          hypothetical protein
   3039696..3041516   +  606   55379973            rrnAC3438            YP_137823.1          hypothetical protein
-->3041639..3041917   +  92    55379974            rrnAC3439            YP_137824.1          hypothetical protein
   3041936..3043591   -  551   55379975            rrnAC3440            YP_137825.1          hypothetical protein
   3043643..3044536   -  297   55379976   hat5     rrnAC3441            YP_137826.1          GNAT family acetyltransferase
   3044630..3045877   -  415   55379977   asbA     rrnAC3442            YP_137827.1          glutamyl-tRNA(Gln) amidotransferase subunit D
   3046073..3046672   +  199   55379978            rrnAC3443            YP_137828.1          hypothetical protein
   3046746..3047255   +  169   55379979            rrnAC3445            YP_137829.1          hypothetical protein
---------------------------------------
  ORGANISM  Natronomonas pharaonis DSM 2160      accession no is YP_331233.1 gi is 76803138
   cds                dir len   gi         gene     locus                pid                  product
   2431281..2431955   +  224   76803133   purQ     NP5010A              YP_331228.1          phosphoribosylformylglycinamidine synthase ,
   2431956..2433032   -  358   76803134            NP5012A              YP_331229.1          hypothetical protein
   2433079..2433447   -  122   76803135            NP5014A              YP_331230.1          hypothetical protein
   2433568..2435508   +  646   76803136   aor_4    NP5016A              YP_331231.1          aldehyde ferredoxin oxidoreductase 4
   2435518..2436624   -  368   76803137            NP5018A              YP_331232.1          hypothetical protein
-->2436689..2436967   -  92    76803138   moaD_1   NP5020A              YP_331233.1          probable molybdopterin converting factor, small
   2437026..2437445   +  139   76803139            NP5022A              YP_331234.1          hypothetical protein
   2437448..2437867   -  139   76803140            NP5024A              YP_331235.1          probable stress response protein
   2437934..2439709   -  591   76803141            NP5026A              YP_331236.1          predicted ATPase
   2439761..2440876   -  371   76803142            NP5028A              YP_331237.1          hypothetical protein
   2441031..2442293   +  420   76803143            NP5030A              YP_331238.1          hypothetical protein
---------------------------------------
  ORGANISM  Methanospirillum hungatei JF-1       accession no is YP_503631.1 gi is 88603453
   cds                dir len   gi         gene     locus                pid                  product
   2471256..2472248   -  330   88603448            Mhun_2202            YP_503626.1          quinolinate synthetase complex, A subunit
   2472369..2473550   +  393   88603449            Mhun_2203            YP_503627.1          aminotransferase, class V
   2473553..2474317   +  254   88603450            Mhun_2204            YP_503628.1          formate dehydrogenase family accessory protein
   2474324..2475322   -  332   88603451            Mhun_2205            YP_503629.1          ABC transporter, substrate-binding protein,
   2475423..2475674   +  83    88603452            Mhun_2206            YP_503630.1          hypothetical protein
-->2475808..2476086   +  92    88603453            Mhun_2207            YP_503631.1          thiamineS
   2476087..2476485   +  132   88603454            Mhun_2208            YP_503632.1          molybdopterin biosynthesis MoaE
   2476469..2477641   +  390   88603455            Mhun_2209            YP_503633.1          aminotransferase, class V
   2477626..2477958   +  110   88603456            Mhun_2210            YP_503634.1          hypothetical protein
   2478032..2479015   -  327   88603457            Mhun_2211            YP_503635.1          serine O-acetyltransferase
   2479434..2480363   +  309   88603458            Mhun_2212            YP_503636.1          Protein of unknown function UPF0021
---------------------------------------
  ORGANISM  Archaeoglobus fulgidus DSM 4304      accession no is NP_068863.1 gi is 11497643
   cds                dir len   gi         gene     locus                pid                  product
   19287..21272       +  661   11497638   hbd-1    AF0017               NP_068858.1          3-hydroxyacyl-CoA dehydrogenase (hbd-1)
   21295..22470       +  391   11497639   acaB-1   AF0018               NP_068859.1          3-ketoacyl-CoA thiolase (acaB-1)
   22475..22957       +  160   11497640            AF0019               NP_068860.1          hypothetical protein
   22960..24222       +  420   11497641   caiB-1   AF0020               NP_068861.1          L-carnitine dehydratase (caiB-1)
   24261..25232       +  323   11497642            AF0021               NP_068862.1          signal-transducing histidine kinase
-->25221..25496       -  91    11497643            AF0022               NP_068863.1          hypothetical protein
   25503..27281       -  592   11497644   aor-1    AF0023               NP_068864.1          aldehyde ferredoxin oxidoreductase (aor-1)
   27284..28534       -  416   11497645            AF0024               NP_068865.1          alcohol dehydrogenase, iron-containing
   28539..29720       -  393   11497646   cynX     AF0025               NP_068866.1          cyanate transport protein (cynX)
   30082..30732       +  216   11497647            AF0026               NP_068867.1          hypothetical protein
   30788..31096       +  102   11497648            AF0027               NP_068868.1          hypothetical protein
---------------------------------------
  ORGANISM  Thermoplasma volcanium GSS1  accession no is BAB59950.1 gi is 14325024
   cds                dir len   gi         gene     locus                pid                  product
   803249..804319     +  356   14325019   TVG0804319                      BAB59945.1
   804316..805746     +  476   14325020   TVG0805746                      BAB59946.1           hypohtetical protein
   805714..806385     -  223   14325021   TVG0805714                      BAB59947.1           hypothetical protein
   806423..807466     -  347   14325022   TVG0806423                      BAB59948.1           cell division protein [FtsZ]
   807617..808276     +  219   14325023   TVG0808276                      BAB59949.1           hypothetical protein
-->808305..808580     +  91    14325024   TVG0808580                      BAB59950.1           molybdopterin converting factor subunit 1
   808604..810415     -  603   14325025   TVG0808604                      BAB59951.1           aldehyde-ferredoxin oxidoreductase [AOR]
   810537..811706     +  389   14325026   TVG0811706                      BAB59952.1           serine hydroxymethyltransferase
   811714..812781     +  355   14325027   TVG0812781                      BAB59953.1           inosine-5 -monophosphate dehydrogenase
   812825..814159     +  444   14325028   TVG0814159                      BAB59954.1           maturation protease
   814156..815451     +  431   14325029   TVG0815451                      BAB59955.1           maturation protease
---------------------------------------
  ORGANISM  Methanospirillum hungatei JF-1       accession no is YP_502003.1 gi is 88601825
   cds                dir len   gi         gene     locus                pid                  product
   597723..598817     -  364   88601820            Mhun_0519            YP_501998.1          molybdenum-pterin binding domain
   598825..599511     -  228   88601821            Mhun_0520            YP_501999.1          binding-protein-dependent transport systems
   599549..600472     -  307   88601822            Mhun_0521            YP_502000.1          ABC transporter tungsten-binding protein
   600824..601753     +  309   88601823            Mhun_0522            YP_502001.1          HhH-GPD
   601962..602369     -  135   88601824            Mhun_0523            YP_502002.1          molybdopterin biosynthesis MoaE
-->602372..602647     -  91    88601825            Mhun_0524            YP_502003.1          thiamineS
   602644..603390     -  248   88601826            Mhun_0525            YP_502004.1          UBA/THIF-type NAD/FAD binding fold
   603387..604028     -  213   88601827            Mhun_0526            YP_502005.1          TfuA-like, core
   603982..605202     -  406   88601828            Mhun_0527            YP_502006.1          protein of unknown function DUF181
   605333..605650     +  105   88601829            Mhun_0528            YP_502007.1          hypothetical protein
   605672..606943     -  423   88601830            Mhun_0529            YP_502008.1          hypothetical protein
---------------------------------------
  ORGANISM  Thermoplasma acidophilum     accession no is AL445065.1 gi is 10640172
   cds                dir len   gi         gene     locus                pid                  product
   275931..276647     +  238   10640167   Ta0890                        CAC12019.1           conserved hypothetical protein
   276650..277495     +  281   10640168   Ta0891                        CAC12020.1           endonuclease IV related protein
   277496..278653     +  385   10640169   Ta0892                        CAC12021.1           conserved hypothetical protein
   279319..280536     +  405   10640170   Ta0893                        CAC12022.1           hypothetical protein
   280736..281344     +  202   10640171   Ta0894                        CAC12023.1           conserved hypothetical protein
-->281644..281916     +  90    10640172   Ta0895                        CAC12024.1           conserved hypothetical protein
   281950..283584     -  544   10640173   Ta0896                        CAC12025.1           pyruvate kinase related protein
   284009..285094     +  361   10640174   Ta0897                        CAC12026.1           glucose 1-dehydrogenase
   285091..285921     +  276   10640175   Ta0898                        CAC12027.1           methylenetetrahydrofolate dehydrogenase (NADP+)
   286169..286894     +  241   10640176   Ta0899                        CAC12028.1           NAD(+) synthase related protein
   286901..288199     +  432   10640177   Ta0900                        CAC12029.1           Na+/H+ antiporter related protein
---------------------------------------
  ORGANISM  Thermoplasma volcanium GSS1  accession no is BAB59710.1 gi is 14324783
   cds                dir len   gi         gene     locus                pid                  product
   550702..551085     +  127   14324778   TVG0551085                      BAB59705.1           ribosomal protein small subunit S14
   551086..551901     +  271   14324779   TVG0551901                      BAB59706.1           DNA-directed RNA polymerase D
   552150..553325     +  391   14324780   TVG0553325                      BAB59707.1           molybdenum cofactor biosynthesis protein moeA
   553322..555211     +  629   14324781   TVG0555211                      BAB59708.1           molybdenum cofactor biosynthesis protein moeB
   555376..556167     -  263   14324782   TVG0555376                      BAB59709.1
-->556254..556526     +  90    14324783   TVG0556526                      BAB59710.1           molybdopterin converting factor subunit 1
   556601..557275     +  224   14324784   TVG0557275                      BAB59711.1           transcription initiation factor B [TFB]
   557379..558521     -  380   14324785   TVG0557379                      BAB59712.1           aspartate aminotransferase
   558589..559821     -  410   14324786   TVG0558589                      BAB59713.1           hypothetical protein
   559784..561151     -  455   14324787   TVG0559784                      BAB59714.1           DNA helicase
   561228..562373     +  381   14324788   TVG0562373                      BAB59715.1           hypothetical protein
---------------------------------------
  ORGANISM  Pyrobaculum aerophilum str. IM2      accession no is AE009852.1 gi is 18160535
   cds                dir len   gi         gene     locus                pid                  product
   144..2297          +  717   18160531   PAE2005                       AAL63877.1           P. aerophilum family 59 protein
   2298..2870         +  190   18160532   PAE2006                       AAL63878.1           P. aerophilum family 3 protein
   2908..3438         -  176   18160533   PAE2008                       AAL63879.1           deoxycytidine triphosphate deaminase
   3531..4037         +  168   18160534   PAE2010                       AAL63880.1           hypothetical protein
-->4034..4306         -  90    18160535   PAE2011                       AAL63881.1           conserved hypothetical protein
   4349..4795         +  148   18160536   PAE2012                       AAL63882.1           methylated-DNA--[protein]-cysteine
   4832..5059         +  75    18160537   PAE2013                       AAL63883.1           ferredoxin
   5256..5435         +  59    18160538   PAE2015                       AAL63884.1           conserved within P. aerophilum
   5467..6117         +  216   18160539   PAE2017                       AAL63885.1           phosphoserine phosphatase (serB)
   6199..7092         +  297   18160540   PAE2019                       AAL63886.1           conserved hypothetical protein
---------------------------------------
  ORGANISM  Haloarcula marismortui ATCC 43049    accession no is YP_136619.1 gi is 55378769
   cds                dir len   gi         gene     locus                pid                  product
   1840688..1841707   -  339   55378764   tfbF     rrnAC2051            YP_136614.1          transcription initiation factor IIB
   1841946..1842737   -  263   55378765            rrnAC2052            YP_136615.1          transcription regulator
   1842827..1843228   +  133   55378766            rrnAC2053            YP_136616.1          hypothetical protein
   1843244..1844023   -  259   55378767            rrnAC2054            YP_136617.1          hypothetical protein
   1844113..1845075   -  320   55378768   serA3    rrnAC2056            YP_136618.1          D-3-phosphoglycerate dehydrogenase
-->1845240..1845512   +  90    55378769   moaD     rrnAC2057            YP_136619.1          molybdopterin converting factor subunit 1
   1845538..1845939   -  133   55378770            rrnAC2058            YP_136620.1          hypothetical protein
   1846031..1846399   +  122   55378771            rrnAC2059            YP_136621.1          hypothetical protein
   1846504..1846758   +  84    55378772            rrnAC2060            YP_136622.1          hypothetical protein
   1846769..1847137   +  122   55378773            rrnAC2061            YP_136623.1          hypothetical protein
   1847121..1847486   -  121   55378774            rrnAC2062            YP_136624.1          hypothetical protein
---------------------------------------
  ORGANISM  Archaeoglobus fulgidus DSM 4304      accession no is NP_070930.1 gi is 11499688
   cds                dir len   gi         gene     locus                pid                  product
   1891101..1892828   +  575   11499683   ilvB-4   AF2100               NP_070925.1          acetolactate synthase, large subunit (ilvB-4)
   1892825..1894033   +  402   11499684            AF2101               NP_070926.1          alcohol dehydrogenase, zinc-dependent
   1894023..1894676   -  217   11499685            AF2102               NP_070927.1          hypothetical protein
   1894712..1895155   -  147   11499686            AF2103               NP_070928.1          hypothetical protein
   1895165..1896304   -  379   11499687            AF2104               NP_070929.1          hypothetical protein
-->1896301..1896570   -  89    11499688            AF2105               NP_070930.1          hypothetical protein
   1896610..1897311   +  233   11499689            AF2106               NP_070931.1          hypothetical protein
   1897308..1898024   +  238   11499690   ribB     AF2107               NP_070932.1          3,4-dihydroxy-2-butanone 4-phosphate synthase
   1898082..1898924   +  280   11499691            AF2108               NP_070933.1          hypothetical protein
   1898905..1900731   -  608   11499692            AF2109               NP_070934.1          signal-transducing histidine kinase
   1900730..1901455   +  241   11499693            AF2110               NP_070935.1          hypothetical protein
---------------------------------------
  ORGANISM  Archaeoglobus fulgidus DSM 4304      accession no is NP_069388.1 gi is 11498162
   cds                dir len   gi         gene     locus                pid                  product
   498121..499554     +  477   11498157            AF0547               NP_069383.1          reductase, iron-sulfur binding subunit
   499614..501473     +  619   11498158   thrS     AF0548               NP_069384.1          threonyl-tRNA synthetase
   501479..501802     +  107   11498159            AF0549               NP_069385.1          hypothetical protein
   501786..503054     -  422   11498160   trzA-1   AF0550               NP_069386.1          N-ethylammeline chlorohydrolase (trzA-1)
   503163..504377     +  404   11498161   thrC-1   AF0551               NP_069387.1          threonine synthase
-->504382..504648     +  88    11498162            AF0552               NP_069388.1          hypothetical protein
   504649..505452     +  267   11498163   thiF     AF0553               NP_069389.1          thiamine biosynthesis protein (thiF)
   505412..505678     +  88    11498164            AF0554               NP_069390.1          hypothetical protein
   505809..506192     +  127   11498165            AF0555               NP_069391.1          hypothetical protein
   506213..506434     +  73    11498166            AF0556               NP_069392.1          hypothetical protein
   506436..507578     +  380   11498167            AF0557               NP_069393.1          flavoprotein reductase
---------------------------------------
  ORGANISM  Pyrobaculum aerophilum str. IM2      accession no is AE009884.1 gi is 18160982
   cds                dir len   gi         gene     locus                pid                  product
   5180..5434         -  84    18160977   PAE2574                       AAL64291.1           hypothetical protein
   5431..6234         -  267   18160978   PAE2575                       AAL64292.1           phosphoadenosine phosphosulfate reductase (PAPS
   6236..6847         -  203   18160979   PAE2576                       AAL64293.1           hypothetical protein
   6844..8448         -  534   18160980   PAE2577                       AAL64294.1           ferredoxin-nitrite reductase
   8558..9424         +  288   18160981   PAE2578                       AAL64295.1           conserved hypothetical protein
-->9521..9787         +  88    18160982   PAE2580                       AAL64296.1           conserved hypothetical protein
   9778..10473        +  231   18160983   PAE2582                       AAL64297.1           thiosulfate sulfurtransferase
---------------------------------------
  ORGANISM  Thermococcus kodakarensis KOD1       accession no is BAD86307.1 gi is 57160377
   cds                dir len   gi         gene     locus                pid                  product
   1896250..1897170   +  306   57160372            TK2113               BAD86302.1           hypothetical protein, conserved
   1897167..1898099   -  310   57160373            TK2114               BAD86303.1           hypothetical protein, conserved
   1898194..1898619   -  141   57160374            TK2115               BAD86304.1           molybdopterin converting factor, subunit 2
   1898673..1899542   +  289   57160375            TK2116               BAD86305.1           hypothetical protein
   1899535..1900233   -  232   57160376            TK2117               BAD86306.1           molybdenum cofactor biosynthesis protein MoeB
-->1900235..1900501   -  88    57160377            TK2118               BAD86307.1           molybdopterin converting factor, subunit 1
   1900569..1901084   -  171   57160378            TK2119               BAD86308.1           hypothetical membrane protein, conserved, DUF46
   1901147..1902070   +  307   57160379            TK2120               BAD86309.1           hypothetical protein, conserved, containing
   1902110..1902637   +  175   57160380            TK2121               BAD86310.1           hypothetical protein, conserved
   1902647..1903798   +  383   57160381            TK2122               BAD86311.1           tRNA/rRNA cytosine-C5-methylase, NOL1/NOP2/Sun
   1903801..1904826   +  341   57160382            TK2123               BAD86312.1           hypothetical membrane protien, conserved,
---------------------------------------
  ORGANISM  Archaeoglobus fulgidus DSM 4304      accession no is NP_070453.1 gi is 11499216
   cds                dir len   gi         gene     locus                pid                  product
   1453093..1454052   -  319   11499211            AF1619               NP_070448.1          hypothetical protein
   1454159..1456504   -  781   11499212            AF1620               NP_070449.1          signal-transducing histidine kinase, putative
   1456261..1457247   -  328   11499213            AF1621               NP_070450.1          hypothetical protein
   1457326..1457817   +  163   11499214   lrp      AF1622               NP_070451.1          leucine responsive regulatory protein (lrp)
   1457783..1458955   +  390   11499215   aspB-3   AF1623               NP_070452.1          aspartate aminotransferase (aspB-3)
-->1458942..1459202   +  86    11499216   moaD     AF1624               NP_070453.1          molybdopterin converting factor, subunit 1
   1459275..1461020   +  581   11499217            AF1625               NP_070454.1          hypothetical protein
   1461368..1461730   +  120   11499218            AF1626               NP_070455.1          hypothetical protein
   1461744..1461959   +  71    11499219            AF1627               NP_070456.1          repressor protein
   1462137..1463171   +  344   11499220            AF1628               NP_070457.1          transposase, putative
   1463218..1463961   +  247   11499221            AF1629               NP_070458.1          hypothetical protein
---------------------------------------
  ORGANISM  Thermoplasma acidophilum     accession no is AL445066.1 gi is 10640334
   cds                dir len   gi         gene     locus                pid                  product
   85186..85746       -  186   10640329   Ta1014                        CAC12143.1           probable GTP cyclohydrolase II
   85825..86961       -  378   10640330   Ta1015                        CAC12144.1           conserved hypothetical protein
   87146..88507       +  453   10640331   Ta1016                        CAC12145.1           DNA repair protein RAD25 related protein
   88476..89699       +  407   10640332   Ta1017                        CAC12146.1           conserved hypothetical protein
   89846..90994       +  382   10640333   Ta1018                        CAC12147.1           serine-glyoxylate aminotransferase related
-->90991..91248       +  85    10640334   Ta1019                        CAC12148.1           MoaD (involved in molybdopterin synthesis)
   91582..92268       +  228   10640335   Ta1020                        CAC12149.1           transcription initiation factor IIB related
   92480..93076       +  198   10640336   Ta1021                        CAC12150.1           hypothetical protein
   93106..94986       -  626   10640337   Ta1022                        CAC12151.1           molybdopterin biosynthesis protein (moeA-1)
   94983..96182       -  399   10640338   Ta1023                        CAC12152.1           molybdopterin biosynthesis protein (moeA-2)
   96432..98183       -  583   10640339   Ta1024                        CAC12153.1           multidrug resistance protein related protein
---------------------------------------
  ORGANISM  Sulfolobus acidocaldarius DSM 639    accession no is AAY80053.1 gi is 68567124
   cds                dir len   gi         gene     locus                pid                  product
   526525..526800     -  91    68567119            Saci_0664            AAY80048.1           conserved Archaeal protein
   526865..527452     +  195   68567120            Saci_0665            AAY80049.1           conserved protein
   527522..529183     +  553   68567121   thsB     Saci_0666            AAY80050.1           thermosome beta subunit
   529180..530820     -  546   68567122            Saci_0667            AAY80051.1           conserved Archaeal protein
   530841..531239     -  132   68567123            Saci_0668            AAY80052.1           conserved protein
-->531229..531483     -  84    68567124            Saci_0669            AAY80053.1           conserved Archaeal protein
   531486..531734     -  82    68567125            Saci_0670            AAY80054.1           30S ribosomal protein S17E
   531780..532937     -  385   68567126            Saci_0671            AAY80055.1           conserved Archaeal protein
   532970..534784     -  604   68567127            Saci_0672            AAY80056.1           RNase L inhibitor-like protein
   534834..534938     -  34    68567128            Saci_0673            AAY80057.1           conserved protein
   535612..536928     -  438   68567129            Saci_0675            AAY80058.1           conserved membrane protein
---------------------------------------
  ORGANISM  Ferroplasma acidarmanus Fer1         accession no is AABC04000004.1 gi is 68140833
   cds                dir len   gi         gene     locus                pid                  product
   113233..114312     -  359   68140837            FaciDRAFT_1242       EAM94132.1           Phosphoribosylaminoimidazole carboxylase, ATPase
   114309..114776     -  155   68140836            FaciDRAFT_1241       EAM94131.1           1-(5-Phosphoribosyl)-5-amino-4-imidazole-
   114825..116468     -  547   68140835            FaciDRAFT_1240       EAM94130.1           NADH:flavin oxidoreductase/NADH oxidase
   116536..117102     -  188   68140834            FaciDRAFT_1239       EAM94129.1           conserved hypothetical protein
   117232..118683     +  483   68140822            FaciDRAFT_1227       EAM94117.1           Gamma-glutamyltransferase
-->118699..118935     -  78    68140833            FaciDRAFT_1238       EAM94128.1           MoaD, archaeal
   118939..119886     -  315   68140832            FaciDRAFT_1237       EAM94127.1           MoaA; Radical SAM:Molybdenum cofactor synthesis C
   119964..120686     -  240   68140831            FaciDRAFT_1236       EAM94126.1           putative ABC-2 type transport system permease
   120676..121515     -  279   68140830            FaciDRAFT_1235       EAM94125.1           ABC transporter
   121621..122430     -  269   68140829            FaciDRAFT_1234       EAM94124.1           Survival protein SurE
   122722..123027     +  101   68140823            FaciDRAFT_1228       EAM94118.1           unknown product
---------------------------------------
  ORGANISM  Picrophilus torridus DSM 9790        accession no is AAT43641.1 gi is 48430776
   cds                dir len   gi         gene     locus                pid                  product
   1064376..1065221   -  281   48430771            PTO1051              AAT43636.1           fumarylacetoacetate hydrolase family protein
   1065218..1065562   -  114   48430772            PTO1052              AAT43637.1           transcriptional regulator
   1065946..1067022   -  358   48430773            PTO1053              AAT43638.1           nucleotide pyrophosphatase/phosphodiesterase I
   1067096..1069000   +  634   48430774            PTO1054              AAT43639.1           zinc metalloprotease
   1069110..1069877   -  255   48430775            PTO1055              AAT43640.1           transcriptional regulatory protein
-->1069907..1070134   -  75    48430776            PTO1056              AAT43641.1           molybdopterin (MPT) converting factor, subunit
   1070138..1071070   -  310   48430777            PTO1057              AAT43642.1           putative molybdopterin cofactor synthesis
   1071170..1072513   +  447   48430778            PTO1058              AAT43643.1           sugar transporter
   1072474..1072887   -  137   48430779            PTO1059              AAT43644.1           molybdopterin (MPT) converting factor, subunit
   1072916..1073392   -  158   48430780            PTO1060              AAT43645.1           molybdenum cofactor biosynthesis protein B
   1073389..1073814   -  141   48430781            PTO1061              AAT43646.1           molybdenum cofactor biosynthesis protein C
---------------------------------------
  ORGANISM  Pyrococcus furiosus DSM 3638         accession no is X79777.1 gi is 736275
   cds                dir len   gi         gene     locus                pid                  product
   <1..955            -  318   736273                                   CAA56169.1
   1150..2967         +  605   736274     AOR                           CAA56170.1           aldehyde:ferredoxin oxidoreductase
-->3016..3225         +  69    736275                                   CAA56171.1
   3330..4403         +  357   736276     cmo                           CAA56172.1           cofactor modifying protein
   4444..>4951        +  169   736277     ado-hcy                       CAA56173.1           S-adenosyl-L-homocysteine hydrolase
---------------------------------------
  ORGANISM  Pyrococcus abyssi GE5        accession no is NP_877624.1 gi is 33356700
   cds                dir len   gi         gene     locus                pid                  product
   536000..536245     -  81    33356699            PAB0385.1n           NP_877623.1          hypothetical protein
   536242..537024     -  260   14520779   minD-1   PAB1983              NP_126254.1          cell division inhibitor
   537163..537555     +  130   14520780            PAB0387              NP_126255.1          hypothetical protein
   537552..539075     -  507   14520781   deoA     PAB1982              NP_126256.1          putative thymidine phosphorylase
   539106..539741     -  211   14520782            PAB1981              NP_126257.1          dolichyl-phosphate mannose synthase related
-->539780..540016     +  78    33356700            PAB1981.1n           NP_877624.1          hypothetical protein
   540019..540543     +  174   14520783            PAB0389              NP_126258.1          hypothetical protein
   540498..541778     +  426   14520784   napA-2   PAB0390              NP_126259.1          Na+/H+ antiporter
   542065..543327     +  420   14520785   gdh      PAB0391              NP_126260.1          Glutamate dehydrogenase (NAD(P)+)
   543435..543860     +  141   14520786            PAB0392              NP_126261.1          transcriptional regulatory protein, AsnC f
   543976..544497     +  173   14520787   fbp      PAB0393              NP_126262.1          ferripyochelin binding protein
---------------------------------------
  ORGANISM  Pyrococcus furiosus DSM 3638         accession no is AE010260.1 gi is 18893753
   cds                dir len   gi         gene     locus                pid                  product
   6576..7097         -  173   18893748   PF1600                        AAL81724.1           ferripyochelin binding protein
   7223..7648         -  141   18893749   PF1601                        AAL81725.1           transcriptional regulatory protein, asnC family
   7739..9001         -  420   18893750   PF1602                        AAL81726.1           glutamate dehydrogenase
   9283..10566        -  427   18893751   PF1603                        AAL81727.1           hypothetical na antiporter
   10566..11045       -  159   18893752   PF1604                        AAL81728.1           hypothetical protein
-->11036..11275       -  79    18893753   PF1605                        AAL81729.1           hypothetical protein
   11324..11971       +  215   18893754   PF1606                        AAL81730.1           dolichol-phosphate mannose synthase
   11989..13500       +  503   18893755   PF1607                        AAL81731.1           thymidine phosphorylase
   13503..13907       -  134   18893756   PF1608                        AAL81732.1           hypothetical protein
---------------------------------------
  ORGANISM  Pyrococcus horikoshii OT3    accession no is NP_877858.1 gi is 33359416
   cds                dir len   gi         gene     locus                pid                  product
   1411063..1411584   -  173   14591369            PH1591               NP_143447.1          ferripyochelin binding protein
   1411724..1412149   -  141   14591370            PH1592               NP_143448.1          transcriptional regulator
   1412255..1413523   -  422   14591371            PH1593               NP_143449.1          glutamate dehydrogenase
   1413799..1415076   -  425   14591372            PH1594               NP_143450.1          Na(+)/H(+) antiporter
   1415046..1415564   -  172   14591373            PH1595               NP_143451.1          hypothetical protein
-->1415561..1415788   -  75    33359416            PH1595.1n            NP_877858.1          hypothetical protein
   1415837..1416484   +  215   14591374            PH1596               NP_143452.1          hypothetical protein
   1416497..1418008   +  503   14591375            PH1598               NP_143453.1          putative thymidine phosphorylase
   1418005..1418589   -  194   14591376            PH1599               NP_143454.1          hypothetical protein
   1418691..1419344   +  217   14591377            PH1600               NP_143455.1          cell division inhibitor
   1419341..1419589   +  82    33359417            PH1600.1n            NP_877859.1          hypothetical protein
---------------------------------------
 operons:
   ORGANISM  Burkholderia pseudomallei 1655       accession no is NZ_AAHR01000057.1 gi is 67669903
    cds                dir len   gi         gene     locus                pid                  product
    <3..785            -  261   67669899            Bpse1_01004520       ZP_00466717.1        COG0583: Transcriptional regulator
    995..2176          +  393   67669900            Bpse1_01004521       ZP_00466718.1        COG0477: Permeases of the major facilitator
    2266..2934         -  222   67669901            Bpse1_01004522       ZP_00466719.1        COG1280: Putative threonine efflux protein
    3033..3872         -  279   67669902            Bpse1_01004523       ZP_00466720.1        COG2084: 3-hydroxyisobutyrate dehydrogenase and
 -->4029..4784         +  251   67669903            Bpse1_01004524       ZP_00466721.1        COG1656: Uncharacterized conserved protein
    4927..5100         -  57    67669904            Bpse1_01004525       ZP_00466722.1        hypothetical protein
    6247..6618         +  123   67669905            Bpse1_01004526       ZP_00466723.1        hypothetical protein
    7827..8090         -  87    67669906            Bpse1_01004527       ZP_00466724.1        hypothetical protein
    8713..10065        +  450   67669907            Bpse1_01004528       ZP_00466725.1        COG1113: Gamma-aminobutyrate permease and
    10379..10693       -  104   67669908            Bpse1_01004529       ZP_00466726.1        COG4654: Cytochrome c551/c552
 ---------------------------------------
   ORGANISM  Mycobacterium tuberculosis F11       accession no is NZ_AAIX01000006.1 gi is 76785598
    cds                dir len   gi         gene     locus                pid                  product
    6396..7709         +  437   76785593            MtubF_01000597       ZP_00772758.1        COG3832: Uncharacterized conserved protein
    7717..8508         +  263   76785594            MtubF_01000598       ZP_00772759.1        COG3324: Predicted enzyme related to
    8553..8930         -  125   76785595            MtubF_01000599       ZP_00772760.1        hypothetical protein
    8887..10734        -  615   76785596            MtubF_01000600       ZP_00772761.1        hypothetical protein
    <10756..12498      -  581   76785597            MtubF_01000601       ZP_00772762.1        hypothetical protein
 -->12820..13578       +  252   76785598            MtubF_01000602       ZP_00772763.1        COG1656: Uncharacterized conserved protein
    13707..14285       -  192   76785599            MtubF_01000603       ZP_00772764.1        hypothetical protein
    14292..14507       +  71    76785600            MtubF_01000604       ZP_00772765.1        COG1077: Actin-like ATPase involved in cell
    14504..14911       +  135   76785601            MtubF_01000605       ZP_00772766.1        COG2402: Predicted nucleic acid-binding protein,
    14971..15657       -  228   76785602            MtubF_01000606       ZP_00772767.1        hypothetical protein
    15811..18444       +  877   76785603            MtubF_01000607       ZP_00772768.1        COG3537: Putative alpha-1,2-mannosidase
 ---------------------------------------
   ORGANISM  Azotobacter vinelandii AvOP  accession no is NZ_AAAU03000001.1 gi is 67154055
    cds                dir len   gi         gene     locus                pid                  product
    836549..837559     -  336   67154050            AvinDRAFT_7923       ZP_00415795.1        Zinc-containing alcohol dehydrogenase
    837638..837919     +  93    67154051            AvinDRAFT_6295       ZP_00415796.1        hypothetical protein
    838236..838529     -  97    67154052            AvinDRAFT_7920       ZP_00415797.1        hypothetical protein
    839091..840014     +  307   67154053            AvinDRAFT_6298       ZP_00415798.1        hypothetical protein
    839931..840953     -  340   67154054            AvinDRAFT_7918       ZP_00415799.1        Patatin
 -->841026..841952     -  308   67154055            AvinDRAFT_7917       ZP_00415800.1        Protein of unknown function DUF82
    841933..842697     +  254   67154056            AvinDRAFT_6300       ZP_00415801.1        Short-chain dehydrogenase/reductase SDR
    842735..843445     -  236   67154057            AvinDRAFT_7915       ZP_00415802.1        RNA-binding S4
    843602..844738     +  378   67154058            AvinDRAFT_6302       ZP_00415803.1        conserved hypothetical protein
    844774..845613     +  279   67154059            AvinDRAFT_6303       ZP_00415804.1        RNA binding S1
    845871..846425     -  184   67154060            AvinDRAFT_7911       ZP_00415805.1        hypothetical protein
 ---------------------------------------
   ORGANISM  Ralstonia solanacearum UW551         accession no is NZ_AAKL01000001.1 gi is 83745680
    cds                dir len   gi         gene     locus                pid                  product
    179624..182833     -  1069  83745675            RRSL_04740           ZP_00942733.1        Transmembrane multidrug-efflux system
    182870..184195     -  441   83745676            RRSL_04741           ZP_00942734.1        Probable transmembrane multidrug efflux system
    184504..185586     -  360   83745677            RRSL_04742           ZP_00942735.1        Hypothetical Protein
    185868..186812     +  314   83745678            RRSL_04743           ZP_00942736.1        Transcriptional regulators, LysR family
    186864..188147     -  427   83745679            RRSL_04744           ZP_00942737.1        Membrane-bound lytic murein transglycosylase B
 -->188712..189545     +  277   83745680            RRSL_04745           ZP_00942738.1        Zinc finger protein
    189660..190679     -  339   83745681            RRSL_04747           ZP_00942739.1        Hypothetical Protein
    190723..191148     -  141   83745682            RRSL_04748           ZP_00942740.1        Hypothetical Protein
    191305..191775     -  156   83745683            RRSL_04749           ZP_00942741.1        Hypothetical Protein
    191832..193229     -  465   83745684            RRSL_04750           ZP_00942742.1        Alpha,alpha-trehalose-phosphate synthase
    193226..194014     -  262   83745685            RRSL_04751           ZP_00942743.1        Trehalose-phosphatase
 ---------------------------------------
   ORGANISM  Burkholderia ambifaria AMMD  accession no is NZ_AAJL01000019.1 gi is 74019866
    cds                dir len   gi         gene     locus                pid                  product
    559..1836          +  425   74019863            BambDRAFT_0382       ZP_00690474.1        Major facilitator superfamily
    1908..2543         -  211   74019864            BambDRAFT_0383       ZP_00690475.1        Lysine exporter protein (LYSE/YGGA)
    2615..3484         -  289   74019865            BambDRAFT_0384       ZP_00690476.1        6-phosphogluconate dehydrogenase, NAD-binding
 -->3582..4388         +  268   74019866            BambDRAFT_0385       ZP_00690477.1        Protein of unknown function DUF82
    5072..6277         +  401   74019867            BambDRAFT_0386       ZP_00690478.1        Secretion protein HlyD
    6288..6986         +  232   74019868            BambDRAFT_0387       ZP_00690479.1        ABC transporter
    7005..8159         +  384   74019869            BambDRAFT_0388       ZP_00690480.1        Protein of unknown function DUF214
    8066..9334         +  422   74019870            BambDRAFT_0389       ZP_00690481.1        Protein of unknown function DUF214
    9348..10685        -  445   74019871            BambDRAFT_0390       ZP_00690482.1        Retinal pigment epithelial membrane protein
 ---------------------------------------
   ORGANISM  Ralstonia metallidurans CH34         accession no is NZ_AAAI03000001.1 gi is 68554875
    cds                dir len   gi         gene     locus                pid                  product
    1135347..1136177   -  276   68554870            RmetDRAFT_5444       ZP_00594215.1        Phosphomethylpyrimidine kinase
    1136475..1137893   +  472   68554871            RmetDRAFT_5445       ZP_00594216.1        S-adenosyl-L-homocysteine hydrolase
    1138027..1138374   +  115   68554872            RmetDRAFT_5446       ZP_00594217.1        Membrane protein of unknown function
    1138422..1139252   +  276   68554873            RmetDRAFT_5447       ZP_00594218.1        5,10-methylenetetrahydrofolate reductase
    1139259..1139621   +  120   68554874            RmetDRAFT_5448       ZP_00594219.1        TfoX, N-terminal
 -->1139673..1140473   +  266   68554875            RmetDRAFT_5449       ZP_00594220.1        Protein of unknown function DUF82
    1140479..1141213   -  244   68554876            RmetDRAFT_5882       ZP_00594221.1        5-formyltetrahydrofolate cyclo-ligase
    1141226..1143193   +  655   68554877            RmetDRAFT_5450       ZP_00594222.1        SLT
    1143294..1144316   +  340   68554878            RmetDRAFT_5451       ZP_00594223.1        putative NADH-ubiquinone oxidoreductase
    1144485..1145174   +  229   68554879            RmetDRAFT_5452       ZP_00594224.1        Glutathione S-transferase, N-terminal
    1145178..1146422   +  414   68554880            RmetDRAFT_5453       ZP_00594225.1        Polynucleotide
 ---------------------------------------
   ORGANISM  Azoarcus sp. EbN1    accession no is CR555306.1 gi is 56311907
    cds                dir len   gi         gene     locus                pid                  product
    469694..470593     -  299   56311902   lysR     ebA811               CAI06547.1           transcription regulator protein
    470625..471470     +  281   56311903            ebA812               CAI06548.1           putative 2-haloalkanoic acid dehalogenase
    471481..472257     +  258   56311904            ebA816               CAI06549.1           predicted permease
    472271..474448     +  725   56311905   glcB     ebA819               CAI06550.1           Malate synthase G
    474456..474974     +  172   56311906            ebA821               CAI06551.1           hypothetical protein
 -->475027..475824     +  265   56311907            ebA822               CAI06552.1           conserved hypothetical protein
    475847..476272     -  141   56311908            ebB21                CAI06553.1           Probably thioesterase
    476497..478122     +  541   56311909            ebA826               CAI06554.1           conserved hypothetical protein
    478206..480257     +  683   56311910            ebA825               CAI06555.1           translation elongation factor G
    480367..480627     +  86    56311911            ebA828               CAI06556.1           predicted membrane protein
    480715..481959     -  414   56311912   icd      ebA829               CAI06557.1           Isocitrate dehydrogenase
 ---------------------------------------
   ORGANISM  Thiobacillus denitrificans ATCC 25259        accession no is NC_007404.1 gi is 74318176
    cds                dir len   gi         gene     locus                pid                  product
    2239499..2241790   +  763   74318171            Tbd_2153             YP_315911.1          Elongator protein 3/MiaB/NifB
    2241817..2242131   +  104   74318172            Tbd_2154             YP_315912.1          hypothetical protein
    2242178..2242663   -  161   74318173            Tbd_2155             YP_315913.1          hypothetical protein
    2242796..2243191   -  131   74318174            Tbd_2156             YP_315914.1          hypothetical protein
    2243263..2243778   -  171   74318175            Tbd_2157             YP_315915.1          hypothetical protein
 -->2243785..2244579   -  264   74318176            Tbd_2158             YP_315916.1          hypothetical protein
    2244620..2246062   -  480   74318177            Tbd_2159             YP_315917.1          Predicted signal transduction protein containing
    2246139..2246543   +  134   74318178            Tbd_2160             YP_315918.1          hypothetical protein
    2246762..2247472   +  236   74318179            Tbd_2161             YP_315919.1          hypothetical protein
    2247614..2248216   +  200   74318180            Tbd_2162             YP_315920.1          hypothetical protein
    2248368..2248991   -  207   74318181            Tbd_2163             YP_315921.1          putative ubiquinone biosynthesis monooxygenase
 ---------------------------------------
   ORGANISM  Burkholderia cenocepacia HI2424      accession no is NZ_AAHL01000050.1 gi is 67666690
    cds                dir len   gi         gene     locus                pid                  product
    44732..45898       -  388   67666687            Bcen2424DRAFT_1948   ZP_00463930.1        Protein of unknown function DUF214
    45907..47061       -  384   67666688            Bcen2424DRAFT_1949   ZP_00463931.1        Protein of unknown function DUF214
    47083..47781       -  232   67666693            Bcen2424DRAFT_1954   ZP_00463936.1        ABC transporter
    47787..49127       -  446   67666689            Bcen2424DRAFT_1950   ZP_00463932.1        Secretion protein HlyD
    49219..49584       +  121   67666691            Bcen2424DRAFT_1952   ZP_00463934.1        hypothetical protein
 -->49664..50443       -  259   67666690            Bcen2424DRAFT_1951   ZP_00463933.1        Protein of unknown function DUF82
 ---------------------------------------
   ORGANISM  Ralstonia solanacearum       accession no is AL646082.1 gi is 17431582
    cds                dir len   gi         gene     locus                pid                  product
    190928..191716     +  262   17431577   otsB                          CAD18255.1           PROBABLE TREHALOSE-PHOSPHATASE PROTEIN
    191713..193110     +  465   17431578   otsA                          CAD18256.1           PROBABLE ALPHA,ALPHA-TREHALOSE-PHOSPHATE
    193175..193636     +  153   17431579   RSp1106                       CAD18257.1           PUTATIVE TRANSCRIPTION REGULATOR PROTEIN
    193782..194207     +  141   17431580   ohr                           CAD18258.1           PROBABLE ORGANIC HYDROPEROXIDE RESISTANCE
    194254..195273     +  339   17431581   RSp1108                       CAD18259.1           PROBABLE LIPASE SIGNAL PEPTIDE PROTEIN
 -->195364..196137     -  257   17431582   RSp1109                       CAD18260.1           CONSERVED HYPOTHETICAL PROTEIN
    196242..197189     -  315   17431583   RSp1110                       CAD18261.1           PROBABLE TRANSCRIPTION REGULATOR PROTEIN
    197471..198544     +  357   17431584   RSp1111                       CAD18262.1           PROBABLE LIPASE PROTEIN
    198849..200165     +  438   17431585   YS02620                       CAD18263.1           PROBABLE TRANSMEMBRANE MULTIDRUG EFFLUX SYSTEM
    200202..203411     +  1069  17431586   YS02621                       CAD18264.1           PROBABLE TRANSMEMBRANE MULTIDRUG-EFFLUX SYSTEM
    203431..204957     +  508   17431587   RSp1114                       CAD18265.1           PUTATIVE OUTER-MEMBRANE DRUG EFFLUX PROTEIN
 ---------------------------------------
   ORGANISM  Burkholderia sp. 383         accession no is CP000151.1 gi is 77965627
    cds                dir len   gi         gene     locus                pid                  product
    258485..259651     -  388   77965622            Bcep18194_A3400      ABB07002.1           ABC efflux pump, inner membrane subunit
    259660..260814     -  384   77965623            Bcep18194_A3401      ABB07003.1           ABC efflux pump, inner membrane subunit
    260836..261534     -  232   77965624            Bcep18194_A3402      ABB07004.1           ABC efflux pump, ATPase subunit
    261542..262747     -  401   77965625            Bcep18194_A3403      ABB07005.1           Secretion protein HlyD family
    262976..263341     +  121   77965626            Bcep18194_A3404      ABB07006.1           hypothetical protein
 -->263420..264184     -  254   77965627            Bcep18194_A3405      ABB07007.1           protein of unknown function DUF82
    264281..265150     +  289   77965628            Bcep18194_A3406      ABB07008.1           6-phosphogluconate dehydrogenase, NAD-binding
    265227..265868     +  213   77965629            Bcep18194_A3407      ABB07009.1           Lysine exporter family protein (LYSE/YGGA)
    265954..267249     -  431   77965630            Bcep18194_A3408      ABB07010.1           Major facilitator superfamily (MFS_1)
    267347..268276     +  309   77965631            Bcep18194_A3409      ABB07011.1           transcriptional regulator, LysR family
    268461..269294     -  277   77965632            Bcep18194_A3410      ABB07012.1           OmpW family protein
 ---------------------------------------
   ORGANISM  Burkholderia dolosa AUO158   accession no is NZ_AAKY01000004.1 gi is 84363527
    cds                dir len   gi         gene     locus                pid                  product
    1619..2452         +  277   84363522            BdolA_01000024       ZP_00988099.1        COG3047: Outer membrane protein W
    2661..3590         -  309   84363523            BdolA_01000025       ZP_00988100.1        COG0583: Transcriptional regulator
    3763..4974         +  403   84363524            BdolA_01000026       ZP_00988101.1        COG0477: Permeases of the major facilitator
    5132..5773         -  213   84363525            BdolA_01000027       ZP_00988102.1        COG1280: Putative threonine efflux protein
    5853..6722         -  289   84363526            BdolA_01000028       ZP_00988103.1        COG2084: 3-hydroxyisobutyrate dehydrogenase and
 -->6816..7574         +  252   84363527            BdolA_01000029       ZP_00988104.1        COG1656: Uncharacterized conserved protein
    7655..7888         -  77    84363528            BdolA_01000030       ZP_00988105.1        hypothetical protein
    8271..9476         +  401   84363529            BdolA_01000031       ZP_00988106.1        COG0845: Membrane-fusion protein
    9478..10173        +  231   84363530            BdolA_01000032       ZP_00988107.1        COG1136: ABC-type antimicrobial peptide
    10192..11346       +  384   84363531            BdolA_01000033       ZP_00988108.1        COG0577: ABC-type antimicrobial peptide
    11353..12519       +  388   84363532            BdolA_01000034       ZP_00988109.1        COG0577: ABC-type antimicrobial peptide
 ---------------------------------------
   ORGANISM  Burkholderia fungorum LB400  accession no is NZ_AAAJ03000013.1 gi is 48782379
    cds                dir len   gi         gene     locus                pid                  product
    25312..26715       +  467   48782374            Bcep02006219         ZP_00278903.1        hypothetical protein
    26813..28057       +  414   48782375            Bcep02006220         ZP_00278904.1        hypothetical protein
    28149..29054       +  301   48782376            Bcep02006221         ZP_00278905.1        COG3047: Outer membrane protein W
    29326..29961       -  211   48782377            Bcep02006222         ZP_00278906.1        COG1280: Putative threonine efflux protein
    30125..30994       -  289   48782378            Bcep02006223         ZP_00278907.1        COG2084: 3-hydroxyisobutyrate dehydrogenase and
 -->31089..31844       +  251   48782379            Bcep02006224         ZP_00278908.1        COG1656: Uncharacterized conserved protein
    31979..32257       +  92    48782380            Bcep02006225         ZP_00278909.1        hypothetical protein
    32412..32621       -  69    48782381            Bcep02006226         ZP_00278910.1        hypothetical protein
    32869..33621       -  250   48782382            Bcep02006227         ZP_00278911.1        COG1414: Transcriptional regulator
    33915..34847       +  310   48782383            Bcep02006228         ZP_00278912.1        COG0697: Permeases of the drug/metabolite
    35988..36665       -  225   48782384            Bcep02006229         ZP_00278913.1        hypothetical protein
 ---------------------------------------
   ORGANISM  Nocardia farcinica IFM 10152         accession no is AP006618.1 gi is 54016307
    cds                dir len   gi         gene     locus                pid                  product
    3012845..3014230   -  461   54016302            nfa28250             BAD57672.1           putative membrane protein
    3014506..3015558   -  350   54016303            nfa28260             BAD57673.1           putative transcriptional regulator
    3015555..3016274   -  239   54016304            nfa28270             BAD57674.1           hypothetical protein
    3016349..3017011   +  220   54016305            nfa28280             BAD57675.1           hypothetical protein
    3017025..3017429   -  134   54016306            nfa28290             BAD57676.1           hypothetical protein
 -->3017514..3018269   -  251   54016307            nfa28300             BAD57677.1           hypothetical protein
    3018301..3019050   -  249   54016308            nfa28310             BAD57678.1           hypothetical protein
    3019190..3020629   -  479   54016309            nfa28320             BAD57679.1           putative amidase
    3020758..3021702   +  314   54016310            nfa28330             BAD57680.1           putative transcriptional regulator
    3021734..3022039   +  101   54016311            nfa28340             BAD57681.1           putative transcriptional regulator
    3022036..3022425   +  129   54016312            nfa28350             BAD57682.1           hypothetical protein
 ---------------------------------------
   ORGANISM  Polaromonas sp. JS666        accession no is NZ_AAFQ02000008.1 gi is 67908809
    cds                dir len   gi         gene     locus                pid                  product
    80959..82386       +  475   67908804            BproDRAFT_2318       ZP_00507202.1        probable benzoyl-CoA oxygenase component
    82466..83746       +  426   67908805            BproDRAFT_2319       ZP_00507203.1        Oxidoreductase FAD/NAD(P)-binding:4Fe-4S
[truncated: 815,908 more chars]
