# Supplementary material for: The prokaryotic antecedents of the ubiquitin-signaling system and the early evolution of ubiquitin-like β-grasp domains
Source: Genome Biol. 2006 Jul 19;7(7):R60. doi: 10.1186/gb-2006-7-7-r60 (PMC1779556; doi:10.1186/gb-2006-7-7-r60)
Supplement: Additional data file 3 — A list of major starting points for PSI-BLAST and HMMer searches and gi numbers detected in the searches conducted with them, along with e values. [file gb-2006-7-7-r60-S3.html]

Hit list for searches with different beta-grasp proteins


Supplementary material- File 3

The prokaryotic antecedents of the Ubiquitin signaling
system

and the early evolution of ubiquitin-like ß-grasp domains

 

Lakshminarayan M. Iyer, A. Maxwell Burroughs and L.
Aravind

 

This file contains hit lists for PSI-BLAST and HMM
searches performed on beta-grasp, E2 (UBC) and JAB domains

 

**A. Hit list for
searches with** **b-grasp
proteins**

------------------------------------------------------------

Reference for composition-based
statistics:

Schaffer, Alejandro A., L.
Aravind, Thomas L. Madden,

Sergei Shavirin, John L.
Spouge, Yuri I. Wolf,

Eugene V. Koonin, and Stephen F. Altschul (2001),

"Improving the
accuracy of PSI-BLAST protein database searches with

composition-based
statistics and other refinements", 
Nucleic Acids Res. 29:2994-3005.

 

Query=
gi|3287920|sp|O32583|THIS\_ECOLI Protein ThiS

         (66 letters)

 

Database: All non-redundant
GenBank CDS

translations+PDB+SwissProt+PIR+PRF
excluding environmental samples

           3,682,060 sequences; 1,264,608,837
total letters

 

Results
from round 57

 

gi|71557339|gb|AAZ36550.1|
thiamine biosynthesis protein ThiS [P...   
86   4e-16

gi|26991781|ref|NP\_747206.1|
sulfur carrier protein ThiS [Pseudo...   
86   5e-16

gi|70733136|ref|YP\_262909.1|
sulfur carrier protein ThiS [Pseudo...   
85   6e-16

gi|9946232|gb|AAG03769.1|
conserved hypothetical protein [Pseudo...   
85   1e-15

gi|94414782|ref|ZP\_01294636.1|
hypothetical protein PaerP\_010033...   
83   3e-15

gi|77385555|gb|ABA77068.1|
ThiS, thiamine-biosynthesis [Pseudomo...   
83   4e-15

gi|49082326|gb|AAT50563.1|
PA0380 [synthetic construct]               
82   9e-15

gi|91798352|gb|ABE60491.1|
thiamine biosynthesis protein ThiS [C...   
82   1e-14

gi|78219002|gb|ABB38351.1|
thiamine biosynthesis protein ThiS [D...   
81   1e-14

gi|67154907|ref|ZP\_00416652.1|
ThiS, thiamine-biosynthesis [Azot...   
81   2e-14

gi|78698313|ref|ZP\_00862816.1|
ThiS, thiamine-biosynthesis [Brad...   
80   2e-14

gi|83311801|ref|YP\_422065.1|
Uncharacterized enzyme of thiazole ...   
80   3e-14

gi|17130691|dbj|BAB73301.1|
asr1344 [Nostoc sp. PCC 7120] >gi|17...   
80   3e-14

gi|94968345|ref|YP\_590393.1|
thiamine biosynthesis protein ThiS ...   
79   5e-14

gi|23130347|ref|ZP\_00112164.1|
COG2104: Sulfur transfer protein ...   
79   5e-14

gi|75704967|gb|ABA24643.1|
ThiS, thiamine-biosynthesis [Anabaena...   
79   5e-14

gi|104779587|ref|YP\_606085.1|
thiamine biosynthesis protein ThiS...   
79   6e-14

gi|77953948|ref|ZP\_00818350.1|
ThiS, thiamine-biosynthesis [Mari...   
79   6e-14

gi|53804598|ref|YP\_113552.1|
thiazole synthase [Methylococcus ca...   
78   7e-14

gi|33564627|emb|CAE43947.1|
conserved hypothetical protein [Bord...   
78   7e-14

gi|82778833|ref|YP\_405182.1|
Sulfur transfer protein involved in...   
78   8e-14

gi|56543208|gb|AAV89362.1|
thiazole biosynthesis protein [Zymomo...   
78   9e-14

gi|82546334|ref|YP\_410281.1|
Sulfur transfer protein [Shigella b...   
78   9e-14

gi|75255488|ref|ZP\_00727287.1|
COG2104: Sulfur transfer protein ...    78  
1e-13

gi|78195384|gb|ABB33151.1|
ThiS, thiamine-biosynthesis [Geobacte...   
78   1e-13

gi|86609832|ref|YP\_478594.1| thiamine biosynthesis protein ThiS ...    78  
1e-13

gi|91785395|ref|YP\_560601.1| Putative sulfur transfer protein in...    78   1e-13

gi|74314485|ref|YP\_312904.1| Sulfur transfer protein [Shigella s...    78  
1e-13

gi|28850898|gb|AAO53977.1| thiamine biosynthesis protein ThiS [P...    77  
2e-13

gi|63258672|gb|AAY39768.1| ThiS, thiamine-biosynthesis [Pseudomo...    77  
2e-13

gi|68561643|ref|ZP\_00600934.1| ThiS, thiamine-biosynthesis [Rubr...    77  
2e-13

gi|74421519|gb|ABA05718.1| ThiS, thiamine-biosynthesis [Nitrobac...    77  
2e-13

gi|77918224|ref|YP\_356039.1| thiamine biosynthesis protein ThiS ...    77  
2e-13

gi|78362777|gb|ABB40742.1| thiamine biosynthesis protein ThiS [T...    77  
2e-13

gi|76883424|gb|ABA58105.1| ThiS, thiamine-biosynthesis [Nitrosoc...    77  
2e-13

gi|16767415|ref|NP\_463030.1| sulfur carrier protein ThiS [Salmon...    77  
2e-13

gi|85715979|ref|ZP\_01046956.1| ThiS, thiamine-biosynthesis [Nitr...    77  
2e-13

gi|86605752|ref|YP\_474515.1| thiamine biosynthesis protein ThiS ...    77  
2e-13

gi|21220586|ref|NP\_626365.1| hypothetical protein SCO2108 [Strep...    77  
3e-13

gi|77957008|ref|ZP\_00821077.1| COG2104: Sulfur transfer protein ...    77   3e-13

gi|91212802|ref|YP\_542788.1| hypothetical protein UTI89\_C3828 [E...    77  
3e-13

gi|48994994|gb|AAT48237.1| sulphur carrier protein [Escherichia ...    77  
3e-13

gi|9105678|gb|AAF83592.1| conserved hypothetical protein [Xylell...    76  
3e-13

gi|36783916|emb|CAE12778.1| ThiS protein [Photorhabdus luminesce...    76  
4e-13

gi|12518920|gb|AAG59188.1| thiamin biosynthesis, probable sulfur...    76  
5e-13

gi|92118391|ref|YP\_578120.1| thiamine biosynthesis protein ThiS ...    76  
5e-13

gi|71836234|ref|ZP\_00676003.1| ThiS, thiamine-biosynthesis [Pelo...    76  
5e-13

gi|71900707|ref|ZP\_00682830.1| ThiS, thiamine-biosynthesis [Xyle...    76  
5e-13

gi|71898215|ref|ZP\_00680389.1| ThiS, thiamine-biosynthesis [Xyle...    75   6e-13

gi|77960648|ref|ZP\_00824510.1|
COG2104: Sulfur transfer protein ...   
75   6e-13

gi|78701988|ref|ZP\_00866433.1|
ThiS, thiamine-biosynthesis [Alka...   
75   6e-13

gi|38704219|ref|NP\_944578.1|
sulfur carrier protein ThiS [Escher...   
75   6e-13

gi|49609716|emb|CAG73149.1|
thiamine biosynthesis protein [Erwin...   
75   7e-13

gi|81299867|ref|YP\_400075.1|
thiamine biosynthesis protein ThiS ...   
75   8e-13

gi|29832636|ref|NP\_827270.1|
sulfur transfer protein involved in...   
75   9e-13

gi|13423328|gb|AAK23856.1|
conserved hypothetical protein [Caulo...   
75   1e-12

gi|83720274|ref|YP\_443511.1|
thiamine biosynthesis protein ThiS ...   
75   1e-12

gi|52427701|gb|AAU48294.1|
thiamine biosynthesis protein ThiS, p...   
75   1e-12

gi|75236721|ref|ZP\_00720794.1|
COG2104: Sulfur transfer protein ...   
74   1e-12

gi|26111193|gb|AAN83376.1|
Thiamin biosynthesis, probable sulfur...   
74   2e-12

gi|83593767|ref|YP\_427519.1|
ThiS, thiamine-biosynthesis [Rhodos...   
74   2e-12

gi|91793129|ref|YP\_562780.1|
thiamine biosynthesis protein ThiS ...   
74   2e-12

gi|78048972|ref|YP\_365147.1|
hypothetical protein XCV3416 [Xanth...   
74   2e-12

gi|74019492|ref|ZP\_00690108.1|
ThiS, thiamine-biosynthesis [Burk...   
74   2e-12

gi|77655361|gb|EAO87002.1|
ThiS, thiamine-biosynthesis [Rhodopse...   
74   2e-12

gi|21109644|gb|AAM38144.1|
conserved hypothetical protein [Xanth...   
74   2e-12

gi|14025577|dbj|BAB52177.1|
thiamin biosynthesis; ThiG [Mesorhiz...   
74   2e-12

gi|90423668|ref|YP\_532038.1|
thiamine biosynthesis protein ThiS ...   
73   2e-12

gi|27381767|ref|NP\_773296.1|
thiamin biosynthesis protein homolo...   
73   2e-12

gi|74318145|ref|YP\_315885.1|
thiamine-biosynthesis protein ThiS ...   
73   2e-12

gi|107024240|ref|YP\_622567.1|
thiamine biosynthesis protein ThiS...    73   3e-12

gi|84360622|ref|ZP\_00985320.1| COG2104: Sulfur transfer protein ...    73  
3e-12

gi|56178887|gb|AAV81609.1| Thiamine biosynthesis protein ThiS [I...    73  
3e-12

gi|56130244|gb|AAV79750.1| thiamine biosynthesis protein [Salmon...    73  
4e-12

gi|99910773|ref|ZP\_01317738.1| hypothetical protein Bpse1\_030028...    73  
4e-12

gi|84690418|gb|EAQ16259.1| thiazole biosynthesis protein [Parvul...    73  
4e-12

gi|83858499|ref|ZP\_00952021.1| thiazole biosynthesis protein [Oc...    73  
4e-12

gi|68246504|gb|EAN28602.1| ThiS, thiamine-biosynthesis [Magnetoc...    72  
5e-12

gi|88949122|ref|ZP\_01151740.1| ThiS, thiamine-biosynthesis [Halo...    72  
6e-12

gi|88932583|ref|ZP\_01138273.1| ThiS, thiamine-biosynthesis [Acid...    72  
7e-12

gi|45437725|gb|AAS63275.1| Sulfur transfer protein involved in t...    72  
7e-12

gi|28199747|ref|NP\_780061.1| hypothetical protein PD1878 [Xylell...    72  
7e-12

gi|77965731|gb|ABB07111.1| ThiS, thiamine-biosynthesis [Burkhold...    72  
8e-12

gi|89092458|ref|ZP\_01165412.1| sulfur carrier protein ThiS [Ocea...    72  
8e-12

gi|90581019|ref|ZP\_01236819.1| sulfur carrier protein ThiS [Vibr...    72  
8e-12

gi|46202840|ref|ZP\_00052512.2| COG2022: Uncharacterized enzyme o...    72  
8e-12

gi|71550737|ref|ZP\_00670826.1| ThiS, thiamine-biosynthesis [Nitr...    72  
9e-12

gi|87306448|ref|ZP\_01088595.1| thiamine biosynthesis protein Thi...    72  
9e-12

gi|67922608|ref|ZP\_00516114.1| ThiS, thiamine-biosynthesis [Croc...    72  
9e-12

gi|51587924|emb|CAH19527.1| thiamin biosynthesis ThiS [Yersinia ...    72  
9e-12

gi|71909473|ref|YP\_287060.1| ThiS, thiamine-biosynthesis [Dechlo...    72  
9e-12

gi|90205897|ref|ZP\_01208535.1| ThiS, thiamine-biosynthesis [Myco...    72  
1e-11

gi|82701206|ref|YP\_410772.1| thiamine biosynthesis protein ThiS ...    71  
1e-11

gi|58582994|ref|YP\_202010.1| hypothetical protein XOO3371 [Xanth...    71  
1e-11

gi|39936636|ref|NP\_948912.1| putative thiamin biosynthesis ThiS ...    71  
1e-11

gi|72161447|ref|YP\_289104.1| ThiS, thiamine-biosynthesis [Thermo...    71  
1e-11

gi|91977895|ref|YP\_570554.1| thiamine biosynthesis protein ThiS ...    71  
1e-11

gi|77741142|ref|ZP\_00809626.1| ThiS, thiamine-biosynthesis [Rhod...    71  
1e-11

gi|88811204|ref|ZP\_01126460.1| hypothetical protein NB231\_10388 ...    71  
1e-11

gi|77977808|ref|ZP\_00833248.1| COG2104: Sulfur transfer protein ...    71  
2e-11

gi|91203815|emb|CAJ71468.1| similar to thiS (Thiamine biosynthes...    71  
2e-11

gi|89338808|ref|ZP\_01191573.1| ThiS, thiamine-biosynthesis [Myco...    71  
2e-11

gi|86749080|ref|YP\_485576.1| thiamine biosynthesis protein ThiS ...    71  
2e-11

gi|21646638|gb|AAM71936.1| thiamine biosynthesis protein ThiS [C...    71  
2e-11

gi|71545061|ref|ZP\_00666026.1| ThiS, thiamine-biosynthesis [Synt...    70  
2e-11

gi|30248304|ref|NP\_840374.1| DUF170 [Nitrosomonas europaea ATCC ...    70  
2e-11

gi|71674937|ref|ZP\_00672683.1| ThiS, thiamine-biosynthesis [Tric...    70   3e-11

gi|9654459|gb|AAF93242.1| thiS protein [Vibrio cholerae O1 biova...    70  
3e-11

gi|67919394|ref|ZP\_00512973.1| ThiS, thiamine-biosynthesis [Chlo...    70  
3e-11

gi|87201302|ref|YP\_498559.1| thiamine biosynthesis protein ThiS ...    70  
3e-11

gi|68055198|ref|ZP\_00539345.1| ThiS, thiamine-biosynthesis [Exig...    70  
3e-11

gi|94495482|ref|ZP\_01302062.1| thiamine biosynthesis protein Thi...    70   3e-11

gi|27360519|gb|AAO09455.1| Sulfur transfer protein involved in t...    70  
3e-11

gi|92916053|ref|ZP\_01284674.1|
ThiS, thiamine-biosynthesis [Myco...   
70   4e-11

gi|78497576|gb|ABB44116.1|
ThiS, thiamine-biosynthesis [Thiomicr...   
69   4e-11

gi|35210963|dbj|BAC88343.1|
ycf40 [Gloeobacter violaceus PCC 742...   
69   5e-11

gi|92909569|ref|ZP\_01278346.1|
ThiS, thiamine-biosynthesis [Myco...   
69   5e-11

gi|39982460|gb|AAR33920.1|
conserved hypothetical protein [Geoba...   
69   5e-11

gi|56379001|dbj|BAD74909.1|
hypothetical conserved protein [Geob...   
69   5e-11

gi|89901242|ref|YP\_523713.1|
thiamine biosynthesis protein ThiS ...   
69   5e-11

gi|77972245|ref|ZP\_00827817.1|
COG2104: Sulfur transfer protein ...   
69   6e-11

gi|95930338|ref|ZP\_01313075.1|
thiamine biosynthesis protein Thi...   
69   6e-11

gi|85666193|ref|ZP\_01028417.1|
hypothetical protein Badol\_010010...   
69   6e-11

gi|67875153|ref|ZP\_00504638.1|
ThiS, thiamine-biosynthesis [Clos...   
69   6e-11

gi|69300050|ref|ZP\_00621926.1|
ThiS, thiamine-biosynthesis [Sili...   
69   6e-11

gi|1723343|sp|P51344|YCF40\_PORPU
Hypothetical 8.1 kDa protein yc...   
69   6e-11

gi|89203238|ref|ZP\_01181926.1|
ThiS, thiamine-biosynthesis [Baci...   
68   7e-11

gi|47501163|gb|AAT29839.1|
conserved hypothetical protein [Bacil...   
68   7e-11

gi|89074422|ref|ZP\_01160899.1|
sulfur carrier protein ThiS [Phot...   
68   7e-11

gi|67930072|ref|ZP\_00523248.1|
ThiS, thiamine-biosynthesis [Soli...   
68   7e-11

gi|29894449|gb|AAP07739.1|
ThiS protein [Bacillus cereus ATCC 14...   
68   8e-11

gi|88858884|ref|ZP\_01133525.1|
putative ThiS protein [Pseudoalte...   
68   8e-11

gi|99078622|ref|YP\_611880.1|
thiamine biosynthesis protein ThiS ...   
68   8e-11

gi|106882327|ref|ZP\_01349730.1|
thiamine biosynthesis protein Th...   
68   9e-11

gi|89103189|ref|ZP\_01175778.1|
COG2104: Sulfur transfer protein ...   
68   9e-11

gi|34541692|ref|NP\_906171.1|
thiS protein [Porphyromonas gingiva...    68  
9e-11

gi|56680462|gb|AAV97128.1|
thiamine biosynthesis protein ThiS [S...   
68   9e-11

gi|86148246|ref|ZP\_01066542.1|
sulfur carrier protein ThiS [Vibr...   
68   1e-10

gi|21114375|gb|AAM42419.1|
conserved hypothetical protein [Xanth...   
68   1e-10

gi|84393666|ref|ZP\_00992417.1|
sulfur carrier protein ThiS [Vibr...   
68   1e-10

gi|90021102|ref|YP\_526929.1|
hypothetical protein Sde\_1455 [Sacc...   
68   1e-10

gi|49331838|gb|AAT62484.1|
thiamine biosynthesis protein [Bacill...   
68   1e-10

gi|22294532|dbj|BAC08362.1|
ycf40 [Thermosynechococcus elongatus...   
68   1e-10

gi|89207101|ref|ZP\_01185649.1|
ThiS, thiamine-biosynthesis [Baci...   
68   1e-10

gi|85709842|ref|ZP\_01040907.1|
thiazole biosynthesis protein [Er...   
68   1e-10

gi|52216684|dbj|BAD49277.1|
ThiS protein involved in thiamine bi...   
68   1e-10

gi|91228042|ref|ZP\_01262127.1|
sulfur carrier protein ThiS [Vibr...   
68   1e-10

gi|67938835|ref|ZP\_00531354.1|
ThiS, thiamine-biosynthesis [Chlo...   
67   2e-10

gi|88608780|ref|YP\_505946.1|
thiamine biosynthesis protein ThiS ...   
67   2e-10

gi|90994535|ref|YP\_537025.1|
hypothetical protein 71 [Porphyra y...   
67   2e-10

gi|83954400|ref|ZP\_00963120.1|
thiamine biosynthesis protein Thi...   
67   2e-10

gi|87125482|ref|ZP\_01081327.1|
hypothetical protein RS9917\_01876...   
67   2e-10

gi|17983762|gb|AAL52915.1|
hypothetical protein [Brucella melite...   
67   2e-10

gi|82499654|ref|ZP\_00885094.1|
ThiS, thiamine-biosynthesis [Cald...   
67   2e-10

gi|90412774|ref|ZP\_01220775.1|
hypothetical protein P3TCK\_22740 ...   
67   2e-10

gi|32262239|gb|AAP77287.1|
conserved hypothetical protein [Helic...   
67   2e-10

gi|71481492|ref|ZP\_00661198.1|
ThiS, thiamine-biosynthesis [Pros...   
67   2e-10

gi|78171431|gb|ABB28527.1|
ThiS, thiamine-biosynthesis [Chlorobi...   
67   2e-10

gi|49238090|emb|CAF27297.1|
Thiamin biosynthesis, thiG1 [Bartone...   
67   3e-10

gi|77917953|ref|YP\_355768.1|
thiamine biosynthesis protein ThiS ...   
67   3e-10

gi|29337957|gb|AAO75760.1|
thiS protein, involved in thiamine bi...   
67   3e-10

gi|58617523|ref|YP\_196722.1|
hypothetical protein ERGA\_CDS\_07960...   
67   3e-10

gi|93453080|gb|EAT03559.1|
ThiS, thiamine-biosynthesis [delta pr...   
66   4e-10

gi|93454223|gb|EAT04541.1| ThiS, thiamine-biosynthesis [delta pr...    66  
4e-10

gi|32034642|ref|ZP\_00134789.1| COG2104: Sulfur transfer protein ...    66   4e-10

gi|23346995|gb|AAN29165.1|
thiamine biosynthesis protein ThiS [B...   
66   4e-10

gi|86137736|ref|ZP\_01056312.1|
hypothetical protein MED193\_07738...   
66   4e-10

gi|41409997|ref|NP\_962833.1|
sulfur carrier protein ThiS [Mycoba...   
66   4e-10

gi|56909742|dbj|BAD64269.1|
thiamine biosynthesis protein ThiS [...   
66   4e-10

gi|53754222|emb|CAH15699.1|
hypothetical protein [Legionella pne...   
66   4e-10

gi|88936313|ref|ZP\_01141926.1|
ThiS, thiamine-biosynthesis [Geob...   
66   4e-10

gi|71367699|ref|ZP\_00658218.1|
ThiS, thiamine-biosynthesis [Noca...   
66   4e-10

gi|88809116|ref|ZP\_01124625.1|
hypothetical protein WH7805\_05471...   
66   5e-10

gi|34499222|ref|NP\_903437.1|
hypothetical protein CV3767 [Chromo...   
66   5e-10

gi|90407317|ref|ZP\_01215503.1|
sulfur carrier protein ThiS [Psyc...   
66   5e-10

gi|6460589|gb|AAF12295.1|
conserved hypothetical protein [Deinoc...   
66   5e-10

gi|94677055|ref|YP\_588522.1|
thiamine biosynthesis protein ThiS ...   
66   5e-10

gi|11465486|ref|NP\_045123.1|
hypothetical protein CycaCp106 [Cya...   
66   5e-10

gi|58579469|ref|YP\_197681.1|
hypothetical protein ERWE\_CDS\_08050...   
66   6e-10

gi|68192288|gb|EAN06942.1|
ThiS, thiamine-biosynthesis [Mesorhiz...   
65   6e-10

gi|68547475|ref|ZP\_00587011.1|
ThiS, thiamine-biosynthesis [Shew...    65   7e-10

gi|54018820|dbj|BAD60190.1| putative sulfur transfer protein [No...    65  
7e-10

gi|16331468|ref|NP\_442196.1| hypothetical protein ssr0102 [Synec...    65  
7e-10

gi|83643048|ref|YP\_431483.1| thiamine biosynthesis protein ThiS ...    65  
8e-10

gi|77996033|gb|ABB14932.1| thiamine biosynthesis protein ThiS [C...    65  
8e-10

gi|49239551|emb|CAF25908.1| Thiamin biosynthesis, thiG1 [Bartone...    65  
9e-10

gi|83952602|ref|ZP\_00961333.1| thiamine biosynthesis protein Thi...    65  
1e-09

gi|86605021|ref|YP\_473784.1| MoaD family protein [Synechococcus ...    65  
1e-09

gi|82499138|ref|ZP\_00884589.1| ThiS, thiamine-biosynthesis [Cald...    65  
1e-09

gi|53751264|emb|CAH12675.1| hypothetical protein [Legionella pne...    65  
1e-09

gi|77996070|gb|ABB14969.1| thiamine biosynthesis protein ThiS [C...    65  
1e-09

gi|85705982|ref|ZP\_01037078.1| thiamine biosynthesis protein Thi...    65  
1e-09

gi|17741061|gb|AAL43548.1| thiamin biosynthesis protein ThiG [Ag...    64  
1e-09

gi|28808050|dbj|BAC61287.1| ThiS protein [Vibrio parahaemolyticu...    64  
1e-09

gi|85375395|ref|YP\_459457.1| thiazole biosynthesis protein [Eryt...    64  
1e-09

gi|68230363|ref|ZP\_00569547.1| ThiS, thiamine-biosynthesis [Fran...    64  
1e-09

gi|39934244|ref|NP\_946520.1| molybdopterin converting factor, su...    64  
1e-09

gi|91975759|ref|YP\_568418.1| molybdopterin converting factor, su...    64  
2e-09

gi|84517612|ref|ZP\_01004961.1| Sulfur transfer protein [Prochlor...    64  
2e-09

gi|86748300|ref|YP\_484796.1|
molybdopterin converting factor, su...   
64   2e-09

gi|88932029|ref|ZP\_01137722.1|
conserved hypothetical protein [A...   
64   2e-09

gi|78167320|gb|ABB24418.1|
ThiS, thiamine-biosynthesis [Pelodict...   
64   2e-09

gi|83751114|ref|ZP\_00947528.1|
COG2104: Sulfur transfer protein ...   
64   2e-09

gi|67935886|ref|ZP\_00528902.1|
ThiS, thiamine-biosynthesis [Chlo...   
64   2e-09

gi|68550360|ref|ZP\_00589811.1|
ThiS, thiamine-biosynthesis [Pelo...   
64   2e-09

gi|77683436|ref|ZP\_00798885.1|
ThiS, thiamine-biosynthesis [Alka...   
64   2e-09

gi|23335286|ref|ZP\_00120523.1|
COG2104: Sulfur transfer protein ...   
64   2e-09

gi|91776986|ref|YP\_546742.1|
thiamine biosynthesis protein ThiS ...   
64   2e-09

gi|49530467|emb|CAG68179.1|
C-terminally thiocarboxylated form i...   
63   2e-09

gi|89101139|ref|ZP\_01173973.1|
sulfur carrier protein ThiS [Baci...   
63   2e-09

gi|33634551|emb|CAE20537.1|
DUF170 [Prochlorococcus marinus str....   
63   3e-09

gi|65318157|ref|ZP\_00391116.1|
COG2104: Sulfur transfer protein ...   
63   3e-09

gi|67939265|ref|ZP\_00531772.1|
ThiS, thiamine-biosynthesis [Chlo...   
63   3e-09

gi|98663026|dbj|GAA00171.1|
unnamed protein product [Pelotomacul...   
63   3e-09

gi|88607118|ref|YP\_504702.1|
thiamine biosynthesis protein ThiS ...   
63   4e-09

gi|48855687|ref|ZP\_00309845.1|
COG2104: Sulfur transfer protein ...   
63   4e-09

gi|87301110|ref|ZP\_01083951.1|
hypothetical protein WH5701\_14531...   
63   5e-09

gi|88803158|ref|ZP\_01118684.1|
hypothetical protein PI23P\_11237 ...   
63   5e-09

gi|16762296|ref|NP\_457913.1|
sulfur carrier protein ThiS [Salmon...   
62   5e-09

gi|34484090|emb|CAE11086.1|
hypothetical protein [Wolinella succ...   
62   5e-09

gi|46449919|gb|AAS96568.1|
thiamine biosynthesis protein ThiS [D...   
62   6e-09

gi|78169364|gb|ABB26461.1|
ThiS, thiamine-biosynthesis [Synechoc...   
62   6e-09

gi|84501020|ref|ZP\_00999255.1|
thiamine biosynthesis protein Thi...    62  
6e-09

gi|29540944|gb|AAO89888.1|
thiamine biosynthesis protein ThiS [C...   
62   8e-09

gi|57160377|dbj|BAD86307.1|
molybdopterin converting factor, sub...   
62   8e-09

gi|90574748|ref|ZP\_01231240.1|
hypothetical protein CdifQ\_020018...   
62   9e-09

gi|88947032|ref|ZP\_01150061.1|
ThiS, thiamine-biosynthesis [Desu...   
62   9e-09

gi|68263525|emb|CAI37013.1|
thiamin biosynthesis ThiS [Corynebac...   
62   9e-09

gi|68208686|ref|ZP\_00560784.1|
ThiS, thiamine-biosynthesis [Desu...   
62   1e-08

gi|60493471|emb|CAH08257.1|
putative thiamine biosynthesis-relat...   
62   1e-08

gi|66964609|ref|ZP\_00412178.1|
ThiS, thiamine-biosynthesis [Arth...   
61   1e-08

gi|85859825|ref|YP\_462027.1|
thiS family protein [Syntrophus aci...   
61   1e-08

gi|78194032|gb|ABB31799.1|
ThiS, thiamine-biosynthesis [Geobacte...   
61   1e-08

gi|92914605|ref|ZP\_01283231.1|
ThiamineS [Mycobacterium sp. KMS]...   
61   2e-08

gi|67873784|ref|ZP\_00503771.1|
ThiS, thiamine-biosynthesis [Clos...   
61   2e-08

gi|94986022|ref|YP\_605386.1|
thiamine biosynthesis protein ThiS ...   
61   2e-08

gi|7227333|gb|AAF42389.1|
thiamine biosynthesis protein ThiS [Ne...   
61   2e-08

gi|56416396|ref|YP\_153470.1|
hypothetical protein AM035 [Anaplas...   
60   2e-08

gi|88657629|ref|YP\_507028.1|
thiamine biosynthesis protein ThiS ...   
60   2e-08

gi|94490916|ref|ZP\_01298140.1|
hypothetical protein CburD\_010020...   
60   2e-08

gi|33640199|emb|CAE19734.1|
DUF170 [Prochlorococcus marinus subs...   
60   2e-08

gi|69953450|ref|ZP\_00640580.1|
ThiS, thiamine-biosynthesis [Shew...   
60   2e-08

gi|76874357|emb|CAI85578.1|
putative ThiS protein [Pseudoalterom...   
60   3e-08

gi|10174049|dbj|BAB05151.1|
BH1432 [Bacillus halodurans C-125] >...   
60   3e-08

gi|52002882|gb|AAU22824.1|
ThiS [Bacillus licheniformis ATCC 145...   
60   3e-08

gi|78693266|ref|ZP\_00857780.1|
Molybdopterin converting factor, ...   
60   3e-08

gi|57636146|gb|AAW52934.1|
thiamine biosynthesis protein ThiS [S...   
60   3e-08

gi|86610242|ref|YP\_479004.1|
MoaD family protein [Synechococcus ...   
60   3e-08

gi|32397914|emb|CAD73915.1|
conserved hypothetical protein [Rhod...   
60   3e-08

gi|90419510|ref|ZP\_01227420.1|
molybdopterin converting factor s...   
60   3e-08

gi|89339708|ref|ZP\_01192306.1|
ThiamineS [Mycobacterium flavesce...    60  
3e-08

gi|51209896|ref|YP\_063560.1|
conserved hypothetical plastid prot...   
60   3e-08

gi|94501853|ref|ZP\_01308364.1|
hypothetical protein RED65\_02138 ...   
60   3e-08

gi|82744797|ref|ZP\_00907312.1|
ThiS, thiamine-biosynthesis [Clos...   
60   3e-08

gi|71676258|ref|ZP\_00674000.1|
MoaD, archaeal [Trichodesmium ery...   
60   3e-08

gi|78196898|gb|ABB34663.1|
thiamine biosynthesis protein ThiS [S...   
60   4e-08

gi|47571794|ref|ZP\_00241843.1|
COG2104: Sulfur transfer protein ...   
60   4e-08

gi|35212628|dbj|BAC90002.1|
gsl2061 [Gloeobacter violaceus PCC 7...   
60   4e-08

gi|7379114|emb|CAB83663.1|
hypothetical protein NMA0362 [Neisser...   
60   4e-08

gi|86158941|ref|YP\_465726.1|
thiamine biosynthesis protein ThiS ...   
60   4e-08

gi|77686501|ref|ZP\_00801846.1|
ThiS, thiamine-biosynthesis [Alka...   
59   4e-08

gi|59802314|ref|YP\_209026.1|
hypothetical protein NGO2006 [Neiss...   
59   5e-08

gi|17427119|emb|CAD13639.1|
probable sulfur transfer protein thi...   
59   6e-08

gi|52696120|pdb|1VJK|A
Chain A, Putative Molybdopterin Convertin...   
59   6e-08

gi|10640334|emb|CAC12148.1|
MoaD (involved in molybdopterin synt...   
59   6e-08

gi|50876627|emb|CAG36467.1|
hypothetical protein [Desulfotalea p...   
59   6e-08

gi|88792777|ref|ZP\_01108495.1|
hypothetical protein MADE\_11015 [...   
59   6e-08

gi|18892532|gb|AAL80667.1|
molybdopterin converting factor, subu...   
59   6e-08

gi|87119895|ref|ZP\_01075791.1|
thiamine biosynthesis protein Thi...   
59   6e-08

gi|68446288|dbj|BAE03872.1|
unnamed protein product [Staphylococ...   
59   7e-08

gi|18145266|dbj|BAB81309.1|
conserved hypothetical protein [Clos...   
59   7e-08

gi|33238327|gb|AAQ00393.1|
Sulfur transfer protein [Prochlorococ...   
59   7e-08

gi|34495641|ref|NP\_899856.1|
molybdopterin-converting factor sub...   
58   7e-08

gi|57159324|dbj|BAD85254.1|
molybdopterin converting factor, sub...   
58   8e-08

gi|23347498|gb|AAN29626.1|
molybdopterin converting factor, subu...   
58   8e-08

gi|76261574|ref|ZP\_00769179.1|
Molybdopterin converting factor, ...   
58   8e-08

gi|84713026|ref|ZP\_01020831.1|
ThiS, thiamine-biosynthesis [Pola...   
58   8e-08

gi|23016726|ref|ZP\_00056479.1|
COG1977: Molybdopterin converting...   
58   8e-08

gi|24373989|ref|NP\_718032.1|
thiS protein, putative [Shewanella ...    58  
9e-08

gi|23011959|ref|ZP\_00052165.1|
COG2104: Sulfur transfer protein ...   
58   9e-08

gi|86155194|ref|ZP\_01073360.1|
thiamine biosynthesis protein Thi...   
58   1e-07

gi|38198932|emb|CAE48543.1|
Conserved hypothetical protein [Cory...   
58   1e-07

gi|5458838|emb|CAB50325.1|
moaD molybdopterin synthase, small su...   
58   1e-07

gi|33866139|ref|NP\_897698.1|
DUF170 [Synechococcus sp. WH 8102] ...   
58   1e-07

gi|86741757|ref|YP\_482157.1|
thiamine biosynthesis protein ThiS ...   
58   1e-07

gi|78713252|gb|ABB50429.1|
thiamine biosynthesis protein ThiS [P...   
58   1e-07

gi|33356787|ref|NP\_127095.2|
molybdopterin converting factor, su...   
58   1e-07

gi|90203237|ref|ZP\_01205883.1|
ThiamineS [Mycobacterium vanbaale...   
58   1e-07

gi|75704874|gb|ABA24550.1|
ThiamineS [Anabaena variabilis ATCC 2...   
58   1e-07

gi|18892299|gb|AAL80469.1|
molybdopterin converting factor, subu...   
58   1e-07

gi|85716037|ref|ZP\_01047014.1|
molybdopterin converting factor, ...   
58   1e-07

gi|74421578|gb|ABA05777.1|
molybdopterin converting factor, subu...   
58   1e-07

gi|106888351|ref|ZP\_01355551.1|
molybdopterin converting factor,...   
58   1e-07

gi|6460436|gb|AAF12145.1|
molybdenum cofactor biosynthesis prote...   
58   2e-07

gi|13092619|emb|CAC29806.1|
conserved hypothetical protein [Myco...   
58   2e-07

gi|55771441|dbj|BAD69882.1|
molybdenum cofactor biosynthesis pro...   
58   2e-07

gi|46201345|ref|ZP\_00055205.2|
COG1977: Molybdopterin converting...   
57   2e-07

gi|57240562|ref|ZP\_00368511.1|
thiamine biosynthesis protein Thi...   
57   2e-07

gi|9655490|gb|AAF94186.1|
molybdenum cofactor biosynthesis prote...   
57   2e-07

gi|88795121|ref|ZP\_01110822.1|
molybdopterin converting factor, ...   
57   2e-07

gi|73663790|ref|YP\_302570.1|
hypothetical protein SSPP134 [Staph...   
57   2e-07

gi|46200249|ref|YP\_005916.1|
molybdopterin (MPT) converting fact...   
57   2e-07

gi|76794014|ref|ZP\_00776492.1|
ThiS, thiamine-biosynthesis [Pseu...   
57   2e-07

gi|92118611|ref|YP\_578340.1|
molybdopterin converting factor, su...   
57   2e-07

gi|78691290|ref|ZP\_00855913.1|
ThiS, thiamine-biosynthesis [Shew...   
57   2e-07

gi|77812832|ref|ZP\_00812115.1|
ThiS, thiamine-biosynthesis [Shew...   
57   2e-07

gi|83312895|ref|YP\_423159.1|
Molybdopterin converting factor, sm...   
57   2e-07

gi|74019145|ref|ZP\_00689763.1|
Molybdopterin converting factor, ...   
57   2e-07

gi|89056408|ref|YP\_511859.1|
molybdopterin converting factor, su...   
57   2e-07

gi|75823609|ref|ZP\_00753099.1|
COG1977: Molybdopterin converting...    57  
2e-07

gi|89362382|ref|ZP\_01200190.1|
ThiS, thiamine-biosynthesis [Xant...   
57   2e-07

gi|83815881|ref|YP\_445359.1|
thiamine biosynthesis protein ThiS ...   
57   2e-07

gi|62424302|ref|ZP\_00379450.1|
COG2104: Sulfur transfer protein ...   
57   2e-07

gi|30468123|ref|NP\_849010.1|
ORF67 [Cyanidioschyzon merolae stra...   
57   2e-07

gi|90022254|ref|YP\_528081.1|
Helix-turn-helix, AraC type [Saccha...   
57   2e-07

gi|68542219|ref|ZP\_00581954.1|
ThiS, thiamine-biosynthesis [Shew...   
57   2e-07

gi|91762145|ref|ZP\_01264110.1|
ThiS family protein [Candidatus P...   
57   2e-07

gi|68545076|ref|ZP\_00584627.1|
Molybdopterin converting factor, ...   
57   2e-07

gi|94984437|ref|YP\_603801.1|
molybdopterin converting factor, su...   
57   3e-07

gi|71907414|ref|YP\_285001.1|
Molybdopterin converting factor, su...   
57   3e-07

gi|91775615|ref|YP\_545371.1|
molybdopterin converting factor, su...   
57   3e-07

gi|57168917|ref|ZP\_00368047.1|
thiamine biosynthesis protein Thi...   
57   3e-07

gi|33359306|ref|NP\_877770.1|
putative molybdopterin converting f...   
57   3e-07

gi|98659923|dbj|GAA02748.1|
unnamed protein product [Pelotomacul...   
57   3e-07

gi|84362038|ref|ZP\_00986675.1|
COG1977: Molybdopterin converting...   
57   3e-07

gi|75819545|ref|ZP\_00749620.1|
COG1977: Molybdopterin converting...   
57   3e-07

gi|16330728|ref|NP\_441456.1|
hypothetical protein slr0821 [Synec...   
56   4e-07

gi|57166737|gb|AAW35516.1|
thiamine biosynthesis protein ThiS [C...   
56   4e-07

gi|67543768|ref|ZP\_00421699.1|
Molybdopterin converting factor, ...   
56   4e-07

gi|82776146|ref|YP\_402493.1|
molybdopterin biosynthesis [Shigell...   
56   5e-07

gi|22294706|dbj|BAC08535.1|
tsr0983 [Thermosynechococcus elongat...   
56   5e-07

gi|77951750|ref|ZP\_00816169.1|
Molybdopterin converting factor, ...   
56   5e-07

gi|55670077|pdb|1TYG|G
Chain G, Structure Of The Thiazole Syntha...   
56   5e-07

gi|16759729|ref|NP\_455346.1|
molybdopterin converting factor, su...   
56   5e-07

gi|90407696|ref|ZP\_01215875.1|
molybdopterin biosynthesis protei...   
56   5e-07

gi|67922943|ref|ZP\_00516439.1|
ThiamineS [Crocosphaera watsonii ...   
56   6e-07

gi|77742224|ref|ZP\_00810703.1|
Molybdopterin converting factor, ...    56   6e-07

gi|2633522|emb|CAB13025.1| thiS [Bacillus subtilis subsp. subtil...    56  
6e-07

gi|42782629|ref|NP\_979876.1|
molybdopterin converting factor, su...   
55   6e-07

gi|87305978|ref|ZP\_01088131.1|
thiamine biosynthesis protein Thi...   
55   6e-07

gi|88798339|ref|ZP\_01113924.1|
putative ThiS protein [Reinekea s...   
55   6e-07

gi|94969084|ref|YP\_591132.1|
molybdopterin converting factor, su...   
55   6e-07

gi|90422422|ref|YP\_530792.1|
molybdopterin converting factor, su...   
55   7e-07

gi|34483110|emb|CAE10109.1|
hypothetical protein [Wolinella succ...   
55   7e-07

gi|18160633|gb|AAL63972.1|
conserved hypothetical protein [Pyrob...   
55   7e-07

gi|94496324|ref|ZP\_01302901.1|
molybdopterin converting factor, ...   
55   7e-07

gi|72119091|gb|AAZ61354.1|
Molybdopterin converting factor, subu...   
55   8e-07

gi|77814639|ref|ZP\_00813897.1|
Molybdopterin converting factor, ...   
55   8e-07

gi|62179373|ref|YP\_215790.1|
molybdopterin biosynthesis protein ...   
55   8e-07

gi|23129177|ref|ZP\_00111010.1|
COG1977: Molybdopterin converting...   
55   8e-07

gi|67156787|ref|ZP\_00418284.1|
ThiamineS [Azotobacter vinelandii...   
55   8e-07

gi|15830116|ref|NP\_308889.1|
molybdopterin biosynthesis protein ...   
55   9e-07

gi|26107155|gb|AAN79339.1|
Molybdopterin converting factor subun...   
55   9e-07

gi|56383279|gb|AAN42369.2|
molybdopterin biosynthesis protein D ...   
55   9e-07

gi|49330814|gb|AAT61460.1|
molybdopterin converting factor, subu...   
55   9e-07

gi|46156355|ref|ZP\_00204683.1|
COG1977: Molybdopterin converting...   
55   9e-07

gi|83312024|ref|YP\_422288.1|
hypothetical protein amb2925 [Magne...   
55   1e-06

gi|71062545|gb|AAZ21548.1|
ThiS family [Candidatus Pelagibacter ...   
55   1e-06

gi|23467044|ref|ZP\_00122629.1|
COG1977: Molybdopterin converting...    55   1e-06

gi|5070641|gb|AAD39227.1| MoaD-like protein [Pseudomonas stutzer...    55  
1e-06

gi|78366583|ref|ZP\_00836862.1|
ThiS, thiamine-biosynthesis [Shew...    55   1e-06

gi|12720897|gb|AAK02707.1| MoaD [Pasteurella multocida subsp. mu...    55  
1e-06

gi|84515715|ref|ZP\_01003076.1|
molybdopterin converting factor, ...   
55   1e-06

gi|47568924|ref|ZP\_00239616.1|
molybdopterin converting factor, ...   
55   1e-06

gi|78686690|ref|ZP\_00851454.1|
ThiS, thiamine-biosynthesis [Shew...   
55   1e-06

gi|49180290|gb|AAT55666.1|
molybdopterin converting factor, subu...  
 55   1e-06

gi|47572157|ref|ZP\_00242203.1|
COG1977: Molybdopterin converting...   
55   1e-06

gi|53802819|ref|YP\_115441.1|
molybdopterin converting factor, su...   
55   1e-06

gi|90411674|ref|ZP\_01219684.1|
hypothetical protein P3TCK\_16464 ...   
54   1e-06

gi|27382583|ref|NP\_774112.1|
molybdopterin converting factor sma...   
54   1e-06

gi|85705896|ref|ZP\_01036992.1|
putative molybdopterin MPT conver...   
54   2e-06

gi|18076267|emb|CAC82488.1|
molybdopterin synthase small subunit...   
54   2e-06

gi|72162768|ref|YP\_290425.1|
hypothetical protein Tfu\_2369 [Ther...   
54   2e-06

gi|63255028|gb|AAY36124.1|
Molybdopterin converting factor, subu...   
54   2e-06

gi|68541239|ref|ZP\_00580985.1|
Molybdopterin converting factor, ...   
54   2e-06

gi|83941421|ref|ZP\_00953883.1|
putative molybdopterin MPT conver...   
54   2e-06

gi|88800262|ref|ZP\_01115829.1|
molybdopterin biosynthesis protei...   
54   2e-06

gi|77967366|gb|ABB08746.1|
Molybdopterin converting factor, subu...   
54   2e-06

gi|34483098|emb|CAE10097.1|
hypothetical protein [Wolinella succ...   
54   2e-06

gi|13883249|gb|AAK47766.1|
molybdopterin cofactor biosynthesis p...   
54   2e-06

gi|15025974|gb|AAK80866.1|
Uncharacterized protein, possibly inv...   
54   2e-06

gi|14027318|dbj|BAB54272.1|
molybdopterin converting factor, sub...   
54   2e-06

gi|83594146|ref|YP\_427898.1|
Molybdopterin converting factor, su...   
54   2e-06

gi|88793099|ref|ZP\_01108816.1|
molybdopterin converting factor, ...   
54   2e-06

gi|88809412|ref|ZP\_01124920.1|
molydbenum cofactor biosynthesis ...   
54   2e-06

gi|89070177|ref|ZP\_01157505.1|
putative molybdopterin MPT conver...   
54   2e-06

gi|99078603|ref|YP\_611861.1|
molybdopterin converting factor, su...   
54   2e-06

gi|33286618|gb|AAQ01711.1|
PdtH [Pseudomonas putida]                  
54   2e-06

gi|83720736|ref|YP\_442722.1|
molybdopterin converting factor, su...   
54   2e-06

gi|57241971|ref|ZP\_00369911.1|
thiamine biosynthesis protein Thi...   
54   2e-06

gi|13880985|gb|AAK45641.1|
conserved hypothetical protein [Mycob...   
54   2e-06

gi|75189826|ref|ZP\_00703093.1|
COG1977: Molybdopterin converting...   
54   2e-06

gi|1787002|gb|AAC73871.1|
molybdopterin synthase, small subunit ...   
53   2e-06

gi|1185170|emb|CAA91653.1| ORF73, homologous to Porphyra ORF71 [...    53  
2e-06

gi|32039081|ref|ZP\_00137353.1|
COG1977: Molybdopterin converting...   
53   2e-06

gi|93005056|ref|YP\_579493.1|
thiamine biosynthesis protein ThiS ...   
53   2e-06

gi|68562526|ref|ZP\_00601781.1|
Molybdopterin converting factor, ...    53  
2e-06

gi|30749759|pdb|1NVI|D
Chain D, Orthorhombic Crystal Form Of Mol...   
53   3e-06

gi|20804221|emb|CAD31247.1|
PROBABLE THIAMIN BIOSYNTHESIS PROTEI...   
53   3e-06

gi|46198624|ref|YP\_004291.1|
putative thiS protein [Thermus ther...   
53   3e-06

gi|75236193|ref|ZP\_00720310.1|
COG1977: Molybdopterin converting...   
53   3e-06

gi|9950101|gb|AAG07304.1|
molybdopterin converting factor, small...   
53   3e-06

gi|84716935|ref|ZP\_01023301.1|
hypothetical protein PnapDRAFT\_04...   
53   3e-06

gi|107028970|ref|YP\_626065.1|
molybdopterin converting factor, s...   
53   3e-06

gi|47551893|gb|AAT32729.2|
molybdopterin converting factor, subu...   
53   3e-06

gi|83369898|ref|ZP\_00914747.1|
Molybdopterin converting factor, ...   
53   3e-06

gi|94415786|ref|ZP\_01295621.1|
hypothetical protein PaerP\_010023...   
53   3e-06

gi|89073364|ref|ZP\_01159888.1|
hypothetical protein SKA34\_20482 ...   
53   3e-06

gi|94310901|ref|YP\_584111.1|
molybdopterin converting factor, su...   
53   3e-06

gi|90587945|ref|ZP\_01243597.1|
ThiS, thiamine-biosynthesis [Flav...   
53   3e-06

gi|49082512|gb|AAT50656.1|
PA3917 [synthetic construct]               
53   3e-06

gi|52307130|gb|AAU37630.1|
MoaD protein [Mannheimia succinicipro...   
53   3e-06

gi|42011|emb|CAA49864.1|
moaD [Escherichia coli]                      
53   3e-06

gi|71366890|ref|ZP\_00657425.1|
ThiamineS [Nocardioides sp. JS614...   
53   3e-06

gi|18159566|gb|AAL62982.1| molybdenum cofactor biosynthesis prot...    53   4e-06

gi|83748716|ref|ZP\_00945732.1|
ThiS family protein [Ralstonia so...   
53   4e-06

gi|78362790|gb|ABB40755.1|
molybdopterin converting factor, subu...   
53   4e-06

gi|56315292|emb|CAI09937.1|
Molybdopterin (MPT) converting facto...   
53   4e-06

gi|84319278|ref|ZP\_00967677.1|
COG0315: Molybdenum cofactor bios...   
53   4e-06

gi|28192390|gb|AAL65287.1|
QbsE [Pseudomonas fluorescens]             
53   4e-06

gi|86739580|ref|YP\_479980.1|
thiamine S [Frankia sp. CcI3] >gi|8...   
53   4e-06

gi|90579138|ref|ZP\_01234948.1|
hypothetical protein VAS14\_05513 ...   
53   4e-06

gi|89093577|ref|ZP\_01166525.1|
molybdopterin converting factor, ...   
53   4e-06

gi|33152460|ref|NP\_873813.1|
molybdopterin converting factor sub...   
53   4e-06

gi|45435691|gb|AAS61249.1|
molybdopterin [mpt] converting factor...  
 53   4e-06

gi|71037770|gb|AAZ18078.1|
possible sulfur transfer protein invo...   
53   4e-06

gi|72117326|gb|AAZ59589.1|
ThiS, thiamine-biosynthesis [Ralstoni...   
53   4e-06

gi|71145353|gb|AAZ25826.1|
thiamine biosynthesis protein ThiS [C...   
53   4e-06

gi|52427038|gb|AAU47631.1|
molybdopterin converting factor, subu...   
53   5e-06

gi|21221358|ref|NP\_627137.1|
hypothetical protein SCO2911 [Strep...   
53   5e-06

gi|70731087|ref|YP\_260828.1|
molybdopterin converting factor, su...   
53   5e-06

gi|72394552|gb|AAZ68829.1|
putative ThiS sulfur transfer protein...   
52   5e-06

gi|23023750|ref|ZP\_00062981.1|
COG2104: Sulfur transfer protein ...   
52   6e-06

gi|86154746|ref|ZP\_01072913.1|
conserved domain protein [Campylo...   
52   6e-06

gi|28851697|gb|AAO54773.1|
molybdenum cofactor biosynthesis prot...   
52   6e-06

gi|77996211|gb|ABB15110.1|
molybdopterin converting factor, subu...   
52   6e-06

gi|78691076|ref|ZP\_00855707.1|
Molybdopterin converting factor, ...   
52   6e-06

gi|71555514|gb|AAZ34725.1|
molybdopterin converting factor, subu...   
52   6e-06

gi|24375928|ref|NP\_719971.1|
molybdenum cofactor biosynthesis pr...   
52   7e-06

gi|15074101|emb|CAC45748.1|
PROBABLE MOLYBDOPTERIN MPT CONVERTIN...   
52   7e-06

gi|86139074|ref|ZP\_01057645.1|
molybdopterin converting factor, ...   
52   7e-06

gi|29897221|gb|AAP10498.1|
Molybdopterin (MPT) converting factor...   
52   7e-06

gi|23493776|dbj|BAC18745.1|
hypothetical protein [Corynebacteriu...   
52   7e-06

gi|88196185|ref|YP\_501002.1|
molybdopterin converting factor, su...   
52   7e-06

gi|83854898|ref|ZP\_00948428.1|
molybdopterin converting factor, ...   
52   7e-06

gi|84354863|ref|ZP\_00979760.1|
COG1977: Molybdopterin converting...   
52   7e-06

gi|75210621|ref|ZP\_00710769.1|
COG1977: Molybdopterin converting...   
52   7e-06

gi|27362554|gb|AAO11408.1|
Molybdenum cofactor biosynthesis prot...   
52   8e-06

gi|49484485|ref|YP\_041709.1|
putative molybdopterin-synthase sma...   
52   8e-06

gi|88950646|ref|ZP\_01153220.1|
MoaD, archaeal [Methanosaeta ther...   
52   8e-06

gi|49612264|emb|CAG75714.1|
molybdopterin converting factor subu...   
52   8e-06

gi|84499780|ref|ZP\_00998068.1|
putative molybdopterin MPT conver...   
52   8e-06

gi|68058473|gb|AAX88726.1|
molybdopterin converting factor subun...  
 52   8e-06

gi|11933033|emb|CAC19353.1|
hypothetical protein [Streptomyces h...   
52   8e-06

gi|89359306|ref|ZP\_01197127.1|
Molybdopterin converting factor, ...   
52   9e-06

gi|18161603|gb|AAL64874.1|
conserved hypothetical protein [Pyrob...   
52   9e-06

gi|81300590|ref|YP\_400798.1|
hypothetical protein Synpcc7942\_178...   
52   9e-06

gi|21228746|ref|NP\_634668.1|
putative molybdopterin converting f...   
52   9e-06

gi|4457226|gb|AAD21202.1|
MoaD [Rhodobacter capsulatus]               
52   9e-06

gi|72160631|ref|YP\_288288.1|
hypothetical protein Tfu\_0227 [Ther...   
52   9e-06

gi|2507066|sp|P45309|MOAD\_HAEIN
Molybdopterin-converting factor ...   
52   1e-05

gi|78700231|ref|ZP\_00864688.1|
hypothetical protein MlgDRAFT\_272...   
52   1e-05

gi|75762421|ref|ZP\_00742290.1|
Molybdopterin converting factor, ...   
52   1e-05

gi|76801893|ref|YP\_326901.1|
probable molybdopterin converting f...   
52   1e-05

gi|72002530|gb|AAZ58332.1|
ThiS, thiamine-biosynthesis [Prochlor...   
52   1e-05

gi|42631902|ref|ZP\_00157440.1|
COG1977: Molybdopterin converting...   
52   1e-05

gi|94501723|ref|ZP\_01308237.1|
molybdopterin biosynthesis [Ocean...   
51   1e-05

gi|82751858|ref|YP\_417599.1|
molybdopterin converting factor sma...   
51   1e-05

gi|67927210|ref|ZP\_00520404.1|
Molybdopterin biosynthesis MoaE:T...   
51   1e-05

gi|86608912|ref|YP\_477674.1|
molybdopterin converting factor, su...   
51   1e-05

gi|68231908|ref|ZP\_00571067.1|
ThiamineS [Frankia sp. EAN1pec] >...   
51   1e-05

gi|83952487|ref|ZP\_00961218.1|
molybdopterin converting factor, ...   
51   1e-05

gi|78170141|gb|ABB27238.1|
molydbenum cofactor biosynthesis prot...   
51   1e-05

gi|84713090|ref|ZP\_01020876.1|
Molybdopterin converting factor, ...   
51   1e-05

gi|13879933|gb|AAK44653.1|
conserved hypothetical protein [Mycob...   
51   1e-05

gi|103486643|ref|YP\_616204.1|
molybdopterin converting factor, s...   
51   1e-05

gi|84685238|ref|ZP\_01013137.1|
molybdopterin converting factor, ...   
51   1e-05

gi|83372471|ref|ZP\_00917251.1|
Molybdopterin converting factor, ...   
51   1e-05

gi|13883002|gb|AAK47534.1|
molybdenum cofactor biosynthesis prot...   
51   1e-05

gi|77960819|ref|ZP\_00824674.1|
COG1977: Molybdopterin converting...   
51   2e-05

gi|94309109|ref|YP\_582319.1|
thiamine biosynthesis protein ThiS ...   
51   2e-05

gi|77389071|gb|ABA80256.1|
putative molybdopterin MPT converting...   
51   2e-05

gi|54014565|dbj|BAD55935.1|
hypothetical protein [Nocardia farci...   
51   2e-05

gi|75431111|ref|ZP\_00732938.1|
molybdopterin (MPT) converting fa...    51  
2e-05

gi|87303475|ref|ZP\_01086258.1|
hypothetical protein WH5701\_09450...   
51   2e-05

gi|77957352|ref|ZP\_00821410.1|
COG1977: Molybdopterin converting...   
51   2e-05

gi|42629022|ref|ZP\_00154572.1|
COG1977: Molybdopterin converting...   
50   2e-05

gi|46912747|emb|CAG19537.1|
hypothetical protein [Photobacterium...   
50   2e-05

gi|91771392|ref|ZP\_01273196.1|
ThiS, thiamine-biosynthesis [Psyc...   
50   2e-05

gi|56680191|gb|AAV96857.1|
molybdopterin converting factor, subu...   
50   2e-05

gi|77973923|ref|ZP\_00829467.1|
COG1977: Molybdopterin converting...   
50   2e-05

gi|51858004|dbj|BAD42162.1|
molybdopterin converting factor-like...   
50   2e-05

gi|57638416|gb|AAW55204.1|
molybdopterin converting factor, subu...   
50   3e-05

gi|36784880|emb|CAE13794.1|
molybdopterin [mpt] converting facto...   
50   3e-05

gi|70982492|ref|XP\_746774.1|
molybdopterin synthase small subuni...   
50   3e-05

gi|91788067|ref|YP\_549019.1|
thiamineS [Polaromonas sp. JS666] >...   
50   3e-05

gi|91204446|emb|CAJ70946.1|
similar to molybdopterin synthase su...   
50   3e-05

gi|51857712|dbj|BAD41870.1|
molybdopterin converting factor smal...   
50   4e-05

gi|69936172|ref|ZP\_00631032.1|
Molybdopterin converting factor, ...   
50   4e-05

gi|71369077|ref|ZP\_00659547.1|
ThiamineS [Nocardioides sp. JS614...   
50   4e-05

gi|82702099|ref|YP\_411665.1|
ThiamineS [Nitrosospira multiformis...   
50   4e-05

gi|77976770|ref|ZP\_00832242.1|
COG1977: Molybdopterin converting...   
50   4e-05

gi|32033743|ref|ZP\_00134038.1|
COG1977: Molybdopterin converting...   
50   4e-05

gi|57167344|gb|AAW36123.1|
thiS family protein [Campylobacter je...   
50   4e-05

gi|88946143|ref|ZP\_01149231.1|
conserved hypothetical protein [D...   
50   4e-05

gi|4262375|gb|AAD14600.1|
molybdopterin-synthase small subunit [...   
49   4e-05

gi|74317044|ref|YP\_314784.1|
molybdopterin converting factor, su...   
49   5e-05

gi|83589575|ref|YP\_429584.1|
MoaD [Moorella thermoacetica ATCC 3...   
49   5e-05

gi|67925013|ref|ZP\_00518396.1|
ThiamineS [Crocosphaera watsonii ...   
49   5e-05

gi|55231494|gb|AAV46913.1|
molybdopterin converting factor subun...   
49   5e-05

gi|14325024|dbj|BAB59950.1|
molybdopterin converting factor subu...   
49   5e-05

gi|88934468|ref|ZP\_01140111.1|
conserved hypothetical protein [G...    49  
5e-05

gi|17428346|emb|CAD15033.1|
probable molybdopterin mpt convertin...   
49   5e-05

gi|68446507|dbj|BAE04091.1|
moaD [Staphylococcus haemolyticus JC...   
49   5e-05

gi|88935576|ref|ZP\_01141206.1|
similar to molybdopterin converti...    49   5e-05

gi|50909475|ref|XP\_466226.1| unknown protein [Oryza sativa (japo...    49  
6e-05

gi|5458384|emb|CAB49872.1|
moaD-like molybdopterin converting fa...   
49   6e-05

gi|83748544|ref|ZP\_00945564.1|
Molybdopterin converting factor, ...   
49   6e-05

gi|6968941|emb|CAB73937.1|
possible molybdopterin converting fac...   
49   6e-05

gi|21222687|ref|NP\_628466.1|
hypothetical protein SCO4294 [Strep...   
49   6e-05

gi|14324783|dbj|BAB59710.1|
molybdopterin converting factor subu...   
49   6e-05

gi|71676256|ref|ZP\_00673998.1|
ThiamineS [Trichodesmium erythrae...   
49   6e-05

gi|15618715|ref|NP\_225001.1|
threonyl-tRNA synthetase [Chlamydop...   
49   7e-05

gi|29831706|ref|NP\_826340.1|
hypothetical protein SAV5163 [Strep...   
49   7e-05

gi|90591660|ref|ZP\_01247301.1|
ThiamineS [Flavobacterium johnson...   
49   7e-05

gi|86153122|ref|ZP\_01071327.1|
conserved domain protein [Campylo...   
49   7e-05

gi|58001333|gb|AAW60227.1|
Bifunctional molybdenum cofactor bios...   
49   7e-05

gi|35214941|dbj|BAC92307.1|
gsr4366 [Gloeobacter violaceus PCC 7...   
48   8e-05

gi|62148593|emb|CAH64365.1|
threonyl-tRNA synthetase [Chlamydoph...   
48   8e-05

gi|91770046|ref|ZP\_01271876.1|
Molybdopterin converting factor, ...   
48   8e-05

gi|93005538|ref|YP\_579975.1|
thiamineS [Psychrobacter cryohalole...   
48   9e-05

gi|91783438|ref|YP\_558644.1|
Molybdopterin converting factor, su...   
48   9e-05

gi|78366785|ref|ZP\_00837062.1|
Molybdopterin converting factor, ...   
48   9e-05

gi|29835062|gb|AAP05696.1|
threonyl-tRNA synthetase [Chlamydophi...   
48   1e-04

gi|106883354|ref|ZP\_01350752.1|
ThiamineS [Psychromonas ingraham...   
48   1e-04

gi|77918070|ref|YP\_355885.1|
molybdopterin converting factor, su...   
48   1e-04

gi|86357115|ref|YP\_469007.1|
molybdopterin converting factor sub...   
48   1e-04

gi|91220231|ref|ZP\_01256641.1|
hypothetical protein P700755\_3102...   
48   1e-04

gi|34483282|emb|CAE10280.1|
hypothetical protein [Wolinella succ...   
48   1e-04

gi|77917869|ref|YP\_355684.1|
hypothetical protein Pcar\_0253 [Pel...    48  
1e-04

gi|15607139|ref|NP\_213755.1|
Molybdopterin converting factor, sm...   
48   1e-04

gi|19915596|gb|AAM05120.1|
predicted protein [Methanosarcina ace...   
48   1e-04

gi|11499216|ref|NP\_070453.1|
molybdopterin converting factor, su...   
48   1e-04

gi|56379152|dbj|BAD75060.1|
molybdopterin converting factor (sub...   
48   1e-04

gi|75855800|ref|ZP\_00763441.1|
COG1977: Molybdopterin converting...   
48   1e-04

gi|85117520|ref|XP\_965278.1|
hypothetical protein [Neurospora cr...   
48   2e-04

gi|56314521|emb|CAI09166.1|
putative tungsten-containing aldehyd...   
48   2e-04

gi|88601825|ref|YP\_502003.1|
thiamineS [Methanospirillum hungate...   
47   2e-04

gi|32261593|gb|AAP76643.1|
molybdopterin converting factor [Heli...   
47   2e-04

gi|3955207|gb|AAC83142.1|
MoaD [Staphylococcus carnosus]              
47   2e-04

gi|59711549|ref|YP\_204325.1|
molybdopterin converting factor, sm...   
47   2e-04

gi|86149491|ref|ZP\_01067722.1|
thiS family protein [Campylobacte...   
47   2e-04

gi|34482542|emb|CAE09542.1|
hypothetical protein [Wolinella succ...   
47   2e-04

gi|88938982|ref|ZP\_01144434.1|
molybdopterin converting factor, ...   
47   2e-04

gi|83590498|ref|YP\_430507.1|
thiamine biosynthesis protein ThiS ...   
47   2e-04

gi|86147951|ref|ZP\_01066255.1|
Molybdenum cofactor biosynthesis ...   
47   2e-04

gi|91220879|ref|ZP\_01257071.1|
putative molybdopterin MPT conver...   
47   2e-04

gi|40063023|gb|AAR37879.1|
molybdenum cofactor biosynthesis prot...   
47   2e-04

gi|33867003|ref|NP\_898562.1|
molydbenum cofactor biosynthesis pr...   
47   2e-04

gi|68553535|ref|ZP\_00592907.1|
ThiamineS [Prosthecochloris aestu...   
47   2e-04

gi|33359354|ref|NP\_877809.1|
putative molybdopterin converting f...   
47   2e-04

gi|10640172|emb|CAC12024.1|
conserved hypothetical protein [Ther...   
47   2e-04

gi|28807086|dbj|BAC60356.1|
molybdenum cofactor biosynthesis pro...   
47   2e-04

gi|77919319|ref|YP\_357134.1|
molybdopterin converting factor, su...   
47   2e-04

gi|66811416|ref|XP\_639888.1|
hypothetical protein DDB0186363 [Di...   
47   2e-04

gi|87301504|ref|ZP\_01084344.1|
molydbenum cofactor biosynthesis ...    47   2e-04

gi|15156160|gb|AAK86930.1| AGR\_C\_2086p [Agrobacterium tumefacien...    47  
2e-04

gi|95930902|ref|ZP\_01313632.1| thiamineS [Desulfuromonas acetoxi...    47  
2e-04

gi|83645616|ref|YP\_434051.1| Molybdopterin converting factor, sm...    47  
2e-04

gi|84393380|ref|ZP\_00992139.1| Molybdenum cofactor biosynthesis ...    47  
3e-04

gi|77382376|gb|ABA73889.1| ThiamineS [Pseudomonas fluorescens Pf...    47  
3e-04

gi|17739525|gb|AAL42140.1| molybdopterin converting factor small...    47  
3e-04

gi|46108760|ref|XP\_381438.1| hypothetical protein FG01262.1 [Gib...    47  
3e-04

gi|47573807|ref|ZP\_00243844.1| COG1977: Molybdopterin converting...    47  
3e-04

gi|76258732|ref|ZP\_00766386.1| ThiamineS [Chloroflexus aurantiac...    47  
3e-04

gi|55771533|dbj|BAD69974.1| molybdopterin converting factor, sub...    47  
3e-04

gi|22295085|dbj|BAC08913.1| molybdopterin biosynthesis protein D...    47  
3e-04

gi|42543908|pdb|1V8C|D
Chain D, Crystal Structure Of Moad Relate...   
47   3e-04

gi|91227353|ref|ZP\_01261742.1|
molybdenum cofactor biosynthesis ...   
47   3e-04

gi|46200137|ref|YP\_005804.1|
molybdopterin converting factor, sm...   
47   3e-04

gi|91797290|gb|ABE59429.1|
molybdenum cofactor biosynthesis prot...   
46   4e-04

gi|76167848|gb|AAX50856.1|
threonyl-tRNA synthetase [Chlamydia t...   
46   4e-04

gi|7437527|pir||G71497 threonine-tRNA ligase (EC 6.1.1.3) - Chla...    46  
4e-04

gi|39982776|gb|AAR34235.1|
moaD family protein [Geobacter sulfur...   
46   4e-04

gi|71838567|ref|ZP\_00678326.1|
ThiamineS [Pelobacter propionicus...   
46   4e-04

gi|83026442|gb|ABB96257.1|
VP15 [Zea mays]                            
46   5e-04

gi|89100759|ref|ZP\_01173613.1|
molybdopterin converting factor, ...   
46   5e-04

gi|40062746|gb|AAR37640.1|
molybdopterin converting factor, subu...   
46   5e-04

gi|78702657|ref|ZP\_00867087.1|
conserved hypothetical protein [A...   
46   5e-04

gi|88801656|ref|ZP\_01117184.1|
putative molybdopterin-synthase s...   
46   6e-04

gi|73661952|ref|YP\_300733.1|
molybdopterin converting factor sma...   
46   6e-04

gi|18160982|gb|AAL64296.1|
conserved hypothetical protein [Pyrob...   
46   6e-04

gi|35213985|dbj|BAC91354.1|
gsl3413 [Gloeobacter violaceus PCC 7...   
45   6e-04

gi|19712997|gb|AAL93871.1|
ThiS protein [Fusobacterium nucleatum...   
45   6e-04

gi|33359535|ref|NP\_578790.2|
sulfur carrier protein ThiS [Pyroco...   
45   7e-04

gi|48425680|pdb|1SF0|A
Chain A, Backbone Solution Structure Of M...   
45   7e-04

gi|34763552|ref|ZP\_00144489.1|
ThiS protein [Fusobacterium nucle...   
45   7e-04

gi|71492602|gb|EAO24906.1|
ThiamineS [Syntrophomonas wolfei subs...   
45   7e-04

gi|26988028|ref|NP\_743453.1|
molybdenum cofactor biosynthesis pr...    45  
7e-04

gi|6226383|sp|O29699|Y552\_ARCFU
Hypothetical UPF0084 protein AF0...   
45   8e-04

gi|18893126|gb|AAL81185.1|
hypothetical protein [Pyrococcus furi...   
45   8e-04

gi|50877366|emb|CAG37206.1|
similar to molybdopterin converting ...   
45   8e-04

gi|89897864|ref|YP\_514974.1|
threoninyl tRNA synthetase [Chlamyd...   
45   8e-04

gi|88931718|ref|ZP\_01137411.1|
Threonyl-tRNA synthetase, class I...   
45   8e-04

gi|88934592|ref|ZP\_01140234.1|
conserved hypothetical protein [G...   
45   9e-04

gi|71842263|ref|YP\_277351.1|
hypothetical chloroplast RF40 [Emil...   
45   9e-04

gi|82737924|ref|ZP\_00900767.1|
Molybdopterin converting factor, ...   
45   9e-04

gi|77997120|gb|ABB16019.1|
conserved hypothetical protein [Carbo...   
45   9e-04

gi|91791472|ref|YP\_561123.1|
molybdopterin converting factor, su...   
45   9e-04

gi|86143258|ref|ZP\_01061660.1|
hypothetical protein MED217\_08750...   
45   9e-04

gi|71143753|gb|AAZ24226.1|
molybdopterin converting factor, subu...   
45   0.001

gi|28631173|ref|NP\_789776.1|
molybdopterin synthase small subuni...   
45   0.001

gi|88703397|ref|ZP\_01101113.1|
Molybdopterin converting factor s...   
45   0.001

gi|95929924|ref|ZP\_01312664.1|
thiamineS [Desulfuromonas acetoxi...   
45   0.001

gi|71038167|gb|AAZ18475.1|
probable molybdopterin converting fac...   
45   0.001

gi|72395205|gb|AAZ69478.1|
molybdopterin converting factor small...   
45   0.001

gi|48430776|gb|AAT43641.1|
molybdopterin (MPT) converting factor...   
45   0.001

gi|78219906|gb|ABB39255.1|
conserved hypothetical protein [Desul...   
45   0.001

gi|23465302|ref|NP\_695905.1|
threonyl-tRNA synthetase [Bifidobac...   
44   0.001

gi|75701250|gb|ABA20926.1|
ThiamineS [Anabaena variabilis ATCC 2...   
44   0.001

gi|57505249|ref|ZP\_00371178.1|
moaD2 [Campylobacter upsaliensis ...   
44   0.001

gi|74018017|ref|ZP\_00688639.1|
ThiamineS [Burkholderia ambifaria...   
44   0.001

gi|46191233|ref|ZP\_00120366.2|
COG0441: Threonyl-tRNA synthetase...   
44   0.001

gi|76791187|ref|ZP\_00773695.1|
ThiamineS [Pseudoalteromonas atla...   
44   0.002

gi|15645420|ref|NP\_207594.1|
molybdopterin converting factor, su...   
44   0.002

gi|7190897|gb|AAF39666.1|
threonyl-tRNA synthetase [Chlamydia mu...   
44   0.002

gi|77995330|gb|ABB14229.1|
thiamine biosynthesis protein ThiS [C...  
 44   0.002

gi|46449006|gb|AAS95658.1|
hypothetical protein DVU\_1180 [Desulf...   
44   0.002

gi|89201285|ref|ZP\_01180030.1|
Molybdopterin converting factor, ...   
44   0.002

gi|23126667|ref|ZP\_00108556.1|
COG1977: Molybdopterin converting...   
44   0.002

gi|67932283|ref|ZP\_00525430.1|
ThiamineS [Solibacter usitatus El...   
44   0.002

gi|69951944|ref|ZP\_00639556.1|
Molybdopterin converting factor, ...   
44   0.002

gi|86159912|ref|YP\_466697.1|
thiamineS [Anaeromyxobacter dehalog...   
44   0.002

gi|57240883|ref|ZP\_00368831.1|
molybdopterin converting factor, ...   
43   0.003

gi|58580685|ref|YP\_199701.1|
molybdopterin-converting factor cha...   
43   0.003

gi|33356745|ref|NP\_126807.2|
sulfur carrier protein ThiS [Pyroco...   
43   0.003

gi|21226933|ref|NP\_632855.1|
Molybdopterin converting factor sma...   
43   0.003

gi|89900702|ref|YP\_523173.1|
molybdopterin converting factor, su...   
43   0.003

gi|89206760|ref|ZP\_01185313.1|
Molybdopterin converting factor, ...   
43   0.003

gi|17133489|dbj|BAB76052.1|
asl4353 [Nostoc sp. PCC 7120] >gi|17...   
43   0.003

gi|72122148|gb|AAZ64334.1|
ThiamineS [Ralstonia eutropha JMP134]...   
43   0.003

gi|90303795|gb|EAS33426.1|
hypothetical protein CIMG\_04450 [Cocc...   
43   0.004

gi|68193704|gb|EAN08356.1|
Molybdopterin converting factor, subu...   
43   0.004

gi|98660190|dbj|GAA02501.1|
unnamed protein product [Pelotomacul...   
43   0.004

gi|67937987|ref|ZP\_00530517.1|
ThiamineS [Chlorobium phaeobacter...   
43   0.004

gi|98662976|dbj|GAA00218.1|
unnamed protein product [Pelotomacul...   
43   0.004

gi|78496994|gb|ABB43534.1|
Thiamine S [Thiomicrospira denitrific...   
43   0.005

gi|86134373|ref|ZP\_01052955.1|
putative molybdopterin-synthase s...   
43   0.005

gi|76795290|ref|ZP\_00777663.1|
ThiamineS [Thermoanaerobacter eth...   
43   0.005

gi|28271030|emb|CAD63935.1|
molybdopterin biosynthesis protein, ...   
43   0.005

gi|106891734|ref|ZP\_01358916.1|
ThiamineS [Roseiflexus sp. RS-1]...   
43   0.005

gi|20517475|gb|AAM25599.1|
hypothetical protein TTE2469 [Thermoa...   
43   0.005

gi|77381195|gb|ABA72708.1|
MoaD [Pseudomonas fluorescens PfO-1] ...   
43   0.005

gi|4155307|gb|AAD06323.1|
putative MOLYBDOPTERIN CONVERTING FACT...   
43   0.005

gi|83648509|ref|YP\_436944.1|
Molybdopterin converting factor, sm...   
42   0.005

gi|88946144|ref|ZP\_01149232.1|
conserved hypothetical protein [D...   
42   0.005

gi|67987927|gb|EAM75712.1|
Threonyl-tRNA synthetase, class IIa [...   
42   0.006

gi|30681325|ref|NP\_849354.1|
CNX7/SIR5; catalytic [Arabidopsis t...    42  
0.006

gi|72162507|ref|YP\_290164.1|
threonyl-tRNA synthetase [Thermobif...   
42   0.006

gi|13880442|gb|AAK45132.1|
conserved hypothetical protein [Mycob...   
42   0.006

gi|77964628|gb|ABB06009.1|
Thiamine S [Burkholderia sp. 383] >gi...   
42   0.007

gi|2633802|emb|CAB13304.1|
molybdopterin converting factor (subu...   
42   0.007

gi|76803138|ref|YP\_331233.1|
probable molybdopterin converting f...   
42   0.007

gi|88934227|ref|ZP\_01139871.1|
Threonyl-tRNA synthetase, class I...   
42   0.007

gi|90416705|ref|ZP\_01224635.1|
molybdenum cofactor biosynthesis ...   
42   0.007

gi|88712833|ref|ZP\_01106918.1|
hypothetical protein FB2170\_09351...   
42   0.007

gi|23395272|gb|AAN31771.1|
molybdopterin converting factor, smal...   
42   0.008

gi|67904312|ref|XP\_682412.1|
hypothetical protein AN9143.2 [Aspe...   
42   0.008

gi|67467174|ref|XP\_649707.1|
hypothetical protein 277.t00008 [En...   
42   0.008

gi|67664217|ref|ZP\_00461493.1|
ThiamineS [Burkholderia cenocepac...   
42   0.008

gi|18145265|dbj|BAB81308.1|
probable molybdopterin biosynthesis ...   
42   0.009

gi|10581293|gb|AAG20053.1|
Vng1848h [Halobacterium sp. NRC-1] >g...   
42   0.009

gi|10175640|dbj|BAB06737.1|
molybdopterin converting factor (sub...   
42   0.009

gi|98661971|dbj|GAA01064.1|
unnamed protein product [Pelotomacul...   
42   0.009

gi|78700359|ref|ZP\_00864816.1|
Rhodanese-like [Alkalilimnicola e...   
42   0.009

gi|39964901|ref|XP\_365048.1|
hypothetical protein MG09893.4 [Mag...   
42   0.009

gi|47502579|gb|AAT31255.1|
molybdopterin converting factor, subu...   
42   0.009

gi|39983496|gb|AAR34889.1|
threonyl-tRNA synthetase [Geobacter s...   
42   0.009

gi|75765030|ref|ZP\_00744342.1|
Molybdopterin converting factor, ...   
42   0.010

gi|67154055|ref|ZP\_00415800.1|
Protein of unknown function DUF82...   
42   0.010

gi|66963966|ref|ZP\_00411537.1|
ThiamineS [Arthrobacter sp. FB24]...   
42   0.010

gi|82498768|ref|ZP\_00884224.1|
conserved hypothetical protein [C...   
42   0.011

gi|84489151|ref|YP\_447383.1|
hypothetical protein Msp\_0330 [Meth...   
42   0.011

gi|32444387|emb|CAD74386.1|
probable molybdopterin converting fa...   
42   0.011

gi|51974253|gb|AAU15803.1|
molybdopterin converting factor, subu...   
41   0.013

gi|71839361|ref|ZP\_00679111.1|
Threonyl-tRNA synthetase, class I...    41  
0.013

gi|75760851|ref|ZP\_00740866.1|
Molybdopterin converting factor, ...   
41   0.013

gi|83816286|ref|YP\_446147.1|
ThiS family, putative [Salinibacter...   
41   0.014

gi|85667142|ref|ZP\_01029364.1|
hypothetical protein Badol\_010006...   
41   0.014

gi|21107237|gb|AAM35973.1|
molybdopterin-converting factor chain...   
41   0.015

gi|78046664|ref|YP\_362839.1|
molybdopterin-converting factor cha...   
41   0.015

gi|29898350|gb|AAP11623.1|
Molybdopterin (MPT) converting factor...   
41   0.016

gi|91785509|ref|YP\_560715.1|
hypothetical protein Bxe\_A0270 [Bur...   
41   0.016

gi|76579340|gb|ABA48815.1|
TGS domain protein [Burkholderia pseu...   
41   0.016

gi|21112021|gb|AAM40299.1|
molybdopterin-converting factor chain...   
41   0.017

gi|68140833|gb|EAM94128.1|
MoaD, archaeal [Ferroplasma acidarman...   
41   0.017

gi|15025973|gb|AAK80865.1|
Dinucleotide-utilizing enzyme involve...   
41   0.018

gi|47566905|ref|ZP\_00237623.1|
molybdopterin converting factor, ...   
41   0.018

gi|88805648|ref|ZP\_01121167.1|
hypothetical protein RB2501\_14949...   
41   0.018

gi|92089022|ref|ZP\_01273976.1|
ThiamineS [Lactobacillus reuteri ...   
41   0.018

gi|89204215|ref|ZP\_01182793.1|
Molybdopterin converting factor, ...   
41   0.019

gi|49333106|gb|AAT63752.1|
molybdopterin biosynthesis protein, s...   
41   0.019

gi|84705083|ref|ZP\_01018583.1|
molybdopterin converting factor, ...   
41   0.019

gi|42557747|emb|CAF28721.1|
putative molybdopterin biosynthesis ...   
41   0.019

gi|84496326|ref|ZP\_00995180.1|
threonyl-tRNA synthetase [Janibac...   
41   0.020

gi|42783907|ref|NP\_981154.1|
molybdopterin converting factor, su...   
40   0.020

gi|41406904|ref|NP\_959740.1|
MoaD2 [Mycobacterium avium subsp. p...   
40   0.020

gi|49330166|gb|AAT60812.1|
molybdopterin converting factor, subu...   
40   0.020

gi|10581690|gb|AAG20393.1|
Vng2279h [Halobacterium sp. NRC-1] >g...   
40   0.020

gi|71544347|ref|ZP\_00665385.1|
ThiamineS [Syntrophobacter fumaro...   
40   0.021

gi|51976766|gb|AAU18316.1|
molybdopterin biosynthesis protein, s...   
40   0.021

gi|56908910|dbj|BAD63437.1|
molybdopterin converting factor subu...   
40   0.023

gi|47505417|gb|AAT34093.1|
molybdopterin converting factor, subu...   
40   0.023

gi|14591747|ref|NP\_143011.1|
sulfur carrier protein ThiS [Pyroco...    40  
0.025

gi|78193878|gb|ABB31645.1|
Threonyl-tRNA synthetase, class IIa [...   
40   0.027

gi|68563154|ref|ZP\_00602356.1|
ThiamineS [Rubrobacter xylanophil...   
40   0.027

gi|29895812|gb|AAP09094.1|
Molybdopterin (MPT) converting factor...   
40   0.028

gi|95930037|ref|ZP\_01312777.1|
conserved hypothetical protein [D...   
40   0.029

gi|70728421|ref|YP\_258170.1|
molybdenum cofactor biosynthesis pr...   
40   0.029

gi|85373384|ref|YP\_457446.1|
hypothetical protein ELI\_02785 [Ery...   
40   0.030

gi|88938068|ref|ZP\_01143581.1|
conserved hypothetical protein [G...   
40   0.030

gi|86740565|ref|YP\_480965.1|
cytochrome P450 [Frankia sp. CcI3] ...   
40   0.030

gi|67154931|ref|ZP\_00416676.1|
ThiamineS [Azotobacter vinelandii...   
40   0.033

gi|58581648|ref|YP\_200664.1|
hypothetical protein XOO2025 [Xanth...   
40   0.033

gi|71837964|ref|ZP\_00677726.1|
conserved hypothetical protein [P...   
40   0.034

gi|74318176|ref|YP\_315916.1|
hypothetical protein Tbd\_2158 [Thio...   
40   0.034

gi|52429778|gb|AAU50371.1|
TGS domain protein [Burkholderia mall...   
40   0.035

gi|21322979|dbj|BAB97608.1|
Hypothetical protein [Corynebacteriu...   
40   0.037

gi|104783496|ref|YP\_609994.1|
molybdenum cofactor biosynthesis p...   
40   0.037

gi|83312026|ref|YP\_422290.1|
hypothetical protein amb2927 [Magne...   
40   0.038

gi|88186128|gb|EAQ93596.1|
hypothetical protein CHGG\_01831 [Chae...   
40   0.039

gi|19887264|gb|AAM02010.1|
Predicted cyclophilin type peptidyl-p...   
40   0.039

gi|25169121|emb|CAD47957.1|
putative molybdopterin cofactor synt...   
40   0.039

gi|21107692|gb|AAM36384.1|
conserved hypothetical protein [Xanth...   
40   0.040

gi|78047114|ref|YP\_363289.1|
hypothetical protein XCV1558 [Xanth...   
39   0.044

gi|77683437|ref|ZP\_00798886.1|
UBA/THIF-type NAD/FAD binding fol...   
39   0.052

gi|107024344|ref|YP\_622671.1|
protein of unknown function DUF82 ...   
39   0.052

gi|18893610|gb|AAL81606.1|
hypothetical protein [Pyrococcus furi...   
39   0.054

gi|57159352|dbj|BAD85282.1|
sulfur transfer protein involved in ...   
39   0.055

gi|44920975|emb|CAF30211.1|
hypothetical protein [Methanococcus ...   
39   0.059

gi|107022979|ref|YP\_621306.1|
thiamineS [Burkholderia cenocepaci...   
39   0.060

gi|77919190|ref|YP\_357005.1|
hypothetical protein Pcar\_1591 [Pel...   
39   0.063

gi|85707987|ref|ZP\_01039053.1|
hypothetical protein NAP1\_02090 [...   
39   0.066

gi|41324446|emb|CAF18786.1|
PUTATIVE MOLYBDOPTERIN CONVERTING FA...   
39   0.068

gi|13540947|ref|NP\_110635.1|
hypothetical protein TVN0116 [Therm...    39  
0.069

gi|84623563|ref|YP\_450935.1|
hypothetical protein XOO\_1906 [Xant...   
39   0.071

gi|67666690|ref|ZP\_00463933.1|
Protein of unknown function DUF82...   
39   0.073

gi|14324330|dbj|BAB59258.1|
hypothetical protein [Thermoplasma v...   
38   0.077

gi|92090350|ref|ZP\_01275216.1|
molybdopterin converting factor s...   
38   0.078

gi|52003241|gb|AAU23183.1|
molybdopterin converting factor (subu...   
38   0.13

gi|83773325|dbj|BAE63452.1|
unnamed protein product [Aspergillus...   
38   0.14

gi|86607092|ref|YP\_475855.1|
ThiS domain protein [Synechococcus ...   
38   0.14

gi|46401290|emb|CAF24739.1|
probable threonine-tRNA ligase [Para...    38  
0.16

gi|89902161|ref|YP\_524632.1|
thiamineS [Rhodoferax ferrireducens...   
38   0.16

gi|82747912|ref|ZP\_00910404.1|
Threonyl-tRNA synthetase, class I...   
37   0.18

gi|76796989|ref|ZP\_00779334.1|
Phosphoribulokinase/uridine kinas...   
37   0.18

gi|72117336|gb|AAZ59599.1|
Protein of unknown function DUF82 [Ra...   
37   0.19

gi|51588192|emb|CAH19799.1|
hypothetical protein [Yersinia pseud...   
37   0.19

gi|83368880|ref|ZP\_00913740.1|
protein RnfH [Rhodobacter sphaero...   
37   0.20

gi|15622711|dbj|BAB66701.1|
68aa long conserved hypothetical pro...   
37   0.21

gi|56312934|emb|CAI07579.1|
Protein rnfH [Azoarcus sp. EbN1] >gi...   
37   0.21

gi|82701281|ref|YP\_410847.1|
Protein of unknown function DUF82 [...   
37   0.22

gi|56311907|emb|CAI06552.1|
conserved hypothetical protein [Azoa...   
37   0.24

gi|86604898|ref|YP\_473661.1|
putative molybdopterin converting f...   
37   0.24

gi|87311482|ref|ZP\_01093601.1|
hypothetical protein DSM3645\_0176...   
37   0.26

gi|76801420|ref|YP\_326428.1|
pterin cluster protein [Natronomona...   
37   0.26

gi|68551819|ref|ZP\_00591213.1|
Threonyl-tRNA synthetase, class I...   
37   0.27

gi|14424008|sp|O30213|Y022\_ARCFU
Hypothetical protein AF0022 >gi...   
37   0.27

gi|99908378|ref|ZP\_01316082.1|
hypothetical protein Bpse1\_030045...   
37   0.29

gi|46201411|ref|ZP\_00055070.2|
hypothetical protein Magn03009730...   
37   0.30

gi|67738583|ref|ZP\_00489224.1|
COG1656: Uncharacterized conserve...   
37   0.32

gi|76580804|gb|ABA50279.1|
Protein of unknown function family [B...   
37   0.32

gi|87121811|ref|ZP\_01077697.1|
molybdenum cofactor biosynthesis ...   
37   0.33

gi|68129461|emb|CAJ08004.1|
ubiquitin related modifier (urm1)-li...   
37   0.36

gi|83719599|ref|YP\_442575.1|
TGS domain protein [Burkholderia th...   
36   0.38

gi|89343138|ref|ZP\_01195366.1|
ThiamineS [Mycobacterium flavesce...   
36   0.39

gi|91787516|ref|YP\_548468.1|
protein of unknown function DUF82 [...   
36   0.43

gi|52002559|gb|AAU22501.1|
threonyl-tRNA synthetase [Bacillus li...   
36   0.45

gi|21112537|gb|AAM40764.1|
conserved hypothetical protein [Xanth...   
36   0.45

gi|82737127|ref|ZP\_00899979.1|
conserved hypothetical protein [P...   
36   0.50

gi|89361078|ref|ZP\_01198894.1|
conserved hypothetical protein [X...   
36   0.50

gi|71546139|ref|ZP\_00666991.1|
conserved hypothetical protein [S...   
36   0.50

gi|51856879|dbj|BAD41037.1|
conserved hypothetical protein [Symb...   
36   0.53

gi|70607385|ref|YP\_256255.1|
hypothetical protein Saci\_1652 [Sul...   
36   0.53

gi|13421105|gb|AAK22005.1|
molybdopterin converting factor, subu...   
36   0.54

gi|50839587|gb|AAT82254.1|
putative molybdopterin converting fac...   
36   0.56

gi|86609522|ref|YP\_478284.1|
ThiS domain protein [Synechococcus ...   
36   0.57

gi|19918186|gb|AAM07434.1|
molybdopterin converting factor, subu...   
36   0.57

gi|2623002|gb|AAB86331.1|
conserved protein [Methanothermobacter...  
 36   0.58

gi|30248385|ref|NP\_840455.1|
spoT; bifunctional enzyme (p)ppgpp ...   
36   0.59

gi|92906590|ref|ZP\_01275370.1|
ThiamineS [Mycobacterium sp. JLS]...   
36   0.62

gi|55240673|gb|EAL40503.1|
ENSANGP00000025406 [Anopheles gambiae...   
35   0.70

gi|94309119|ref|YP\_582329.1|
protein of unknown function DUF82 [...   
35   0.70

gi|92914564|ref|ZP\_01283191.1|
ThiamineS [Mycobacterium sp. KMS]...   
35   0.70

gi|68231652|ref|ZP\_00570816.1|
ThiamineS [Frankia sp. EAN1pec] >...   
35   0.71

gi|2633570|emb|CAB13073.1|
yjgC [Bacillus subtilis subsp. subtil...   
35   0.72

gi|55231495|gb|AAV46914.1|
unknown [Haloarcula marismortui ATCC ...   
35   0.78

gi|88811647|ref|ZP\_01126901.1|
hypothetical protein NB231\_04560 ...   
35   0.79

gi|77389630|gb|ABA80814.1|
probable rnfH protein [Rhodobacter sp...   
35   0.79

gi|50876317|emb|CAG36157.1|
probable threonyl-tRNA synthetase [D...   
35   0.80

gi|72163135|ref|YP\_290792.1|
putative molybdopterin converting f...   
35   0.81

gi|71652006|ref|XP\_814668.1|
hypothetical protein [Trypanosoma c...   
35   0.82

gi|16410447|emb|CAC99123.1|
lmo1045 [Listeria monocytogenes] >gi...   
35   0.84

gi|87198948|ref|YP\_496205.1|
thiamineS [Novosphingobium aromatic...   
35   0.85

gi|88931571|ref|ZP\_01137265.1|
Ferredoxin--nitrite reductase [Ac...   
35   0.86

gi|16331655|ref|NP\_442383.1|
hypothetical protein ssr1527 [Synec...   
35   0.87

gi|94413394|ref|ZP\_01293279.1|
hypothetical protein PaerP\_010047...   
35   0.88

gi|19917335|gb|AAM06670.1|
predicted protein [Methanosarcina ace...   
35   0.92

gi|71416009|ref|XP\_810051.1|
hypothetical protein [Trypanosoma c...   
35   0.93

gi|88949024|ref|ZP\_01151642.1|
hypothetical protein HhalDRAFT\_10...   
35   0.99

gi|66967003|ref|ZP\_00414553.1|
Threonyl-tRNA synthetase, class I...   
35   0.99

gi|90201062|ref|ZP\_01203708.1|
ThiamineS [Mycobacterium vanbaale...   
35   1.0

gi|23492025|dbj|BAC17000.1|
putative molybdopterin biosynthesis ...   
35   1.0

gi|67928178|ref|ZP\_00521367.1|
hypothetical protein AcidDRAFT\_57...   
35   1.1

gi|67874585|ref|ZP\_00504295.1|
Threonyl-tRNA synthetase, class I...   
35   1.1

gi|51858105|dbj|BAD42263.1|
molybdopterin converting factor smal...   
35   1.1

gi|83719003|ref|YP\_443618.1|
Protein of unknown function family ...   
35   1.1

gi|21637142|gb|AAM70358.1|
CalU18 [Micromonospora echinospora]        
35   1.1

gi|10640784|emb|CAC12562.1|
hypothetical protein [Thermoplasma a...   
35   1.1

gi|59410400|gb|AAW84299.1|
putative MaoD-like protein [unculture...   
35   1.1

gi|33572311|emb|CAE41865.1|
guanosine-3',5'-bis(diphosphate) 3'-...   
35   1.2

gi|33576387|emb|CAE33465.1|
guanosine-3',5'-bis(diphosphate) 3'-...   
35   1.2

gi|76802608|ref|YP\_327616.1|
homolog to molybdopterin converting...   
35   1.3

gi|56378836|dbj|BAD74744.1|
formate dehydrogenase chain A [Geoba...   
35   1.3

gi|72398144|gb|AAZ72417.1|
conserved hypothetical protein [Metha...   
35   1.4

gi|47096132|ref|ZP\_00233732.1|
molybdenum cofactor biosynthesis ...   
35   1.4

gi|52348470|gb|AAU41104.1|
YjgC [Bacillus licheniformis DSM 13] ...   
34   1.5

gi|33565867|emb|CAE36414.1|
molybdopterin converting factor [Bor...   
34   1.5

gi|52003802|gb|AAU23744.1|
iron-sulfur binding domain containing...   
34   1.5

gi|82748786|ref|ZP\_00911263.1|
UBA/THIF-type NAD/FAD binding fol...   
34   1.5

gi|46907277|ref|YP\_013666.1| molybdenum cofactor biosynthesis pr...    34  
1.5

gi|82499276|ref|ZP\_00884724.1| hypothetical protein CsacDRAFT\_13...    34  
1.6

gi|71549789|ref|ZP\_00669971.1| RelA/SpoT protein [Nitrosomonas e...    34  
1.6

gi|77965627|gb|ABB07007.1| protein of unknown function DUF82 [Bu...    34  
1.6

gi|83372801|ref|ZP\_00917580.1| protein RnfH [Rhodobacter sphaero...    34  
1.6

gi|71491252|gb|EAO23583.1| Ferredoxin:4Fe-4S ferredoxin, iron-su...    34  
1.7

gi|70606737|ref|YP\_255607.1| molybdenum cofactor biosynthesis pr...    34  
1.7

gi|10581281|gb|AAG20043.1| threonyl-tRNA synthetase; ThrS [Halob...    34  
1.7

gi|21222559|ref|NP\_628338.1| molybdopterin converting factor [St...    34  
1.8

gi|67986515|gb|EAM74335.1| ThiamineS [Kineococcus radiotolerans ...    34  
1.8

gi|73921305|sp|Q9HP27|SYT\_HALSA Threonyl-tRNA synthetase (Threon...    34  
1.8

gi|6689424|emb|CAB65483.1| threonyl-tRNA synthetase [Thermus the...    34  
1.9

gi|89255717|ref|YP\_513078.1| GTP pyrophosphokinase [Francisella ...    34  
1.9

gi|46199818|ref|YP\_005485.1| threonyl-tRNA synthetase [Thermus t...    34  
1.9

gi|56605038|emb|CAG46141.1| GTP pyrophosphokinase [Francisella t...    34  
1.9

gi|29830583|ref|NP\_825217.1| molybdopterin converting factor [St...    34  
1.9

gi|62425453|ref|ZP\_00380587.1|
COG1977: Molybdopterin converting...   
34   2.1

gi|78221054|gb|ABB40403.1|
hypothetical protein Dde\_3610 [Desulf...   
34   2.2

gi|20429175|dbj|BAB91333.1|
chloroplast ppGpp synthase/degradase...   
34   2.2

gi|71837115|ref|ZP\_00676881.1|
UBA/THIF-type NAD/FAD binding fol...   
34   2.3

gi|9947297|gb|AAG04744.1|
hypothetical protein PA1355 [Pseudomon...   
33   2.4

gi|736275|emb|CAA56171.1|
unnamed protein product [Pyrococcus fu...   
33   2.5

gi|48430257|gb|AAT43122.1|
hypothetical protein PTO0537 [Picroph...   
33   2.6

gi|49077442|gb|AAT49679.1|
PA1355 [synthetic construct]               
33   2.6

gi|57103812|ref|XP\_542738.1|
PREDICTED: similar to Cytochrome P4...   
33   2.7

gi|46198413|ref|YP\_004080.1|
hypothetical protein TTC0105 [Therm...   
33   2.7

gi|91788153|ref|YP\_549105.1|
hypothetical protein Bpro\_2284 [Pol...   
33   2.7

gi|74020038|ref|ZP\_00690646.1|
Protein of unknown function UPF01...   
33   2.8

gi|21648164|gb|AAM73342.1|
threonyl-tRNA synthetase [Chlorobium ...   
33   2.8

gi|53804046|ref|YP\_114354.1|
GTP pyrophosphokinase [Methylococcu...   
33   3.1

gi|85859558|ref|YP\_461760.1|
GTP pyrophosphokinase / guanosine-3...   
33   3.2

gi|33567914|emb|CAE31827.1|
molybdopterin converting factor [Bor...   
33   3.3

gi|15621527|dbj|BAB65522.1|
236aa long hypothetical molybdopteri...   
33   3.3

gi|16413496|emb|CAC96268.1|
lin1037 [Listeria innocua] >gi|16800...   
33   3.5

gi|88931544|ref|ZP\_01137238.1|
putative molybdopterin converting...   
33   3.6

gi|56381096|dbj|BAD77004.1|
threonyl-tRNA synthetase [Geobacillu...   
33   3.6

gi|33563747|emb|CAE42987.1|
molybdopterin converting factor [Bor...   
33   3.8

gi|89892986|ref|YP\_516473.1|
hypothetical protein DSY0240 [Desul...   
33   3.9

gi|56381270|dbj|BAD77178.1|
N-methylhydantoinase B (ATP-hydrolyz...   
33   4.0

gi|54017794|dbj|BAD59164.1|
hypothetical protein [Nocardia farci...   
33   4.0

gi|56315277|emb|CAI09922.1|
conserved hypothetical protein [Azoa...   
33   4.2

gi|41326217|emb|CAF20379.1|
Sulfur transfer protein involved in ...   
33   4.5

gi|71366157|ref|ZP\_00656703.1|
Ferredoxin--nitrite reductase [No...   
33   4.5

gi|77918225|ref|YP\_356040.1|
molybdopterin biosynthesis protein ...   
33   4.6

gi|83746448|ref|ZP\_00943499.1|
GTP pyrophosphokinase / Guanosine...   
33   5.0

gi|39575280|emb|CAE79448.1|
GTP pyrophosphokinase [Bdellovibrio ...   
33   5.0

gi|10443847|gb|AAG17607.1|
ppGpp synthetase/hydrolase Rel [Geoba...   
33   5.1

gi|62359300|gb|AAX79740.1|
hypothetical protein, conserved [Tryp...   
33   5.1

gi|13815697|gb|AAK42541.1|
Molybdenum cofactor biosynthesis prot...   
33   5.2

gi|83716552|ref|YP\_439903.1|
ThiS family domain protein, putativ...   
33   5.2

gi|53802996|ref|YP\_115212.1|
electron transport complex, H subun...   
33   5.3

gi|83748296|ref|ZP\_00945321.1|
GTP pyrophosphokinase / Guanosine...   
32   5.4

gi|72393974|gb|AAZ68251.1|
Trigger factor [Ehrlichia canis str. ...   
32   5.5

gi|67549235|ref|ZP\_00427105.1|
Protein of unknown function UPF01...   
32   5.6

gi|86739160|ref|YP\_479560.1|
thiamine S [Frankia sp. CcI3] >gi|8...   
32   5.6

gi|106890176|ref|ZP\_01357372.1|
Protein of unknown function DUF8...   
32   5.7

gi|58581359|ref|YP\_200375.1|
ATP:GTP 3'-pyrophosphotranferase [X...   
32   5.8

gi|2622875|gb|AAB86213.1|
unknown [Methanothermobacter thermauto...   
32   5.9

gi|91788565|ref|YP\_549517.1|
(p)ppGpp synthetase I (GTP pyrophos...   
32   5.9

gi|84623294|ref|YP\_450666.1|
ATP:GTP 3'-pyrophosphotranferase [X...   
32   6.0

gi|77995664|gb|ABB14563.1|
putative cell division protein FtsA [...   
32   6.1

gi|17428592|emb|CAD15278.1|
probable gtp pyrophosphokinase (atp:...   
32   6.3

gi|78048800|ref|YP\_364975.1|
Guanosine 3',5'-bis-pyrophosphate s...   
32   6.6

gi|21109438|gb|AAM37958.1|
ATP:GTP 3'-pyrophosphotranferase [Xan...   
32   6.8

gi|89901918|ref|YP\_524389.1|
(p)ppGpp synthetase I (GTP pyrophos...   
32   6.9

gi|66967317|ref|ZP\_00414858.1|
ThiamineS [Arthrobacter sp. FB24]...   
32   7.2

gi|83373271|ref|ZP\_00918050.1|
ATPas [Rhodobacter sphaeroides AT...   
32   7.5

gi|88951090|ref|ZP\_01153664.1|
conserved hypothetical protein [M...   
32   7.5

gi|55232699|gb|AAV48118.1|
unknown [Haloarcula marismortui ATCC ...   
32   7.5

gi|91776407|ref|YP\_546163.1|
NADH-quinone oxidoreductase, chain ...   
32   7.5

gi|11499688|ref|NP\_070930.1|
hypothetical protein AF2105 [Archae...   
32   8.0

gi|55232719|gb|AAV48138.1|
threonyl-tRNA synthetase [Haloarcula ...   
32   8.0

gi|77383741|gb|ABA75254.1|
Ferredoxin [Pseudomonas fluorescens P...   
32   8.0

gi|19887637|gb|AAM02299.1|
Ferredoxin domain fused to pyruvate-f...   
32   8.2

gi|21226239|ref|NP\_632161.1|
hypothetical protein MM0137 [Methan...   
32   8.2

gi|7379707|emb|CAB84274.1|
hypothetical protein NMA1005 [Neisser...   
32   8.2

gi|85059780|ref|YP\_455482.1|
hypothetical protein SG1802 [Sodali...   
32   8.7

gi|46447964|gb|AAS94620.1|
hypothetical protein DVU\_0136 [Desulf...   
32   8.8

gi|17431817|emb|CAD18495.1|
conserved hypothetical protein [Rals...   
32   8.9

gi|84328291|ref|ZP\_00976298.1|
COG1977: Molybdopterin converting...   
32   9.0

gi|73919796|sp|Q5UX65|SYT\_HALMA
Threonyl-tRNA synthetase (Threon...   
31   9.2

gi|16761538|ref|NP\_457155.1|
hypothetical protein STY2872 [Salmo...   
31   9.2

gi|16766001|ref|NP\_461616.1|
hypothetical protein STM2686 [Salmo...   
31   9.4

gi|49531021|emb|CAG68733.1|
hypothetical protein [Acinetobacter ...   
31   9.5

gi|84502919|ref|ZP\_01001032.1|
branched-chain amino acid ABC tra...   
31   9.7

gi|23013438|ref|ZP\_00053332.1|
COG1034: NADH dehydrogenase/NADH:...   
31   10.0

 

------------------------------------------------------------

Reference for
composition-based statistics:

Schaffer, Alejandro A., L.
Aravind, Thomas L. Madden,

Sergei Shavirin, John L.
Spouge, Yuri I. Wolf,

Eugene V. Koonin, and Stephen F. Altschul (2001),

"Improving the
accuracy of PSI-BLAST protein database searches with

composition-based
statistics and other refinements", 
Nucleic Acids Res. 29:2994-3005.

 

Query=
16079947 [TGS domain from B. subtilis]

         (66 letters)

 

Database: All non-redundant
GenBank CDS

translations+PDB+SwissProt+PIR+PRF
excluding environmental samples

           3,682,060 sequences; 1,264,608,837
total letters

 

Results
from round 13

 

gi|88931701|ref|ZP\_01137394.1|
RelA/SpoT protein [Acidothermus c...   
91   1e-17

gi|10443847|gb|AAG17607.1|
ppGpp synthetase/hydrolase Rel [Geoba...   
90   2e-17

gi|10173858|dbj|BAB04961.1|
GTP pyrophosphokinase (stringent res...   
89   4e-17

gi|22294305|dbj|BAC08136.1|
GTP pyrophosphokinase [Thermosynecho...   
89   4e-17

gi|85712954|ref|ZP\_01043993.1|
(p)ppGpp synthetase II [Idiomarin...   
89   4e-17

gi|94493265|ref|ZP\_01300469.1|
hypothetical protein Rgryl\_010010...   
89   6e-17

gi|55773099|dbj|BAD71540.1|
guanosine-3',5'-bis(diphosphate) 3'-...   
89   7e-17

gi|46199657|ref|YP\_005324.1|
guanosine-3',5'-bis(diphosphate) 3'...   
89   7e-17

gi|32030153|ref|ZP\_00133054.1|
COG0317: Guanosine polyphosphate ...   
88   1e-16

gi|56380955|dbj|BAD76863.1|
GTP pyrophosphokinase (ppGpp synthet...   
87   2e-16

gi|78194783|gb|ABB32550.1|
(p)ppGpp synthetase I (GTP pyrophosph...   
87   2e-16

gi|35213317|dbj|BAC90689.1|
(p)ppGpp 3'-pyrophosphohydrolase [Gl...   
87   2e-16

gi|17135369|dbj|BAB77915.1|
(p)ppGpp 3-pyrophosphohydrolase [Nos...   
87   2e-16

gi|77384441|gb|ABA75954.1|
(p)ppGpp synthetase I (GTP pyrophosph...   
87   2e-16

gi|104780553|ref|YP\_607051.1|
GTP pyrophosphokinase RelA [Pseudo...   
87   2e-16

gi|82738644|ref|ZP\_00901472.1|
RelA/SpoT protein [Pseudomonas pu...   
87   2e-16

gi|26988388|ref|NP\_743813.1|
GTP pyrophosphokinase [Pseudomonas ...   
87   2e-16

gi|71674872|ref|ZP\_00672618.1|
RelA/SpoT protein [Trichodesmium ...    87   2e-16

gi|68560858|ref|ZP\_00600166.1| RelA/SpoT protein [Rubrobacter xy...    87  
2e-16

gi|90416445|ref|ZP\_01224376.1|
RelA/SpoT protein [marine gamma p...   
87   2e-16

gi|77635117|ref|ZP\_00797206.1|
COG0317: Guanosine polyphosphate ...   
87   2e-16

gi|77956803|ref|ZP\_00820881.1|
COG0317: Guanosine polyphosphate ...   
87   2e-16

gi|70731792|ref|YP\_261534.1|
GTP pyrophosphokinase [Pseudomonas ...   
87   2e-16

gi|77976031|ref|ZP\_00831564.1|
COG0317: Guanosine polyphosphate ...   
87   2e-16

gi|71554396|gb|AAZ33607.1|
GTP pyrophosphokinase [Pseudomonas sy...   
87   2e-16

gi|45435021|gb|AAS60581.1|
GTP pyrophosphokinase [Yersinia pesti...   
87   2e-16

gi|63257631|gb|AAY38727.1|
RelA/SpoT protein [Pseudomonas syring...   
87   2e-16

gi|7226990|gb|AAF42080.1|
GTP pyrophosphokinase [Neisseria menin...   
87   2e-16

gi|77962817|ref|ZP\_00826633.1|
COG0317: Guanosine polyphosphate ...   
87   2e-16

gi|75704910|gb|ABA24586.1|
(p)ppGpp synthetase I (GTP pyrophosph...   
87   2e-16

gi|77979606|ref|ZP\_00835023.1|
COG0317: Guanosine polyphosphate ...   
87   3e-16

gi|76258990|ref|ZP\_00766642.1|
RelA/SpoT protein [Chloroflexus a...   
87   3e-16

gi|93454321|gb|EAT04628.1|
RelA/SpoT protein [delta proteobacter...   
87   3e-16

gi|33577193|emb|CAE35750.1|
putative GTP pyrophosphokinase [Bord...   
87   3e-16

gi|59801730|ref|YP\_208442.1|
putative GTP pyrophosphokinase [Nei...   
87   3e-16

gi|77996833|gb|ABB15732.1|
GTP pyrophosphokinase [Carboxydotherm...   
87   3e-16

gi|88946349|ref|ZP\_01149430.1|
RelA/SpoT protein [Desulfotomacul...   
87   3e-16

gi|7380622|emb|CAB85211.1|
GTP pyrophosphokinase [Neisseria meni...   
87   3e-16

gi|85667297|ref|ZP\_01029516.1|
hypothetical protein Badol\_010002...   
87   3e-16

gi|39575280|emb|CAE79448.1|
GTP pyrophosphokinase [Bdellovibrio ...  
 86   3e-16

gi|16761735|ref|NP\_457352.1|
GTP pyrophosphokinase [Salmonella e...   
86   3e-16

gi|62181466|ref|YP\_217883.1|
(p)ppGpp synthetase I (GTP pyrophos...   
86   3e-16

gi|71899187|ref|ZP\_00681350.1| RelA/SpoT protein [Xylella fastid...    86  
3e-16

gi|9106309|gb|AAF84125.1| ATP:GTP 3'-pyrophosphotranferase [Xyle...    86  
3e-16

gi|78166182|gb|ABB23280.1| metal dependent phosphohydrolase [Pel...    86  
3e-16

gi|71274878|ref|ZP\_00651166.1| RelA/SpoT protein [Xylella fastid...    86  
3e-16

gi|28198473|ref|NP\_778787.1| ATP:GTP 3'-pyrophosphotranferase [X...    86  
3e-16

gi|98661027|dbj|GAA01813.1| unnamed protein product [Pelotomacul...    86  
4e-16

gi|71901846|ref|ZP\_00683910.1| RelA/SpoT protein [Xylella fastid...    86  
4e-16

gi|46143604|ref|ZP\_00134847.2| COG0317: Guanosine polyphosphate ...    86  
4e-16

gi|29341315|gb|AAO79103.1| GTP pyrophosphokinase [Bacteroides th...    86  
4e-16

gi|94491518|ref|ZP\_01298729.1| hypothetical protein CburD\_010013...    86  
4e-16

gi|33572311|emb|CAE41865.1| guanosine-3',5'-bis(diphosphate) 3'-...    86  
4e-16

gi|49613016|emb|CAG76467.1| GTP pyrophosphokinase [Erwinia carot...    86  
4e-16

gi|75176354|ref|ZP\_00696498.1| COG0317: Guanosine polyphosphate ...    86  
4e-16

gi|75256877|ref|ZP\_00728471.1| COG0317: Guanosine polyphosphate ...    86  
4e-16

gi|29541940|gb|AAO90878.1| GTP pyrophosphokinase [Coxiella burne...    86  
4e-16

gi|36784328|emb|CAE13205.1| GTP pyrophosphokinase [Photorhabdus ...    86  
4e-16

gi|6900418|emb|CAB72029.1| GTP-pyrophosphokinase [Neisseria meni...    86  
4e-16

gi|75196755|ref|ZP\_00706825.1| COG0317: Guanosine polyphosphate ...    86  
4e-16

gi|147562|gb|AAA03237.1| ATP:GTP 3'-pyrophosphotransferase             86   5e-16

gi|24053195|gb|AAN44285.1| (p)ppGpp synthetase I (GTP pyrophosph...    86  
5e-16

gi|75241826|ref|ZP\_00725645.1| COG0317: Guanosine polyphosphate ...    86  
5e-16

gi|16766262|ref|NP\_461877.1| (p)ppGpp synthetase I [Salmonella t...    86  
5e-16

gi|56909578|dbj|BAD64105.1| GTP pyrophosphokinase [Bacillus clau...    86  
5e-16

gi|52214922|dbj|BAD47515.1| GTP pyrophosphokinase [Bacteroides f...    86  
5e-16

gi|53804046|ref|YP\_114354.1| GTP pyrophosphokinase [Methylococcu...    86  
5e-16

gi|60491685|emb|CAH06437.1| putative RelA/SpoT GTP pyrophosphoki...    85  
5e-16

gi|91788565|ref|YP\_549517.1| (p)ppGpp synthetase I (GTP pyrophos...    85  
5e-16

gi|85058488|ref|YP\_454190.1| GTP pyrophosphokinase [Sodalis glos...    85  
5e-16

gi|78171930|gb|ABB29026.1| metal dependent phosphohydrolase [Chl...    85  
6e-16

gi|57224123|gb|AAW39180.1| GTP pyrophosphokinase [Dehalococcoide...    85  
6e-16

gi|68057138|gb|AAX87391.1| GTP pyrophosphokinase [Haemophilus in...    85   6e-16

gi|71481204|ref|ZP\_00660912.1|
RelA/SpoT protein [Prosthecochlor...   
85   6e-16

gi|21647542|gb|AAM72771.1|
GTP pyrophosphokinase [Chlorobium tep...   
85   6e-16

gi|53732953|ref|ZP\_00155341.2|
COG0317: Guanosine polyphosphate ...   
85   6e-16

gi|78702020|ref|ZP\_00866464.1|
RelA/SpoT protein [Alkalilimnicol...   
85   6e-16

gi|88932675|ref|ZP\_01138357.1|
RelA/SpoT protein [Dehalococcoide...   
85   6e-16

gi|94986614|ref|YP\_594547.1|
Guanosine polyphosphate pyrophospho...   
85   6e-16

gi|83748296|ref|ZP\_00945321.1|
GTP pyrophosphokinase / Guanosine...   
85   7e-16

gi|73659677|emb|CAI82284.1|
GTP pyrophosphokinase [Dehalococcoid...   
85   7e-16

gi|28852139|gb|AAO55214.1|
GTP pyrophosphokinase [Pseudomonas sy...   
85   7e-16

gi|1172903|sp|P44644|RELA\_HAEIN
GTP pyrophosphokinase (ATP:GTP 3...   
85   7e-16

gi|17428592|emb|CAD15278.1|
probable gtp pyrophosphokinase (atp:...   
85   7e-16

gi|33564531|emb|CAE43846.1|
putative GTP pyrophosphokinase [Bord...   
85   8e-16

gi|89100794|ref|ZP\_01173647.1|
GTP pyrophosphokinase [Bacillus s...   
85   8e-16

gi|33576387|emb|CAE33465.1|
guanosine-3',5'-bis(diphosphate) 3'-...   
85   9e-16

gi|52306348|gb|AAU36848.1|
SpoT protein [Mannheimia succinicipro...   
85   1e-15

gi|66966401|ref|ZP\_00413961.1|
RelA/SpoT protein [Arthrobacter s...   
85   1e-15

gi|50840248|gb|AAT82915.1|
GTP pyrophosphokinase [Propionibacter...   
85   1e-15

gi|42630634|ref|ZP\_00156173.1|
COG0317: Guanosine polyphosphate ...   
85   1e-15

gi|27262490|gb|AAN87526.1|
GTP pyrophosphokinase [Heliobacillus ...   
85   1e-15

gi|12722293|gb|AAK03949.1| RelA [Pasteurella multocida subsp. mu...    84  
1e-15

gi|88856261|ref|ZP\_01130921.1|
GTP pyrophosphokinase [marine act...   
84   1e-15

gi|85859448|ref|YP\_461650.1|
GTP pyrophosphokinase / guanosine-3...   
84   1e-15

gi|78219017|gb|ABB38366.1|
metal dependent phosphohydrolase [Des...   
84   1e-15

gi|53754334|emb|CAH15811.1|
GTP pyrophosphokinase [Legionella pn...   
84   1e-15

gi|39984225|gb|AAR35612.1|
GTP pyrophosphokinase [Geobacter sulf...   
84   1e-15

gi|53751153|emb|CAH12564.1|
GTP pyrophosphokinase [Legionella pn...  
 84   1e-15

gi|77918891|ref|YP\_356706.1|
GTP pyrophosphokinase [Pelobacter c...   
84   1e-15

gi|52628798|gb|AAU27539.1|
GTP pyrophosphokinase ((p)ppGpp synth...   
84   1e-15

gi|23467900|ref|ZP\_00123476.1|
COG0317: Guanosine polyphosphate ...   
84   1e-15

gi|94985409|ref|YP\_604773.1|
(p)ppGpp synthetase I, SpoT/RelA [D...   
84   1e-15

gi|67158852|ref|ZP\_00419670.1|
RelA/SpoT protein [Azotobacter vi...   
84   1e-15

gi|83644638|ref|YP\_433073.1|
GTP pyrophosphokinase [Hahella chej...   
84   1e-15

gi|2635224|emb|CAB14719.1|
GTP pyrophosphokinase [Bacillus subti...   
84   2e-15

gi|2739435|gb|AAC46041.1|
(p)ppGpp synthetase [Bacillus subtilis]     
84   2e-15

gi|92911903|ref|ZP\_01280541.1|
RelA/SpoT protein [Mycobacterium ...   
84   2e-15

gi|92915905|ref|ZP\_01284527.1|
RelA/SpoT protein [Mycobacterium ...   
84   2e-15

gi|23465997|ref|NP\_696600.1| RelA [Bifidobacterium longum NCC270...    84  
2e-15

gi|88806995|ref|ZP\_01122510.1| GTP pyrophosphokinase [Robiginita...    84  
2e-15

gi|84316541|ref|ZP\_00965014.1| COG0317: Guanosine polyphosphate ...    84  
2e-15

gi|82499307|ref|ZP\_00884755.1| RelA/SpoT protein [Caldicellulosi...    84  
2e-15

gi|88936674|ref|ZP\_01142276.1| RelA/SpoT protein [Geobacter uran...    84  
2e-15

gi|84711700|ref|ZP\_01019846.1| RelA/SpoT protein [Polaromonas na...    84  
2e-15

gi|75815082|ref|ZP\_00745609.1| COG0317: Guanosine polyphosphate ...    84  
2e-15

gi|68548662|ref|ZP\_00588131.1| RelA/SpoT protein [Pelodictyon ph...    84  
2e-15

gi|88703547|ref|ZP\_01101263.1| GTP pyrophosphokinase [gamma prot...    84  
2e-15

gi|9946839|gb|AAG04323.1| GTP pyrophosphokinase [Pseudomonas aer...    84  
2e-15

gi|94417967|ref|ZP\_01297783.1| hypothetical protein PaerP\_010001...    84  
2e-15

gi|9657028|gb|AAF95593.1| GTP pyrophosphokinase [Vibrio cholerae...    84  
2e-15

gi|62426374|ref|ZP\_00381502.1| COG0317: Guanosine polyphosphate ...    84  
2e-15

gi|75817547|ref|ZP\_00747913.1| COG0317: Guanosine polyphosphate ...    84  
2e-15

gi|82703620|ref|YP\_413186.1| (p)ppGpp synthetase I (GTP pyrophos...    84  
2e-15

gi|71144597|gb|AAZ25070.1| GTP pyrophosphokinase [Colwellia psyc...    84  
2e-15

gi|71908440|ref|YP\_286027.1| RelA/SpoT protein [Dechloromonas ar...    84  
2e-15

gi|34541428|ref|NP\_905907.1| guanosine-3',5'-bis(diphosphate) 3'...    84  
2e-15

gi|6647734|sp|O52177|RELA\_MYXXA GTP pyrophosphokinase (ATP:GTP 3...    84  
2e-15

gi|72162489|ref|YP\_290146.1| RelA/SpoT protein [Thermobifida fus...    84  
2e-15

gi|89891182|ref|ZP\_01202689.1| GTP pyrophosphokinase, RelA/SpoT ...    84   2e-15

gi|34499157|ref|NP\_903372.1|
GTP diphosphokinase [Chromobacteriu...   
84   2e-15

gi|88795069|ref|ZP\_01110771.1|
GTP pyrophosphokinase [Alteromona...   
84   2e-15

gi|86158972|ref|YP\_465757.1|
(p)ppGpp synthetase I (GTP pyrophos...   
84   3e-15

gi|68055166|ref|ZP\_00539314.1|
RelA/SpoT protein [Exiguobacteriu...   
83   3e-15

gi|78048800|ref|YP\_364975.1|
Guanosine 3',5'-bis-pyrophosphate s...   
83   3e-15

gi|68195641|gb|EAN10081.1|
Threonyl-tRNA synthetase, class IIa [...   
83   3e-15

gi|67987940|gb|EAM75725.1|
RelA/SpoT protein [Kineococcus radiot...   
83   3e-15

gi|21109438|gb|AAM37958.1|
ATP:GTP 3'-pyrophosphotranferase [Xan...   
83   3e-15

gi|84623294|ref|YP\_450666.1|
ATP:GTP 3'-pyrophosphotranferase [X...   
83   3e-15

gi|71837995|ref|ZP\_00677757.1|
RelA/SpoT protein [Pelobacter pro...   
83   3e-15

gi|16330670|ref|NP\_441398.1|
(p)ppGpp 3'-pyrophosphohydrolase [S...   
83   3e-15

gi|56178922|gb|AAV81644.1|
(p)ppGpp synthetase II; guanosine-3',...   
83   3e-15

gi|91784429|ref|YP\_559635.1|
(p)ppGpp synthetase I (GTP pyrophos...   
83   3e-15

gi|84361562|ref|ZP\_00986225.1|
COG0317: Guanosine polyphosphate ...   
83   3e-15

gi|71547992|ref|ZP\_00668495.1|
RelA/SpoT protein [Syntrophobacte...   
83   3e-15

gi|21114142|gb|AAM42208.1|
ATP:GTP 3'-pyrophosphotranferase [Xan...   
83   3e-15

gi|58581359|ref|YP\_200375.1|
ATP:GTP 3'-pyrophosphotranferase [X...   
83   3e-15

gi|66572833|gb|AAY48243.1|
ATP:GTP 3'-pyrophosphotranferase [Xan...   
83   3e-15

gi|83719568|ref|YP\_443112.1|
GTP pyrophosphokinase [Burkholderia...   
83   3e-15

gi|89201354|ref|ZP\_01180095.1| RelA/SpoT protein [Bacillus cereu...    83  
3e-15

gi|89895197|ref|YP\_518684.1| hypothetical protein DSY2451 [Desul...    83  
3e-15

gi|86143087|ref|ZP\_01061509.1| GTP pyrophosphokinase [Flavobacte...    83  
3e-15

gi|72118382|gb|AAZ60645.1| RelA/SpoT protein [Ralstonia eutropha...    83  
3e-15

gi|37958841|gb|AAP51105.1| putative pyrophosphokinase [unculture...    83  
3e-15

gi|88813282|ref|ZP\_01128521.1| (p)ppGpp synthetase I [Nitrococcu...    83   3e-15

gi|106883705|ref|ZP\_01351099.1|
RelA/SpoT family protein [Psychr...   
83   4e-15

gi|52004450|gb|AAU24392.1| GTP pyrophosphokinase [Bacillus liche...    83  
4e-15

gi|83590516|ref|YP\_430525.1| (p)ppGpp synthetase I (GTP pyrophos...    83  
4e-15

gi|77966830|gb|ABB08210.1| (p)ppGpp synthetase I (GTP pyrophosph...    83  
4e-15

gi|34499223|ref|NP\_903438.1| guanosine-3',5'-bis(diphosphate) 3'...    83  
4e-15

gi|2983390|gb|AAC06975.1| (p)ppGpp 3-pyrophosphohydrolase [Aquif...    83  
4e-15

gi|67938770|ref|ZP\_00531290.1| RelA/SpoT protein [Chlorobium pha...    83  
4e-15

gi|33152293|ref|NP\_873646.1| GTP pyrophosphokinase [Haemophilus ...    83  
4e-15

gi|77815280|ref|ZP\_00814518.1| RelA/SpoT protein [Shewanella put...    83  
4e-15

gi|88797492|ref|ZP\_01113081.1| RelA/SpoT protein [Reinekea sp. M...    82  
5e-15

gi|58002652|gb|AAW61546.1| GTP pyrophosphokinase [Gluconobacter ...    82  
5e-15

gi|30248385|ref|NP\_840455.1| spoT; bifunctional enzyme (p)ppgpp ...    82  
5e-15

gi|67917945|ref|ZP\_00511548.1| RelA/SpoT protein [Chlorobium lim...    82  
5e-15

gi|71549789|ref|ZP\_00669971.1| RelA/SpoT protein [Nitrosomonas e...    82  
5e-15

gi|82523943|emb|CAI78665.1| guanosine polyphosphate pyrophosphoh...    82  
5e-15

gi|29898040|gb|AAP11314.1| GTP pyrophosphokinase [Bacillus cereu...    82   5e-15

gi|50951235|gb|AAT88936.1|
GTP pyrophosphokinase [Leifsonia xyli...   
82   5e-15

gi|42783537|ref|NP\_980784.1|
GTP pyrophosphokinase [Bacillus cer...   
82   5e-15

gi|47505083|gb|AAT33759.1|
GTP pyrophosphokinase [Bacillus anthr...   
82   5e-15

gi|47566596|ref|ZP\_00237418.1|
GTP pyrophosphokinase [Bacillus c...   
82   5e-15

gi|49333045|gb|AAT63691.1|
GTP diphosphokinase (GTP pyrophosphok...   
82   5e-15

gi|49532055|emb|CAG69767.1|
GTP pyrophosphokinase (ATP:GTP 3'-py...   
82   5e-15

gi|81300186|ref|YP\_400394.1|
metal dependent phosphohydrolase [S...   
82   5e-15

gi|56685147|dbj|BAD78369.1|
GTP pyrophosphokinase [Synechococcus...   
82   6e-15

gi|56315211|emb|CAI09856.1|
GTP pyrophosphokinase (ATP:GTP 3'-py...   
82   6e-15

gi|75760574|ref|ZP\_00740607.1|
GTP pyrophosphokinase  / Guanosin...    82  
6e-15

gi|87118649|ref|ZP\_01074548.1|
RelA/SpoT protein [Marinomonas sp...   
82   6e-15

gi|68263724|emb|CAI37212.1|
GTP pyrophosphokinase [Corynebacteri...   
82   6e-15

gi|86609008|ref|YP\_477770.1|
RelA/SpoT family protein [Synechoco...   
82   7e-15

gi|91775651|ref|YP\_545407.1|
(p)ppGpp synthetase I (GTP pyrophos...   
82   7e-15

gi|16303981|gb|AAL16895.1|
ppGpp synthetase [Streptomyces clavul...  
 82   7e-15

gi|23493608|dbj|BAC18577.1|
GTP pyrophosphokinase [Corynebacteri...   
82   7e-15

gi|51974569|gb|AAU16119.1|
GTP diphosphokinase (GTP pyrophosphok...   
82   7e-15

gi|18145602|dbj|BAB81644.1|
GTP pyrophosphokinase [Clostridium p...   
82   7e-15

gi|68546863|ref|ZP\_00586407.1| RelA/SpoT protein [Shewanella ama...    82  
7e-15

gi|6459617|gb|AAF11392.1| GTP pyrophosphokinase [Deinococcus rad...    82  
8e-15

gi|1710084|sp|P55133|RELA\_VIBSS GTP pyrophosphokinase (ATP:GTP 3...    82   8e-15

gi|48870705|ref|ZP\_00323424.1|
COG0317: Guanosine polyphosphate ...   
82   8e-15

gi|41325873|emb|CAF20036.1|
PPGPP SYNTHETASE, PPGPP PYROPHOSPHOR...   
82   8e-15

gi|21324422|dbj|BAB99046.1|
Guanosine polyphosphate pyrophosphoh...   
82   8e-15

gi|28807574|dbj|BAC60827.1|
GTP pyrophosphokinase [Vibrio paraha...   
82   8e-15

gi|89075081|ref|ZP\_01161522.1|
putative GTP pyrophosphokinase [P...   
82   9e-15

gi|77955427|ref|ZP\_00819793.1|
RelA/SpoT protein [Marinobacter a...   
82   9e-15

gi|76578167|gb|ABA47642.1|
GTP pyrophosphokinase [Burkholderia p...   
82   9e-15

gi|13092715|emb|CAC29999.1|
putative GTP pyrophosphokinase [Myco...   
82   9e-15

gi|86132238|ref|ZP\_01050833.1|
GTP pyrophosphokinase [Cellulopha...   
82   9e-15

gi|106890468|ref|ZP\_01357662.1|
RelA/SpoT family protein [Roseif...   
82   9e-15

gi|90290647|ref|ZP\_01210296.1|
hypothetical protein Bpse17\_02004...   
82   9e-15

gi|85064736|ref|ZP\_01025589.1|
COG0317: Guanosine polyphosphate ...   
82   9e-15

gi|31619361|emb|CAD94799.1|
PROBABLE GTP PYROPHOSPHOKINASE RELA ...   
82   9e-15

gi|52209973|emb|CAH35945.1|
GTP pyrophosphokinase [Burkholderia ...   
82   9e-15

gi|84522846|ref|ZP\_01009982.1|
COG0317: Guanosine polyphosphate ...   
82   9e-15

gi|13882405|gb|AAK46973.1|
GTP pyrophosphokinase [Mycobacterium ...   
82   9e-15

gi|56313451|emb|CAI08096.1|
guanosine-3',5'-bis(diphosphate) 3'-...   
82   1e-14

gi|78365752|ref|ZP\_00836037.1|
RelA/SpoT protein [Shewanella sp....    82   1e-14

gi|59801660|ref|YP\_208372.1| putative guanosine-3',5'-bis(diphos...    82  
1e-14

gi|27361090|gb|AAO09999.1| Guanosine polyphosphate pyrophosphohy...    82  
1e-14

gi|84496349|ref|ZP\_00995203.1| putative ppGpp synthetase [Janiba...    81  
1e-14

gi|68233513|ref|ZP\_00572631.1| RelA/SpoT protein [Frankia sp. EA...    81  
1e-14

gi|52428718|gb|AAU49311.1| GTP pyrophosphokinase [Burkholderia m...    81   1e-14

gi|94310104|ref|YP\_583314.1|
(p)ppGpp synthetase I (GTP pyrophos...   
81   1e-14

gi|91796852|gb|ABE58991.1|
(p)ppGpp synthetase I (GTP pyrophosph...   
81   1e-14

gi|94502135|ref|ZP\_01308636.1|
GTP pyrophosphokinase [Oceanobact...   
81   1e-14

gi|75208678|ref|ZP\_00708975.1|
COG0317: Guanosine polyphosphate ...   
81   1e-14

gi|88705448|ref|ZP\_01103159.1|
guanosine-3',5'-bis(diphosphate) ...   
81   1e-14

gi|26110727|gb|AAN82911.1|
Guanosine-3',5'-bis(Diphosphate) 3'-p...   
81   1e-14

gi|82779141|ref|YP\_405490.1|
(p)ppGpp synthetase II [Shigella dy...   
81   1e-14

gi|73671332|gb|AAZ80077.1|
SpoT [Escherichia coli]                    
81   1e-14

gi|89070106|ref|ZP\_01157436.1|
guanosine-3',5'-bis(Diphosphate) ...   
81   1e-14

gi|82701185|ref|YP\_410751.1|
(p)ppGpp synthetase I (GTP pyrophos...   
81   1e-14

gi|24054163|gb|AAN45137.1|
(p)ppGpp synthetase II and guanosine-...   
81   1e-14

gi|18071685|gb|AAL58286.1|
putative GTP pyrophosphokinase RelA [...   
81   1e-14

gi|71490588|gb|EAO22958.1|
RelA/SpoT protein [Syntrophomonas wol...   
81   1e-14

gi|77958430|ref|ZP\_00822463.1|
COG0317: Guanosine polyphosphate ...   
81   1e-14

gi|77962008|ref|ZP\_00825835.1|
COG0317: Guanosine polyphosphate ...   
81   1e-14

gi|23124721|ref|ZP\_00106692.1|
COG0317: Guanosine polyphosphate ...   
81   1e-14

gi|22777708|dbj|BAC13980.1|
GTP pyrophosphokinase (stringent res...   
81   1e-14

gi|52629337|gb|AAU28078.1|
guanosine-3,5-bis(diphosphate)-3-pyro...   
81   1e-14

gi|90205646|ref|ZP\_01208285.1|
RelA/SpoT protein [Mycobacterium ...   
81   1e-14

gi|53754738|emb|CAH16225.1|
guanosine-3',5'-bis(diphosphate) 3'-...   
81   1e-14

gi|29833382|ref|NP\_828016.1|
ppGpp synthetase [Streptomyces aver...   
81   1e-14

gi|91774406|ref|YP\_544162.1|
(p)ppGpp synthetase I (GTP pyrophos...   
81   1e-14

gi|53751720|emb|CAH13142.1|
guanosine-3',5'-bis(diphosphate) 3'-...   
81   1e-14

gi|91216450|ref|ZP\_01253416.1|
GTP pyrophosphokinase [Psychrofle...   
81   1e-14

gi|62463551|ref|ZP\_00382875.1|
COG0317: Guanosine polyphosphate ...   
81   1e-14

gi|88939892|ref|ZP\_01145336.1|
RelA/SpoT family protein [Acidiph...   
81   1e-14

gi|41407145|ref|NP\_959981.1|
RelA [Mycobacterium avium subsp. pa...   
81   1e-14

gi|15025278|gb|AAK80231.1|
RelA/SpoT protein, (p)ppGpp synthetas...   
81   1e-14

gi|38200214|emb|CAE49898.1|
GTP pyrophosphokinase [Corynebacteri...   
81   1e-14

gi|71369363|ref|ZP\_00659815.1|
RelA/SpoT protein [Nocardioides s...    81  
1e-14

gi|91227845|ref|ZP\_01262018.1|
GTP pyrophosphokinase [Vibrio alg...   
81   1e-14

gi|86163582|gb|EAQ64856.1|
guanosine-3',5'-bis(diphosphate) 3'-p...   
81   2e-14

gi|90408289|ref|ZP\_01216454.1|
(p)ppGpp synthetase I (GTP pyroph...   
81   2e-14

gi|86606590|ref|YP\_475353.1|
RelA/SpoT family protein [Synechoco...   
81   2e-14

gi|12722952|gb|AAK04204.1|
ppGpp synthetase I (EC 2.7.6.5) [Lact...   
81   2e-14

gi|107022546|ref|YP\_620873.1|
(p)ppGpp synthetase I, SpoT/RelA [...   
81   2e-14

gi|16762568|ref|NP\_458185.1|
guanosine-3',5'-bis(diphosphate) 3'...   
81   2e-14

gi|16767027|ref|NP\_462642.1|
(p)ppGpp synthetase II/guanosine-3'...   
81   2e-14

gi|84357523|ref|ZP\_00982340.1|
COG0317: Guanosine polyphosphate ...   
81   2e-14

gi|59712687|ref|YP\_205463.1|
GTP pyrophosphokinase [Vibrio fisch...   
81   2e-14

gi|67668174|ref|ZP\_00465362.1| RelA/SpoT protein [Burkholderia c...    81  
2e-14

gi|69953342|ref|ZP\_00640509.1|
RelA/SpoT protein [Shewanella fri...   
80   2e-14

gi|84500567|ref|ZP\_00998816.1|
guanosine-3',5'-bis(Diphosphate) ...   
80   2e-14

gi|7226911|gb|AAF42008.1|
guanosine-3`,5`-bis(diphosphate) 3`-py...   
80   2e-14

gi|90588990|ref|ZP\_01244641.1|
RelA/SpoT protein [Flavobacterium...   
80   2e-14

gi|104773939|ref|YP\_618919.1|
GTP pyrophosphokinase [Lactobacill...    80   2e-14

gi|74016055|ref|ZP\_00686682.1| RelA/SpoT protein [Burkholderia a...    80  
2e-14

gi|37681005|ref|NP\_935614.1| GTP pyrophosphokinase [Vibrio vulni...    80   2e-14

gi|1515319|emb|CAA63297.1|
(p)ppGpp synthetase [Streptomyces coe...   
80   2e-14

gi|86740082|ref|YP\_480482.1|
(p)ppGpp synthetase I [Frankia sp. ...   
80   2e-14

gi|24374961|ref|NP\_719004.1|
GTP pyrophosphokinase [Shewanella o...   
80   2e-14

gi|21220013|ref|NP\_625792.1|
GTP pyrophosphokinase [Streptomyces...   
80   2e-14

gi|78685813|ref|ZP\_00850588.1|
RelA/SpoT protein [Shewanella sp....   
80   2e-14

gi|78691674|ref|ZP\_00856277.1|
RelA/SpoT protein [Shewanella sp....   
80   2e-14

gi|28493242|ref|NP\_787403.1|
GTP pyrophosphokinase [Tropheryma w...   
80   2e-14

gi|7380551|emb|CAB85138.1|
guanosine-3',5'-bis(diphosphate) 3'-p...   
80   2e-14

gi|28410777|emb|CAD67163.1|
GTP pyrophosphokinase [Tropheryma wh...   
80   2e-14

gi|74317776|ref|YP\_315516.1|
GTP diphosphokinase, RelA/SpoT prot...    80  
2e-14

gi|30259135|gb|AAP28340.1|
GTP pyrophosphokinase [Bacillus anthr...   
80   2e-14

gi|90021883|ref|YP\_527710.1|
GTP diphosphokinase [Saccharophagus...   
80   2e-14

gi|67546980|ref|ZP\_00424888.1| RelA/SpoT protein [Burkholderia v...    80  
2e-14

gi|51857264|dbj|BAD41422.1| GTP pyrophosphokinase [Symbiobacteri...    80  
2e-14

gi|68544166|ref|ZP\_00583835.1| RelA/SpoT protein [Shewanella bal...    80  
2e-14

gi|89094556|ref|ZP\_01167494.1|
GTP pyrophosphokinase [Oceanospir...   
80   2e-14

gi|68551501|ref|ZP\_00590896.1|
RelA/SpoT protein [Prosthecochlor...   
80   3e-14

gi|88712828|ref|ZP\_01106913.1|
GTP pyrophosphokinase [Flavobacte...   
80   3e-14

gi|4981255|gb|AAD35811.1|
(p)ppGpp synthetase [Thermotoga mariti...   
80   3e-14

gi|89341145|ref|ZP\_01193390.1|
RelA/SpoT protein [Mycobacterium ...   
80   3e-14

gi|50403843|gb|AAT76675.1|
pp(p)Gpp synthetase/hydrolase [Polyan...   
80   3e-14

gi|83858064|ref|ZP\_00951591.1|
GTP pyrophosphokinase [Croceibact...   
80   3e-14

gi|83815613|ref|YP\_445194.1|
RelA/SpoT family protein [Salinibac...   
80   3e-14

gi|67921546|ref|ZP\_00515064.1|
RelA/SpoT protein [Crocosphaera w...   
80   3e-14

gi|83593191|ref|YP\_426943.1|
(p)ppGpp synthetase I (GTP pyrophos...   
80   3e-14

gi|83746448|ref|ZP\_00943499.1|
GTP pyrophosphokinase / Guanosine...   
80   3e-14

gi|46449907|gb|AAS96556.1|
GTP pyrophosphokinase [Desulfovibrio ...   
80   3e-14

gi|84515158|ref|ZP\_01002521.1| RelA/SpoT family protein [Loktane...    80  
3e-14

gi|89208654|ref|ZP\_01187149.1| RelA/SpoT protein [Bacillus weihe...    80  
3e-14

gi|91787229|ref|YP\_548181.1| (p)ppGpp synthetase I (GTP pyrophos...    80  
3e-14

gi|95931489|ref|ZP\_01314187.1| GTP diphosphokinase [Desulfuromon...    80  
3e-14

gi|47094426|ref|ZP\_00232112.1| GTP pyrophosphokinase [Listeria m...    80  
4e-14

gi|76874601|emb|CAI85822.1| (p)ppGpp synthetase I (GTP pyrophosp...    80  
4e-14

gi|89255717|ref|YP\_513078.1| GTP pyrophosphokinase [Francisella ...    80  
4e-14

gi|67873236|ref|ZP\_00503515.1| RelA/SpoT protein [Clostridium th...    79  
4e-14

gi|14325225|dbj|BAB60670.1| Rel [Listeria monocytogenes]               79   4e-14

gi|45434759|gb|AAS60320.1| guanosine-3',5'-bisbis(diphosphate) 3...    79  
4e-14

gi|46907751|ref|YP\_014140.1| GTP pyrophosphokinase [Listeria mon...    79  
4e-14

gi|16410952|emb|CAC99601.1| relA [Listeria monocytogenes] >gi|47...    79  
4e-14

gi|56605038|emb|CAG46141.1| GTP pyrophosphokinase [Francisella t...    79  
4e-14

gi|1589235|prf||2210370A (p)ppGpp synthetase                           79   4e-14

gi|9951657|gb|AAG08723.1| guanosine-3',5'-bis(diphosphate) 3'-py...    79  
4e-14

gi|47573841|ref|ZP\_00243878.1| COG0317: Guanosine polyphosphate ...    79  
4e-14

gi|89901918|ref|YP\_524389.1| (p)ppGpp synthetase I (GTP pyrophos...    79  
4e-14

gi|78363842|gb|ABB41807.1| (p)ppGpp synthetase I (GTP pyrophosph...    79  
4e-14

gi|88858178|ref|ZP\_01132820.1| (p)ppGpp synthetase I (GTP pyroph...    79  
4e-14

gi|58254550|gb|AAV42787.1| ppGpp synthetase [Lactobacillus acido...    79  
4e-14

gi|87200546|ref|YP\_497803.1| (p)ppGpp synthetase I (GTP pyrophos...    79  
4e-14

gi|28211806|ref|NP\_782750.1| putative GTP pyrophosphokinase [Clo...    79  
4e-14

gi|16414045|emb|CAC96789.1| relA [Listeria innocua] >gi|16800626...    79   4e-14

gi|77955594|ref|ZP\_00819952.1| RelA/SpoT protein [Marinobacter a...    79  
5e-14

gi|91792551|ref|YP\_562202.1|
RelA/SpoT family protein [Shewanell...   
79   5e-14

gi|94495396|ref|ZP\_01301976.1|
(p)ppGpp synthetase I (GTP pyroph...   
79   5e-14

gi|85705105|ref|ZP\_01036205.1|
guanosine-3',5'-bis(Diphosphate) ...   
79   5e-14

gi|68189940|gb|EAN04602.1|
RelA/SpoT protein [Mesorhizobium sp. ...   
79   5e-14

gi|68245488|gb|EAN27610.1|
RelA/SpoT protein [Magnetococcus sp. ...   
79   6e-14

gi|94493440|ref|ZP\_01300644.1|
hypothetical protein Rgryl\_010012...   
79   6e-14

gi|88797955|ref|ZP\_01113542.1|
GTP pyrophosphokinase [Reinekea s...   
79   6e-14

gi|55820243|ref|YP\_138685.1|
(p)ppGpp synthetase [Streptococcus ...   
79   7e-14

gi|42519264|ref|NP\_965194.1|
GTP pyrophosphokinase [Lactobacillu...   
79   7e-14

gi|55738118|gb|AAV61759.1|
(p)ppGpp synthetase [Streptococcus th...   
79   7e-14

gi|90820993|gb|ABD99632.1|
GTP pyrophosphokinase [Lactobacillus ...   
79   7e-14

gi|23002867|ref|ZP\_00046539.1|
hypothetical protein Lgas\_0300079...   
79   7e-14

gi|54017162|dbj|BAD58532.1|
putative ppGpp synthetase [Nocardia ...   
79   7e-14

gi|86146352|ref|ZP\_01064676.1|
GTP pyrophosphokinase [Vibrio sp....   
79   7e-14

gi|89090612|ref|ZP\_01163656.1|
RelA/SpoT protein [Lactobacillus ...   
79   7e-14

gi|48865083|ref|ZP\_00318948.1|
COG0317: Guanosine polyphosphate ...   
79   7e-14

gi|92089513|ref|ZP\_01274453.1|
RelA/SpoT protein [Lactobacillus ...   
79   7e-14

gi|68194634|gb|EAN09121.1|
RelA/SpoT protein [Enterococcus faeci...  
 79   7e-14

gi|77972420|ref|ZP\_00827986.1|
COG0317: Guanosine polyphosphate ...   
79   7e-14

gi|84390111|ref|ZP\_00991373.1|
GTP pyrophosphokinase [Vibrio spl...   
79   8e-14

gi|77976741|ref|ZP\_00832214.1|
COG0317: Guanosine polyphosphate ...   
79   8e-14

gi|99082405|ref|YP\_614559.1|
metal dependent phosphohydrolase [S...   
79   8e-14

gi|24380383|ref|NP\_722338.1|
putative stringent response protein...   
79   8e-14

gi|36783714|emb|CAE12567.1|
guanosine-3',5'-bis(Diphosphate) 3'-...   
79   8e-14

gi|77386633|gb|ABA77818.1|
RelA/SpoT family protein [Rhodobacter...   
78   8e-14

gi|20516195|gb|AAM24425.1|
Guanosine polyphosphate pyrophosphohy...   
78   8e-14

gi|85060201|ref|YP\_455903.1|
guanosine-3',5'-bisbis(diphosphate)...   
78   9e-14

gi|8163822|gb|AAF73866.1|
pentaphosphate guanosine-3'-pyrophosph...   
78   9e-14

gi|88948520|ref|ZP\_01151283.1|
RelA/SpoT protein [Halorhodospira...   
78   9e-14

gi|71839361|ref|ZP\_00679111.1|
Threonyl-tRNA synthetase, class I...    78   9e-14

gi|76883118|gb|ABA57799.1| RelA/SpoT protein [Nitrosococcus ocea...    78  
9e-14

gi|78702353|ref|ZP\_00866791.1| RelA/SpoT protein [Alkalilimnicol...    78  
9e-14

gi|17429172|emb|CAD15860.1| probable bifunctional enzyme : (p)pp...    78  
9e-14

gi|76876613|emb|CAI87835.1| bifunctional: (p)ppGpp synthetase II...    78  
1e-13

gi|70733467|ref|YP\_263242.1| threonyl-tRNA synthetase [Pseudomon...    78  
1e-13

gi|94309803|ref|YP\_583013.1| (p)ppGpp synthetase I (GTP pyrophos...    78  
1e-13

gi|68209554|ref|ZP\_00561529.1| RelA/SpoT protein [Desulfitobacte...    78  
1e-13

gi|83855032|ref|ZP\_00948562.1| guanosine-3',5'-bis(Diphosphate) ...    78  
1e-13

gi|94491025|ref|ZP\_01298246.1| hypothetical protein CburD\_010019...    78  
1e-13

gi|62526979|ref|ZP\_00388284.1| COG0317: Guanosine polyphosphate ...    78  
1e-13

gi|29540916|gb|AAO89860.1| guanosine-3,5-bis(diphosphate) 3-pyro...    78  
1e-13

gi|83941555|ref|ZP\_00954017.1| guanosine-3',5'-bis(Diphosphate) ...    78  
1e-13

gi|95926602|ref|ZP\_01309383.1| hypothetical protein CburR\_010016...    78  
1e-13

gi|88802840|ref|ZP\_01118367.1| GTP pyrophosphokinase [Polaribact...    78  
1e-13

gi|17983284|gb|AAL52477.1| GUANOSINE-3',5'-BIS(DIPHOSPHATE) 3'-P...    78  
1e-13

gi|42572809|ref|NP\_974501.1| RSH1 (RELA-SPOT HOMOLOG); ATP bindi...    78  
1e-13

gi|86133427|ref|ZP\_01052009.1| GTP pyrophosphokinase [Tenacibacu...    78  
1e-13

gi|76797936|ref|ZP\_00780197.1| (p)ppGpp synthetase [Streptococcu...    78  
1e-13

gi|42570124|ref|NP\_849287.2| RSH1 (RELA-SPOT HOMOLOG); ATP bindi...    78  
1e-13

gi|18411893|ref|NP\_567226.1| RSH1 (RELA-SPOT HOMOLOG); ATP bindi...    78  
1e-13

gi|76797241|ref|ZP\_00779574.1|
RelA/SpoT protein [Thermoanaeroba...   
78   1e-13

gi|66877207|ref|ZP\_00402836.1|
COG0317: Guanosine polyphosphate ...   
78   1e-13

gi|88809826|ref|ZP\_01125332.1|
metal dependent phosphohydrolase ...   
78   1e-13

gi|15459146|gb|AAL00291.1|
GTP pyrophosphokinase [Streptococcus ...   
78   1e-13

gi|14973135|gb|AAK75725.1|
GTP pyrophosphokinase [Streptococcus ...   
78   1e-13

gi|23347449|gb|AAN29581.1|
RelA/SpoT family protein [Brucella su...   
77   1e-13

gi|62195744|gb|AAX74044.1|
RelA/SpoT family protein [Brucella ab...   
77   1e-13

gi|47573564|ref|ZP\_00243602.1|
COG0317: Guanosine polyphosphate ...    77   1e-13

gi|72119570|gb|AAZ61833.1| RelA/SpoT protein [Ralstonia eutropha...    77  
1e-13

gi|7268986|emb|CAB80719.1| putative GTP pyrophosphokinase [Arabi...    77  
1e-13

gi|83367851|ref|ZP\_00912718.1| RelA/SpoT protein [Rhodobacter sp...    77  
2e-13

gi|77686431|ref|ZP\_00801781.1| RelA/SpoT protein [Alkaliphilus m...    77  
2e-13

gi|77411860|ref|ZP\_00788193.1| GTP pyrophosphokinase family prot...    77  
2e-13

gi|76563219|gb|ABA45803.1| GTP pyrophosphokinase [Streptococcus ...    77  
2e-13

gi|77414296|ref|ZP\_00790454.1| GTP pyrophosphokinase family prot...    77  
2e-13

gi|56604411|emb|CAG45441.1| Guanosine-3',5'-bis(Diphosphate) 3'-...    77   2e-13

gi|71037904|gb|AAZ18212.1|
putative RelA/SpoT family protein [Ps...   
77   2e-13

gi|93005207|ref|YP\_579644.1|
(p)ppGpp synthetase I (GTP pyrophos...   
77   2e-13

gi|103488154|ref|YP\_617715.1|
(p)ppGpp synthetase I, SpoT/RelA [...   
77   2e-13

gi|3309186|gb|AAC26021.1|
RelA protein [Streptomyces antibioticu...   
77   2e-13

gi|89256706|ref|YP\_514068.1|
Guanosine-3',5'-bis(Diphosphate) 3'...   
77   2e-13

gi|40063134|gb|AAR37981.1|
GTP pyrophosphokinase [uncultured bac...   
77   2e-13

gi|83648950|ref|YP\_437385.1|
Guanosine polyphosphate pyrophospho...   
77   2e-13

gi|62261561|gb|AAX77998.1|
unknown protein [synthetic construct]     
 77   2e-13

gi|89900856|ref|YP\_523327.1|
(p)ppGpp synthetase I (GTP pyrophos...   
77   2e-13

gi|91771351|ref|ZP\_01273157.1|
Amino acid-binding ACT:TGS [Psych...   
77   2e-13

gi|89093832|ref|ZP\_01166778.1|
guanosine-3,5-bis(diphosphate) 3-...   
77   2e-13

gi|88949942|ref|ZP\_01152555.1|
RelA/SpoT protein [Halorhodospira...   
77   2e-13

gi|90415473|ref|ZP\_01223407.1|
guanosine-3',5'-bis(diphosphate) ...   
77   2e-13

gi|90573535|ref|ZP\_01230048.1|
hypothetical protein CdifQ\_020030...   
77   2e-13

gi|89053005|ref|YP\_508456.1|
(p)ppGpp synthetase I (GTP pyrophos...   
77   2e-13

gi|14027197|dbj|BAB54151.1|
GTP pyrophosphokinase [Mesorhizobium...   
77   2e-13

gi|34762466|ref|ZP\_00143465.1|
Guanosine-3',5'-bis(Diphosphate) ...   
77   2e-13

gi|84684014|ref|ZP\_01011916.1|
RelA/SpoT family protein [Rhodoba...   
77   2e-13

gi|67154923|ref|ZP\_00416668.1|
RelA/SpoT protein [Azotobacter vi...   
77   2e-13

gi|48856298|ref|ZP\_00310455.1|
COG0317: Guanosine polyphosphate ...   
77   2e-13

gi|82744819|ref|ZP\_00907334.1|
RelA/SpoT protein [Clostridium be...   
77   2e-13

gi|89900130|ref|YP\_522601.1|
threonyl-tRNA synthetase [Rhodofera...   
77   2e-13

gi|68547060|ref|ZP\_00586602.1|
RelA/SpoT protein [Shewanella ama...   
77   2e-13

gi|78700884|ref|ZP\_00865338.1|
Threonyl-tRNA synthetase, class I...   
77   2e-13

gi|76794711|ref|ZP\_00777172.1|
RelA/SpoT protein [Pseudoalteromo...   
77   2e-13

gi|81428351|ref|YP\_395351.1|
GTP pyrophosphokinase [Lactobacillu...   
77   2e-13

gi|45656898|ref|YP\_000984.1|
guanosine polyphosphate pyrophospho...   
77   2e-13

gi|63254186|gb|AAY35282.1|
RelA/SpoT protein [Pseudomonas syring...   
77   2e-13

gi|94495624|ref|ZP\_01302204.1|
threonyl-tRNA synthetase [Sphingo...   
77   2e-13

gi|3377803|gb|AAC28176.1|
T2H3.9 [Arabidopsis thaliana]               
77   2e-13

gi|7379634|emb|CAB84201.1|
putative threonyl-tRNA synthetase [Ne...   
77   2e-13

gi|82739885|ref|ZP\_00902666.1| RelA/SpoT protein [Pseudomonas pu...    77  
3e-13

gi|67874585|ref|ZP\_00504295.1| Threonyl-tRNA synthetase, class I...    77  
3e-13

gi|26991978|ref|NP\_747403.1| guanosine-3',5'-bis(diphosphate) 3'...    77  
3e-13

gi|71557356|gb|AAZ36567.1| guanosine-3,5-bis(diphosphate) 3-pyro...    77  
3e-13

gi|28850547|gb|AAO53627.1| guanosine-3,5-bis(diphosphate) 3-pyro...    77  
3e-13

gi|24215784|ref|NP\_713265.1| GTP pyrophosphokinase [Leptospira i...    77  
3e-13

gi|70733346|ref|YP\_263121.1| guanosine-3,5-bis(diphosphate) 3-py...    77  
3e-13

gi|71909454|ref|YP\_287041.1| RelA/SpoT protein [Dechloromonas ar...    77  
3e-13

gi|78170000|gb|ABB27097.1| metal dependent phosphohydrolase [Syn...    77  
3e-13

gi|104784343|ref|YP\_610841.1| guanosine-3',5'-bis(diphosphate) 3...    77  
3e-13

gi|77385775|gb|ABA77288.1| (p)ppGpp synthetase I (GTP pyrophosph...    77  
3e-13

gi|85711933|ref|ZP\_01042988.1| (p)ppGpp synthetase I [Idiomarina...    77  
3e-13

gi|78366934|ref|ZP\_00837210.1| RelA/SpoT protein [Shewanella sp....    77  
3e-13

gi|52307843|gb|AAU38343.1| SpoT protein [Mannheimia succinicipro...    77  
3e-13

gi|28271464|emb|CAD64369.1| GTP pyrophosphokinase [Lactobacillus...    77  
3e-13

gi|56807780|ref|ZP\_00365635.1| COG0317: Guanosine polyphosphate ...    76  
3e-13

gi|34541290|ref|NP\_905769.1| RelA/SpoT family protein [Porphyrom...    76  
3e-13

gi|56381096|dbj|BAD77004.1| threonyl-tRNA synthetase [Geobacillu...    76  
3e-13

gi|83590594|ref|YP\_430603.1| threonyl-tRNA synthetase [Moorella ...    76   3e-13

gi|72003244|gb|AAZ59046.1|
RelA/SpoT protein [Prochlorococcus ma...   
76   4e-13

gi|33866854|ref|NP\_898413.1|
guanosine-3',5'-bis(diphosphate) 3'...   
76   4e-13

gi|94991264|ref|YP\_599364.1|
GTP pyrophosphokinase / Guanosine-3...   
76   4e-13

gi|21905450|gb|AAM80308.1|
(p)ppGpp synthetase [Streptococcus py...   
76   4e-13

gi|47575500|ref|ZP\_00245535.1|
COG0441: Threonyl-tRNA synthetase...   
76   4e-13

gi|93007241|ref|YP\_581678.1|
threonyl-tRNA synthetase [Psychroba...   
76   4e-13

gi|19715157|gb|AAL95675.1|
Guanosine-3',5'-bis(Diphosphate) 3'-p...   
76   4e-13

gi|29343960|gb|AAO81720.1|
GTP pyrophosphokinase [Enterococcus f...   
76   4e-13

gi|22087659|gb|AAM90994.1|
ppGpp [Fusobacterium nucleatum]            
76   4e-13

gi|57637823|gb|AAW54611.1|
threonyl-tRNA synthetase [Staphylococ...   
76   4e-13

gi|38258358|sp|Q8CS74|SYT\_STAES
Threonyl-tRNA synthetase (Threon...    76   4e-13

gi|7225946|gb|AAF41133.1| threonyl-tRNA synthetase [Neisseria me...    76  
4e-13

gi|19749128|gb|AAL98519.1| (p)ppGpp synthetase [Streptococcus py...    76  
4e-13

gi|28211884|ref|NP\_782828.1| threonyl-tRNA synthetase [Clostridi...    76  
4e-13

gi|407881|emb|CAA51353.1| stringent response-like protein [Strep...    76  
4e-13

gi|94995176|ref|YP\_603274.1| GTP pyrophosphokinase / Guanosine-3...    76  
4e-13

gi|91770061|ref|ZP\_01271890.1| Threonyl-tRNA synthetase, class I...    76  
4e-13

gi|71803431|gb|AAX72784.1| GTP pyrophosphokinase [Streptococcus ...    76  
4e-13

gi|94993206|ref|YP\_601305.1| GTP pyrophosphokinase / Guanosine-3...    76  
4e-13

gi|77966326|gb|ABB07706.1| (p)ppGpp synthetase I (GTP pyrophosph...    76  
4e-13

gi|28811868|dbj|BAC64797.1| putative (p)ppGpp synthetase [Strept...    76  
4e-13

gi|50904114|gb|AAT87829.1| GTP pyrophosphokinase; Guanosine-3',5...    76  
4e-13

gi|74015927|ref|ZP\_00686554.1| RelA/SpoT protein [Burkholderia a...    76  
4e-13

gi|71854281|gb|AAZ52304.1| GTP pyrophosphokinase/guanosine-3',5'...    76  
4e-13

gi|107022080|ref|YP\_620407.1| (p)ppGpp synthetase I, SpoT/RelA [...    76  
4e-13

gi|13622996|gb|AAK34667.1| (p)ppGpp synthetase [Streptococcus py...    76  
4e-13

gi|89892986|ref|YP\_516473.1| hypothetical protein DSY0240 [Desul...    76  
4e-13

gi|88934227|ref|ZP\_01139871.1| Threonyl-tRNA synthetase, class I...    76  
4e-13

gi|91784884|ref|YP\_560090.1| (p)ppGpp synthetase I (GTP pyrophos...    76  
4e-13

gi|84361066|ref|ZP\_00985749.1| COG0317: Guanosine polyphosphate ...    76  
4e-13

gi|83719914|ref|YP\_442129.1| guanosine-3`,5`-bis(diphosphate) 3`...    76  
4e-13

gi|52429441|gb|AAU50034.1| guanosine-3`,5`-bis(diphosphate) 3`-p...    76  
4e-13

gi|100917740|ref|ZP\_01346217.1| hypothetical protein Bmal10\_0300...    76  
4e-13

gi|87123541|ref|ZP\_01079392.1| guanosine-3',5'-bis(diphosphate) ...    76  
5e-13

gi|106895755|ref|ZP\_01362826.1| RelA/SpoT protein [Clostridium s...    76  
5e-13

gi|83951638|ref|ZP\_00960370.1| guanosine-3',5'-bis(Diphosphate) ...    76  
5e-13

gi|76883034|gb|ABA57715.1| RelA/SpoT protein [Nitrosococcus ocea...    76  
5e-13

gi|29338004|gb|AAO75807.1| GTP pyrophosphokinase [Bacteroides th...    76  
5e-13

gi|82751271|ref|YP\_417012.1| threonyl-tRNA synthetase 1 [Staphyl...    75  
5e-13

gi|78193878|gb|ABB31645.1| Threonyl-tRNA synthetase, class IIa [...    75  
5e-13

gi|87300813|ref|ZP\_01083655.1| metal dependent phosphohydrolase ...    75  
6e-13

gi|32562922|dbj|BAC79235.1| guanosine-3', 5'-pyrophosphohydrolas...    75  
6e-13

gi|84709909|ref|ZP\_01018844.1| RelA/SpoT protein [Polaromonas na...    75  
6e-13

gi|84518744|ref|ZP\_01006093.1| GTP pyrophosphokinase [Prochloroc...    75  
6e-13

gi|69950142|ref|ZP\_00638074.1| RelA/SpoT protein [Shewanella fri...    75  
6e-13

gi|55247283|gb|EAA02099.2| ENSANGP00000014602 [Anopheles gambiae...    75  
6e-13

gi|59710711|ref|YP\_203487.1| GTP pyrophosphokinase [Vibrio fisch...    75  
6e-13

gi|49238105|emb|CAF27312.1| GTP pyrophosphokinase [Bartonella he...    75  
6e-13

gi|24371957|ref|NP\_715999.1| guanosine-3',5'-bis(diphosphate) 3'...    75  
6e-13

gi|77382161|gb|ABA73674.1| Threonyl-tRNA synthetase, class IIa [...    75  
6e-13

gi|91794782|ref|YP\_564433.1|
RelA/SpoT family protein [Shewanell...   
75   6e-13

gi|47716787|gb|AAT37571.1|
RelA [Sinorhizobium meliloti] >gi|150...   
75   7e-13

gi|49239565|emb|CAF25922.1|
GTP pyrophosphokinase [Bartonella qu...   
75   7e-13

gi|50876054|emb|CAG35894.1|
probable GTP pyrophosphokinase [Desu...   
75   7e-13

gi|83311352|ref|YP\_421616.1|
Guanosine polyphosphate pyrophospho...   
75   7e-13

gi|78689356|ref|ZP\_00854019.1|
RelA/SpoT protein [Shewanella sp....   
75   7e-13

gi|73662391|ref|YP\_301172.1|
threonyl-tRNA synthetase [Staphyloc...   
75   7e-13

gi|82495673|ref|ZP\_00881247.1|
RelA/SpoT protein [Shewanella sp....   
75   7e-13

gi|78685911|ref|ZP\_00850685.1|
RelA/SpoT protein [Shewanella sp....   
75   7e-13

gi|77813466|ref|ZP\_00812741.1|
RelA/SpoT protein [Shewanella put...   
75   7e-13

gi|23014395|ref|ZP\_00054214.1|
COG0317: Guanosine polyphosphate ...   
75   7e-13

gi|90412683|ref|ZP\_01220684.1|
putative guanosine-3,5-bis(diphos...   
75   8e-13

gi|46911830|emb|CAG18628.1|
putative guanosine-3,5-bis(diphospha...   
75   8e-13

gi|85059397|ref|YP\_455099.1|
threonyl-tRNA synthase [Sodalis glo...   
75   8e-13

gi|68541173|ref|ZP\_00580919.1|
RelA/SpoT protein [Shewanella bal...   
75   8e-13

gi|69935867|ref|ZP\_00630754.1|
RelA/SpoT protein [Paracoccus den...   
75   9e-13

gi|33641155|emb|CAE22285.1|
guanosine-3',5'-bis(diphosphate) 3'-...   
75   9e-13

gi|46914621|emb|CAG21398.1|
putative GTP pyrophosphokinase [Phot...   
75   9e-13

gi|90412093|ref|ZP\_01220100.1|
putative GTP pyrophosphokinase [P...   
75   9e-13

gi|53805116|ref|YP\_113204.1|
threonyl-tRNA synthetase [Methyloco...   
75   9e-13

gi|90023337|ref|YP\_529164.1|
GTP diphosphokinase [Saccharophagus...   
75   1e-12

gi|49609527|emb|CAG72960.1|
guanosine-3',5'-bis(diphosphate) 3'-...   
75   1e-12

gi|56679772|gb|AAV96438.1|
guanosine-3',5'-bis(Diphosphate) 3'-p...   
75   1e-12

gi|90820664|gb|ABD99303.1|
Threonyl-tRNA synthetase [Lactobacill...   
75   1e-12

gi|71145011|gb|AAZ25484.1|
guanosine-3,5-bis(diphosphate) 3-pyro...   
75   1e-12

gi|82743592|ref|ZP\_00906229.1|
RelA/SpoT protein [Shewanella sp....    75  
1e-12

gi|53803680|ref|YP\_114455.1|
guanosine-3,5-bis(diphosphate) 3-py...   
75   1e-12

gi|56542556|gb|AAV88710.1|
guanosine polyphosphate pyrophosphohy...   
75   1e-12

gi|8163820|gb|AAF73865.1|
pentaphosphate guanosine-3'-pyrophosph...   
75   1e-12

gi|68233514|ref|ZP\_00572632.1|
RelA/SpoT protein [Frankia sp. EA...   
74   1e-12

gi|33237194|gb|AAP99263.1|
GTP pyrophosphokinase [Prochlorococcu...   
74   1e-12

gi|52002559|gb|AAU22501.1|
threonyl-tRNA synthetase [Bacillus li...   
74   1e-12

gi|58582810|ref|YP\_201826.1|
threonyl-tRNA synthetase [Xanthomon...   
74   1e-12

gi|39983496|gb|AAR34889.1|
threonyl-tRNA synthetase [Geobacter s...   
74   1e-12

gi|67940005|ref|ZP\_00532480.1|
Threonyl-tRNA synthetase, class I...   
74   1e-12

gi|102191532|ref|ZP\_01347344.1|
hypothetical protein RcanM\_01000...   
74   1e-12

gi|57637787|gb|AAW54575.1|
GTP pyrophosphokinase [Staphylococcus...   
74   1e-12

gi|88861112|ref|ZP\_01135746.1|
bifunctional: (p)ppGpp synthetase...   
74   1e-12

gi|2316008|gb|AAC45548.1|
(p)ppGpp 3'-pyrophosphohydrolase [Spir...   
74   1e-12

gi|106887107|ref|ZP\_01354426.1|
RelA/SpoT family protein [Clostr...   
74   1e-12

gi|78198422|gb|ABB36187.1|
metal dependent phosphohydrolase [Syn...   
74   2e-12

gi|85709956|ref|ZP\_01041021.1|
threonyl-tRNA synthetase [Erythro...   
74   2e-12

gi|76793846|ref|ZP\_00776327.1|
RelA/SpoT protein [Pseudoalteromo...   
74   2e-12

gi|60493136|emb|CAH07917.1|
GTP pyrophosphokinase [Bacteroides f...   
74   2e-12

gi|77408338|ref|ZP\_00785079.1|
threonyl-tRNA synthetase [Strepto...   
74   2e-12

gi|82751232|ref|YP\_416973.1|
GTP pyrophosphokinase [Staphylococc...   
74   2e-12

gi|82523710|emb|CAI78491.1|
guanosine polyphosphate pyrophosphoh...   
74   2e-12

gi|23095093|emb|CAD46328.1|
theronyl-tRNA synthetase [Streptococ...   
74   2e-12

gi|76562861|gb|ABA45445.1|
threonyl-tRNA synthetase [Streptococc...   
74   2e-12

gi|49483878|ref|YP\_041102.1|
GTP pyrophosphokinase [Staphylococc...   
74   2e-12

gi|2635360|emb|CAB14855.1|
threonyl-tRNA synthetase [Bacillus su...   
74   2e-12

gi|88195445|ref|YP\_500249.1|
GTP pyrophosphokinase [Staphylococc...   
74   2e-12

gi|84624684|ref|YP\_452056.1|
threonyl-tRNA synthetase [Xanthomon...   
74   2e-12

gi|21204753|dbj|BAB95449.1|
GTP pyrophosphokinase [Staphylococcu...  
 74   2e-12

gi|49244910|emb|CAG43371.1|
GTP pyrophosphokinase [Staphylococcu...   
74   2e-12

gi|85711636|ref|ZP\_01042693.1|
threonyl-tRNA synthetase [Idiomar...   
74   2e-12

gi|14247406|dbj|BAB57796.1|
GTP pyrophosphokinase [Staphylococcu...   
74   2e-12

gi|88792790|ref|ZP\_01108508.1|
bifunctional (p)ppGpp synthetase ...   
74   2e-12

gi|59798426|sp|Q931Q4|RELA\_STAAM
GTP pyrophosphokinase (ATP:GTP ...   
74   2e-12

gi|70729481|ref|YP\_259219.1|
threonyl-tRNA synthetase [Pseudomon...   
74   2e-12

gi|9828169|gb|AAG00076.1|
SpoT [Candidatus Phlomobacter fragariae]    
74   2e-12

gi|39721721|dbj|BAD04211.1|
guanosine polyphosphate pyrophosphoh...   
74   2e-12

gi|68447012|dbj|BAE04596.1|
GTP pyrophosphokinase (ATP:GTP 3&apo...   
74   2e-12

gi|30268666|dbj|BAC76004.1|
RelA-SpoT like protein RSH1 [Nicotia...   
74   2e-12

gi|94310105|ref|YP\_583315.1|
threonyl-tRNA synthetase [Ralstonia...   
74   2e-12

gi|83595865|gb|ABC25227.1|
guanosine-3'''',5''''-bis(diphosphate...   
74   2e-12

gi|46913777|emb|CAG20559.1|
putative threonyl-tRNA synthetase [P...   
74   2e-12

gi|46143480|ref|ZP\_00135156.2|
COG0317: Guanosine polyphosphate ...   
74   2e-12

gi|74316489|ref|YP\_314229.1|
Metal dependent phosphohydrolase, H...   
74   2e-12

gi|90410628|ref|ZP\_01218644.1|
threonyl-tRNA synthetase [Photoba...   
74   2e-12

gi|94985607|ref|YP\_604971.1|
threonyl-tRNA synthetase [Deinococc...   
74   2e-12

gi|83319815|ref|YP\_424469.1|
RelA/SpoT family protein [Mycoplasm...   
74   3e-12

gi|85374708|ref|YP\_458770.1|
guanosine polyphosphate pyrophospho...   
74   3e-12

gi|98662355|dbj|GAA00906.1|
unnamed protein product [Pelotomacul...   
74   3e-12

gi|63256109|gb|AAY37205.1|
Threonyl-tRNA synthetase, class IIa [...   
73   3e-12

gi|94967206|ref|YP\_589254.1|
(p)ppGpp synthetase I (GTP pyrophos...   
73   3e-12

gi|52698608|ref|ZP\_00340016.1|
COG0441: Threonyl-tRNA synthetase...   
73   3e-12

gi|90581393|ref|ZP\_01237188.1|
putative guanosine-3,5-bis(diphos...   
73   3e-12

gi|89076057|ref|ZP\_01162417.1|
putative guanosine-3,5-bis(diphos...   
73   3e-12

gi|49483925|ref|YP\_041149.1|
threonyl-tRNA synthetase [Staphyloc...   
73   3e-12

gi|88195489|ref|YP\_500293.1|
threonyl-tRNA synthetase [Staphyloc...   
73   3e-12

gi|15156050|gb|AAK86838.1|
AGR\_C\_1896p [Agrobacterium tumefacien...  
 73   3e-12

gi|12724948|gb|AAK06009.1|
theronyl-tRNA synthetase (EC 6.1.1.3)...   
73   3e-12

gi|84690856|gb|EAQ16696.1|
Threonyl-tRNA synthetase [Parvularcul...   
73   3e-12

gi|55738527|gb|AAV62168.1|
threonyl-tRNA synthetase 1 [Streptoco...   
73   3e-12

gi|41323484|gb|AAR99902.1|
RelA [Agrobacterium tumefaciens]           
73   3e-12

gi|75815743|ref|ZP\_00746237.1|
COG0317: Guanosine polyphosphate ...   
73   3e-12

gi|75824742|ref|ZP\_00754188.1|
COG0317: Guanosine polyphosphate ...   
73   3e-12

gi|91798440|gb|ABE60579.1|
(p)ppGpp synthetase I (GTP pyrophosph...   
73   3e-12

gi|75820609|ref|ZP\_00750652.1|
COG0317: Guanosine polyphosphate ...   
73   3e-12

gi|83816191|ref|YP\_446893.1|
threonyl-tRNA synthetase [Salinibac...   
73   3e-12

gi|77919019|ref|YP\_356834.1|
threonyl-tRNA synthetase [Pelobacte...   
73   3e-12

gi|104781106|ref|YP\_607604.1|
threonyl-tRNA synthetase [Pseudomo...   
73   3e-12

gi|75830635|ref|ZP\_00759909.1|
COG0317: Guanosine polyphosphate ...   
73   3e-12

gi|71802176|gb|AAX71529.1|
threonyl-tRNA synthetase [Streptococc...   
73   3e-12

gi|94991943|ref|YP\_600042.1|
Threonyl-tRNA synthetase [Streptoco...   
73   3e-12

gi|21904088|gb|AAM78972.1|
putative threonyl-tRNA synthetase [St...   
73   3e-12

gi|94989937|ref|YP\_598037.1|
Threonyl-tRNA synthetase [Streptoco...   
73   3e-12

gi|94993836|ref|YP\_601934.1|
Threonyl-tRNA synthetase [Streptoco...   
73   3e-12

gi|71853022|gb|AAZ51045.1|
threonyl-tRNA synthetase [Streptococc...   
73   3e-12

gi|9657306|gb|AAF95850.1|
guanosine-3',5'-bis(diphosphate) 3'-py...   
73   3e-12

gi|56180490|gb|AAV83212.1|
(p)ppGpp synthetase I; guanosine-3',5...   
73   3e-12

gi|82543879|ref|YP\_407826.1|
threonine tRNA synthetase [Shigella...   
73   4e-12

gi|43066|emb|CAA23560.1|
unnamed protein product [Escherichia coli]   
73   4e-12

gi|17739420|gb|AAL42043.1|
GTP pyrophosphohydrolases/synthetases...   
73   4e-12

gi|24051829|gb|AAN43102.1|
threonine tRNA synthetase [Shigella f...   
73   4e-12

gi|15831680|ref|NP\_310453.1|
threonyl-tRNA synthetase [Escherich...   
73   4e-12

gi|86131542|ref|ZP\_01050140.1|
threonyl-tRNA synthetase [Cellulo...   
73   4e-12

gi|77975251|ref|ZP\_00830787.1|
COG0441: Threonyl-tRNA synthetase...   
73   4e-12

gi|10175763|dbj|BAB06860.1|
threonyl-tRNA synthetase 1 [Bacillus...   
73   4e-12

gi|82498615|ref|ZP\_00884096.1|
Threonyl-tRNA synthetase, class I...   
73   4e-12

gi|78692360|ref|ZP\_00856900.1|
Threonyl-tRNA synthetase, class I...   
73   4e-12

gi|50902874|gb|AAT86589.1|
Threonyl-tRNA synthetase [Streptococc...  
 73   4e-12

gi|45436874|gb|AAS62427.1|
threonyl-tRNA synthetase [Yersinia pe...   
73   4e-12

gi|75429917|ref|ZP\_00732514.1|
Guanosine-3',5'-bis(diphosphate) ...   
73   4e-12

gi|59800743|ref|YP\_207455.1|
putative threonyl-tRNA synthetase [...   
73   4e-12

gi|12583661|dbj|BAB21484.1|
chloroplast RelA homologue 1 [Oryza ...   
73   4e-12

gi|71481942|ref|ZP\_00661644.1|
Threonyl-tRNA synthetase, class I...   
73   4e-12

gi|66864711|gb|AAY57436.1|
RelA [Rhizobium etli]                      
73   4e-12

gi|86740083|ref|YP\_480483.1|
(p)ppGpp synthetase I [Frankia sp. ...   
73   4e-12

gi|28852817|gb|AAO55889.1|
threonyl-tRNA synthetase [Pseudomonas...   
72   5e-12

gi|687792|gb|AAC43729.1|
putative threonyl-tRNA synthetase; simi...   
72   5e-12

gi|21113622|gb|AAM41739.1|
threonyl-tRNA synthetase [Xanthomonas...   
72   5e-12

gi|86357023|ref|YP\_468915.1|
guanosine-3`,5`-bis(diphosphate) 3`...   
72   5e-12

gi|14973120|gb|AAK75711.1|
threonyl-tRNA synthetase [Streptococc...   
72   5e-12

gi|50541737|gb|AAT78347.1|
RelA [Rhizobium etli]                      
72   5e-12

gi|73621971|sp|Q8DNX8|SYT\_STRR6
Threonyl-tRNA synthetase (Threon...   
72   5e-12

gi|15459130|gb|AAL00276.1|
Threonyl-tRNA synthetase 1 [Streptoco...   
72   5e-12

gi|42561010|ref|NP\_975461.1|
GTP diphosphokinase [Mycoplasma myc...   
72   5e-12

gi|90420858|ref|ZP\_01228763.1|
GTP pyrophosphokinase, Guanosine-...   
72   5e-12

gi|27360418|gb|AAO09355.1|
Guanosine-3',5'-bis(diphosphate) 3'-p...   
72   5e-12

gi|16760562|ref|NP\_456179.1|
threonyl-tRNA synthetase [Salmonell...   
72   5e-12

gi|56127938|gb|AAV77444.1|
threonyl-tRNA synthetase [Salmonella ...   
72   5e-12

gi|91224995|ref|ZP\_01260254.1|
guanosine-3',5'-bis(diphosphate) ...   
72   5e-12

gi|78048351|ref|YP\_364526.1|
threonyl-tRNA synthetase [Xanthomon...   
72   5e-12

gi|22654130|sp|Q97GK4|SYT\_CLOAB
Threonyl-tRNA synthetase (Threon...   
72   6e-12

gi|75855788|ref|ZP\_00763430.1|
COG0317: Guanosine polyphosphate ...   
72   6e-12

gi|21108867|gb|AAM37443.1|
threonyl-tRNA synthetase [Xanthomonas...   
72   6e-12

gi|37678425|ref|NP\_933034.1|
guanosine-3',5'-bis(diphosphate) 3'...   
72   6e-12

gi|56179517|gb|AAV82239.1|
Threonyl-tRNA synthetase [Idiomarina ...   
72   6e-12

gi|83855584|ref|ZP\_00949113.1|
threonyl-tRNA synthetase [Croceib...    72  
6e-12

gi|89199764|ref|ZP\_01178521.1|
Threonyl-tRNA synthetase, class I...   
72   6e-12

gi|16764684|ref|NP\_460299.1|
threonyl-tRNA synthetase [Salmonell...   
72   6e-12

gi|62462448|ref|ZP\_00381856.1|
COG0441: Threonyl-tRNA synthetase...   
72   6e-12

gi|89360914|ref|ZP\_01198731.1|
RelA/SpoT protein [Xanthobacter a...   
72   6e-12

gi|83647272|ref|YP\_435707.1|
threonyl-tRNA synthetase [Hahella c...   
72   6e-12

gi|28805141|dbj|BAC58422.1|
guanosine-3',5'-bis(diphosphate) 3'-...   
72   6e-12

gi|62179922|ref|YP\_216339.1|
threonyl-tRNA synthetase [Salmonell...   
72   6e-12

gi|84386373|ref|ZP\_00989401.1|
Guanosine-3',5'-bis(diphosphate) ...   
72   6e-12

gi|49532043|emb|CAG69755.1|
threonyl-tRNA synthetase [Acinetobac...   
72   6e-12

gi|26989188|ref|NP\_744613.1|
threonyl-tRNA synthetase [Pseudomon...   
72   6e-12

gi|56966073|pdb|1TKY|A
Chain A, Crystal Structure Of The Editing...   
72   7e-12

gi|85057876|ref|YP\_456792.1|
GTP pyrophosphokinase [Aster yellow...   
72   7e-12

gi|24379968|ref|NP\_721923.1|
threonyl-tRNA synthetase [Streptoco...   
72   7e-12

gi|86146550|ref|ZP\_01064872.1|
Guanosine-3',5'-bis(diphosphate) ...   
72   7e-12

gi|76664725|emb|CAJ17802.1|
guanosine polyphosphate pyrophosphoh...   
72   7e-12

gi|94499848|ref|ZP\_01306384.1|
guanosine-3',5'-bis(diphosphate) ...   
72   7e-12

gi|78712078|gb|ABB49255.1|
metal dependent phosphohydrolase [Pro...   
72   7e-12

gi|1174548|sp|P43014|SYT\_HAEIN
Threonyl-tRNA synthetase (Threoni...   
72   7e-12

gi|24373852|ref|NP\_717895.1|
threonyl-tRNA synthetase [Shewanell...   
72   7e-12

gi|67157280|ref|ZP\_00418582.1|
Threonyl-tRNA synthetase, class I...   
72   8e-12

gi|83751134|ref|ZP\_00947548.1|
COG0317: Guanosine polyphosphate ...   
72   8e-12

gi|85710706|ref|ZP\_01041770.1|
guanosine polyphosphate pyrophosp...    72   8e-12

gi|67930305|ref|ZP\_00523479.1| RelA/SpoT protein [Solibacter usi...    72  
8e-12

gi|68544838|ref|ZP\_00584428.1| Threonyl-tRNA synthetase, class I...    72  
8e-12

gi|71039637|gb|AAZ19945.1| threonyl-tRNA synthetase [Psychrobact...    72  
8e-12

gi|34580760|ref|ZP\_00142240.1| threonyl-tRNA synthetase [Rickett...    72  
8e-12

gi|59711821|ref|YP\_204597.1| threonyl-tRNA synthetase [Vibrio fi...    72  
9e-12

gi|95929501|ref|ZP\_01312244.1| metal dependent phosphohydrolase ...    72  
9e-12

gi|53692579|ref|ZP\_00347332.1| COG0317: Guanosine polyphosphate ...    72  
9e-12

gi|50363650|gb|AAT75635.1| guanosine-3',5'-bis(diphosphate)3'-py...    72  
9e-12

gi|91068894|gb|ABE04616.1| Threonyl-tRNA synthetase [Rickettsia ...    72  
1e-11

gi|12721239|gb|AAK03004.1| SpoT [Pasteurella multocida subsp. mu...    72  
1e-11

gi|88811934|ref|ZP\_01127187.1| guanosine-3',5'-bis(diphosphate) ...    72  
1e-11

gi|78364767|gb|ABB42732.1| (p)ppGpp synthetase I (GTP pyrophosph...    72  
1e-11

gi|23023554|ref|ZP\_00062788.1| COG0317: Guanosine polyphosphate ...    72  
1e-11

gi|33152904|ref|NP\_874257.1| guanosine-3',5'-bis(diphosphate) 3'...    72  
1e-11

gi|51573029|gb|AAU07054.1| guanosine-3,5-bis(diphosphate) 3-pyro...    71  
1e-11

gi|82777067|ref|YP\_403416.1| threonine tRNA synthetase [Shigella...    71  
1e-11

gi|88949485|ref|ZP\_01152102.1| Threonyl-tRNA synthetase, class I...    71  
1e-11

gi|27802072|gb|AAL71859.1| (p)ppGpp synthase/pyrophosphohydrolas...    71  
1e-11

gi|33633693|emb|CAE18650.1| guanosine-3',5'-bis(diphosphate) 3'-...    71  
1e-11

gi|27802074|gb|AAL71860.1| (p)ppGpp synthase/pyrophosphohydrolas...    71  
1e-11

gi|12720863|gb|AAK02677.1| ThrS [Pasteurella multocida subsp. mu...    71  
1e-11

gi|67004904|gb|AAY61830.1| Threonyl-tRNA synthetase [Rickettsia ...    71  
1e-11

gi|36785991|emb|CAE15043.1| threonyl-tRNA synthetase (threonine-...    71  
1e-11

gi|28199582|ref|NP\_779896.1| pentaphosphate guanosine-3'-pyropho...    71  
1e-11

gi|42781481|ref|NP\_978728.1| threonyl-tRNA synthetase [Bacillus ...    71  
1e-11

gi|51976562|gb|AAU18112.1| threonine--tRNA ligase (threonyl-tRNA...    71  
1e-11

gi|91797012|gb|ABE59151.1| threonyl-tRNA synthetase [Chromohalob...    71  
1e-11

gi|51459731|gb|AAU03694.1| threonine--tRNA ligase; Threonine tra...    71  
1e-11

gi|91787975|ref|YP\_548927.1| threonyl-tRNA synthetase [Polaromon...    71  
1e-11

gi|106882482|ref|ZP\_01349884.1| threonyl-tRNA synthetase [Psychr...    71  
1e-11

gi|67927927|ref|ZP\_00521117.1| Threonyl-tRNA synthetase, class I...    71  
1e-11

gi|77815770|ref|ZP\_00814990.1| Threonyl-tRNA synthetase, class I...    71  
1e-11

gi|75429625|ref|ZP\_00732336.1| threonyl-tRNA synthetase [Actinob...    71  
1e-11

gi|82744236|ref|ZP\_00906819.1| Threonyl-tRNA synthetase, class I...    71  
1e-11

gi|71898963|ref|ZP\_00681129.1| RelA/SpoT protein [Xylella fastid...    71  
1e-11

gi|42631665|ref|ZP\_00157203.1| COG0441: Threonyl-tRNA synthetase...    71  
1e-11

gi|91977118|ref|YP\_569777.1| RelA/SpoT family protein [Rhodopseu...    71  
1e-11

gi|90424134|ref|YP\_532504.1| (p)ppGpp synthetase I (GTP pyrophos...    71  
1e-11

gi|68058317|gb|AAX88570.1| threonyl-tRNA synthetase [Haemophilus...    71  
1e-11

gi|88793221|ref|ZP\_01108937.1| threonyl-tRNA synthetase [Alterom...    71  
1e-11

gi|32030756|ref|ZP\_00133531.1| COG0441: Threonyl-tRNA synthetase...    71  
1e-11

gi|72118383|gb|AAZ60646.1| Threonyl-tRNA synthetase, class IIa [...    71  
1e-11

gi|91220685|ref|ZP\_01256941.1| GTP pyrophosphokinase [Psychrofle...    71  
1e-11

gi|23466698|ref|ZP\_00122285.1| COG0441: Threonyl-tRNA synthetase...    71  
2e-11

gi|82735019|ref|ZP\_00897882.1| Threonyl-tRNA synthetase, class I...    71  
2e-11

gi|49331498|gb|AAT62144.1| threonine--tRNA ligase (threonyl-tRNA...    71  
2e-11

gi|6689424|emb|CAB65483.1| threonyl-tRNA synthetase [Thermus the...    71  
2e-11

gi|46199818|ref|YP\_005485.1| threonyl-tRNA synthetase [Thermus t...    71  
2e-11

gi|47502829|gb|AAT31505.1| threonyl-tRNA synthetase [Bacillus an...    71  
2e-11

gi|58255124|gb|AAV43361.1| theronyl-tRNA synthetase [Lactobacill...    71  
2e-11

gi|52307160|gb|AAU37660.1| ThrS protein [Mannheimia succinicipro...    71  
2e-11

gi|9948822|gb|AAG06132.1| threonyl-tRNA synthetase [Pseudomonas ...    71  
2e-11

gi|106883573|ref|ZP\_01350968.1| RelA/SpoT family protein [Psychr...    71  
2e-11

gi|39884|emb|CAA28636.1| unnamed protein product [Bacillus subti...    70  
2e-11

gi|71556384|gb|AAZ35595.1| threonyl-tRNA synthetase [Pseudomonas...    70  
2e-11

gi|86749726|ref|YP\_486222.1| (p)ppGpp synthetase I [Rhodopseudom...    70  
2e-11

gi|82747912|ref|ZP\_00910404.1| Threonyl-tRNA synthetase, class I...    70  
2e-11

gi|90407011|ref|ZP\_01215201.1| threonyl-tRNA synthetase [Psychro...    70  
2e-11

gi|77980144|ref|ZP\_00835559.1| COG0441: Threonyl-tRNA synthetase...    70  
2e-11

gi|42453437|ref|ZP\_00153344.1| COG0441: Threonyl-tRNA synthetase...    70   2e-11

gi|91087799|ref|XP\_967345.1|
PREDICTED: similar to CG5353-PA, is...   
70   2e-11

gi|53728893|ref|ZP\_00134603.2|
COG0441: Threonyl-tRNA synthetase...   
70   2e-11

gi|57167616|ref|ZP\_00366756.1|
penta-phosphate guanosine-3'-pyro...   
70   2e-11

gi|20516726|gb|AAM24913.1|
Threonyl-tRNA synthetase [Thermoanaer...   
70   2e-11

gi|77959152|ref|ZP\_00823175.1|
COG0441: Threonyl-tRNA synthetase...    70  
2e-11

gi|13422940|gb|AAK23532.1|
guanosine-3',5'-bis(diphosphate) 3'-p...   
70   2e-11

gi|77962042|ref|ZP\_00825868.1|
COG0441: Threonyl-tRNA synthetase...   
70   2e-11

gi|57505447|ref|ZP\_00371375.1|
penta-phosphate guanosine-3'-pyro...   
70   2e-11

gi|83858192|ref|ZP\_00951714.1|
guanosine-3',5'-bis(diphosphate) ...   
70   2e-11

gi|83373197|ref|ZP\_00917976.1|
Threonyl-tRNA synthetase, class I...   
70   3e-11

gi|77388375|gb|ABA79560.1|
Threonyl-tRNA synthetase, class IIa [...   
70   3e-11

gi|46156858|ref|ZP\_00204773.1|
COG0317: Guanosine polyphosphate ...   
70   3e-11

gi|9105180|gb|AAF83162.1|
pentaphosphate guanosine-3'-pyrophosph...   
70   3e-11

gi|76875250|emb|CAI86471.1|
threonyl-tRNA synthetase [Pseudoalte...   
70   3e-11

gi|27361865|gb|AAO10771.1|
Threonyl-tRNA synthetase [Vibrio vuln...   
70   3e-11

gi|14247455|dbj|BAB57845.1|
threonyl-tRNA synthetase 1 [Staphylo...   
70   3e-11

gi|89209015|ref|ZP\_01187485.1|
Threonyl-tRNA synthetase, class I...   
70   3e-11

gi|81096574|ref|ZP\_00874914.1|
Threonyl-tRNA synthetase, class I...   
70   3e-11

gi|87305393|ref|ZP\_01087548.1|
GTP pyrophosphokinase (ATP:GTP 3'...   
70   3e-11

gi|33152811|ref|NP\_874164.1|
threonyl-tRNA synthetase [Haemophil...   
70   3e-11

gi|57166948|gb|AAW35727.1|
RelA/SpoT family protein [Campylobact...   
70   3e-11

gi|94493089|ref|ZP\_01300293.1|
hypothetical protein Rgryl\_010009...   
70   3e-11

gi|39935759|ref|NP\_948035.1|
GTP pyrophosphokinase [Rhodopseudom...   
70   3e-11

gi|6968705|emb|CAB73526.1|
putative guanosine-3',5'-bis(diphosph...   
70   3e-11

gi|29896005|gb|AAP09286.1|
Threonyl-tRNA synthetase [Bacillus ce...   
70   3e-11

gi|88810665|ref|ZP\_01125922.1|
threonyl-tRNA synthetase [Nitroco...   
70   3e-11

gi|78699271|ref|ZP\_00863767.1|
Threonyl-tRNA synthetase, class I...   
70   3e-11

gi|71900212|ref|ZP\_00682351.1|
RelA/SpoT protein [Xylella fastid...   
70   3e-11

gi|42783753|ref|NP\_981000.1|
threonyl-tRNA synthetase [Bacillus ...   
70   3e-11

gi|49333256|gb|AAT63902.1|
threonine--tRNA ligase (threonyl-tRNA...   
70   3e-11

gi|29898209|gb|AAP11483.1|
Threonyl-tRNA synthetase [Bacillus ce...   
70   3e-11

gi|91226476|ref|ZP\_01261225.1|
threonyl-tRNA synthetase [Vibrio ...    70   3e-11

gi|16414081|emb|CAC96825.1| threonyl-tRNA synthetase [Listeria i...    70  
3e-11

gi|47096724|ref|ZP\_00234309.1| threonyl-tRNA synthetase [Listeri...    70  
3e-11

gi|46907789|ref|YP\_014178.1| threonyl-tRNA synthetase [Listeria ...    70  
3e-11

gi|67668173|ref|ZP\_00465361.1| Threonyl-tRNA synthetase, class I...    70  
3e-11

gi|67546979|ref|ZP\_00424887.1| Threonyl-tRNA synthetase, class I...    70  
3e-11

gi|48853747|ref|ZP\_00307913.1| COG0441: Threonyl-tRNA synthetase...    70  
3e-11

gi|94499191|ref|ZP\_01305729.1| threonyl-tRNA synthetase [Oceanob...    70  
4e-11

gi|34482395|emb|CAE09396.1| GUANOSINE-PYROPHOSPHOHYDROLASE [Woli...    70  
4e-11

gi|88800311|ref|ZP\_01115877.1| threonyl-tRNA synthetase [Reineke...    70  
4e-11

gi|16410988|emb|CAC99637.1| threonyl-tRNA synthetase [Listeria m...    70  
4e-11

gi|34540737|ref|NP\_905216.1| threonyl-tRNA synthetase [Porphyrom...    69  
4e-11

gi|89100404|ref|ZP\_01173268.1| threonyl-tRNA synthetase [Bacillu...    69  
4e-11

gi|15619354|gb|AAL02834.1| threonyl-tRNA synthetase [EC:6.1.1.3]...    69  
4e-11

gi|68560899|ref|ZP\_00600207.1| Threonyl-tRNA synthetase, class I...    69  
4e-11

gi|57241087|ref|ZP\_00369034.1| penta-phosphate guanosine-3'-pyro...    69  
4e-11

gi|53732666|ref|ZP\_00154888.2| COG0441: Threonyl-tRNA synthetase...    69  
4e-11

gi|67922230|ref|ZP\_00515744.1| TGS [Crocosphaera watsonii WH 850...    69  
5e-11

gi|74016054|ref|ZP\_00686681.1| Threonyl-tRNA synthetase, class I...    69  
5e-11

gi|10581281|gb|AAG20043.1| threonyl-tRNA synthetase; ThrS [Halob...    69  
5e-11

gi|37680129|ref|NP\_934738.1| threonyl-tRNA synthetase [Vibrio vu...    69  
5e-11

gi|73921305|sp|Q9HP27|SYT\_HALSA Threonyl-tRNA synthetase (Threon...    69  
5e-11

gi|88859310|ref|ZP\_01133950.1| threonyl-tRNA synthetase [Pseudoa...    69  
5e-11

gi|56749447|sp|Q7MK65|SYT\_VIBVY Threonyl-tRNA synthetase (Threon...    69  
5e-11

gi|77918669|ref|YP\_356484.1| bifunctional enzyme (p)ppgpp synthe...    69  
5e-11

gi|21109746|gb|AAM38236.1| pentaphosphate guanosine-3'-pyrophosp...    69  
5e-11

gi|49532285|emb|CAG69997.1| putative bifunctional protein (SpoT)...    69  
5e-11

gi|78049066|ref|YP\_365241.1| guanosine-3',5'-bis(diphosphate) 3'...    69  
5e-11

gi|21114485|gb|AAM42517.1| pentaphosphate guanosine-3'-pyrophosp...    69  
5e-11

gi|28806268|dbj|BAC59543.1| threonyl-tRNA synthetase [Vibrio par...    69  
5e-11

gi|83369970|ref|ZP\_00914818.1| Threonyl-tRNA synthetase, class I...    69  
6e-11

gi|89891844|ref|ZP\_01203345.1| threonyl-tRNA synthetase [Flavoba...    69  
6e-11

gi|68548001|ref|ZP\_00587522.1| Threonyl-tRNA synthetase, class I...    69  
6e-11

gi|66572620|gb|AAY48030.1| pentaphosphate guanosine-3'-pyrophosp...    69  
6e-11

gi|58580772|ref|YP\_199788.1| pentaphosphate guanosine-3'-pyropho...    69  
6e-11

gi|84501632|ref|ZP\_00999804.1| Threonyl-tRNA synthetase, class I...    69  
6e-11

gi|22654147|sp|Q9RSP3|SYT\_DEIRA Threonyl-tRNA synthetase (Threon...    69   7e-11

gi|85842296|gb|EAQ59542.1|
RelA/SpoT family protein [Campylobact...   
69   7e-11

gi|92117778|ref|YP\_577507.1|
(p)ppGpp synthetase I (GTP pyrophos...   
69   7e-11

gi|69952318|ref|ZP\_00639831.1|
Threonyl-tRNA synthetase, class I...   
69   7e-11

gi|8928373|sp|Q9WZJ9|SYT\_THEMA
Threonyl-tRNA synthetase (Threoni...   
69   7e-11

gi|84391304|ref|ZP\_00991635.1|
threonyl-tRNA synthetase [Vibrio ...   
69   7e-11

gi|27380176|ref|NP\_771705.1|
GTP pyrophosphokinase [Bradyrhizobi...   
69   7e-11

gi|47565742|ref|ZP\_00236782.1|
threonyl-tRNA synthetase [Bacillu...   
69   7e-11

gi|2073475|emb|CAA72469.1|
threonyl-tRNA synthetase [Rickettsia ...   
69   7e-11

gi|93451702|gb|EAT02477.1|
GTP diphosphokinase [delta proteobact...   
69   8e-11

gi|21648164|gb|AAM73342.1|
threonyl-tRNA synthetase [Chlorobium ...   
69   8e-11

gi|75763967|ref|ZP\_00743592.1|
Threonyl-tRNA synthetase [Bacillu...   
69   8e-11

gi|86147528|ref|ZP\_01065839.1|
threonyl-tRNA synthetase [Vibrio ...   
69   8e-11

gi|75854097|ref|ZP\_00761814.1|
COG0441: Threonyl-tRNA synthetase...   
69   8e-11

gi|71062861|gb|AAZ21864.1|
GTP pyrophosphokinase [Candidatus Pel...   
69   8e-11

gi|82499365|ref|ZP\_00884811.1|
Threonyl-tRNA synthetase, class I...   
69   8e-11

gi|74420983|gb|ABA05182.1|
RelA/SpoT protein [Nitrobacter winogr...   
69   8e-11

gi|68055064|ref|ZP\_00539212.1|
Threonyl-tRNA synthetase, class I...   
68   9e-11

gi|85716288|ref|ZP\_01047262.1|
RelA/SpoT protein [Nitrobacter sp...   
68   9e-11

gi|51895835|gb|AAH81263.1|
MGC86352 protein [Xenopus laevis]          
68   9e-11

gi|78695364|ref|ZP\_00859876.1|
RelA/SpoT protein [Bradyrhizobium...   
68   9e-11

gi|51857385|dbj|BAD41543.1|
threonyl-tRNA synthetase [Symbiobact...   
68   9e-11

gi|93452665|gb|EAT03222.1|
TGS [delta proteobacterium MLMS-1] >g...   
68   1e-10

gi|73919789|sp|Q830D0|SYT\_ENTFA
Threonyl-tRNA synthetase (Threon...   
68   1e-10

gi|53752491|emb|CAH13923.1|
Threonyl tRNA synthetase [Legionella...  
 68   1e-10

gi|53755388|emb|CAH16884.1|
Threonyl tRNA synthetase [Legionella...   
68   1e-10

gi|89203670|ref|ZP\_01182280.1|
Threonyl-tRNA synthetase, class I...   
68   1e-10

gi|51259375|gb|AAH79154.1|
Threonyl-tRNA synthetase [Rattus norv...   
68   1e-10

gi|73919802|sp|Q5ZS05|SYT\_LEGPH
Threonyl-tRNA synthetase (Threon...   
68   1e-10

gi|55232719|gb|AAV48138.1|
threonyl-tRNA synthetase [Haloarcula ...   
68   1e-10

gi|71547348|ref|ZP\_00667994.1|
GTP diphosphokinase [Syntrophobac...   
68   1e-10

gi|45709710|gb|AAH67949.1|
Threonyl-tRNA synthetase-like 2 [Xeno...   
68   1e-10

gi|54644729|gb|EAL33469.1| GA18821-PA [Drosophila pseudoobscura]       68  
1e-10

gi|89359773|ref|ZP\_01197593.1| Threonyl-tRNA synthetase, class I...    68  
1e-10

gi|90417311|ref|ZP\_01225237.1| threonyl-tRNA synthetase [marine ...    68  
1e-10

gi|52630031|gb|AAU28772.1| threonyl tRNA synthase [Legionella pn...    68  
1e-10

gi|78366522|ref|ZP\_00836801.1| Threonyl-tRNA synthetase, class I...    68  
1e-10

gi|90579635|ref|ZP\_01235444.1| threonyl-tRNA synthetase [Vibrio ...    68  
1e-10

gi|73919847|sp|Q5NPH1|SYT\_ZYMMO Threonyl-tRNA synthetase (Threon...    68  
1e-10

gi|73919796|sp|Q5UX65|SYT\_HALMA Threonyl-tRNA synthetase (Threon...    68  
1e-10

gi|90576051|ref|ZP\_01232521.1| hypothetical protein CdifQ\_020005...    68  
1e-10

gi|1174427|sp|P43811|SPOT\_HAEIN Guanosine-3',5'-bis(diphosphate)...    68  
1e-10

gi|77966831|gb|ABB08211.1| Threonyl-tRNA synthetase, class IIa [...    68  
1e-10

gi|48870474|ref|ZP\_00323196.1| COG0441: Threonyl-tRNA synthetase...    68  
1e-10

gi|52632425|gb|AAH10578.2| Threonyl-tRNA synthetase [Homo sapiens]     68  
1e-10

gi|1464742|gb|AAB04939.1| threonyl-tRNA synthetase                     68   1e-10

gi|33875537|gb|AAH00517.2| Threonyl-tRNA synthetase [Homo sapien...    68  
1e-10

gi|62897167|dbj|BAD96524.1| threonyl-tRNA synthetase variant [Ho...    68  
1e-10

gi|56789234|gb|AAH88355.1| TARS protein [Homo sapiens]                 68   1e-10

gi|67926091|ref|ZP\_00519338.1| TGS [Crocosphaera watsonii WH 850...    68  
1e-10

gi|53732563|ref|ZP\_00349517.1| COG0317: Guanosine polyphosphate ...    68  
1e-10

gi|68058539|gb|AAX88792.1| guanosine-3',5'-bis 3'-pyrophosphohyd...    68  
1e-10

gi|30995477|ref|NP\_439885.2| guanosine-3'5'-bis(diphosphate) 3'-...    68  
1e-10

gi|46133641|ref|ZP\_00203220.1| COG0317: Guanosine polyphosphate ...    68  
1e-10

gi|55235240|gb|EAA14818.3| ENSANGP00000021424 [Anopheles gambiae...    67  
2e-10

gi|60492622|emb|CAH07394.1| putative threonyl-tRNA synthetase [B...    67  
2e-10

gi|89071308|ref|ZP\_01158467.1| threonyl-tRNA synthetase [Oceanic...    67  
2e-10

gi|75825688|ref|ZP\_00755124.1| COG0441: Threonyl-tRNA synthetase...    67  
2e-10

gi|9657684|gb|AAF96196.1| threonyl-tRNA synthetase [Vibrio chole...    67  
2e-10

gi|87122885|ref|ZP\_01078753.1| threonyl-tRNA synthetase [Marinom...    67  
2e-10

gi|76801848|ref|YP\_326856.1| threonine--tRNA ligase [Natronomona...    67  
2e-10

gi|73662434|ref|YP\_301215.1| GTP pyrophosphokinase [Staphylococc...    67  
2e-10

gi|84690138|gb|EAQ15979.1| guanosine polyphosphate pyrophosphohy...    67  
2e-10

gi|91215264|ref|ZP\_01252236.1| threonyl-tRNA synthetase [Psychro...    67  
2e-10

gi|49611876|emb|CAG75325.1| threonyl-tRNA synthetase [Erwinia ca...    67  
2e-10

gi|23003064|ref|ZP\_00046734.1| hypothetical protein Lgas\_0300116...    67  
2e-10

gi|77740398|ref|ZP\_00808886.1| RelA/SpoT protein [Rhodopseudomon...    67  
2e-10

gi|89073900|ref|ZP\_01160406.1| threonyl-tRNA synthetase [Photoba...    67  
2e-10

gi|76882963|gb|ABA57644.1| Threonyl-tRNA synthetase, class IIa [...    67  
2e-10

gi|73586669|gb|AAI03083.1| Hypothetical protein LOC510075 [Bos t...    67  
2e-10

gi|91762425|ref|ZP\_01264390.1| Threonyl-tRNA synthetase [Candida...    67  
2e-10

gi|68247746|gb|EAN29830.1| Guanosine-3',5'-bis(diphosphate) 3'-p...    67  
2e-10

gi|88806350|ref|ZP\_01121868.1| threonyl-tRNA synthetase [Robigin...    67  
2e-10

gi|84714305|ref|ZP\_01021770.1| Threonyl-tRNA synthetase, class I...    67  
3e-10

gi|84517881|ref|ZP\_01005230.1| threonyl-tRNA synthetase, class I...    67  
3e-10

gi|2636292|emb|CAB15783.1| threonyl-tRNA synthetase [Bacillus su...    67  
3e-10

gi|87198229|ref|YP\_495486.1| threonyl-tRNA synthetase [Novosphin...    67  
3e-10

gi|52004608|gb|AAU24550.1| threonyl-tRNA synthetase [Bacillus li...    67   3e-10

gi|73954351|ref|XP\_536509.2|
PREDICTED: similar to threonyl-tRNA...   
67   3e-10

gi|89205476|ref|ZP\_01184045.1|
Threonyl-tRNA synthetase, class I...   
67   3e-10

gi|81429010|ref|YP\_396010.1|
Threonyl-tRNA synthetase [Lactobaci...   
67   3e-10

gi|86137404|ref|ZP\_01055981.1|
threonyl-tRNA synthetase [Roseoba...   
66   3e-10

gi|68446967|dbj|BAE04551.1|
threonyl-tRNA synthetase 1 [Staphylo...   
66   3e-10

gi|75814362|ref|ZP\_00744961.1|
COG0441: Threonyl-tRNA synthetase...    66  
3e-10

gi|52428719|gb|AAU49312.1|
threonyl-tRNA synthetase [Burkholderi...   
66   3e-10

gi|55624526|ref|XP\_517835.1|
PREDICTED: threonyl-tRNA synthetase...   
66   3e-10

gi|86143057|ref|ZP\_01061479.1|
threonyl-tRNA synthetase [Flavoba...   
66   4e-10

gi|53680543|gb|AAU89472.1|
theronyl tRNA synthatase-like protein...   
66   4e-10

gi|91784428|ref|YP\_559634.1|
Threonyl-tRNA synthetase, class IIa...   
66   4e-10

gi|65319683|ref|ZP\_00392642.1|
COG0441: Threonyl-tRNA synthetase...   
66   4e-10

gi|71062257|gb|AAZ21260.1|
Threonyl-tRNA synthetase [Candidatus ...   
66   4e-10

gi|83751293|ref|ZP\_00947707.1|
COG0441: Threonyl-tRNA synthetase...   
66   4e-10

gi|39936469|ref|NP\_948745.1|
threonyl-tRNA synthetase [Rhodopseu...   
66   4e-10

gi|83309439|ref|YP\_419703.1|
Threonyl-tRNA synthetase [Magnetosp...   
66   4e-10

gi|83719712|ref|YP\_443111.1|
threonyl-tRNA synthetase [Burkholde...   
66   5e-10

gi|46401290|emb|CAF24739.1|
probable threonine-tRNA ligase [Para...   
66   5e-10

gi|68550184|ref|ZP\_00589638.1|
Threonyl-tRNA synthetase, class I...   
66   5e-10

gi|74218920|dbj|BAE37840.1|
unnamed protein product [Mus musculus]    
66   5e-10

gi|21313644|ref|NP\_082207.1|
threonyl-tRNA synthetase-like 1 [Mu...   
66   5e-10

gi|74216488|dbj|BAE25159.1|
unnamed protein product [Mus musculus]    
66   5e-10

gi|14290460|gb|AAH08995.1|
Tarsl1 protein [Mus musculus]              
66   5e-10

gi|86154539|ref|ZP\_01072707.1|
GTP pyrophosphokinase (ATP:GTP 3'...   
66   5e-10

gi|22777839|dbj|BAC14110.1|
threonine-tRNA ligase [Oceanobacillu...   
66   5e-10

gi|28199783|ref|NP\_780097.1|
threonyl-tRNA synthetase [Xylella f...   
66   5e-10

gi|71275167|ref|ZP\_00651454.1|
Threonyl-tRNA synthetase, class I...   
66   5e-10

gi|77997086|gb|ABB15985.1|
threonyl-tRNA synthetase [Carboxydoth...   
66   5e-10

gi|17740186|gb|AAL42745.1|
threonyl-tRNA synthetase [Agrobacteri...   
66   5e-10

gi|78165915|gb|ABB23013.1|
Threonyl-tRNA synthetase, class IIa [...   
66   5e-10

gi|86357812|ref|YP\_469704.1|
threonyl-tRNA synthetase protein [R...   
66   5e-10

gi|71895375|ref|NP\_001026618.1|
threonyl-tRNA synthetase [Gallus...   
66   5e-10

gi|86134203|ref|ZP\_01052785.1|
threonyl-tRNA synthetase [Tenacib...   
65   6e-10

gi|25013040|gb|AAN71609.1|
RH56418p [Drosophila melanogaster]        
 65   6e-10

gi|24583839|ref|NP\_723725.1|
Threonyl-tRNA synthetase CG5353-PA,...   
65   6e-10

gi|24583842|ref|NP\_723726.1|
Threonyl-tRNA synthetase CG5353-PC,...   
65   6e-10

gi|21428938|gb|AAM50188.1|
GH20022p [Drosophila melanogaster]         
65   6e-10

gi|88941862|ref|ZP\_01147256.1|
threonyl-tRNA synthetase [Acidiph...    65   6e-10

gi|71899804|ref|ZP\_00681954.1| TGS [Xylella fastidiosa Ann-1]
>g...    65   6e-10

gi|50752847|ref|XP\_413774.1|
PREDICTED: similar to FLJ25005 prot...   
65   6e-10

gi|26326983|dbj|BAC27235.1|
unnamed protein product [Mus musculus]    
65   6e-10

gi|12845562|dbj|BAB26799.1|
unnamed protein product [Mus musculus]    
65   6e-10

gi|74142262|dbj|BAE31895.1|
unnamed protein product [Mus musculus]    
65   6e-10

gi|55741823|ref|NP\_001006977.1|
threonyl-tRNA synthetase [Rattus...    65   6e-10

gi|33286920|gb|AAH55371.1| Hypothetical protein D15Wsu59e [Mus m...    65  
6e-10

gi|27229277|ref|NP\_149065.2| threonyl-tRNA synthetase [Mus muscu...    65   6e-10

gi|73535478|pdb|1WWT|A
Chain A, Solution Structure Of The Tgs Do...   
65   6e-10

gi|88706032|ref|ZP\_01103740.1|
threonyl-tRNA synthetase [gamma p...   
65   7e-10

gi|78712481|gb|ABB49658.1|
threonyl-tRNA synthetase [Prochloroco...   
65   8e-10

gi|103486185|ref|YP\_615746.1|
threonyl-tRNA synthetase [Sphingop...   
65   8e-10

gi|90407204|ref|ZP\_01215391.1|
guanosine-3',5'-bis(diphosphate) ...   
65   8e-10

gi|86749289|ref|YP\_485785.1|
threonyl-tRNA synthetase [Rhodopseu...   
65   8e-10

gi|42519522|ref|NP\_965452.1|
threonyl-tRNA synthetase [Lactobaci...   
65   8e-10

gi|56604420|emb|CAG45450.1|
Threonyl-tRNA synthetase [Francisell...   
65   9e-10

gi|89256700|ref|YP\_514062.1|
Threonyl-tRNA synthetase [Francisel...   
65   9e-10

gi|84516459|ref|ZP\_01003818.1|
threonyl-tRNA synthetase [Loktane...   
65   9e-10

gi|29337725|gb|AAO75529.1|
threonyl-tRNA synthetase [Bacteroides...   
65   9e-10

gi|68551819|ref|ZP\_00591213.1|
Threonyl-tRNA synthetase, class I...   
65   1e-09

gi|91776355|ref|YP\_546111.1|
threonyl-tRNA synthetase [Methyloba...   
65   1e-09

gi|17982868|gb|AAL52096.1|
THREONYL-TRNA SYNTHETASE [Brucella me...   
65   1e-09

gi|23347895|gb|AAN29991.1|
threonyl-tRNA synthetase [Brucella su...   
65   1e-09

gi|68445859|dbj|BAE03443.1|
threonyl-tRNA synthetase 2 (Threonin...   
64   1e-09

gi|71492060|gb|EAO24371.1|
Threonyl-tRNA synthetase, class IIa [...   
64   1e-09

gi|9105624|gb|AAF83546.1|
threonyl-tRNA synthetase [Xylella fast...   
64   1e-09

gi|15074358|emb|CAC46004.1|
PROBABLE THREONYL-TRNA SYNTHETASE PR...   
64   1e-09

gi|76664608|ref|XP\_603703.2|
PREDICTED: similar to threonyl-tRNA...   
64   1e-09

gi|76664612|ref|XP\_877531.1|
PREDICTED: similar to threonyl-tRNA...   
64   1e-09

gi|76664614|ref|XP\_869309.1|
PREDICTED: similar to threonyl-tRNA...    64  
1e-09

gi|71549915|ref|ZP\_00670087.1|
Threonyl-tRNA synthetase, class I...   
64   1e-09

gi|14021790|dbj|BAB48401.1|
threonyl-tRNA synthetase [Mesorhizob...   
64   1e-09

gi|83942907|ref|ZP\_00955367.1|
threonyl-tRNA synthetase [Sulfito...   
64   1e-09

gi|47226601|emb|CAG08617.1|
unnamed protein product [Tetraodon n...   
64   1e-09

gi|91793061|ref|YP\_562712.1|
threonyl-tRNA synthetase [Shewanell...   
64   1e-09

gi|73919816|sp|Q7VBM8|SYT\_PROMA
Threonyl-tRNA synthetase (Threon...   
64   2e-09

gi|84687895|ref|ZP\_01015762.1|
threonyl-tRNA synthetase [Rhodoba...   
64   2e-09

gi|28271063|emb|CAD63968.1|
threonine--tRNA ligase 1 [Lactobacil...   
64   2e-09

gi|49612264|emb|CAG75714.1|
molybdopterin converting factor subu...   
64   2e-09

gi|85716379|ref|ZP\_01047351.1|
threonyl-tRNA synthetase [Nitroba...   
64   2e-09

gi|99081562|ref|YP\_613716.1|
threonyl-tRNA synthetase [Silicibac...   
64   2e-09

gi|91977727|ref|YP\_570386.1|
threonyl-tRNA synthetase [Rhodopseu...   
64   2e-09

gi|74317025|ref|YP\_314765.1|
threonyl-tRNA synthetase, class IIa...   
64   2e-09

gi|83951679|ref|ZP\_00960411.1|
threonyl-tRNA synthetase [Roseova...   
64   2e-09

gi|27380648|ref|NP\_772177.1|
threonyl-tRNA synthetase [Bradyrhiz...   
64   2e-09

gi|104774352|ref|YP\_619332.1|
Threonyl-tRNA synthetase [Lactobac...    64   2e-09

gi|3875006|emb|CAA93762.1| Hypothetical protein C47D12.6a [Caeno...    64  
2e-09

gi|30145813|emb|CAD89723.1| Hypothetical protein C47D12.6b [Caen...    64  
2e-09

gi|62515087|ref|ZP\_00386561.1| COG0441: Threonyl-tRNA synthetase...    64  
2e-09

gi|71898248|ref|ZP\_00680422.1| Threonyl-tRNA synthetase, class I...    64  
3e-09

gi|86158405|ref|YP\_465190.1| threonyl-tRNA synthetase [Anaeromyx...    64  
3e-09

gi|78171870|gb|ABB28966.1| Threonyl-tRNA synthetase, class IIa [...    64  
3e-09

gi|30248961|ref|NP\_841031.1| thrS; threonyl-tRNA synthetase (thr...    64  
3e-09

gi|88714286|ref|ZP\_01108360.1| threonyl-tRNA synthetase [Flavoba...    64  
3e-09

gi|50943671|ref|XP\_481363.1| putative threonyl-tRNA synthetase [...    64  
3e-09

gi|62196119|gb|AAX74419.1| ThrS, threonyl-tRNA synthetase [Bruce...    63  
3e-09

gi|90424612|ref|YP\_532982.1| threonyl-tRNA synthetase [Rhodopseu...    63  
3e-09

gi|72001723|gb|AAZ57525.1| threonyl-tRNA synthetase, class IIa [...    63   3e-09

gi|33634613|emb|CAE20599.1| Threonyl-tRNA synthatase [Prochloroc...    63  
3e-09

gi|90589478|ref|ZP\_01245127.1| Threonyl-tRNA synthetase, class I...    63  
3e-09

gi|78364294|gb|ABB42259.1| threonyl-tRNA synthetase [Thiomicrosp...    63  
4e-09

gi|82747601|ref|ZP\_00910096.1| Threonyl-tRNA synthetase, class I...    63  
4e-09

gi|56677908|gb|AAV94574.1| threonyl-tRNA synthetase [Silicibacte...    63  
4e-09

gi|52001909|gb|AAU21851.1| threonyl-tRNA synthetase [Bacillus li...    63  
4e-09

gi|12802990|gb|AAH00541.1| Threonyl-tRNA synthetase-like 1 [Homo...    63  
4e-09

gi|55958997|emb|CAI15489.1| threonyl-tRNA synthetase-like 1 [Hom...    63  
4e-09

gi|62897187|dbj|BAD96534.1| threonyl-tRNA synthetase-like 1 vari...    63  
4e-09

gi|55958995|emb|CAI15487.1| threonyl-tRNA synthetase-like 1 [Hom...    63  
4e-09

gi|39644807|gb|AAH07824.2| TARSL1 protein [Homo sapiens]               63   4e-09

gi|38570111|ref|NP\_689547.2| threonyl-tRNA synthetase-like 2 [Ho...    63  
4e-09

gi|55642773|ref|XP\_510628.1| PREDICTED: hypothetical protein XP\_...    63  
4e-09

gi|71147959|gb|AAZ28432.1| threonyl-tRNA synthetase [Colwellia p...    63  
5e-09

gi|67941803|ref|ZP\_00533816.1| TGS [Chlorobium phaeobacteroides ...    63  
5e-09

gi|67935508|ref|ZP\_00528528.1| Threonyl-tRNA synthetase, class I...    62  
5e-09

gi|85375301|ref|YP\_459363.1| threonyl-tRNA synthetase [Erythroba...    62  
5e-09

gi|74421095|gb|ABA05294.1| threonyl-tRNA synthetase, class IIa [...    62  
5e-09

gi|83954081|ref|ZP\_00962801.1| threonyl-tRNA synthetase [Sulfito...    62  
5e-09

gi|88803834|ref|ZP\_01119357.1| threonyl-tRNA synthetase [Polarib...    62  
5e-09

gi|91070150|gb|ABE11072.1| threonyl-tRNA synthetase [uncultured ...    62  
5e-09

gi|91201672|emb|CAJ74732.1| strongly similar to threonyl-tRNA sy...    62  
5e-09

gi|33639886|emb|CAE19056.1| Threonyl-tRNA synthetase [Prochloroc...    62  
6e-09

gi|69938663|ref|ZP\_00633088.1| Threonyl-tRNA synthetase, class I...    62  
6e-09

gi|50555740|ref|XP\_505278.1| hypothetical protein [Yarrowia lipo...    62  
6e-09

gi|82701621|ref|YP\_411187.1| threonyl-tRNA synthetase [Nitrososp...    62  
6e-09

gi|16764168|ref|NP\_459783.1| molybdopterin biosynthetic protein ...    62  
6e-09

gi|39597482|emb|CAE59712.1| Hypothetical protein CBG03144 [Caeno...    62  
7e-09

gi|90021224|ref|YP\_527051.1| Peptidase S16, ATP-dependent protea...    62  
7e-09

gi|62179373|ref|YP\_215790.1| molybdopterin biosynthesis protein ...    62  
7e-09

gi|106886170|ref|ZP\_01353516.1| threonyl-tRNA synthetase [Clostr...    62  
8e-09

gi|29829014|ref|NP\_823648.1| ppGpp synthetase/hydrolase [Strepto...    62  
8e-09

gi|21224139|ref|NP\_629918.1| kinase/phosphohydrolase [Streptomyc...    62  
9e-09

gi|18145987|dbj|BAB82028.1| threonine-tRNA ligase [Clostridium p...    62  
9e-09

gi|77955231|ref|ZP\_00819605.1| Threonyl-tRNA synthetase, class I...    62  
9e-09

gi|58001703|gb|AAW60597.1| Threonyl-tRNA synthetase [Gluconobact...    62  
1e-08

gi|77738972|ref|ZP\_00807465.1| Threonyl-tRNA synthetase, class I...    62  
1e-08

gi|62148593|emb|CAH64365.1| threonyl-tRNA synthetase [Chlamydoph...    61  
1e-08

gi|94491562|ref|ZP\_01298773.1| hypothetical protein CburD\_010013...    61  
1e-08

gi|72394688|gb|AAZ68965.1| Threonyl-tRNA synthetase, class IIa [...    61  
1e-08

gi|68190001|gb|EAN04663.1| Threonyl-tRNA synthetase, class IIa [...    61  
1e-08

gi|67919799|ref|ZP\_00513361.1| Threonyl-tRNA synthetase, class I...    61  
1e-08

gi|75823609|ref|ZP\_00753099.1| COG1977: Molybdopterin converting...    61  
1e-08

gi|32262380|gb|AAP77428.1| conserved hypothetical protein [Helic...    61   1e-08

gi|73951029|ref|XP\_857141.1|
PREDICTED: similar to threonyl-tRNA...   
61   1e-08

gi|73951031|ref|XP\_857184.1|
PREDICTED: similar to threonyl-tRNA...   
61   1e-08

gi|73951033|ref|XP\_857231.1|
PREDICTED: similar to threonyl-tRNA...   
61   1e-08

gi|73951035|ref|XP\_857273.1|
PREDICTED: similar to threonyl-tRNA...   
61   1e-08

gi|73951037|ref|XP\_536172.2|
PREDICTED: similar to threonyl-tRNA...   
61   1e-08

gi|16759729|ref|NP\_455346.1|
molybdopterin converting factor, su...   
61   1e-08

gi|92117217|ref|YP\_576946.1|
threonyl-tRNA synthetase [Nitrobact...   
61   1e-08

gi|17428593|emb|CAD15279.1|
probable threonyl-trna synthetase (t...   
61   1e-08

gi|9655490|gb|AAF94186.1|
molybdenum cofactor biosynthesis prote...  
 61   1e-08

gi|49239675|emb|CAF26046.1|
Threonyl-tRNA synthetase [Bartonella...   
61   2e-08

gi|30690426|ref|NP\_198035.2|
ATP binding / tRNA ligase/ threonin...   
61   2e-08

gi|2191162|gb|AAB61048.1|
Similar to threonyl-tRNA synthetase; c...   
61   2e-08

gi|3617770|emb|CAA74705.1|
threonyl-tRNA synthetase [Arabidopsis...   
61   2e-08

gi|55588130|ref|XP\_513775.1|
PREDICTED: hypothetical protein XP\_...   
61   2e-08

gi|89897864|ref|YP\_514974.1|
threoninyl tRNA synthetase [Chlamyd...   
61   2e-08

gi|56311941|emb|CAI06586.1|
threonyl-tRNA synthetase [Azoarcus s...   
61   2e-08

gi|29835062|gb|AAP05696.1|
threonyl-tRNA synthetase [Chlamydophi...   
60   2e-08

gi|66513879|ref|XP\_395662.2|
PREDICTED: similar to GA18821-PA [A...   
60   2e-08

gi|75819545|ref|ZP\_00749620.1|
COG1977: Molybdopterin converting...   
60   2e-08

gi|83594816|ref|YP\_428568.1|
Threonyl-tRNA synthetase, class IIa...   
60   2e-08

gi|58263294|ref|XP\_569057.1|
threonine-tRNA ligase [Cryptococcus...   
60   3e-08

gi|40062682|gb|AAR37595.1|
threonyl-tRNA synthetase [uncultured ...   
60   3e-08

gi|95926701|ref|ZP\_01309478.1|
hypothetical protein CburR\_010015...   
60   3e-08

gi|15081626|gb|AAK82468.1|
AT5g26830/F2P16\_90 [Arabidopsis thali...   
60   3e-08

gi|89053589|ref|YP\_509040.1|
threonyl-tRNA synthetase [Jannaschi...   
60   3e-08

gi|29541892|gb|AAO90830.1|
threonyl-tRNA synthetase [Coxiella bu...   
60   4e-08

gi|73981321|ref|XP\_860741.1|
PREDICTED: similar to threonyl-tRNA...   
60   4e-08

gi|73981323|ref|XP\_860781.1|
PREDICTED: similar to threonyl-tRNA...   
60   4e-08

gi|73981325|ref|XP\_850160.1|
PREDICTED: similar to threonyl-tRNA...   
60   4e-08

gi|73981327|ref|XP\_860843.1|
PREDICTED: similar to threonyl-tRNA...   
60   4e-08

gi|13421637|gb|AAK22451.1|
threonyl-tRNA synthetase [Caulobacter...   
60   4e-08

gi|71655606|ref|XP\_816364.1|
threonyl-tRNA synthetase [Trypanoso...   
60   4e-08

gi|71418959|ref|XP\_811024.1|
threonyl-tRNA synthetase [Trypanoso...   
60   4e-08

gi|39575326|emb|CAE79494.1|
thrS [Bdellovibrio bacteriovorus HD1...   
59   4e-08

gi|56910705|dbj|BAD65232.1|
threonyl-tRNA synthetase [Bacillus c...   
59   4e-08

gi|49238343|emb|CAF27567.1|
Threonyl-tRNA synthetase [Bartonella...   
59   5e-08

gi|91769119|ref|ZP\_01270953.1|
GTP diphosphokinase [Psychrobacte...   
59   5e-08

gi|15830116|ref|NP\_308889.1|
molybdopterin biosynthesis protein ...   
59   5e-08

gi|83748297|ref|ZP\_00945322.1|
Threonyl-tRNA synthetase [Ralston...   
59   5e-08

gi|71021529|ref|XP\_760995.1|
hypothetical protein UM04848.1 [Ust...    59  
5e-08

gi|56383279|gb|AAN42369.2|
molybdopterin biosynthesis protein D ...   
59   5e-08

gi|50876794|emb|CAG36634.1|
probable GTP pyrophosphokinase [Desu...   
59   5e-08

gi|89094703|ref|ZP\_01167639.1|
threonyl-tRNA synthetase [Oceanos...   
59   6e-08

gi|82534581|ref|ZP\_00893613.1|
hypothetical protein Bpse110\_0200...   
59   6e-08

gi|76156813|gb|AAX27938.2|
SJCHGC03429 protein [Schistosoma japo...   
59   6e-08

gi|82776146|ref|YP\_402493.1|
molybdopterin biosynthesis [Shigell...   
59   7e-08

gi|76612754|ref|XP\_884311.1|
PREDICTED: similar to threonyl-tRNA...   
58   7e-08

gi|76612756|ref|XP\_884338.1|
PREDICTED: similar to threonyl-tRNA...   
58   7e-08

gi|76612758|ref|XP\_589133.2|
PREDICTED: similar to threonyl-tRNA...   
58   7e-08

gi|76612760|ref|XP\_884398.1|
PREDICTED: similar to threonyl-tRNA...   
58   7e-08

gi|76612762|ref|XP\_872033.1|
PREDICTED: similar to threonyl-tRNA...   
58   7e-08

gi|76612764|ref|XP\_884462.1|
PREDICTED: similar to threonyl-tRNA...   
58   7e-08

gi|78497458|gb|ABB43998.1|
metal dependent phosphohydrolase [Thi...   
58   9e-08

gi|26107155|gb|AAN79339.1|
Molybdopterin converting factor subun...   
58   9e-08

gi|6136306|gb|AAF04327.1|
RelA/SpoT homolog [Bradyrhizobium japo...   
58   1e-07

gi|16552114|dbj|BAB71241.1| unnamed protein product [Homo sapiens]     58  
1e-07

gi|76167848|gb|AAX50856.1| threonyl-tRNA synthetase [Chlamydia t...    58  
1e-07

gi|7437527|pir||G71497 threonine-tRNA ligase (EC 6.1.1.3) - Chla...    58  
1e-07

gi|82524022|emb|CAI78700.1| Threonyl-tRNA synthetase [uncultured...    58  
1e-07

gi|21363114|gb|AAM00430.2| putative ppGpp synthetase/hydrolase [...    58  
1e-07

gi|27362554|gb|AAO11408.1| Molybdenum cofactor biosynthesis prot...    58  
1e-07

gi|53734186|gb|AAH83525.1| Zgc:92586 [Danio rerio]
>gi|54400362|...    58   1e-07

gi|1787002|gb|AAC73871.1| molybdopterin synthase, small subunit ...    58  
1e-07

gi|85057943|ref|YP\_456859.1| threonyl-tRNA synthetase [Aster yel...    57  
2e-07

gi|58579605|ref|YP\_197817.1| Threonyl-tRNA synthetase [Ehrlichia...    57  
2e-07

gi|76261574|ref|ZP\_00769179.1| Molybdopterin converting factor, ...    57  
2e-07

gi|88657649|ref|YP\_506841.1| threonyl-tRNA synthetase [Ehrlichia...    57  
2e-07

gi|58617659|ref|YP\_196858.1| Threonyl-tRNA synthetase [Ehrlichia...    57  
2e-07

gi|54035500|gb|AAH83878.1| Threonyl-tRNA synthetase-like 2 [Ratt...    57  
2e-07

gi|70905726|gb|AAZ14365.1| threonyl-tRNA synthetase, putative [L...    57  
2e-07

gi|24657723|gb|AAH39225.1| Threonyl-tRNA synthetase-like 2 [Mus ...    57  
2e-07

gi|26334063|dbj|BAC30749.1| unnamed protein product [Mus musculus]     57  
2e-07

gi|90411674|ref|ZP\_01219684.1| hypothetical protein P3TCK\_16464 ...    57  
2e-07

gi|75189826|ref|ZP\_00703093.1| COG1977: Molybdopterin converting...    57  
2e-07

gi|18893936|gb|AAL81887.1| GTP-binding protein, gtp1/obg family ...    57  
2e-07

gi|67916523|ref|ZP\_00510230.1| TGS domain:Phosphoribulokinase/ur...    57  
2e-07

gi|7190897|gb|AAF39666.1| threonyl-tRNA synthetase [Chlamydia mu...    57  
2e-07

gi|85705178|ref|ZP\_01036278.1| threonyl-tRNA synthetase [Roseova...    57  
2e-07

gi|50952758|gb|AAT90291.1| putative threonyl-tRNA synthetase [un...    57  
2e-07

gi|33152460|ref|NP\_873813.1| molybdopterin converting factor sub...    57  
2e-07

gi|42011|emb|CAA49864.1| moaD [Escherichia coli]                       57   2e-07

gi|75236193|ref|ZP\_00720310.1|
COG1977: Molybdopterin converting...   
57   2e-07

gi|50363549|gb|AAT75534.1|
threonyl-tRNA synthetase [Mesoplasma ...   
57   3e-07

gi|62512520|ref|ZP\_00384164.1|
COG0441: Threonyl-tRNA synthetase...   
57   3e-07

gi|33575568|emb|CAE32646.1|
threonyl-tRNA synthetase [Bordetella...   
57   3e-07

gi|19887790|gb|AAM02412.1|
Predicted GTPase of the OBG/HflX supe...   
57   3e-07

gi|68491354|ref|XP\_710514.1| threonyl-tRNA synthetase [Candida a...    57  
3e-07

gi|90579138|ref|ZP\_01234948.1| hypothetical protein VAS14\_05513 ...    56  
3e-07

gi|62176044|gb|AAX70165.1| threonyl-tRNA synthetase, putative [T...    56   3e-07

gi|30749759|pdb|1NVI|D
Chain D, Orthorhombic Crystal Form Of Mol...   
56   4e-07

gi|57159545|dbj|BAD85475.1|
predicted GTPase, GTP1/OBG family, c...   
56   4e-07

gi|75210621|ref|ZP\_00710769.1|
COG1977: Molybdopterin converting...   
56   4e-07

gi|15618715|ref|NP\_225001.1|
threonyl-tRNA synthetase [Chlamydop...   
56   4e-07

gi|89073364|ref|ZP\_01159888.1|
hypothetical protein SKA34\_20482 ...   
56   4e-07

gi|3258101|dbj|BAA30784.1|
387aa long hypothetical developmental...   
56   5e-07

gi|32033743|ref|ZP\_00134038.1|
COG1977: Molybdopterin converting...    56  
5e-07

gi|45435691|gb|AAS61249.1|
molybdopterin [mpt] converting factor...   
56   5e-07

gi|5105750|dbj|BAA81062.1|
368aa long hypothetical developmental...   
56   5e-07

gi|55771441|dbj|BAD69882.1|
molybdenum cofactor biosynthesis pro...   
56   5e-07

gi|94415786|ref|ZP\_01295621.1|
hypothetical protein PaerP\_010023...   
56   6e-07

gi|9950101|gb|AAG07304.1|
molybdopterin converting factor, small...   
55   7e-07

gi|32039081|ref|ZP\_00137353.1|
COG1977: Molybdopterin converting...   
55   7e-07

gi|49082512|gb|AAT50656.1|
PA3917 [synthetic construct]               
55   7e-07

gi|52696120|pdb|1VJK|A
Chain A, Putative Molybdopterin Convertin...   
55   7e-07

gi|15621520|dbj|BAB65515.1|
359aa long hypothetical GTP-binding ...   
55   7e-07

gi|93006829|ref|YP\_581266.1| metal dependent phosphohydrolase [P...    55  
7e-07

gi|5457931|emb|CAB49421.1| GTP-binding protein [Pyrococcus abyss...    55  
7e-07

gi|68545076|ref|ZP\_00584627.1| Molybdopterin converting factor, ...    55  
9e-07

gi|94971761|ref|YP\_593809.1| threonyl-tRNA synthetase [Acidobact...    55  
9e-07

gi|50310653|ref|XP\_455348.1| unnamed protein product [Kluyveromy...    55  
1e-06

gi|88793099|ref|ZP\_01108816.1| molybdopterin converting factor, ...    55  
1e-06

gi|18892532|gb|AAL80667.1| molybdopterin converting factor, subu...    55  
1e-06

gi|66806197|ref|XP\_636821.1| threonine-tRNA ligase [Dictyosteliu...    55  
1e-06

gi|56911982|dbj|BAD66509.1| threonyl-tRNA synthetase [Bacillus c...    55  
1e-06

gi|55167981|gb|AAV43849.1| putative GTP binding protein [Oryza s...    54  
1e-06

gi|77957352|ref|ZP\_00821410.1| COG1977: Molybdopterin converting...    54  
1e-06

gi|46200249|ref|YP\_005916.1| molybdopterin (MPT) converting fact...    54  
1e-06

gi|90417791|ref|ZP\_01225703.1| threonyl-tRNA synthetase [Auranti...    54  
1e-06

gi|12720897|gb|AAK02707.1| MoaD [Pasteurella multocida subsp. mu...    54  
1e-06

gi|71039265|gb|AAZ19573.1| guanosine-3',5'-bis(diphosphate) 3'-p...    54  
1e-06

gi|15645394|ref|NP\_207568.1| penta-phosphate guanosine-3'-pyroph...    54  
1e-06

gi|71908290|ref|YP\_285877.1| Threonyl-tRNA synthetase, class IIa...    54  
1e-06

gi|4155268|gb|AAD06287.1| GUANOSINE-3',5'-BIS(DIPHOSPHATE) 3'-PY...    54  
1e-06

gi|77960819|ref|ZP\_00824674.1| COG1977: Molybdopterin converting...    54  
2e-06

gi|34496803|ref|NP\_901018.1| threonyl-tRNA synthetase [Chromobac...    54  
2e-06

gi|2104451|emb|CAB08788.1| ths1 [Schizosaccharomyces pombe]
>gi|...    54   2e-06

gi|46108732|ref|XP\_381424.1| hypothetical protein FG01248.1 [Gib...    54  
2e-06

gi|12620119|gb|AAG60573.1| putative molybdenum cofactor biosynth...    54  
2e-06

gi|18159566|gb|AAL62982.1| molybdenum cofactor biosynthesis prot...    53  
2e-06

gi|2345148|gb|AAB67829.1| developmentally regulated GTP binding ...    53  
3e-06

gi|90407696|ref|ZP\_01215875.1| molybdopterin biosynthesis protei...    53  
3e-06

gi|75431111|ref|ZP\_00732938.1| molybdopterin (MPT) converting fa...    53  
3e-06

gi|23467044|ref|ZP\_00122629.1| COG1977: Molybdopterin converting...    53  
3e-06

gi|84319278|ref|ZP\_00967677.1| COG0315: Molybdenum cofactor bios...    53  
3e-06

gi|38503291|sp|Q58722|Y1326\_METJA Hypothetical GTP-binding prote...    53  
3e-06

gi|94984437|ref|YP\_603801.1| molybdopterin converting factor, su...    53  
3e-06

gi|56908910|dbj|BAD63437.1| molybdopterin converting factor subu...    53  
4e-06

gi|77976770|ref|ZP\_00832242.1| COG1977: Molybdopterin converting...    53  
4e-06

gi|34482542|emb|CAE09542.1| hypothetical protein [Wolinella succ...    53  
4e-06

gi|77973923|ref|ZP\_00829467.1| COG1977: Molybdopterin converting...    53  
4e-06

gi|87309081|ref|ZP\_01091218.1| threonyl-tRNA synthetase [Blastop...    53  
4e-06

gi|98659923|dbj|GAA02748.1| unnamed protein product [Pelotomacul...    53  
4e-06

gi|36784880|emb|CAE13794.1| molybdopterin [mpt] converting facto...    53  
4e-06

gi|51976504|gb|AAU18054.1| GTP pyrophosphokinase [Bacillus cereu...    53  
5e-06

gi|78219002|gb|ABB38351.1| thiamine biosynthesis protein ThiS [D...    53  
5e-06

gi|68562526|ref|ZP\_00601781.1| Molybdopterin converting factor, ...    53  
5e-06

gi|44981367|gb|AAS51189.1| ACL039Wp [Ashbya gossypii ATCC 10895]...    53  
5e-06

gi|50876317|emb|CAG36157.1| probable threonyl-tRNA synthetase [D...    52  
5e-06

gi|47570054|ref|ZP\_00240714.1| guanosine-3',5'-bis(diphosphate) ...    52  
5e-06

gi|47220916|emb|CAG03123.1| unnamed protein product [Tetraodon n...    52  
5e-06

gi|46912747|emb|CAG19537.1| hypothetical protein [Photobacterium...    52  
5e-06

gi|2950451|emb|CAA17801.1| gtp1 [Schizosaccharomyces pombe] >gi|...    52  
5e-06

gi|77814639|ref|ZP\_00813897.1| Molybdopterin converting factor, ...    52   6e-06

gi|67599391|ref|XP\_666284.1| GTP-binding-like protein [Cryptospo...    52  
6e-06

gi|86609832|ref|YP\_478594.1|
thiamine biosynthesis protein ThiS ...    52  
6e-06

gi|39942942|ref|XP\_361008.1|
hypothetical protein MG03551.4 [Mag...   
52   6e-06

gi|52307130|gb|AAU37630.1|
MoaD protein [Mannheimia succinicipro...   
52   6e-06

gi|33356787|ref|NP\_127095.2|
molybdopterin converting factor, su...   
52   6e-06

gi|66514467|ref|XP\_394753.2|
PREDICTED: similar to ENSANGP000000...   
52   7e-06

gi|66514472|ref|XP\_623152.1|
PREDICTED: similar to ENSANGP000000...   
52   7e-06

gi|34763461|ref|ZP\_00144407.1|
Threonyl-tRNA synthetase [Fusobac...   
52   7e-06

gi|57160377|dbj|BAD86307.1|
molybdopterin converting factor, sub...   
52   7e-06

gi|5458838|emb|CAB50325.1|
moaD molybdopterin synthase, small su...   
52   7e-06

gi|50939391|ref|XP\_479223.1|
putative GTP-binding protein DRG [O...   
52   7e-06

gi|86147951|ref|ZP\_01066255.1|
Molybdenum cofactor biosynthesis ...   
52   8e-06

gi|84393380|ref|ZP\_00992139.1|
Molybdenum cofactor biosynthesis ...   
52   9e-06

gi|27803058|emb|CAD60761.1|
unnamed protein product [Podospora a...   
52   9e-06

gi|33359306|ref|NP\_877770.1|
putative molybdopterin converting f...   
52   9e-06

gi|42560810|ref|NP\_975261.1|
threonine-tRNA ligase [Mycoplasma m...   
52   9e-06

gi|50414355|ref|XP\_457398.1|
hypothetical protein DEHA0B10472g [...   
52   9e-06

gi|67156787|ref|ZP\_00418284.1|
ThiamineS [Azotobacter vinelandii...   
52   9e-06

gi|91775615|ref|YP\_545371.1|
molybdopterin converting factor, su...   
52   1e-05

gi|9946232|gb|AAG03769.1|
conserved hypothetical protein [Pseudo...   
52   1e-05

gi|40068632|gb|AAR38967.1|
NEQ112 [Nanoarchaeum equitans Kin4-M]...   
52   1e-05

gi|68541239|ref|ZP\_00580985.1|
Molybdopterin converting factor, ...   
52   1e-05

gi|89207619|ref|ZP\_01186156.1|
GTP diphosphokinase [Bacillus wei...   
52   1e-05

gi|75177353|ref|ZP\_00697442.1|
COG0441: Threonyl-tRNA synthetase...   
51   1e-05

gi|75513782|ref|ZP\_00736158.1|
COG0441: Threonyl-tRNA synthetase...   
51   1e-05

gi|35214941|dbj|BAC92307.1|
gsr4366 [Gloeobacter violaceus PCC 7...   
51   1e-05

gi|419815|pir||JT0741
GTP-binding protein 1 - fission yeast (Sch...   
51   1e-05

gi|83319726|ref|YP\_424209.1|
threonyl-tRNA synthetase [Mycoplasm...   
51   1e-05

gi|75227717|ref|ZP\_00714452.1|
COG0441: Threonyl-tRNA synthetase...   
51   1e-05

gi|50294994|ref|XP\_449908.1|
hypothetical protein CAGL0M12991g [...    51  
2e-05

gi|66540013|ref|XP\_624466.1|
PREDICTED: similar to GA19430-PA [A...   
51   2e-05

gi|86605752|ref|YP\_474515.1|
thiamine biosynthesis protein ThiS ...   
51   2e-05

gi|75855800|ref|ZP\_00763441.1|
COG1977: Molybdopterin converting...   
51   2e-05

gi|63054708|ref|NP\_595225.2|
hypothetical protein SPBC354.01 [Sc...   
51   2e-05

gi|94414782|ref|ZP\_01294636.1|
hypothetical protein PaerP\_010033...   
51   2e-05

gi|91088325|ref|XP\_970263.1|
PREDICTED: similar to CG8340-PA [Tr...   
51   2e-05

gi|87301504|ref|ZP\_01084344.1|
molydbenum cofactor biosynthesis ...   
51   2e-05

gi|42631902|ref|ZP\_00157440.1|
COG1977: Molybdopterin converting...   
51   2e-05

gi|2507066|sp|P45309|MOAD\_HAEIN
Molybdopterin-converting factor ...   
51   2e-05

gi|68058473|gb|AAX88726.1|
molybdopterin converting factor subun...   
51   2e-05

gi|72162507|ref|YP\_290164.1|
threonyl-tRNA synthetase [Thermobif...   
51   2e-05

gi|71557339|gb|AAZ36550.1|
thiamine biosynthesis protein ThiS [P...   
50   2e-05

gi|18089152|gb|AAH20803.1|
Developmentally regulated GTP binding...   
50   2e-05

gi|147985|gb|AAA24674.1|
threonine-tRNA ligase (EC 6.1.1.3)           
50   2e-05

gi|59711549|ref|YP\_204325.1|
molybdopterin converting factor, sm...   
50   2e-05

gi|88811204|ref|ZP\_01126460.1|
hypothetical protein NB231\_10388 ...   
50   2e-05

gi|56543208|gb|AAV89362.1|
thiazole biosynthesis protein [Zymomo...   
50   2e-05

gi|60826622|gb|AAX36765.1|
developmentally regulated GTP binding...   
50   2e-05

gi|74019145|ref|ZP\_00689763.1|
Molybdopterin converting factor, ...   
50   2e-05

gi|88949122|ref|ZP\_01151740.1|
ThiS, thiamine-biosynthesis [Halo...   
50   2e-05

gi|67538984|ref|XP\_663266.1|
hypothetical protein AN5662.2 [Aspe...   
50   2e-05

gi|30585289|gb|AAP36917.1|
Homo sapiens developmentally regulate...   
50   2e-05

gi|14318437|ref|NP\_116578.1|
Threonyl-tRNA synthetase, essential...   
50   2e-05

gi|26449536|dbj|BAC41894.1|
unknown protein [Arabidopsis thaliana]    
50   2e-05

gi|4619|emb|CAA26666.1|
unnamed protein product [Saccharomyces c...   
50   2e-05

gi|75236810|ref|ZP\_00720881.1|
COG0441: Threonyl-tRNA synthetase...   
50   2e-05

gi|75256124|ref|ZP\_00727820.1|
COG0441: Threonyl-tRNA synthetase...   
50   2e-05

gi|4758796|ref|NP\_004138.1|
developmentally regulated GTP bindin...   
50   2e-05

gi|346685|pir||JC1349
GTP-binding protein DRG [validated] - mouse     
50   2e-05

gi|21646638|gb|AAM71936.1|
thiamine biosynthesis protein ThiS [C...   
50   2e-05

gi|6681225|ref|NP\_031905.1|
developmentally regulated GTP bindin...  
 50   2e-05

gi|47568924|ref|ZP\_00239616.1|
molybdopterin converting factor, ...   
50   2e-05

gi|30178247|gb|EAA05833.2|
ENSANGP00000016179 [Anopheles gambiae...   
50   2e-05

gi|88809412|ref|ZP\_01124920.1|
molydbenum cofactor biosynthesis ...   
50   2e-05

gi|98162715|ref|NP\_001027533.1|
developmentally regulated GTP bi...   
50   2e-05

gi|92888440|gb|ABE89416.1|
AAA ATPase; TGS; GTP1/OBG subdomain [...   
50   2e-05

gi|91089351|ref|XP\_972899.1|
PREDICTED: similar to CG6195-PA [Tr...   
50   2e-05

gi|26348781|dbj|BAC38030.1|
unnamed protein product [Mus musculus]    
50   2e-05

gi|73995093|ref|XP\_866345.1|
PREDICTED: similar to developmental...   
50   2e-05

gi|73995095|ref|XP\_866356.1|
PREDICTED: similar to developmental...   
50   2e-05

gi|24586704|gb|AAH39649.1|
Developmentally regulated GTP binding...   
50   2e-05

gi|73995097|ref|XP\_866370.1|
PREDICTED: similar to developmental...   
50   3e-05

gi|84361561|ref|ZP\_00986224.1|
COG0441: Threonyl-tRNA synthetase...   
50   3e-05

gi|88795121|ref|ZP\_01110822.1|
molybdopterin converting factor, ...   
50   3e-05

gi|34495641|ref|NP\_899856.1|
molybdopterin-converting factor sub...   
50   3e-05

gi|73995101|ref|XP\_866396.1|
PREDICTED: similar to developmental...   
50   3e-05

gi|57225806|gb|AAW42267.1|
cytoplasm protein, putative [Cryptoco...   
50   3e-05

gi|70606742|ref|YP\_255612.1| GTPase-like protein [Sulfolobus aci...    50  
3e-05

gi|15220911|ref|NP\_173238.1| ATP binding / tRNA ligase/ threonin...    50  
3e-05

gi|85093975|ref|XP\_959801.1| hypothetical protein [Neurospora cr...    50  
3e-05

gi|53804598|ref|YP\_113552.1| thiazole synthase [Methylococcus ca...    50  
3e-05

gi|94969084|ref|YP\_591132.1|
molybdopterin converting factor, su...   
50   3e-05

gi|33564627|emb|CAE43947.1|
conserved hypothetical protein [Bord...   
50   3e-05

gi|6453535|emb|CAB61403.1|
hypothetical protein [Homo sapiens]        
50   3e-05

gi|91227353|ref|ZP\_01261742.1|
molybdenum cofactor biosynthesis ...   
50   4e-05

gi|106888351|ref|ZP\_01355551.1|
molybdopterin converting factor,...   
50   4e-05

gi|53802819|ref|YP\_115441.1|
molybdopterin converting factor, su...   
50   4e-05

gi|38016781|gb|AAR07802.1|
theronyl-tRNA synthetase [Klebsiella ...   
50   4e-05

gi|78170141|gb|ABB27238.1|
molydbenum cofactor biosynthesis prot...  
 50   4e-05

gi|88950726|ref|ZP\_01153300.1| GTP-binding protein, HSR1-related...    50  
4e-05

gi|77918070|ref|YP\_355885.1|
molybdopterin converting factor, su...   
49   4e-05

gi|26991781|ref|NP\_747206.1|
sulfur carrier protein ThiS [Pseudo...   
49   4e-05

gi|50546703|ref|XP\_500821.1|
hypothetical protein [Yarrowia lipo...   
49   4e-05

gi|34483098|emb|CAE10097.1|
hypothetical protein [Wolinella succ...   
49   4e-05

gi|70733136|ref|YP\_262909.1|
sulfur carrier protein ThiS [Pseudo...   
49   4e-05

gi|58419045|gb|AAW71060.1|
Threonyl-tRNA synthetase [Wolbachia e...   
49   4e-05

gi|13815687|gb|AAK42533.1|
GTP binding conserved hypothetical pr...   
49   5e-05

gi|68476655|ref|XP\_717620.1|
hypothetical protein CaO19\_5083 [Ca...   
49   5e-05

gi|28807086|dbj|BAC60356.1|
molybdenum cofactor biosynthesis pro...   
49   5e-05

gi|67543768|ref|ZP\_00421699.1|
Molybdopterin converting factor, ...   
49   5e-05

gi|13883249|gb|AAK47766.1|
molybdopterin cofactor biosynthesis p...   
49   5e-05

gi|9105678|gb|AAF83592.1|
conserved hypothetical protein [Xylell...   
49   5e-05

gi|32447888|emb|CAD77406.1|
threonyl-tRNA synthetase [Rhodopirel...   
49   5e-05

gi|23024014|ref|ZP\_00063239.1|
COG0441: Threonyl-tRNA synthetase...   
49   5e-05

gi|54636104|gb|EAL25507.1|
GA21003-PA [Drosophila pseudoobscura]      
49   5e-05

gi|84362038|ref|ZP\_00986675.1|
COG1977: Molybdopterin converting...   
49   5e-05

gi|78366785|ref|ZP\_00837062.1|
Molybdopterin converting factor, ...   
49   5e-05

gi|49330166|gb|AAT60812.1|
molybdopterin converting factor, subu...   
49   5e-05

gi|6460436|gb|AAF12145.1|
molybdenum cofactor biosynthesis prote...   
49   5e-05

gi|107022548|ref|YP\_620875.1|
threonyl-tRNA synthetase [Burkhold...   
49   6e-05

gi|49330814|gb|AAT61460.1|
molybdopterin converting factor, subu...   
49   6e-05

gi|48866274|ref|ZP\_00320130.1|
COG0441: Threonyl-tRNA synthetase...   
49   6e-05

gi|76634732|ref|XP\_870904.1|
PREDICTED: similar to developmental...   
49   6e-05

gi|77951750|ref|ZP\_00816169.1|
Molybdopterin converting factor, ...   
49   6e-05

gi|77996211|gb|ABB15110.1|
molybdopterin converting factor, subu...   
49   6e-05

gi|42783907|ref|NP\_981154.1|
molybdopterin converting factor, su...   
49   6e-05

gi|29897221|gb|AAP10498.1|
Molybdopterin (MPT) converting factor...  
 49   6e-05

gi|12644208|sp|P32235|GTP1\_SCHPO
GTP-binding protein 1                
49   6e-05

gi|42782629|ref|NP\_979876.1|
molybdopterin converting factor, su...   
49   6e-05

gi|58699329|ref|ZP\_00374107.1|
threonyl-tRNA synthetase [Wolbach...   
49   6e-05

gi|88931718|ref|ZP\_01137411.1|
Threonyl-tRNA synthetase, class I...   
49   6e-05

gi|24375928|ref|NP\_719971.1|
molybdenum cofactor biosynthesis pr...   
49   7e-05

gi|71074746|ref|XP\_769907.1|
hypothetical protein GLP\_14\_19528\_2...   
49   7e-05

gi|2622747|gb|AAB86094.1| GTP-binding protein, GTP1/OBG family [...    49  
7e-05

gi|8778457|gb|AAF79465.1| F1L3.17 [Arabidopsis thaliana]               49   7e-05

gi|94414450|ref|ZP\_01294310.1| hypothetical protein PaerP\_010036...    49  
7e-05

gi|90300878|gb|EAS30509.1| hypothetical protein CIMG\_05988 [Cocc...    49  
7e-05

gi|71481492|ref|ZP\_00661198.1| ThiS, thiamine-biosynthesis [Pros...    49  
7e-05

gi|78691076|ref|ZP\_00855707.1| Molybdopterin converting factor, ...    49  
7e-05

gi|98660058|dbj|GAA02642.1| unnamed protein product [Pelotomacul...    48   7e-05

gi|11499729|ref|NP\_070971.1|
GTP-binding protein [Archaeoglobus ...   
48   7e-05

gi|76634734|ref|XP\_882198.1|
PREDICTED: similar to developmental...   
48   7e-05

gi|17981711|ref|NP\_536733.1|
CG8340-PA [Drosophila melanogaster]...   
48   8e-05

gi|49180290|gb|AAT55666.1|
molybdopterin converting factor, subu...   
48   8e-05

gi|86158536|ref|YP\_465321.1| GTP-binding protein, HSR1-related [...    48  
8e-05

gi|88942337|sp|Q7UJ52|SYT\_RHOBA Threonyl-tRNA synthetase (Threon...    48  
8e-05

gi|13877991|gb|AAK44073.1| putative developmentally regulated GT...    48  
8e-05

gi|15220113|ref|NP\_173190.1| ATDRG1 (ARABIDOPSIS THALIANA DEVELO...    48  
8e-05

gi|88184699|gb|EAQ92167.1| hypothetical protein CHGG\_00402 [Chae...    48  
9e-05

gi|21107692|gb|AAM36384.1| conserved hypothetical protein [Xanth...    48  
9e-05

gi|71143753|gb|AAZ24226.1| molybdopterin converting factor, subu...    48  
9e-05

gi|88932583|ref|ZP\_01138273.1| ThiS, thiamine-biosynthesis [Acid...    48  
9e-05

gi|2058456|gb|AAB53256.1| GTP-binding protein [Arabidopsis thali...    48  
9e-05

gi|74318145|ref|YP\_315885.1| thiamine-biosynthesis protein ThiS ...    48  
9e-05

gi|50729875|ref|XP\_416683.1| PREDICTED: similar to mitochondrial...    48  
9e-05

gi|67939265|ref|ZP\_00531772.1| ThiS, thiamine-biosynthesis [Chlo...    48  
9e-05

gi|2633802|emb|CAB13304.1| molybdopterin converting factor (subu...    48   9e-05

gi|94370179|ref|XP\_993084.1|
PREDICTED: similar to threonyl-tRNA...    48  
9e-05

gi|29898350|gb|AAP11623.1|
Molybdopterin (MPT) converting factor...   
48   9e-05

gi|311343|emb|CAA50701.1|
GTP-binding protein [Drosophila melano...   
48   1e-04

gi|49082326|gb|AAT50563.1|
PA0380 [synthetic construct]               
48   1e-04

gi|46156355|ref|ZP\_00204683.1|
COG1977: Molybdopterin converting...   
48   1e-04

gi|67919394|ref|ZP\_00512973.1|
ThiS, thiamine-biosynthesis [Chlo...   
48   1e-04

gi|46121521|ref|XP\_385315.1|
conserved hypothetical protein [Gib...   
48   1e-04

gi|39584747|emb|CAE67642.1|
Hypothetical protein CBG13201 [Caeno...   
48   1e-04

gi|46449006|gb|AAS95658.1|
hypothetical protein DVU\_1180 [Desulf...   
48   1e-04

gi|77953948|ref|ZP\_00818350.1|
ThiS, thiamine-biosynthesis [Mari...   
48   1e-04

gi|67154907|ref|ZP\_00416652.1|
ThiS, thiamine-biosynthesis [Azot...   
48   1e-04

gi|45047881|emb|CAF30999.1|
GTP1/OBG family:ATP/GTP-binding site...   
48   1e-04

gi|51974253|gb|AAU15803.1|
molybdopterin converting factor, subu...   
48   1e-04

gi|84623563|ref|YP\_450935.1|
hypothetical protein XOO\_1906 [Xant...   
48   1e-04

gi|6018399|emb|CAB57908.1|
Hypothetical protein T28D6.6 [Caenorh...   
48   1e-04

gi|67987927|gb|EAM75712.1|
Threonyl-tRNA synthetase, class IIa [...   
48   1e-04

gi|47502579|gb|AAT31255.1|
molybdopterin converting factor, subu...   
48   1e-04

gi|90303258|gb|EAS32889.1|
hypothetical protein CIMG\_03913 [Cocc...   
48   1e-04

gi|71898215|ref|ZP\_00680389.1|
ThiS, thiamine-biosynthesis [Xyle...   
48   1e-04

gi|30024666|gb|AAP13583.1|
developmentally regulated GTP-binding...   
48   1e-04

gi|76579340|gb|ABA48815.1|
TGS domain protein [Burkholderia pseu...   
48   1e-04

gi|46163808|ref|ZP\_00136054.2|
COG0441: Threonyl-tRNA synthetase...   
48   1e-04

gi|47551893|gb|AAT32729.2|
molybdopterin converting factor, subu...   
48   1e-04

gi|87201302|ref|YP\_498559.1|
thiamine biosynthesis protein ThiS ...   
48   1e-04

gi|75765030|ref|ZP\_00744342.1|
Molybdopterin converting factor, ...   
47   2e-04

gi|13883002|gb|AAK47534.1|
molybdenum cofactor biosynthesis prot...   
47   2e-04

gi|42629022|ref|ZP\_00154572.1|
COG1977: Molybdopterin converting...   
47   2e-04

gi|88946143|ref|ZP\_01149231.1|
conserved hypothetical protein [D...   
47   2e-04

gi|21109644|gb|AAM38144.1|
conserved hypothetical protein [Xanth...  
 47   2e-04

gi|89206760|ref|ZP\_01185313.1|
Molybdopterin converting factor, ...   
47   2e-04

gi|78048972|ref|YP\_365147.1|
hypothetical protein XCV3416 [Xanth...   
47   2e-04

gi|95929924|ref|ZP\_01312664.1|
thiamineS [Desulfuromonas acetoxi...   
47   2e-04

gi|84489469|ref|YP\_447701.1|
predicted GTPase [Methanosphaera st...   
47   2e-04

gi|70906111|gb|AAZ14750.1|
GTP-binding protein, putative [Leishm...   
47   2e-04

gi|83764551|dbj|BAE54695.1|
unnamed protein product [Aspergillus...   
47   2e-04

gi|78362790|gb|ABB40755.1|
molybdopterin converting factor, subu...   
47   2e-04

gi|77967366|gb|ABB08746.1|
Molybdopterin converting factor, subu...   
47   2e-04

gi|83648509|ref|YP\_436944.1|
Molybdopterin converting factor, sm...   
47   2e-04

gi|85858497|ref|YP\_460699.1|
threonyl-tRNA synthetase [Syntrophu...   
47   2e-04

gi|90416705|ref|ZP\_01224635.1|
molybdenum cofactor biosynthesis ...   
47   2e-04

gi|89204215|ref|ZP\_01182793.1|
Molybdopterin converting factor, ...   
47   2e-04

gi|11499216|ref|NP\_070453.1|
molybdopterin converting factor, su...   
47   2e-04

gi|51858004|dbj|BAD42162.1|
molybdopterin converting factor-like...   
47   2e-04

gi|58697298|ref|ZP\_00372664.1|
threonyl-tRNA synthetase [Wolbach...   
47   2e-04

gi|24213940|ref|NP\_711421.1|
threonyl-tRNA synthetase [Leptospir...   
47   2e-04

gi|84320476|ref|ZP\_00968851.1|
COG0441: Threonyl-tRNA synthetase...   
47   2e-04

gi|45658307|ref|YP\_002393.1|
threonyl-tRNA synthetase [Leptospir...   
47   2e-04

gi|76796989|ref|ZP\_00779334.1|
Phosphoribulokinase/uridine kinas...   
47   2e-04

gi|89307734|gb|EAS05722.1|
GTP1/OBG family protein [Tetrahymena ...   
47   2e-04

gi|27904019|gb|AAO26853.1|
threonyl-tRNA synthetase [Buchnera ap...   
47   2e-04

gi|94968345|ref|YP\_590393.1|
thiamine biosynthesis protein ThiS ...   
47   2e-04

gi|78193517|gb|ABB31284.1|
Thiamine S [Geobacter metallireducens...   
47   2e-04

gi|77918224|ref|YP\_356039.1|
thiamine biosynthesis protein ThiS ...   
47   2e-04

gi|84496326|ref|ZP\_00995180.1|
threonyl-tRNA synthetase [Janibac...   
47   2e-04

gi|32444387|emb|CAD74386.1|
probable molybdopterin converting fa...   
47   2e-04

gi|28851697|gb|AAO54773.1|
molybdenum cofactor biosynthesis prot...   
47   2e-04

gi|47505417|gb|AAT34093.1|
molybdopterin converting factor, subu...  
 47   3e-04

gi|44980036|gb|AAS50232.1|
AAL134Wp [Ashbya gossypii ATCC 10895]...   
47   3e-04

gi|19173579|ref|NP\_597382.1|
DEVELOPMENTALLY REGULATED GTP-BINDI...   
47   3e-04

gi|88196185|ref|YP\_501002.1|
molybdopterin converting factor, su...   
47   3e-04

gi|68129877|emb|CAJ09183.1|
developmentally regulated GTP-bindin...   
47   3e-04

gi|73919843|sp|Q73GH6|SYT\_WOLPM
Threonyl-tRNA synthetase (Threon...   
47   3e-04

gi|85859558|ref|YP\_461760.1|
GTP pyrophosphokinase / guanosine-3...   
47   3e-04

gi|50312171|ref|XP\_456117.1|
unnamed protein product [Kluyveromy...   
47   3e-04

gi|68550360|ref|ZP\_00589811.1|
ThiS, thiamine-biosynthesis [Pelo...   
47   3e-04

gi|6319281|ref|NP\_009364.1|
Member of the DRG family of GTP-bind...   
47   3e-04

gi|88948276|ref|ZP\_01151039.1|
conserved hypothetical protein [H...   
47   3e-04

gi|72049892|ref|XP\_787655.1|
PREDICTED: similar to developmental...   
47   3e-04

gi|50542888|ref|XP\_499610.1|
hypothetical protein [Yarrowia lipo...   
47   3e-04

gi|77997120|gb|ABB16019.1|
conserved hypothetical protein [Carbo...   
47   3e-04

gi|28199747|ref|NP\_780061.1|
hypothetical protein PD1878 [Xylell...   
47   3e-04

gi|71900707|ref|ZP\_00682830.1|
ThiS, thiamine-biosynthesis [Xyle...   
47   3e-04

gi|78047114|ref|YP\_363289.1|
hypothetical protein XCV1558 [Xanth...   
47   3e-04

gi|78701988|ref|ZP\_00866433.1|
ThiS, thiamine-biosynthesis [Alka...   
47   3e-04

gi|68467777|ref|XP\_722047.1|
hypothetical protein CaO19.11460 [C...   
47   3e-04

gi|76795290|ref|ZP\_00777663.1|
ThiamineS [Thermoanaerobacter eth...   
47   3e-04

gi|56315292|emb|CAI09937.1|
Molybdopterin (MPT) converting facto...   
47   3e-04

gi|82751858|ref|YP\_417599.1|
molybdopterin converting factor sma...   
46   4e-04

gi|77385555|gb|ABA77068.1|
ThiS, thiamine-biosynthesis [Pseudomo...   
46   4e-04

gi|87306448|ref|ZP\_01088595.1|
thiamine biosynthesis protein Thi...   
46   4e-04

gi|70998468|ref|XP\_753956.1|
GTP-binding protein Gtp1 [Aspergill...   
46   4e-04

gi|63258672|gb|AAY39768.1|
ThiS, thiamine-biosynthesis [Pseudomo...   
46   4e-04

gi|67935886|ref|ZP\_00528902.1|
ThiS, thiamine-biosynthesis [Chlo...   
46   4e-04

gi|91798352|gb|ABE60491.1|
thiamine biosynthesis protein ThiS [C...   
46   4e-04

gi|62088260|dbj|BAD92577.1|
developmentally regulated GTP bindin...   
46   4e-04

gi|89210063|ref|ZP\_01188456.1|
Phosphoribulokinase/uridine kinas...   
46   4e-04

gi|15607139|ref|NP\_213755.1|
Molybdopterin converting factor, sm...   
46   4e-04

gi|68561643|ref|ZP\_00600934.1|
ThiS, thiamine-biosynthesis [Rubr...    46  
4e-04

gi|57227841|gb|AAW44299.1|
cytoplasm protein, putative [Cryptoco...   
46   4e-04

gi|5104835|dbj|BAA80149.1| 410aa long hypothetical protein [Aero...    46  
4e-04

gi|94310901|ref|YP\_584111.1|
molybdopterin converting factor, su...   
46   4e-04

gi|75762421|ref|ZP\_00742290.1|
Molybdopterin converting factor, ...   
46   4e-04

gi|76791187|ref|ZP\_00773695.1|
ThiamineS [Pseudoalteromonas atla...   
46   4e-04

gi|73970001|ref|XP\_853758.1|
PREDICTED: similar to developmental...   
46   4e-04

gi|71659517|ref|XP\_821480.1|
developmentally regulated GTP-bindi...   
46   4e-04

gi|71907414|ref|YP\_285001.1|
Molybdopterin converting factor, su...   
46   4e-04

gi|17133489|dbj|BAB76052.1|
asl4353 [Nostoc sp. PCC 7120] >gi|17...   
46   5e-04

gi|19714122|gb|AAL94807.1|
Threonyl-tRNA synthetase [Fusobacteri...   
46   5e-04

gi|72037482|ref|XP\_791171.1|
PREDICTED: similar to developmental...   
46   5e-04

gi|74001348|ref|XP\_544833.2|
PREDICTED: similar to mitochondrial...   
46   5e-04

gi|52429778|gb|AAU50371.1|
TGS domain protein [Burkholderia mall...   
46   5e-04

gi|71747732|ref|XP\_822921.1|
developmentally regulated GTP-bindi...   
46   5e-04

gi|44983089|gb|AAS52292.1|
ADR372Cp [Ashbya gossypii ATCC 10895]...   
46   5e-04

gi|13423328|gb|AAK23856.1|
conserved hypothetical protein [Caulo...   
46   5e-04

gi|62319690|dbj|BAD95228.1|
GTP-binding like protein [Arabidopsi...   
46   5e-04

gi|23465302|ref|NP\_695905.1|
threonyl-tRNA synthetase [Bifidobac...   
46   5e-04

gi|86608912|ref|YP\_477674.1|
molybdopterin converting factor, su...   
46   5e-04

gi|58582994|ref|YP\_202010.1|
hypothetical protein XOO3371 [Xanth...   
46   5e-04

gi|46191233|ref|ZP\_00120366.2|
COG0441: Threonyl-tRNA synthetase...   
46   5e-04

gi|50257637|gb|EAL20342.1|
hypothetical protein CNBF1530 [Crypto...   
46   5e-04

gi|58581648|ref|YP\_200664.1|
hypothetical protein XOO2025 [Xanth...   
46   5e-04

gi|66805123|ref|XP\_636294.1|
hypothetical protein DDB0188361 [Di...   
46   5e-04

gi|91770046|ref|ZP\_01271876.1|
Molybdopterin converting factor, ...   
46   5e-04

gi|50294916|ref|XP\_449869.1|
hypothetical protein CAGL0M12122g [...   
46   5e-04

gi|29895812|gb|AAP09094.1|
Molybdopterin (MPT) converting factor...   
46   6e-04

gi|433422|dbj|BAA02978.1|
GTP-binding protein DRG [Xenopus laevi...  
 46   6e-04

gi|48425680|pdb|1SF0|A
Chain A, Backbone Solution Structure Of M...   
46   6e-04

gi|83771821|dbj|BAE61951.1|
unnamed protein product [Aspergillus...   
46   6e-04

gi|85103579|ref|XP\_961549.1|
hypothetical protein [Neurospora cr...   
46   6e-04

gi|107028970|ref|YP\_626065.1|
molybdopterin converting factor, s...   
46   6e-04

gi|21112537|gb|AAM40764.1|
conserved hypothetical protein [Xanth...   
46   6e-04

gi|90306808|gb|EAS36439.1|
threonyl-tRNA synthetase, cytoplasmic...   
45   6e-04

gi|74831330|emb|CAI39284.1|
drg\_B81 [Paramecium tetraurelia]          
45   6e-04

gi|74317044|ref|YP\_314784.1|
molybdopterin converting factor, su...   
45   6e-04

gi|62858781|ref|NP\_001016294.1|
developmentally regulated GTP bi...   
45   6e-04

gi|67678259|gb|AAH97752.1|
Unknown (protein for IMAGE:6324417) [...   
45   6e-04

gi|21112021|gb|AAM40299.1|
molybdopterin-converting factor chain...   
45   6e-04

gi|50419273|ref|XP\_458160.1|
hypothetical protein DEHA0C11990g [...   
45   6e-04

gi|89361078|ref|ZP\_01198894.1|
conserved hypothetical protein [X...   
45   6e-04

gi|23016726|ref|ZP\_00056479.1|
COG1977: Molybdopterin converting...   
45   7e-04

gi|94676542|ref|YP\_588910.1|
threonyl-tRNA synthetase [Baumannia...   
45   7e-04

gi|70731087|ref|YP\_260828.1|
molybdopterin converting factor, su...   
45   7e-04

gi|76667017|ref|XP\_593171.2|
PREDICTED: similar to mitochondrial...   
45   7e-04

gi|75701250|gb|ABA20926.1|
ThiamineS [Anabaena variabilis ATCC 2...   
45   7e-04

gi|30584259|gb|AAP36378.1|
Homo sapiens developmentally regulate...   
45   7e-04

gi|32262239|gb|AAP77287.1|
conserved hypothetical protein [Helic...   
45   7e-04

gi|71676256|ref|ZP\_00673998.1|
ThiamineS [Trichodesmium erythrae...   
45   7e-04

gi|79321186|ref|NP\_001031270.1|
GTP binding [Arabidopsis thalian...   
45   7e-04

gi|4557537|ref|NP\_001379.1|
developmentally regulated GTP bindin...   
45   7e-04

gi|12653443|gb|AAH00493.1|
Developmentally regulated GTP binding...   
45   7e-04

gi|73956213|ref|XP\_536661.2|
PREDICTED: similar to Developmental...   
45   7e-04

gi|67925013|ref|ZP\_00518396.1|
ThiamineS [Crocosphaera watsonii ...   
45   7e-04

gi|83719599|ref|YP\_442575.1|
TGS domain protein [Burkholderia th...   
45   7e-04

gi|52221213|gb|AAH82564.1|
Developmentally regulated GTP binding...  
 45   7e-04

gi|78171431|gb|ABB28527.1|
ThiS, thiamine-biosynthesis [Chlorobi...   
45   7e-04

gi|86605021|ref|YP\_473784.1|
MoaD family protein [Synechococcus ...   
45   7e-04

gi|50286275|ref|XP\_445566.1|
unnamed protein product [Candida gl...   
45   7e-04

gi|33359535|ref|NP\_578790.2|
sulfur carrier protein ThiS [Pyroco...   
45   8e-04

gi|2622634|gb|AAB85990.1|
GTP-binding protein [Methanothermobact...   
45   8e-04

gi|56379152|dbj|BAD75060.1|
molybdopterin converting factor (sub...   
45   8e-04

gi|67938835|ref|ZP\_00531354.1|
ThiS, thiamine-biosynthesis [Chlo...   
45   8e-04

gi|7441586|pir||T42381
probable GTP-binding protein - fission ye...   
45   8e-04

gi|21703234|gb|AAM76114.1|
developmentally-regulated GTP binding...   
45   8e-04

gi|49333106|gb|AAT63752.1|
molybdopterin biosynthesis protein, s...   
45   8e-04

gi|84354863|ref|ZP\_00979760.1|
COG1977: Molybdopterin converting...   
45   8e-04

gi|71836234|ref|ZP\_00676003.1|
ThiS, thiamine-biosynthesis [Pelo...   
45   8e-04

gi|28850898|gb|AAO53977.1|
thiamine biosynthesis protein ThiS [P...   
45   8e-04

gi|71894771|ref|NP\_001025805.1|
developmentally regulated GTP bi...   
45   8e-04

gi|18893126|gb|AAL81185.1|
hypothetical protein [Pyrococcus furi...   
45   9e-04

gi|19114262|ref|NP\_593350.1|
hypothetical protein SPAC9.07c [Sch...   
45   9e-04

gi|68233923|ref|ZP\_00573027.1|
Threonyl-tRNA synthetase, class I...   
45   9e-04

gi|67523577|ref|XP\_659848.1|
hypothetical protein AN2244.2 [Aspe...   
45   9e-04

gi|89901242|ref|YP\_523713.1|
thiamine biosynthesis protein ThiS ...   
45   9e-04

gi|61744139|gb|AAX55653.1|
GTP binding protein [Gibberella monil...   
45   0.001

gi|16741492|gb|AAH16561.1|
Mitochondrial ribosomal protein L39 [...   
45   0.001

gi|70993228|ref|XP\_751461.1|
threonyl-tRNA synthetase [Aspergill...   
45   0.001

gi|92118391|ref|YP\_578120.1|
thiamine biosynthesis protein ThiS ...   
45   0.001

gi|50909475|ref|XP\_466226.1|
unknown protein [Oryza sativa (japo...   
45   0.001

gi|90022254|ref|YP\_528081.1|
Helix-turn-helix, AraC type [Saccha...   
45   0.001

gi|83720736|ref|YP\_442722.1|
molybdopterin converting factor, su...   
45   0.001

gi|31560438|ref|NP\_059100.2|
mitochondrial ribosomal protein L39...   
45   0.001

gi|63255028|gb|AAY36124.1|
Molybdopterin converting factor, subu...   
45   0.001

gi|62460422|ref|NP\_001014865.1|
developmentally regulated GTP bi...   
45   0.001

gi|7271935|gb|AAF44697.1|
unknown [Mus musculus]                      
45   0.001

gi|58001333|gb|AAW60227.1| Bifunctional molybdenum cofactor bios...    45  
0.001

gi|67937987|ref|ZP\_00530517.1| ThiamineS [Chlorobium phaeobacter...    45  
0.001

gi|104779587|ref|YP\_606085.1| thiamine biosynthesis protein ThiS...    45  
0.001

gi|49484485|ref|YP\_041709.1| putative molybdopterin-synthase sma...    45  
0.001

gi|73920761|sp|Q9JKF7|RM39\_MOUSE Mitochondrial 39S ribosomal pro...    45  
0.001

gi|51976766|gb|AAU18316.1| molybdopterin biosynthesis protein, s...    45  
0.001

gi|47566905|ref|ZP\_00237623.1| molybdopterin converting factor, ...    45  
0.001

gi|48430776|gb|AAT43641.1| molybdopterin (MPT) converting factor...    45  
0.001

gi|83312895|ref|YP\_423159.1| Molybdopterin converting factor, sm...    45   0.001

gi|72114599|ref|XP\_794470.1|
PREDICTED: similar to mitochondrial...   
45   0.001

gi|91785395|ref|YP\_560601.1|
Putative sulfur transfer protein in...   
45   0.001

gi|84690418|gb|EAQ16259.1|
thiazole biosynthesis protein [Parvul...   
45   0.001

gi|95930902|ref|ZP\_01313632.1|
thiamineS [Desulfuromonas acetoxi...   
45   0.001

gi|56756577|gb|AAW26461.1|
SJCHGC06596 protein [Schistosoma japo...   
45   0.001

gi|71492602|gb|EAO24906.1|
ThiamineS [Syntrophomonas wolfei subs...   
45   0.001

gi|73661952|ref|YP\_300733.1|
molybdopterin converting factor sma...   
45   0.001

gi|68067551|ref|XP\_675731.1|
developmentally regulated GTP-bindi...   
45   0.001

gi|85709842|ref|ZP\_01040907.1|
thiazole biosynthesis protein [Er...   
45   0.001

gi|66815061|ref|XP\_641633.1|
hypothetical protein DDB0205772 [Di...   
45   0.001

gi|81299867|ref|YP\_400075.1|
thiamine biosynthesis protein ThiS ...   
45   0.001

gi|52427701|gb|AAU48294.1|
thiamine biosynthesis protein ThiS, p...   
45   0.001

gi|66967003|ref|ZP\_00414553.1|
Threonyl-tRNA synthetase, class I...   
45   0.001

gi|85667142|ref|ZP\_01029364.1|
hypothetical protein Badol\_010006...   
45   0.001

gi|72119091|gb|AAZ61354.1|
Molybdopterin converting factor, subu...   
45   0.001

gi|23126667|ref|ZP\_00108556.1|
COG1977: Molybdopterin converting...   
44   0.001

gi|67936989|ref|ZP\_00529977.1|
GTP diphosphokinase [Chlorobium p...   
44   0.001

gi|89201285|ref|ZP\_01180030.1|
Molybdopterin converting factor, ...   
44   0.001

gi|52427038|gb|AAU47631.1|
molybdopterin converting factor, subu...   
44   0.001

gi|89362382|ref|ZP\_01200190.1|
ThiS, thiamine-biosynthesis [Xant...    44  
0.001

gi|19173454|ref|NP\_597257.1|
DEVELOPMENTALLY REGULATED GTP BINDI...   
44   0.001

gi|91977895|ref|YP\_570554.1|
thiamine biosynthesis protein ThiS ...   
44   0.001

gi|78362777|gb|ABB40742.1|
thiamine biosynthesis protein ThiS [T...   
44   0.001

gi|67549235|ref|ZP\_00427105.1|
Protein of unknown function UPF01...   
44   0.001

gi|15235111|ref|NP\_195662.1|
GTP binding [Arabidopsis thaliana] ...   
44   0.002

gi|83368880|ref|ZP\_00913740.1|
protein RnfH [Rhodobacter sphaero...   
44   0.002

gi|71544970|ref|ZP\_00665947.1|
GTP-binding protein, HSR1-related...   
44   0.002

gi|14325276|dbj|BAB60180.1|
GTP-binding protein [Thermoplasma vo...   
44   0.002

gi|57638416|gb|AAW55204.1|
molybdopterin converting factor, subu...   
44   0.002

gi|89268180|emb|CAJ81960.1|
mitochondrial ribosomal protein L39 ...   
44   0.002

gi|56789558|gb|AAH88527.1|
Mitochondrial ribosomal protein L39 [...   
44   0.002

gi|68553535|ref|ZP\_00592907.1|
ThiamineS [Prosthecochloris aestu...   
44   0.002

gi|23016433|ref|ZP\_00056189.1|
COG0441: Threonyl-tRNA synthetase...   
44   0.002

gi|2654192|gb|AAC33135.1|
GTP-binding protein [Oncorhynchus tsha...   
44   0.002

gi|83720274|ref|YP\_443511.1|
thiamine biosynthesis protein ThiS ...   
44   0.002

gi|71745170|ref|XP\_827215.1|
developmentally regulated GTP-bindi...   
44   0.002

gi|77655361|gb|EAO87002.1|
ThiS, thiamine-biosynthesis [Rhodopse...   
44   0.002

gi|31418943|gb|AAH53264.1|
Developmentally regulated GTP binding...   
44   0.002

gi|83858499|ref|ZP\_00952021.1|
thiazole biosynthesis protein [Oc...   
44   0.002

gi|91203815|emb|CAJ71468.1|
similar to thiS (Thiamine biosynthes...   
44   0.002

gi|91791472|ref|YP\_561123.1|
molybdopterin converting factor, su...   
44   0.002

gi|70728421|ref|YP\_258170.1|
molybdenum cofactor biosynthesis pr...   
44   0.002

gi|27886570|ref|NP\_542984.2|
mitochondrial ribosomal protein L39...   
44   0.002

gi|55656842|ref|XP\_514842.1|
PREDICTED: similar to mitochondrial...   
44   0.002

gi|55655652|ref|XP\_531401.1|
PREDICTED: similar to mitochondrial...   
44   0.002

gi|78394909|gb|AAI07720.1|
Mitochondrial ribosomal protein L39, ...   
44   0.002

gi|34499222|ref|NP\_903437.1|
hypothetical protein CV3767 [Chromo...   
44   0.002

gi|33337737|gb|AAQ13505.1|
MSTP003 [Homo sapiens] >gi|38014060|g...  
 44   0.002

gi|40063023|gb|AAR37879.1|
molybdenum cofactor biosynthesis prot...   
44   0.002

gi|47223702|emb|CAF99311.1|
unnamed protein product [Tetraodon n...   
44   0.002

gi|88935576|ref|ZP\_01141206.1|
similar to molybdopterin converti...   
44   0.002

gi|47210849|emb|CAF89715.1|
unnamed protein product [Tetraodon n...   
44   0.002

gi|33867003|ref|NP\_898562.1|
molydbenum cofactor biosynthesis pr...   
44   0.002

gi|83594146|ref|YP\_427898.1|
Molybdopterin converting factor, su...   
44   0.002

gi|74020038|ref|ZP\_00690646.1|
Protein of unknown function UPF01...   
44   0.002

gi|10038808|dbj|BAB12843.1|
threonyl-tRNA synthetase [Buchnera a...   
44   0.002

gi|82546334|ref|YP\_410281.1|
Sulfur transfer protein [Shigella b...   
44   0.002

gi|77995330|gb|ABB14229.1|
thiamine biosynthesis protein ThiS [C...   
44   0.002

gi|76883424|gb|ABA58105.1|
ThiS, thiamine-biosynthesis [Nitrosoc...   
44   0.002

gi|78698313|ref|ZP\_00862816.1|
ThiS, thiamine-biosynthesis [Brad...   
44   0.002

gi|94496324|ref|ZP\_01302901.1|
molybdopterin converting factor, ...   
44   0.002

gi|7271933|gb|AAF44696.1|
unknown [Homo sapiens]                      
44   0.002

gi|46125727|ref|XP\_387417.1|
conserved hypothetical protein [Gib...   
44   0.002

gi|72161447|ref|YP\_289104.1|
ThiS, thiamine-biosynthesis [Thermo...   
44   0.002

gi|77967521|gb|ABB08901.1|
protein of unknown function UPF0125 [...   
44   0.002

gi|71545061|ref|ZP\_00666026.1|
ThiS, thiamine-biosynthesis [Synt...   
44   0.002

gi|19886731|gb|AAM01588.1|
Predicted GTPase, probable translatio...   
44   0.002

gi|91783438|ref|YP\_558644.1|
Molybdopterin converting factor, su...   
44   0.002

gi|98661971|dbj|GAA01064.1|
unnamed protein product [Pelotomacul...   
44   0.002

gi|71006336|ref|XP\_757834.1|
hypothetical protein UM01687.1 [Ust...   
44   0.002

gi|1905814|emb|CAA72667.1|
RnfH protein [Rhodobacter capsulatus]...   
43   0.002

gi|21220586|ref|NP\_626365.1|
hypothetical protein SCO2108 [Strep...   
43   0.002

gi|67927210|ref|ZP\_00520404.1|
Molybdopterin biosynthesis MoaE:T...   
43   0.002

gi|71555514|gb|AAZ34725.1|
molybdopterin converting factor, subu...   
43   0.002

gi|88179004|gb|EAQ86472.1|
conserved hypothetical protein [Chaet...   
43   0.002

gi|7020559|dbj|BAA91177.1|
unnamed protein product [Homo sapiens]     
43   0.002

gi|71017847|ref|XP\_759154.1|
hypothetical protein UM03007.1 [Ust...   
43   0.002

gi|84362161|ref|ZP\_00986796.1|
COG2914: Uncharacterized protein ...   
43   0.002

gi|89056408|ref|YP\_511859.1|
molybdopterin converting factor, su...    43  
0.002

gi|83593767|ref|YP\_427519.1|
ThiS, thiamine-biosynthesis [Rhodos...   
43   0.003

gi|67930072|ref|ZP\_00523248.1|
ThiS, thiamine-biosynthesis [Soli...   
43   0.003

gi|82705616|ref|XP\_727043.1|
hypothetical protein PY06396 [Plasm...   
43   0.003

gi|10640334|emb|CAC12148.1|
MoaD (involved in molybdopterin synt...   
43   0.003

gi|23504826|emb|CAD51607.1|
developmentally regulated GTP-bindin...   
43   0.003

gi|71550737|ref|ZP\_00670826.1|
ThiS, thiamine-biosynthesis [Nitr...   
43   0.003

gi|85666193|ref|ZP\_01028417.1|
hypothetical protein Badol\_010010...   
43   0.003

gi|82737924|ref|ZP\_00900767.1|
Molybdopterin converting factor, ...   
43   0.003

gi|30178981|gb|EAA03774.2|
ENSANGP00000013421 [Anopheles gambiae...   
43   0.003

gi|26988028|ref|NP\_743453.1|
molybdenum cofactor biosynthesis pr...   
43   0.003

gi|20516791|gb|AAM24972.1|
Uridine kinase [Thermoanaerobacter te...   
43   0.003

gi|18076267|emb|CAC82488.1|
molybdopterin synthase small subunit...   
43   0.003

gi|20517475|gb|AAM25599.1|
hypothetical protein TTE2469 [Thermoa...   
43   0.003

gi|84355032|ref|ZP\_00979924.1|
COG2914: Uncharacterized protein ...   
43   0.003

gi|12518920|gb|AAG59188.1|
thiamin biosynthesis, probable sulfur...   
43   0.003

gi|40062746|gb|AAR37640.1|
molybdopterin converting factor, subu...   
43   0.003

gi|89269049|emb|CAJ81597.1|
developmentally regulated GTP bindin...   
43   0.003

gi|37681783|gb|AAQ97769.1|
developmentally regulated GTP binding...   
43   0.003

gi|86749080|ref|YP\_485576.1|
thiamine biosynthesis protein ThiS ...   
43   0.003

gi|47571794|ref|ZP\_00241843.1|
COG2104: Sulfur transfer protein ...   
43   0.003

gi|77917869|ref|YP\_355684.1|
hypothetical protein Pcar\_0253 [Pel...   
43   0.003

gi|30248304|ref|NP\_840374.1|
DUF170 [Nitrosomonas europaea ATCC ...   
43   0.003

gi|50311459|ref|XP\_455754.1|
unnamed protein product [Kluyveromy...   
43   0.003

gi|71032329|ref|XP\_765806.1|
hypothetical protein TP01\_0279 [The...   
43   0.004

gi|84999454|ref|XP\_954448.1|
hypothetical protein TA21445 [Theil...   
43   0.004

gi|107028823|ref|YP\_625918.1|
protein of unknown function UPF012...   
43   0.004

gi|11496155|gb|AAF97355.1|
Obg-like protein [Thermococcus zilligii]   
43   0.004

gi|92890615|gb|ABE90425.1|
ATP-dependent DNA ligase; Metal-depen...  
 43   0.004

gi|82498768|ref|ZP\_00884224.1|
conserved hypothetical protein [C...   
43   0.004

gi|99910773|ref|ZP\_01317738.1|
hypothetical protein Bpse1\_030028...   
43   0.004

gi|88800262|ref|ZP\_01115829.1|
molybdopterin biosynthesis protei...   
43   0.004

gi|70937899|ref|XP\_739695.1|
GTP-binding protein [Plasmodium cha...   
43   0.004

gi|75255488|ref|ZP\_00727287.1|
COG2104: Sulfur transfer protein ...   
43   0.004

gi|88946144|ref|ZP\_01149232.1|
conserved hypothetical protein [D...   
43   0.004

gi|57159324|dbj|BAD85254.1|
molybdopterin converting factor, sub...   
43   0.004

gi|91773505|ref|YP\_566197.1|
Small GTP-binding protein [Methanoc...   
43   0.004

gi|77919319|ref|YP\_357134.1|
molybdopterin converting factor, su...   
43   0.004

gi|57158765|dbj|BAD84695.1|
predicted GTPase, containing TGS dom...   
43   0.004

gi|90021102|ref|YP\_526929.1|
hypothetical protein Sde\_1455 [Sacc...   
43   0.004

gi|83312024|ref|YP\_422288.1|
hypothetical protein amb2925 [Magne...   
43   0.004

gi|21356473|ref|NP\_650822.1|
CG6195-PA [Drosophila melanogaster]...   
43   0.004

gi|74314485|ref|YP\_312904.1|
Sulfur transfer protein [Shigella s...   
43   0.005

gi|91220231|ref|ZP\_01256641.1|
hypothetical protein P700755\_3102...   
43   0.005

gi|57159352|dbj|BAD85282.1|
sulfur transfer protein involved in ...   
43   0.005

gi|47226855|emb|CAG06697.1|
unnamed protein product [Tetraodon n...   
43   0.005

gi|88934468|ref|ZP\_01140111.1|
conserved hypothetical protein [G...   
43   0.005

gi|93453080|gb|EAT03559.1|
ThiS, thiamine-biosynthesis [delta pr...   
42   0.005

gi|83748544|ref|ZP\_00945564.1|
Molybdopterin converting factor, ...   
42   0.005

gi|71367699|ref|ZP\_00658218.1|
ThiS, thiamine-biosynthesis [Noca...   
42   0.005

gi|50427637|ref|XP\_462431.1|
hypothetical protein DEHA0G21725g [...   
42   0.005

gi|69951944|ref|ZP\_00639556.1|
Molybdopterin converting factor, ...   
42   0.005

gi|71077812|ref|XP\_771418.1|
GTP-binding protein [Giardia lambli...   
42   0.005

gi|89901068|ref|YP\_523539.1|
protein of unknown function UPF0125...   
42   0.005

gi|82778833|ref|YP\_405182.1|
Sulfur transfer protein involved in...   
42   0.006

gi|74421519|gb|ABA05718.1|
ThiS, thiamine-biosynthesis [Nitrobac...   
42   0.006

gi|14025577|dbj|BAB52177.1|
thiamin biosynthesis; ThiG [Mesorhiz...  
 42   0.006

gi|35505538|gb|AAH57756.1|
MGC69136 protein [Xenopus laevis] >gi...   
42   0.006

gi|23395272|gb|AAN31771.1|
molybdopterin converting factor, smal...   
42   0.006

gi|94495482|ref|ZP\_01302062.1|
thiamine biosynthesis protein Thi...   
42   0.006

gi|91212802|ref|YP\_542788.1|
hypothetical protein UTI89\_C3828 [E...   
42   0.006

gi|74831283|emb|CAI39276.1|
drg\_A82 [Paramecium tetraurelia]          
42   0.006

gi|74831277|emb|CAI39275.1|
rab\_C82 [Paramecium tetraurelia]          
42   0.006

gi|54639793|gb|EAL29195.1|
GA19430-PA [Drosophila pseudoobscura]      
42   0.006

gi|69300050|ref|ZP\_00621926.1|
ThiS, thiamine-biosynthesis [Sili...   
42   0.006

gi|49903487|gb|AAH76919.1|
Developmentally regulated GTP binding...   
42   0.006

gi|78167320|gb|ABB24418.1|
ThiS, thiamine-biosynthesis [Pelodict...   
42   0.006

gi|39947938|ref|XP\_363056.1|
hypothetical protein MG08640.4 [Mag...   
42   0.006

gi|21107237|gb|AAM35973.1|
molybdopterin-converting factor chain...   
42   0.007

gi|71838567|ref|ZP\_00678326.1|
ThiamineS [Pelobacter propionicus...   
42   0.007

gi|78046664|ref|YP\_362839.1|
molybdopterin-converting factor cha...   
42   0.007

gi|62083345|gb|AAX62397.1|
putative mitochondrial ribosomal prot...   
42   0.007

gi|48994994|gb|AAT48237.1|
sulphur carrier protein [Escherichia ...   
42   0.007

gi|77382376|gb|ABA73889.1|
ThiamineS [Pseudomonas fluorescens Pf...   
42   0.007

gi|71909473|ref|YP\_287060.1|
ThiS, thiamine-biosynthesis [Dechlo...   
42   0.007

gi|38704219|ref|NP\_944578.1|
sulfur carrier protein ThiS [Escher...   
42   0.007

gi|83311801|ref|YP\_422065.1|
Uncharacterized enzyme of thiazole ...   
42   0.007

gi|3955207|gb|AAC83142.1|
MoaD [Staphylococcus carnosus]              
42   0.008

gi|77389630|gb|ABA80814.1|
probable rnfH protein [Rhodobacter sp...   
42   0.008

gi|70940295|ref|XP\_740581.1|
GTP-binding protein [Plasmodium cha...   
42   0.008

gi|58580685|ref|YP\_199701.1|
molybdopterin-converting factor cha...   
42   0.008

gi|85715979|ref|ZP\_01046956.1|
ThiS, thiamine-biosynthesis [Nitr...   
42   0.008

gi|76801911|ref|YP\_326919.1|
probable GTP-binding protein [Natro...   
42   0.008

gi|21227149|ref|NP\_633071.1|
GTP-binding protein [Methanosarcina...   
42   0.008

gi|39936636|ref|NP\_948912.1|
putative thiamin biosynthesis ThiS ...   
42   0.008

gi|91776986|ref|YP\_546742.1|
thiamine biosynthesis protein ThiS ...   
42   0.008

gi|74019492|ref|ZP\_00690108.1|
ThiS, thiamine-biosynthesis [Burk...   
42   0.009

gi|89338808|ref|ZP\_01191573.1|
ThiS, thiamine-biosynthesis [Myco...    42  
0.009

gi|18160633|gb|AAL63972.1|
conserved hypothetical protein [Pyrob...   
42   0.009

gi|14324783|dbj|BAB59710.1|
molybdopterin converting factor subu...   
42   0.009

gi|73919765|sp|Q5P9F6|SYT\_ANAMM
Threonyl-tRNA synthetase (Threon...   
42   0.009

gi|82499654|ref|ZP\_00885094.1|
ThiS, thiamine-biosynthesis [Cald...   
42   0.009

gi|39579327|emb|CAE56931.1|
Hypothetical protein CBG24776 [Caeno...   
42   0.009

gi|77917953|ref|YP\_355768.1|
thiamine biosynthesis protein ThiS ...   
42   0.009

gi|21114375|gb|AAM42419.1|
conserved hypothetical protein [Xanth...   
42   0.009

gi|52003241|gb|AAU23183.1|
molybdopterin converting factor (subu...   
42   0.009

gi|71368764|ref|ZP\_00659250.1|
Threonyl-tRNA synthetase, class I...   
42   0.010

gi|54018820|dbj|BAD60190.1|
putative sulfur transfer protein [No...   
42   0.010

gi|50911801|ref|XP\_467308.1|
putative relA/spoT homologous prote...   
42   0.010

gi|27381767|ref|NP\_773296.1|
thiamin biosynthesis protein homolo...   
42   0.010

gi|107024240|ref|YP\_622567.1|
thiamine biosynthesis protein ThiS...   
42   0.010

gi|83372801|ref|ZP\_00917580.1|
protein RnfH [Rhodobacter sphaero...   
42   0.011

gi|68073569|ref|XP\_678699.1|
GTP-binding protein [Plasmodium ber...   
42   0.011

gi|18892299|gb|AAL80469.1|
molybdopterin converting factor, subu...   
42   0.011

gi|22295085|dbj|BAC08913.1|
molybdopterin biosynthesis protein D...   
42   0.011

gi|56680462|gb|AAV97128.1|
thiamine biosynthesis protein ThiS [S...   
42   0.011

gi|93005538|ref|YP\_579975.1|
thiamineS [Psychrobacter cryohalole...   
42   0.011

gi|17428346|emb|CAD15033.1|
probable molybdopterin mpt convertin...   
42   0.011

gi|90423668|ref|YP\_532038.1|
thiamine biosynthesis protein ThiS ...   
42   0.011

gi|70990912|ref|XP\_750305.1|
GTP binding protein [Aspergillus fu...   
41   0.011

gi|106883354|ref|ZP\_01350752.1|
ThiamineS [Psychromonas ingraham...   
41   0.011

gi|72157740|ref|XP\_788099.1|
PREDICTED: similar to developmental...   
41   0.011

gi|82539543|ref|XP\_724151.1|
developmentally regulated GTP-bindi...   
41   0.012

gi|83026442|gb|ABB96257.1|
VP15 [Zea mays]                            
41   0.012

gi|88934592|ref|ZP\_01140234.1|
conserved hypothetical protein [G...   
41   0.012

gi|78219906|gb|ABB39255.1|
conserved hypothetical protein [Desul...  
 41   0.012

gi|39941638|ref|XP\_360356.1|
hypothetical protein MG05730.4 [Mag...   
41   0.012

gi|78702657|ref|ZP\_00867087.1|
conserved hypothetical protein [A...   
41   0.012

gi|86741833|ref|YP\_482233.1|
threonyl-tRNA synthetase [Frankia s...   
41   0.012

gi|98662976|dbj|GAA00218.1|
unnamed protein product [Pelotomacul...   
41   0.013

gi|88608780|ref|YP\_505946.1|
thiamine biosynthesis protein ThiS ...   
41   0.013

gi|50877366|emb|CAG37206.1|
similar to molybdopterin converting ...   
41   0.013

gi|88951937|ref|ZP\_01154411.1|
GTP-binding protein, HSR1-related...   
41   0.013

gi|10640182|emb|CAC12034.1|
GTP-binding protein Obg related prot...   
41   0.013

gi|78497576|gb|ABB44116.1|
ThiS, thiamine-biosynthesis [Thiomicr...   
41   0.013

gi|46201345|ref|ZP\_00055205.2|
COG1977: Molybdopterin converting...   
41   0.014

gi|19918478|gb|AAM07694.1|
GTP-binding protein [Methanosarcina a...   
41   0.014

gi|72395281|gb|AAZ69554.1|
GTP-binding protein [Methanosarcina b...   
41   0.014

gi|41018426|sp|Q58728|Y1332\_METJA
Hypothetical GTP-binding prote...   
41   0.014

gi|21228746|ref|NP\_634668.1|
putative molybdopterin converting f...   
41   0.014

gi|29832636|ref|NP\_827270.1|
sulfur transfer protein involved in...   
41   0.014

gi|67154055|ref|ZP\_00415800.1|
Protein of unknown function DUF82...   
41   0.015

gi|68446507|dbj|BAE04091.1|
moaD [Staphylococcus haemolyticus JC...   
41   0.015

gi|37904746|gb|AAP57208.1|
developmentally regulated GTP-binding...   
41   0.015

gi|33356745|ref|NP\_126807.2|
sulfur carrier protein ThiS [Pyroco...   
41   0.015

gi|10640829|emb|CAC12607.1|
GTP-binding protein [Thermoplasma ac...   
41   0.015

gi|40068973|gb|AAR39308.1|
NEQ463 [Nanoarchaeum equitans Kin4-M]...   
41   0.016

gi|37904742|gb|AAP57207.1|
developmentally regulated GTP-binding...   
41   0.016

gi|10580108|gb|AAG19036.1|
GTP-binding protein homolog; Gbp5 [Ha...   
41   0.016

gi|75760851|ref|ZP\_00740866.1|
Molybdopterin converting factor, ...   
41   0.016

gi|47571785|ref|ZP\_00241834.1|
COG2914: Uncharacterized protein ...   
41   0.016

gi|91773897|ref|YP\_566589.1|
GTP-binding protein, HSR1-related [...   
41   0.017

gi|6321612|ref|NP\_011689.1|
Protein with similarity to mammalian...   
41   0.017

gi|42527791|ref|NP\_972889.1|
phosphoribulokinase/uridine kinase ...    41  
0.017

gi|28199260|ref|NP\_779574.1|
hypothetical protein PD1376 [Xylell...   
41   0.017

gi|84717489|ref|ZP\_01023548.1|
similar to Uncharacterized protei...   
41   0.018

gi|77741142|ref|ZP\_00809626.1|
ThiS, thiamine-biosynthesis [Rhod...   
41   0.018

gi|71546139|ref|ZP\_00666991.1|
conserved hypothetical protein [S...   
41   0.018

gi|84360622|ref|ZP\_00985320.1|
COG2104: Sulfur transfer protein ...   
41   0.018

gi|10175640|dbj|BAB06737.1|
molybdopterin converting factor (sub...   
41   0.018

gi|86158941|ref|YP\_465726.1|
thiamine biosynthesis protein ThiS ...   
41   0.018

gi|91784097|ref|YP\_559303.1|
hypothetical protein Bxe\_A1704 [Bur...   
40   0.019

gi|33572188|emb|CAE41734.1|
conserved hypothetical protein [Bord...   
40   0.020

gi|21672408|ref|NP\_660475.1|
threonyl-tRNA synthetase [Buchnera ...   
40   0.020

gi|33576043|emb|CAE33122.1|
conserved hypothetical protein [Bord...   
40   0.020

gi|23347498|gb|AAN29626.1|
molybdopterin converting factor, subu...   
40   0.020

gi|75236721|ref|ZP\_00720794.1|
COG2104: Sulfur transfer protein ...   
40   0.021

gi|78195384|gb|ABB33151.1|
ThiS, thiamine-biosynthesis [Geobacte...   
40   0.021

gi|31340448|sp|Q8D3C0|SYT\_WIGBR
Threonyl-tRNA synthetase (Threon...   
40   0.021

gi|56379001|dbj|BAD74909.1|
hypothetical conserved protein [Geob...   
40   0.021

gi|26111193|gb|AAN83376.1|
Thiamin biosynthesis, probable sulfur...   
40   0.021

gi|48429956|gb|AAT42821.1|
GTP binding protein [Picrophilus torr...   
40   0.021

gi|1698846|gb|AAC44620.1|
GTP-binding protein [Thermoplasma acid...   
40   0.022

gi|16331468|ref|NP\_442196.1|
hypothetical protein ssr0102 [Synec...   
40   0.022

gi|50939231|ref|XP\_479143.1|
putative RSH, disease resistance-re...   
40   0.022

gi|88603859|ref|YP\_504037.1|
GTP-binding protein, HSR1-related [...   
40   0.023

gi|88183490|gb|EAQ90958.1|
conserved hypothetical protein [Chaet...   
40   0.023

gi|86604898|ref|YP\_473661.1|
putative molybdopterin converting f...   
40   0.023

gi|92916053|ref|ZP\_01284674.1|
ThiS, thiamine-biosynthesis [Myco...   
40   0.023

gi|78196898|gb|ABB34663.1|
thiamine biosynthesis protein ThiS [S...   
40   0.023

gi|71898356|ref|ZP\_00680529.1|
Protein of unknown function UPF01...   
40   0.023

gi|9843879|emb|CAC03730.1|
RnfH protein [Pseudomonas stutzeri] >...   
40   0.023

gi|18893610|gb|AAL81606.1|
hypothetical protein [Pyrococcus furi...   
40   0.023

gi|55231348|gb|AAV46767.1|
GTP-binding protein [Haloarcula maris...   
40   0.023

gi|19074578|ref|NP\_586084.1|
THREONYL tRNA SYNTHETASE [Encephali...    40  
0.024

gi|92909569|ref|ZP\_01278346.1|
ThiS, thiamine-biosynthesis [Myco...   
40   0.024

gi|84713090|ref|ZP\_01020876.1|
Molybdopterin converting factor, ...   
40   0.025

gi|53751264|emb|CAH12675.1|
hypothetical protein [Legionella pne...   
40   0.025

gi|90574748|ref|ZP\_01231240.1|
hypothetical protein CdifQ\_020018...   
40   0.025

gi|106887565|ref|ZP\_01354862.1|
Phosphoribulokinase/uridine kina...   
40   0.026

gi|17130691|dbj|BAB73301.1|
asr1344 [Nostoc sp. PCC 7120] >gi|17...   
40   0.026

gi|9107520|gb|AAF85145.1|
conserved hypothetical protein [Xylell...   
40   0.026

gi|91785509|ref|YP\_560715.1|
hypothetical protein Bxe\_A0270 [Bur...   
40   0.027

gi|30143060|gb|AAP15447.1|
RelA/SpoT [Staphylococcus aureus subs...   
40   0.027

gi|71837964|ref|ZP\_00677726.1|
conserved hypothetical protein [P...   
40   0.027

gi|13813911|gb|AAK41039.1|
GTP binding protein [Sulfolobus solfa...   
40   0.027

gi|88703397|ref|ZP\_01101113.1|
Molybdopterin converting factor s...   
40   0.028

gi|86610242|ref|YP\_479004.1|
MoaD family protein [Synechococcus ...   
40   0.028

gi|71414858|ref|XP\_809515.1|
GTP-binding protein [Trypanosoma cr...   
40   0.028

gi|38638838|gb|AAR25659.1|
Hypothetical protein C02F5.3 [Caenorh...   
40   0.028

gi|68246504|gb|EAN28602.1|
ThiS, thiamine-biosynthesis [Magnetoc...   
40   0.029

gi|21227184|ref|NP\_633106.1|
GTP-binding protein [Methanosarcina...   
40   0.029

gi|88858884|ref|ZP\_01133525.1|
putative ThiS protein [Pseudoalte...   
40   0.029

gi|3256930|dbj|BAA29613.1|
397aa long hypothetical GTP-binding p...   
40   0.030

gi|41409997|ref|NP\_962833.1|
sulfur carrier protein ThiS [Mycoba...   
40   0.030

gi|88712833|ref|ZP\_01106918.1|
hypothetical protein FB2170\_09351...   
40   0.032

gi|88938068|ref|ZP\_01143581.1|
conserved hypothetical protein [G...   
40   0.032

gi|74019866|ref|ZP\_00690477.1|
Protein of unknown function DUF82...   
40   0.032

gi|90205897|ref|ZP\_01208535.1|
ThiS, thiamine-biosynthesis [Myco...   
40   0.032

gi|88932029|ref|ZP\_01137722.1|
conserved hypothetical protein [A...   
40   0.032

gi|103486643|ref|YP\_616204.1|
molybdopterin converting factor, s...   
40   0.033

gi|77965731|gb|ABB07111.1|
ThiS, thiamine-biosynthesis [Burkhold...   
40   0.033

gi|60495008|emb|CAH09826.1|
putative phosphoribulose/uridine kin...  
 40   0.033

gi|52218497|dbj|BAD51090.1|
uridine kinase [Bacteroides fragilis...   
40   0.033

gi|95930037|ref|ZP\_01312777.1|
conserved hypothetical protein [D...   
40   0.033

gi|67922608|ref|ZP\_00516114.1|
ThiS, thiamine-biosynthesis [Croc...   
40   0.033

gi|72395768|gb|AAZ70041.1|
GTP-binding protein [Methanosarcina b...   
40   0.035

gi|99031619|pdb|1WXQ|A
Chain A, Crystal Structure Of Gtp Binding...   
40   0.036

gi|94986022|ref|YP\_605386.1|
thiamine biosynthesis protein ThiS ...   
40   0.036

gi|71900376|ref|ZP\_00682510.1|
Protein of unknown function UPF01...   
40   0.036

gi|67527606|ref|XP\_661684.1|
hypothetical protein AN4080.2 [Aspe...   
40   0.036

gi|89092458|ref|ZP\_01165412.1|
sulfur carrier protein ThiS [Ocea...   
40   0.037

gi|77381195|gb|ABA72708.1|
MoaD [Pseudomonas fluorescens PfO-1] ...   
40   0.037

gi|89303095|gb|EAS01083.1|
GTP1/OBG family protein [Tetrahymena ...   
40   0.038

gi|71038167|gb|AAZ18475.1|
probable molybdopterin converting fac...   
40   0.038

gi|11498960|ref|NP\_070193.1|
GTP-binding protein [Archaeoglobus ...   
40   0.038

gi|82744797|ref|ZP\_00907312.1|
ThiS, thiamine-biosynthesis [Clos...   
40   0.039

gi|45047551|emb|CAF30678.1|
GTP1/OBG family:ATP/GTP-binding site...   
40   0.040

gi|88947032|ref|ZP\_01150061.1|
ThiS, thiamine-biosynthesis [Desu...   
40   0.041

gi|6460589|gb|AAF12295.1|
conserved hypothetical protein [Deinoc...   
40   0.041

gi|71544347|ref|ZP\_00665385.1|
ThiamineS [Syntrophobacter fumaro...   
40   0.042

gi|83954400|ref|ZP\_00963120.1|
thiamine biosynthesis protein Thi...   
40   0.042

gi|19918534|gb|AAM07743.1|
GTP-binding protein [Methanosarcina a...   
40   0.043

gi|18161603|gb|AAL64874.1|
conserved hypothetical protein [Pyrob...   
39   0.043

gi|23493776|dbj|BAC18745.1|
hypothetical protein [Corynebacteriu...   
39   0.045

gi|56312934|emb|CAI07579.1|
Protein rnfH [Azoarcus sp. EbN1] >gi...   
39   0.045

gi|75704967|gb|ABA24643.1|
ThiS, thiamine-biosynthesis [Anabaena...   
39   0.045

gi|33866139|ref|NP\_897698.1|
DUF170 [Synechococcus sp. WH 8102] ...   
39   0.046

gi|77919190|ref|YP\_357005.1|
hypothetical protein Pcar\_1591 [Pel...   
39   0.047

gi|93454223|gb|EAT04541.1|
ThiS, thiamine-biosynthesis [delta pr...   
39   0.047

gi|23335286|ref|ZP\_00120523.1|
COG2104: Sulfur transfer protein ...    39  
0.047

gi|11095295|gb|AAG29821.1|
RnfH protein [Azotobacter vinelandii]...   
39   0.049

gi|27360519|gb|AAO09455.1|
Sulfur transfer protein involved in t...   
39   0.050

gi|76801483|ref|YP\_326491.1|
probable GTP-binding protein [Natro...   
39   0.051

gi|91788067|ref|YP\_549019.1|
thiamineS [Polaromonas sp. JS666] >...   
39   0.052

gi|92089022|ref|ZP\_01273976.1|
ThiamineS [Lactobacillus reuteri ...   
39   0.054

gi|53753195|emb|CAH14642.1|
hypothetical protein [Legionella pne...   
39   0.056

gi|56311907|emb|CAI06552.1|
conserved hypothetical protein [Azoa...   
39   0.056

gi|53802996|ref|YP\_115212.1|
electron transport complex, H subun...   
39   0.056

gi|76794014|ref|ZP\_00776492.1|
ThiS, thiamine-biosynthesis [Pseu...   
39   0.057

gi|14591747|ref|NP\_143011.1|
sulfur carrier protein ThiS [Pyroco...   
39   0.058

gi|83589575|ref|YP\_429584.1|
MoaD [Moorella thermoacetica ATCC 3...   
39   0.058

gi|99078622|ref|YP\_611880.1|
thiamine biosynthesis protein ThiS ...   
39   0.059

gi|89100759|ref|ZP\_01173613.1|
molybdopterin converting factor, ...   
39   0.060

gi|99908378|ref|ZP\_01316082.1|
hypothetical protein Bpse1\_030045...   
39   0.060

gi|49331838|gb|AAT62484.1|
thiamine biosynthesis protein [Bacill...   
39   0.060

gi|62426385|ref|ZP\_00381513.1|
COG0441: Threonyl-tRNA synthetase...   
39   0.060

gi|17983762|gb|AAL52915.1|
hypothetical protein [Brucella melite...   
39   0.061

gi|67738583|ref|ZP\_00489224.1|
COG1656: Uncharacterized conserve...   
39   0.064

gi|76580804|gb|ABA50279.1|
Protein of unknown function family [B...   
39   0.064

gi|51856879|dbj|BAD41037.1|
conserved hypothetical protein [Symb...   
39   0.065

gi|35210963|dbj|BAC88343.1|
ycf40 [Gloeobacter violaceus PCC 742...   
39   0.067

gi|5458384|emb|CAB49872.1|
moaD-like molybdopterin converting fa...   
39   0.068

gi|72117336|gb|AAZ59599.1|
Protein of unknown function DUF82 [Ra...   
39   0.068

gi|74318176|ref|YP\_315916.1|
hypothetical protein Tbd\_2158 [Thio...   
39   0.070

gi|4262375|gb|AAD14600.1|
molybdopterin-synthase small subunit [...   
39   0.071

gi|15023548|gb|AAK78649.1|
Fision threonyl-tRNA synthetase (N-te...   
39   0.072

gi|68192288|gb|EAN06942.1|
ThiS, thiamine-biosynthesis [Mesorhiz...   
38   0.074

gi|47501163|gb|AAT29839.1|
conserved hypothetical protein [Bacil...   
38   0.074

gi|85705982|ref|ZP\_01037078.1|
thiamine biosynthesis protein Thi...   
38   0.080

gi|55229907|gb|AAV45326.1|
GTP-binding protein [Haloarcula maris...   
38   0.081

gi|88803158|ref|ZP\_01118684.1|
hypothetical protein PI23P\_11237 ...    38  
0.082

gi|78169364|gb|ABB26461.1|
ThiS, thiamine-biosynthesis [Synechoc...   
38   0.088

gi|66964609|ref|ZP\_00412178.1|
ThiS, thiamine-biosynthesis [Arth...   
38   0.089

gi|94310400|ref|YP\_583610.1|
protein of unknown function UPF0125...   
38   0.090

gi|17741061|gb|AAL43548.1|
thiamin biosynthesis protein ThiG [Ag...   
38   0.091

gi|28631173|ref|NP\_789776.1|
molybdopterin synthase small subuni...   
38   0.094

gi|82701281|ref|YP\_410847.1|
Protein of unknown function DUF82 [...   
38   0.095

gi|29894449|gb|AAP07739.1|
ThiS protein [Bacillus cereus ATCC 14...   
38   0.096

gi|14324215|dbj|BAB59143.1|
GTP1/OBG family GTP-binding protein ...   
38   0.096

gi|82745974|ref|ZP\_00908483.1|
fision threonyl-tRNA synthetase (...   
38   0.098

gi|71078434|ref|XP\_771726.1|
GTP-binding protein [Giardia lambli...   
38   0.10

gi|23130347|ref|ZP\_00112164.1|
COG2104: Sulfur transfer protein ...   
38   0.10

gi|18892333|gb|AAL80498.1|
GTP-binding protein [Pyrococcus furio...   
38   0.10

gi|89203238|ref|ZP\_01181926.1|
ThiS, thiamine-biosynthesis [Baci...   
38   0.10

gi|76156816|gb|AAX27940.2|
SJCHGC03543 protein [Schistosoma japo...   
38   0.10

gi|65318157|ref|ZP\_00391116.1|
COG2104: Sulfur transfer protein ...   
38   0.10

gi|104783496|ref|YP\_609994.1|
molybdenum cofactor biosynthesis p...   
38   0.11

gi|88601924|ref|YP\_502102.1|
Small GTP-binding protein domain [M...   
38   0.12

gi|89093577|ref|ZP\_01166525.1|
molybdopterin converting factor, ...   
38   0.12

gi|67875153|ref|ZP\_00504638.1|
ThiS, thiamine-biosynthesis [Clos...   
38   0.12

gi|84501020|ref|ZP\_00999255.1|
thiamine biosynthesis protein Thi...   
38   0.13

gi|72148811|ref|XP\_788712.1|
PREDICTED: similar to threonyl-tRNA...   
38   0.13

gi|92090350|ref|ZP\_01275216.1|
molybdopterin converting factor s...   
38   0.15

gi|29833364|ref|NP\_827998.1|
threonyl-tRNA synthetase [Streptomy...   
38   0.16

gi|23346995|gb|AAN29165.1|
thiamine biosynthesis protein ThiS [B...   
38   0.16

gi|34484090|emb|CAE11086.1|
hypothetical protein [Wolinella succ...   
37   0.17

gi|77996070|gb|ABB14969.1|
thiamine biosynthesis protein ThiS [C...   
37   0.18

gi|91793129|ref|YP\_562780.1|
thiamine biosynthesis protein ThiS ...   
37   0.18

gi|87125482|ref|ZP\_01081327.1|
hypothetical protein RS9917\_01876...   
37   0.19

gi|78700231|ref|ZP\_00864688.1|
hypothetical protein MlgDRAFT\_272...   
37   0.19

gi|89207101|ref|ZP\_01185649.1|
ThiS, thiamine-biosynthesis [Baci...   
37   0.20

gi|46202840|ref|ZP\_00052512.2|
COG2022: Uncharacterized enzyme o...   
37   0.20

gi|84489151|ref|YP\_447383.1|
hypothetical protein Msp\_0330 [Meth...   
37   0.20

gi|48430572|gb|AAT43437.1|
GTP-binding protein [Picrophilus torr...   
37   0.21

gi|107024344|ref|YP\_622671.1|
protein of unknown function DUF82 ...   
37   0.21

gi|52627727|gb|AAU26468.1| hypothetical
protein lpg0371 [Legione...    37   0.21

gi|55230921|gb|AAV46340.1|
GTP-binding protein Drg [Haloarcula m...   
37   0.21

gi|50840156|gb|AAT82823.1|
threonyl-tRNA synthetase [Propionibac...   
37   0.23

gi|68055198|ref|ZP\_00539345.1|
ThiS, thiamine-biosynthesis [Exig...   
37   0.23

gi|49238090|emb|CAF27297.1|
Thiamin biosynthesis, thiG1 [Bartone...   
37   0.23

gi|88809116|ref|ZP\_01124625.1|
hypothetical protein WH7805\_05471...   
37   0.23

gi|16761538|ref|NP\_457155.1|
hypothetical protein STY2872 [Salmo...   
37   0.23

gi|90419510|ref|ZP\_01227420.1|
molybdopterin converting factor s...   
37   0.24

gi|67666690|ref|ZP\_00463933.1|
Protein of unknown function DUF82...   
37   0.24

gi|56416396|ref|YP\_153470.1|
hypothetical protein AM035 [Anaplas...   
37   0.24

gi|82701206|ref|YP\_410772.1|
thiamine biosynthesis protein ThiS ...   
37   0.25

gi|95930338|ref|ZP\_01313075.1|
thiamine biosynthesis protein Thi...   
37   0.26

gi|16766001|ref|NP\_461616.1|
hypothetical protein STM2686 [Salmo...   
37   0.27

gi|82499138|ref|ZP\_00884589.1|
ThiS, thiamine-biosynthesis [Cald...   
37   0.28

gi|84517612|ref|ZP\_01004961.1|
Sulfur transfer protein [Prochlor...   
37   0.30

gi|92914605|ref|ZP\_01283231.1|
ThiamineS [Mycobacterium sp. KMS]...   
37   0.30

gi|88607118|ref|YP\_504702.1|
thiamine biosynthesis protein ThiS ...   
37   0.31

gi|11465486|ref|NP\_045123.1|
hypothetical protein CycaCp106 [Cya...   
37   0.31

gi|55242853|gb|EAA07061.2|
ENSANGP00000016734 [Anopheles gambiae...   
37   0.32

gi|56314521|emb|CAI09166.1|
putative tungsten-containing aldehyd...   
37   0.32

gi|91797290|gb|ABE59429.1|
molybdenum cofactor biosynthesis prot...   
37   0.33

gi|91787516|ref|YP\_548468.1|
protein of unknown function DUF82 [...   
37   0.34

gi|83952602|ref|ZP\_00961333.1|
thiamine biosynthesis protein Thi...   
37   0.35

gi|78700359|ref|ZP\_00864816.1|
Rhodanese-like [Alkalilimnicola e...   
37   0.35

gi|85716037|ref|ZP\_01047014.1|
molybdopterin converting factor, ...   
36   0.36

gi|68263525|emb|CAI37013.1|
thiamin biosynthesis ThiS [Corynebac...   
36   0.36

gi|6899256|gb|AAF30692.1|
ppGpp 3'-pyrophosphohydrolase [Ureapla...   
36   0.36

gi|46201411|ref|ZP\_00055070.2|
hypothetical protein Magn03009730...   
36   0.39

gi|57240562|ref|ZP\_00368511.1|
thiamine biosynthesis protein Thi...   
36   0.40

gi|39982460|gb|AAR33920.1|
conserved hypothetical protein [Geoba...   
36   0.40

gi|52002882|gb|AAU22824.1|
ThiS [Bacillus licheniformis ATCC 145...   
36   0.41

gi|89070177|ref|ZP\_01157505.1|
putative molybdopterin MPT conver...   
36   0.43

gi|72139438|ref|XP\_784410.1|
PREDICTED: similar to mitochondrial...   
36   0.43

gi|15621446|dbj|BAB65441.1|
400aa long hypothetical GTP-binding ...   
36   0.43

gi|86134373|ref|ZP\_01052955.1|
putative molybdopterin-synthase s...   
36   0.43

gi|77686501|ref|ZP\_00801846.1|
ThiS, thiamine-biosynthesis [Alka...   
36   0.43

gi|88860469|ref|ZP\_01135107.1|
hypothetical protein PTD2\_15632 [...   
36   0.44

gi|69936172|ref|ZP\_00631032.1|
Molybdopterin converting factor, ...   
36   0.44

gi|29540944|gb|AAO89888.1|
thiamine biosynthesis protein ThiS [C...   
36   0.44

gi|75704874|gb|ABA24550.1|
ThiamineS [Anabaena variabilis ATCC 2...   
36   0.45

gi|31418760|gb|AAH53133.1|
Unknown (protein for MGC:63888) [Dani...   
36   0.45

gi|67470824|ref|XP\_651375.1|
GTP-binding protein [Entamoeba hist...   
36   0.46

gi|67470854|ref|XP\_651389.1|
GTP-binding protein [Entamoeba hist...   
36   0.47

gi|98663026|dbj|GAA00171.1|
unnamed protein product [Pelotomacul...   
36   0.47

gi|32261593|gb|AAP76643.1|
molybdopterin converting factor [Heli...   
36   0.47

gi|47573807|ref|ZP\_00243844.1|
COG1977: Molybdopterin converting...   
36   0.51

gi|68140976|gb|EAM94269.1|
Small GTP-binding protein domain:GTP-...   
36   0.51

gi|32397914|emb|CAD73915.1|
conserved hypothetical protein [Rhod...   
36   0.51

gi|84685238|ref|ZP\_01013137.1|
molybdopterin converting factor, ...   
36   0.53

gi|85705896|ref|ZP\_01036992.1|
putative molybdopterin MPT conver...   
36   0.53

gi|77917688|ref|YP\_355503.1|
hypothetical protein Pcar\_0070 [Pel...   
36   0.55

gi|76874357|emb|CAI85578.1|
putative ThiS protein [Pseudoalterom...   
36   0.55

gi|89894584|ref|YP\_518071.1|
hypothetical protein DSY1838 [Desul...   
36   0.56

gi|30681325|ref|NP\_849354.1|
CNX7/SIR5; catalytic [Arabidopsis t...   
36   0.56

gi|88805648|ref|ZP\_01121167.1|
hypothetical protein RB2501\_14949...   
36   0.57

gi|76801893|ref|YP\_326901.1|
probable molybdopterin converting f...   
36   0.57

gi|77965627|gb|ABB07007.1|
protein of unknown function DUF82 [Bu...   
36   0.59

gi|83719003|ref|YP\_443618.1|
Protein of unknown function family ...   
35   0.62

gi|74421578|gb|ABA05777.1|
molybdopterin converting factor, subu...   
35   0.64

gi|91772430|ref|YP\_565122.1|
hypothetical protein Mbur\_0377 [Met...   
35   0.65

gi|46449919|gb|AAS96568.1| thiamine
biosynthesis protein ThiS [D...   
35   0.67

gi|1565235|emb|CAB02510.1|
Threonyl tRNA Synthetase [Bacillus su...   
35   0.67

gi|94501723|ref|ZP\_01308237.1|
molybdopterin biosynthesis [Ocean...   
35   0.68

gi|10640172|emb|CAC12024.1|
conserved hypothetical protein [Ther...   
35   0.70

gi|77978427|ref|ZP\_00833856.1|
COG2914: Uncharacterized protein ...   
35   0.70

gi|5070641|gb|AAD39227.1|
MoaD-like protein [Pseudomonas stutzer...   
35   0.70

gi|7141308|gb|AAF37283.1|
RSH3 [Arabidopsis thaliana]                 
35   0.71

gi|76874703|emb|CAI85924.1|
conserved protein of unknown functio...   
35   0.71

gi|35212628|dbj|BAC90002.1|
gsl2061 [Gloeobacter violaceus PCC 7...   
35   0.71

gi|29339839|gb|AAO77634.1|
uridine kinase (uridine monophosphoki...   
35   0.71

gi|50946481|ref|XP\_482768.1|
plastid (p)ppGpp synthase [Oryza sa...   
35   0.72

gi|33359354|ref|NP\_877809.1|
putative molybdopterin converting f...   
35   0.73

gi|47123958|gb|AAH70765.1|
MGC83793 protein [Xenopus laevis]          
35   0.73

gi|49609716|emb|CAG73149.1|
thiamine biosynthesis protein [Erwin...   
35   0.74

gi|94309119|ref|YP\_582329.1|
protein of unknown function DUF82 [...   
35   0.76

gi|15074101|emb|CAC45748.1|
PROBABLE MOLYBDOPTERIN MPT CONVERTIN...   
35   0.77

gi|7227333|gb|AAF42389.1|
thiamine biosynthesis protein ThiS [Ne...   
35   0.77

gi|62424302|ref|ZP\_00379450.1|
COG2104: Sulfur transfer protein ...   
35   0.79

gi|72118961|gb|AAZ61224.1|
Protein of unknown function UPF0125 [...   
35   0.79

gi|67479391|ref|XP\_655077.1|
GTP-binding protein [Entamoeba hist...   
35   0.80

gi|88608148|ref|YP\_506185.1|
threonyl-tRNA synthetase [Neoricket...   
35   0.82

gi|77953016|ref|ZP\_00817427.1|
conserved hypothetical protein [M...   
35   0.83

gi|84705083|ref|ZP\_01018583.1|
molybdopterin converting factor, ...   
35   0.84

gi|94309109|ref|YP\_582319.1|
thiamine biosynthesis protein ThiS ...   
35   0.88

gi|7379707|emb|CAB84274.1|
hypothetical protein NMA1005 [Neisser...   
35   0.89

gi|15645420|ref|NP\_207594.1|
molybdopterin converting factor, su...   
35   0.90

gi|7141306|gb|AAF37282.1|
RSH2 [Arabidopsis thaliana]                 
35   0.90

gi|1723343|sp|P51344|YCF40\_PORPU
Hypothetical 8.1 kDa protein yc...   
35   0.92

gi|74317772|ref|YP\_315512.1|
hypothetical protein Tbd\_1754 [Thio...   
35   0.95

gi|67475342|ref|XP\_653365.1|
threonyl-tRNA synthetase [Entamoeba...   
35   0.96

gi|89101139|ref|ZP\_01173973.1|
sulfur carrier protein ThiS [Baci...   
35   0.96

gi|84515715|ref|ZP\_01003076.1|
molybdopterin converting factor, ...   
35   0.97

gi|34498917|ref|NP\_903132.1|
hypothetical protein CV3462 [Chromo...   
35   0.97

gi|46202216|ref|ZP\_00208434.1|
COG2914: Uncharacterized protein ...   
35   0.99

gi|77958607|ref|ZP\_00822638.1|
COG2914: Uncharacterized protein ...   
35   1.00

gi|89211486|ref|ZP\_01189849.1|
cell division protein [Halothermo...   
35   1.00

gi|76802861|ref|YP\_330956.1|
GTP-binding protein [Natronomonas p...   
35   1.0

gi|86157026|ref|YP\_463811.1|
Ferredoxin [Anaeromyxobacter dehalo...    35  
1.0

gi|77683436|ref|ZP\_00798885.1|
ThiS, thiamine-biosynthesis [Alka...   
35   1.0

gi|91788303|ref|YP\_549255.1|
protein of unknown function UPF0125...   
35   1.0

gi|86154746|ref|ZP\_01072913.1|
conserved domain protein [Campylo...   
35   1.0

gi|77974741|ref|ZP\_00830280.1|
COG2914: Uncharacterized protein ...   
35   1.0

gi|23129177|ref|ZP\_00111010.1|
COG1977: Molybdopterin converting...   
35   1.1

gi|68245723|gb|EAN27838.1|
Protein of unknown function UPF0125 [...   
35   1.1

gi|46202194|ref|ZP\_00208426.1|
COG2914: Uncharacterized protein ...   
35   1.1

gi|18404800|ref|NP\_564652.1|
RSH3 (RELA/SPOT HOMOLOG 3); catalyt...   
35   1.1

gi|4587556|gb|AAD25787.1|
Similar to gi|1653162 (p)ppGpp 3-pyrop...   
35   1.1

gi|62319609|dbj|BAD95087.1|
RSH3 [Arabidopsis thaliana]               
35   1.1

gi|13540947|ref|NP\_110635.1|
hypothetical protein TVN0116 [Therm...   
35   1.1

gi|14325024|dbj|BAB59950.1|
molybdopterin converting factor subu...   
35   1.1

gi|92118611|ref|YP\_578340.1|
molybdopterin converting factor, su...   
35   1.2

gi|84490244|ref|YP\_448476.1|
predicted GTPase [Methanosphaera st...   
35   1.2

gi|14324330|dbj|BAB59258.1|
hypothetical protein [Thermoplasma v...   
35   1.2

gi|26109388|gb|AAN81590.1|
Protein yfjF [Escherichia coli CFT073... 
  35   1.2

gi|50876627|emb|CAG36467.1|
hypothetical protein [Desulfotalea p...   
35   1.2

gi|83311718|ref|YP\_421982.1|
hypothetical protein amb2619 [Magne...   
35   1.3

gi|68208686|ref|ZP\_00560784.1|
ThiS, thiamine-biosynthesis [Desu...   
35   1.3

gi|85375395|ref|YP\_459457.1|
thiazole biosynthesis protein [Eryt...   
35   1.4

gi|43450|emb|CAA33176.1|
unnamed protein product [Halobacterium ...   
35   1.4

gi|31340487|sp|Q8FEY5|YFJF\_ECOL6
UPF0125 protein yfjF >gi|752416...   
34   1.4

gi|18266202|gb|AAL67491.1|
RSH-like protein [Narcissus pseudonar...   
34   1.4

gi|33238327|gb|AAQ00393.1|
Sulfur transfer protein [Prochlorococ...   
34   1.4

gi|49531021|emb|CAG68733.1|
hypothetical protein [Acinetobacter ...   
34   1.5

gi|33354143|dbj|BAC81141.1|
plastid (p)ppGpp synthase [Oryza sat...   
34   1.5

gi|51535999|dbj|BAD38079.1|
putative plastid (p)ppGpp synthase [...   
34   1.5

gi|868027|gb|AAC43605.1|
threonyl-tRNA synthetase >gi|1098080|pr...   
34   1.5

gi|55231494|gb|AAV46913.1|
molybdopterin converting factor subun...   
34   1.5

gi|71676258|ref|ZP\_00674000.1|
MoaD, archaeal [Trichodesmium ery...   
34   1.6

gi|71674937|ref|ZP\_00672683.1|
ThiS, thiamine-biosynthesis [Tric...   
34   1.6

gi|91975759|ref|YP\_568418.1|
molybdopterin converting factor, su...   
34   1.6

gi|14027318|dbj|BAB54272.1|
molybdopterin converting factor, sub...   
34   1.7

gi|47572157|ref|ZP\_00242203.1|
COG1977: Molybdopterin converting...   
34   1.7

gi|82702330|ref|YP\_411896.1|
hypothetical protein Nmul\_A1201 [Ni...   
34   1.7

gi|34541692|ref|NP\_906171.1|
thiS protein [Porphyromonas gingiva...   
34   1.7

gi|90203237|ref|ZP\_01205883.1|
ThiamineS [Mycobacterium vanbaale...   
34   1.7

gi|68230363|ref|ZP\_00569547.1|
ThiS, thiamine-biosynthesis [Fran...   
34   1.7

gi|74313178|ref|YP\_311597.1|
hypothetical protein SSO\_2745 [Shig...   
34   1.7

gi|82777976|ref|YP\_404325.1|
hypothetical protein SDY\_2791 [Shig...   
34   1.7

gi|82545161|ref|YP\_409108.1|
hypothetical protein SBO\_2753 [Shig...   
34   1.7

gi|88950646|ref|ZP\_01153220.1|
MoaD, archaeal [Methanosaeta ther...   
34   1.8

gi|56383706|gb|AAN44172.2|
orf, conserved hypothetical protein [...   
34   1.8

gi|90591660|ref|ZP\_01247301.1|
ThiamineS [Flavobacterium johnson...   
34   1.8

gi|21220031|ref|NP\_625810.1|
threonyl-tRNA synthetase [Streptomy...   
34   1.8

gi|77957008|ref|ZP\_00821077.1|
COG2104: Sulfur transfer protein ...   
34   1.8

gi|77960648|ref|ZP\_00824510.1|
COG2104: Sulfur transfer protein ...   
34   1.8

gi|50951221|gb|AAT88922.1| threonyl-tRNA
synthetase [Leifsonia x...    34   1.8

gi|87082139|gb|AAC75667.2|
predicted protein [Escherichia coli K...   
34   1.8

gi|71907128|ref|YP\_284715.1|
hypothetical protein Daro\_1496 [Dec...   
34   1.8

gi|7379114|emb|CAB83663.1|
hypothetical protein NMA0362 [Neisser...   
34   1.9

gi|15231772|ref|NP\_188021.1|
RSH2 (RELA-SPOT HOMOLOG); catalytic...   
34   1.9

gi|85374155|ref|YP\_458217.1|
NADH dehydrogenase I, G subunit [Er...   
34   1.9

gi|77963358|ref|ZP\_00827168.1|
COG2914: Uncharacterized protein ...   
34   2.0

gi|88798339|ref|ZP\_01113924.1|
putative ThiS protein [Reinekea s...   
34   2.0

gi|32034642|ref|ZP\_00134789.1|
COG2104: Sulfur transfer protein ...   
34   2.1

gi|78221054|gb|ABB40403.1|
hypothetical protein Dde\_3610 [Desulf...   
34   2.1

gi|77684791|ref|ZP\_00800211.1|
cell division protein FtsA [Alkal...   
34   2.1

gi|33637487|gb|AAQ23899.1|
RSH2 [Nicotiana tabacum]                   
34   2.1

gi|78693266|ref|ZP\_00857780.1|
Molybdopterin converting factor, ...   
34   2.2

gi|33286618|gb|AAQ01711.1|
PdtH [Pseudomonas putida]                  
34   2.2

gi|18145266|dbj|BAB81309.1|
conserved hypothetical protein [Clos...   
34   2.2

gi|36786684|emb|CAE15750.1|
unnamed protein product [Photorhabdu...   
34   2.3

gi|83369898|ref|ZP\_00914747.1|
Molybdopterin converting factor, ...   
34   2.3

gi|89339708|ref|ZP\_01192306.1|
ThiamineS [Mycobacterium flavesce...   
34   2.3

gi|44920975|emb|CAF30211.1|
hypothetical protein [Methanococcus ...   
34   2.3

gi|15832734|ref|NP\_311507.1|
hypothetical protein ECs3480 [Esche...   
33   2.4

gi|4981276|gb|AAD35832.1|
uridine kinase-related protein [Thermo...   
33   2.4

gi|46198624|ref|YP\_004291.1|
putative thiS protein [Thermus ther...   
33   2.4

gi|39934244|ref|NP\_946520.1|
molybdopterin converting factor, su...   
33   2.4

gi|78496994|gb|ABB43534.1|
Thiamine S [Thiomicrospira denitrific...   
33   2.4

gi|59800824|ref|YP\_207536.1|
hypothetical protein NGO0380 [Neiss...   
33   2.4

gi|86359332|ref|YP\_471224.1|
NADH-ubiquinone oxidoreductase chai...   
33   2.4

gi|87119895|ref|ZP\_01075791.1|
thiamine biosynthesis protein Thi...   
33   2.4

gi|15622711|dbj|BAB66701.1|
68aa long conserved hypothetical pro...   
33   2.5

gi|88856273|ref|ZP\_01130933.1|
threonyl-tRNA synthetase [marine ...   
33   2.5

gi|18202332|sp|P58318|YFJF\_ECO57
UPF0125 protein yfjF                 
33   2.5

gi|62656441|ref|XP\_577100.1|
PREDICTED: similar to myosin XV [Ra...   
33   2.5

gi|52550524|gb|AAU84373.1|
predicted GTPase probable translation...   
33   2.5

gi|4155307|gb|AAD06323.1|
putative MOLYBDOPTERIN CONVERTING FACT...   
33   2.6

gi|83941421|ref|ZP\_00953883.1|
putative molybdopterin MPT conver...   
33   2.7

gi|83751114|ref|ZP\_00947528.1|
COG2104: Sulfur transfer protein ...   
33   2.7

gi|14424008|sp|O30213|Y022\_ARCFU
Hypothetical protein AF0022 >gi...   
33   2.7

gi|53688960|ref|ZP\_00112390.2|
COG1656: Uncharacterized conserve...   
33   2.8

gi|72162768|ref|YP\_290425.1|
hypothetical protein Tfu\_2369 [Ther...   
33   2.8

gi|85708746|ref|ZP\_01039812.1|
NADH dehydrogenase gamma subunit ...   
33   2.9

gi|89359306|ref|ZP\_01197127.1|
Molybdopterin converting factor, ...   
33   2.9

gi|83952487|ref|ZP\_00961218.1|
molybdopterin converting factor, ...   
33   2.9

gi|99078603|ref|YP\_611861.1|
molybdopterin converting factor, su...   
33   3.0

gi|30249731|ref|NP\_841801.1|
NADH dehydrogenase gamma subunit [N...   
33   3.0

gi|90994535|ref|YP\_537025.1|
hypothetical protein 71 [Porphyra y...   
33   3.1

gi|88801656|ref|ZP\_01117184.1|
putative molybdopterin-synthase s...   
33   3.2

gi|51857712|dbj|BAD41870.1|
molybdopterin converting factor smal...   
33   3.2

gi|89900702|ref|YP\_523173.1|
molybdopterin converting factor, su...   
33   3.2

gi|28210102|ref|NP\_781046.1|
uridine kinase [Clostridium tetani ...   
33   3.2

gi|10581293|gb|AAG20053.1|
Vng1848h [Halobacterium sp. NRC-1] >g...   
33   3.3

gi|67591456|ref|XP\_665553.1|
developmentally regulated GTP-bindi...   
33   3.3

gi|74018017|ref|ZP\_00688639.1|
ThiamineS [Burkholderia ambifaria...   
33   3.5

gi|91068460|gb|ABE04182.1|
NADH dehydrogenase I chain G [Rickett...   
33   3.6

gi|56544278|gb|AAV90432.1|
conserved hypothetical protein [Zymom...   
33   3.6

gi|77920460|ref|YP\_358275.1|
hypothetical protein Pcar\_2870 [Pel...   
33   3.6

gi|67664217|ref|ZP\_00461493.1|
ThiamineS [Burkholderia cenocepac...   
33   3.6

gi|89298985|gb|EAR96973.1|
threonyl-tRNA synthetase family prote...   
33   3.7

gi|55771533|dbj|BAD69974.1|
molybdopterin converting factor, sub...   
33   3.7

gi|72117326|gb|AAZ59589.1|
ThiS, thiamine-biosynthesis [Ralstoni...   
33   3.7

gi|94490916|ref|ZP\_01298140.1|
hypothetical protein CburD\_010020...   
33   3.8

gi|42543908|pdb|1V8C|D
Chain D, Crystal Structure Of Moad Relate...   
33   3.8

gi|22294706|dbj|BAC08535.1|
tsr0983 [Thermosynechococcus elongat...   
33   3.8

gi|28808050|dbj|BAC61287.1|
ThiS protein [Vibrio parahaemolyticu...   
33   3.8

gi|46200137|ref|YP\_005804.1|
molybdopterin converting factor, sm...   
33   3.8

gi|77995664|gb|ABB14563.1|
putative cell division protein FtsA [...   
33   3.8

gi|27904127|gb|AAO26961.1|
hypothetical protein bbp\_234 [Buchner...   
33   3.9

gi|22294532|dbj|BAC08362.1|
ycf40 [Thermosynechococcus elongatus...   
33   3.9

gi|10581690|gb|AAG20393.1|
Vng2279h [Halobacterium sp. NRC-1] >g...   
33   3.9

gi|23011959|ref|ZP\_00052165.1|
COG2104: Sulfur transfer protein ...   
33   4.0

gi|86137736|ref|ZP\_01056312.1|
hypothetical protein MED193\_07738...   
33   4.1

gi|45435745|gb|AAS61303.1|
conserved hypothetical protein [Yersi...   
33   4.1

gi|33634551|emb|CAE20537.1|
DUF170 [Prochlorococcus marinus str....   
33   4.1

gi|91775496|ref|YP\_545252.1|
protein of unknown function UPF0125...   
33   4.1

gi|86739580|ref|YP\_479980.1|
thiamine S [Frankia sp. CcI3] >gi|8...   
33   4.2

gi|66356360|ref|XP\_625327.1|
DRG-like GTPase fused to an RNA-bin...   
33   4.3

gi|70606433|ref|YP\_255303.1|
GTPase [Sulfolobus acidocaldarius D...   
33   4.4

gi|85059780|ref|YP\_455482.1|
hypothetical protein SG1802 [Sodali...   
33   4.4

gi|84713026|ref|ZP\_01020831.1|
ThiS, thiamine-biosynthesis [Pola...   
33   4.5

gi|77632096|ref|ZP\_00794682.1|
COG2914: Uncharacterized protein ...   
33   4.5

gi|7491710|pir||T40599
hypothetical protein SPBC649.06 - fission...   
33   4.5

gi|57636146|gb|AAW52934.1|
thiamine biosynthesis protein ThiS [S...   
33   4.6

gi|6478160|emb|CAA19048.2|
gtp1 [Schizosaccharomyces pombe]           
33   4.6

gi|56909742|dbj|BAD64269.1|
thiamine biosynthesis protein ThiS [...   
33   4.6

gi|49239551|emb|CAF25908.1|
Thiamin biosynthesis, thiG1 [Bartone...   
33   4.7

gi|6226383|sp|O29699|Y552\_ARCFU
Hypothetical UPF0084 protein AF0...   
33   4.7

gi|88704211|ref|ZP\_01101925.1|
conserved hypothetical protein [g...   
33   4.8

gi|78701201|ref|ZP\_00865653.1|
conserved hypothetical protein [A...   
33   4.8

gi|91773385|ref|YP\_566077.1|
Molybdopterin converting factor sma...   
33   4.8

gi|87119518|ref|ZP\_01075415.1|
LPS biosynthesis protein RfaE [Ma...   
33   4.9

gi|59802314|ref|YP\_209026.1|
hypothetical protein NGO2006 [Neiss...   
33   5.1

gi|88931870|ref|ZP\_01137563.1|
Initiation factor 2:Small GTP-bin...   
32   5.4

gi|66811416|ref|XP\_639888.1|
hypothetical protein DDB0186363 [Di...   
32   5.4

gi|10639813|emb|CAC11785.1|
hypothetical protein [Thermoplasma a...   
32   5.6

gi|86748300|ref|YP\_484796.1|
molybdopterin converting factor, su...   
32   5.6

gi|68193704|gb|EAN08356.1|
Molybdopterin converting factor, subu...   
32   5.7

gi|39982776|gb|AAR34235.1|
moaD family protein [Geobacter sulfur...   
32   5.7

gi|68129461|emb|CAJ08004.1|
ubiquitin related modifier (urm1)-li...   
32   5.8

gi|23023750|ref|ZP\_00062981.1|
COG2104: Sulfur transfer protein ...   
32   5.8

gi|86357115|ref|YP\_469007.1|
molybdopterin converting factor sub...   
32   6.0

gi|83749959|ref|ZP\_00946913.1|
Hypothetical Protein RRSL\_00102 [...   
32   6.1

gi|10174049|dbj|BAB05151.1|
BH1432 [Bacillus halodurans C-125] >...   
32   6.3

gi|77742224|ref|ZP\_00810703.1|
Molybdopterin converting factor, ...   
32   6.4

gi|88938982|ref|ZP\_01144434.1|
molybdopterin converting factor, ...   
32   6.4

gi|28271030|emb|CAD63935.1|
molybdopterin biosynthesis protein, ...   
32   6.5

gi|49530467|emb|CAG68179.1|
C-terminally thiocarboxylated form i...   
32   6.6

gi|102192177|ref|ZP\_01347989.1|
hypothetical protein RcanM\_01000...   
32   6.6

gi|88949835|ref|ZP\_01152449.1|
protein RnfH [Halorhodospira halo...   
32   6.6

gi|77382082|gb|ABA73595.1|
Twin-arginine translocation pathway s...   
32   6.6

gi|91762145|ref|ZP\_01264110.1|
ThiS family protein [Candidatus P...   
32   6.7

gi|2984004|gb|AAC07549.1|
threonyl-tRNA synthetase [Aquifex aeol...   
32   6.9

gi|107022979|ref|YP\_621306.1|
thiamineS [Burkholderia cenocepaci...   
32   7.2

gi|56178887|gb|AAV81609.1|
Thiamine biosynthesis protein ThiS [I...   
32   7.3

gi|78364037|gb|ABB42002.1|
Protein of unknown function UPF0125 [...   
32   7.4

gi|77389071|gb|ABA80256.1|
putative molybdopterin MPT converting...   
32   7.4

gi|77964628|gb|ABB06009.1|
Thiamine S [Burkholderia sp. 383] >gi...   
32   7.8

gi|4457226|gb|AAD21202.1|
MoaD [Rhodobacter capsulatus]               
32   7.8

gi|87311482|ref|ZP\_01093601.1|
hypothetical protein DSM3645\_0176...   
32   8.2

gi|57168917|ref|ZP\_00368047.1|
thiamine biosynthesis protein Thi...   
32   8.2

gi|104782273|ref|YP\_608771.1|
aminotransferase, class V [Pseudom...   
32   8.3

gi|86139074|ref|ZP\_01057645.1|
molybdopterin converting factor, ...   
32   8.9

gi|41406904|ref|NP\_959740.1|
MoaD2 [Mycobacterium avium subsp. p...   
32   9.2

gi|46108760|ref|XP\_381438.1|
hypothetical protein FG01262.1 [Gib...   
32   9.5

gi|32403724|ref|XP\_322475.1|
hypothetical protein [Neurospora cr...   
32   9.5

gi|57166737|gb|AAW35516.1|
thiamine biosynthesis protein ThiS [C...   
32   9.5

gi|83854898|ref|ZP\_00948428.1|
molybdopterin converting factor, ...   
32   9.9

gi|88795306|ref|ZP\_01111005.1|
hypothetical protein MADE\_19606 [...   
32   10.0

gi|86741757|ref|YP\_482157.1|
thiamine biosynthesis protein ThiS ...   
32   10.0

gi|56179014|gb|AAV81736.1|
Uncharacterized conserved protein [Id...   
32   10.0

 

------------------------------------------------------------

Reference for
composition-based statistics:

Schaffer, Alejandro A., L.
Aravind, Thomas L. Madden,

Sergei Shavirin, John L.
Spouge, Yuri I. Wolf,

Eugene V. Koonin, and Stephen F. Altschul (2001),

"Improving the
accuracy of PSI-BLAST protein database searches with

        composition-based statistics and other
refinements",  Nucleic Acids Res.
29:2994-3005.

 

Query=
gi|\_PBP\_76556246|   [Tail assembly
protein I]

         (85 letters)

 

Database: All non-redundant
GenBank CDS

translations+PDB+SwissProt+PIR+PRF
excluding environmental samples

           3,682,060 sequences; 1,264,608,837
total letters

 

Results
from round 17

 

gi|86610242|ref|YP\_479004.1|
MoaD family protein [Synechococcus ...   
91   2e-17

gi|86605021|ref|YP\_473784.1|
MoaD family protein [Synechococcus ...   
90   2e-17

gi|35212628|dbj|BAC90002.1|
gsl2061 [Gloeobacter violaceus PCC 7...   
87   2e-16

gi|67922943|ref|ZP\_00516439.1|
ThiamineS [Crocosphaera watsonii ...   
86   3e-16

gi|16330728|ref|NP\_441456.1|
hypothetical protein slr0821 [Synec...   
86   4e-16

gi|22294706|dbj|BAC08535.1|
tsr0983 [Thermosynechococcus elongat...   
85   8e-16

gi|88932029|ref|ZP\_01137722.1|
conserved hypothetical protein [A...   
84   2e-15

gi|81300590|ref|YP\_400798.1|
hypothetical protein Synpcc7942\_178...   
84   2e-15

gi|71676258|ref|ZP\_00674000.1|
MoaD, archaeal [Trichodesmium ery...   
83   3e-15

gi|23129177|ref|ZP\_00111010.1|
COG1977: Molybdopterin converting...   
83   3e-15

gi|90579138|ref|ZP\_01234948.1|
hypothetical protein VAS14\_05513 ...   
83   4e-15

gi|57160377|dbj|BAD86307.1|
molybdopterin converting factor, sub...   
83   4e-15

gi|91204446|emb|CAJ70946.1|
similar to molybdopterin synthase su...   
82   5e-15

gi|75704874|gb|ABA24550.1|
ThiamineS [Anabaena variabilis ATCC 2... 
  82   6e-15

gi|68545076|ref|ZP\_00584627.1|
Molybdopterin converting factor, ...   
82   9e-15

gi|18892299|gb|AAL80469.1|
molybdopterin converting factor, subu...   
81   1e-14

gi|72162768|ref|YP\_290425.1|
hypothetical protein Tfu\_2369 [Ther...   
80   2e-14

gi|11499216|ref|NP\_070453.1|
molybdopterin converting factor, su...   
80   2e-14

gi|89073364|ref|ZP\_01159888.1|
hypothetical protein SKA34\_20482 ...   
80   3e-14

gi|92914605|ref|ZP\_01283231.1|
ThiamineS [Mycobacterium sp. KMS]...   
79   4e-14

gi|56383279|gb|AAN42369.2|
molybdopterin biosynthesis protein D ...   
79   4e-14

gi|14325024|dbj|BAB59950.1|
molybdopterin converting factor subu...   
79   4e-14

gi|82776146|ref|YP\_402493.1|
molybdopterin biosynthesis [Shigell...   
79   5e-14

gi|71555514|gb|AAZ34725.1|
molybdopterin converting factor, subu...   
79   5e-14

gi|5458838|emb|CAB50325.1|
moaD molybdopterin synthase, small su...   
79   6e-14

gi|49612264|emb|CAG75714.1|
molybdopterin converting factor subu...   
79   6e-14

gi|23016726|ref|ZP\_00056479.1|
COG1977: Molybdopterin converting...   
79   7e-14

gi|18160633|gb|AAL63972.1|
conserved hypothetical protein [Pyrob...   
79   7e-14

gi|107028970|ref|YP\_626065.1|
molybdopterin converting factor, s...   
79   7e-14

gi|13880985|gb|AAK45641.1| conserved
hypothetical protein [Mycob...    79   7e-14

gi|15830116|ref|NP\_308889.1|
molybdopterin biosynthesis protein ...   
79   7e-14

gi|57159324|dbj|BAD85254.1|
molybdopterin converting factor, sub...   
79   7e-14

gi|74019145|ref|ZP\_00689763.1|
Molybdopterin converting factor, ...   
78   8e-14

gi|88950646|ref|ZP\_01153220.1|
MoaD, archaeal [Methanosaeta ther...   
78   8e-14

gi|35213985|dbj|BAC91354.1|
gsl3413 [Gloeobacter violaceus PCC 7...   
78   1e-13

gi|77814639|ref|ZP\_00813897.1|
Molybdopterin converting factor, ...   
78   1e-13

gi|19915596|gb|AAM05120.1|
predicted protein [Methanosarcina ace...   
78   1e-13

gi|72395205|gb|AAZ69478.1|
molybdopterin converting factor small...   
78   1e-13

gi|62179373|ref|YP\_215790.1|
molybdopterin biosynthesis protein ...   
78   1e-13

gi|10640172|emb|CAC12024.1|
conserved hypothetical protein [Ther...   
78   1e-13

gi|33359306|ref|NP\_877770.1|
putative molybdopterin converting f...   
78   1e-13

gi|77967366|gb|ABB08746.1|
Molybdopterin converting factor, subu...   
78   1e-13

gi|67543768|ref|ZP\_00421699.1|
Molybdopterin converting factor, ...   
77   1e-13

gi|86739580|ref|YP\_479980.1|
thiamine S [Frankia sp. CcI3] >gi|8...   
77   1e-13

gi|16764168|ref|NP\_459783.1|
molybdopterin biosynthetic protein ...   
77   2e-13

gi|68541239|ref|ZP\_00580985.1|
Molybdopterin converting factor, ...   
77   2e-13

gi|90411674|ref|ZP\_01219684.1|
hypothetical protein P3TCK\_16464 ...   
77   2e-13

gi|16759729|ref|NP\_455346.1|
molybdopterin converting factor, su...   
77   2e-13

gi|89339708|ref|ZP\_01192306.1|
ThiamineS [Mycobacterium flavesce...   
77   2e-13

gi|75189826|ref|ZP\_00703093.1|
COG1977: Molybdopterin converting...   
77   2e-13

gi|42011|emb|CAA49864.1|
moaD [Escherichia coli]                      
77   2e-13

gi|32039081|ref|ZP\_00137353.1|
COG1977: Molybdopterin converting...   
77   2e-13

gi|88800262|ref|ZP\_01115829.1|
molybdopterin biosynthesis protei...   
77   2e-13

gi|84362038|ref|ZP\_00986675.1|
COG1977: Molybdopterin converting...   
77   2e-13

gi|90407696|ref|ZP\_01215875.1|
molybdopterin biosynthesis protei...   
77   2e-13

gi|1787002|gb|AAC73871.1|
molybdopterin synthase, small subunit ...   
77   3e-13

gi|9950101|gb|AAG07304.1|
molybdopterin converting factor, small...   
76   3e-13

gi|27362554|gb|AAO11408.1|
Molybdenum cofactor biosynthesis prot...   
76   3e-13

gi|52696120|pdb|1VJK|A
Chain A, Putative Molybdopterin Convertin...   
76   3e-13

gi|68231908|ref|ZP\_00571067.1|
ThiamineS [Frankia sp. EAN1pec] >...   
76   4e-13

gi|34495641|ref|NP\_899856.1|
molybdopterin-converting factor sub...   
76   4e-13

gi|49082512|gb|AAT50656.1|
PA3917 [synthetic construct]               
76   4e-13

gi|30749759|pdb|1NVI|D
Chain D, Orthorhombic Crystal Form Of Mol...   
76   4e-13

gi|21226933|ref|NP\_632855.1|
Molybdopterin converting factor sma...   
76   4e-13

gi|75236193|ref|ZP\_00720310.1|
COG1977: Molybdopterin converting...   
76   4e-13

gi|83720736|ref|YP\_442722.1|
molybdopterin converting factor, su...   
76   4e-13

gi|9655490|gb|AAF94186.1|
molybdenum cofactor biosynthesis prote...   
76   4e-13

gi|32033743|ref|ZP\_00134038.1|
COG1977: Molybdopterin converting...   
76   4e-13

gi|94415786|ref|ZP\_01295621.1|
hypothetical protein PaerP\_010023...   
76   4e-13

gi|78693266|ref|ZP\_00857780.1|
Molybdopterin converting factor, ...   
76   4e-13

gi|18892532|gb|AAL80667.1|
molybdopterin converting factor, subu...   
76   5e-13

gi|78691076|ref|ZP\_00855707.1|
Molybdopterin converting factor, ...   
76   5e-13

gi|75823609|ref|ZP\_00753099.1|
COG1977: Molybdopterin converting...    76  
5e-13

gi|87303475|ref|ZP\_01086258.1|
hypothetical protein WH5701\_09450...   
76   5e-13

gi|30250278|ref|NP\_842348.1|
DUF170 [Nitrosomonas europaea ATCC ...   
76   5e-13

gi|77976770|ref|ZP\_00832242.1|
COG1977: Molybdopterin converting...   
75   5e-13

gi|90203237|ref|ZP\_01205883.1|
ThiamineS [Mycobacterium vanbaale...   
75   6e-13

gi|24375928|ref|NP\_719971.1|
molybdenum cofactor biosynthesis pr...   
75   6e-13

gi|67932283|ref|ZP\_00525430.1|
ThiamineS [Solibacter usitatus El...   
75   6e-13

gi|46200137|ref|YP\_005804.1|
molybdopterin converting factor, sm...   
75   6e-13

gi|28851697|gb|AAO54773.1|
molybdenum cofactor biosynthesis prot...   
75   7e-13

gi|75819545|ref|ZP\_00749620.1|
COG1977: Molybdopterin converting...   
75   7e-13

gi|77951750|ref|ZP\_00816169.1|
Molybdopterin converting factor, ...   
75   7e-13

gi|23467044|ref|ZP\_00122629.1|
COG1977: Molybdopterin converting...   
75   7e-13

gi|76258732|ref|ZP\_00766386.1|
ThiamineS [Chloroflexus aurantiac...   
75   7e-13

gi|29830476|ref|NP\_825110.1|
hypothetical protein SAV3933 [Strep...   
75   8e-13

gi|71369077|ref|ZP\_00659547.1|
ThiamineS [Nocardioides sp. JS614...   
75   8e-13

gi|55771533|dbj|BAD69974.1|
molybdopterin converting factor, sub...   
75   8e-13

gi|83312895|ref|YP\_423159.1|
Molybdopterin converting factor, sm...   
75   8e-13

gi|33356787|ref|NP\_127095.2|
molybdopterin converting factor, su...   
75   8e-13

gi|17428346|emb|CAD15033.1|
probable molybdopterin mpt convertin...   
75   8e-13

gi|52427038|gb|AAU47631.1|
molybdopterin converting factor, subu...   
75   9e-13

gi|83645616|ref|YP\_434051.1|
Molybdopterin converting factor, sm...   
75   1e-12

gi|77742224|ref|ZP\_00810703.1|
Molybdopterin converting factor, ...   
75   1e-12

gi|75210621|ref|ZP\_00710769.1|
COG1977: Molybdopterin converting...   
75   1e-12

gi|83748544|ref|ZP\_00945564.1|
Molybdopterin converting factor, ...   
75   1e-12

gi|84319278|ref|ZP\_00967677.1|
COG0315: Molybdenum cofactor bios...   
75   1e-12

gi|106891734|ref|ZP\_01358916.1|
ThiamineS [Roseiflexus sp. RS-1]...   
74   1e-12

gi|5458384|emb|CAB49872.1|
moaD-like molybdopterin converting fa...   
74   1e-12

gi|62179570|ref|YP\_215987.1|
Gifsy-2 prophage probable tail asse...   
74   1e-12

gi|71907414|ref|YP\_285001.1|
Molybdopterin converting factor, su...    74  
2e-12

gi|21222687|ref|NP\_628466.1|
hypothetical protein SCO4294 [Strep...   
74   2e-12

gi|16764407|ref|NP\_460022.1|
probable tail assembly protein [Pha...   
74   2e-12

gi|46912747|emb|CAG19537.1|
hypothetical protein [Photobacterium...   
74   2e-12

gi|12620119|gb|AAG60573.1|
putative molybdenum cofactor biosynth...   
74   2e-12

gi|94501723|ref|ZP\_01308237.1|
molybdopterin biosynthesis [Ocean...   
74   2e-12

gi|21221358|ref|NP\_627137.1|
hypothetical protein SCO2911 [Strep...   
74   2e-12

gi|55231494|gb|AAV46913.1|
molybdopterin converting factor subun...   
74   2e-12

gi|11933033|emb|CAC19353.1|
hypothetical protein [Streptomyces h...   
74   2e-12

gi|56315292|emb|CAI09937.1|
Molybdopterin (MPT) converting facto...   
74   2e-12

gi|59711549|ref|YP\_204325.1|
molybdopterin converting factor, sm...   
74   2e-12

gi|36784880|emb|CAE13794.1|
molybdopterin [mpt] converting facto...   
74   2e-12

gi|45435691|gb|AAS61249.1|
molybdopterin [mpt] converting factor...   
74   2e-12

gi|5070641|gb|AAD39227.1| MoaD-like
protein [Pseudomonas stutzer...   
73   3e-12

gi|91775615|ref|YP\_545371.1|
molybdopterin converting factor, su...   
73   3e-12

gi|14027318|dbj|BAB54272.1|
molybdopterin converting factor, sub...   
73   3e-12

gi|71366890|ref|ZP\_00657425.1|
ThiamineS [Nocardioides sp. JS614...   
73   3e-12

gi|91975759|ref|YP\_568418.1|
molybdopterin converting factor, su...   
73   3e-12

gi|33152460|ref|NP\_873813.1|
molybdopterin converting factor sub...   
73   3e-12

gi|42543908|pdb|1V8C|D
Chain D, Crystal Structure Of Moad Relate...   
73   4e-12

gi|91791472|ref|YP\_561123.1|
molybdopterin converting factor, su...   
73   4e-12

gi|46200249|ref|YP\_005916.1|
molybdopterin (MPT) converting fact...   
73   4e-12

gi|28192390|gb|AAL65287.1| QbsE [Pseudomonas fluorescens]              73   4e-12

gi|82702099|ref|YP\_411665.1| ThiamineS [Nitrosospira multiformis...    73   4e-12

gi|91214077|ref|YP\_544063.1|
putative tail component of prophage...   
72   5e-12

gi|15156160|gb|AAK86930.1|
AGR\_C\_2086p [Agrobacterium tumefacien...   
72   5e-12

gi|52307130|gb|AAU37630.1|
MoaD protein [Mannheimia succinicipro...   
72   5e-12

gi|18159566|gb|AAL62982.1|
molybdenum cofactor biosynthesis prot...   
72   5e-12

gi|106883354|ref|ZP\_01350752.1|
ThiamineS [Psychromonas ingraham...    72   5e-12

gi|12720897|gb|AAK02707.1|
MoaD [Pasteurella multocida subsp. mu...   
72   5e-12

gi|21228746|ref|NP\_634668.1|
putative molybdopterin converting f...   
72   5e-12

gi|74421578|gb|ABA05777.1|
molybdopterin converting factor, subu...   
72   6e-12

gi|39934244|ref|NP\_946520.1|
molybdopterin converting factor, su...   
72   7e-12

gi|33359354|ref|NP\_877809.1|
putative molybdopterin converting f...   
72   7e-12

gi|55771441|dbj|BAD69882.1|
molybdenum cofactor biosynthesis pro...   
72   7e-12

gi|91210538|ref|YP\_540524.1|
putative tail component of prophage...   
72   7e-12

gi|16764285|ref|NP\_459900.1|
putative phage tail assembly protei...   
72   7e-12

gi|72160631|ref|YP\_288288.1|
hypothetical protein Tfu\_0227 [Ther...   
72   7e-12

gi|85716037|ref|ZP\_01047014.1|
molybdopterin converting factor, ...   
72   7e-12

gi|91773385|ref|YP\_566077.1|
Molybdopterin converting factor sma...   
72   8e-12

gi|78366785|ref|ZP\_00837062.1|
Molybdopterin converting factor, ...   
72   8e-12

gi|77957352|ref|ZP\_00821410.1|
COG1977: Molybdopterin converting...   
72   8e-12

gi|26988028|ref|NP\_743453.1|
molybdenum cofactor biosynthesis pr...   
72   8e-12

gi|82737924|ref|ZP\_00900767.1|
Molybdopterin converting factor, ...   
72   8e-12

gi|88793099|ref|ZP\_01108816.1|
molybdopterin converting factor, ...   
72   9e-12

gi|78362790|gb|ABB40755.1|
molybdopterin converting factor, subu...   
72   9e-12

gi|18076267|emb|CAC82488.1|
molybdopterin synthase small subunit...   
72   9e-12

gi|76261574|ref|ZP\_00769179.1|
Molybdopterin converting factor, ...    72   1e-11

gi|76556246|emb|CAD88872.2| putative tail component [Phage BP-4795]    72  
1e-11

gi|29831706|ref|NP\_826340.1| hypothetical protein SAV5163 [Strep...    71  
1e-11

gi|91220231|ref|ZP\_01256641.1| hypothetical protein P700755\_3102...    71  
1e-11

gi|15830095|ref|NP\_308868.1| putative tail assembly protein [Esc...    71  
1e-11

gi|23347498|gb|AAN29626.1|
molybdopterin converting factor, subu...   
71   1e-11

gi|9626263|ref|NP\_040599.1|
tail component [Enterobacteria phage...   
71   1e-11

gi|26109404|gb|AAN81607.1|
Putative tail component of prophage [...   
71   1e-11

gi|94310901|ref|YP\_584111.1|
molybdopterin converting factor, su...   
71   2e-11

gi|33286618|gb|AAQ01711.1|
PdtH [Pseudomonas putida]                   71  
2e-11

gi|75431111|ref|ZP\_00732938.1|
molybdopterin (MPT) converting fa...   
71   2e-11

gi|12515098|gb|AAG56210.1|
putative tail component of prophage C...   
71   2e-11

gi|84393380|ref|ZP\_00992139.1|
Molybdenum cofactor biosynthesis ...    70   2e-11

gi|15832199|ref|NP\_310972.1|
putative tail assembly protein [Esc...   
70   2e-11

gi|77973923|ref|ZP\_00829467.1|
COG1977: Molybdopterin converting...   
70   2e-11

gi|89900702|ref|YP\_523173.1|
molybdopterin converting factor, su...   
70   2e-11

gi|94496324|ref|ZP\_01302901.1|
molybdopterin converting factor, ...   
70   2e-11

gi|86147951|ref|ZP\_01066255.1|
Molybdenum cofactor biosynthesis ...   
70   2e-11

gi|77960819|ref|ZP\_00824674.1|
COG1977: Molybdopterin converting...   
70   2e-11

gi|67156787|ref|ZP\_00418284.1|
ThiamineS [Azotobacter vinelandii...    70   2e-11

gi|15830372|ref|NP\_309145.1| putative tail assembly protein [Esc...    70  
2e-11

gi|15831241|ref|NP\_310014.1|
putative tail assembly protein [Esc...   
70   3e-11

gi|49180290|gb|AAT55666.1|
molybdopterin converting factor, subu...   
70   3e-11

gi|92118611|ref|YP\_578340.1|
molybdopterin converting factor, su...   
70   3e-11

gi|89056408|ref|YP\_511859.1|
molybdopterin converting factor, su...   
70   3e-11

gi|15831490|ref|NP\_310263.1|
putative tail assembly protein [Esc...   
70   3e-11

gi|51857712|dbj|BAD41870.1|
molybdopterin converting factor smal...   
70   3e-11

gi|72119091|gb|AAZ61354.1|
Molybdopterin converting factor, subu...   
70   4e-11

gi|91788067|ref|YP\_549019.1|
thiamineS [Polaromonas sp. JS666] >...   
69   4e-11

gi|68563154|ref|ZP\_00602356.1|
ThiamineS [Rubrobacter xylanophil...   
69   4e-11

gi|49330814|gb|AAT61460.1|
molybdopterin converting factor, subu...   
69   4e-11

gi|26107858|gb|AAN80057.1|
Putative tail component of prophage [...   
69   4e-11

gi|47551893|gb|AAT32729.2|
molybdopterin converting factor, subu...   
69   4e-11

gi|83748716|ref|ZP\_00945732.1|
ThiS family protein [Ralstonia so...   
69   4e-11

gi|15830901|ref|NP\_309674.1|
tail assembly protein [Escherichia ...   
69   5e-11

gi|42782629|ref|NP\_979876.1|
molybdopterin converting factor, su...   
69   5e-11

gi|53802819|ref|YP\_115441.1|
molybdopterin converting factor, su...   
69   5e-11

gi|84713090|ref|ZP\_01020876.1|
Molybdopterin converting factor, ...   
69   5e-11

gi|94969084|ref|YP\_591132.1|
molybdopterin converting factor, su...   
69   5e-11

gi|17739525|gb|AAL42140.1|
molybdopterin converting factor small...   
69   5e-11

gi|106888351|ref|ZP\_01355551.1|
molybdopterin converting factor,...    69  
6e-11

gi|76791187|ref|ZP\_00773695.1|
ThiamineS [Pseudoalteromonas atla...   
69   6e-11

gi|88795121|ref|ZP\_01110822.1|
molybdopterin converting factor, ...   
69   6e-11

gi|75254904|ref|ZP\_00726869.1|
COG4723: Phage-related protein, t...   
69   6e-11

gi|27382583|ref|NP\_774112.1|
molybdopterin converting factor sma...   
69   7e-11

gi|47568924|ref|ZP\_00239616.1|
molybdopterin converting factor, ...   
69   8e-11

gi|87301504|ref|ZP\_01084344.1|
molydbenum cofactor biosynthesis ...   
69   8e-11

gi|83589575|ref|YP\_429584.1|
MoaD [Moorella thermoacetica ATCC 3...   
69   8e-11

gi|75762421|ref|ZP\_00742290.1|
Molybdopterin converting factor, ...   
68   9e-11

gi|29897221|gb|AAP10498.1|
Molybdopterin (MPT) converting factor...   
68   9e-11

gi|89201285|ref|ZP\_01180030.1|
Molybdopterin converting factor, ...   
68   9e-11

gi|2507066|sp|P45309|MOAD\_HAEIN
Molybdopterin-converting factor ...   
68   9e-11

gi|69951944|ref|ZP\_00639556.1|
Molybdopterin converting factor, ...   
68   1e-10

gi|71143753|gb|AAZ24226.1| molybdopterin
converting factor, subu...    68   1e-10

gi|74312870|ref|YP\_311289.1|
putative tail component of prophage...   
68   1e-10

gi|68058473|gb|AAX88726.1|
molybdopterin converting factor subun...   
68   1e-10

gi|69936172|ref|ZP\_00631032.1|
Molybdopterin converting factor, ...   
68   1e-10

gi|77381195|gb|ABA72708.1|
MoaD [Pseudomonas fluorescens PfO-1] ...   
68   1e-10

gi|74317044|ref|YP\_314784.1|
molybdopterin converting factor, su...   
68   1e-10

gi|42783907|ref|NP\_981154.1|
molybdopterin converting factor, su...   
68   1e-10

gi|10640334|emb|CAC12148.1|
MoaD (involved in molybdopterin synt...   
68   1e-10

gi|86608912|ref|YP\_477674.1|
molybdopterin converting factor, su...   
68   1e-10

gi|83594146|ref|YP\_427898.1|
Molybdopterin converting factor, su...   
68   1e-10

gi|42631902|ref|ZP\_00157440.1|
COG1977: Molybdopterin converting...   
68   1e-10

gi|35214941|dbj|BAC92307.1|
gsr4366 [Gloeobacter violaceus PCC 7...   
67   1e-10

gi|51974253|gb|AAU15803.1|
molybdopterin converting factor, subu...   
67   2e-10

gi|91770046|ref|ZP\_01271876.1|
Molybdopterin converting factor, ...   
67   2e-10

gi|54014565|dbj|BAD55935.1|
hypothetical protein [Nocardia farci...   
67   2e-10

gi|46108760|ref|XP\_381438.1|
hypothetical protein FG01262.1 [Gib...   
67   2e-10

gi|90419510|ref|ZP\_01227420.1|
molybdopterin converting factor s...   
67   2e-10

gi|28807086|dbj|BAC60356.1|
molybdenum cofactor biosynthesis pro...   
67   2e-10

gi|42629022|ref|ZP\_00154572.1|
COG1977: Molybdopterin converting...   
67   2e-10

gi|49330166|gb|AAT60812.1|
molybdopterin converting factor, subu...   
67   2e-10

gi|83816286|ref|YP\_446147.1|
ThiS family, putative [Salinibacter...   
67   2e-10

gi|70728421|ref|YP\_258170.1|
molybdenum cofactor biosynthesis pr...   
67   2e-10

gi|29898350|gb|AAP11623.1|
Molybdopterin (MPT) converting factor...   
67   2e-10

gi|91227353|ref|ZP\_01261742.1|
molybdenum cofactor biosynthesis ...   
67   3e-10

gi|89093577|ref|ZP\_01166525.1|
molybdopterin converting factor, ...   
67   3e-10

gi|75855800|ref|ZP\_00763441.1|
COG1977: Molybdopterin converting...   
67   3e-10

gi|86357115|ref|YP\_469007.1|
molybdopterin converting factor sub...   
67   3e-10

gi|86159912|ref|YP\_466697.1|
thiamineS [Anaeromyxobacter dehalog...   
67   3e-10

gi|84685238|ref|ZP\_01013137.1|
molybdopterin converting factor, ...   
66   3e-10

gi|89204215|ref|ZP\_01182793.1|
Molybdopterin converting factor, ...   
66   3e-10

gi|76801893|ref|YP\_326901.1|
probable molybdopterin converting f...   
66   4e-10

gi|78700359|ref|ZP\_00864816.1|
Rhodanese-like [Alkalilimnicola e...   
66   4e-10

gi|91783438|ref|YP\_558644.1|
Molybdopterin converting factor, su...   
66   4e-10

gi|88938982|ref|ZP\_01144434.1|
molybdopterin converting factor, ...   
66   5e-10

gi|18161603|gb|AAL64874.1| conserved
hypothetical protein [Pyrob...    66   5e-10

gi|86607092|ref|YP\_475855.1|
ThiS domain protein [Synechococcus ...   
66   5e-10

gi|47505417|gb|AAT34093.1|
molybdopterin converting factor, subu...   
66   5e-10

gi|13883002|gb|AAK47534.1|
molybdenum cofactor biosynthesis prot...   
65   6e-10

gi|12514222|gb|AAG55511.1|
putative tail component encoded by cr...   
65   6e-10

gi|83648509|ref|YP\_436944.1|
Molybdopterin converting factor, sm...   
65   6e-10

gi|56908910|dbj|BAD63437.1|
molybdopterin converting factor subu...   
65   6e-10

gi|89070177|ref|ZP\_01157505.1|
putative molybdopterin MPT conver...   
65   6e-10

gi|94984437|ref|YP\_603801.1|
molybdopterin converting factor, su...   
65   6e-10

gi|28631173|ref|NP\_789776.1|
molybdopterin synthase small subuni...   
65   6e-10

gi|71676256|ref|ZP\_00673998.1|
ThiamineS [Trichodesmium erythrae...   
65   6e-10

gi|88601825|ref|YP\_502003.1|
thiamineS [Methanospirillum hungate...   
65   6e-10

gi|88809412|ref|ZP\_01124920.1|
molydbenum cofactor biosynthesis ...   
65   7e-10

gi|90422422|ref|YP\_530792.1|
molybdopterin converting factor, su...   
65   7e-10

gi|4457226|gb|AAD21202.1|
MoaD [Rhodobacter capsulatus]               
65   7e-10

gi|91220879|ref|ZP\_01257071.1|
putative molybdopterin MPT conver...   
65   8e-10

gi|15607139|ref|NP\_213755.1|
Molybdopterin converting factor, sm...   
65   8e-10

gi|83952487|ref|ZP\_00961218.1|
molybdopterin converting factor, ...   
65   9e-10

gi|86609522|ref|YP\_478284.1|
ThiS domain protein [Synechococcus ...   
65   9e-10

gi|15074101|emb|CAC45748.1|
PROBABLE MOLYBDOPTERIN MPT CONVERTIN...   
65   1e-09

gi|47502579|gb|AAT31255.1|
molybdopterin converting factor, subu...   
65   1e-09

gi|104783150|ref|YP\_609648.1|
phage tail protein [Pseudomonas en...   
65   1e-09

gi|90416705|ref|ZP\_01224635.1|
molybdenum cofactor biosynthesis ...   
64   1e-09

gi|21112021|gb|AAM40299.1|
molybdopterin-converting factor chain...   
64   1e-09

gi|2633802|emb|CAB13304.1|
molybdopterin converting factor (subu...   
64   1e-09

gi|58001333|gb|AAW60227.1|
Bifunctional molybdenum cofactor bios...   
64   1e-09

gi|99078603|ref|YP\_611861.1|
molybdopterin converting factor, su...   
64   2e-09

gi|89901619|ref|YP\_524090.1|
thiamineS [Rhodoferax ferrireducens...   
64   2e-09

gi|84515715|ref|ZP\_01003076.1|
molybdopterin converting factor, ...   
64   2e-09

gi|83369898|ref|ZP\_00914747.1|
Molybdopterin converting factor, ...   
64   2e-09

gi|68140833|gb|EAM94128.1|
MoaD, archaeal [Ferroplasma acidarman...   
64   2e-09

gi|4262375|gb|AAD14600.1|
molybdopterin-synthase small subunit [...   
64   2e-09

gi|91797290|gb|ABE59429.1|
molybdenum cofactor biosynthesis prot...   
64   2e-09

gi|84716935|ref|ZP\_01023301.1|
hypothetical protein PnapDRAFT\_04...   
64   2e-09

gi|104783496|ref|YP\_609994.1|
molybdenum cofactor biosynthesis p...   
64   2e-09

gi|19918186|gb|AAM07434.1|
molybdopterin converting factor, subu...   
64   2e-09

gi|23126667|ref|ZP\_00108556.1|
COG1977: Molybdopterin converting...   
64   3e-09

gi|67925013|ref|ZP\_00518396.1|
ThiamineS [Crocosphaera watsonii ...   
64   3e-09

gi|70731087|ref|YP\_260828.1|
molybdopterin converting factor, su...   
64   3e-09

gi|47566905|ref|ZP\_00237623.1|
molybdopterin converting factor, ...   
63   3e-09

gi|83372471|ref|ZP\_00917251.1|
Molybdopterin converting factor, ...    63   3e-09

gi|77389071|gb|ABA80256.1|
putative molybdopterin MPT converting...   
63   3e-09

gi|90022254|ref|YP\_528081.1|
Helix-turn-helix, AraC type [Saccha...   
63   4e-09

gi|29895812|gb|AAP09094.1|
Molybdopterin (MPT) converting factor...   
63   4e-09

gi|82543715|ref|YP\_407662.1|
putative tail component [Shigella b...   
63   4e-09

gi|76803138|ref|YP\_331233.1|
probable molybdopterin converting f...   
63   4e-09

gi|75765030|ref|ZP\_00744342.1|
Molybdopterin converting factor, ...   
63   4e-09

gi|85705896|ref|ZP\_01036992.1|
putative molybdopterin MPT conver...   
63   4e-09

gi|48430776|gb|AAT43641.1|
molybdopterin (MPT) converting factor...   
63   5e-09

gi|21107237|gb|AAM35973.1|
molybdopterin-converting factor chain...   
62   5e-09

gi|51858004|dbj|BAD42162.1|
molybdopterin converting factor-like...   
62   5e-09

gi|89206760|ref|ZP\_01185313.1|
Molybdopterin converting factor, ...   
62   5e-09

gi|16410447|emb|CAC99123.1|
lmo1045 [Listeria monocytogenes] >gi...   
62   5e-09

gi|51858105|dbj|BAD42263.1|
molybdopterin converting factor smal...   
62   7e-09

gi|26107735|gb|AAN79934.1|
Putative tail assembly protein of cry...   
62   7e-09

gi|78046664|ref|YP\_362839.1|
molybdopterin-converting factor cha...   
62   7e-09

gi|93005538|ref|YP\_579975.1|
thiamineS [Psychrobacter cryohalole...   
62   8e-09

gi|15621527|dbj|BAB65522.1|
236aa long hypothetical molybdopteri...   
62   9e-09

gi|10581293|gb|AAG20053.1| Vng1848h [Halobacterium sp. NRC-1]
>g...    62   9e-09

gi|70606737|ref|YP\_255607.1| molybdenum cofactor biosynthesis pr...    62  
9e-09

gi|49333106|gb|AAT63752.1| molybdopterin biosynthesis protein, s...    62  
9e-09

gi|89359306|ref|ZP\_01197127.1| Molybdopterin converting factor, ...    62  
1e-08

gi|56680191|gb|AAV96857.1| molybdopterin converting factor, subu...    62  
1e-08

gi|17431817|emb|CAD18495.1| conserved hypothetical protein [Rals...    62  
1e-08

gi|47096132|ref|ZP\_00233732.1| molybdenum cofactor biosynthesis ...    61  
1e-08

gi|46907277|ref|YP\_013666.1| molybdenum cofactor biosynthesis pr...    61  
1e-08

gi|83941421|ref|ZP\_00953883.1| putative molybdopterin MPT conver...    61  
1e-08

gi|51976766|gb|AAU18316.1| molybdopterin biosynthesis protein, s...    61  
1e-08

gi|40063023|gb|AAR37879.1| molybdenum cofactor biosynthesis prot...    61  
1e-08

gi|84354863|ref|ZP\_00979760.1| COG1977: Molybdopterin converting...    61  
1e-08

gi|6460436|gb|AAF12145.1| molybdenum cofactor biosynthesis prote...    61  
1e-08

gi|56379152|dbj|BAD75060.1| molybdopterin converting factor (sub...    61  
1e-08

gi|47573807|ref|ZP\_00243844.1| COG1977: Molybdopterin converting...    61  
2e-08

gi|15645420|ref|NP\_207594.1| molybdopterin converting factor, su...    61  
2e-08

gi|33867003|ref|NP\_898562.1| molydbenum cofactor biosynthesis pr...    61  
2e-08

gi|75701250|gb|ABA20926.1| ThiamineS [Anabaena variabilis ATCC 2...    61  
2e-08

gi|16413496|emb|CAC96268.1| lin1037 [Listeria innocua]
>gi|16800...    61   2e-08

gi|74018017|ref|ZP\_00688639.1| ThiamineS [Burkholderia ambifaria...    61  
2e-08

gi|46201345|ref|ZP\_00055205.2| COG1977: Molybdopterin converting...    60  
2e-08

gi|18160982|gb|AAL64296.1| conserved hypothetical protein [Pyrob...    60  
2e-08

gi|10175640|dbj|BAB06737.1| molybdopterin converting factor (sub...    60  
2e-08

gi|66811416|ref|XP\_639888.1| hypothetical protein DDB0186363 [Di...    60  
2e-08

gi|83854898|ref|ZP\_00948428.1|
molybdopterin converting factor, ...   
60   2e-08

gi|17133489|dbj|BAB76052.1|
asl4353 [Nostoc sp. PCC 7120] >gi|17...   
60   2e-08

gi|68562526|ref|ZP\_00601781.1|
Molybdopterin converting factor, ...   
60   2e-08

gi|11877308|emb|CAC19022.1|
putative protein I [Neisseria mening...   
60   2e-08

gi|77964628|gb|ABB06009.1|
Thiamine S [Burkholderia sp. 383] >gi...   
60   2e-08

gi|84499780|ref|ZP\_00998068.1|
putative molybdopterin MPT conver...   
60   2e-08

gi|13883249|gb|AAK47766.1|
molybdopterin cofactor biosynthesis p...   
60   3e-08

gi|58580685|ref|YP\_199701.1|
molybdopterin-converting factor cha...   
60   3e-08

gi|40062746|gb|AAR37640.1|
molybdopterin converting factor, subu...   
60   3e-08

gi|89100759|ref|ZP\_01173613.1|
molybdopterin converting factor, ...   
60   3e-08

gi|16331655|ref|NP\_442383.1|
hypothetical protein ssr1527 [Synec...   
60   3e-08

gi|6226383|sp|O29699|Y552\_ARCFU
Hypothetical UPF0084 protein AF0...   
60   3e-08

gi|78170141|gb|ABB27238.1|
molydbenum cofactor biosynthesis prot...   
60   4e-08

gi|30681325|ref|NP\_849354.1|
CNX7/SIR5; catalytic [Arabidopsis t...   
60   4e-08

gi|28271030|emb|CAD63935.1|
molybdopterin biosynthesis protein, ...   
60   4e-08

gi|71038167|gb|AAZ18475.1|
probable molybdopterin converting fac...   
60   4e-08

gi|24050968|gb|AAN42352.1| putative tail component [Shigella fle...    59  
5e-08

gi|88801656|ref|ZP\_01117184.1| putative molybdopterin-synthase s...    59  
5e-08

gi|23395272|gb|AAN31771.1| molybdopterin converting factor, smal...    59  
5e-08

gi|17431786|emb|CAD18464.1| conserved hypothetical protein [Rals...    59  
6e-08

gi|67904312|ref|XP\_682412.1| hypothetical protein AN9143.2 [Aspe...    59  
6e-08

gi|86139074|ref|ZP\_01057645.1| molybdopterin converting factor, ...    59  
7e-08

gi|84705083|ref|ZP\_01018583.1| molybdopterin converting factor, ...    59  
7e-08

gi|92089022|ref|ZP\_01273976.1| ThiamineS [Lactobacillus reuteri ...    59  
8e-08

gi|68193704|gb|EAN08356.1| Molybdopterin converting factor, subu...    59  
8e-08

gi|46156355|ref|ZP\_00204683.1| COG1977: Molybdopterin converting...    59  
8e-08

gi|85373384|ref|YP\_457446.1| hypothetical protein ELI\_02785 [Ery...    59  
8e-08

gi|39964901|ref|XP\_365048.1| hypothetical protein MG09893.4 [Mag...    59  
8e-08

gi|57638416|gb|AAW55204.1| molybdopterin converting factor, subu...    59  
8e-08

gi|45552983|ref|NP\_996018.1| CG33276-PA [Drosophila melanogaster...    58  
9e-08

gi|21959032|gb|AAM85750.1| putative phage tail protein [Yersinia...    58  
9e-08

gi|56966838|pdb|1WGK|A
Chain A, Solution Structure Of Mouse Hypo...   
58   1e-07

gi|52003241|gb|AAU23183.1|
molybdopterin converting factor (subu...   
58   1e-07

gi|39982776|gb|AAR34235.1| moaD
family protein [Geobacter sulfur...   
58   1e-07

gi|94313559|ref|YP\_586768.1|
thiamineS [Ralstonia metallidurans ...   
58   1e-07

gi|70982492|ref|XP\_746774.1|
molybdopterin synthase small subuni...   
58   1e-07

gi|66363058|ref|XP\_628495.1|
hypothetical protein cgd7\_3310 [Cry...   
58   1e-07

gi|75239568|ref|ZP\_00723538.1|
COG4723: Phage-related protein, t...    58   1e-07

gi|71037999|gb|AAZ18307.1| probable phage protein tail protein [...    58  
1e-07

gi|86739160|ref|YP\_479560.1|
thiamine S [Frankia sp. CcI3] >gi|8...   
58   1e-07

gi|8670895|emb|CAB94946.1|
SPCC548.04 [Schizosaccharomyces pombe...   
58   1e-07

gi|98961773|gb|ABF59216.1|
unknown protein [Arabidopsis thaliana...   
57   2e-07

gi|67623933|ref|XP\_668249.1|
chromosome 9 open reading frame 74 ...   
57   2e-07

gi|75175531|ref|ZP\_00695747.1|
COG4723: Phage-related protein, t...   
57   2e-07

gi|83026442|gb|ABB96257.1|
VP15 [Zea mays]                            
57   2e-07

gi|72083928|ref|XP\_789250.1|
PREDICTED: similar to CG33276-PA [S...   
57   2e-07

gi|75255278|ref|ZP\_00727116.1|
COG4723: Phage-related protein, t...   
57   2e-07

gi|88805648|ref|ZP\_01121167.1|
hypothetical protein RB2501\_14949...   
57   2e-07

gi|82533244|ref|ZP\_00892340.1|
hypothetical protein Bpse110\_0200...   
57   2e-07

gi|56554614|pdb|1XO3|A
Chain A, Solution Structure Of Ubiquitin ...   
57   2e-07

gi|68446507|dbj|BAE04091.1|
moaD [Staphylococcus haemolyticus JC...   
57   2e-07

gi|75214996|ref|ZP\_00713461.1|
COG4723: Phage-related protein, t...   
57   2e-07

gi|75259495|ref|ZP\_00730814.1|
COG4723: Phage-related protein, t...   
57   2e-07

gi|88603453|ref|YP\_503631.1|
thiamineS [Methanospirillum hungate...   
57   2e-07

gi|95102780|gb|ABF51331.1|
ubiquitin related modifier [Bombyx mori]   
57   2e-07

gi|67664217|ref|ZP\_00461493.1|
ThiamineS [Burkholderia cenocepac...   
57   3e-07

gi|73661952|ref|YP\_300733.1|
molybdopterin converting factor sma...   
57   3e-07

gi|6968941|emb|CAB73937.1|
possible molybdopterin converting fac...   
57   3e-07

gi|57167344|gb|AAW36123.1|
thiS family protein [Campylobacter je...   
57   3e-07

gi|75239817|ref|ZP\_00723778.1| COG4723: Phage-related protein, t...    57  
3e-07

gi|87309615|ref|ZP\_01091749.1| hypothetical protein DSM3645\_0234...    57  
3e-07

gi|21311875|ref|NP\_080891.1| hypothetical protein LOC68205 [Mus ...    57  
3e-07

gi|26339916|dbj|BAC33621.1| unnamed protein product [Mus musculus]     57  
3e-07

gi|56314521|emb|CAI09166.1| putative tungsten-containing aldehyd...    56  
3e-07

gi|78700231|ref|ZP\_00864688.1| hypothetical protein MlgDRAFT\_272...    56  
3e-07

gi|55632749|ref|XP\_520407.1| PREDICTED: similar to chromosome 9 ...    56   4e-07

gi|37787961|gb|AAP49965.1| putative tail assembly protein [Enter...    56  
4e-07

gi|47571833|ref|ZP\_00241881.1| hypothetical protein Rgel02003817...    56  
4e-07

gi|67483722|ref|XP\_657081.1| hypothetical protein 4.t00118 [Enta...    56  
4e-07

gi|75215134|ref|ZP\_00713582.1| COG4723: Phage-related protein, t...    56  
4e-07

gi|14324783|dbj|BAB59710.1| molybdopterin converting factor subu...    56  
4e-07

gi|75233804|ref|ZP\_00718281.1| COG4723: Phage-related protein, t...    56  
4e-07

gi|67154931|ref|ZP\_00416676.1| ThiamineS [Azotobacter vinelandii...    56  
4e-07

gi|75259293|ref|ZP\_00730641.1| COG4723: Phage-related protein, t...    56  
4e-07

gi|75207839|ref|ZP\_00708320.1| COG4723: Phage-related protein, t...    56  
5e-07

gi|88931544|ref|ZP\_01137238.1| putative molybdopterin converting...    56  
5e-07

gi|75257430|ref|ZP\_00728933.1| COG4723: Phage-related protein, t...    56  
5e-07

gi|75258709|ref|ZP\_00730116.1| COG4723: Phage-related protein, t...    56  
5e-07

gi|75210818|ref|ZP\_00710950.1| COG4723: Phage-related protein, t...    56  
5e-07

gi|47572157|ref|ZP\_00242203.1| COG1977: Molybdopterin converting...    56   5e-07

gi|74018319|ref|ZP\_00688940.1|
conserved hypothetical protein [B...   
56   6e-07

gi|68367092|ref|XP\_686397.1|
PREDICTED: similar to CG33276-PA, p...   
56   6e-07

gi|75255450|ref|ZP\_00727253.1|
COG4723: Phage-related protein, t...    56  
6e-07

gi|75208698|ref|ZP\_00708992.1|
COG4723: Phage-related protein, t...   
55   6e-07

gi|17428712|emb|CAD15398.1|
probable phage hk022 gp20-related pr...   
55   6e-07

gi|34483282|emb|CAE10280.1|
hypothetical protein [Wolinella succ...   
55   6e-07

gi|75820383|ref|ZP\_00750432.1|
COG4723: Phage-related protein, t...   
55   6e-07

gi|32444387|emb|CAD74386.1|
probable molybdopterin converting fa...   
55   6e-07

gi|82751858|ref|YP\_417599.1|
molybdopterin converting factor sma...   
55   6e-07

gi|90303795|gb|EAS33426.1|
hypothetical protein CIMG\_04450 [Cocc...   
55   7e-07

gi|68129461|emb|CAJ08004.1| ubiquitin related modifier (urm1)-li...    55  
7e-07

gi|88186128|gb|EAQ93596.1| hypothetical protein CHGG\_01831 [Chae...    55  
7e-07

gi|89361078|ref|ZP\_01198894.1| conserved hypothetical protein [X...    55  
7e-07

gi|75238944|ref|ZP\_00722926.1| COG4723: Phage-related protein, t...    55   7e-07

gi|73967924|ref|XP\_850726.1|
PREDICTED: similar to CG33276-PA is...   
55   7e-07

gi|88703397|ref|ZP\_01101113.1|
Molybdopterin converting factor s...   
55   7e-07

gi|83717443|ref|YP\_439258.1|
Bacteriophage lambda tail assembly ...   
55   7e-07

gi|71416009|ref|XP\_810051.1|
hypothetical protein [Trypanosoma c...   
55   7e-07

gi|76579036|gb|ABA48511.1| Bacteriophage lambda tail assembly pr...    55  
7e-07

gi|76630667|ref|XP\_611592.2|
PREDICTED: similar to CG33276-PA is...   
55   7e-07

gi|83587164|ref|ZP\_00925793.1|
COG4723: Phage-related protein, t...   
55   8e-07

gi|85059620|ref|YP\_455322.1|
putative phage tail assembly protei...   
55   8e-07

gi|75235846|ref|ZP\_00720001.1|
COG4723: Phage-related protein, t...   
55   9e-07

gi|13421105|gb|AAK22005.1|
molybdopterin converting factor, subu...   
55   9e-07

gi|53133340|emb|CAG31999.1|
hypothetical protein [Gallus gallus]...   
55   9e-07

gi|89902161|ref|YP\_524632.1|
thiamineS [Rhodoferax ferrireducens...   
55   9e-07

gi|85707987|ref|ZP\_01039053.1|
hypothetical protein NAP1\_02090 [...   
55   9e-07

gi|27706604|ref|XP\_231116.1|
PREDICTED: similar to RIKEN cDNA 29...   
55   9e-07

gi|72163135|ref|YP\_290792.1|
putative molybdopterin converting f...   
55   1e-06

gi|75235151|ref|ZP\_00719393.1|
COG4723: Phage-related protein, t...   
55   1e-06

gi|13097768|gb|AAH03581.1|
Chromosome 9 open reading frame 74 [H...   
55   1e-06

gi|76795290|ref|ZP\_00777663.1|
ThiamineS [Thermoanaerobacter eth...   
54   1e-06

gi|88712833|ref|ZP\_01106918.1|
hypothetical protein FB2170\_09351...   
54   1e-06

gi|86153122|ref|ZP\_01071327.1|
conserved domain protein [Campylo...    54  
1e-06

gi|86149491|ref|ZP\_01067722.1|
thiS family protein [Campylobacte...   
54   1e-06

gi|56383531|gb|AAN43440.2|
putative tail component [Shigella fle...   
54   1e-06

gi|66963966|ref|ZP\_00411537.1|
ThiamineS [Arthrobacter sp. FB24]...   
54   1e-06

gi|66565892|ref|XP\_623975.1|
PREDICTED: hypothetical protein XP\_...   
54   1e-06

gi|55232699|gb|AAV48118.1|
unknown [Haloarcula marismortui ATCC ...   
54   1e-06

gi|75234649|ref|ZP\_00718969.1|
COG4723: Phage-related protein, t...   
54   1e-06

gi|83312024|ref|YP\_422288.1|
hypothetical protein amb2925 [Magne...   
54   1e-06

gi|107022979|ref|YP\_621306.1|
thiamineS [Burkholderia cenocepaci...   
54   1e-06

gi|74312266|ref|YP\_310685.1|
putative tail component of prophage...   
54   1e-06

gi|88196185|ref|YP\_501002.1|
molybdopterin converting factor, su...   
54   1e-06

gi|84352786|ref|ZP\_00977734.1|
hypothetical protein BcenP\_010050...   
54   2e-06

gi|68553535|ref|ZP\_00592907.1|
ThiamineS [Prosthecochloris aestu...   
54   2e-06

gi|88931571|ref|ZP\_01137265.1|
Ferredoxin--nitrite reductase [Ac...   
54   2e-06

gi|75229909|ref|ZP\_00716426.1|
COG4723: Phage-related protein, t...   
54   2e-06

gi|75208766|ref|ZP\_00709055.1|
COG4723: Phage-related protein, t...   
54   2e-06

gi|50936971|ref|XP\_478013.1|
putative ubiquitin-related modifier...   
54   2e-06

gi|4155307|gb|AAD06323.1|
putative MOLYBDOPTERIN CONVERTING FACT...   
54   2e-06

gi|70731105|ref|YP\_260846.1|
prophage LambdaSo, tail assembly pr...   
54   2e-06

gi|11499688|ref|NP\_070930.1|
hypothetical protein AF2105 [Archae...   
54   2e-06

gi|21637142|gb|AAM70358.1|
CalU18 [Micromonospora echinospora]        
54   2e-06

gi|59410400|gb|AAW84299.1|
putative MaoD-like protein [unculture...   
54   2e-06

gi|75239670|ref|ZP\_00723637.1|
COG4723: Phage-related protein, t...   
54   2e-06

gi|19173038|ref|NP\_597589.1|
hypothetical protein ECU03\_0800 [En...   
54   2e-06

gi|86143258|ref|ZP\_01061660.1|
hypothetical protein MED217\_08750...   
54   2e-06

gi|83646801|ref|YP\_435236.1|
ThiS family protein [Hahella chejue...   
54   2e-06

gi|71652006|ref|XP\_814668.1|
hypothetical protein [Trypanosoma c...   
54   2e-06

gi|6322183|ref|NP\_012258.1|
Ubiquitin-like protein with only wea...   
54   2e-06

gi|50285825|ref|XP\_445341.1|
unnamed protein product [Candida gl...    54  
2e-06

gi|32261593|gb|AAP76643.1|
molybdopterin converting factor [Heli...   
54   2e-06

gi|49484485|ref|YP\_041709.1|
putative molybdopterin-synthase sma...   
54   3e-06

gi|75211970|ref|ZP\_00712025.1|
COG4723: Phage-related protein, t...   
54   3e-06

gi|60688583|gb|AAX30383.1|
SJCHGC03168 protein [Schistosoma japo...   
53   3e-06

gi|38505401|gb|AAR23170.1|
gp19 [Bacteriophage phi1026b] >gi|387...   
53   3e-06

gi|81343970|ref|YP\_398986.1|
putative tail assembly protein [Bac...   
53   3e-06

gi|78496994|gb|ABB43534.1|
Thiamine S [Thiomicrospira denitrific...   
53   3e-06

gi|62425453|ref|ZP\_00380587.1|
COG1977: Molybdopterin converting...    53   3e-06

gi|24374467|ref|NP\_718510.1| prophage LambdaSo, tail assembly pr...    53  
3e-06

gi|40218250|gb|AAR83036.1| Gp20 [Bacteriophage phiKO2]
>gi|46402...    53   3e-06

gi|17484042|gb|AAL40293.1| gp20 [Bacteriophage phiE125]
>gi|1797...    53   4e-06

gi|13815697|gb|AAK42541.1| Molybdenum cofactor biosynthesis prot...    53  
4e-06

gi|86134373|ref|ZP\_01052955.1| putative molybdopterin-synthase s...    53  
4e-06

gi|85117520|ref|XP\_965278.1| hypothetical protein [Neurospora cr...    53  
4e-06

gi|83773325|dbj|BAE63452.1| unnamed protein product [Aspergillus...    52  
5e-06

gi|86604898|ref|YP\_473661.1| putative molybdopterin converting f...    52  
5e-06

gi|84328291|ref|ZP\_00976298.1| COG1977: Molybdopterin converting...    52  
7e-06

gi|51588192|emb|CAH19799.1| hypothetical protein [Yersinia pseud...    52  
7e-06

gi|67663026|ref|ZP\_00460311.1| conserved hypothetical protein [B...    52  
7e-06

gi|76885874|gb|ABA60064.1| gp63 [Burkholderia cepacia phage Bcep...    52  
7e-06

gi|57505249|ref|ZP\_00371178.1| moaD2 [Campylobacter upsaliensis ...    52  
7e-06

gi|86158644|ref|YP\_465429.1| thiamineS [Anaeromyxobacter dehalog...    52  
7e-06

gi|71149516|gb|AAZ29254.1| JK\_4P [Bacteriophage JK06]
>gi|718340...    52   7e-06

gi|22295085|dbj|BAC08913.1| molybdopterin biosynthesis protein D...    52  
8e-06

gi|14524271|gb|AAK65772.1| Hypothetical protein SMa2034 [Sinorhi...    52  
8e-06

gi|3955207|gb|AAC83142.1| MoaD [Staphylococcus carnosus]               52   8e-06

gi|86154746|ref|ZP\_01072913.1|
conserved domain protein [Campylo...   
52   8e-06

gi|83951776|ref|ZP\_00960508.1|
hypothetical protein ISM\_14475 [R...    52  
9e-06

gi|21624374|dbj|BAC01162.1|
ubiquitin-related modifier-1 [Oryza ...   
52   9e-06

gi|66810572|ref|XP\_638993.1|
hypothetical protein DDB0185658 [Di...   
52   9e-06

gi|33565867|emb|CAE36414.1|
molybdopterin converting factor [Bor...   
52   1e-05

gi|55238641|gb|EAA11560.2|
ENSANGP00000017452 [Anopheles gambiae...   
52   1e-05

gi|90591660|ref|ZP\_01247301.1|
ThiamineS [Flavobacterium johnson...   
52   1e-05

gi|67543327|ref|ZP\_00421260.1|
conserved hypothetical protein [B...   
51   1e-05

gi|70606476|ref|YP\_255346.1|
hypothetical protein Saci\_0669 [Sul...   
51   1e-05

gi|55240673|gb|EAL40503.1|
ENSANGP00000025406 [Anopheles gambiae...   
51   1e-05

gi|50909475|ref|XP\_466226.1| unknown protein [Oryza sativa (japo...    51  
1e-05

gi|107025560|ref|YP\_623071.1| hypothetical protein Bcen\_3203 [Bu...    51  
1e-05

gi|90307635|gb|EAS37266.1| hypothetical protein CIMG\_02620 [Cocc...    51  
2e-05

gi|18160535|gb|AAL63881.1| conserved hypothetical protein [Pyrob...    51  
2e-05

gi|33563747|emb|CAE42987.1| molybdopterin converting factor [Bor...    50  
2e-05

gi|77918070|ref|YP\_355885.1| molybdopterin converting factor, su...    50  
2e-05

gi|71022553|ref|XP\_761506.1| hypothetical protein UM05359.1 [Ust...    50  
2e-05

gi|72122148|gb|AAZ64334.1| ThiamineS [Ralstonia eutropha JMP134]...    50  
2e-05

gi|68492387|ref|XP\_710043.1| hypothetical protein CaO19.1016 [Ca...    50  
2e-05

gi|14424008|sp|O30213|Y022\_ARCFU Hypothetical protein AF0022
>gi...    50   2e-05

gi|33567914|emb|CAE31827.1| molybdopterin converting factor [Bor...    50  
2e-05

gi|44982644|gb|AAS51907.1| ADL014Wp [Ashbya gossypii ATCC 10895]...    50  
2e-05

gi|67927210|ref|ZP\_00520404.1| Molybdopterin biosynthesis MoaE:T...    50  
3e-05

gi|84359228|ref|ZP\_00983978.1| COG1977: Molybdopterin converting...    50   3e-05

gi|83716552|ref|YP\_439903.1|
ThiS family domain protein, putativ...   
50   3e-05

gi|78702657|ref|ZP\_00867087.1|
conserved hypothetical protein [A...   
50   3e-05

gi|77996211|gb|ABB15110.1|
molybdopterin converting factor, subu...   
50   3e-05

gi|82741527|ref|ZP\_00904246.1|
prophage LambdaSo, tail assembly ...   
50   3e-05

gi|21222559|ref|NP\_628338.1|
molybdopterin converting factor [St...   
50   3e-05

gi|76802608|ref|YP\_327616.1|
homolog to molybdopterin converting...   
50   3e-05

gi|71366157|ref|ZP\_00656703.1|
Ferredoxin--nitrite reductase [No...   
50   4e-05

gi|29830583|ref|NP\_825217.1|
molybdopterin converting factor [St...   
50   4e-05

gi|67937987|ref|ZP\_00530517.1|
ThiamineS [Chlorobium phaeobacter...    49  
6e-05

gi|71031556|ref|XP\_765420.1|
hypothetical protein TP02\_0852 [The...   
49   6e-05

gi|89286674|gb|EAR84671.1|
RIKEN cDNA 2900073H19-related [Tetrah...   
49   6e-05

gi|34482542|emb|CAE09542.1|
hypothetical protein [Wolinella succ...   
49   7e-05

gi|32043835|ref|ZP\_00141097.1|
COG4723: Phage-related protein, t...   
49   8e-05

gi|17554136|ref|NP\_498102.1|
K10D2.7 [Caenorhabditis elegans] >g...   
49   8e-05

gi|9946516|gb|AAG04029.1|
probable bacteriophage protein [Pseudo...   
49   9e-05

gi|84318835|ref|ZP\_00967252.1|
COG4723: Phage-related protein, t...   
48   9e-05

gi|50307433|ref|XP\_453695.1|
unnamed protein product [Kluyveromy...   
48   1e-04

gi|32405808|ref|XP\_323517.1|
related to ubiquitin related modifi...   
48   1e-04

gi|87198948|ref|YP\_496205.1|
thiamineS [Novosphingobium aromatic...   
48   1e-04

gi|82743846|ref|ZP\_00906465.1|
prophage LambdaSo, tail assembly ...   
48   1e-04

gi|85702836|ref|ZP\_01033940.1|
hypothetical protein ROS217\_18882...   
48   1e-04

gi|57240883|ref|ZP\_00368831.1|
molybdopterin converting factor, ...   
48   1e-04

gi|50839587|gb|AAT82254.1|
putative molybdopterin converting fac...   
48   1e-04

gi|71010320|ref|XP\_758375.1|
hypothetical protein UM02228.1 [Ust...   
48   1e-04

gi|75760851|ref|ZP\_00740866.1|
Molybdopterin converting factor, ...   
48   1e-04

gi|9630484|ref|NP\_046915.1|
gp20 [Bacteriophage N15] >gi|3192703...   
48   1e-04

gi|63256717|gb|AAY37813.1|
Bacteriophage lambda tail assembly I ...   
48   1e-04

gi|76801420|ref|YP\_326428.1|
pterin cluster protein [Natronomona...   
48   1e-04

gi|83771659|dbj|BAE61789.1|
unnamed protein product [Aspergillus...   
47   2e-04

gi|87121811|ref|ZP\_01077697.1|
molybdenum cofactor biosynthesis ...   
47   2e-04

gi|88946143|ref|ZP\_01149231.1|
conserved hypothetical protein [D...   
47   2e-04

gi|68484140|ref|XP\_713962.1|
hypothetical protein CaO19.9835 [Ca...   
47   2e-04

gi|54017794|dbj|BAD59164.1|
hypothetical protein [Nocardia farci...   
47   2e-04

gi|55231495|gb|AAV46914.1|
unknown [Haloarcula marismortui ATCC ...   
47   3e-04

gi|77997120|gb|ABB16019.1|
conserved hypothetical protein [Carbo...   
47   3e-04

gi|98660190|dbj|GAA02501.1|
unnamed protein product [Pelotomacul...   
47   3e-04

gi|25169121|emb|CAD47957.1|
putative molybdopterin cofactor synt...  
 47   3e-04

gi|88935576|ref|ZP\_01141206.1|
similar to molybdopterin converti...   
46   4e-04

gi|51773733|emb|CAH23258.1|
putative tail fiber component I [Bac...   
46   5e-04

gi|87311482|ref|ZP\_01093601.1|
hypothetical protein DSM3645\_0176...   
46   5e-04

gi|13813427|gb|AAK40623.1|
Hypothetical protein SSO5559 [Sulfolo...   
46   5e-04

gi|98659923|dbj|GAA02748.1|
unnamed protein product [Pelotomacul...   
46   5e-04

gi|46125923|ref|XP\_387515.1|
hypothetical protein FG07339.1 [Gib...   
46   5e-04

gi|71492602|gb|EAO24906.1|
ThiamineS [Syntrophomonas wolfei subs...   
46   6e-04

gi|71546139|ref|ZP\_00666991.1|
conserved hypothetical protein [S...   
45   6e-04

gi|86740565|ref|YP\_480965.1|
cytochrome P450 [Frankia sp. CcI3] ...   
45   6e-04

gi|42557747|emb|CAF28721.1|
putative molybdopterin biosynthesis ...   
45   7e-04

gi|77917869|ref|YP\_355684.1|
hypothetical protein Pcar\_0253 [Pel...   
45   9e-04

gi|41406904|ref|NP\_959740.1|
MoaD2 [Mycobacterium avium subsp. p...   
45   0.001

gi|56680881|gb|AAV97546.1|
conserved hypothetical protein [Silic...   
45   0.001

gi|95929924|ref|ZP\_01312664.1|
thiamineS [Desulfuromonas acetoxi...   
45   0.001

gi|88934592|ref|ZP\_01140234.1|
conserved hypothetical protein [G...   
45   0.001

gi|90201062|ref|ZP\_01203708.1|
ThiamineS [Mycobacterium vanbaale...   
44   0.001

gi|45047786|emb|CAF30913.1|
ThiamineS [Methanococcus maripaludis...   
44   0.001

gi|84994270|ref|XP\_951857.1|
hypothetical protein TA15510 [Theil...   
44   0.002

gi|90021102|ref|YP\_526929.1|
hypothetical protein Sde\_1455 [Sacc...   
44   0.002

gi|50417028|ref|XP\_457622.1|
hypothetical protein DEHA0B15631g [...   
44   0.002

gi|23492025|dbj|BAC17000.1|
putative molybdopterin biosynthesis ...   
44   0.002

gi|21322979|dbj|BAB97608.1|
Hypothetical protein [Corynebacteriu...   
44   0.002

gi|82546334|ref|YP\_410281.1|
Sulfur transfer protein [Shigella b...   
44   0.003

gi|71838567|ref|ZP\_00678326.1|
ThiamineS [Pelobacter propionicus...   
44   0.003

gi|13880442|gb|AAK45132.1|
conserved hypothetical protein [Mycob...   
44   0.003

gi|47571794|ref|ZP\_00241843.1|
COG2104: Sulfur transfer protein ...   
44   0.003

gi|84498430|ref|ZP\_00997200.1|
putative molybdopterin converting...   
44   0.003

gi|82778833|ref|YP\_405182.1|
Sulfur transfer protein involved in...    43  
0.003

gi|71544347|ref|ZP\_00665385.1|
ThiamineS [Syntrophobacter fumaro...   
43   0.003

gi|90581019|ref|ZP\_01236819.1|
sulfur carrier protein ThiS [Vibr...   
43   0.004

gi|91785395|ref|YP\_560601.1|
Putative sulfur transfer protein in...   
43   0.004

gi|77919319|ref|YP\_357134.1|
molybdopterin converting factor, su...   
43   0.005

gi|51856879|dbj|BAD41037.1|
conserved hypothetical protein [Symb...   
42   0.006

gi|46449006|gb|AAS95658.1|
hypothetical protein DVU\_1180 [Desulf...   
42   0.007

gi|41324446|emb|CAF18786.1|
PUTATIVE MOLYBDOPTERIN CONVERTING FA...   
42   0.008

gi|12518920|gb|AAG59188.1|
thiamin biosynthesis, probable sulfur...   
42   0.009

gi|89343138|ref|ZP\_01195366.1|
ThiamineS [Mycobacterium flavesce...   
42   0.009

gi|95930902|ref|ZP\_01313632.1|
thiamineS [Desulfuromonas acetoxi...   
42   0.009

gi|63255028|gb|AAY36124.1|
Molybdopterin converting factor, subu...   
74   2e-12

gi|86748300|ref|YP\_484796.1|
molybdopterin converting factor, su...   
73   4e-12

gi|16765911|ref|NP\_461526.1|
tail assembly protein I-like [Salmo...   
68   1e-10

gi|77382376|gb|ABA73889.1|
ThiamineS [Pseudomonas fluorescens Pf...   
62   6e-09

gi|9634177|ref|NP\_037714.1|
Gp21 [Bacteriophage HK97] >gi|963413...   
49   9e-05

gi|15830813|ref|NP\_309586.1| putative tail assembly protein [Esc...    47  
3e-04

gi|736275|emb|CAA56171.1| unnamed protein product [Pyrococcus fu...    43  
0.003

gi|57230153|gb|AAW46554.1| conserved hypothetical protein [Crypt...    43  
0.005

gi|39943934|ref|XP\_361504.1| hypothetical protein MG03978.4 [Mag...    42   0.007

gi|12516097|gb|AAG56996.1|
putative tail fiber component I of pr...   
42   0.007

gi|62179803|ref|YP\_216220.1|
Gifsy-1 prophage VtiI [Salmonella e...   
42   0.008

gi|74314485|ref|YP\_312904.1|
Sulfur transfer protein [Shigella s...   
42   0.011

gi|75255488|ref|ZP\_00727287.1|
COG2104: Sulfur transfer protein ...   
41   0.012

gi|91212802|ref|YP\_542788.1|
hypothetical protein UTI89\_C3828 [E...   
41   0.012

gi|9947297|gb|AAG04744.1|
hypothetical protein PA1355 [Pseudomon...   
41   0.012

gi|49077442|gb|AAT49679.1|
PA1355 [synthetic construct]               
41   0.013

gi|38704219|ref|NP\_944578.1|
sulfur carrier protein ThiS [Escher...   
41   0.013

gi|48994994|gb|AAT48237.1|
sulphur carrier protein [Escherichia ...   
41   0.015

gi|89074422|ref|ZP\_01160899.1|
sulfur carrier protein ThiS [Phot...   
41   0.015

gi|14025577|dbj|BAB52177.1|
thiamin biosynthesis; ThiG [Mesorhiz...   
40   0.020

gi|86609832|ref|YP\_478594.1|
thiamine biosynthesis protein ThiS ...    40  
0.025

gi|88811647|ref|ZP\_01126901.1|
hypothetical protein NB231\_04560 ...   
40   0.029

gi|7227333|gb|AAF42389.1|
thiamine biosynthesis protein ThiS [Ne...   
40   0.030

gi|71080598|ref|XP\_779378.1|
hypothetical protein GLP\_165\_47231\_...   
40   0.032

gi|9654459|gb|AAF93242.1|
thiS protein [Vibrio cholerae O1 biova...   
40   0.035

gi|76874357|emb|CAI85578.1|
putative ThiS protein [Pseudoalterom...   
40   0.035

gi|36783916|emb|CAE12778.1|
ThiS protein [Photorhabdus luminesce...   
40   0.037

gi|52427701|gb|AAU48294.1|
thiamine biosynthesis protein ThiS, p...   
40   0.038

gi|78193517|gb|ABB31284.1|
Thiamine S [Geobacter metallireducens...   
40   0.041

gi|106882327|ref|ZP\_01349730.1|
thiamine biosynthesis protein Th...   
39   0.044

gi|74318466|ref|YP\_316206.1|
hypothetical protein Tbd\_2448 [Thio...   
39   0.046

gi|89901242|ref|YP\_523713.1|
thiamine biosynthesis protein ThiS ...   
39   0.047

gi|78219906|gb|ABB39255.1|
conserved hypothetical protein [Desul...   
39   0.047

gi|83720274|ref|YP\_443511.1|
thiamine biosynthesis protein ThiS ...   
39   0.052

gi|86605752|ref|YP\_474515.1|
thiamine biosynthesis protein ThiS ...   
39   0.052

gi|75236721|ref|ZP\_00720794.1|
COG2104: Sulfur transfer protein ...   
39   0.053

gi|71905747|ref|YP\_283334.1|
hypothetical protein Daro\_0105 [Dec...   
39   0.056

gi|77995330|gb|ABB14229.1|
thiamine biosynthesis protein ThiS [C...   
39   0.062

gi|94677055|ref|YP\_588522.1|
thiamine biosynthesis protein ThiS ...   
39   0.076

gi|32041399|ref|ZP\_00138982.1|
COG1977: Molybdopterin converting...   
39   0.079

gi|16767415|ref|NP\_463030.1|
sulfur carrier protein ThiS [Salmon...   
39   0.080

gi|7379114|emb|CAB83663.1|
hypothetical protein NMA0362 [Neisser...   
39   0.090

gi|28808050|dbj|BAC61287.1|
ThiS protein [Vibrio parahaemolyticu...   
39   0.091

gi|59802314|ref|YP\_209026.1|
hypothetical protein NGO2006 [Neiss...   
39   0.091

gi|56178887|gb|AAV81609.1|
Thiamine biosynthesis protein ThiS [I...   
38   0.098

gi|38198932|emb|CAE48543.1|
Conserved hypothetical protein [Cory...   
38   0.10

gi|92906590|ref|ZP\_01275370.1|
ThiamineS [Mycobacterium sp. JLS]...   
38   0.10

gi|77957008|ref|ZP\_00821077.1|
COG2104: Sulfur transfer protein ...   
38   0.11

gi|99910773|ref|ZP\_01317738.1|
hypothetical protein Bpse1\_030028...   
38   0.12

gi|92090350|ref|ZP\_01275216.1|
molybdopterin converting factor s...   
38   0.12

gi|32034642|ref|ZP\_00134789.1|
COG2104: Sulfur transfer protein ...   
38   0.12

gi|77965731|gb|ABB07111.1|
ThiS, thiamine-biosynthesis [Burkhold...   
38   0.13

gi|92914564|ref|ZP\_01283191.1|
ThiamineS [Mycobacterium sp. KMS]...   
38   0.13

gi|17427119|emb|CAD13639.1|
probable sulfur transfer protein thi...   
38   0.13

gi|77919190|ref|YP\_357005.1|
hypothetical protein Pcar\_1591 [Pel...   
38   0.14

gi|84360622|ref|ZP\_00985320.1|
COG2104: Sulfur transfer protein ...   
38   0.15

gi|84393666|ref|ZP\_00992417.1|
sulfur carrier protein ThiS [Vibr...   
38   0.16

gi|90407317|ref|ZP\_01215503.1|
sulfur carrier protein ThiS [Psyc...   
38   0.16

gi|107024240|ref|YP\_622567.1|
thiamine biosynthesis protein ThiS...   
37   0.17

gi|94501853|ref|ZP\_01308364.1|
hypothetical protein RED65\_02138 ...   
37   0.18

gi|89362382|ref|ZP\_01200190.1|
ThiS, thiamine-biosynthesis [Xant...   
37   0.19

gi|82499654|ref|ZP\_00885094.1|
ThiS, thiamine-biosynthesis [Cald...   
37   0.19

gi|66964609|ref|ZP\_00412178.1|
ThiS, thiamine-biosynthesis [Arth...   
37   0.20

gi|62424302|ref|ZP\_00379450.1|
COG2104: Sulfur transfer protein ...   
37   0.20

gi|77960648|ref|ZP\_00824510.1|
COG2104: Sulfur transfer protein ...   
37   0.21

gi|91086189|ref|XP\_971291.1|
PREDICTED: similar to CG10238-PA [T...   
37   0.22

gi|83716430|ref|YP\_438505.1|
ThiS family domain protein, putativ...   
37   0.22

gi|16762296|ref|NP\_457913.1|
sulfur carrier protein ThiS [Salmon...   
37   0.23

gi|99078622|ref|YP\_611880.1|
thiamine biosynthesis protein ThiS ...   
37   0.23

gi|5104258|dbj|BAA79573.1|
249aa long hypothetical molybdopterin...   
37   0.23

gi|69300050|ref|ZP\_00621926.1|
ThiS, thiamine-biosynthesis [Sili...   
37   0.24

gi|91762114|ref|ZP\_01264079.1|
probable molybdopterin biosynthes...   
37   0.26

gi|75176997|ref|ZP\_00697104.1|
COG4723: Phage-related protein, t...   
37   0.26

gi|86137736|ref|ZP\_01056312.1|
hypothetical protein MED193\_07738...   
37   0.28

gi|74019492|ref|ZP\_00690108.1|
ThiS, thiamine-biosynthesis [Burk...   
37   0.28

gi|86148246|ref|ZP\_01066542.1|
sulfur carrier protein ThiS [Vibr...   
37   0.28

gi|83643048|ref|YP\_431483.1|
thiamine biosynthesis protein ThiS ...   
37   0.29

gi|91228042|ref|ZP\_01262127.1|
sulfur carrier protein ThiS [Vibr...   
37   0.32

gi|88858884|ref|ZP\_01133525.1|
putative ThiS protein [Pseudoalte...   
37   0.32

gi|88949122|ref|ZP\_01151740.1|
ThiS, thiamine-biosynthesis [Halo...   
37   0.33

gi|45437725|gb|AAS63275.1|
Sulfur transfer protein involved in t...   
36   0.39

gi|51587924|emb|CAH19527.1|
thiamin biosynthesis ThiS [Yersinia ...   
36   0.40

gi|53751264|emb|CAH12675.1|
hypothetical protein [Legionella pne...   
36   0.46

gi|5105769|dbj|BAA81081.1|
272aa long hypothetical hypoxanthine ...   
36   0.47

gi|90423668|ref|YP\_532038.1|
thiamine biosynthesis protein ThiS ...   
36   0.48

gi|23508583|ref|NP\_701252.1|
hypothetical protein PF11\_0393 [Pla...   
36   0.48

gi|82705685|ref|XP\_727070.1|
hypothetical protein PY06420 [Plasm...   
36   0.51

gi|68071305|ref|XP\_677566.1|
hypothetical protein [Plasmodium be...   
36   0.51

gi|70941377|ref|XP\_740984.1|
hypothetical protein [Plasmodium ch...   
36   0.51

gi|78698313|ref|ZP\_00862816.1|
ThiS, thiamine-biosynthesis [Brad...   
36   0.53

gi|89103189|ref|ZP\_01175778.1|
COG2104: Sulfur transfer protein ...   
36   0.54

gi|78362777|gb|ABB40742.1|
thiamine biosynthesis protein ThiS [T...   
36   0.54

gi|82499276|ref|ZP\_00884724.1|
hypothetical protein CsacDRAFT\_13...   
36   0.56

gi|88934468|ref|ZP\_01140111.1|
conserved hypothetical protein [G...   
36   0.57

gi|88932583|ref|ZP\_01138273.1|
ThiS, thiamine-biosynthesis [Acid...   
36   0.60

gi|85715979|ref|ZP\_01046956.1|
ThiS, thiamine-biosynthesis [Nitr...    35  
0.69

gi|27360519|gb|AAO09455.1|
Sulfur transfer protein involved in t...   
35   0.71

gi|83589027|ref|YP\_429036.1|
hypothetical protein Moth\_0155 [Moo...   
35   0.71

gi|23011959|ref|ZP\_00052165.1|
COG2104: Sulfur transfer protein ...   
35   0.73

gi|91788153|ref|YP\_549105.1|
hypothetical protein Bpro\_2284 [Pol...   
35   0.79

gi|76883424|gb|ABA58105.1|
ThiS, thiamine-biosynthesis [Nitrosoc...   
35   0.82

gi|68561643|ref|ZP\_00600934.1|
ThiS, thiamine-biosynthesis [Rubr...   
35   0.82

gi|90412774|ref|ZP\_01220775.1|
hypothetical protein P3TCK\_22740 ...   
35   0.83

gi|9843879|emb|CAC03730.1|
RnfH protein [Pseudomonas stutzeri] >...   
35   0.83

gi|71557339|gb|AAZ36550.1|
thiamine biosynthesis protein ThiS [P...   
35   0.84

gi|87119895|ref|ZP\_01075791.1|
thiamine biosynthesis protein Thi...   
35   0.87

gi|20804221|emb|CAD31247.1|
PROBABLE THIAMIN BIOSYNTHESIS PROTEI...   
35   0.94

gi|88798339|ref|ZP\_01113924.1|
putative ThiS protein [Reinekea s...   
35   0.96

gi|91798352|gb|ABE60491.1|
thiamine biosynthesis protein ThiS [C...   
35   0.96

gi|21646638|gb|AAM71936.1|
thiamine biosynthesis protein ThiS [C...   
35   0.99

gi|94967232|ref|YP\_589280.1|
hypothetical protein Acid345\_0201 [...   
35   1.0

gi|71898215|ref|ZP\_00680389.1|
ThiS, thiamine-biosynthesis [Xyle...   
35   1.0

gi|53754222|emb|CAH15699.1|
hypothetical protein [Legionella pne...   
35   1.1

gi|17983762|gb|AAL52915.1|
hypothetical protein [Brucella melite...   
35   1.1

gi|49609716|emb|CAG73149.1|
thiamine biosynthesis protein [Erwin...    35  
1.2

gi|9105678|gb|AAF83592.1|
conserved hypothetical protein [Xylell...   
35   1.2

gi|91977895|ref|YP\_570554.1|
thiamine biosynthesis protein ThiS ...   
35   1.3

gi|86749080|ref|YP\_485576.1|
thiamine biosynthesis protein ThiS ...   
34   1.4

gi|98661971|dbj|GAA01064.1|
unnamed protein product [Pelotomacul...   
34   1.4

gi|88607118|ref|YP\_504702.1|
thiamine biosynthesis protein ThiS ...   
34   1.5

gi|18893610|gb|AAL81606.1|
hypothetical protein [Pyrococcus furi...   
34   1.5

gi|70733136|ref|YP\_262909.1|
sulfur carrier protein ThiS [Pseudo...   
34   1.6

gi|16414661|emb|CAC97377.1|
lin2147 [Listeria innocua] >gi|16801...   
34   1.6

gi|82737127|ref|ZP\_00899979.1|
conserved hypothetical protein [P...   
34   1.7

gi|77996070|gb|ABB14969.1|
thiamine biosynthesis protein ThiS [C...   
34   1.7

gi|23346995|gb|AAN29165.1|
thiamine biosynthesis protein ThiS [B...   
34   1.7

gi|77655361|gb|EAO87002.1|
ThiS, thiamine-biosynthesis [Rhodopse...   
34   1.8

gi|47093593|ref|ZP\_00231351.1|
S-adenosyl-methyltransferase MraW...   
34   1.8

gi|46908277|ref|YP\_014666.1|
S-adenosyl-methyltransferase [Liste...   
34   1.8

gi|83751114|ref|ZP\_00947528.1|
COG2104: Sulfur transfer protein ...   
34   2.0

gi|39936636|ref|NP\_948912.1|
putative thiamin biosynthesis ThiS ...    34  
2.1

gi|94416794|ref|ZP\_01296617.1|
hypothetical protein PaerP\_010013...   
34   2.1

gi|49331838|gb|AAT62484.1|
thiamine biosynthesis protein [Bacill...   
34   2.2

gi|56130244|gb|AAV79750.1|
thiamine biosynthesis protein [Salmon...   
33   2.3

gi|74421519|gb|ABA05718.1|
ThiS, thiamine-biosynthesis [Nitrobac...   
33   2.4

gi|92118391|ref|YP\_578120.1|
thiamine biosynthesis protein ThiS ...   
33   2.5

gi|21109644|gb|AAM38144.1|
conserved hypothetical protein [Xanth...   
33   2.5

gi|94414782|ref|ZP\_01294636.1|
hypothetical protein PaerP\_010033...   
33   2.5

gi|47097273|ref|ZP\_00234832.1|
S-adenosyl-methyltransferase MraW...   
33   2.5

gi|78048972|ref|YP\_365147.1|
hypothetical protein XCV3416 [Xanth...   
33   2.5

gi|77977808|ref|ZP\_00833248.1|
COG2104: Sulfur transfer protein ...   
33   2.7

gi|16411511|emb|CAD00119.1|
lmo2041 [Listeria monocytogenes] >gi...   
33   2.7

gi|53755686|emb|CAH17188.1|
hypothetical protein [Legionella pne...   
33   2.8

gi|74318145|ref|YP\_315885.1|
thiamine-biosynthesis protein ThiS ...   
33   2.9

gi|17741061|gb|AAL43548.1|
thiamin biosynthesis protein ThiG [Ag...   
33   3.0

gi|52216684|dbj|BAD49277.1|
ThiS protein involved in thiamine bi...   
33   3.0

gi|84713026|ref|ZP\_01020831.1|
ThiS, thiamine-biosynthesis [Pola...   
33   3.0

gi|33564627|emb|CAE43947.1|
conserved hypothetical protein [Bord...   
33   3.1

gi|10639813|emb|CAC11785.1|
hypothetical protein [Thermoplasma a...   
33   3.2

gi|71145353|gb|AAZ25826.1|
thiamine biosynthesis protein ThiS [C...   
33   3.2

gi|96771770|emb|CAI78352.1|
unknown hypothetical protein [Strept...   
33   3.2

gi|58582994|ref|YP\_202010.1|
hypothetical protein XOO3371 [Xanth...   
33   3.3

gi|12514736|gb|AAG55920.1|
unknown protein encoded by prophage C...   
33   3.3

gi|47501163|gb|AAT29839.1|
conserved hypothetical protein [Bacil...   
33   3.3

gi|72161447|ref|YP\_289104.1|
ThiS, thiamine-biosynthesis [Thermo...   
33   3.3

gi|49239551|emb|CAF25908.1|
Thiamin biosynthesis, thiG1 [Bartone...   
33   3.3

gi|86157547|ref|YP\_464332.1|
molybdopterin biosynthesis MoaE / t...   
33   3.4

gi|94309109|ref|YP\_582319.1|
thiamine biosynthesis protein ThiS ...   
33   3.4

gi|98662976|dbj|GAA00218.1|
unnamed protein product [Pelotomacul...   
33   3.6

gi|68263525|emb|CAI37013.1|
thiamin biosynthesis ThiS [Corynebac...   
33   3.7

gi|88949024|ref|ZP\_01151642.1|
hypothetical protein HhalDRAFT\_10...   
33   3.8

gi|65318157|ref|ZP\_00391116.1|
COG2104: Sulfur transfer protein ...   
33   3.8

gi|9946232|gb|AAG03769.1|
conserved hypothetical protein [Pseudo...   
33   3.8

gi|76794014|ref|ZP\_00776492.1|
ThiS, thiamine-biosynthesis [Pseu...   
33   4.0

gi|71836234|ref|ZP\_00676003.1|
ThiS, thiamine-biosynthesis [Pelo...   
33   4.1

gi|46201411|ref|ZP\_00055070.2|
hypothetical protein Magn03009730...   
33   4.2

gi|23493776|dbj|BAC18745.1|
hypothetical protein [Corynebacteriu...   
33   4.3

gi|83954400|ref|ZP\_00963120.1|
thiamine biosynthesis protein Thi...   
33   4.4

gi|91076742|ref|XP\_973219.1|
PREDICTED: similar to elongation pr...   
33   4.5

gi|66508519|ref|XP\_392361.2|
PREDICTED: similar to GA15696-PA [A...   
33   4.6

gi|77953948|ref|ZP\_00818350.1|
ThiS, thiamine-biosynthesis [Mari...   
33   4.7

gi|95930037|ref|ZP\_01312777.1|
conserved hypothetical protein [D...   
33   4.7

gi|28199747|ref|NP\_780061.1|
hypothetical protein PD1878 [Xylell...   
33   4.8

gi|71900707|ref|ZP\_00682830.1|
ThiS, thiamine-biosynthesis [Xyle...   
33   4.9

gi|52003327|gb|AAU23269.1|
putative methyltransferase [Bacillus ...   
33   5.0

gi|89203238|ref|ZP\_01181926.1|
ThiS, thiamine-biosynthesis [Baci...   
33   5.1

gi|27381767|ref|NP\_773296.1|
thiamin biosynthesis protein homolo...   
32   5.2

gi|34483098|emb|CAE10097.1|
hypothetical protein [Wolinella succ...   
32   5.4

gi|86605901|ref|YP\_474664.1|
GTP-binding protein EngA [Synechoco...   
32   5.7

gi|67926734|ref|ZP\_00519930.1|
hypothetical protein AcidDRAFT\_75...   
32   5.8

gi|50877366|emb|CAG37206.1|
similar to molybdopterin converting ...   
32   5.8

gi|46136055|ref|XP\_389719.1|
hypothetical protein FG09543.1 [Gib...   
32   5.9

gi|29337957|gb|AAO75760.1|
thiS protein, involved in thiamine bi...   
32   6.0

gi|68192288|gb|EAN06942.1|
ThiS, thiamine-biosynthesis [Mesorhiz...   
32   6.1

gi|34541692|ref|NP\_906171.1|
thiS protein [Porphyromonas gingiva...   
32   6.2

gi|62515019|ref|ZP\_00386512.1|
COG4283: Uncharacterized conserve...   
32   6.3

gi|67939265|ref|ZP\_00531772.1|
ThiS, thiamine-biosynthesis [Chlo...   
32   6.3

gi|68231652|ref|ZP\_00570816.1|
ThiamineS [Frankia sp. EAN1pec] >...   
32   6.4

gi|77972245|ref|ZP\_00827817.1|
COG2104: Sulfur transfer protein ...   
32   6.4

gi|6460589|gb|AAF12295.1|
conserved hypothetical protein [Deinoc...   
32   6.5

gi|90821224|gb|ABD99863.1|
S-adenosyl-methyltransferase [Lactoba...   
32   6.6

gi|87306448|ref|ZP\_01088595.1|
thiamine biosynthesis protein Thi...   
32   7.0

gi|89203108|ref|ZP\_01181805.1|
methyltransferase [Bacillus cereu...   
32   7.0

gi|49531021|emb|CAG68733.1|
hypothetical protein [Acinetobacter ...   
32   7.0

gi|58580110|ref|YP\_199126.1|
hydrolase [Xanthomonas oryzae pv. o...   
32   7.5

gi|56379488|dbj|BAD75396.1|
hypothetical conserved protein [Geob...   
32   7.8

gi|21114375|gb|AAM42419.1|
conserved hypothetical protein [Xanth...   
32   8.0

gi|56680462|gb|AAV97128.1|
thiamine biosynthesis protein ThiS [S...   
32   8.3

gi|93454223|gb|EAT04541.1|
ThiS, thiamine-biosynthesis [delta pr...   
32   8.5

gi|60493471|emb|CAH08257.1|
putative thiamine biosynthesis-relat...   
32   8.7

gi|29894449|gb|AAP07739.1| ThiS
protein [Bacillus cereus ATCC 14...   
32   9.0

gi|88946144|ref|ZP\_01149232.1|
conserved hypothetical protein [D...   
32   9.2

gi|29832636|ref|NP\_827270.1|
sulfur transfer protein involved in...   
32   9.3

gi|48870419|ref|ZP\_00323142.1|
COG0275: Predicted S-adenosylmeth...   
32   9.8

gi|49180695|gb|AAT56071.1|
S-adenosyl-methyltransferase MraW [Ba...   
32   9.8

gi|49330004|gb|AAT60650.1|
S-adenosyl-methyltransferase [Bacillu...   
32   9.8

gi|78167320|gb|ABB24418.1|
ThiS, thiamine-biosynthesis [Pelodict...   
32   9.9

 

------------------------------------------------------------

Reference for
composition-based statistics:

Schaffer, Alejandro A., L.
Aravind, Thomas L. Madden,

Sergei Shavirin, John L.
Spouge, Yuri I. Wolf,

Eugene V. Koonin, and Stephen F. Altschul (2001),

"Improving the
accuracy of PSI-BLAST protein database searches with

composition-based
statistics and other refinements", 
Nucleic Acids Res. 29:2994-3005.

 

Query=
Bcep1808DRAFT\_0451\_Bvie\_67549235   [RnfH]

         (77 letters)

 

Database: All non-redundant
GenBank CDS

translations+PDB+SwissProt+PIR+PRF
excluding environmental samples

           3,682,060 sequences; 1,264,608,837
total letters

 

Results
from round 21

 

gi|57160377|dbj|BAD86307.1|
molybdopterin converting factor, sub...   
84   1e-15

gi|68545076|ref|ZP\_00584627.1|
Molybdopterin converting factor, ...   
82   8e-15

gi|76261574|ref|ZP\_00769179.1|
Molybdopterin converting factor, ...   
81   1e-14

gi|16764168|ref|NP\_459783.1|
molybdopterin biosynthetic protein ...   
80   2e-14

gi|16759729|ref|NP\_455346.1|
molybdopterin converting factor, su...   
80   2e-14

gi|62179373|ref|YP\_215790.1|
molybdopterin biosynthesis protein ...   
80   2e-14

gi|90407696|ref|ZP\_01215875.1|
molybdopterin biosynthesis protei...   
80   2e-14

gi|88795121|ref|ZP\_01110822.1|
molybdopterin converting factor, ...   
80   2e-14

gi|90579138|ref|ZP\_01234948.1|
hypothetical protein VAS14\_05513 ...   
80   3e-14

gi|82776146|ref|YP\_402493.1|
molybdopterin biosynthesis [Shigell...   
79   5e-14

gi|56383279|gb|AAN42369.2|
molybdopterin biosynthesis protein D ...   
79   7e-14

gi|5458838|emb|CAB50325.1|
moaD molybdopterin synthase, small su...   
79   8e-14

gi|36784880|emb|CAE13794.1|
molybdopterin [mpt] converting facto...   
79   8e-14

gi|46200249|ref|YP\_005916.1|
molybdopterin (MPT) converting fact...   
79   8e-14

gi|15830116|ref|NP\_308889.1|
molybdopterin biosynthesis protein ...   
79   8e-14

gi|46202194|ref|ZP\_00208426.1|
COG2914: Uncharacterized protein ...   
78   8e-14

gi|55771441|dbj|BAD69882.1|
molybdenum cofactor biosynthesis pro...   
78   9e-14

gi|26107155|gb|AAN79339.1|
Molybdopterin converting factor subun...   
78   9e-14

gi|46202216|ref|ZP\_00208434.1|
COG2914: Uncharacterized protein ...   
78   1e-13

gi|53802996|ref|YP\_115212.1|
electron transport complex, H subun...   
78   1e-13

gi|106888351|ref|ZP\_01355551.1|
molybdopterin converting factor,...   
78   1e-13

gi|71555514|gb|AAZ34725.1|
molybdopterin converting factor, subu...   
78   1e-13

gi|34495641|ref|NP\_899856.1|
molybdopterin-converting factor sub...   
77   1e-13

gi|90411674|ref|ZP\_01219684.1|
hypothetical protein P3TCK\_16464 ...   
77   2e-13

gi|77814639|ref|ZP\_00813897.1|
Molybdopterin converting factor, ...   
77   2e-13

gi|76883017|gb|ABA57698.1|
Protein of unknown function UPF0125 [...   
77   2e-13

gi|74019145|ref|ZP\_00689763.1|
Molybdopterin converting factor, ...   
77   2e-13

gi|49612264|emb|CAG75714.1|
molybdopterin converting factor subu...   
77   2e-13

gi|91775615|ref|YP\_545371.1|
molybdopterin converting factor, su...    77  
2e-13

gi|75189826|ref|ZP\_00703093.1|
COG1977: Molybdopterin converting...   
77   2e-13

gi|68541239|ref|ZP\_00580985.1|
Molybdopterin converting factor, ...   
77   2e-13

gi|1787002|gb|AAC73871.1|
molybdopterin synthase, small subunit ...   
77   2e-13

gi|88800262|ref|ZP\_01115829.1|
molybdopterin biosynthesis protei...   
77   2e-13

gi|77958607|ref|ZP\_00822638.1|
COG2914: Uncharacterized protein ...   
77   3e-13

gi|83311718|ref|YP\_421982.1|
hypothetical protein amb2619 [Magne...   
77   3e-13

gi|77951750|ref|ZP\_00816169.1|
Molybdopterin converting factor, ...   
77   3e-13

gi|75823609|ref|ZP\_00753099.1|
COG1977: Molybdopterin converting...   
77   3e-13

gi|23467044|ref|ZP\_00122629.1|
COG1977: Molybdopterin converting...   
76   3e-13

gi|28851697|gb|AAO54773.1|
molybdenum cofactor biosynthesis prot...   
76   3e-13

gi|77974741|ref|ZP\_00830280.1|
COG2914: Uncharacterized protein ...   
76   3e-13

gi|63255028|gb|AAY36124.1|
Molybdopterin converting factor, subu...   
76   3e-13

gi|75236193|ref|ZP\_00720310.1|
COG1977: Molybdopterin converting...   
76   3e-13

gi|77978427|ref|ZP\_00833856.1|
COG2914: Uncharacterized protein ...   
76   3e-13

gi|75819545|ref|ZP\_00749620.1|
COG1977: Molybdopterin converting...   
76   3e-13

gi|59711549|ref|YP\_204325.1|
molybdopterin converting factor, sm...   
76   4e-13

gi|32033743|ref|ZP\_00134038.1|
COG1977: Molybdopterin converting...   
76   4e-13

gi|33152460|ref|NP\_873813.1|
molybdopterin converting factor sub...   
76   4e-13

gi|42011|emb|CAA49864.1|
moaD [Escherichia coli]                      
76   4e-13

gi|30749759|pdb|1NVI|D
Chain D, Orthorhombic Crystal Form Of Mol...   
76   4e-13

gi|33359306|ref|NP\_877770.1|
putative molybdopterin converting f...   
76   4e-13

gi|94984437|ref|YP\_603801.1|
molybdopterin converting factor, su...   
76   4e-13

gi|77632096|ref|ZP\_00794682.1|
COG2914: Uncharacterized protein ...   
76   4e-13

gi|67543768|ref|ZP\_00421699.1|
Molybdopterin converting factor, ...   
76   4e-13

gi|107028970|ref|YP\_626065.1|
molybdopterin converting factor, s...   
76   5e-13

gi|27362554|gb|AAO11408.1|
Molybdenum cofactor biosynthesis prot...   
76   5e-13

gi|89073364|ref|ZP\_01159888.1|
hypothetical protein SKA34\_20482 ...   
76   5e-13

gi|18159566|gb|AAL62982.1|
molybdenum cofactor biosynthesis prot...  
 76   5e-13

gi|12620119|gb|AAG60573.1|
putative molybdenum cofactor biosynth...   
76   5e-13

gi|86608912|ref|YP\_477674.1|
molybdopterin converting factor, su...   
75   6e-13

gi|12720897|gb|AAK02707.1| MoaD [Pasteurella multocida subsp. mu...    75  
6e-13

gi|33356787|ref|NP\_127095.2|
molybdopterin converting factor, su...   
75   7e-13

gi|9655490|gb|AAF94186.1|
molybdenum cofactor biosynthesis prote...   
75   8e-13

gi|56312934|emb|CAI07579.1|
Protein rnfH [Azoarcus sp. EbN1] >gi...   
75   8e-13

gi|24375928|ref|NP\_719971.1|
molybdenum cofactor biosynthesis pr...   
75   8e-13

gi|23347498|gb|AAN29626.1|
molybdopterin converting factor, subu...   
75   8e-13

gi|9843879|emb|CAC03730.1|
RnfH protein [Pseudomonas stutzeri] >...   
75   8e-13

gi|45435745|gb|AAS61303.1|
conserved hypothetical protein [Yersi...   
75   9e-13

gi|75210621|ref|ZP\_00710769.1|
COG1977: Molybdopterin converting...   
75   9e-13

gi|52696120|pdb|1VJK|A
Chain A, Putative Molybdopterin Convertin...   
75   9e-13

gi|94969084|ref|YP\_591132.1|
molybdopterin converting factor, su...   
75   1e-12

gi|40063292|gb|AAR38110.1|
conserved hypothetical protein [uncul...   
75   1e-12

gi|16761538|ref|NP\_457155.1|
hypothetical protein STY2872 [Salmo...   
75   1e-12

gi|84362038|ref|ZP\_00986675.1|
COG1977: Molybdopterin converting...   
75   1e-12

gi|18892532|gb|AAL80667.1|
molybdopterin converting factor, subu...   
75   1e-12

gi|15607139|ref|NP\_213755.1|
Molybdopterin converting factor, sm...   
75   1e-12

gi|78691076|ref|ZP\_00855707.1|
Molybdopterin converting factor, ...   
74   1e-12

gi|77967366|gb|ABB08746.1|
Molybdopterin converting factor, subu...   
74   1e-12

gi|52307130|gb|AAU37630.1|
MoaD protein [Mannheimia succinicipro...   
74   1e-12

gi|56544278|gb|AAV90432.1|
conserved hypothetical protein [Zymom...   
74   1e-12

gi|77963358|ref|ZP\_00827168.1|
COG2914: Uncharacterized protein ...   
74   2e-12

gi|12720385|gb|AAK02250.1|
unknown [Pasteurella multocida subsp....   
74   2e-12

gi|13883249|gb|AAK47766.1|
molybdopterin cofactor biosynthesis p...   
74   2e-12

gi|71907414|ref|YP\_285001.1|
Molybdopterin converting factor, su...   
74   2e-12

gi|85711977|ref|ZP\_01043031.1|
hypothetical protein OS145\_12789 ...   
74   2e-12

gi|26109388|gb|AAN81590.1|
Protein yfjF [Escherichia coli CFT073...  
 74   3e-12

gi|67549235|ref|ZP\_00427105.1|
Protein of unknown function UPF01...   
73   3e-12

gi|91784097|ref|YP\_559303.1|
hypothetical protein Bxe\_A1704 [Bur...   
73   3e-12

gi|75431111|ref|ZP\_00732938.1|
molybdopterin (MPT) converting fa...   
73   3e-12

gi|68057197|gb|AAX87450.1|
conserved hypothetical protein [Haemo...   
73   3e-12

gi|84713090|ref|ZP\_01020876.1|
Molybdopterin converting factor, ...   
73   3e-12

gi|82545161|ref|YP\_409108.1|
hypothetical protein SBO\_2753 [Shig...   
73   3e-12

gi|56315292|emb|CAI09937.1|
Molybdopterin (MPT) converting facto...   
73   3e-12

gi|77976770|ref|ZP\_00832242.1|
COG1977: Molybdopterin converting...   
73   3e-12

gi|77973923|ref|ZP\_00829467.1|
COG1977: Molybdopterin converting...   
73   4e-12

gi|78366785|ref|ZP\_00837062.1|
Molybdopterin converting factor, ...   
73   4e-12

gi|74020038|ref|ZP\_00690646.1|
Protein of unknown function UPF01...   
73   4e-12

gi|74313178|ref|YP\_311597.1|
hypothetical protein SSO\_2745 [Shig...   
73   4e-12

gi|31340487|sp|Q8FEY5|YFJF\_ECOL6
UPF0125 protein yfjF >gi|752416...   
73   4e-12

gi|87082139|gb|AAC75667.2|
predicted protein [Escherichia coli K...   
73   4e-12

gi|45435691|gb|AAS61249.1|
molybdopterin [mpt] converting factor...   
73   4e-12

gi|56383706|gb|AAN44172.2|
orf, conserved hypothetical protein [...   
72   5e-12

gi|83720736|ref|YP\_442722.1|
molybdopterin converting factor, su...   
72   5e-12

gi|10640334|emb|CAC12148.1|
MoaD (involved in molybdopterin synt...   
72   5e-12

gi|16766001|ref|NP\_461616.1|
hypothetical protein STM2686 [Salmo...   
72   5e-12

gi|77957352|ref|ZP\_00821410.1|
COG1977: Molybdopterin converting...   
72   5e-12

gi|58001333|gb|AAW60227.1|
Bifunctional molybdenum cofactor bios...   
72   5e-12

gi|52306665|gb|AAU37165.1|
unknown [Mannheimia succiniciproducen...   
72   6e-12

gi|77967521|gb|ABB08901.1|
protein of unknown function UPF0125 [...   
72   6e-12

gi|82777976|ref|YP\_404325.1|
hypothetical protein SDY\_2791 [Shig...   
72   7e-12

gi|68245723|gb|EAN27838.1|
Protein of unknown function UPF0125 [...   
72   7e-12

gi|91788067|ref|YP\_549019.1|
thiamineS [Polaromonas sp. JS666] >...   
72   7e-12

gi|83719599|ref|YP\_442575.1|
TGS domain protein [Burkholderia th...   
72   7e-12

gi|15832734|ref|NP\_311507.1|
hypothetical protein ECs3480 [Esche...    72  
8e-12

gi|85059780|ref|YP\_455482.1|
hypothetical protein SG1802 [Sodali...   
72   8e-12

gi|35214941|dbj|BAC92307.1|
gsr4366 [Gloeobacter violaceus PCC 7...   
72   8e-12

gi|91770046|ref|ZP\_01271876.1|
Molybdopterin converting factor, ...   
72   9e-12

gi|71676256|ref|ZP\_00673998.1|
ThiamineS [Trichodesmium erythrae...   
72   9e-12

gi|46912747|emb|CAG19537.1|
hypothetical protein [Photobacterium...   
72   1e-11

gi|18202332|sp|P58318|YFJF\_ECO57
UPF0125 protein yfjF                 
71   1e-11

gi|17428346|emb|CAD15033.1|
probable molybdopterin mpt convertin...   
71   1e-11

gi|88948276|ref|ZP\_01151039.1|
conserved hypothetical protein [H...   
71   1e-11

gi|84355032|ref|ZP\_00979924.1|
COG2914: Uncharacterized protein ...   
71   1e-11

gi|11095295|gb|AAG29821.1|
RnfH protein [Azotobacter vinelandii]...   
71   1e-11

gi|94415786|ref|ZP\_01295621.1|
hypothetical protein PaerP\_010023...   
71   1e-11

gi|84362161|ref|ZP\_00986796.1|
COG2914: Uncharacterized protein ...   
71   1e-11

gi|82702330|ref|YP\_411896.1|
hypothetical protein Nmul\_A1201 [Ni...   
71   1e-11

gi|88809412|ref|ZP\_01124920.1|
molydbenum cofactor biosynthesis ...   
71   1e-11

gi|23016726|ref|ZP\_00056479.1|
COG1977: Molybdopterin converting...   
71   1e-11

gi|78362790|gb|ABB40755.1|
molybdopterin converting factor, subu...   
71   1e-11

gi|52427038|gb|AAU47631.1|
molybdopterin converting factor, subu...   
71   1e-11

gi|6460436|gb|AAF12145.1|
molybdenum cofactor biosynthesis prote...   
71   1e-11

gi|83748544|ref|ZP\_00945564.1|
Molybdopterin converting factor, ...   
71   1e-11

gi|13883002|gb|AAK47534.1|
molybdenum cofactor biosynthesis prot...   
71   1e-11

gi|42782629|ref|NP\_979876.1|
molybdopterin converting factor, su...   
71   1e-11

gi|49082512|gb|AAT50656.1|
PA3917 [synthetic construct]               
71   2e-11

gi|107028823|ref|YP\_625918.1|
protein of unknown function UPF012...   
71   2e-11

gi|84393380|ref|ZP\_00992139.1|
Molybdenum cofactor biosynthesis ...   
71   2e-11

gi|32039081|ref|ZP\_00137353.1|
COG1977: Molybdopterin converting...   
71   2e-11

gi|9950101|gb|AAG07304.1|
molybdopterin converting factor, small...   
71   2e-11

gi|36786684|emb|CAE15750.1|
unnamed protein product [Photorhabdu...   
71   2e-11

gi|74421578|gb|ABA05777.1|
molybdopterin converting factor, subu...   
71   2e-11

gi|88795306|ref|ZP\_01111005.1|
hypothetical protein MADE\_19606 [...   
70   2e-11

gi|49610307|emb|CAG73751.1|
conserved hypothetical protein [Erwi...   
70   2e-11

gi|90407677|ref|ZP\_01215857.1|
hypothetical protein PCNPT3\_09004...    70  
2e-11

gi|49330814|gb|AAT61460.1|
molybdopterin converting factor, subu...   
70   2e-11

gi|86147951|ref|ZP\_01066255.1|
Molybdenum cofactor biosynthesis ...   
70   2e-11

gi|88793099|ref|ZP\_01108816.1|
molybdopterin converting factor, ...   
70   2e-11

gi|2507066|sp|P45309|MOAD\_HAEIN
Molybdopterin-converting factor ...   
70   2e-11

gi|91791472|ref|YP\_561123.1|
molybdopterin converting factor, su...   
70   2e-11

gi|85716037|ref|ZP\_01047014.1|
molybdopterin converting factor, ...   
70   2e-11

gi|56315277|emb|CAI09922.1|
conserved hypothetical protein [Azoa...   
70   2e-11

gi|76791575|ref|ZP\_00774081.1|
Protein of unknown function UPF01...   
70   2e-11

gi|56179014|gb|AAV81736.1|
Uncharacterized conserved protein [Id...   
70   2e-11

gi|89056408|ref|YP\_511859.1|
molybdopterin converting factor, su...   
70   2e-11

gi|47568924|ref|ZP\_00239616.1|
molybdopterin converting factor, ...   
70   2e-11

gi|91775496|ref|YP\_545252.1|
protein of unknown function UPF0125...   
70   2e-11

gi|78693266|ref|ZP\_00857780.1|
Molybdopterin converting factor, ...   
70   3e-11

gi|77960819|ref|ZP\_00824674.1|
COG1977: Molybdopterin converting...   
70   3e-11

gi|67156787|ref|ZP\_00418284.1|
ThiamineS [Azotobacter vinelandii...   
70   3e-11

gi|83312895|ref|YP\_423159.1|
Molybdopterin converting factor, sm...   
70   3e-11

gi|49180290|gb|AAT55666.1|
molybdopterin converting factor, subu...   
70   3e-11

gi|77996211|gb|ABB15110.1|
molybdopterin converting factor, subu...   
70   3e-11

gi|68058473|gb|AAX88726.1|
molybdopterin converting factor subun...   
70   3e-11

gi|42631902|ref|ZP\_00157440.1|
COG1977: Molybdopterin converting...   
70   3e-11

gi|76579340|gb|ABA48815.1|
TGS domain protein [Burkholderia pseu...   
70   4e-11

gi|90412545|ref|ZP\_01220548.1|
hypothetical protein P3TCK\_14273 ...   
70   4e-11

gi|78701201|ref|ZP\_00865653.1|
conserved hypothetical protein [A...   
69   4e-11

gi|88860469|ref|ZP\_01135107.1|
hypothetical protein PTD2\_15632 [...   
69   4e-11

gi|53802819|ref|YP\_115441.1|
molybdopterin converting factor, su...   
69   5e-11

gi|1905814|emb|CAA72667.1|
RnfH protein [Rhodobacter capsulatus]...   
69   5e-11

gi|70731087|ref|YP\_260828.1|
molybdopterin converting factor, su...   
69   5e-11

gi|92118611|ref|YP\_578340.1|
molybdopterin converting factor, su...    69  
5e-11

gi|56908910|dbj|BAD63437.1|
molybdopterin converting factor subu...   
69   6e-11

gi|69936172|ref|ZP\_00631032.1|
Molybdopterin converting factor, ...   
69   6e-11

gi|42629022|ref|ZP\_00154572.1|
COG1977: Molybdopterin converting...   
69   6e-11

gi|83368880|ref|ZP\_00913740.1|
protein RnfH [Rhodobacter sphaero...   
69   6e-11

gi|88950646|ref|ZP\_01153220.1|
MoaD, archaeal [Methanosaeta ther...   
69   7e-11

gi|26988028|ref|NP\_743453.1|
molybdenum cofactor biosynthesis pr...   
69   7e-11

gi|77742224|ref|ZP\_00810703.1|
Molybdopterin converting factor, ...   
69   7e-11

gi|82737924|ref|ZP\_00900767.1|
Molybdopterin converting factor, ...   
69   7e-11

gi|74317772|ref|YP\_315512.1|
hypothetical protein Tbd\_1754 [Thio...   
69   8e-11

gi|52429778|gb|AAU50371.1|
TGS domain protein [Burkholderia mall...   
69   8e-11

gi|94496324|ref|ZP\_01302901.1|
molybdopterin converting factor, ...   
69   8e-11

gi|86748300|ref|YP\_484796.1|
molybdopterin converting factor, su...   
69   8e-11

gi|86605021|ref|YP\_473784.1|
MoaD family protein [Synechococcus ...   
68   9e-11

gi|53692251|ref|ZP\_00123587.2|
COG2914: Uncharacterized protein ...   
68   9e-11

gi|47551893|gb|AAT32729.2|
molybdopterin converting factor, subu...   
68   9e-11

gi|29897221|gb|AAP10498.1|
Molybdopterin (MPT) converting factor...   
68   1e-10

gi|94310901|ref|YP\_584111.1|
molybdopterin converting factor, su...   
68   1e-10

gi|46156590|ref|ZP\_00204738.1|
COG2914: Uncharacterized protein ...   
68   1e-10

gi|83644081|ref|YP\_432516.1|
hypothetical protein HCH\_01218 [Hah...   
68   1e-10

gi|93005538|ref|YP\_579975.1|
thiamineS [Psychrobacter cryohalole...   
68   1e-10

gi|71143753|gb|AAZ24226.1|
molybdopterin converting factor, subu...   
68   1e-10

gi|91975759|ref|YP\_568418.1|
molybdopterin converting factor, su...   
68   1e-10

gi|15156160|gb|AAK86930.1| AGR\_C\_2086p [Agrobacterium tumefacien...    68  
1e-10

gi|77381195|gb|ABA72708.1| MoaD [Pseudomonas fluorescens PfO-1] ...    68  
1e-10

gi|84319278|ref|ZP\_00967677.1| COG0315: Molybdenum cofactor bios...    68  
1e-10

gi|84685238|ref|ZP\_01013137.1| molybdopterin converting factor, ...    68  
1e-10

gi|83372801|ref|ZP\_00917580.1| protein RnfH [Rhodobacter sphaero...    68  
1e-10

gi|91227353|ref|ZP\_01261742.1| molybdenum cofactor biosynthesis ...    68  
1e-10

gi|23395272|gb|AAN31771.1| molybdopterin converting factor, smal...    68  
1e-10

gi|46912321|emb|CAG19113.1| conserved hypothetical protein [Phot...    68  
1e-10

gi|21112021|gb|AAM40299.1| molybdopterin-converting factor chain...    67  
2e-10

gi|7379707|emb|CAB84274.1| hypothetical protein NMA1005 [Neisser...    67  
2e-10

gi|106883354|ref|ZP\_01350752.1| ThiamineS [Psychromonas ingraham...    67  
2e-10

gi|77389630|gb|ABA80814.1| probable rnfH protein [Rhodobacter sp...    67  
2e-10

gi|56379152|dbj|BAD75060.1|
molybdopterin converting factor (sub...   
67   2e-10

gi|72119091|gb|AAZ61354.1|
Molybdopterin converting factor, subu...   
67   2e-10

gi|78364037|gb|ABB42002.1|
Protein of unknown function UPF0125 [...   
67   2e-10

gi|106880965|ref|ZP\_01348370.1|
Protein of unknown function UPF0...   
67   2e-10

gi|90419510|ref|ZP\_01227420.1|
molybdopterin converting factor s...   
67   2e-10

gi|91225103|ref|ZP\_01260325.1|
hypothetical protein V12G01\_12785...   
67   2e-10

gi|75701250|gb|ABA20926.1|
ThiamineS [Anabaena variabilis ATCC 2...   
67   2e-10

gi|75857320|ref|ZP\_00764936.1|
COG2914: Uncharacterized protein ...   
67   2e-10

gi|75855800|ref|ZP\_00763441.1|
COG1977: Molybdopterin converting...   
67   2e-10

gi|74317044|ref|YP\_314784.1|
molybdopterin converting factor, su...   
67   2e-10

gi|9655302|gb|AAF94012.1|
conserved hypothetical protein [Vibrio...   
67   2e-10

gi|67925013|ref|ZP\_00518396.1|
ThiamineS [Crocosphaera watsonii ...   
67   2e-10

gi|68562526|ref|ZP\_00601781.1|
Molybdopterin converting factor, ...   
67   2e-10

gi|14027318|dbj|BAB54272.1|
molybdopterin converting factor, sub...   
67   2e-10

gi|83026442|gb|ABB96257.1|
VP15 [Zea mays]                            
67   2e-10

gi|91220879|ref|ZP\_01257071.1|
putative molybdopterin MPT conver...   
67   2e-10

gi|76791187|ref|ZP\_00773695.1|
ThiamineS [Pseudoalteromonas atla...   
67   2e-10

gi|75831932|ref|ZP\_00761191.1|
COG2914: Uncharacterized protein ...   
67   2e-10

gi|89900702|ref|YP\_523173.1|
molybdopterin converting factor, su...   
67   3e-10

gi|75762421|ref|ZP\_00742290.1|
Molybdopterin converting factor, ...   
67   3e-10

gi|23126667|ref|ZP\_00108556.1|
COG1977: Molybdopterin converting...   
67   3e-10

gi|1723617|sp|P52120|Y850\_VIBCH
UPF0125 protein VC0850 >gi|11008...   
67   3e-10

gi|94500148|ref|ZP\_01306682.1|
hypothetical protein RED65\_13472 ...   
67   3e-10

gi|75822958|ref|ZP\_00752503.1|
COG2914: Uncharacterized protein ...   
67   3e-10

gi|89070177|ref|ZP\_01157505.1|
putative molybdopterin MPT conver...    67  
3e-10

gi|92089022|ref|ZP\_01273976.1|
ThiamineS [Lactobacillus reuteri ...   
67   3e-10

gi|77389071|gb|ABA80256.1|
putative molybdopterin MPT converting...   
67   3e-10

gi|18160633|gb|AAL63972.1|
conserved hypothetical protein [Pyrob...   
67   3e-10

gi|89093577|ref|ZP\_01166525.1|
molybdopterin converting factor, ...   
66   3e-10

gi|4262375|gb|AAD14600.1|
molybdopterin-synthase small subunit [...   
66   3e-10

gi|51858004|dbj|BAD42162.1|
molybdopterin converting factor-like...   
66   3e-10

gi|32035426|ref|ZP\_00135397.1|
COG2914: Uncharacterized protein ...   
66   4e-10

gi|27359955|gb|AAO08893.1|
Conserved hypothetical protein [Vibri...   
66   4e-10

gi|59800824|ref|YP\_207536.1|
hypothetical protein NGO0380 [Neiss...   
66   4e-10

gi|70734317|ref|YP\_257957.1|
hypothetical protein PFL\_0822 [Pseu...   
66   5e-10

gi|83369898|ref|ZP\_00914747.1|
Molybdopterin converting factor, ...   
66   5e-10

gi|28807086|dbj|BAC60356.1|
molybdenum cofactor biosynthesis pro...   
66   5e-10

gi|77918070|ref|YP\_355885.1|
molybdopterin converting factor, su...   
66   5e-10

gi|78170141|gb|ABB27238.1|
molydbenum cofactor biosynthesis prot...   
66   5e-10

gi|17133489|dbj|BAB76052.1|
asl4353 [Nostoc sp. PCC 7120] >gi|17...   
66   5e-10

gi|28805632|dbj|BAC58909.1|
conserved hypothetical protein [Vibr...   
66   5e-10

gi|83648509|ref|YP\_436944.1|
Molybdopterin converting factor, sm...   
65   6e-10

gi|90022254|ref|YP\_528081.1|
Helix-turn-helix, AraC type [Saccha...   
65   7e-10

gi|83372471|ref|ZP\_00917251.1|
Molybdopterin converting factor, ...   
65   8e-10

gi|86610242|ref|YP\_479004.1|
MoaD family protein [Synechococcus ...   
65   8e-10

gi|71907959|ref|YP\_285546.1|
hypothetical protein Daro\_2340 [Dec...   
65   9e-10

gi|27382583|ref|NP\_774112.1|
molybdopterin converting factor sma...   
65   9e-10

gi|68545933|ref|ZP\_00585482.1|
Protein of unknown function UPF01...   
65   9e-10

gi|83594146|ref|YP\_427898.1|
Molybdopterin converting factor, su...   
65   1e-09

gi|30681325|ref|NP\_849354.1|
CNX7/SIR5; catalytic [Arabidopsis t...   
65   1e-09

gi|89201285|ref|ZP\_01180030.1|
Molybdopterin converting factor, ...   
65   1e-09

gi|11499216|ref|NP\_070453.1|
molybdopterin converting factor, su...   
65   1e-09

gi|83941421|ref|ZP\_00953883.1|
putative molybdopterin MPT conver...    65  
1e-09

gi|78366485|ref|ZP\_00836765.1|
Protein of unknown function UPF01...   
65   1e-09

gi|47573807|ref|ZP\_00243844.1|
COG1977: Molybdopterin converting...   
65   1e-09

gi|17739525|gb|AAL42140.1|
molybdopterin converting factor small...   
64   1e-09

gi|70728421|ref|YP\_258170.1|
molybdenum cofactor biosynthesis pr...   
64   1e-09

gi|58580685|ref|YP\_199701.1|
molybdopterin-converting factor cha...   
64   2e-09

gi|88801004|ref|ZP\_01116554.1|
hypothetical protein MED297\_09656...   
64   2e-09

gi|87301504|ref|ZP\_01084344.1|
molydbenum cofactor biosynthesis ...   
64   2e-09

gi|29898350|gb|AAP11623.1|
Molybdopterin (MPT) converting factor...   
64   2e-09

gi|21107237|gb|AAM35973.1|
molybdopterin-converting factor chain...   
64   2e-09

gi|69951944|ref|ZP\_00639556.1|
Molybdopterin converting factor, ...   
64   2e-09

gi|47502579|gb|AAT31255.1|
molybdopterin converting factor, subu...   
64   2e-09

gi|84387679|ref|ZP\_00990696.1|
hypothetical protein V12B01\_09416...   
64   2e-09

gi|49330166|gb|AAT60812.1|
molybdopterin converting factor, subu...   
64   2e-09

gi|89072613|ref|ZP\_01159185.1|
hypothetical protein SKA34\_18849 ...   
64   2e-09

gi|91220231|ref|ZP\_01256641.1|
hypothetical protein P700755\_3102...   
64   2e-09

gi|94310400|ref|YP\_583610.1|
protein of unknown function UPF0125...   
64   2e-09

gi|50909475|ref|XP\_466226.1|
unknown protein [Oryza sativa (japo...   
64   2e-09

gi|71492602|gb|EAO24906.1|
ThiamineS [Syntrophomonas wolfei subs...   
64   2e-09

gi|84499780|ref|ZP\_00998068.1|
putative molybdopterin MPT conver...   
64   2e-09

gi|91783438|ref|YP\_558644.1|
Molybdopterin converting factor, su...   
64   2e-09

gi|83952487|ref|ZP\_00961218.1|
molybdopterin converting factor, ...   
64   2e-09

gi|88704211|ref|ZP\_01101925.1|
conserved hypothetical protein [g...   
64   2e-09

gi|72162768|ref|YP\_290425.1|
hypothetical protein Tfu\_2369 [Ther...   
64   2e-09

gi|34498917|ref|NP\_903132.1|
hypothetical protein CV3462 [Chromo...   
64   2e-09

gi|88932029|ref|ZP\_01137722.1|
conserved hypothetical protein [A...   
64   2e-09

gi|89204215|ref|ZP\_01182793.1|
Molybdopterin converting factor, ...   
64   2e-09

gi|104783496|ref|YP\_609994.1|
molybdenum cofactor biosynthesis p...   
64   2e-09

gi|88812323|ref|ZP\_01127573.1|
hypothetical protein NB231\_01014 ...    64  
3e-09

gi|51974253|gb|AAU15803.1|
molybdopterin converting factor, subu...   
64   3e-09

gi|85705896|ref|ZP\_01036992.1|
putative molybdopterin MPT conver...   
63   3e-09

gi|78046664|ref|YP\_362839.1|
molybdopterin-converting factor cha...   
63   3e-09

gi|77382376|gb|ABA73889.1|
ThiamineS [Pseudomonas fluorescens Pf...   
63   3e-09

gi|86145628|ref|ZP\_01063958.1|
hypothetical protein MED222\_01702...   
63   3e-09

gi|18892299|gb|AAL80469.1|
molybdopterin converting factor, subu...   
63   3e-09

gi|35212628|dbj|BAC90002.1|
gsl2061 [Gloeobacter violaceus PCC 7...   
63   3e-09

gi|29895812|gb|AAP09094.1|
Molybdopterin (MPT) converting factor...   
63   3e-09

gi|28631173|ref|NP\_789776.1|
molybdopterin synthase small subuni...   
63   4e-09

gi|84515715|ref|ZP\_01003076.1|
molybdopterin converting factor, ...   
63   4e-09

gi|68553535|ref|ZP\_00592907.1|
ThiamineS [Prosthecochloris aestu...   
63   4e-09

gi|33867003|ref|NP\_898562.1|
molydbenum cofactor biosynthesis pr...   
63   4e-09

gi|90581513|ref|ZP\_01237306.1|
hypothetical protein VAS14\_07154 ...   
63   4e-09

gi|99078603|ref|YP\_611861.1|
molybdopterin converting factor, su...   
63   5e-09

gi|89206760|ref|ZP\_01185313.1|
Molybdopterin converting factor, ...   
62   5e-09

gi|88938982|ref|ZP\_01144434.1|
molybdopterin converting factor, ...   
62   5e-09

gi|59712607|ref|YP\_205383.1|
hypothetical protein VF2000 [Vibrio...   
62   5e-09

gi|42783907|ref|NP\_981154.1|
molybdopterin converting factor, su...   
62   5e-09

gi|22652759|gb|AAN03805.1|
10 kDa hypothetical protein [Azotobac...   
62   6e-09

gi|83854898|ref|ZP\_00948428.1|
molybdopterin converting factor, ...   
62   6e-09

gi|57159324|dbj|BAD85254.1|
molybdopterin converting factor, sub...   
62   6e-09

gi|51976766|gb|AAU18316.1|
molybdopterin biosynthesis protein, s...   
62   7e-09

gi|89100759|ref|ZP\_01173613.1|
molybdopterin converting factor, ...   
62   7e-09

gi|2633802|emb|CAB13304.1|
molybdopterin converting factor (subu...   
62   7e-09

gi|49333106|gb|AAT63752.1|
molybdopterin biosynthesis protein, s...   
62   7e-09

gi|4457226|gb|AAD21202.1|
MoaD [Rhodobacter capsulatus]               
62   8e-09

gi|47505417|gb|AAT34093.1|
molybdopterin converting factor, subu...   
62   8e-09

gi|47566905|ref|ZP\_00237623.1|
molybdopterin converting factor, ...   
62   9e-09

gi|28271030|emb|CAD63935.1|
molybdopterin biosynthesis protein, ...   
62   9e-09

gi|22295085|dbj|BAC08913.1|
molybdopterin biosynthesis protein D...   
62   1e-08

gi|16330728|ref|NP\_441456.1|
hypothetical protein slr0821 [Synec...    62  
1e-08

gi|71143975|gb|AAZ24448.1|
conserved hypothetical protein [Colwe...   
62   1e-08

gi|34482542|emb|CAE09542.1|
hypothetical protein [Wolinella succ...   
62   1e-08

gi|88935576|ref|ZP\_01141206.1|
similar to molybdopterin converti...   
61   1e-08

gi|15645420|ref|NP\_207594.1|
molybdopterin converting factor, su...   
61   1e-08

gi|71557339|gb|AAZ36550.1|
thiamine biosynthesis protein ThiS [P...   
61   1e-08

gi|32444387|emb|CAD74386.1|
probable molybdopterin converting fa...   
61   1e-08

gi|75765030|ref|ZP\_00744342.1|
Molybdopterin converting factor, ...   
61   1e-08

gi|15074101|emb|CAC45748.1|
PROBABLE MOLYBDOPTERIN MPT CONVERTIN...   
61   1e-08

gi|86139074|ref|ZP\_01057645.1|
molybdopterin converting factor, ...   
61   1e-08

gi|88946143|ref|ZP\_01149231.1|
conserved hypothetical protein [D...   
61   1e-08

gi|77380988|gb|ABA72501.1|
Protein of unknown function UPF0125 [...   
61   1e-08

gi|56680191|gb|AAV96857.1|
molybdopterin converting factor, subu...   
61   1e-08

gi|91773385|ref|YP\_566077.1|
Molybdopterin converting factor sma...   
61   1e-08

gi|90422422|ref|YP\_530792.1|
molybdopterin converting factor, su...   
61   1e-08

gi|77917869|ref|YP\_355684.1|
hypothetical protein Pcar\_0253 [Pel...   
61   1e-08

gi|88934592|ref|ZP\_01140234.1|
conserved hypothetical protein [G...   
61   1e-08

gi|33152835|ref|NP\_874188.1|
hypothetical protein HD1828 [Haemop...   
61   1e-08

gi|70733136|ref|YP\_262909.1|
sulfur carrier protein ThiS [Pseudo...   
61   2e-08

gi|78219906|gb|ABB39255.1|
conserved hypothetical protein [Desul...   
61   2e-08

gi|86357115|ref|YP\_469007.1|
molybdopterin converting factor sub...   
61   2e-08

gi|14324783|dbj|BAB59710.1|
molybdopterin converting factor subu...   
61   2e-08

gi|77953016|ref|ZP\_00817427.1|
conserved hypothetical protein [M...   
60   2e-08

gi|104780007|ref|YP\_606505.1|
hypothetical protein PSEEN0772 [Ps...   
60   2e-08

gi|72118961|gb|AAZ61224.1|
Protein of unknown function UPF0125 [...   
60   2e-08

gi|71558661|gb|AAZ37872.1|
conserved hypothetical protein [Pseud...   
60   2e-08

gi|74018017|ref|ZP\_00688639.1|
ThiamineS [Burkholderia ambifaria...   
60   2e-08

gi|76874703|emb|CAI85924.1|
conserved protein of unknown functio...   
60   2e-08

gi|84354863|ref|ZP\_00979760.1|
COG1977: Molybdopterin converting...    60  
2e-08

gi|16413496|emb|CAC96268.1|
lin1037 [Listeria innocua] >gi|16800...   
60   2e-08

gi|40062746|gb|AAR37640.1|
molybdopterin converting factor, subu...   
60   2e-08

gi|21672525|ref|NP\_660592.1|
hypothetical protein BUsg244 [Buchn...   
60   3e-08

gi|10175640|dbj|BAB06737.1|
molybdopterin converting factor (sub...   
60   3e-08

gi|94494183|ref|ZP\_01301386.1|
hypothetical protein Rgryl\_010007...   
60   3e-08

gi|28854898|gb|AAO57960.1|
conserved hypothetical protein [Pseud...   
60   3e-08

gi|14325024|dbj|BAB59950.1|
molybdopterin converting factor subu...   
60   3e-08

gi|91797290|gb|ABE59429.1|
molybdenum cofactor biosynthesis prot...   
60   3e-08

gi|90416705|ref|ZP\_01224635.1|
molybdenum cofactor biosynthesis ...   
60   3e-08

gi|67927210|ref|ZP\_00520404.1|
Molybdopterin biosynthesis MoaE:T...   
60   3e-08

gi|76795290|ref|ZP\_00777663.1|
ThiamineS [Thermoanaerobacter eth...   
60   3e-08

gi|46156355|ref|ZP\_00204683.1|
COG1977: Molybdopterin converting...   
60   3e-08

gi|40063023|gb|AAR37879.1|
molybdenum cofactor biosynthesis prot...   
60   3e-08

gi|89359306|ref|ZP\_01197127.1|
Molybdopterin converting factor, ...   
60   3e-08

gi|67922943|ref|ZP\_00516439.1|
ThiamineS [Crocosphaera watsonii ...   
60   3e-08

gi|75704874|gb|ABA24550.1|
ThiamineS [Anabaena variabilis ATCC 2...   
60   3e-08

gi|46449006|gb|AAS95658.1|
hypothetical protein DVU\_1180 [Desulf...   
60   3e-08

gi|46108760|ref|XP\_381438.1|
hypothetical protein FG01262.1 [Gib...   
60   4e-08

gi|10038928|dbj|BAB12963.1|
hypothetical protein [Buchnera aphid...   
59   4e-08

gi|57638416|gb|AAW55204.1|
molybdopterin converting factor, subu...   
59   5e-08

gi|63258134|gb|AAY39230.1|
Protein of unknown function UPF0125 [...   
59   5e-08

gi|91785395|ref|YP\_560601.1|
Putative sulfur transfer protein in...   
59   5e-08

gi|71038167|gb|AAZ18475.1|
probable molybdopterin converting fac...   
59   5e-08

gi|22294706|dbj|BAC08535.1|
tsr0983 [Thermosynechococcus elongat...   
59   6e-08

gi|56604742|emb|CAG45817.1|
conserved hypothetical protein [Fran...   
59   6e-08

gi|68140833|gb|EAM94128.1|
MoaD, archaeal [Ferroplasma acidarman...   
59   6e-08

gi|54113521|gb|AAV29394.1|
NT02FT1312 [synthetic construct]           
59   6e-08

gi|77919319|ref|YP\_357134.1|
molybdopterin converting factor, su...    59  
6e-08

gi|29541871|gb|AAO90809.1|
conserved hypothetical protein [Coxie...   
59   7e-08

gi|88949835|ref|ZP\_01152449.1|
protein RnfH [Halorhodospira halo...   
59   7e-08

gi|21228746|ref|NP\_634668.1|
putative molybdopterin converting f...   
59   8e-08

gi|94417380|ref|ZP\_01297198.1|
hypothetical protein PaerP\_010006...   
59   8e-08

gi|71676258|ref|ZP\_00674000.1|
MoaD, archaeal [Trichodesmium ery...   
59   8e-08

gi|68542640|ref|ZP\_00582366.1|
Protein of unknown function UPF01...   
59   9e-08

gi|103486643|ref|YP\_616204.1|
molybdopterin converting factor, s...   
59   9e-08

gi|88934468|ref|ZP\_01140111.1|
conserved hypothetical protein [G...   
58   9e-08

gi|95929924|ref|ZP\_01312664.1|
thiamineS [Desulfuromonas acetoxi...   
58   1e-07

gi|68231908|ref|ZP\_00571067.1|
ThiamineS [Frankia sp. EAN1pec] >...   
58   1e-07

gi|92914605|ref|ZP\_01283231.1|
ThiamineS [Mycobacterium sp. KMS]...   
58   1e-07

gi|26991781|ref|NP\_747206.1|
sulfur carrier protein ThiS [Pseudo...   
58   1e-07

gi|69952904|ref|ZP\_00640232.1|
Protein of unknown function UPF01...   
58   1e-07

gi|78702657|ref|ZP\_00867087.1|
conserved hypothetical protein [A...   
58   1e-07

gi|9946232|gb|AAG03769.1|
conserved hypothetical protein [Pseudo...   
58   1e-07

gi|33359354|ref|NP\_877809.1|
putative molybdopterin converting f...   
58   1e-07

gi|90591660|ref|ZP\_01247301.1|
ThiamineS [Flavobacterium johnson...   
58   1e-07

gi|41406904|ref|NP\_959740.1|
MoaD2 [Mycobacterium avium subsp. p...   
58   1e-07

gi|77385555|gb|ABA77068.1|
ThiS, thiamine-biosynthesis [Pseudomo...   
58   1e-07

gi|5458384|emb|CAB49872.1|
moaD-like molybdopterin converting fa...   
58   1e-07

gi|86739580|ref|YP\_479980.1|
thiamine S [Frankia sp. CcI3] >gi|8...   
58   1e-07

gi|48430776|gb|AAT43641.1|
molybdopterin (MPT) converting factor...   
58   1e-07

gi|49088896|gb|AAT51619.1|
PA4766 [synthetic construct]               
58   1e-07

gi|78685307|ref|ZP\_00850086.1|
conserved hypothetical protein [S...   
58   1e-07

gi|9951030|gb|AAG08152.1|
conserved hypothetical protein [Pseudo...   
58   1e-07

gi|84717489|ref|ZP\_01023548.1|
similar to Uncharacterized protei...   
58   1e-07

gi|89901068|ref|YP\_523539.1|
protein of unknown function UPF0125...   
57   2e-07

gi|89256146|ref|YP\_513508.1|
hypothetical protein FTL\_0763 [Fran...   
57   2e-07

gi|84323578|ref|ZP\_00971647.1|
COG2914: Uncharacterized protein ...   
57   2e-07

gi|94414782|ref|ZP\_01294636.1|
hypothetical protein PaerP\_010033...   
57   2e-07

gi|68446507|dbj|BAE04091.1|
moaD [Staphylococcus haemolyticus JC...  
 57   2e-07

gi|94501723|ref|ZP\_01308237.1|
molybdopterin biosynthesis [Ocean...   
57   2e-07

gi|87120236|ref|ZP\_01076131.1|
hypothetical protein MED121\_08593...   
57   2e-07

gi|10640172|emb|CAC12024.1|
conserved hypothetical protein [Ther...   
57   2e-07

gi|82751858|ref|YP\_417599.1|
molybdopterin converting factor sma...   
57   2e-07

gi|68193704|gb|EAN08356.1|
Molybdopterin converting factor, subu...   
57   2e-07

gi|24373049|ref|NP\_717091.1|
hypothetical protein SO1475 [Shewan...   
57   2e-07

gi|77953948|ref|ZP\_00818350.1|
ThiS, thiamine-biosynthesis [Mari...   
57   2e-07

gi|47571785|ref|ZP\_00241834.1|
COG2914: Uncharacterized protein ...   
57   2e-07

gi|23129177|ref|ZP\_00111010.1|
COG1977: Molybdopterin converting...   
57   2e-07

gi|46907277|ref|YP\_013666.1|
molybdenum cofactor biosynthesis pr...   
57   2e-07

gi|16410447|emb|CAC99123.1|
lmo1045 [Listeria monocytogenes] >gi...   
57   2e-07

gi|53726413|ref|ZP\_00347571.1|
COG2914: Uncharacterized protein ...   
57   2e-07

gi|52003241|gb|AAU23183.1|
molybdopterin converting factor (subu...   
57   2e-07

gi|66811416|ref|XP\_639888.1|
hypothetical protein DDB0186363 [Di...   
57   2e-07

gi|88703397|ref|ZP\_01101113.1|
Molybdopterin converting factor s...   
57   2e-07

gi|88801656|ref|ZP\_01117184.1|
putative molybdopterin-synthase s...   
57   3e-07

gi|86143258|ref|ZP\_01061660.1|
hypothetical protein MED217\_08750...   
57   3e-07

gi|35213985|dbj|BAC91354.1|
gsl3413 [Gloeobacter violaceus PCC 7...   
57   3e-07

gi|71548925|ref|ZP\_00669149.1|
Protein of unknown function UPF01...   
57   3e-07

gi|16331655|ref|NP\_442383.1|
hypothetical protein ssr1527 [Synec...   
57   3e-07

gi|89339708|ref|ZP\_01192306.1|
ThiamineS [Mycobacterium flavesce...   
57   3e-07

gi|90203237|ref|ZP\_01205883.1|
ThiamineS [Mycobacterium vanbaale...   
57   3e-07

gi|47096132|ref|ZP\_00233732.1|
molybdenum cofactor biosynthesis ...   
57   3e-07

gi|72395205|gb|AAZ69478.1|
molybdopterin converting factor small...   
57   3e-07

gi|78195384|gb|ABB33151.1|
ThiS, thiamine-biosynthesis [Geobacte...   
57   3e-07

gi|49082326|gb|AAT50563.1|
PA0380 [synthetic construct]               
57   3e-07

gi|91792599|ref|YP\_562250.1|
protein of unknown function UPF0125...   
57   3e-07

gi|46200137|ref|YP\_005804.1|
molybdopterin converting factor, sm...    57  
3e-07

gi|77964628|gb|ABB06009.1|
Thiamine S [Burkholderia sp. 383] >gi...   
56   3e-07

gi|73661952|ref|YP\_300733.1|
molybdopterin converting factor sma...   
56   4e-07

gi|88196185|ref|YP\_501002.1|
molybdopterin converting factor, su...   
56   4e-07

gi|55231494|gb|AAV46913.1|
molybdopterin converting factor subun...   
56   4e-07

gi|81300590|ref|YP\_400798.1|
hypothetical protein Synpcc7942\_178...   
56   4e-07

gi|13880985|gb|AAK45641.1|
conserved hypothetical protein [Mycob...   
56   4e-07

gi|95926545|ref|ZP\_01309328.1|
hypothetical protein CburR\_010016...   
56   4e-07

gi|13880442|gb|AAK45132.1|
conserved hypothetical protein [Mycob...   
56   4e-07

gi|78047114|ref|YP\_363289.1|
hypothetical protein XCV1558 [Xanth...   
56   4e-07

gi|57340004|gb|AAW49989.1|
hypothetical protein FTT1184 [synthet...   
56   5e-07

gi|78193517|gb|ABB31284.1|
Thiamine S [Geobacter metallireducens...   
56   5e-07

gi|55771533|dbj|BAD69974.1|
molybdopterin converting factor, sub...   
56   5e-07

gi|91204446|emb|CAJ70946.1|
similar to molybdopterin synthase su...   
56   5e-07

gi|3955207|gb|AAC83142.1|
MoaD [Staphylococcus carnosus]              
56   5e-07

gi|21221358|ref|NP\_627137.1|
hypothetical protein SCO2911 [Strep...   
56   5e-07

gi|84623563|ref|YP\_450935.1|
hypothetical protein XOO\_1906 [Xant...   
56   5e-07

gi|49484485|ref|YP\_041709.1|
putative molybdopterin-synthase sma...   
56   5e-07

gi|77814255|ref|ZP\_00813518.1|
Protein of unknown function UPF01...   
56   5e-07

gi|98659923|dbj|GAA02748.1|
unnamed protein product [Pelotomacul...   
56   5e-07

gi|88712833|ref|ZP\_01106918.1|
hypothetical protein FB2170\_09351...   
56   6e-07

gi|67937987|ref|ZP\_00530517.1|
ThiamineS [Chlorobium phaeobacter...   
55   6e-07

gi|89091960|ref|ZP\_01164915.1|
hypothetical protein MED92\_07331 ...   
55   6e-07

gi|71366890|ref|ZP\_00657425.1|
ThiamineS [Nocardioides sp. JS614...   
55   6e-07

gi|78219002|gb|ABB38351.1|
thiamine biosynthesis protein ThiS [D...   
55   6e-07

gi|19915596|gb|AAM05120.1|
predicted protein [Methanosarcina ace...   
55   7e-07

gi|86134373|ref|ZP\_01052955.1|
putative molybdopterin-synthase s...   
55   7e-07

gi|50877366|emb|CAG37206.1|
similar to molybdopterin converting ...   
55   7e-07

gi|34483098|emb|CAE10097.1|
hypothetical protein [Wolinella succ...  
 55   8e-07

gi|74318145|ref|YP\_315885.1|
thiamine-biosynthesis protein ThiS ...   
55   8e-07

gi|42543908|pdb|1V8C|D
Chain D, Crystal Structure Of Moad Relate...   
55   8e-07

gi|107024240|ref|YP\_622567.1|
thiamine biosynthesis protein ThiS...   
55   8e-07

gi|63258672|gb|AAY39768.1|
ThiS, thiamine-biosynthesis [Pseudomo...   
55   8e-07

gi|27904127|gb|AAO26961.1|
hypothetical protein bbp\_234 [Buchner...   
55   9e-07

gi|14025577|dbj|BAB52177.1|
thiamin biosynthesis; ThiG [Mesorhiz...   
55   9e-07

gi|6968941|emb|CAB73937.1|
possible molybdopterin converting fac...   
55   1e-06

gi|86604898|ref|YP\_473661.1|
putative molybdopterin converting f...   
55   1e-06

gi|57167344|gb|AAW36123.1|
thiS family protein [Campylobacter je...   
55   1e-06

gi|90416962|ref|ZP\_01224891.1|
hypothetical protein GB2207\_06863...   
55   1e-06

gi|78698313|ref|ZP\_00862816.1|
ThiS, thiamine-biosynthesis [Brad...   
55   1e-06

gi|84690418|gb|EAQ16259.1|
thiazole biosynthesis protein [Parvul...   
55   1e-06

gi|95930902|ref|ZP\_01313632.1|
thiamineS [Desulfuromonas acetoxi...   
55   1e-06

gi|84360622|ref|ZP\_00985320.1|
COG2104: Sulfur transfer protein ...   
55   1e-06

gi|21112537|gb|AAM40764.1|
conserved hypothetical protein [Xanth...   
55   1e-06

gi|4155307|gb|AAD06323.1|
putative MOLYBDOPTERIN CONVERTING FACT...   
54   1e-06

gi|67664217|ref|ZP\_00461493.1|
ThiamineS [Burkholderia cenocepac...   
54   1e-06

gi|28850898|gb|AAO53977.1|
thiamine biosynthesis protein ThiS [P...   
54   2e-06

gi|76801893|ref|YP\_326901.1|
probable molybdopterin converting f...   
54   2e-06

gi|30250278|ref|NP\_842348.1|
DUF170 [Nitrosomonas europaea ATCC ...   
54   2e-06

gi|71838567|ref|ZP\_00678326.1|
ThiamineS [Pelobacter propionicus...   
54   2e-06

gi|74019492|ref|ZP\_00690108.1|
ThiS, thiamine-biosynthesis [Burk...   
54   2e-06

gi|94968345|ref|YP\_590393.1|
thiamine biosynthesis protein ThiS ...   
54   2e-06

gi|82546334|ref|YP\_410281.1|
Sulfur transfer protein [Shigella b...   
54   2e-06

gi|83645616|ref|YP\_434051.1|
Molybdopterin converting factor, sm...   
54   2e-06

gi|83720274|ref|YP\_443511.1|
thiamine biosynthesis protein ThiS ...   
54   2e-06

gi|58581648|ref|YP\_200664.1|
hypothetical protein XOO2025 [Xanth...   
54   2e-06

gi|16767415|ref|NP\_463030.1|
sulfur carrier protein ThiS [Salmon...   
54   2e-06

gi|86154746|ref|ZP\_01072913.1|
conserved domain protein [Campylo...   
54   2e-06

gi|82778833|ref|YP\_405182.1|
Sulfur transfer protein involved in...   
54   2e-06

gi|86605752|ref|YP\_474515.1|
thiamine biosynthesis protein ThiS ...    54  
2e-06

gi|15621527|dbj|BAB65522.1|
236aa long hypothetical molybdopteri...   
54   2e-06

gi|83748716|ref|ZP\_00945732.1|
ThiS family protein [Ralstonia so...   
54   2e-06

gi|92118391|ref|YP\_578120.1|
thiamine biosynthesis protein ThiS ...   
54   2e-06

gi|33565867|emb|CAE36414.1|
molybdopterin converting factor [Bor...   
54   2e-06

gi|29830476|ref|NP\_825110.1|
hypothetical protein SAV3933 [Strep...   
54   2e-06

gi|91785509|ref|YP\_560715.1|
hypothetical protein Bxe\_A0270 [Bur...   
54   2e-06

gi|88805648|ref|ZP\_01121167.1|
hypothetical protein RB2501\_14949...   
54   2e-06

gi|77965731|gb|ABB07111.1|
ThiS, thiamine-biosynthesis [Burkhold...   
54   2e-06

gi|67154907|ref|ZP\_00416652.1|
ThiS, thiamine-biosynthesis [Azot...   
54   2e-06

gi|75255488|ref|ZP\_00727287.1|
COG2104: Sulfur transfer protein ...   
54   2e-06

gi|77960648|ref|ZP\_00824510.1|
COG2104: Sulfur transfer protein ...   
54   3e-06

gi|70606737|ref|YP\_255607.1|
molybdenum cofactor biosynthesis pr...   
54   3e-06

gi|21226933|ref|NP\_632855.1|
Molybdopterin converting factor sma...   
54   3e-06

gi|84705083|ref|ZP\_01018583.1|
molybdopterin converting factor, ...   
54   3e-06

gi|53804598|ref|YP\_113552.1|
thiazole synthase [Methylococcus ca...   
53   3e-06

gi|21222687|ref|NP\_628466.1|
hypothetical protein SCO4294 [Strep...   
53   3e-06

gi|91798352|gb|ABE60491.1|
thiamine biosynthesis protein ThiS [C...   
53   3e-06

gi|85715979|ref|ZP\_01046956.1|
ThiS, thiamine-biosynthesis [Nitr...   
53   3e-06

gi|50839587|gb|AAT82254.1|
putative molybdopterin converting fac...   
53   3e-06

gi|77957008|ref|ZP\_00821077.1|
COG2104: Sulfur transfer protein ...   
53   3e-06

gi|48994994|gb|AAT48237.1|
sulphur carrier protein [Escherichia ...   
53   3e-06

gi|87306448|ref|ZP\_01088595.1|
thiamine biosynthesis protein Thi...   
53   3e-06

gi|90303795|gb|EAS33426.1|
hypothetical protein CIMG\_04450 [Cocc...   
53   3e-06

gi|72160631|ref|YP\_288288.1|
hypothetical protein Tfu\_0227 [Ther...   
53   3e-06

gi|74314485|ref|YP\_312904.1|
Sulfur transfer protein [Shigella s...   
53   4e-06

gi|86609832|ref|YP\_478594.1|
thiamine biosynthesis protein ThiS ...   
53   4e-06

gi|91788303|ref|YP\_549255.1|
protein of unknown function UPF0125...   
53   4e-06

gi|52427701|gb|AAU48294.1|
thiamine biosynthesis protein ThiS, p...  
 53   4e-06

gi|9105678|gb|AAF83592.1|
conserved hypothetical protein [Xylell...   
53   4e-06

gi|91212802|ref|YP\_542788.1|
hypothetical protein UTI89\_C3828 [E...   
53   4e-06

gi|94676639|ref|YP\_588996.1|
hypothetical protein BCI\_0567 [Baum...   
53   4e-06

gi|85707987|ref|ZP\_01039053.1|
hypothetical protein NAP1\_02090 [...   
53   5e-06

gi|67904312|ref|XP\_682412.1|
hypothetical protein AN9143.2 [Aspe...   
52   5e-06

gi|74421519|gb|ABA05718.1|
ThiS, thiamine-biosynthesis [Nitrobac...   
52   5e-06

gi|39964901|ref|XP\_365048.1|
hypothetical protein MG09893.4 [Mag...   
52   5e-06

gi|33563747|emb|CAE42987.1|
molybdopterin converting factor [Bor...   
52   5e-06

gi|89343138|ref|ZP\_01195366.1|
ThiamineS [Mycobacterium flavesce...   
52   5e-06

gi|33567914|emb|CAE31827.1|
molybdopterin converting factor [Bor...   
52   5e-06

gi|16762296|ref|NP\_457913.1|
sulfur carrier protein ThiS [Salmon...   
52   5e-06

gi|86149491|ref|ZP\_01067722.1|
thiS family protein [Campylobacte...   
52   6e-06

gi|87303475|ref|ZP\_01086258.1|
hypothetical protein WH5701\_09450...   
52   6e-06

gi|89901619|ref|YP\_524090.1|
thiamineS [Rhodoferax ferrireducens...   
52   6e-06

gi|104779587|ref|YP\_606085.1|
thiamine biosynthesis protein ThiS...   
52   6e-06

gi|18161603|gb|AAL64874.1|
conserved hypothetical protein [Pyrob...   
52   6e-06

gi|33564627|emb|CAE43947.1|
conserved hypothetical protein [Bord...   
52   6e-06

gi|88608780|ref|YP\_505946.1|
thiamine biosynthesis protein ThiS ...   
52   7e-06

gi|12518920|gb|AAG59188.1|
thiamin biosynthesis, probable sulfur...   
52   7e-06

gi|71836234|ref|ZP\_00676003.1|
ThiS, thiamine-biosynthesis [Pelo...   
52   7e-06

gi|86749080|ref|YP\_485576.1|
thiamine biosynthesis protein ThiS ...   
52   7e-06

gi|67154055|ref|ZP\_00415800.1|
Protein of unknown function DUF82...   
52   8e-06

gi|17130691|dbj|BAB73301.1|
asr1344 [Nostoc sp. PCC 7120] >gi|17...   
52   8e-06

gi|77918224|ref|YP\_356039.1|
thiamine biosynthesis protein ThiS ...   
52   8e-06

gi|86153122|ref|ZP\_01071327.1|
conserved domain protein [Campylo...   
52   8e-06

gi|90022383|ref|YP\_528210.1|
hypothetical protein Sde\_2738 [Sacc...   
52   8e-06

gi|26111193|gb|AAN83376.1|
Thiamin biosynthesis, probable sulfur...   
52   8e-06

gi|18160982|gb|AAL64296.1|
conserved hypothetical protein [Pyrob...  
 52   8e-06

gi|99908378|ref|ZP\_01316082.1|
hypothetical protein Bpse1\_030045...   
52   8e-06

gi|75236721|ref|ZP\_00720794.1|
COG2104: Sulfur transfer protein ...   
52   9e-06

gi|77919190|ref|YP\_357005.1|
hypothetical protein Pcar\_1591 [Pel...   
52   9e-06

gi|36783916|emb|CAE12778.1|
ThiS protein [Photorhabdus luminesce...   
52   9e-06

gi|21107692|gb|AAM36384.1|
conserved hypothetical protein [Xanth...   
52   9e-06

gi|85373384|ref|YP\_457446.1|
hypothetical protein ELI\_02785 [Ery...   
52   9e-06

gi|11933033|emb|CAC19353.1|
hypothetical protein [Streptomyces h...   
52   9e-06

gi|76580804|gb|ABA50279.1|
Protein of unknown function family [B...   
52   1e-05

gi|68055198|ref|ZP\_00539345.1|
ThiS, thiamine-biosynthesis [Exig...   
52   1e-05

gi|52627727|gb|AAU26468.1|
hypothetical protein lpg0371 [Legione...   
52   1e-05

gi|34483282|emb|CAE10280.1|
hypothetical protein [Wolinella succ...   
52   1e-05

gi|67738583|ref|ZP\_00489224.1|
COG1656: Uncharacterized conserve...   
52   1e-05

gi|62425453|ref|ZP\_00380587.1|
COG1977: Molybdopterin converting...   
52   1e-05

gi|77997120|gb|ABB16019.1|
conserved hypothetical protein [Carbo...   
51   1e-05

gi|38704219|ref|NP\_944578.1|
sulfur carrier protein ThiS [Escher...   
51   1e-05

gi|107022979|ref|YP\_621306.1|
thiamineS [Burkholderia cenocepaci...   
51   1e-05

gi|99910773|ref|ZP\_01317738.1|
hypothetical protein Bpse1\_030028...   
51   1e-05

gi|49609716|emb|CAG73149.1|
thiamine biosynthesis protein [Erwin...   
51   1e-05

gi|89092458|ref|ZP\_01165412.1|
sulfur carrier protein ThiS [Ocea...   
51   1e-05

gi|33286618|gb|AAQ01711.1|
PdtH [Pseudomonas putida]                  
51   1e-05

gi|88949122|ref|ZP\_01151740.1|
ThiS, thiamine-biosynthesis [Halo...   
51   1e-05

gi|57505249|ref|ZP\_00371178.1|
moaD2 [Campylobacter upsaliensis ...   
51   1e-05

gi|13815697|gb|AAK42541.1|
Molybdenum cofactor biosynthesis prot...   
51   1e-05

gi|71898215|ref|ZP\_00680389.1|
ThiS, thiamine-biosynthesis [Xyle...   
51   2e-05

gi|76803138|ref|YP\_331233.1|
probable molybdopterin converting f...   
51   2e-05

gi|98662976|dbj|GAA00218.1|
unnamed protein product [Pelotomacul...   
51   2e-05

gi|107024344|ref|YP\_622671.1|
protein of unknown function DUF82 ...   
51   2e-05

gi|71900707|ref|ZP\_00682830.1|
ThiS, thiamine-biosynthesis [Xyle...   
51   2e-05

gi|71544347|ref|ZP\_00665385.1|
ThiamineS [Syntrophobacter fumaro...   
51   2e-05

gi|32261593|gb|AAP76643.1|
molybdopterin converting factor [Heli...   
51   2e-05

gi|32262239|gb|AAP77287.1|
conserved hypothetical protein [Helic...  
 51   2e-05

gi|88931544|ref|ZP\_01137238.1|
putative molybdopterin converting...   
51   2e-05

gi|86739160|ref|YP\_479560.1|
thiamine S [Frankia sp. CcI3] >gi|8...   
50   2e-05

gi|89338808|ref|ZP\_01191573.1|
ThiS, thiamine-biosynthesis [Myco...   
50   2e-05

gi|83858499|ref|ZP\_00952021.1|
thiazole biosynthesis protein [Oc...   
50   2e-05

gi|84716935|ref|ZP\_01023301.1|
hypothetical protein PnapDRAFT\_04...   
50   2e-05

gi|76883424|gb|ABA58105.1|
ThiS, thiamine-biosynthesis [Nitrosoc...   
50   2e-05

gi|106891734|ref|ZP\_01358916.1|
ThiamineS [Roseiflexus sp. RS-1]...   
50   2e-05

gi|67666690|ref|ZP\_00463933.1|
Protein of unknown function DUF82...   
50   2e-05

gi|78700359|ref|ZP\_00864816.1|
Rhodanese-like [Alkalilimnicola e...   
50   2e-05

gi|54014565|dbj|BAD55935.1|
hypothetical protein [Nocardia farci...   
50   2e-05

gi|5070641|gb|AAD39227.1| MoaD-like protein [Pseudomonas stutzer...    50  
2e-05

gi|90581019|ref|ZP\_01236819.1|
sulfur carrier protein ThiS [Vibr...   
50   2e-05

gi|23130347|ref|ZP\_00112164.1|
COG2104: Sulfur transfer protein ...   
50   2e-05

gi|82702099|ref|YP\_411665.1|
ThiamineS [Nitrosospira multiformis...   
50   2e-05

gi|86137736|ref|ZP\_01056312.1|
hypothetical protein MED193\_07738...   
50   2e-05

gi|91977895|ref|YP\_570554.1|
thiamine biosynthesis protein ThiS ...   
50   2e-05

gi|28192390|gb|AAL65287.1|
QbsE [Pseudomonas fluorescens]             
50   2e-05

gi|45437725|gb|AAS63275.1|
Sulfur transfer protein involved in t...   
50   2e-05

gi|39936636|ref|NP\_948912.1|
putative thiamin biosynthesis ThiS ...   
50   2e-05

gi|21322979|dbj|BAB97608.1|
Hypothetical protein [Corynebacteriu...   
50   2e-05

gi|13421105|gb|AAK22005.1|
molybdopterin converting factor, subu...   
50   2e-05

gi|17983762|gb|AAL52915.1|
hypothetical protein [Brucella melite...   
50   2e-05

gi|51587924|emb|CAH19527.1|
thiamin biosynthesis ThiS [Yersinia ...   
50   3e-05

gi|74318176|ref|YP\_315916.1|
hypothetical protein Tbd\_2158 [Thio...   
50   3e-05

gi|78048972|ref|YP\_365147.1|
hypothetical protein XCV3416 [Xanth...   
50   3e-05

gi|53753195|emb|CAH14642.1|
hypothetical protein [Legionella pne...   
50   3e-05

gi|27381767|ref|NP\_773296.1|
thiamin biosynthesis protein homolo...   
50   3e-05

gi|82701206|ref|YP\_410772.1|
thiamine biosynthesis protein ThiS ...    50  
3e-05

gi|47571794|ref|ZP\_00241843.1|
COG2104: Sulfur transfer protein ...   
50   3e-05

gi|92906590|ref|ZP\_01275370.1|
ThiamineS [Mycobacterium sp. JLS]...   
50   3e-05

gi|90423668|ref|YP\_532038.1|
thiamine biosynthesis protein ThiS ...   
50   3e-05

gi|71369077|ref|ZP\_00659547.1|
ThiamineS [Nocardioides sp. JS614...   
50   3e-05

gi|75760851|ref|ZP\_00740866.1|
Molybdopterin converting factor, ...   
50   3e-05

gi|71909473|ref|YP\_287060.1|
ThiS, thiamine-biosynthesis [Dechlo...   
50   4e-05

gi|92914564|ref|ZP\_01283191.1|
ThiamineS [Mycobacterium sp. KMS]...   
50   4e-05

gi|77995330|gb|ABB14229.1|
thiamine biosynthesis protein ThiS [C...   
50   4e-05

gi|54017794|dbj|BAD59164.1|
hypothetical protein [Nocardia farci...   
50   4e-05

gi|56130244|gb|AAV79750.1|
thiamine biosynthesis protein [Salmon...   
50   4e-05

gi|71898356|ref|ZP\_00680529.1|
Protein of unknown function UPF01...   
50   4e-05

gi|77655361|gb|EAO87002.1|
ThiS, thiamine-biosynthesis [Rhodopse...   
50   4e-05

gi|83719003|ref|YP\_443618.1|
Protein of unknown function family ...   
50   4e-05

gi|21109644|gb|AAM38144.1|
conserved hypothetical protein [Xanth...   
50   4e-05

gi|55240673|gb|EAL40503.1|
ENSANGP00000025406 [Anopheles gambiae...   
50   4e-05

gi|89103189|ref|ZP\_01175778.1|
COG2104: Sulfur transfer protein ...   
50   4e-05

gi|19918186|gb|AAM07434.1|
molybdopterin converting factor, subu...   
50   4e-05

gi|28199747|ref|NP\_780061.1|
hypothetical protein PD1878 [Xylell...   
50   4e-05

gi|76258732|ref|ZP\_00766386.1|
ThiamineS [Chloroflexus aurantiac...   
49   5e-05

gi|78362777|gb|ABB40742.1|
thiamine biosynthesis protein ThiS [T...   
49   5e-05

gi|88186128|gb|EAQ93596.1|
hypothetical protein CHGG\_01831 [Chae...   
49   5e-05

gi|51857712|dbj|BAD41870.1|
molybdopterin converting factor smal...   
49   5e-05

gi|98661971|dbj|GAA01064.1|
unnamed protein product [Pelotomacul...   
49   5e-05

gi|11499688|ref|NP\_070930.1|
hypothetical protein AF2105 [Archae...   
49   5e-05

gi|41324446|emb|CAF18786.1|
PUTATIVE MOLYBDOPTERIN CONVERTING FA...   
49   5e-05

gi|83952602|ref|ZP\_00961333.1|
thiamine biosynthesis protein Thi...   
49   5e-05

gi|56314521|emb|CAI09166.1|
putative tungsten-containing aldehyd...   
49   6e-05

gi|85709842|ref|ZP\_01040907.1|
thiazole biosynthesis protein [Er...    49  
6e-05

gi|88936313|ref|ZP\_01141926.1|
ThiS, thiamine-biosynthesis [Geob...   
49   6e-05

gi|71550737|ref|ZP\_00670826.1|
ThiS, thiamine-biosynthesis [Nitr...   
49   6e-05

gi|23492025|dbj|BAC17000.1|
putative molybdopterin biosynthesis ...   
49   6e-05

gi|67930072|ref|ZP\_00523248.1|
ThiS, thiamine-biosynthesis [Soli...   
49   6e-05

gi|71900376|ref|ZP\_00682510.1|
Protein of unknown function UPF01...   
49   6e-05

gi|29831706|ref|NP\_826340.1|
hypothetical protein SAV5163 [Strep...   
49   7e-05

gi|57240883|ref|ZP\_00368831.1|
molybdopterin converting factor, ...   
49   7e-05

gi|83311801|ref|YP\_422065.1|
Uncharacterized enzyme of thiazole ...   
49   7e-05

gi|81299867|ref|YP\_400075.1|
thiamine biosynthesis protein ThiS ...   
48   7e-05

gi|85666193|ref|ZP\_01028417.1|
hypothetical protein Badol\_010010...   
48   7e-05

gi|58582994|ref|YP\_202010.1|
hypothetical protein XOO3371 [Xanth...   
48   8e-05

gi|67932283|ref|ZP\_00525430.1|
ThiamineS [Solibacter usitatus El...   
48   8e-05

gi|56543208|gb|AAV89362.1|
thiazole biosynthesis protein [Zymomo...   
48   8e-05

gi|78701988|ref|ZP\_00866433.1|
ThiS, thiamine-biosynthesis [Alka...   
48   9e-05

gi|90205897|ref|ZP\_01208535.1|
ThiS, thiamine-biosynthesis [Myco...   
48   9e-05

gi|17554136|ref|NP\_498102.1|
K10D2.7 [Caenorhabditis elegans] >g...   
48   1e-04

gi|78700231|ref|ZP\_00864688.1|
hypothetical protein MlgDRAFT\_272...   
48   1e-04

gi|23346995|gb|AAN29165.1|
thiamine biosynthesis protein ThiS [B...   
48   1e-04

gi|21220586|ref|NP\_626365.1|
hypothetical protein SCO2108 [Strep...   
48   1e-04

gi|13423328|gb|AAK23856.1|
conserved hypothetical protein [Caulo...   
48   1e-04

gi|29832636|ref|NP\_827270.1| sulfur transfer protein involved in...    48  
1e-04

gi|87311482|ref|ZP\_01093601.1| hypothetical protein DSM3645\_0176...    48  
1e-04

gi|91793129|ref|YP\_562780.1| thiamine biosynthesis protein ThiS ...    48  
1e-04

gi|10581293|gb|AAG20053.1| Vng1848h [Halobacterium sp. NRC-1] >g...   
48   1e-04

gi|89074422|ref|ZP\_01160899.1|
sulfur carrier protein ThiS [Phot...   
48   1e-04

gi|86607092|ref|YP\_475855.1|
ThiS domain protein [Synechococcus ...   
48   1e-04

gi|89207101|ref|ZP\_01185649.1|
ThiS, thiamine-biosynthesis [Baci...   
48   2e-04

gi|84498430|ref|ZP\_00997200.1|
putative molybdopterin converting...   
48   2e-04

gi|77972245|ref|ZP\_00827817.1|
COG2104: Sulfur transfer protein ...   
47   2e-04

gi|68563154|ref|ZP\_00602356.1|
ThiamineS [Rubrobacter xylanophil...   
47   2e-04

gi|85705982|ref|ZP\_01037078.1|
thiamine biosynthesis protein Thi...    47  
2e-04

gi|46201345|ref|ZP\_00055205.2|
COG1977: Molybdopterin converting...   
47   2e-04

gi|69300050|ref|ZP\_00621926.1|
ThiS, thiamine-biosynthesis [Sili...   
47   2e-04

gi|87125482|ref|ZP\_01081327.1|
hypothetical protein RS9917\_01876...   
47   2e-04

gi|88946144|ref|ZP\_01149232.1|
conserved hypothetical protein [D...   
47   2e-04

gi|49238090|emb|CAF27297.1|
Thiamin biosynthesis, thiG1 [Bartone...   
47   2e-04

gi|45047786|emb|CAF30913.1|
ThiamineS [Methanococcus maripaludis...   
47   2e-04

gi|78497576|gb|ABB44116.1|
ThiS, thiamine-biosynthesis [Thiomicr...   
47   2e-04

gi|90201062|ref|ZP\_01203708.1|
ThiamineS [Mycobacterium vanbaale...   
47   2e-04

gi|77977808|ref|ZP\_00833248.1|
COG2104: Sulfur transfer protein ...   
47   2e-04

gi|33576043|emb|CAE33122.1|
conserved hypothetical protein [Bord...   
47   2e-04

gi|47501163|gb|AAT29839.1|
conserved hypothetical protein [Bacil...   
47   2e-04

gi|99078622|ref|YP\_611880.1|
thiamine biosynthesis protein ThiS ...   
47   2e-04

gi|93453080|gb|EAT03559.1|
ThiS, thiamine-biosynthesis [delta pr...   
47   2e-04

gi|51856879|dbj|BAD41037.1|
conserved hypothetical protein [Symb...   
47   2e-04

gi|39982460|gb|AAR33920.1|
conserved hypothetical protein [Geoba...   
47   2e-04

gi|88932583|ref|ZP\_01138273.1|
ThiS, thiamine-biosynthesis [Acid...   
47   2e-04

gi|89101139|ref|ZP\_01173973.1|
sulfur carrier protein ThiS [Baci...   
47   2e-04

gi|78167320|gb|ABB24418.1|
ThiS, thiamine-biosynthesis [Pelodict...   
47   2e-04

gi|32034642|ref|ZP\_00134789.1|
COG2104: Sulfur transfer protein ...   
47   3e-04

gi|65318157|ref|ZP\_00391116.1|
COG2104: Sulfur transfer protein ...   
47   3e-04

gi|56311907|emb|CAI06552.1|
conserved hypothetical protein [Azoa...   
47   3e-04

gi|89203238|ref|ZP\_01181926.1|
ThiS, thiamine-biosynthesis [Baci...   
47   3e-04

gi|91798304|gb|ABE60443.1|
protein of unknown function UPF0125 [...   
47   3e-04

gi|92909569|ref|ZP\_01278346.1|
ThiS, thiamine-biosynthesis [Myco...   
47   3e-04

gi|71545061|ref|ZP\_00666026.1|
ThiS, thiamine-biosynthesis [Synt...   
47   3e-04

gi|78171431|gb|ABB28527.1|
ThiS, thiamine-biosynthesis [Chlorobi...   
47   3e-04

gi|84517612|ref|ZP\_01004961.1|
Sulfur transfer protein [Prochlor...   
47   3e-04

gi|86609522|ref|YP\_478284.1|
ThiS domain protein [Synechococcus ...    47  
3e-04

gi|29894449|gb|AAP07739.1|
ThiS protein [Bacillus cereus ATCC 14...   
47   3e-04

gi|87201302|ref|YP\_498559.1|
thiamine biosynthesis protein ThiS ...   
46   3e-04

gi|35210963|dbj|BAC88343.1|
ycf40 [Gloeobacter violaceus PCC 742...   
46   4e-04

gi|90412774|ref|ZP\_01220775.1|
hypothetical protein P3TCK\_22740 ...   
46   4e-04

gi|85117520|ref|XP\_965278.1|
hypothetical protein [Neurospora cr...   
46   4e-04

gi|92916053|ref|ZP\_01284674.1|
ThiS, thiamine-biosynthesis [Myco...   
46   4e-04

gi|68192288|gb|EAN06942.1|
ThiS, thiamine-biosynthesis [Mesorhiz...   
46   4e-04

gi|83593767|ref|YP\_427519.1|
ThiS, thiamine-biosynthesis [Rhodos...   
46   4e-04

gi|6226383|sp|O29699|Y552\_ARCFU
Hypothetical UPF0084 protein AF0...   
46   4e-04

gi|72163135|ref|YP\_290792.1|
putative molybdopterin converting f...   
46   4e-04

gi|47572157|ref|ZP\_00242203.1|
COG1977: Molybdopterin converting...   
46   4e-04

gi|21646638|gb|AAM71936.1|
thiamine biosynthesis protein ThiS [C...   
46   4e-04

gi|34499222|ref|NP\_903437.1|
hypothetical protein CV3767 [Chromo...   
46   5e-04

gi|88811204|ref|ZP\_01126460.1|
hypothetical protein NB231\_10388 ...   
46   5e-04

gi|56379001|dbj|BAD74909.1|
hypothetical conserved protein [Geob...   
46   5e-04

gi|91787516|ref|YP\_548468.1|
protein of unknown function DUF82 [...   
46   5e-04

gi|49331838|gb|AAT62484.1|
thiamine biosynthesis protein [Bacill...   
46   5e-04

gi|77741142|ref|ZP\_00809626.1|
ThiS, thiamine-biosynthesis [Rhod...   
46   5e-04

gi|49530092|emb|CAG67804.1|
conserved hypothetical protein [Acin...   
46   5e-04

gi|53751264|emb|CAH12675.1|
hypothetical protein [Legionella pne...   
46   5e-04

gi|95930338|ref|ZP\_01313075.1|
thiamine biosynthesis protein Thi...   
46   5e-04

gi|82701281|ref|YP\_410847.1|
Protein of unknown function DUF82 [...   
46   5e-04

gi|56178887|gb|AAV81609.1|
Thiamine biosynthesis protein ThiS [I...   
46   5e-04

gi|67922608|ref|ZP\_00516114.1|
ThiS, thiamine-biosynthesis [Croc...   
46   5e-04

gi|68561643|ref|ZP\_00600934.1|
ThiS, thiamine-biosynthesis [Rubr...   
46   6e-04

gi|70982492|ref|XP\_746774.1|
molybdopterin synthase small subuni...   
45   6e-04

gi|67919394|ref|ZP\_00512973.1|
ThiS, thiamine-biosynthesis [Chlo...   
45   7e-04

gi|91776986|ref|YP\_546742.1|
thiamine biosynthesis protein ThiS ...    45  
7e-04

gi|68246504|gb|EAN28602.1|
ThiS, thiamine-biosynthesis [Magnetoc...   
45   7e-04

gi|77965627|gb|ABB07007.1|
protein of unknown function DUF82 [Bu...   
45   8e-04

gi|94309119|ref|YP\_582329.1|
protein of unknown function DUF82 [...   
45   8e-04

gi|83773325|dbj|BAE63452.1|
unnamed protein product [Aspergillus...   
45   8e-04

gi|88607118|ref|YP\_504702.1|
thiamine biosynthesis protein ThiS ...   
45   8e-04

gi|94495482|ref|ZP\_01302062.1|
thiamine biosynthesis protein Thi...   
45   8e-04

gi|68550360|ref|ZP\_00589811.1|
ThiS, thiamine-biosynthesis [Pelo...   
45   8e-04

gi|88947032|ref|ZP\_01150061.1|
ThiS, thiamine-biosynthesis [Desu...   
45   8e-04

gi|83751114|ref|ZP\_00947528.1|
COG2104: Sulfur transfer protein ...   
45   8e-04

gi|91203815|emb|CAJ71468.1|
similar to thiS (Thiamine biosynthes...   
45   8e-04

gi|77683436|ref|ZP\_00798885.1|
ThiS, thiamine-biosynthesis [Alka...   
45   9e-04

gi|89361078|ref|ZP\_01198894.1|
conserved hypothetical protein [X...    45   9e-04

gi|27360519|gb|AAO09455.1| Sulfur transfer protein involved in t...    45  
9e-04

gi|33359535|ref|NP\_578790.2|
sulfur carrier protein ThiS [Pyroco...   
45   0.001

gi|30248304|ref|NP\_840374.1|
DUF170 [Nitrosomonas europaea ATCC ...   
45   0.001

gi|48425680|pdb|1SF0|A
Chain A, Backbone Solution Structure Of M...   
45   0.001

gi|86148246|ref|ZP\_01066542.1|
sulfur carrier protein ThiS [Vibr...   
45   0.001

gi|72117336|gb|AAZ59599.1|
Protein of unknown function DUF82 [Ra...   
45   0.001

gi|67935886|ref|ZP\_00528902.1|
ThiS, thiamine-biosynthesis [Chlo...   
45   0.001

gi|83589575|ref|YP\_429584.1|
MoaD [Moorella thermoacetica ATCC 3...   
45   0.001

gi|71481492|ref|ZP\_00661198.1|
ThiS, thiamine-biosynthesis [Pros...   
45   0.001

gi|18893126|gb|AAL81185.1|
hypothetical protein [Pyrococcus furi...   
45   0.001

gi|71546139|ref|ZP\_00666991.1|
conserved hypothetical protein [S...   
45   0.001

gi|52216684|dbj|BAD49277.1|
ThiS protein involved in thiamine bi...   
45   0.001

gi|82499654|ref|ZP\_00885094.1|
ThiS, thiamine-biosynthesis [Cald...   
45   0.001

gi|33634551|emb|CAE20537.1|
DUF170 [Prochlorococcus marinus str....   
45   0.001

gi|33572188|emb|CAE41734.1|
conserved hypothetical protein [Bord...   
45   0.001

gi|49239551|emb|CAF25908.1|
Thiamin biosynthesis, thiG1 [Bartone...   
45   0.001

gi|29830583|ref|NP\_825217.1|
molybdopterin converting factor [St...   
44   0.001

gi|56909742|dbj|BAD64269.1|
thiamine biosynthesis protein ThiS [...   
44   0.001

gi|88951090|ref|ZP\_01153664.1|
conserved hypothetical protein [M...    44  
0.001

gi|56680462|gb|AAV97128.1|
thiamine biosynthesis protein ThiS [S...   
44   0.002

gi|41409997|ref|NP\_962833.1|
sulfur carrier protein ThiS [Mycoba...   
44   0.002

gi|49530467|emb|CAG68179.1|
C-terminally thiocarboxylated form i...   
44   0.002

gi|93005120|ref|YP\_579557.1|
protein of unknown function UPF0125...   
44   0.002

gi|83312024|ref|YP\_422288.1|
hypothetical protein amb2925 [Magne...   
44   0.002

gi|28808050|dbj|BAC61287.1|
ThiS protein [Vibrio parahaemolyticu...   
44   0.002

gi|7227333|gb|AAF42389.1|
thiamine biosynthesis protein ThiS [Ne...   
44   0.002

gi|21222559|ref|NP\_628338.1|
molybdopterin converting factor [St...   
44   0.002

gi|94677055|ref|YP\_588522.1|
thiamine biosynthesis protein ThiS ...   
44   0.002

gi|67939265|ref|ZP\_00531772.1|
ThiS, thiamine-biosynthesis [Chlo...   
44   0.002

gi|18893610|gb|AAL81606.1|
hypothetical protein [Pyrococcus furi...   
44   0.002

gi|67875153|ref|ZP\_00504638.1|
ThiS, thiamine-biosynthesis [Clos...   
44   0.002

gi|39982776|gb|AAR34235.1|
moaD family protein [Geobacter sulfur...   
43   0.002

gi|53754222|emb|CAH15699.1|
hypothetical protein [Legionella pne...   
43   0.002

gi|6460589|gb|AAF12295.1|
conserved hypothetical protein [Deinoc...   
43   0.002

gi|77917953|ref|YP\_355768.1|
thiamine biosynthesis protein ThiS ...   
43   0.003

gi|1723343|sp|P51344|YCF40\_PORPU
Hypothetical 8.1 kDa protein yc...   
43   0.003

gi|82499276|ref|ZP\_00884724.1|
hypothetical protein CsacDRAFT\_13...   
43   0.003

gi|17428441|emb|CAD15128.1|
conserved hypothetical protein [Rals...   
43   0.003

gi|87121811|ref|ZP\_01077697.1|
molybdenum cofactor biosynthesis ...   
43   0.003

gi|84501020|ref|ZP\_00999255.1|
thiamine biosynthesis protein Thi...   
43   0.003

gi|68547475|ref|ZP\_00587011.1|
ThiS, thiamine-biosynthesis [Shew...   
43   0.003

gi|91228042|ref|ZP\_01262127.1|
sulfur carrier protein ThiS [Vibr...   
43   0.003

gi|88811647|ref|ZP\_01126901.1|
hypothetical protein NB231\_04560 ...   
43   0.003

gi|70606476|ref|YP\_255346.1|
hypothetical protein Saci\_0669 [Sul...   
43   0.003

gi|84393666|ref|ZP\_00992417.1|
sulfur carrier protein ThiS [Vibr...   
43   0.003

gi|83816286|ref|YP\_446147.1|
ThiS family, putative [Salinibacter...   
43   0.004

gi|29337957|gb|AAO75760.1|
thiS protein, involved in thiamine bi...  
 43   0.004

gi|91086189|ref|XP\_971291.1|
PREDICTED: similar to CG10238-PA [T...   
43   0.004

gi|15622711|dbj|BAB66701.1|
68aa long conserved hypothetical pro...   
43   0.004

gi|23335286|ref|ZP\_00120523.1|
COG2104: Sulfur transfer protein ...   
43   0.004

gi|21114375|gb|AAM42419.1|
conserved hypothetical protein [Xanth...   
43   0.004

gi|16331468|ref|NP\_442196.1|
hypothetical protein ssr0102 [Synec...   
43   0.004

gi|33866139|ref|NP\_897698.1|
DUF170 [Synechococcus sp. WH 8102] ...   
43   0.004

gi|77996070|gb|ABB14969.1|
thiamine biosynthesis protein ThiS [C...   
43   0.005

gi|7379114|emb|CAB83663.1|
hypothetical protein NMA0362 [Neisser...   
43   0.005

gi|88858884|ref|ZP\_01133525.1|
putative ThiS protein [Pseudoalte...   
43   0.005

gi|10639813|emb|CAC11785.1|
hypothetical protein [Thermoplasma a...   
43   0.005

gi|86159912|ref|YP\_466697.1|
thiamineS [Anaeromyxobacter dehalog...   
42   0.005

gi|22294532|dbj|BAC08362.1|
ycf40 [Thermosynechococcus elongatus...   
42   0.005

gi|95930037|ref|ZP\_01312777.1|
conserved hypothetical protein [D...   
42   0.005

gi|67986515|gb|EAM74335.1|
ThiamineS [Kineococcus radiotolerans ...   
42   0.006

gi|78169364|gb|ABB26461.1|
ThiS, thiamine-biosynthesis [Synechoc...   
42   0.006

gi|72161447|ref|YP\_289104.1|
ThiS, thiamine-biosynthesis [Thermo...   
42   0.006

gi|9654459|gb|AAF93242.1|
thiS protein [Vibrio cholerae O1 biova...   
42   0.006

gi|42557747|emb|CAF28721.1|
putative molybdopterin biosynthesis ...   
42   0.006

gi|68230363|ref|ZP\_00569547.1|
ThiS, thiamine-biosynthesis [Fran...   
42   0.007

gi|84363527|ref|ZP\_00988104.1|
COG1656: Uncharacterized conserve...   
42   0.008

gi|46202840|ref|ZP\_00052512.2|
COG2022: Uncharacterized enzyme o...   
42   0.008

gi|28199260|ref|NP\_779574.1|
hypothetical protein PD1376 [Xylell...   
42   0.008

gi|34541692|ref|NP\_906171.1|
thiS protein [Porphyromonas gingiva...   
42   0.009

gi|85375395|ref|YP\_459457.1|
thiazole biosynthesis protein [Eryt...   
42   0.009

gi|98663026|dbj|GAA00171.1|
unnamed protein product [Pelotomacul...   
42   0.009

gi|33640199|emb|CAE19734.1|
DUF170 [Prochlorococcus marinus subs...   
42   0.009

gi|71674937|ref|ZP\_00672683.1|
ThiS, thiamine-biosynthesis [Tric...   
42   0.009

gi|53688960|ref|ZP\_00112390.2|
COG1656: Uncharacterized conserve...    42  
0.009

gi|90994535|ref|YP\_537025.1|
hypothetical protein 71 [Porphyra y...   
42   0.010

gi|54018820|dbj|BAD60190.1|
putative sulfur transfer protein [No...   
42   0.010

gi|82499138|ref|ZP\_00884589.1|
ThiS, thiamine-biosynthesis [Cald...   
42   0.011

gi|88938068|ref|ZP\_01143581.1|
conserved hypothetical protein [G...   
42   0.011

gi|83954400|ref|ZP\_00963120.1|
thiamine biosynthesis protein Thi...   
42   0.011

gi|90407317|ref|ZP\_01215503.1|
sulfur carrier protein ThiS [Psyc...   
41   0.011

gi|59802314|ref|YP\_209026.1|
hypothetical protein NGO2006 [Neiss...   
41   0.011

gi|34484090|emb|CAE11086.1|
hypothetical protein [Wolinella succ...   
41   0.011

gi|85702836|ref|ZP\_01033940.1|
hypothetical protein ROS217\_18882...   
41   0.013

gi|89362382|ref|ZP\_01200190.1|
ThiS, thiamine-biosynthesis [Xant...   
41   0.013

gi|106890176|ref|ZP\_01357372.1|
Protein of unknown function DUF8...   
41   0.013

gi|92090350|ref|ZP\_01275216.1|
molybdopterin converting factor s...   
41   0.013

gi|48855085|ref|ZP\_00309245.1|
COG1977: Molybdopterin converting...   
41   0.013

gi|9107520|gb|AAF85145.1|
conserved hypothetical protein [Xylell...   
41   0.013

gi|90574748|ref|ZP\_01231240.1|
hypothetical protein CdifQ\_020018...   
41   0.014

gi|88949024|ref|ZP\_01151642.1|
hypothetical protein HhalDRAFT\_10...   
41   0.014

gi|87198948|ref|YP\_496205.1|
thiamineS [Novosphingobium aromatic...   
41   0.014

gi|51858105|dbj|BAD42263.1|
molybdopterin converting factor smal...   
41   0.015

gi|67938835|ref|ZP\_00531354.1|
ThiS, thiamine-biosynthesis [Chlo...   
41   0.015

gi|60493471|emb|CAH08257.1|
putative thiamine biosynthesis-relat...   
41   0.015

gi|106882327|ref|ZP\_01349730.1|
thiamine biosynthesis protein Th...   
41   0.015

gi|17427119|emb|CAD13639.1|
probable sulfur transfer protein thi...   
41   0.016

gi|46401290|emb|CAF24739.1|
probable threonine-tRNA ligase [Para...   
41   0.016

gi|17741061|gb|AAL43548.1|
thiamin biosynthesis protein ThiG [Ag...   
40   0.019

gi|94501853|ref|ZP\_01308364.1|
hypothetical protein RED65\_02138 ...   
40   0.019

gi|71037825|gb|AAZ18133.1|
conserved hypothetical protein [Psych...   
40   0.019

gi|98660190|dbj|GAA02501.1|
unnamed protein product [Pelotomacul...   
40   0.020

gi|57240562|ref|ZP\_00368511.1|
thiamine biosynthesis protein Thi...   
40   0.022

gi|71837964|ref|ZP\_00677726.1|
conserved hypothetical protein [P...   
40   0.024

gi|87309615|ref|ZP\_01091749.1|
hypothetical protein DSM3645\_0234...   
40   0.025

gi|74019866|ref|ZP\_00690477.1|
Protein of unknown function DUF82...    40  
0.025

gi|33238327|gb|AAQ00393.1|
Sulfur transfer protein [Prochlorococ...   
40   0.026

gi|87119895|ref|ZP\_01075791.1|
thiamine biosynthesis protein Thi...   
40   0.027

gi|50876627|emb|CAG36467.1|
hypothetical protein [Desulfotalea p...   
40   0.027

gi|85859825|ref|YP\_462027.1|
thiS family protein [Syntrophus aci...   
40   0.027

gi|48855687|ref|ZP\_00309845.1|
COG2104: Sulfur transfer protein ...   
40   0.028

gi|91762145|ref|ZP\_01264110.1|
ThiS family protein [Candidatus P...   
40   0.030

gi|74318466|ref|YP\_316206.1|
hypothetical protein Tbd\_2448 [Thio...   
40   0.030

gi|88809116|ref|ZP\_01124625.1|
hypothetical protein WH7805\_05471...   
40   0.031

gi|72122148|gb|AAZ64334.1|
ThiamineS [Ralstonia eutropha JMP134]...   
40   0.032

gi|84713026|ref|ZP\_01020831.1|
ThiS, thiamine-biosynthesis [Pola...   
40   0.035

gi|15618715|ref|NP\_225001.1|
threonyl-tRNA synthetase [Chlamydop...   
40   0.035

gi|86158941|ref|YP\_465726.1|
thiamine biosynthesis protein ThiS ...   
40   0.035

gi|23011959|ref|ZP\_00052165.1|
COG2104: Sulfur transfer protein ...   
40   0.037

gi|49531021|emb|CAG68733.1|
hypothetical protein [Acinetobacter ...   
40   0.040

gi|82744797|ref|ZP\_00907312.1|
ThiS, thiamine-biosynthesis [Clos...   
40   0.040

gi|88798339|ref|ZP\_01113924.1|
putative ThiS protein [Reinekea s...   
39   0.046

gi|33356745|ref|NP\_126807.2|
sulfur carrier protein ThiS [Pyroco...   
39   0.049

gi|94986022|ref|YP\_605386.1|
thiamine biosynthesis protein ThiS ...   
39   0.049

gi|55670077|pdb|1TYG|G
Chain G, Structure Of The Thiazole Syntha...   
39   0.049

gi|52002882|gb|AAU22824.1|
ThiS [Bacillus licheniformis ATCC 145...   
39   0.051

gi|67987927|gb|EAM75712.1|
Threonyl-tRNA synthetase, class IIa [...   
39   0.058

gi|2633522|emb|CAB13025.1|
thiS [Bacillus subtilis subsp. subtil...   
39   0.058

gi|68263525|emb|CAI37013.1|
thiamin biosynthesis ThiS [Corynebac...   
39   0.062

gi|83951776|ref|ZP\_00960508.1|
hypothetical protein ISM\_14475 [R...   
39   0.062

gi|84489151|ref|YP\_447383.1|
hypothetical protein Msp\_0330 [Meth...   
39   0.063

gi|29540944|gb|AAO89888.1|
thiamine biosynthesis protein ThiS [C...   
39   0.064

gi|94490916|ref|ZP\_01298140.1|
hypothetical protein CburD\_010020...   
39   0.067

gi|14424008|sp|O30213|Y022\_ARCFU
Hypothetical protein AF0022 >gi...    38  
0.086

gi|88931718|ref|ZP\_01137411.1|
Threonyl-tRNA synthetase, class I...   
38   0.098

gi|78713252|gb|ABB50429.1|
thiamine biosynthesis protein ThiS [P...   
38   0.12

gi|14591747|ref|NP\_143011.1|
sulfur carrier protein ThiS [Pyroco...   
38   0.12

gi|57159352|dbj|BAD85282.1|
sulfur transfer protein involved in ...   
38   0.13

gi|76802608|ref|YP\_327616.1|
homolog to molybdopterin converting...   
38   0.14

gi|86155194|ref|ZP\_01073360.1|
thiamine biosynthesis protein Thi...   
38   0.14

gi|56416396|ref|YP\_153470.1|
hypothetical protein AM035 [Anaplas...   
38   0.15

gi|46449919|gb|AAS96568.1|
thiamine biosynthesis protein ThiS [D...   
38   0.16

gi|91762114|ref|ZP\_01264079.1|
probable molybdopterin biosynthes...   
37   0.16

gi|83643048|ref|YP\_431483.1|
thiamine biosynthesis protein ThiS ...   
37   0.16

gi|78196898|gb|ABB34663.1|
thiamine biosynthesis protein ThiS [S...   
37   0.16

gi|18145266|dbj|BAB81309.1|
conserved hypothetical protein [Clos...   
37   0.17

gi|20517475|gb|AAM25599.1|
hypothetical protein TTE2469 [Thermoa...   
37   0.18

gi|69953450|ref|ZP\_00640580.1|
ThiS, thiamine-biosynthesis [Shew...   
37   0.18

gi|86741757|ref|YP\_482157.1|
thiamine biosynthesis protein ThiS ...   
37   0.18

gi|736275|emb|CAA56171.1|
unnamed protein product [Pyrococcus fu...   
37   0.21

gi|71367699|ref|ZP\_00658218.1|
ThiS, thiamine-biosynthesis [Noca...   
37   0.23

gi|59410400|gb|AAW84299.1|
putative MaoD-like protein [unculture...   
37   0.25

gi|70607385|ref|YP\_256255.1|
hypothetical protein Saci\_1652 [Sul...   
37   0.25

gi|44920975|emb|CAF30211.1|
hypothetical protein [Methanococcus ...   
37   0.26

gi|88792777|ref|ZP\_01108495.1|
hypothetical protein MADE\_11015 [...   
37   0.26

gi|77996033|gb|ABB14932.1|
thiamine biosynthesis protein ThiS [C...   
37   0.28

gi|57636146|gb|AAW52934.1|
thiamine biosynthesis protein ThiS [S...   
37   0.29

gi|55232699|gb|AAV48118.1|
unknown [Haloarcula marismortui ATCC ...   
37   0.30

gi|46198624|ref|YP\_004291.1|
putative thiS protein [Thermus ther...   
37   0.31

gi|78194032|gb|ABB31799.1|
ThiS, thiamine-biosynthesis [Geobacte...   
37   0.31

gi|85859558|ref|YP\_461760.1|
GTP pyrophosphokinase / guanosine-3...   
37   0.31

gi|54016307|dbj|BAD57677.1|
hypothetical protein [Nocardia farci...   
37   0.32

gi|88934227|ref|ZP\_01139871.1|
Threonyl-tRNA synthetase, class I...   
37   0.32

gi|93454223|gb|EAT04541.1|
ThiS, thiamine-biosynthesis [delta pr...   
37   0.32

gi|76874357|emb|CAI85578.1|
putative ThiS protein [Pseudoalterom...   
37   0.33

gi|20804221|emb|CAD31247.1|
PROBABLE THIAMIN BIOSYNTHESIS PROTEI...   
37   0.34

gi|10174049|dbj|BAB05151.1|
BH1432 [Bacillus halodurans C-125] >...   
37   0.35

gi|82498768|ref|ZP\_00884224.1|
conserved hypothetical protein [C...   
36   0.39

gi|77812832|ref|ZP\_00812115.1|
ThiS, thiamine-biosynthesis [Shew...   
36   0.41

gi|62527990|ref|ZP\_00389252.1|
COG0564: Pseudouridylate synthase...   
36   0.49

gi|66964609|ref|ZP\_00412178.1|
ThiS, thiamine-biosynthesis [Arth...   
36   0.49

gi|55738184|gb|AAV61825.1| ribosomal
large subunit pseudouridine...    36   0.49

gi|2494622|sp|Q57557|Y092\_METJA
Putative iron-sulfur protein MJ0...   
36   0.51

gi|68542219|ref|ZP\_00581954.1|
ThiS, thiamine-biosynthesis [Shew...   
36   0.51

gi|88657629|ref|YP\_507028.1|
thiamine biosynthesis protein ThiS ...   
36   0.52

gi|30468123|ref|NP\_849010.1|
ORF67 [Cyanidioschyzon merolae stra...   
36   0.54

gi|32397914|emb|CAD73915.1|
conserved hypothetical protein [Rhod...   
36   0.55

gi|41410171|ref|NP\_963007.1|
hypothetical protein MAP4073 [Mycob...   
36   0.56

gi|71839361|ref|ZP\_00679111.1|
Threonyl-tRNA synthetase, class I...   
36   0.60

gi|66967317|ref|ZP\_00414858.1|
ThiamineS [Arthrobacter sp. FB24]...   
35   0.62

gi|78193878|gb|ABB31645.1|
Threonyl-tRNA synthetase, class IIa [...   
35   0.65

gi|13879933|gb|AAK44653.1|
conserved hypothetical protein [Mycob...   
35   0.68

gi|71416009|ref|XP\_810051.1|
hypothetical protein [Trypanosoma c...   
35   0.69

gi|51588192|emb|CAH19799.1|
hypothetical protein [Yersinia pseud...   
35   0.69

gi|83312026|ref|YP\_422290.1|
hypothetical protein amb2927 [Magne...   
35   0.71

gi|72002530|gb|AAZ58332.1|
ThiS, thiamine-biosynthesis [Prochlor...   
35   0.74

gi|66963966|ref|ZP\_00411537.1|
ThiamineS [Arthrobacter sp. FB24]...   
35   0.76

gi|58617523|ref|YP\_196722.1|
hypothetical protein ERGA\_CDS\_07960...   
35   0.78

gi|72394552|gb|AAZ68829.1|
putative ThiS sulfur transfer protein...   
35   0.79

gi|71652006|ref|XP\_814668.1|
hypothetical protein [Trypanosoma c...   
35   0.84

gi|13880123|gb|AAK44832.1|
conserved hypothetical protein [Mycob...   
35   0.85

gi|86157547|ref|YP\_464332.1|
molybdopterin biosynthesis MoaE / t...   
35   0.90

gi|57166737|gb|AAW35516.1|
thiamine biosynthesis protein ThiS [C...   
35   0.92

gi|10581690|gb|AAG20393.1|
Vng2279h [Halobacterium sp. NRC-1] >g...   
35   0.93

gi|57168917|ref|ZP\_00368047.1|
thiamine biosynthesis protein Thi...   
35   0.98

gi|13540947|ref|NP\_110635.1|
hypothetical protein TVN0116 [Therm...   
35   1.0

gi|2909638|emb|CAA17450.1|
CONSERVED HYPOTHETICAL PROTEIN [Mycob...   
35   1.1
[truncated: 432,277 more chars]
